# Enhanced Selectivity of Chalcogen Bonding over Halogen Bonding Catalyzed *C*‐glycosylation Through Differentiated Intermediate Activation

**DOI:** 10.1002/anie.202517553

**Published:** 2025-11-13

**Authors:** Hao Guo, Charles C. J. Loh

**Affiliations:** ^1^ College of Chemistry and Materials Science Guangdong Provincial Key Laboratory of Functional Supramolecular Coordination Materials and Applications Jinan University Guangzhou 510632 P.R. China; ^2^ UCD School of Chemistry University College Dublin Belfield, Dublin 4 Dublin Ireland

**Keywords:** Chalcogen bonding, Glycosylation, Noncovalent interactions, Organocatalysis, Reaction mechanisms

## Abstract

σ‐hole‐based noncovalent interactions are gaining intense attention as robust tools in stereoselective carbohydrate synthesis. However, the mechanistic understanding behind the differing performance between chalcogen bonding (ChB) catalysis versus halogen bonding (XB) catalysis in chemical glycosylations still remains unresolved. Herein, we disclose a remarkable instance whereby phosphonochalcogenide (PCH) catalysis displays pronounced selectivity and reactivity elevation in aryl‐*C*‐glycosylations compared to XB catalysis. Mechanistic studies revealed that the enhanced stereocontrol can be attributed to the differentiated downstream activation of a key bicyclic intermediate. Hammett analysis supported that ChB catalysis shifted the *C*‐glycosylation toward the S_N_1 domain, while the XB catalysis proceeded with S_N_2 characteristics. DFT calculations further illuminated that the downstream ChB catalytic engagement involved all four selenium σ‐holes engaging in two bifurcated ChB modes. This study thus sheds new light that the selectivity benefits of ChB catalysis could be accounted for by privileged mechanistic access into an otherwise inaccessible stereoselective pathway.

## Graphical abstract

By harnessing chalcogen bonding (ChB) catalysis, an exclusive access into a highly stereoselective strain‐release *C*‐glycosylation pathway was discovered. This was surprisingly not replicable by the conceptually similar halogen bonding (XB) catalysis. Mechanistic investigation revealed that the stereoselectivity divergence can be traced to the privileged ChB activation of a downstream bicyclic intermediate by a phosphonochalcogenide (PCH) catalyst.

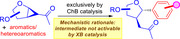

## Introduction

**Figure 1:**
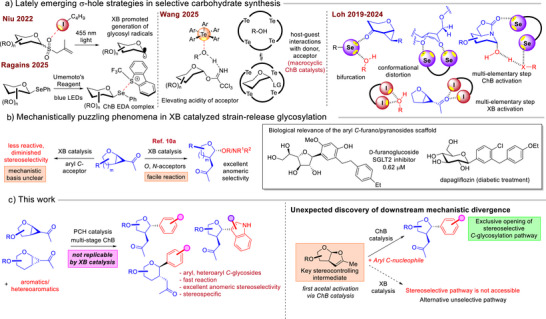
Emerging σ‐hole‐based catalytic glycosylation strategy and mechanistic divergence between different types of σ‐hole catalysts. a) Lately reported glycosylation strategies using the emerging σ‐hole catalytic strategy; b) mechanistic enigma in XB catalyzed *O*,*N*‐glycosylations versus aryl‐*C*‐glycosylations; c) this work.

The harnessing of noncovalent interactions (NCIs) is broadly recognized as a vital strategy in stereoselective synthesis.^[^ ^1^, ^2^, ^3^, ^4^, ^5^, ^6^, ^7^ ^]^ Lately, there is a strong momentum in the investigation of nonclassical weak interactions to access favorable pathways in stereoselective glycosylations.^[^ ^8^, ^9^, ^10^ ^]^ Of recent immense focus is a family of unconventional NCIs known as σ‐hole‐based interactions,^[^ ^11^, ^12^, ^13^, ^14^ ^]^ such as halogen bonding (XB)^[^ ^15^, ^16^, ^17^ ^]^ and chalcogen bonding (ChB),^[^ ^18^, ^19^, ^20^ ^]^ which have emerged lately as a formidable catalytic tool to gain entry into difficult‐to‐access glycosidic chemical space. Besides the classical electrostatic component that is endowed in the σ‐hole definition, it is crucial to appreciate that such NCIs also involve other components such as polarizability, orbital delocalization, dispersion, alongside charge transfer.^[^ ^20^, ^21^ ^]^ Notably, rapidly emerging contributions such as those by the group of Niu in halogen bonding (XB) promoted radical glycosylations,^[^ ^22^ ^]^ by Wang in telluronium based chalcogen bonding (ChB) catalyzed glycosylations,^[^ ^23^, ^24^ ^]^ lately by Ragains in ChB mediated photoredox glycosylations,^[^ ^25^ ^]^ and those from our group in XB^[^ ^26^, ^27^ ^]^ and ChB^[^ ^28^, ^29^, ^30^, ^31^ ^]^ catalyzed glycosylations had shaped the current broad significance of σ‐hole based interactions in the carbohydrate chemistry realm (Figure 1a).^[^ ^32^, ^33^ ^]^

Moving beyond early elegant proof‐of‐concept cases by Huber^[^ ^34^ ^]^ and Takemoto^[^ ^35^, ^36^ ^]^ using XB promoters or co‐catalysts in glycosylations, our research group had been active in advancing and conceptualizing the σ‐hole based catalytic strategy in stereoselective carbohydrate synthesis.^[^ ^32^ ^]^ In our seminal contributions in 2019,^[^ ^26^ ^]^ we showcased an intriguing early instance of an exclusively halogen bonding (XB) catalyzed *O*,*N*‐strain‐release glycosylation where dynamic multistage reversible X···O σ‐hole activation in multiple elementary steps was found to be operative. The elevation of anomeric selectivity compared to our previously thiourea catalyzed *O*‐glycosylation case^[^ ^37^ ^]^ was a salient feature whereby differentiated selectivity benefits could arise by utilizing more directional XB interactions. Emerging contributions also showcased that catalytic access of difficult *N*,*N*‐dimethylamino sugars—a problematic reaction that classically required large excess of stoichiometric promoters—can be realized by XB catalysis.^[^ ^38^ ^]^ We have lately reported several cases whereby the usage of Wang's versatile phosphonochalcogenide (PCH)^[^ ^39^, ^40^, ^41^, ^42^, ^43^, ^44^, ^45^ ^]^ catalysts paved out counter‐intuitive ChB mechanistic pathways, facilitating the stereoselective synthesis of septanosides,^[^ ^29^, ^30^ ^]^ indolyl glycosides^[^ ^28^ ^]^ and iminoglycosides.^[^ ^31^ ^]^ Unconventional mechanistic manifolds that arose include bifurcated ChB,^[^ ^29^ ^]^ intramolecular aglycone transposition,^[^ ^30^ ^]^ conformational distortion^[^ ^28^ ^]^ and multi‐elementary step Se···O ChB activation.^[^ ^31^ ^]^ Unexpectedly, the late success of ChB catalyzed transformations was generally not replicable using XB catalysis despite being similarly endowed with σ‐hole donor elements. These results inevitably led us to a fundamentally unresolved question: *Why are there differentiated glycosylation outcomes as a consequence of using different σ‐hole donors?*

An interesting development appeared in a lately published report by Wang,^[^ ^23^ ^]^ where the authors described unusual site‐selectivity in Schmidt glycosylations when a novel σ‐hole donor was employed. Notwithstanding the important insights uncovered, a noncovalent mechanistic rationale explaining why ChB catalysis displays benefits over other σ‐hole modes such as XB catalysis in improving anomeric selectivity and glycosylation reactivity remains an enigma.

Motivated by our earlier puzzling observation that XB‐catalyzed strain‐release glycosylation^[^ ^26^ ^]^ was only effective for *O*‐ and *N*‐nucleophiles, but not *C*‐aryl nucleophiles (Figure 1b), we were driven to reinvestigate if the reaction outcome would differ in light of fascinating advances in ChB catalysis.^[^ ^39^ ^]^ Inspired by lately emerging aryl‐*C*‐glycosylation strategies using radical catalysis^[^ ^46^, ^47^, ^48^ ^]^ elegantly spearheaded by the groups of Koh,^[^ ^49^ ^]^ Niu,^[^ ^50^ ^]^ and Zhu,^[^ ^51^, ^52^ ^]^ as well as the attention surge in accessing nonclassical sugars,^[^ ^53^, ^54^ ^]^ we were eager to study whether a σ‐hole strategy toward aryl‐*C*‐furano and pyranosides would be feasible. Further, molecules bearing aryl *C*‐furano/pyranosides privileged scaffolds^[^ ^55^ ^]^ possess broad biological profiles such as anti‐diabetic activity (Figure 1b, right panel),^[^ ^56^, ^57^ ^]^ and are hydrolytically more stable than the *O*‐glycosidic linkage.^[^ ^58^, ^59^, ^60^ ^]^ Particularly, the rare access of 2C‐branched versions of *C*‐glycosides would be synthetically valuable,^[^ ^61^, ^62^ ^]^ as such scaffolds are found in antibiotics and natural products. 2C‐branched sugars containing ketones are also mimics of 2‐*N*‐acetylsugars, which offer a synthetic handle for bioconjugation.^[^ ^63^ ^]^ However, very limited methods are known to access them. Our previous report also disclosed that novel anticancer‐relevant bioactivity was endowed in 2C‐branched glycosides obtained through strain‐release glycosylation.^[^ ^26^ ^]^

Herein, we present a highly stereospecific and anomeric selective *C*‐aryl‐strain‐release furano‐ and pyranosylation through the exclusive employment of ChB catalysis (Figure 1c). In direct contrast, we noted that our XB catalyzed method consistently gave diminished stereoselectivity and yields over a broad array of Friedel–Crafts type aryl nucleophiles (see Supporting Information; Table S5). By isolating and performing control experiments on a vital bicyclic intermediate, we bring forth new insights that the divergent glycosylation outcomes can be traced to the differentiated catalytic activation between ChB and XB donors in a downstream glycosylation step. Hammett analysis additionally supports the hypothesis that ChB catalysis played a key role in shifting the mechanism toward the S_N_1 segment on the S_N_1‐S_N_2 glycosylation continuum.^[^ ^64^ ^]^ Computations further unveiled that the downstream ChB catalytic activation likely involved all four σ‐holes on the PCH catalyst participating in two bifurcated ChB modes with the intermediate and nucleophile. It is worthwhile to emphasize that our study demonstrates the first instance of a direct ChB activation of acetals—an important moiety in carbohydrate synthesis that was previously not known to be activable via ChB methods.^[^ ^41^, ^42^, ^45^, ^65^ ^]^ Our present study thus unveils seminal insights that ChB catalysis can facilitate mechanistic entry into stereoselective pathways that are unavailable to alternative σ‐hole‐based catalytic modes.

## Results and Discussion

### Establishment of the Exclusively PCH Catalyzed α‐Selective *C*‐Strain‐Release Glycosylation

We began our investigation by evaluating a broad panel of noncovalent catalysts on a prototypical strain‐release glycosylation^[^ ^26^, ^29^, ^30^, ^37^, ^66^, ^67^, ^68^, ^69^, ^70^ ^]^ of a cyclopropanated glycosyl donor^[^ ^71^, ^72^, ^73^, ^74^, ^75^ ^]^ **1a** with 1,3,5‐trimethoxybenzene (TMB) **2a** as the *C*‐acceptor (Table 1). We noted that the widely used Schreiner's thiourea catalyst **A** ^[^ ^76^, ^77^ ^]^ was ineffective in activating the glycosylation. Attempts at boosting HB strength/Brønsted acidity using the charged Kass's thiourea catalyst **B** ^[^ ^37^, ^78^ ^]^ were sluggish and only proceeded under prolonged heating conditions, albeit with unsatisfactory anomeric selectivity. Further study of previously well‐performing XB donors^[^ ^79^, ^80^ ^]^ in strain‐release glycosylations also revealed that only the well‐known Huber's *bis*‐imidazolinium salt **C** ^[^ ^79^ ^]^ gave acceptable yields, although the anomeric selectivity remained unsatisfactory at 3.6:1.

**Table 1:** Reaction optimization.

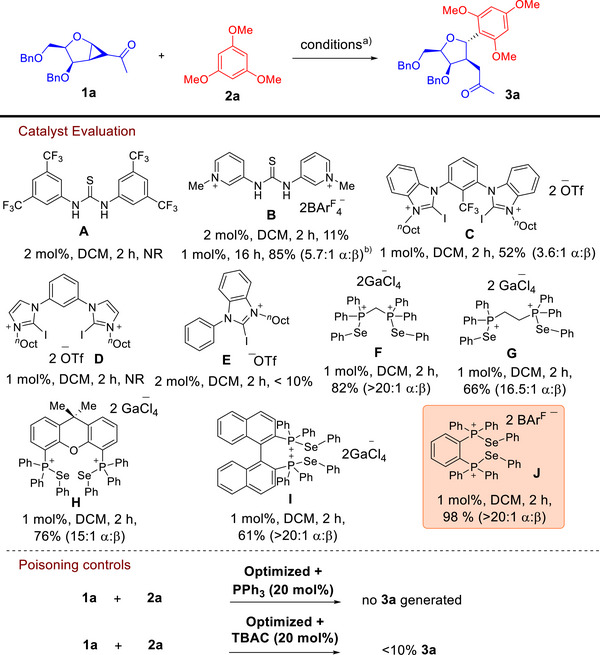

Conditions: **1a** (0.1 mmol), **2a** (0.15 mmol), catalyst (1–2 mol%), 1 mL solvent, 2 h, rt. ^a)^Yields and α:β ratios were determined by crude ^1^H NMR spectra analysis using 1,1,2,2‐tetrachloroethane as an internal standard. ^b)^Toluene, LiClO_4_ (1 equiv) additive, 80 °C instead. NR = no reaction.

When we subsequently turned to a family of PCH catalysts **F–J**,^[^ ^39^ ^]^ we noted distinctive improvements in reactivity and substantial elevation in anomeric selectivity. Observation of marginal fluctuations in stereoselectivity between 15:1 to >20:1 over different phosphine scaffolds suggested that electronics and spacer distance between both seleniums could serve as a fine‐tuner to impact yields and selectivity outcome. We eventually arrived at the optimal 1,2‐*bis*(diphenylphosphino)benzene derived PCH catalyst **J**,^[^ ^43^ ^]^ which yielded the *C*‐glycoside with almost quantitative yields and excellent anomeric selectivity. It is also worthy to emphasize that this reaction worked at ambient conditions without any efforts to exclude moisture, showcasing its synthetic practicability. Further, diagnostic σ‐hole poisoning experiments^[^ ^81^, ^82^, ^83^ ^]^ were conducted and led to reaction termination through the use of phosphine (PPh_3_) and halide (tetrabutyl ammonium chloride, TBAC) additives (Table 1). This observation underscores the critical involvement of ChB activation in our *C*‐strain‐release glycosylation.

Equipped with the optimal catalytic conditions, we proceeded to examine the substrate scope of the reaction with respect to the glycosyl donor (Table 2). We delightfully observed that the ChB catalytic activation is amenable over a remarkable range of furanosyl cyclopropanated donors^[^ ^84^ ^]^ such as d,l‐Xyl*f*, d‐Ara*f* derived ones, as well as pyranosyl donors^[^ ^85^, ^86^ ^]^ such as glucosyl and galactosyl derivatives. In all of these cases, the *C*‐glycosylation yielded consistently excellent anomeric selectivity (>20:1) in a stereospecific fashion. Hence, the resulting α/β‐stereochemistry is the inverted configuration from that of the glycosyl donor. d,l‐Xyl*f‐*derived substrates gave α‐*C*‐glycosides, while d‐Ara*f*, d‐glucose, and d‐galactose‐derived substrates yielded solely β‐*C*‐glycosides. Reactions were also extremely facile, as some *C*‐glycosides were formed in as fast as 10 min, and most instances in the substrate scope were completed within 1 h. Additionally, we noted that this strategy nicely accommodates various modifications on the ether substituents such as benzyl, allyl, and proparyl groups, which offer additional synthetic handles for downstream functional group modifications.

**Table 2:** Strain‐release *C*‐glycosylation substrate scope by modifying cyclopropanated glycosyl donors.

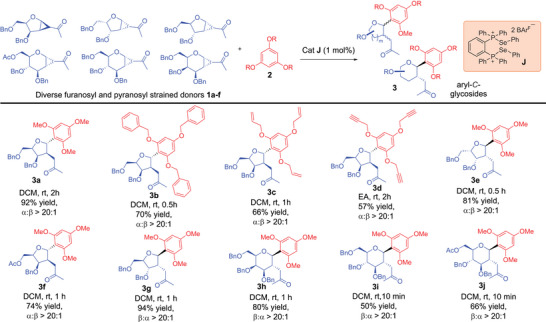

General Conditions: **1a–f** (0.1 mmol), 1.5 equiv **2** (0.15 mmol), cat. **J** (1 mol%), 1 mL solvent was used. Yields reported are isolated yields after flash column chromatography. α:β ratios were determined by crude ^1^H NMR spectra analysis.

In light of the known biological importance of indolyl‐glycosides,^[^ ^55^, ^87^ ^]^ as well as the late emergence of the pseudo‐natural product concept^[^ ^88^, ^89^, ^90^, ^91^ ^]^ in chemical biology where the merging of privileged scaffolds is well recognized as valuable to access novel chemical space with disease relevance, we successfully expanded our ChB catalyzed strategy toward a broad range of *C*‐glycosides appended to heteroaromatics (Table 3).

**Table 3:** Strain‐release *C*‐glycosylation substrate scope by modifying *C*‐glycosyl acceptors.

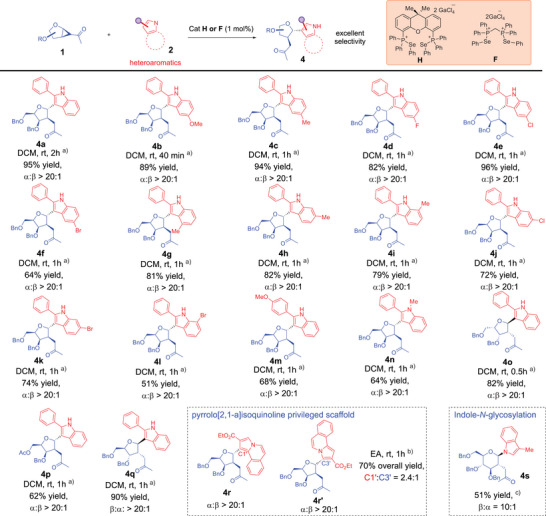

General conditions: ^a)^ **1** (0.1 mmol), 1.5 equiv **2** (0.15 mmol), cat. **H** (1 mol%), 1 mL DCM was used. ^b)^cat. **F** (1 mol%) and 1 mL ethyl acetate used instead. ^c)^cat. **J** (1 mol%), rt, 6 h, 3‐methylindole used as nucleophile instead. All yields reported are isolated yields after flash column chromatography. α:β ratios were determined by crude ^1^H NMR spectra analysis.

The modularity of PCH catalysis^[^ ^39^ ^]^ provided versatility, as tuning of the phosphine scaffold toward catalyst **H** and **F** facilitated optimal yields over a range of heterocyclic nucleophiles, although **J** still performed robustly (see Supporting Information; Table S2). Further preservation of excellent anomeric selectivity (Table 3) along with optimal yields was observed. It is worthwhile emphasizing that the reaction is extremely facile, with most of the examples completing within 1h. We noted, however, that an aryl‐substitution on the indole ring is crucial, and reaction trials on non‐arylated indoles had been ineffective (see Supporting Information; Table S4). We hypothesize that the aryl group might be crucial in stabilizing the developing positive charge on the heteroaromatic indole ring in the Wheland‐type intermediate during the Friedel–Crafts type reaction.

Considering recent attention in accessing the pyrrolo[2,1‐a]isoquinoline privileged scaffold^[^ ^92^, ^93^, ^94^, ^95^ ^]^ that is endowed in disease relevant molecules, we further tested such heterocycles and noted that both C1 (**4r**) and C3 (**4r′**) regioisomers can be accessed with excellent anomeric selectivity. Interestingly, our strategy is also amenable on the C3‐blocked 3‐methylindole substrate to yield *N*‐glycoside **4s** with good β‐selectivity. In parallel, we conducted the *C*‐strain‐release glycosylation on a representative panel of cyclopropanated donor and acceptor substrates using the versatile Huber's XB catalyst **C** to understand the impact of switching the σ‐hole donor element (see Supporting Information; Table S5). This switch interestingly resulted in a clear diminishment of both anomeric selectivity and reaction yields. It is even worthwhile to mention that for a series of pyranosyl donors bearing arming and disarming protecting groups, the XB strategy was incapable in activating the *C*‐glycosylation, and no reaction occurred.

### Mechanistic Study

**Figure 2:**
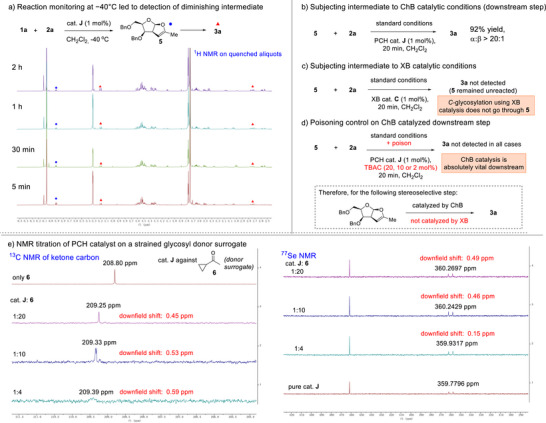
Monitoring experiments, control experiments and NMR titrations. a) ^1^H NMR reaction monitoring on quenched aliquots at different timepoints; b) subjecting intermediate **5** to standard ChB catalytic conditions; c) subjecting **5** to XB catalytic conditions; d) poisoning control on reaction conditions in (b); e) NMR titrations of cat. **J** versus surrogate donor **6**.

In an effort to seek a mechanistic rationale that had led to the different reaction outcomes across σ‐hole donors, we first conducted a ^1^H NMR monitoring experiment at −40 °C (Figure 2a) by withdrawing and quenching aliquots at time intervals, as the facile reaction at RT was too fast for NMR analysis. Importantly, we detected a bicyclic intermediate **5** that we previously observed in XB‐catalyzed *O*‐strain‐release glycosylations.^[^ ^26^ ^]^ Since **5** was previously found to be critical in the stereoselective forging of the C‐O glycosidic linkage, our initial hypothesis was that **5** could be a vital stereoselectivity‐enforcing intermediate in *C*‐glycosylations. From this branching point, we reasoned that the *C*‐glycosylation pathway may diverge depending on different catalytic modes employed.

We thus isolated intermediate **5** and subjected **5** separately to the optimized PCH (Figure 2b) and XB catalyzed (Figure 2c) conditions. Surprisingly, we noted that the PCH catalyzed conditions cleanly converted **5** into the *C*‐glycoside with excellent anomeric selectivity while no conversion occurred using XB donor **C**. This unexpected observation suggested that the mechanistic route taken by the PCH catalyst in *C*‐glycosylation differentiates from XB catalysis by the exclusive activation of **5** in the stereoselective downstream glycosylation step. A further ^1^H NMR control experiment that subjected β‐*C*‐glycoside **3h** to the standard ChB catalyzed protocol revealed that no β‐ to α‐anomerization is operative under the catalytic conditions (see Supporting Information; Figure S28).

We further performed the ChB catalyzed downstream step in the presence of different amounts of the σ‐hole poison^[^ ^81^, ^82^, ^83^ ^]^ TBAC (Figure 2d) using our optimized conditions. Employing 2, 10, or 20 mol% of TBAC did not result in any *C*‐glycosylation in all cases (see Supporting Information; Figure S11). This control experiment ascertained that this downstream elementary step required the vital participation of ChB interactions.

To better understand the noncovalent engagements between the catalyst and the substrates, we first conducted a NMR titration (Figure 2e) between a surrogate cyclopropyl ketone **6** and the ChB catalyst owing to the instability of the cyclopropyl carbohydrate **1** under NMR titration conditions with the catalyst—an observation which is consistent with our prior report with XB catalysis.^[^ ^26^ ^]^ Concomitant chemical shift perturbations on both the ^13^C NMR (0.59 ppm downfield, ketone resonance) and ^77^Se NMR (0.49 ppm downfield, selenium resonance) suggest that Se···O catalytic activation is operative in the upstream glycosyl donor activation step. Attempts at titrating **5** with the PCH catalyst to understand the downstream catalyst‐intermediate supramolecular interactions were unsuccessful (see Supporting Information; Figure S26) due to the lability of the intermediate, which led to decomposition in the NMR tube.

**Figure 3:**
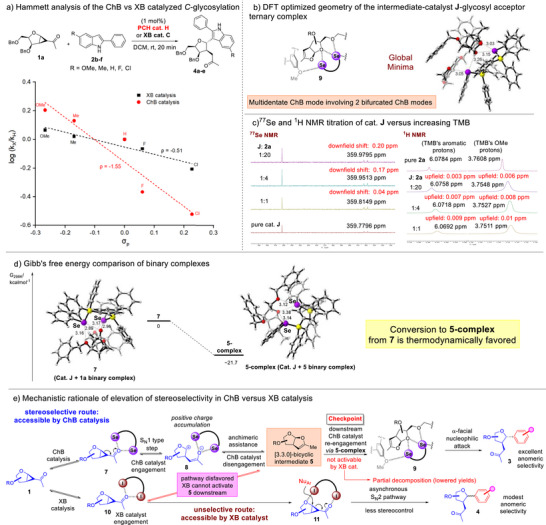
Hammett analysis, DFT analysis of NCI activation in the downstream step and proposed mechanism. a) Hammett plot to compare the linear free energy relationships of the ChB versus XB catalyzed *C*‐strain‐release glycosylation; b) CYLView 2.0 render of the DFT optimized ternary complex in the downstream ChB catalyzed step. Distances corresponding to bifurcated ChBs (in Å) are labeled beside dotted lines; c) ^77^Se and ^1^H NMR titrations of PCH catalyst **J** versus increasing TMB that support the DFT model; d) computed relative Gibb's free energies and DFT optimized geometries for global minima of binary complexes **7** and **5‐complex**. (NCIs denoted using dotted lines and distances in Å were labeled. Atom colors: purple = selenium, yellow = phosphorus, gray = carbon, white = hydrogen); e) Proposed mechanistic rationale of stereoselectivity elevation using ChB catalysis over XB catalysis through differentiated intermediate **5** activation.

Seeking deeper mechanistic insights, we performed a Hammett analysis^[^ ^96^, ^97^, ^98^ ^]^ for the model reaction between donor **1a** and indoles **2b–f** with different electron donating and withdrawing substituents for both the ChB and the XB catalyzed reaction (Figure 3a). The Hammett plot revealed that the ChB catalyzed case had a steeper and negative slope (*ρ* = −1.55), while the XB case displayed diminished sensitivity of the reaction rate to substituent effects (*ρ* = −0.51). This supports the postulate that when PCH catalysis was employed, a pathway of appreciable build‐up of defined positive charges in the rate limiting transition state was operative, and the reaction shifted toward the S_N_1 end of the glycosylation spectrum.^[^ ^85^ ^]^ On the other hand, the comparatively insensitiveness toward electronic effects of the XB catalyzed case is indicative of an asynchronous S_N_2 character with marginal dissociative characteristics. This constitutes a pathway in between the S_N_1‐S_N_2 extremes, where the S_N_2‐like reaction has a faster bond breaking than the bond forming process, creating temporal positive charges (partial S_N_1 character) on the anomeric carbon. These are also congruent with our working hypothesis that PCH catalysis led to the exclusive opening of the stereoselective pathway via intermediate **5**, while XB catalysis plausibly involved a mechanistic detour toward an unselective asynchronous S_N_2 pathway directly from the cyclopropyl donor, without the involvement of anchimeric assistance.

Since intermediate **5** was not amenable toward NMR titration, we modeled the ternary complex between the PCH catalyst **J**, intermediate **5** and TMB to glean insights regarding the supramolecular activation mode (Figure 3b). First, we sampled the vast conformational landscape using the robust GOAT^[^ ^99^ ^]^ conformational search algorithm implemented in ORCA^[^ ^100^ ^]^ at the GFN2‐XTB/ALPB(CH_2_Cl_2_) level of theory.^[^ ^101^, ^102^, ^103^, ^104^ ^]^ As Minnesota functionals^[^ ^105^ ^]^ had been used successfully in describing PCH^[^ ^44^ ^]^ and XB^[^ ^106^ ^]^ catalysts, we refined the global minima from the GOAT conformational sampling at the M06‐2X‐D3(0)/def2‐TZVP/CPCM(CH_2_Cl_2_) level of theory using ORCA.^[^ ^100^ ^]^ The wavefunction of well converged geometries were then subjected to Lu's IGMH (Independent Gradient Model based on Hirshfeld Partition) analysis^[^ ^107^ ^]^ to unravel the interfragment NCIs through colored isosurfaces using the open source Multiwfn package.^[^ ^108^, ^109^ ^]^

Intriguingly, the DFT optimized structure **9** revealed that both seleniums of the PCH catalyst were well positioned to engage in four Se···O interactions through two concurrent bifurcated ChB modes: One that involved the sugar's endocyclic oxygen and the methoxy's oxygen on TMB; and a second one that involved the same endocyclic oxygen with the O5 of the sugar. The calculated ChB distances are further in line with the magnitude of previously reported examples involving PCH catalysts.^[^ ^28^, ^31^, ^44^, ^45^ ^]^ The presence of these NCIs is further reinforced by IGMH analysis (see Computational Section, Supporting Information; Figure S29). Moreover, this activation model is supported by contemporaneous ^77^Se NMR and ^1^H NMR titrations when catalyst **J** was titrated against increasing TMB (Figure 3c). In the ^77^Se NMR titration, a downfield shift on the catalyst's selenium resonances (∼0.2 ppm) was observed. On the other hand, simultaneous upfield shifts were also observed on both TMB's aromatic protons as well as methyl protons on the TMB's methoxy group. These NMR chemical shift perturbations support the Se···O interaction between the catalyst's selenium and the etheric oxygen on TMB that was revealed in the DFT model of **9**. Thus, the DFT analysis, the NMR titration data together with the phosphine poisoning of the downstream ChB catalytic step (Figure 2d, vide supra) collectively strengthen the postulate that a compact multidentate bifurcated mode depicted in **9** is likely responsible for the excellent stereoselectivity and reactivity. Further, the observation that 2 mol% TBAC was already sufficient for a complete downstream poisoning reaction supports the postulate that the effective operation of all 4 selenium σ‐holes in ternary complex **9** is critical for the progression of the stereoselectivity pathway. On the contrary, such a multidentate mode cannot be enforced similarly by XB catalyst **C** due to the reduced amount of operative σ‐holes inherent in halogens.

Since our Hammett analysis suggested an asynchronous S_N_2 mode for XB catalysis, we further modeled the cyclopropanated donor‐catalyst **C**‐TMB ternary complex **11** by subjecting its global minimal identified by the GOAT conformational search to the above‐mentioned DFT workflow. Interestingly, IGMH analysis revealed that the energetically lowest conformer enforced a bifunctional XB activation mode (see Computational Section, Supporting Information; Figure S30), whereby one halogen is establishing an I···O interaction with the ketone oxygen, while the other halogen is engaged with an I···O interaction with the endocyclic oxygen of the sugar. This sparse geometry distinctively differs from **9** in that the region in the vicinity of the anomeric carbon is considerably more spacious for nucleophilic attacks on both faces. This hence suggests that putative nucleophilic attack paths leading to the α‐ or β‐*C*‐glycosides were relatively unhindered, and could provide a basis for why the XB catalyzed S_N_2 manifold led to diminished selectivities.

We further computed the geometries and the relative Gibb's free energies of the binary complexes **7** and **5‐complex** to better understand the catalytic influences on the ChB catalyzed pathway (Figure 3d). We observed that the Gibb's free energy of **5‐complex** is 21.7 kcal mol^−1^ more stable than **7**. This supports the postulate that the equilibration between **7** and **5** favors **5‐complex** formation. Since **5‐complex** is the binary precursor that culminates in ternary complex **9** formation, this also indirectly strengthens the thermodynamic favorability of **9** generation in the exergonic pathway. Despite our repeated attempts to model the zwitterionic glycosyl cation binary complex **8**, we were however not successful due to the well‐known challenge of ion‐pair recombination of glycosyl ion‐pair intermediates.^[^ ^110^ ^]^ Evaluating the C─O bond distance in **5‐complex** through a relaxed surface scan as well as the growing string method^[^ ^111^, ^112^ ^]^ did not provide any noticeable first order saddle points on the potential energy surface (PES), suggesting that **7** to **5‐complex** conversion may occur through a (quasi)barrierless transformation.^[^ ^113^ ^]^ Furthermore, despite meticulous attempts to locate the transition state of the nucleophilic attack from **9** using the fore‐mentioned strategies, we were not able to converge upon a reasonable transition state. We surmise that the unique dicationic nature of the PCH catalysts could lead to DFT modelling deficiencies, as it is known that charged systems could result in charge delocalization error and spin contamination that may distort the PES in the transition state search.^[^ ^114^ ^]^ It is worthwhile to appreciate the versatility of σ‐hole engagements in different binary complexes as double bifurcated ChB is operative in **7**, while bidentate and bifurcated ChB engagements occur in **5‐complex**. This showcases the capacity of the multiple σ‐holes in PCH catalysts to flexibly engage oxygenated moieties on glycosyl substrates and intermediates in different geometric orientations.

Conglomerating the experimental and computational insights, we propose the following working hypothesis for the mechanism (Figure 3e): First, the usage of PCH catalysis paved the access of a stereoselective route that involved more pronounced S_N_1 type character. The multi‐staged ChB^[^ ^31^ ^]^ engagement mode begins with a bidentate ChB activation of the ketone in **7**, which resulted in the C─C bond cleavage on the cyclopropane. This leads to localization of positive charges on the anomeric center, together with stabilization of the negative charge on the oxyanion by the PCH catalyst in **8**. A key stereocontrolling [3.3.0]‐bicyclic intermediate **5** will then form as a result of anchimeric assistance,^[^ ^115^ ^]^ alongside with the ChB catalyst disengagement. The mechanistically diverging “checkpoint” occurs with intermediate **5**. The PCH catalyst which is capable of activating **5** through a network of bifurcated ChB interactions in **9** forged the path into a highly stereoselective attack of the *C*‐glycosyl donor, which led to excellent reactivity and stereoselectivity of the final 2C‐branched *C*‐glycosides **3–4**. It is unlikely that the dual bifurcated ChB network in **9** would be inhibitory to the ChB catalytic turnover as Se···O interactions determined in our previous study was in the weak interaction range of ∼1.3 M^−1^,^[^ ^29^ ^]^ which differs by two orders of magnitude from literature known values of Se···halide interactions (∼300 M^−1^) often employed in PCH poisoning control experiments.^[^ ^116^ ^]^

On the other hand, the XB catalyzed route toward *C*‐glycosides involved a mechanistic detour away from intermediate **5**, as control experiments revealed that the XB catalysis did not catalyze the glycosylation from **5**. The insensitivity of the XB catalyzed glycosylation rate to substituent effects in our Hammett analysis further suggested a shift toward a more asynchronous S_N_2 type manifold with mild dissociative character. The *C*‐glycosylation likely involved then a nucleophilic attack directly on the anomeric carbon of the [3.1.0]‐cyclopropanated donor **1**. This XB catalyzed manifold conferred sub‐optimal stereocontrol due to the absence of anchimeric assistance, as well as the looser conformation in **11** (see Supporting Information; Figure S30) that likely accommodated nucleophilic attack on both faces. Concurrently, we do not exclude partial XB catalyzed formation of **5**,^[^ ^26^ ^]^ which likely ended up in decomposition side‐reactions and thus negatively impacting the reaction yield (Figure 3e, red reaction route). The unfavorable XB catalyzed pathway eventually resulted in diminished *C*‐glycoside yields and modest stereoselectivity. Looking forward, the presented differentiated σ‐hole activation of substrates and intermediates could potentially serve as a broader mechanistic‐tuning tool to favor or shut down competing pathways in a wider range of chemical glycosylations by judiciously modifying the σ‐hole donor.

## Conclusion

In conclusion, we developed a highly stereoselective ChB‐catalyzed *C*‐strain‐release glycosylation that was not replicable using its sister interaction, XB catalysis. Importantly, this work offers a first explanatory account of why the employment of different σ‐hole catalysts could lead to vastly different mechanistic outcomes, which in turn have profound implications in glycosylation selectivity. Our key advancement involved unraveling the differentiated σ‐hole activation of a key bicyclic intermediate **5** as the rationale underscoring the selectivity and reactivity differences in *C*‐strain‐release glycosylations. Mechanistic investigations further support that ChB catalysis shifted the mechanism toward a stereoselective pathway with S_N_1‐type character through **5**, while XB catalysis involved a rerouting toward an unselective asynchronous S_N_2 pathway away from **5**. This divergence is attributed to the unique multidentate mode **9** that is enforced by the PCH catalyst on the downstream intermediate. Besides enabling the valuable stereospecific access into *C*‐aryl glycosides, we believe that our study constitutes a significant early milestone to deepen understanding of the mechanistic rationale of selectivity and reactivity benefits when ChB catalysis is employed in chemical glycosylations.

## Conflict of Interests

The authors declare no conflict of interest.

## Supporting information



Supporting Information

## Data Availability

The data that support the findings of this study are available in the Supporting Information of this article.
