# Supplementary material for: Enhanced Selectivity of Chalcogen Bonding over Halogen Bonding Catalyzed C‐glycosylation Through Differentiated Intermediate Activation
Source: Angew Chem Int Ed Engl. 2025 Nov 13;65(2):e17553. doi: 10.1002/anie.202517553 (PMC12790375; doi:10.1002/anie.202517553)
Supplement: Supplementary file 1 — Supporting Information [file ANIE-65-e17553-s001.pdf]

Supplementary information

Enhanced Selectivity of Chalcogen Bonding over Halogen Bonding Catalyzed *C*-glycosylation through Differentiated Intermediate Activation

Hao Guo† and Charles C. J. Loh *,‡

† College of Chemistry and Materials Science, and Guangdong Provincial Key Laboratory of Functional Supramolecular Coordination Materials and Applications, Jinan University Guangzhou 510632, P. R. China.

‡ UCD School of Chemistry, University College Dublin, Belfield, Dublin 4, Ireland.

Table of contents

1. General information ········· S4

2. Synthesis of starting materials ········· S5

2.1 Synthesis of catalysts ········· S5

2.2 Synthesis of glycosyl donors **1** ········· S5

2.3 Synthesis of trialkoxybenzene **2a-2d** ········· S7

2.4 Synthesis of indoles **2e-2s** ········· S8

3. Supplementary Methods ········· S9

3.1 Optimization reaction conditions ········· S9

3.2 General procedure for Strain-Release *C*-glycosylation ········· S13

3.3 Characterization data for products ········· S13

4. Strain-release *C*-glycosylation substrate scope with XB catalysis ········· S28

5. Mechanistic study ········· S28

5.1 *In situ* NMR monitoring at -40°C for intermediate detection ········· S28

5.2 Synthesis of intermediate **5** ········· S30

5.3 NMR monitoring for the downstream reaction of intermediate **5** ········· S31

5.3.1 Reaction of intermediate **5** and **2a** in the presence of ChB catalyst **J** ········· S31

5.3.2 Reaction of intermediate **5** and **2e** in the presence of ChB catalyst **H** ········· S32

5.3.3 Reaction of intermediate **5** and **2a** in the presence of XB catalyst **C** ········· S33

5.3.4 Reaction of intermediate **5** and **2e** in the presence of XB catalyst **C** ········· S33

5.4 Poisoning experiments ········· S34

5.5 NMR titration experiments ········· S35

5.5.1 ^{77}Se NMR titration study of cyclopropyl ketone **6** and catalyst **J** ········· S35

5.5.2 ^{1}H NMR and ^{13}C NMR titration study of cyclopropyl ketone **6** and catalyst **J** ········· S36

5.5.3 ^{77}Se NMR titration study of **2a** and catalyst **J** ········· S38

5.5.4 ^{1}H NMR and ^{13}C NMR titration study of **2a** and catalyst **J** ········· S39

5.5.5 ^1H NMR and ^{13}C NMR titration study of cyclopropyl ketone **6** and catalyst **C**······S41

5.5.6 ^1H NMR and ^{13}C NMR titration study of **2a** and catalyst **C**······S43

5.5.7 ^1H NMR titration study of intermediate **5** and ChB catalyst **J**······S45

6. Hammett analysis······S45

7. Anomerization stability of product **3h** in the presence of catalyst **J**. ······S47

8. Computational Details ······S47

9. References ······S62

10. NMR spectra······S 65

1. General information

Unless otherwise stated, all reactions were set up under air. Silica Gel Flash Column Chromatography was performed on deactivated Silica gel Merck 60 (particle size 40-63 μm). Starting materials were purchased directly from commercial suppliers (Sigma Aldrich, Acros, Alfa Aesar, VWR, TCI) and used without further purifications unless otherwise stated. All solvents were dried according to standard procedures or brought from commercial suppliers. Reactions were monitored using thin-layer chromatography (TLC) on Merck silica gel aluminium plates with F254 indicator. Visualization of the developed plates was performed under UV light (254 nm) or $KMnO_4$ stain.

NMR characterization data (^1H NMR, ^{13}C NMR and 2D spectra) were collected at 300 K on a Bruker DRX400 (400 MHz), Bruker DRX600 (600 MHz), INOVA500 (500 MHz), Bruker DRX700 (700 MHz) and Bruker AVANCE NEO Ascend (400 MHz) using $CDCl_3$ and CD_2Cl_2 as solvent. Data for ^1H NMR are reported as follows: chemical shift (δ ppm), multiplicity (s = singlet, d = doublet, t = triplet, q = quartet, m = multiplet, br = broad), coupling constant (Hz), referenced with the solvent resonance as internal standard (CDCl3: δ = 7.26 ppm for ^1H, δ = 77.16 ppm for ^{13}C; CD_2Cl_2: δ = 5.32 ppm for ^1H, δ = 54.0 ppm for ^{13}C).

High resolution mass spectra were recorded on an LTQ Orbitrap mass spectrometer coupled to an Accela HPLC-System (HPLC column: Hypersyl GOLD, 50 mm x 1 mm, particle size 1.9 μm, ionization method: electron spray ionization), Bruker ultrafleXtreme MALDI-TOF-TOF (3 decimal accuracy) and Waters Xevo G2-S QTof mass spectrometer. Optical rotations were measured in a Krüss Polarimeter P3000 equipped with a sodium lamp source (589 nm), and are reported as follows: $[\alpha]_D^{T\,°C}$ (c = g/100 mL, solvent).

The ratio of anomers was determined by ^1H-NMR of the crude reaction mixture *via* integration of characteristic signals in the ^1H NMR spectra, Chemical yields refer to isolated substances after flash column chromatography. NMR yields were determined using 1,1,2,2-tetrachloroethane as internal standard.

2. Synthesis of starting materials

2.1 Synthesis of catalysts

Catalysts **A-H** were prepared according to the literature procedure.[1-4]

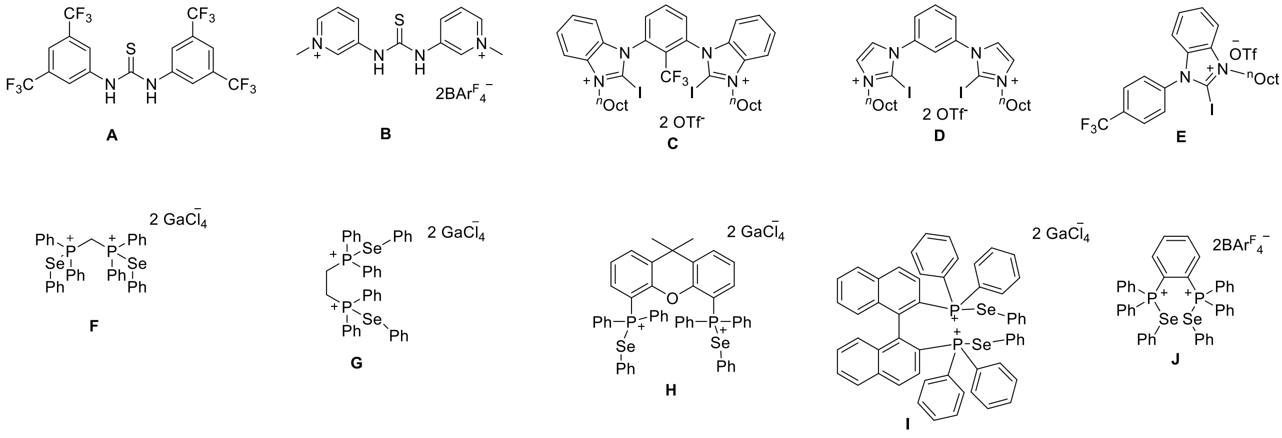

Figure S1. Structure of catalysts

2.2 Synthesis of glycosyl donors 1

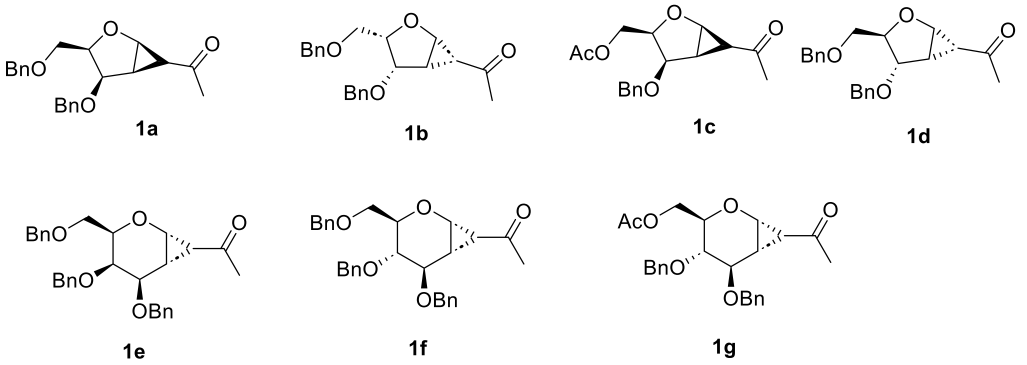

Figure S2. Structures of glycosyl donors

Glycosyl donors (**1a**-**1g** and **1j**) were prepared according to the previous published protocols.[5-6]

1-((1*R*,3*R*,4*R*,5*R*,6*R*)-4-(benzyloxy)-3-((benzyloxy)methyl)-2-oxabicyclo[3.1.0]hexan-6-yl)ethan-1-one (1a)

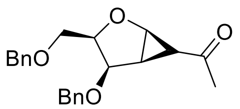

1a

^{1}H NMR (CD_2Cl_2, 700 MHz) δ (ppm) 7.37-7.27 (m, 10H), 4.56-4.50 (m, 3H), 4.50-4.45 (m, 2H), 4.37 (dd, J = 11.9, 2.1 Hz, 1H), 4.15-4.07 (m, 1H), 3.69-3.63 (m, 1H), 3.55-3.48 (m, 1H), 2.61-2.57 (m, 1H), 2.57-2.52 (m, 1H), 2.22 (s, 3H). ^{13}C NMR (CD_2Cl_2, 176 MHz) δ (ppm) 203.90, 139.06, 138.48, 128.94, 128.83, 128.20, 128.18, 88.61, 79.03, 73.79, 71.41, 69.81, 69.34, 37.08, 33.69, 31.34.

1-((1*R*,3*S*,4*S*,5*R*,6*R*)-4-(benzyloxy)-3-((benzyloxy)methyl)-2-oxabicyclo[3.1.0]hexan-6-yl)ethan-1-one (1b)

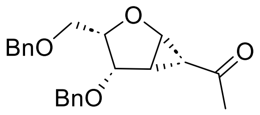

1b

^{1}H NMR (CD_2Cl_2, 500 MHz) δ (ppm) 7.46-7.16 (m, 10H), 4.58-4.45 (m, 5H), 4.37 (d, *J* = 11.8 Hz, 1H), 4.12 (dd, *J* = 5.7, 1.2 Hz, 1H), 3.66 (d, *J* = 3.5 Hz, 1H), 3.56-3.48 (m, 1H), 2.61-2.57 (m, 1H), 2.56-2.53 (m, 1H), 2.23 (s, 3H). ^{13}C NMR (CD_2Cl_2, 126 MHz) δ (ppm) 203.87, 139.06, 138.48, 128.94, 128.83, 128.26, 128.19, 128.17, 128.05, 88.60, 79.03, 73.79, 71.41, 69.81, 69.32, 37.07, 33.68, 31.33.

1-((1*R*,3*R*,4*S*,5*R*,6*R*)-4-(benzyloxy)-3-((benzyloxy)methyl)-2-oxabicyclo[3.1.0]hexan-6-yl)ethan-1-one (1d)

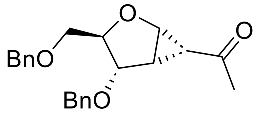

1d

^{1}H NMR (CD_2Cl_2, 500 MHz) δ (ppm) 7.42-7.24 (m, 10H), 4.60 (dd, *J* = 11.7, 4.0 Hz, 1H), 4.58-4.47 (m, 3H), 4.41-4.34 (m, 1H), 4.22-4.13 (m, 1H), 3.84-3.74 (m, 1H), 3.60-3.47 (m, 2H), 2.75-2.65 (m, 1H), 2.51-2.41 (m, 1H), 2.24 (s, 3H), 2.04-1.99 (m, 2H). ^{13}C NMR (CD_2Cl_2, 126 MHz) δ (ppm) 204.49, 138.81, 138.65, 128.92, 128.41, 128.25, 83.55, 80.89, 73.88, 72.29, 70.42, 69.77, 31.97, 31.32, 30.98.

1-((1*R*,3*R*,4*R*,5*R*,6*R*,7*R*)-4,5-bis(benzyloxy)-3-((benzyloxy)methyl)-2-oxabicyclo[4.1.0]heptan-7-yl)ethan-1-one (1e)

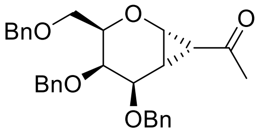

1e

^{1}H NMR (CD_2Cl_2, 500 MHz) δ (ppm) 7.48-7.19 (m, 15H), 4.84 (d, *J* = 11.4 Hz, 1H), 4.76 (d, *J* = 11.9 Hz, 1H), 4.63 (d, *J* = 11.9 Hz, 1H), 4.57 (d, *J* = 11.4 Hz, 1H), 4.53-4.41 (m, 2H), 3.81 (dd, *J* = 7.3, 2.4 Hz, 1H), 3.72 (dt, *J* = 7.5, 2.8 Hz, 2H), 3.68-3.54 (m, 3H), 2.22 (s, 3H). ^{13}C NMR (CD_2Cl_2, 126 MHz) δ (ppm) 205.14, 139.18, 138.93, 138.82, 128.91, 128.86, 128.82, 128.49, 128.35, 128.17, 128.08, 76.08, 74.82, 74.14, 73.84, 72.82, 71.86, 69.80, 61.29, 34.45, 31.21, 26.68.

1-((1*R*,3*R*,4*S*,5*R*,6*R*,7*R*)-4,5-bis(benzyloxy)-3-((benzyloxy)methyl)-2-oxabicyclo[4.1.0]heptan-7-yl)ethan-1-one (1f)

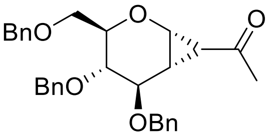

1f

^{1}H NMR (CD_2Cl_2, 500 MHz) δ (ppm) 7.37-7.26 (m, 16H), 4.74-4.65 (m, 2H), 4.61-4.54 (m, 2H), 4.51 (d, *J* = 1.4 Hz, 2H), 3.82-3.76 (m, 2H), 3.75-3.68 (m, 2H), 3.62-3.51 (m, 2H), 2.39-2.33 (m, 1H), 2.23 (s, 3H), 1.94-1.84 (m, 1H). ^{13}C NMR (CD_2Cl_2, 126 MHz) δ (ppm) 205.27, 138.93, 138.69, 128.99, 128.92,

128.87, 128.43, 128.33, 128.26, 128.23, 128.12, 127.98, 127.41, 76.56, 76.09, 75.11, 73.85, 73.72, 71.85, 69.99, 60.88, 33.38, 31.39, 27.11.

((1*R*,3*R*,4*S*,5*R*,6*R*,7*R*)-7-acetyl-4,5-bis(benzyloxy)-2-oxabicyclo[4.1.0]heptan-3-yl)methyl acetate (1g)

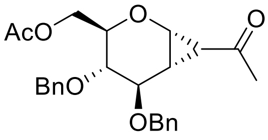

1g

^{1}H NMR (CD_2Cl_2, 500 MHz) δ (ppm) 7.43-7.20 (m, 10H), 4.75-4.69 (m, 2H), 4.62-4.54 (m, 2H), 4.39-4.31 (m, 1H), 4.16-4.08 (m, 1H), 3.86-3.73 (m, 3H), 3.54-3.44 (m, 1H), 2.38 (d, *J* = 5.9 Hz, 1H), 2.24 (s, 3H), 2.03 (s, 3H), 1.98-1.92 (m, 1H). ^{13}C NMR (CD_2Cl_2, 126 MHz) δ (ppm) 205.10, 171.08, 138.60, 138.51, 128.98, 128.94, 128.48, 128.34, 75.75, 73.61, 71.91, 63.60, 60.34, 33.15, 31.40, 26.83, 21.20.

2.3 Synthesis of trialkoxybenzene 2a-2d

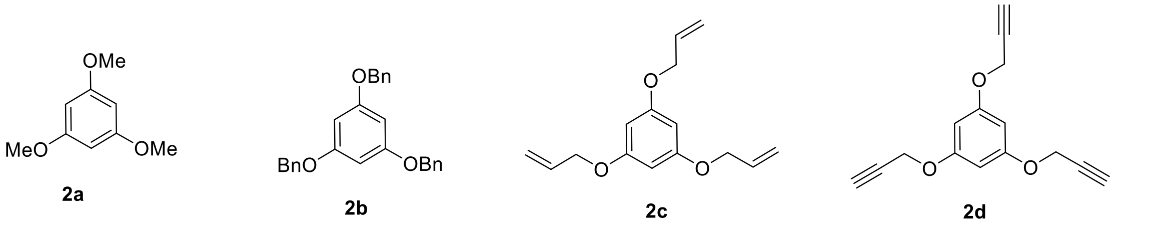

Figure S3. Structures of trialkoxybenzene

1,3,5-trimethoxybenzene **2a** was commercially available and used directly and **2b**-**2c** were prepared according to the previous published protocols.[7-9]

1,3,5-tris(benzyloxy)benzene (2b)[7]

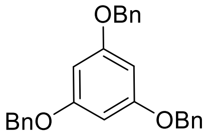

2b

^{1}H NMR ($CDCl_3$, 700 MHz) δ (ppm) 7.50-7.36 (m, 12H), 7.36-7.32 (m, 3H), 6.29 (s, 3H), 5.02 (s, 6H). ^{13}C NMR ($CDCl_3$, 176 MHz) δ (ppm) 160.79, 136.94, 128.73, 128.14, 127.71, 95.03, 70.24.

1,3,5-tris(allyloxy)benzene (2c)[8]

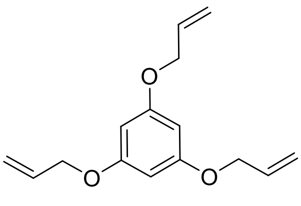

2c

^{1}H NMR ($CDCl_3$, 500 MHz) δ (ppm) 6.13 (s, 3H), 6.09-6.00 (m, 3H), 5.44-5.37 (m, 3H), 5.31-5.26 (m, 3H), 4.53-4.45 (m, 6H). ^{13}C NMR ($CDCl_3$, 126 MHz) δ (ppm) 160.51, 133.28, 117.83, 94.67, 68.98.

1,3,5-tris(prop-2-yn-1-yloxy)benzene (2d)[9]

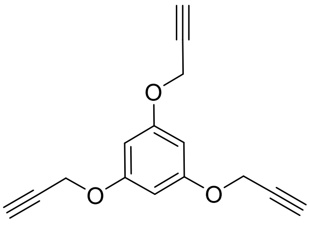

2d

^{1}H NMR ($CDCl_3$, 400 MHz) δ (ppm) 6.27 (s, 3H), 4.64 (d, J = 2.5 Hz, 6H), 2.54 (t, J = 2.4 Hz, 3H). ^{13}C NMR ($CDCl_3$, 101 MHz) δ (ppm) 159.46, 95.58, 78.38, 75.87, 56.08.

2.4 Synthesis of indoles 2e-2s

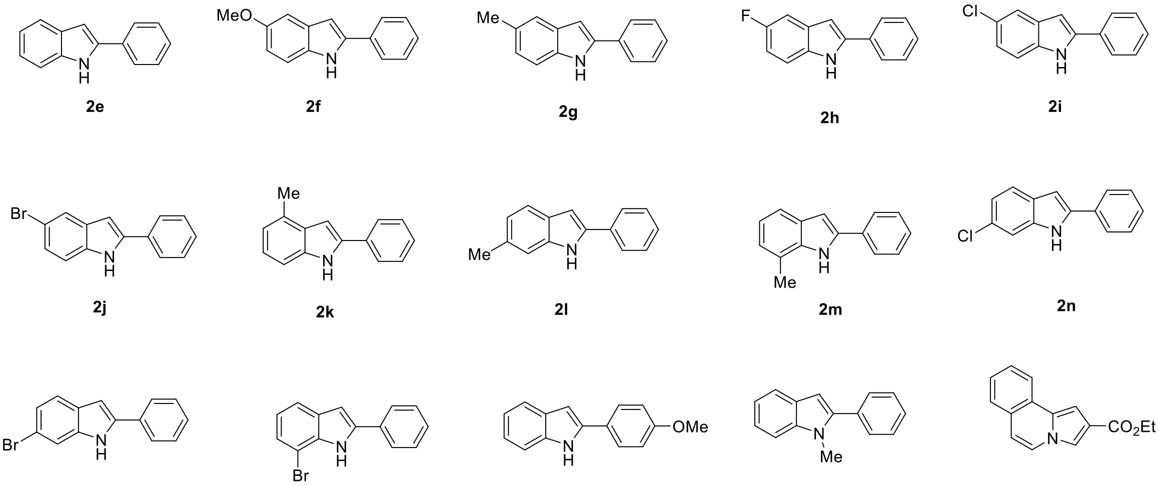

Figure S4. Structures of indoles and pyrrolo[2,1-a]isoquinoline employed in this study

Indoles **2e-2r** were prepared according to our previous published protocols,[1] pyrrolo[2,1-a]isoquinoline **2s** was synthesized according to the previous published protocols.[10]

Ethyl pyrrolo[2,1-a]isoquinoline-2-carboxylate (2s)

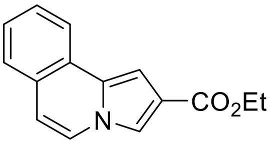

2s

^{1}H NMR ($CDCl_3$, 500 MHz) δ (ppm) 8.01 (d, *J* = 8.1 Hz, 1H), 7.79 (d, *J* = 1.6 Hz, 1H), 7.69-7.63 (m, 1H), 7.54 (d, *J* = 7.4 Hz, 1H), 7.51-7.45 (m, 1H), 7.41-7.36 (m, 1H), 7.33 (d, *J* = 0.6 Hz, 1H), 6.79 (d, *J* = 7.3 Hz, 1H), 4.37 (q, *J* = 7.1 Hz, 2H), 1.41 (t, *J* = 7.1 Hz, 3H). ^{13}C NMR ($CDCl_3$, 126 MHz) δ (ppm) 165.14, 128.18, 127.22, 126.84, 126.56, 126.50, 124.28, 122.41, 118.94, 118.80, 113.39, 101.20, 77.36, 60.32, 14.63.

3. Supplementary Methods

3.1 Optimization reaction conditions

Procedure: A mixture of catalyst (0.5 mol%-2 mol%), strained cyclopropanated furanoside **1a** (0.1 mmol, 1.0 equiv) and 1,3,5-trimethoxybenzene **2a** or indole **2e** or pyrrolo[2,1-a]isoquinoline **2s** (1.5 eq.) was dissolved in anhydrous solvent (0.1 M) in a dry tube. The mixture was stirred at room temperature. Upon completion of the reaction, the reaction mixture was filtered over a short silica plug and flushed with of dichloromethane. The filtrate was then evaporated and the reaction mixture was analyzed by crude ^{1}H NMR with 1,1,2,2-tetrachloroethane as the internal standard.

Table S1. Optimization of reaction conditions with **2a** as substrate

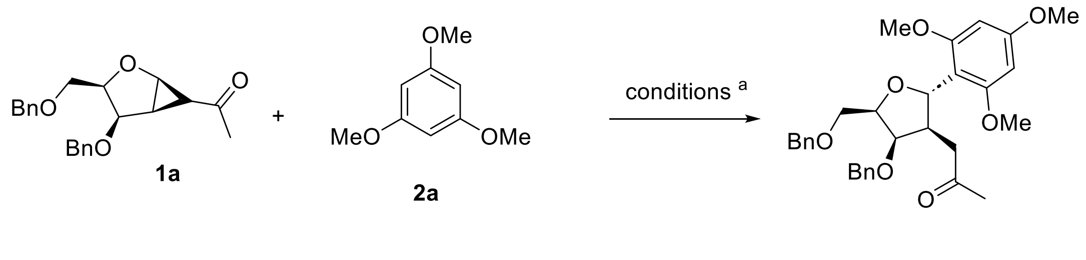

Entry	Cat.	Additive	Solvent	T (°C)	Time	NMR Yield	α:β
1	**A** (2 mol%)	-	DCM	rt	2 h	NR	NA
2	**B** (2 mol%)	-	DCM	rt	2 h	11%	NA
3	**C** (1 mol%)	-	DCM	rt	2 h	52%	3.6:1
4	**D** (1 mol%)	-	DCM	rt	2 h	NR	NA
5	**E** (2 mol%)	-	DCM	rt	2 h	<10%	NA
6	**F** (1 mol%)	-	DCM	rt	2 h	82%	> 20:1
7	**G** (1 mol%)	-	DCM	rt	2 h	66%	16.5:1
8	**H** (1 mol%)	-	DCM	rt	2 h	76%	15:1
9	**I** (1 mol%)	-	DCM	rt	2 h	61%	> 20:1

10	**J** (1 mol%)	-	DCM	rt	2 h	98%	> 20:1
11	**B** (1 mol%)	$LiClO_4$ (1.0 eq.)	Toluene	80 °C	16 h	85%	5.7:1
12	**J** (1 mol%)	PPh_3 (20 mol%)	DCM	rt	2 h	NR	NA
13	**J** (1 mol%)	TBAC (20 mol%)	DCM	rt	2 h	<10%	NA

[a] condition: 0.1 mmol **1a**, 1.5 eq **2a**, 1 mL solvent was used.; Yield and α:β ratio were determined by crude ^{1}H NMR spectra analysis using 1,1,2,2-tetrachloroethane as an internal standard. NR: no reaction; NA: no analysis; TBAC: tetrabutyl ammonium chloride.

Table S2. Optimization of reaction conditions with **2e** as substrate

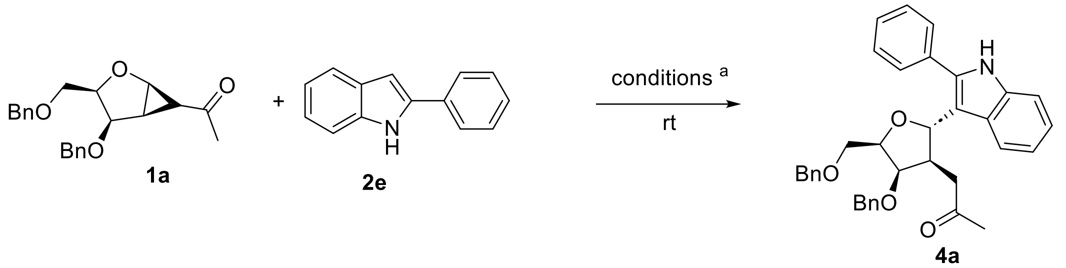

Entry	Cat.	Additive	Solvent	Time	NMR Yield	α:β
1	**A** (2 mol%)	-	DCM	12 h	NR	NA
2	**B** (2 mol%)	-	DCM	12 h	36%	8.6:1
3	**C** (2 mol%)	-	DCM	12 h	64%	5.6:1
4	**D** (2 mol%)	-	DCM	12 h	NR	NA
5	**E** (2 mol%)	-	DCM	12 h	30%	messy
6	**F** (2 mol%)	-	DCM	12 h	75%	> 20:1
7	**G** (2 mol%)	-	DCM	12 h	84%	> 20:1
8	**H** (2 mol%)	-	DCM	12 h	93%	> 20:1
9	**I** (2 mol%)	-	DCM	12 h	95%	> 20:1
10	**J** (2 mol%)	-	DCM	12 h	74%	> 20:1
11	**I** (2 mol%)	-	DCM	1 h	93%	> 20:1
12	**I** (2 mol%)	-	Toluene	1 h	76%	> 20:1

13	**I** (2 mol%)	-	THF	1 h	57%	6.5:1
14	**I** (1 mol%)	-	DCM	2 h	93%	> 20:1
15	**I** (0.5 mol%)	-	DCM	2 h	78%	> 20:1
16	**H** (1 mol%)	-	DCM	2 h	98%	> 20:1
17	**F** (1 mol%)	-	DCM	2 h	97%	> 20:1
18	**G** (1 mol%)	-	DCM	2 h	92%	> 20:1
19	**J** (1 mol%)	-	DCM	2 h	98%	> 20:1
20	**H** (1 mol%)	PPh_3 (20 mol%)	DCM	2 h	trace	NA
21	**H** (1 mol%)	TBAC (20 mol%)	DCM	2 h	trace	NA

[a] condition: 0.1 mmol **1a**, 1.5 eq **2e**, 1 mL solvent was used.; Yield and α:β ratio were determined by crude ^{1}H NMR spectra analysis using 1,1,2,2-tetrachloroethane as an internal standard. NR: no reaction; NA: no analysis; TBAC: tetrabutyl ammonium chloride.

Table S3. Optimization of reaction conditions with **2s** as substrate

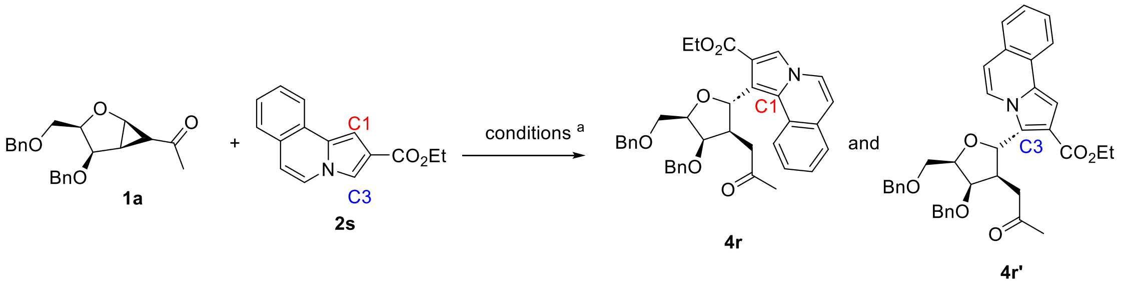

Entry	Cat.	Solvent	T (°C)	Time	NMR Yield	**4r** : **4r'** (C1:C3)	α:β for **4r**	α:β for **4r'**
1	**F** (1 mol%)	DCM	rt	40 min	50%	2.7:1	> 20:1	> 20:1
2	**G** (1 mol%)	DCM	rt	24 h	25%	2.7:1	> 20:1	> 20:1
3	**H** (1 mol%)	DCM	rt	30 min	42%	2.9:1	> 20:1	> 20:1
4	**J** (1 mol%)	DCM	rt	40 min	43%	2.3:1	> 20:1	> 20:1
5	**J** (1 mol%)	DCM	0t	2 h	24%	2.3:1	> 20:1	> 20:1

6	**J** (1 mol%)	DCM	-20	12 h	15%	4.4:1	> 20:1	> 20:1
7	**J** (1 mol%)	DCM	rt	10 min	48%	2.4:1	> 20:1	> 20:1
8	**J** (1 mol%)	DCM	0	3 h	38	2.7:1	> 20:1	> 20:1
9	**J** (1 mol%)	Toluene	rt	1 h	31%	2.1:1	> 20:1	> 20:1
10	**J** (1 mol%)	Chlorobe nzene	rt	1 h	34%	2:1	> 20:1	> 20:1
11	**J** (1 mol%)	benzene	rt	1 h	33%	2:1	> 20:1	> 20:1
12	**J** (1 mol%)	CCl_4	rt	1 h	41%	1.3:1	-	-
13	**J** (1 mol%)	EA	rt	1 h	64%	2:1	> 20:1	> 20:1
14	**J** (1 mol%)	THF	rt	1 h	41%	2.5:1	> 20:1	> 20:1
15	**F** (1 mol%)	EA	rt	1 h	71%	2.4:1	> 20:1	> 20:1

[a] condition: 0.1 mmol **1a**, 1.5 eq **2s**, 1 mL solvent was used.; Yield and α:β ratio were determined by crude ^{1}H NMR spectra analysis using 1,1,2,2-tetrachloroethane as an internal standard. EA: ethyl acetate.

Table S4. Optimization of reaction conditions with **2t** as substrate

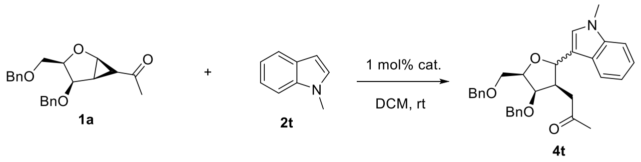

Entry	Cat.	T (°C)	Time	Conversion of **1a**	NMR yield	α:β
1	**F** (1 mol%)	rt	10 min	100%	<10%	NA
2	**G** (1 mol%)	rt	10 min	100%	trace	NA
3	**H** (1 mol%)	rt	1 h	100%	<15%	NA
4	**H** (1 mol%)	0	1 h	100%	trace	NA
5	**J** (1 mol%)	rt	10 min	100%	<10%	NA

[a] condition: 0.1 mmol **1a**, 1.5 eq **2t**, 1 mL solvent was used.; Yield and α:β ratio were determined by crude ^{1}H NMR spectra analysis using 1,1,2,2-tetrachloroethane as an internal standard. NA: no analysis.

3.2 General procedure for Strain-Release *C*-glycosylation

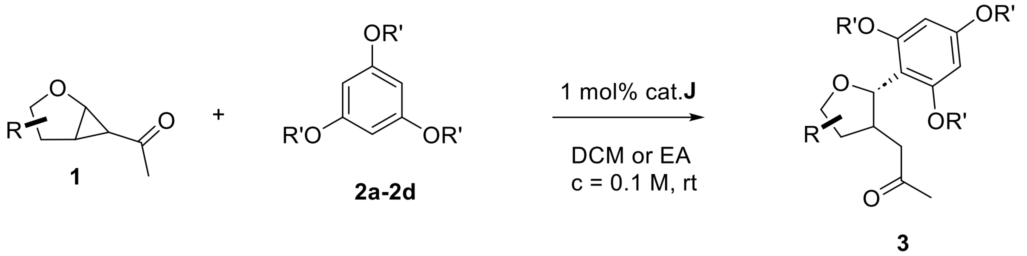

General procedure A: A mixture of glycosyl donor **1** (1.0 eq. 0.1 mmol), trialkoxybenzene **2a-2d** (1.5 eq.) and catalyst **J** (1 mol%) were dissolved in the 1 mL corresponding solvent (CH_2Cl_2 or ethyl acetate), the resulting reaction mixture was stirred at room temperature for 10 min to 2 h (depending on the substrate). Subsequently, the reaction mixture was filtered over a short silica plug and flushed with dichloromethane. The filtrate was removed under reduced pressure and the residue was analyzed by crude ^{1}H NMR and then subjected to flash column chromatography (dry loading) to give the desired product **3**.

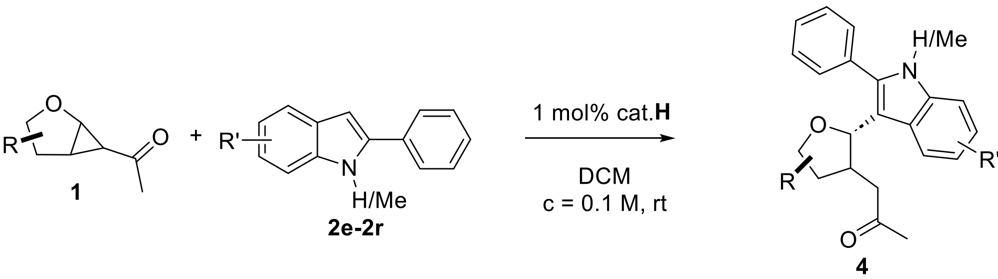

General procedure B: A mixture of glycosyl donor **1** (1.0 eq. 0.1 mmol), indole **2e**-**2r** or pyrrolo[2,1-a]isoquinoline **2s** (1.5 eq.) and catalyst **H** (1 mol%) were dissolved in the 1 mL corresponding solvent (CH_2Cl_2), the resulting reaction mixture was stirred at room temperature for 10 min to 2 h (depending on the substrate). Subsequently, the reaction mixture was filtered over a short silica plug and flushed with dichloromethane. The filtrate was removed under reduced pressure and the residue was analyzed by crude ^{1}H NMR and then subjected to flash column chromatography (dry loading) to give the desired product **4**.

3.3 Characterization data for products

1-((2*S*,3*R*,4*R*,5*R*)-4-(benzyloxy)-5-((benzyloxy)methyl)-2-(2,4,6-trimethoxyphenyl)tetrahydrofuran-3-yl)propan-2-one (3a)

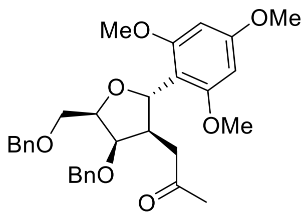

3a

The title product compound is prepared according to the general procedure A with 0.1 mmol scale in CH_2Cl_2 at room temperature for 2 h and isolated by flash column chromatography (2:1 Pentane: Ethyl Acetate) giving a colorless oil (48 mg, 92% yield, α/β ratio > 20:1). ^{1}H NMR (CD_2Cl_2, 500 MHz) δ (ppm) 7.39-7.27 (m, 10H), 6.14 (s, 2H), 5.22 (d, J = 10.9 Hz, 1H), 4.63 (d, J = 11.2 Hz, 1H), 4.59-4.52 (m, 2H), 4.52-4.48 (m, 1H), 4.39 (d, *J* = 11.3 Hz, 1H), 4.33 (dd, *J* = 4.3, 2.9 Hz, 1H), 3.82-3.76 (m, 10H), 3.67 (dd, *J* = 9.3, 5.8 Hz, 1H), 3.24-3.15 (m, 1H), 2.77 (dd, *J* = 18.2, 10.4 Hz, 1H), 2.23 (dd, *J* = 18.3, 3.6 Hz,

1H), 1.95 (s, 3H). ^{13}C NMR (CD_2Cl_2, 126 MHz) δ (ppm) 208.35, 161.53, 160.59, 139.36, 139.12, 128.87, 128.83, 128.58, 128.36, 128.15, 128.05, 109.03, 91.47, 81.90, 81.58, 75.51, 74.79, 73.94, 69.65, 56.35, 55.77, 43.28, 40.23, 30.25. HRMS (ESI$^+$) calc. for [M+H]$^+$ ($C_{31}H_{36}O_7Na^+$), 543.2353, found:543.2353; $[\alpha]_D^{20}$ = +31.5 (*c* = 0.85, CH_2Cl_2).

1-((2*S*,3*R*,4*R*,5*R*)-4-(benzyloxy)-5-((benzyloxy)methyl)-2-(2,4,6-tris(benzyloxy)phenyl)tetrahydrofuran-3-yl)propan-2-one (3b)

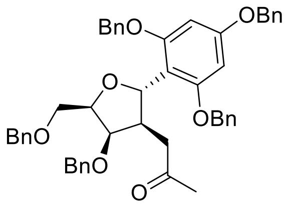

3b

The title product compound is prepared according to the general procedure A with 0.1 mmol scale in CH_2Cl_2 at room temperature for 0.5 h and isolated by flash column chromatography (5:1 Pentane: Ethyl Acetate) giving a colorless oil (52 mg, 70% yield, α/β ratio > 20:1). ^{1}H NMR (CD_2Cl_2, 500 MHz) δ (ppm) 7.48-7.43 (m, 4H), 7.42-7.37 (m, 8H), 7.37-7.32 (m, 7H), 7.32-7.26 (m, 6H), 6.28 (s, 2H), 5.37 (d, *J* = 10.8 Hz, 1H), 5.11-5.02 (m, 4H), 5.00 (s, 2H), 4.58 (d, *J* = 11.6 Hz, 1H), 4.48-4.38 (m, 2H), 4.34 (d, *J* = 11.6 Hz, 1H), 4.26-4.19 (m, 1H), 4.18-4.13 (m, 1H), 3.70 (dd, *J* = 9.5, 7.2 Hz, 1H), 3.57 (dd, *J* = 9.5, 5.9 Hz, 1H), 3.33-3.16 (m, 1H), 2.75 (dd, *J* = 18.2, 10.5 Hz, 1H), 2.26 (dd, *J* = 18.2, 3.4 Hz, 1H), 1.92 (s, 3H). ^{13}C NMR (CD_2Cl_2, 126 MHz) δ (ppm) 208.14, 160.35, 159.59, 139.44, 139.26, 137.62, 137.47, 129.11, 128.82, 128.76, 128.59, 128.51, 128.29, 128.23, 128.17, 128.16, 128.01, 127.92, 110.33, 94.02, 81.70, 81.31, 75.73, 74.54, 73.63, 71.25, 70.63, 69.51, 43.59, 40.23, 30.24. HRMS (ESI$^+$) calc. for [M+H]$^+$ ($C_{49}H_{48}O_7Na^+$), 771.3292, found:543.3315; $[\alpha]_D^{20}$ = +23.4 (*c* = 0.32, CH_2Cl_2).

1-((2*S*,3*R*,4*R*,5*R*)-4-(benzyloxy)-5-((benzyloxy)methyl)-2-(2,4,6-tris(allyloxy)phenyl)tetrahydrofuran-3-yl)propan-2-one (3c)

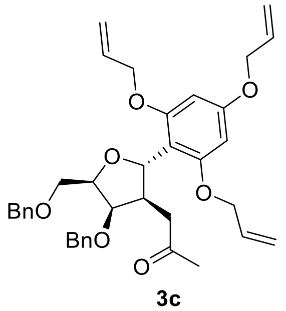

3c

The title product compound is prepared according to the general procedure A with 0.1 mmol scale in CH_2Cl_2 at room temperature for 1 h and isolated by flash column chromatography (3:1 Pentane: Ethyl Acetate) giving a colorless oil (40 mg, 66% yield, α/β ratio > 20:1). ^{1}H NMR (CD_2Cl_2, 500 MHz) δ (ppm) 7.37-7.33 (m, 6H), 7.33-7.27 (m, 4H), 6.14 (s, 2H), 6.13-5.99 (m, 3H), 5.40 (d, *J* = 17.2 Hz, 3H), 5.29 (d, *J* = 10.8 Hz, 3H), 4.67-4.47 (m, 11H), 4.40 (d, *J* = 11.4 Hz, 1H), 4.34-4.30 (m, 1H), 3.78 (dd, *J* = 9.4, 7.1 Hz, 1H), 3.65 (dd, *J* = 9.4, 5.8 Hz, 1H), 3.27-3.17 (m, 1H), 2.80-2.73 (m, 1H), 2.27 (dd, *J* = 18.3, 3.6 Hz, 1H), 1.93 (s, 3H). ^{13}C NMR (CD_2Cl_2, 126 MHz) δ (ppm) 208.22, 160.20, 159.50, 139.39, 139.19, 134.07, 133.89, 128.86, 128.81, 128.48, 128.28, 128.11, 128.01, 118.02, 117.93, 110.10, 93.72, 81.84, 81.62, 75.69, 74.69, 73.84, 70.18, 69.64, 69.43, 43.53, 40.23, 30.24. HRMS (ESI$^+$) calc. for [M+H]$^+$ ($C_{37}H_{42}O_7Na^+$), 628.2823, found:621.2821; $[\alpha]_D^{20}$ = +25.9 (*c* = 0.32, CH_2Cl_2).

1-((2*S*,3*R*,4*R*,5*R*)-4-(benzyloxy)-5-((benzyloxy)methyl)-2-(2,4,6-tris(prop-2-yn-1-yloxy)phenyl)tetrahydrofuran-3-yl)propan-2-one (3d)

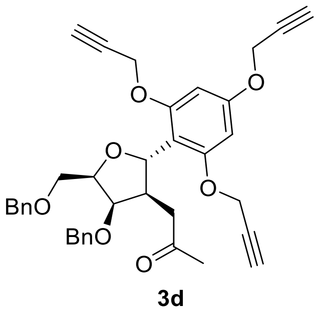

3d

The title product compound is prepared according to the general procedure A with 0.1 mmol scale in ethyl acetate at room temperature for 1 h and isolated by flash column chromatography (3:1 Pentane: Ethyl Acetate) giving a colorless oil (33.8 mg, 57% yield, α/β ratio > 20:1). ^{1}H NMR (CD_2Cl_2, 500 MHz) δ (ppm) 7.36-7.32 (m, 5H), 7.32-7.27 (m, 5H), 6.35 (s, 2H), 5.20 (d, J = 10.8 Hz, 1H), 4.72-4.49 (m, 10H), 4.38 (d, J = 11.3 Hz, 1H), 4.35-4.31 (m, 1H), 3.78 (dd, J = 9.4, 7.3 Hz, 1H), 3.66 (dd, J = 9.4, 5.8 Hz, 1H), 3.21-3.07 (m, 1H), 2.77 (dd, J = 18.4, 10.4 Hz, 1H), 2.59 (dt, J = 4.9, 2.3 Hz, 3H), 2.27 (dd, J = 18.3, 3.6 Hz, 1H), 1.94 (s, 3H). ^{13}C NMR (CD_2Cl_2, 126 MHz) δ (ppm) 208.16, 158.96, 158.50, 128.88, 128.82, 128.60, 128.31, 128.17, 128.03, 111.91, 94.88, 81.78, 81.71, 79.01, 78.81, 76.21, 76.09, 75.50, 74.81, 73.86, 69.56, 57.23, 56.57, 54.43, 43.86, 40.11, 30.26. HRMS (ESI$^+$) calc. for $[M+H]^+$ ($C_{37}H_{36}O_7Na^+$), 615.2353, found:615.2351; $[\alpha]_D^{20}$ = +19.5 (c = 0.20, CH_2Cl_2).

1-((2*R*,3*S*,4*S*,5*S*)-4-(benzyloxy)-5-((benzyloxy)methyl)-2-(2,4,6-trimethoxyphenyl)tetrahydrofuran-3-yl)propan-2-one (3e)

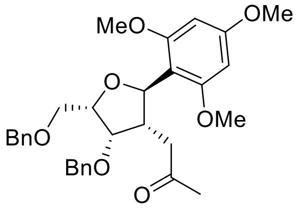

3e

The title product compound is prepared according to the general procedure A with 0.1 mmol scale in CH_2Cl_2 at room temperature for 0.5 h and isolated by flash column chromatography (2:1 Pentane: Ethyl Acetate) giving a colorless oil (42 mg, 81% yield, α/β ratio > 20:1). ^{1}H NMR (CD_2Cl_2, 500 MHz) δ (ppm) 7.39-7.26 (m, 10H), 6.13 (s, 2H), 5.21 (d, J = 10.8 Hz, 1H), 4.62 (d, J = 11.3 Hz, 1H), 4.59-4.51 (m, 2H), 4.51-4.46 (m, 1H), 4.38 (d, J = 11.3 Hz, 1H), 4.32 (dd, J = 4.3, 2.9 Hz, 1H), 3.81-3.78 (m, 9H), 3.78-3.75 (m, 1H), 3.66 (dd, J = 9.3, 5.7 Hz, 1H), 3.23-3.14 (m, 1H), 2.76 (dd, J = 18.3, 10.4 Hz, 1H), 2.22 (dd, J = 18.3, 3.6 Hz, 1H), 1.94 (s, 3H). ^{13}C NMR (CD_2Cl_2, 126 MHz) δ (ppm) 208.36, 161.53, 160.59, 139.36, 139.13, 128.87, 128.83, 128.58, 128.36, 128.15, 128.05, 109.03, 91.47, 81.90, 81.58, 75.51, 74.80, 73.94, 69.65, 56.36, 55.78, 43.28, 40.23, 30.25. HRMS (ESI$^+$) calc. for $[M+H]^+$ ($C_{31}H_{36}O_7Na^+$), 543.2353, found:543.2355; $[\alpha]_D^{20}$ = -29.1 (c = 0.34, CH_2Cl_2).

((2*R*,3*R*,4*R*,5*S*)-3-(benzyloxy)-4-(2-oxopropyl)-5-(2,4,6-trimethoxyphenyl)tetrahydrofuran-2-yl)methyl acetate (3f)

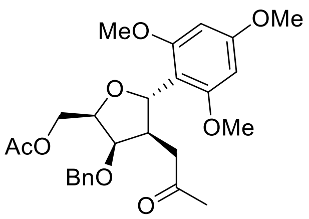

3f

The title product compound is prepared according to the general procedure A with 0.1 mmol scale in CH_2Cl_2 at room temperature for 1 h and isolated by flash column chromatography (1:1 Pentane: Ethyl Acetate) giving a colorless oil (35 mg, 74% yield, α/β ratio > 20:1). ^{1}H NMR (CD_2Cl_2, 500 MHz) δ (ppm) 7.40-7.29 (m, 5H), 6.13 (s, 2H), 5.24 (d, J = 10.9 Hz, 1H), 4.57 (d, J = 11.1 Hz, 1H), 4.49-4.44 (m, 1H), 4.38 (d, J = 11.2 Hz, 1H), 4.34 (dd, J = 4.4, 3.1 Hz, 1H), 4.29-4.21 (m, 2H), 3.79 (s, 3H), 3.78 (s, 6H), 3.22-3.15 (m, 1H), 2.76 (dd, J = 18.4, 10.6 Hz, 1H), 2.25 (dd, J = 18.5, 3.5 Hz, 1H), 2.04 (s, 3H), 1.96 (s, 3H). ^{13}C NMR (CD_2Cl_2, 126 MHz) δ (ppm) 208.41, 171.15, 161.64, 160.61, 138.93, 128.95, 128.63, 128.32, 108.67, 91.53, 81.95, 80.35, 75.75, 75.01, 63.89, 56.38, 55.80, 43.26, 40.09, 30.26, 21.29. HRMS (ESI$^+$) calc. for $[M+H]^+$ ($C_{26}H_{32}O_8Na^+$), 495.1589, found:495.1587; $[\alpha]_D^{20}$ = +43.0 (c = 0.30, CH_2Cl_2).

1-((2*R*,3*S*,4*S*,5*R*)-4-(benzyloxy)-5-((benzyloxy)methyl)-2-(2,4,6-trimethoxyphenyl)tetrahydrofuran-3-yl)propan-2-one (3g)

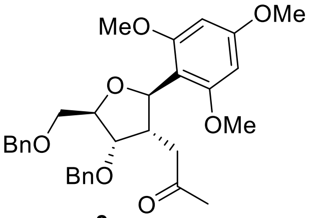

3g

The title product compound is prepared according to the general procedure A with 0.1 mmol scale in CH_2Cl_2 at room temperature for 1 h and isolated by flash column chromatography (2:1 Pentane: Ethyl Acetate) giving a colorless oil (48.8 mg, 94% yield, β/α ratio > 20:1). ^{1}H NMR (CD_2Cl_2, 500 MHz) δ (ppm) δ 7.42-7.26 (m, 10H), 6.11 (s, 2H), 5.21 (d, J = 10.8 Hz, 1H), 4.60 (d, J = 11.1 Hz, 3H), 4.34 (d, J = 11.4 Hz, 1H), 4.22-4.17 (m, 1H), 4.13 (dd, J = 6.1, 1.8 Hz, 1H), 3.79 (s, 3H), 3.73 (s, 6H), 3.64-3.58 (m, 1H), 3.56 (dd, J = 9.6, 7.6 Hz, 1H), 3.29-3.21 (m, 1H), 2.81 (dd, J = 17.7, 10.2 Hz, 1H), 2.14 (dd, J = 17.7, 4.3 Hz, 1H), 1.99 (s, 3H). ^{13}C NMR (CD_2Cl_2, 126 MHz) δ (ppm) 208.23, 161.78, 161.01, 139.30, 139.17, 128.84, 128.77, 128.61, 128.16, 128.08, 128.00, 107.16, 91.53, 82.67, 82.60, 76.31, 73.80, 72.05, 71.97, 56.09, 55.77, 40.50, 39.90, 30.47. HRMS (ESI$^+$) calc. for $[M+H]^+$ ($C_{31}H_{36}O_7Na^+$), 543.2353, found:543.2353; $[\alpha]_D^{20}$ = -51.2 (c = 0.41, CH_2Cl_2).

1-((2*R*,3*S*,4*R*,5*R*,6*R*)-4,5-bis(benzyloxy)-6-((benzyloxy)methyl)-2-(2,4,6-trimethoxyphenyl)tetrahydro-2H-pyran-3-yl)propan-2-one (3h)

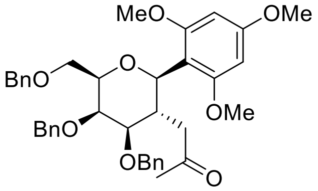

3h

The title product compound is prepared according to the general procedure A with 0.1 mmol scale in CH_2Cl_2 at room temperature for 1 h and isolated by flash column chromatography (2:1 Pentane: Ethyl

Acetate) giving a colorless oil (51 mg, 80% yield, β/α ratio > 20:1). ^{1}H NMR (CD_2Cl_2, 500 MHz) δ (ppm) 7.45-7.39 (m, 2H), 7.37-7.31 (m, 10H), 7.31-7.25 (m, 3H), 6.11 (dt, *J* = 12.0, 2.1 Hz, 2H), 4.95 (d, *J* = 11.5 Hz, 1H), 4.77-4.70 (m, 2H), 4.64-4.57 (m, 1H), 4.53-4.48 (m, 1H), 4.45 (ddd, *J* = 11.7, 5.4, 1.9 Hz, 2H), 4.03 (s, 1H), 3.82-3.73 (m, 9H), 3.72-3.63 (m, 3H), 3.61-3.55 (m, 1H), 3.47-3.41 (m, 1H), 2.22-2.12 (m, 1H), 2.09-2.00 (m, 1H), 1.81 (s, 3H). ^{13}C NMR (CD_2Cl_2, 126 MHz) δ (ppm) 208.16, 161.96, 161.67, 159.83, 140.34, 138.97, 138.85, 128.84, 128.82, 128.59, 128.50, 128.44, 128.14, 128.06, 127.78, 127.62, 108.43, 92.63, 91.02, 84.70, 77.92, 74.78, 74.49, 73.86, 72.51, 71.64, 70.13, 56.59, 56.36, 55.78, 44.39, 37.08, 29.60. HRMS (ESI$^+$) calc. for [M+H]$^+$ ($C_{39}H_{44}O_8Na^+$), 663.2928, found:663.2931; $[\alpha]_D^{20}$ = +36.3 (*c* = 0.40, CH_2Cl_2).

1-((2*R*,3*S*,4*R*,5*S*,6*R*)-4,5-bis(benzyloxy)-6-((benzyloxy)methyl)-2-(2,4,6-trimethoxyphenyl)tetrahydro-2H-pyran-3-yl)propan-2-one (3i)

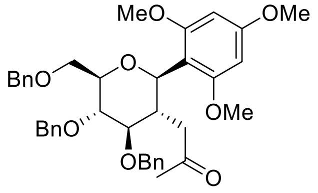

3i

The title product compound is prepared according to the general procedure A with 0.1 mmol scale in CH_2Cl_2 at room temperature for 10 min and isolated by flash column chromatography (2:1 Pentane: Ethyl Acetate) giving a colorless oil (32 mg, 50% yield, β/α ratio > 20:1). ^{1}H NMR (CD_2Cl_2, 500 MHz) δ (ppm) 7.37-7.35 (m, 2H), 7.32 (d, *J* = 3.1 Hz, 5H), 7.29-7.26 (m, 6H), 7.25-7.22 (m, 2H), 6.16-6.06 (m, 2H), 4.92 (d, *J* = 11.0 Hz, 1H), 4.82 (d, *J* = 10.9 Hz, 1H), 4.74 (d, *J* = 10.5 Hz, 1H), 4.70 (dd, *J* = 11.5, 8.3 Hz, 2H), 4.57 (d, *J* = 11.0 Hz, 1H), 4.52 (d, *J* = 11.9 Hz, 1H), 3.82 (dd, *J* = 11.3, 4.4 Hz, 1H), 3.81-3.77 (m, 9H), 3.77-3.74 (m, 1H), 3.73-3.69 (m, 1H), 3.54-3.49 (m, 1H), 3.45 (dd, *J* = 10.6, 8.8 Hz, 1H), 3.26-3.16 (m, 1H), 2.19 (dd, *J* = 15.6, 6.5 Hz, 1H), 2.08 (dd, *J* = 15.6, 5.4 Hz, 1H), 1.75 (s, 3H). ^{13}C NMR (126 MHz, CD_2Cl_2) δ (ppm) 207.49, 162.03, 161.46, 159.69, 139.44, 139.27, 128.87, 128.83, 128.78, 128.33, 128.24, 128.22, 128.06, 127.91, 127.88, 107.92, 92.29, 90.91, 86.24, 80.99, 80.49, 75.11, 75.09, 74.24, 73.79, 70.11, 56.38, 56.34, 55.82, 44.10, 41.95, 29.58. HRMS (ESI$^+$) calc. for [M+H]$^+$ ($C_{39}H_{44}O_8Na^+$), 663.2928, found:663.2930; $[\alpha]_D^{20}$ = +31.3 (*c* = 0.16, CH_2Cl_2).

((2*R*,3*S*,4*R*,5*S*,6*R*)-3,4-bis(benzyloxy)-5-(2-oxopropyl)-6-(2,4,6-trimethoxyphenyl)tetrahydro-2H-pyran-2-yl)methyl acetate (3j)

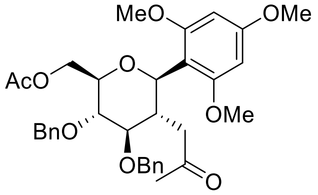

3j

The title product compound is prepared according to the general procedure A with 0.1 mmol scale in CH_2Cl_2 at room temperature for 10 min and isolated by flash column chromatography (2:1 Pentane: Ethyl Acetate) giving a colorless oil (39 mg, 66% yield, β/α ratio > 20:1). ^{1}H NMR (CD_2Cl_2, 500 MHz) δ (ppm) 7.36-7.26 (m, 10H), 6.15-6.08 (m, 2H), 4.93 (d, *J* = 11.1 Hz, 1H), 4.87 (d, *J* = 10.9 Hz, 1H), 4.77 (d, *J* = 10.6 Hz, 1H), 4.61 (dd, *J* = 14.3, 11.0 Hz, 2H), 4.41 (dd, *J* = 11.8, 2.0 Hz, 1H), 4.15 (dd, *J* = 11.8, 5.0 Hz, 1H), 3.82 (s, 3H), 3.80 (s, 3H), 3.78 (s, 3H), 3.66 – 3.60 (m, 1H), 3.60 – 3.54 (m, 1H), 3.49 (dd, *J* = 10.6, 8.5 Hz, 1H), 3.26-3.17 (m, 1H), 2.20 (dd, *J* = 15.8, 6.5 Hz, 1H), 2.09 (dd, *J* = 15.8, 5.3 Hz, 1H), 2.04 (s, 3H), 1.75 (s, 3H). ^{13}C NMR (126 MHz, CD_2Cl_2) δ (ppm) 207.34, 171.21, 162.11, 161.50, 159.69, 139.26, 138.86, 128.92, 128.82, 128.37, 128.27, 128.19, 128.00, 107.83, 92.57, 91.07, 86.15, 80.91,

78.12, 75.25, 75.20, 74.13, 64.38, 56.51, 56.41, 55.83, 43.76, 41.84, 29.67, 21.28. HRMS (ESI$^+$) calc. for $[M+H]^+$ ($C_{34}H_{40}O_9Na^+$), 615.2565, found:615.2572; $[\alpha]_D^{20}$ = +31.9 (*c* = 0.32, CH_2Cl_2).

1-((2*S*,3*R*,4*R*,5*R*)-4-(benzyloxy)-5-((benzyloxy)methyl)-2-(2-phenyl-1H-indol-3-yl)tetrahydrofuran-3-yl)propan-2-one (4a)

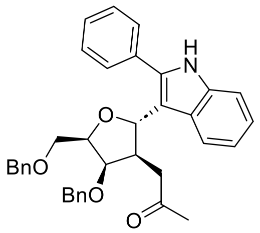

4a

The title product compound is prepared according to the general procedure B with 0.1 mmol scale in CH_2Cl_2 at room temperature for 2 h and isolated by flash column chromatography (2:1 Pentane: Ethyl Acetate) giving a colorless oil (52 mg, 95% yield, α/β ratio > 20:1). ^{1}H NMR (CD_2Cl_2, 700 MHz) δ (ppm) 8.43 (s, 1H), 7.75 (d, J = 8.0 Hz, 1H), 7.54-7.48 (m, 2H), 7.45-7.39 (m, 3H), 7.39-7.32 (m, 7H), 7.31-7.28 (m, 2H), 7.28-7.25 (m, 2H), 7.22-7.18 (m, 1H), 7.15-7.12 (m, 1H), 5.05 (d, *J* = 11.2 Hz, 1H), 4.68 (td, *J* = 6.4, 3.5 Hz, 1H), 4.65-4.53 (m, 3H), 4.48 (t, *J* = 3.9 Hz, 1H), 4.38 (d, *J* = 11.4 Hz, 1H), 3.83 (dd, *J* = 9.6, 6.9 Hz, 1H), 3.74 (dd, *J* = 9.6, 6.0 Hz, 1H), 3.17 (tt, *J* = 10.9, 3.3 Hz, 1H), 2.64 (dd, J = 18.2, 10.7 Hz, 1H), 2.12 (dd, *J* = 18.2, 3.1 Hz, 1H), 1.82 (s, 3H). ^{13}C NMR (CD_2Cl_2, 176 MHz) δ (ppm) 207.72, 139.08, 139.01, 138.38, 136.86, 132.97, 129.45, 129.35, 128.89, 128.84, 128.81, 128.54, 128.34, 128.21, 128.06, 127.41, 122.88, 120.71, 120.42, 111.68, 111.15, 81.76, 81.22, 77.81, 74.90, 73.95, 69.69, 54.31, 54.15, 54.00, 53.84, 53.69, 45.30, 39.69, 30.14. HRMS (ESI$^+$) calc. for $[M+H]^+$ ($C_{36}H_{35}NO_4Na^+$), 568.2458, found:548.2460; $[\alpha]_D^{20}$ = +27.3 (*c* = 0.92, CH_2Cl_2).

1-((2*S*,3*R*,4*R*,5*R*)-4-(benzyloxy)-5-((benzyloxy)methyl)-2-(2-phenyl-1H-indol-3-yl)tetrahydrofuran-3-yl)propan-2-one (4a)

1-((2*R*,3*R*,4*R*,5*R*)-4-(benzyloxy)-5-((benzyloxy)methyl)-2-(2-phenyl-1H-indol-3-yl)tetrahydrofuran-3-yl)propan-2-one (4a')

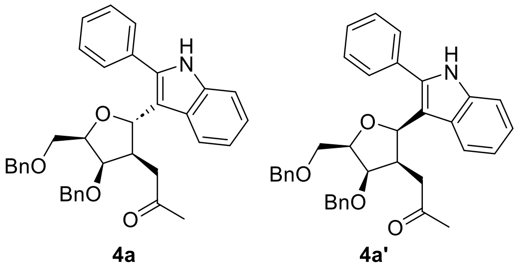

4a **4a'**

The title product compound is prepared according to the general procedure B with 1 mol% cat.**C** and 0.1 mmol scale in CH_2Cl_2 at room temperature for 2 h and isolated by flash column chromatography (2:1 Pentane: Ethyl Acetate) giving a colorless syrup (27.3 mg, 50% yield, α/β ratio = 3.6:1). ^{1}H NMR (CD_2Cl_2, 700 MHz) δ (ppm) 8.56 (s, 1H), 7.77 (d, *J* = 7.9 Hz, 1H), 7.53-7.47 (m, 2H), 7.45-7.39 (m, 3.4H), 7.38-7.24 (m, 13.2H), 7.22-7.18 (m, 1.2H), 7.17-7.12 (m, 1H), 5.09-5.07 (m, 1H), 4.74-4.67 (m, 1H), 4.67-4.61 (m, 1.3H), 4.60-4.53 (m, 2.2H), 4.53-4.47 (m, 1.2H), 4.39 (d, *J* = 11.4 Hz, 1H), 4.36-4.33 (m, 0.3H), 4.30-4.28 (m, 0.2H), 3.87-3.83 (m, 1H), 3.78-3.74 (m, 1.2H), 3.69-3.65 (m, 0.2H), 3.22-3.16 (m, 1H), 2.80 (dd, *J* = 17.6, 9.7 Hz,, 0.2H), 2.65 (dd, *J* = 18.2, 10.6 Hz, 1H), 2.55 (dd, *J* = 17.8, 4.6 Hz, 1H), 2.13 (dd, *J* = 18.2, 3.1 Hz, 1H), 2.0(s, 0.4H), 1.83 (s, 3H). ^{13}C NMR (CD_2Cl_2, 126 MHz) δ (ppm) 208.90, 207.77, 139.27, 139.07, 138.99, 138.86, 138.80, 138.42, 136.89, 136.61, 132.97, 132.90, 129.46, 129.31, 129.26, 129.03, 128.89, 128.87, 128.84, 128.77, 128.54, 128.36, 128.34, 128.21,

128.14, 128.07, 128.01, 127.40, 122.85, 120.69, 120.40, 111.74, 111.06, 81.78, 81.21, 80.70, 80.37, 77.85, 76.91, 74.89, 74.76, 73.95, 73.92, 69.69, 69.25, 54.43, 54.21, 54.00, 53.79, 53.57, 46.07, 45.29, 39.93, 39.68, 30.31, 30.12. HRMS (ESI$^+$) calc. for $[M+H]^+$ ($C_{36}H_{35}NO_4Na^+$), 568.2458, found:548.2460;

1-((2*S*,3*R*,4*R*,5*R*)-4-(benzyloxy)-5-((benzyloxy)methyl)-2-(5-methoxy-2-phenyl-1H-indol-3-yl)tetrahydrofuran-3-yl)propan-2-one (4b)

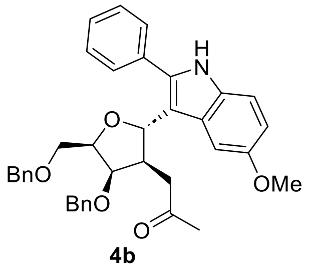

4b

The title product compound is prepared according to the general procedure B with 0.1 mmol scale in CH_2Cl_2 at room temperature for 40 min and isolated by flash column chromatography (2:1 Pentane: Ethyl Acetate) giving a colorless oil (50 mg, 89% yield, α/β ratio > 20:1). ^1H NMR (CD_2Cl_2, 600 MHz) δ (ppm) 8.32 (s, 1H), 7.52-7.49 (m, 2H), 7.45-7.39 (m, 3H), 7.39-7.32 (m, 7H), 7.32-7.28 (m, 2H), 7.26 (d, *J* = 8.4 Hz, 3H), 7.17 (d, *J* = 2.4 Hz, 1H), 6.84 (dd, *J* = 8.7, 2.4 Hz, 1H), 5.03 (d, *J* = 11.2 Hz, 1H), 4.68-4.65 (m, 1H), 4.63 (d, *J* = 11.4 Hz, 1H), 4.57 (q, *J* = 11.8 Hz, 2H), 4.47 (t, *J* = 4.0 Hz, 1H), 4.38 (d, *J* = 11.4 Hz, 1H), 3.85 (s, 3H), 3.83 (dd, *J* = 9.5, 7.0 Hz, 1H), 3.78-3.70 (m, 1H), 3.16-3.09 (m, 1H), 2.63 (dd, *J* = 18.2, 10.7 Hz, 1H), 2.12 (dd, *J* = 18.2, 3.1 Hz, 1H), 1.83 (s, 3H).). ^{13}C NMR (CD_2Cl_2, 151 MHz) δ (ppm) 207.68, 154.71, 139.16, 139.08, 139.02, 133.11, 131.97, 129.39, 129.32, 128.89, 128.84, 128.75, 128.55, 128.32, 128.21, 128.07, 128.03, 112.64, 112.33, 110.74, 102.86, 81.73, 81.16, 77.82, 74.89, 73.95, 69.66, 56.40, 44.93, 39.68, 30.15. HRMS (ESI$^+$) calc. for $[M+H]^+$ ($C_{37}H_{37}NO_5Na^+$), 598.2564, found:598.2567; $[\alpha]_D^{20}$ = +27.2 (*c* = 0.92, CH_2Cl_2).

1-((2*S*,3*R*,4*R*,5*R*)-4-(benzyloxy)-5-((benzyloxy)methyl)-2-(5-methyl-2-phenyl-1H-indol-3-yl)tetrahydrofuran-3-yl)propan-2-one (4c)

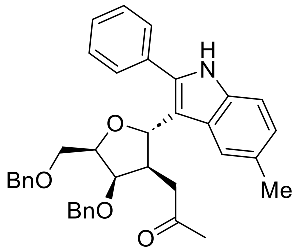

4c

The title product compound is prepared according to the general procedure B with 0.1 mmol scale in CH_2Cl_2 at room temperature for 1 h and isolated by flash column chromatography (3:1 Pentane: Ethyl Acetate) giving a colorless oil (52.8 mg, 94% yield, α/β ratio > 20:1). ^1H NMR (CD_2Cl_2, 600 MHz) δ (ppm) 8.33 (s, 1H), 7.55-7.48 (m, 3H), 7.46-7.39 (m, 3H), 7.39-7.29 (m, 8H), 7.29-7.24 (m, 3H), 7.06-6.99 (m, 1H), 5.04 (d, *J* = 11.2 Hz, 1H), 4.74-4.68 (m, 1H), 4.64 (d, *J* = 11.4 Hz, 1H), 4.58 (q, *J* = 11.8 Hz, 2H), 4.50 (t, *J* = 4.0 Hz, 1H), 4.39 (d, *J* = 11.5 Hz, 1H), 3.84 (dd, *J* = 9.5, 6.9 Hz, 1H), 3.74 (dd, *J* = 9.4, 6.0 Hz, 1H), 3.21-3.12 (m, 1H), 2.64 (dd, *J* = 18.2, 10.8 Hz, 1H), 2.48 (s, 3H), 2.13 (dd, *J* = 18.2, 3.1 Hz, 1H), 1.83 (s, 3H). ^{13}C NMR (CD_2Cl_2, 126 MHz) δ (ppm) 207.81, 139.12, 139.03, 138.49, 135.20, 133.14, 129.71, 129.41, 129.32, 128.89, 128.85, 128.70, 128.54, 128.35, 128.20, 128.08, 127.67, 124.52, 120.30, 111.35, 110.62, 81.70, 81.23, 77.85, 74.90, 73.98, 69.71, 45.16, 39.74, 30.16, 21.93. HRMS (ESI$^+$) calc. for $[M+H]^+$ ($C_{37}H_{37}NO_4Na^+$), 582.2615, found:582.2620; $[\alpha]_D^{20}$ = +26.6 (*c* = 0.47, CH_2Cl_2).

1-((2*S*,3*R*,4*R*,5*R*)-4-(benzyloxy)-5-((benzyloxy)methyl)-2-(5-fluoro-2-phenyl-1H-indol-3-yl)tetrahydrofuran-3-yl)propan-2-one (4d)

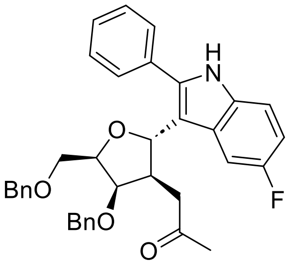

4d

The title product compound is prepared according to the general procedure B with 0.1 mmol scale in CH_2Cl_2 at room temperature for 1 h and isolated by flash column chromatography (3:1 Pentane: Ethyl Acetate) giving a colorless oil (46 mg, 82% yield, α/β ratio > 20:1). ^{1}H NMR (CD_2Cl_2, 700 MHz) δ (ppm) 8.50 (s, 1H), 7.48-7.44 (m, 2H), 7.44-7.40 (m, 3H), 7.39-7.37 (m, 1H), 7.35-7.31 (m, 6H), 7.31-7.23 (m, 5H), 6.96-6.90 (m, 1H), 5.02 (d, *J* = 11.2 Hz, 1H), 4.65 (td, *J* = 6.4, 3.6 Hz, 1H), 4.63-4.59 (m, 1H), 4.59-4.52 (m, 2H), 4.46 (t, *J* = 4.0 Hz, 1H), 4.37 (d, *J* = 11.4 Hz, 1H), 3.81 (dd, *J* = 9.6, 6.7 Hz, 1H), 3.72 (dd, *J* = 9.5, 6.1 Hz, 1H), 3.09-3.02 (m, 1H), 2.62 (dd, *J* = 18.2, 10.6 Hz, 1H), 2.10 (dd, *J* = 18.2, 3.2 Hz, 1H), 1.83 (s, 3H). ^{13}C NMR (CD_2Cl_2, 176 MHz) δ (ppm) 207.61, 158.91, 157.58, 140.15, 139.00, 138.95, 133.35, 132.63, 129.38, 129.03, 128.90, 128.84, 128.54, 128.33, 128.24, 128.08, 127.76, 127.70, 112.46, 112.40, 111.34, 111.31, 111.07, 110.92, 105.68, 105.55, 81.78, 81.13, 77.64, 74.92, 73.96, 69.65, 45.22, 39.61, 30.14. HRMS (ESI$^+$) calc. for [M+H]$^+$ ($C_{36}H_{34}FNO_4Na^+$), 586.2364, found:586.2359; $[\alpha]_D^{20}$ = +27.7 (*c* = 0.44, CH_2Cl_2).

1-((2*S*,3*R*,4*R*,5*R*)-4-(benzyloxy)-5-((benzyloxy)methyl)-2-(5-chloro-2-phenyl-1H-indol-3-yl)tetrahydrofuran-3-yl)propan-2-one (4e)

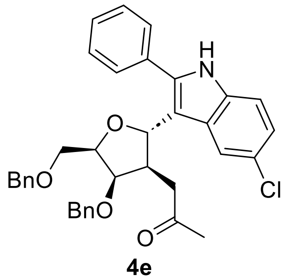

4e

The title product compound is prepared according to the general procedure B with 0.1 mmol scale in CH_2Cl_2 at room temperature for 1 h and isolated by flash column chromatography (3:1 Pentane: Ethyl Acetate) giving a colorless oil (56 mg, 96% yield, α/β ratio > 20:1). ^{1}H NMR (CD_2Cl_2, 700 MHz) δ (ppm) 8.57 (s, 1H), 7.69 (d, *J* = 2.1 Hz, 1H), 7.46-7.44 (m, 2H), 7.43-7.39 (m, 3H), 7.35-7.31 (m, 6H), 7.31-7.24 (m, 5H), 7.14-7.11 (m, 1H), 5.01 (d, *J* = 11.2 Hz, 1H), 4.66 (dt, *J* = 6.5, 3.2 Hz, 1H), 4.61 (d, *J* = 11.4 Hz, 1H), 4.58-4.50 (m, 2H), 4.46 (t, *J* = 4.0 Hz, 1H), 4.36 (d, *J* = 11.4 Hz, 1H), 3.79 (dd, *J* = 9.6, 6.7 Hz, 1H), 3.71 (dd, *J* = 9.6, 6.1 Hz, 1H), 3.08-3.00 (m, 1H), 2.62 (dd, *J* = 18.2, 10.6 Hz, 1H), 2.09 (dd, *J* = 18.2, 3.2 Hz, 1H), 1.83 (s, 3H). ^{13}C NMR (CD_2Cl_2, 176 MHz) δ (ppm) 207.59, 139.76, 138.99, 138.92, 135.19, 132.45, 129.41, 129.40, 129.10, 128.90, 128.84, 128.53, 128.48, 128.33, 128.33, 128.24, 128.08, 125.89, 123.02, 119.99, 112.82, 110.90, 81.76, 81.11, 77.54, 74.93, 73.95, 69.62, 45.42, 39.59, 30.16. HRMS (ESI$^+$) calc. for [M+H]$^+$ ($C_{36}H_{34}ClNO_4Na^+$), 602.2069, found:602.2077; $[\alpha]_D^{20}$ = +28.8 (*c* = 0.50, CH_2Cl_2).

1-((2S,3R,4R,5R)-4-(benzyloxy)-5-((benzyloxy)methyl)-2-(5-bromo-2-phenyl-1H-indol-3-yl)tetrahydrofuran-3-yl)propan-2-one (4f)

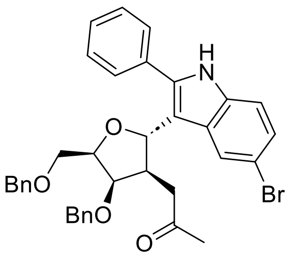

4f

The title product compound is prepared according to the general procedure B with 0.1 mmol scale in CH_2Cl_2 at room temperature for 1 h and isolated by flash column chromatography (2:1 Pentane: Ethyl Acetate) giving a colorless oil (40 mg, 64% yield, α/β ratio > 20:1). ^1H NMR (CD_2Cl_2, 500 MHz) δ (ppm) 8.50 (s, 1H), 7.87-7.80 (m, 1H), 7.49-7.46 (m, 2H), 7.43 (dd, *J* = 7.2, 1.0 Hz, 3H), 7.35-7.32 (m, 5H), 7.32-7.28 (m, 3H), 7.27-7.23 (m, 4H), 5.00 (d, *J* = 11.2 Hz, 1H), 4.65 (td, *J* = 6.4, 3.6 Hz, 1H), 4.61 (d, *J* = 11.4 Hz, 1H), 4.55 (q, *J* = 11.8 Hz, 2H), 4.46 (t, *J* = 4.0 Hz, 1H), 4.36 (d, *J* = 11.4 Hz, 1H), 3.79 (dd, *J* = 9.5, 6.7 Hz, 1H), 3.71 (dd, *J* = 9.5, 6.1 Hz, 1H), 3.06-2.98 (m, 1H), 2.61 (dd, *J* = 18.2, 10.6 Hz, 1H), 2.09 (dd, *J* = 18.2, 3.2 Hz, 1H), 1.83 (s, 3H). ^{13}C NMR (CD_2Cl_2, 126 MHz) δ (ppm) 207.54, 139.56, 139.01, 138.95, 135.44, 132.44, 129.43, 129.20, 129.14, 128.90, 128.85, 128.53, 128.34, 128.24, 128.08, 125.65, 123.06, 113.59, 113.23, 110.90, 81.74, 81.11, 77.51, 74.93, 73.96, 69.62, 45.47, 39.60, 30.18. HRMS (ESI$^+$) calc. for $[M+H]^+$ ($C_{36}H_{34}BrNO_4Na^+$), 646.1563, found:646.1578; $[\alpha]_D^{20}$ = +12.0 (*c* = 0.30, CH_2Cl_2).

1-((2S,3R,4R,5R)-4-(benzyloxy)-5-((benzyloxy)methyl)-2-(4-methyl-2-phenyl-1H-indol-3-yl)tetrahydrofuran-3-yl)propan-2-one (4g)

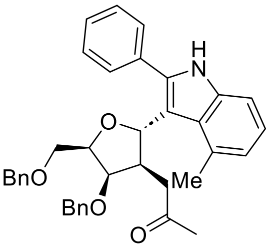

4g

The title product compound is prepared according to the general procedure B with 0.1 mmol scale in CH_2Cl_2 at room temperature for 1 h and isolated by flash column chromatography (2:1 Pentane: Ethyl Acetate) giving a colorless oil (45 mg, 81% yield, α/β ratio > 20:1). ^1H NMR (CD_2Cl_2, 500 MHz) δ (ppm) 8.43 (s, 1H), 7.60 – 7.52 (m, 2H), 7.44 (dd, J = 5.0, 2.0 Hz, 3H), 7.39-7.26 (m, 8H), 7.25-7.18 (m, 3H), 7.09 (dd, *J* = 8.1, 7.1 Hz, 1H), 6.93 (dd, *J* = 7.1, 1.0 Hz, 1H), 5.29 (d, *J* = 11.2 Hz, 1H), 4.61-4.52 (m, 3H), 4.50-4.43 (m, 1H), 4.33 (d, *J* = 11.6 Hz, 1H), 4.30 (t, *J* = 4.7 Hz, 1H), 3.80 (dd, *J* = 9.6, 6.6 Hz, 1H), 3.72 (dd, *J* = 9.6, 6.1 Hz, 1H), 2.75 (s, 3H), 2.70 (d, *J* = 13.5 Hz, 1H), 2.50 (dd, *J* = 18.0, 10.6 Hz, 1H), 2.01 (dd, *J* = 18.0, 3.2 Hz, 1H), 1.76 (s, 3H). ^{13}C NMR (CD_2Cl_2, 126 MHz) δ (ppm) 207.76, 139.18, 139.13, 139.01, 136.93, 133.97, 131.36, 130.21, 129.09, 128.91, 128.83, 128.28, 128.18, 128.06, 128.03, 127.57, 122.99, 122.76, 111.07, 109.30, 80.83, 80.55, 77.53, 74.67, 73.87, 69.84, 46.28, 39.61, 30.05, 22.73. HRMS (ESI$^+$) calc. for $[M+H]^+$ ($C_{37}H_{37}NO_4Na^+$), 582.2615, found:586.2615; $[\alpha]_D^{20}$ = +59.7 (*c* = 0.37, CH_2Cl_2).

1-((2S,3R,4R,5R)-4-(benzyloxy)-5-((benzyloxy)methyl)-2-(6-methyl-2-phenyl-1H-indol-3-yl)tetrahydrofuran-3-yl)propan-2-one (4h)

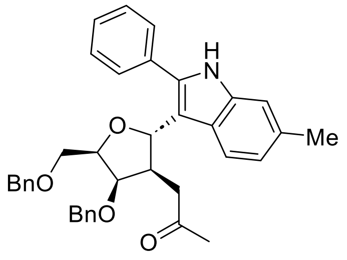

4h

The title product compound is prepared according to the general procedure B with 0.1 mmol scale in CH_2Cl_2 at room temperature for 1 h and isolated by flash column chromatography (2:1 Pentane: Ethyl Acetate) giving a colorless oil (46 mg, 82% yield, α/β ratio > 20:1). ^1H NMR (CD_2Cl_2, 500 MHz) δ (ppm) 8.21 (s, 1H), 7.60 (d, *J* = 8.2 Hz, 1H), 7.54-7.50 (m, 2H), 7.48-7.43 (m, 2H), 7.42-7.39 (m, 1H), 7.37-7.23 (m, 10H), 7.17 (s, 1H), 6.96 (dd, *J* = 8.2, 1.5 Hz, 1H), 5.01 (d, *J* = 11.1 Hz, 1H), 4.68-4.63 (m, 1H), 4.62 (d, *J* = 11.4 Hz, 1H), 4.56 (q, *J* = 11.8 Hz, 2H), 4.45 (t, *J* = 3.9 Hz, 1H), 4.37 (d, *J* = 11.4 Hz, 1H), 3.81 (dd, *J* = 9.5, 6.9 Hz, 1H), 3.72 (dd, *J* = 9.5, 6.0 Hz, 1H), 3.17-3.11 (m, 1H), 2.63 (dd, *J* = 18.2, 10.7 Hz, 1H), 2.45 (s, 3H), 2.12 (dd, *J* = 18.2, 3.2 Hz, 1H), 1.82 (s, 3H). ^{13}C NMR (CD_2Cl_2, 126 MHz) δ (ppm) 207.69, 139.11, 139.06, 137.71, 137.32, 133.16, 132.88, 129.36, 128.88, 128.84, 128.66, 128.54, 128.34, 128.20, 128.06, 125.25, 122.20, 120.36, 111.54, 111.06, 81.72, 81.24, 77.80, 74.89, 73.96, 69.70, 45.29, 39.72, 30.15, 21.94. HRMS (ESI$^+$) calc. for $[M+H]^+$ ($C_{37}H_{37}NO_4Na^+$), 582.2615, found:586.2605; $[\alpha]_D^{20}$ = +44.2 (*c* = 0.24, CH_2Cl_2).

1-((2*S*,3*R*,4*R*,5*R*)-4-(benzyloxy)-5-((benzyloxy)methyl)-2-(7-methyl-2-phenyl-1H-indol-3-yl)tetrahydrofuran-3-yl)propan-2-one (4i)

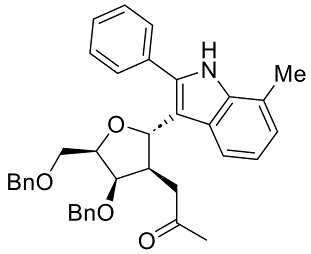

4i

The title product compound is prepared according to the general procedure B with 0.1 mmol scale in CH_2Cl_2 at room temperature for 1 h and isolated by flash column chromatography (3:1 Pentane: Ethyl Acetate) giving a colorless oil (44 mg, 79% yield, α/β ratio > 20:1). ^1H NMR (CD_2Cl_2, 700 MHz) δ (ppm) 8.21 (s, 1H), 7.61-7.55 (m, 3H), 7.52-7.48 (m, 2H), 7.46-7.42 (m, 1H), 7.38-7.32 (m, 6H), 7.31-7.25 (m, 4H), 7.07-7.01 (m, 2H), 5.03 (d, *J* = 11.1 Hz, 1H), 4.68-4.64 (m, 1H), 4.63 (d, *J* = 11.4 Hz, 1H), 4.61-4.54 (m, 2H), 4.46 (t, *J* = 4.0 Hz, 1H), 4.37 (d, *J* = 11.4 Hz, 1H), 3.82 (dd, *J* = 9.5, 6.9 Hz, 1H), 3.73 (dd, *J* = 9.5, 6.0 Hz, 1H), 3.15 (tdd, *J* = 10.9, 4.4, 3.1 Hz, 1H), 2.64 (dd, *J* = 18.2, 10.7 Hz, 1H), 2.50 (s, 3H), 2.13 (dd, *J* = 18.2, 3.1 Hz, 1H), 1.84 (s, 3H). ^{13}C NMR (CD_2Cl_2, 176 MHz) δ (ppm) 207.70, 139.10, 139.06, 138.08, 136.36, 133.17, 129.57, 129.37, 128.88, 128.84, 128.54, 128.34, 128.20, 128.06, 126.94, 123.45, 120.98, 120.71, 118.43, 111.77, 81.74, 81.24, 77.83, 74.89, 73.96, 69.70, 45.33, 39.73, 30.15, 16.98. HRMS (ESI$^+$) calc. for $[M+H]^+$ ($C_{37}H_{37}NO_4Na^+$), 582.2615, found:586.2614; $[\alpha]_D^{20}$ = +23.8 (*c* = 0.37, CH_2Cl_2).

1-((2*S*,3*R*,4*R*,5*R*)-4-(benzyloxy)-5-((benzyloxy)methyl)-2-(6-chloro-2-phenyl-1H-indol-3-yl)tetrahydrofuran-3-yl)propan-2-one (4j)

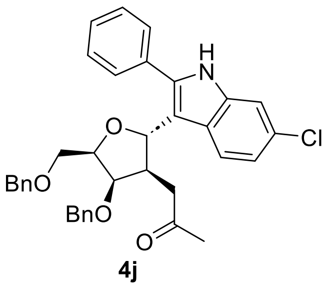

4j

The title product compound is prepared according to the general procedure B with 0.1 mmol scale in CH_2Cl_2 at room temperature for 1 h and isolated by flash column chromatography (3:1 Pentane: Ethyl Acetate) giving a colorless oil (42 mg, 72% yield, α/β ratio > 20:1). ^{1}H NMR (CD_2Cl_2, 700 MHz) δ (ppm) 8.55 (s, 1H), 7.65 (d, J = 8.5 Hz, 1H), 7.47-7.44 (m, 2H), 7.44-7.39 (m, 3H), 7.35-7.23 (m, 11H), 7.09 (dd, *J* = 8.5, 1.9 Hz, 1H), 5.03 (d, *J* = 11.2 Hz, 1H), 4.66-4.63 (m, 1H), 4.61 (d, *J* = 11.4 Hz, 1H), 4.58-4.51 (m, 2H), 4.45 (t, *J* = 4.0 Hz, 1H), 4.36 (d, *J* = 11.4 Hz, 1H), 3.79 (dd, *J* = 9.6, 6.7 Hz, 1H), 3.71 (dd, *J* = 9.6, 6.1 Hz, 1H), 3.11-3.03 (m, 1H), 2.62 (dd, *J* = 18.2, 10.6 Hz, 1H), 2.09 (dd, *J* = 18.2, 3.2 Hz, 1H), 1.82 (s, 3H). ^{13}C NMR (CD_2Cl_2, 176 MHz) δ (ppm) 207.65, 139.09, 138.98, 138.93, 137.22, 132.47, 129.40, 129.38, 129.03, 128.90, 128.84, 128.55, 128.49, 128.33, 128.25, 128.09, 126.04, 121.63, 120.96, 111.59, 111.27, 81.83, 81.11, 77.58, 74.91, 73.96, 69.66, 45.48, 39.58, 30.15. HRMS (ESI$^+$) calc. for $[M+H]^+$ ($C_{36}H_{34}ClNO_4Na^+$), 602.2069, found:602.2063; $[\alpha]_D^{20}$ = +32.1 (*c* = 0.38, CH_2Cl_2).

1-((2*S*,3*R*,4*R*,5*R*)-4-(benzyloxy)-5-((benzyloxy)methyl)-2-(6-bromo-2-phenyl-1H-indol-3-yl)tetrahydrofuran-3-yl)propan-2-one (4k)

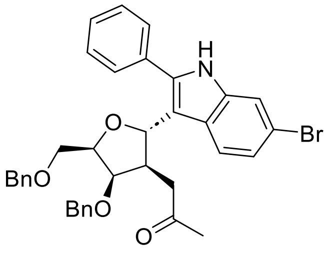

4k

The title product compound is prepared according to the general procedure B with 0.1 mmol scale in CH_2Cl_2 at room temperature for 1 h and isolated by flash column chromatography (3:1 Pentane: Ethyl Acetate) giving a colorless oil (46 mg, 74% yield, α/β ratio > 20:1). ^{1}H NMR (CD_2Cl_2, 700 MHz) δ (ppm) 8.57 (s, 1H), 7.60 (d, *J* = 8.5 Hz, 1H), 7.48 (d, *J* = 1.8 Hz, 1H), 7.45 (dd, *J* = 7.5, 2.1 Hz, 2H), 7.44-7.40 (m, 3H), 7.35-7.31 (m, 6H), 7.31-7.27 (m, 2H), 7.26-7.24 (m, 2H), 7.22 (dd, *J* = 8.5, 1.8 Hz, 1H), 5.33-5.31 (m, 2H), 5.03 (d, *J* = 11.2 Hz, 1H), 4.66-4.62 (m, 1H), 4.61 (d, *J* = 11.4 Hz, 1H), 4.58-4.51 (m, 2H), 4.45 (t, *J* = 4.0 Hz, 1H), 4.36 (d, *J* = 11.4 Hz, 1H), 3.79 (dd, *J* = 9.6, 6.7 Hz, 1H), 3.70 (dd, *J* = 9.6, 6.1 Hz, 1H), 3.12-3.01 (m, 1H), 2.62 (dd, *J* = 18.2, 10.6 Hz, 1H), 2.09 (dd, *J* = 18.2, 3.2 Hz, 1H). ^{13}C NMR (CD_2Cl_2, 176 MHz) δ (ppm) 207.64, 139.01, 138.97, 138.92, 137.62, 132.42, 129.40, 129.38, 129.05, 128.90, 128.84, 128.55, 128.33, 128.24, 128.08, 126.34, 123.56, 121.96, 116.15, 114.59, 111.29, 81.82, 81.10, 77.55, 74.90, 73.95, 69.65, 45.47, 39.57, 30.15. HRMS (ESI$^+$) calc. for $[M+H]^+$ ($C_{36}H_{34}BrNO_4Na^+$), 646.1563, found:646.1573; $[\alpha]_D^{20}$ = +32.1 (*c* = 0.38, CH_2Cl_2).

1-((2*S*,3*R*,4*R*,5*R*)-4-(benzyloxy)-5-((benzyloxy)methyl)-2-(7-bromo-2-phenyl-1H-indol-3-yl)tetrahydrofuran-3-yl)propan-2-one (4l)

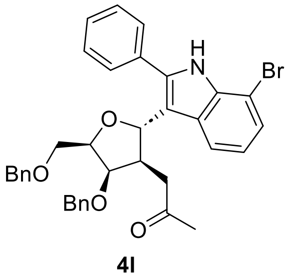

4l

The title product compound is prepared according to the general procedure B with 0.1 mmol scale in CH_2Cl_2 at room temperature for 1 h and isolated by flash column chromatography (3:1 Pentane: Ethyl Acetate) giving a colorless oil (32 mg, 51% yield, α/β ratio > 20:1). ^{1}H NMR (CD_2Cl_2, 700 MHz) δ (ppm) 8.43 (s, 1H), 7.70 (d, *J* = 7.9 Hz, 1H), 7.61-7.57 (m, 2H), 7.51 (t, *J* = 7.5 Hz, 2H), 7.48-7.44 (m, 1H), 7.40-7.35 (m, 4H), 7.34-7.31 (m, 3H), 7.30-7.27 (m, 2H), 7.26-7.23 (m, 2H), 7.04 (t, *J* = 7.8 Hz, 1H), 5.01 (d, *J* = 11.2 Hz, 1H), 4.66-4.63 (m, 1H), 4.63-4.54 (m, 3H), 4.45 (t, *J* = 4.0 Hz, 1H), 4.36 (d, *J* = 11.4 Hz, 1H), 3.81 (dd, *J* = 9.6, 6.8 Hz, 1H), 3.72 (dd, *J* = 9.6, 6.0 Hz, 1H), 3.10-3.06 (m, 1H), 2.63 (dd, *J* = 18.2, 10.6 Hz, 1H), 2.11 (dd, *J* = 18.2, 3.1 Hz, 1H), 1.84 (s, 3H). ^{13}C NMR (CD_2Cl_2, 176 MHz) δ (ppm) 207.51, 139.01, 139.00, 138.98, 135.43, 132.36, 129.63, 129.47, 129.28, 128.90, 128.86, 128.62, 128.55, 128.55, 128.35, 128.24, 128.09, 125.21, 121.73, 120.09, 112.71, 105.01, 81.86, 81.13, 77.70, 74.93, 73.98, 69.65, 45.55, 39.56, 30.17. HRMS (ESI$^+$) calc. for [M+H]$^+$ ($C_{36}H_{34}BrNO_4Na^+$), 646.1563, found:646.1577; $[\alpha]_D^{20}$ = +27.4 (*c* = 0.27, CH_2Cl_2).

1-((2*S*,3*R*,4*R*,5*R*)-4-(benzyloxy)-5-((benzyloxy)methyl)-2-(2-(4-methoxyphenyl)-1H-indol-3-yl)tetrahydrofuran-3-yl)propan-2-one (4m)

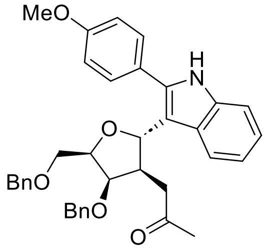

4m

The title product compound is prepared according to the general procedure B with 0.1 mmol scale in CH_2Cl_2 at room temperature for 1 h and isolated by flash column chromatography (2:1 Pentane: Ethyl Acetate) giving a colorless oil (39 mg, 68% yield, α/β ratio > 20:1). ^{1}H NMR (CD_2Cl_2, 700 MHz) δ (ppm) 8.38 (s, 1H), 7.72 (d, *J* = 7.9 Hz, 1H), 7.42-7.39 (m, 2H), 7.37-7.32 (m, 7H), 7.31-7.25 (m, 4H), 7.19-7.15 (m, 1H), 7.13-7.09 (m, 1H), 6.92 (d, *J* = 8.7 Hz, 2H), 5.01 (d, *J* = 11.2 Hz, 1H), 4.70-4.65 (m, 1H), 4.62 (d, *J* = 11.3 Hz, 1H), 4.60-4.52 (m, 2H), 4.47 (t, *J* = 3.9 Hz, 1H), 4.37 (d, *J* = 11.4 Hz, 1H), 3.86-3.79 (m, 4H), 3.74 (dd, *J* = 9.5, 6.0 Hz, 1H), 3.18-3.12 (m, 1H), 2.62 (dd, *J* = 18.2, 10.7 Hz, 1H), 2.10 (dd, *J* = 18.2, 3.1 Hz, 1H), 1.82 (s, 3H). ^{13}C NMR (CD_2Cl_2, 176 MHz) δ (ppm) 207.73, 160.30, 139.08, 139.02, 138.41, 136.70, 130.65, 128.90, 128.84, 128.56, 128.33, 128.22, 128.05, 127.50, 125.23, 122.60, 120.50, 120.33, 114.73, 111.57, 110.49, 81.72, 81.25, 77.92, 74.93, 73.95, 69.72, 55.85, 45.16, 39.70, 30.13. HRMS (ESI$^+$) calc. for [M+H]$^+$ ($C_{37}H_{37}NO_5Na^+$), 598.2564, found:598.2564; $[\alpha]_D^{20}$ = +22.8 (*c* = 0.32, CH_2Cl_2).

1-((2*S*,3*R*,4*R*,5*R*)-4-(benzyloxy)-5-((benzyloxy)methyl)-2-(1-methyl-2-phenyl-1H-indol-3-yl)tetrahydrofuran-3-yl)propan-2-one (4n)

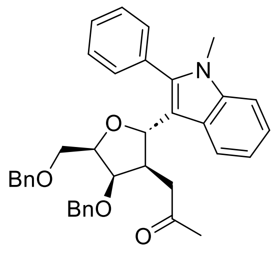

4n

The title product compound is prepared according to the general procedure B with 0.1 mmol scale in CH_2Cl_2 at room temperature for 1 h and isolated by flash column chromatography (3:1 Pentane: Ethyl Acetate) giving a colorless oil (36 mg, 64% yield, α/β ratio > 20:1). ^1H NMR (CD_2Cl_2, 700 MHz) δ (ppm) 7.73 (d, J = 7.9 Hz, 1H), 7.55-7.48 (m, 3H), 7.43-7.21 (m, 14H), 7.17-7.12 (m, 1H), 4.73 (d, *J* = 11.1 Hz, 1H), 4.62-4.51 (m, 4H), 4.42 (t, *J* = 3.9 Hz, 1H), 4.33 (s, 1H), 3.78 (dd, *J* = 9.5, 7.0 Hz, 1H), 3.68 (dd, *J* = 9.5, 5.9 Hz, 1H), 3.53 (s, 3H), 3.14-3.00 (m, 1H), 2.56 (dd, *J* = 18.2, 10.7 Hz, 1H), 2.14 (dd, *J* = 18.2, 3.1 Hz, 1H), 1.87 (s, 3H). ^{13}C NMR (CD_2Cl_2, 176 MHz) δ (ppm) 207.67, 140.91, 139.08, 138.19, 131.87, 131.43, 129.08, 128.97, 128.87, 128.84, 128.57, 128.33, 128.19, 128.06, 126.30, 122.39, 120.42, 120.10, 111.62, 110.16, 81.67, 81.21, 78.23, 74.85, 73.93, 69.66, 45.45, 39.70, 31.10, 30.17. HRMS (ESI$^+$) calc. for $[M+H]^+$ ($C_{37}H_{37}NO_4Na^+$), 582.2615, found:582.2605; $[\alpha]_D^{20}$ = -26.4 (*c* = 0.33, CH_2Cl_2).

1-((2*R*,3*S*,4*S*,5*S*)-4-(benzyloxy)-5-((benzyloxy)methyl)-2-(2-phenyl-1H-indol-3-yl)tetrahydrofuran-3-yl)propan-2-one (4o)

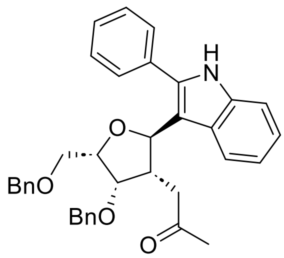

4o

The title product compound is prepared according to the general procedure B with 0.1 mmol scale in CH_2Cl_2 at room temperature for 1 h and isolated by flash column chromatography (3:1 Pentane: Ethyl Acetate) giving a colorless oil (45 mg, 82% yield, α/β ratio > 20:1). ^1H NMR (CD_2Cl_2, 500 MHz) δ (ppm) 8.41 (s, 1H), 7.75 (d, *J* = 7.8 Hz, 1H), 7.54-7.49 (m, 2H), 7.47-7.40 (m, 3H), 7.39-7.25 (m, 11H), 7.22-7.17 (m, 1H), 7.16-7.10 (m, 1H), 5.06 (dd, *J* = 11.4, 3.1 Hz, 1H), 4.72-4.66 (m, 1H), 4.63 (d, *J* = 11.4 Hz, 1H), 4.57 (q, *J* = 11.8 Hz, 2H), 4.50-4.44 (m, 1H), 4.38 (dd, *J* = 11.4, 1.6 Hz, 1H), 3.86-3.79 (m, 1H), 3.77-3.69 (m, 1H), 3.21-3.11 (m, 1H), 2.64 (dd, *J* = 18.2, 10.7 Hz, 1H), 2.12 (dd, *J* = 18.2, 3.1 Hz, 1H), 1.82 (s, 3H). ^{13}C NMR (CD_2Cl_2, 126 MHz) δ (ppm) 207.72, 139.09, 139.02, 136.87, 132.98, 129.46, 129.35, 128.90, 128.85, 128.81, 128.54, 128.34, 128.21, 128.07, 127.42, 122.88, 120.72, 120.43, 111.70, 111.68, 111.17, 81.77, 81.23, 77.82, 74.90, 73.96, 69.70, 45.31, 39.70, 30.14. HRMS (ESI$^+$) calc. for $[M+H]^+$ ($C_{36}H_{35}NO_4Na^+$), 568.2458, found:568.2466; $[\alpha]_D^{20}$ = -23.4 (*c* = 0.41, CH_2Cl_2).

((2*R*,3*R*,4*R*,5*S*)-3-(benzyloxy)-4-(2-oxopropyl)-5-(2-phenyl-1H-indol-3-yl)tetrahydrofuran-2-yl)methyl acetate (4p)

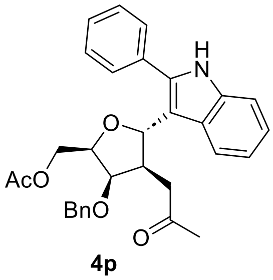

4p

The title product compound is prepared according to the general procedure B with 0.1 mmol scale in CH_2Cl_2 at room temperature for 1 h and isolated by flash column chromatography (3:1 Pentane: Ethyl Acetate) giving a colorless oil (31 mg, 62% yield, α/β ratio > 20:1). ^1H NMR (CD_2Cl_2, 700 MHz) δ (ppm) 8.40 (s, 1H), 7.72 (d, *J* = 8.2 Hz, 1H), 7.57-7.52 (m, 2H), 7.50-7.45 (m, 2H), 7.45-7.41 (m, 1H), 7.41-7.37 (m, 1H), 7.37-7.33 (m, 2H), 7.32-7.26 (m, 3H), 7.23-7.18 (m, 1H), 7.13-7.11 (m, 1H), 5.07 (d, *J* = 11.2 Hz, 1H), 4.68-4.62 (m, 1H), 4.57 (d, *J* = 11.3 Hz, 1H), 4.51 (t, *J* = 4.2 Hz, 1H), 4.37 (d, *J* = 11.3 Hz, 1H), 4.35-4.28 (m, 2H), 3.20-3.13 (m, 1H), 2.63 (dd, *J* = 18.4, 10.8 Hz, 1H), 2.15 (dd, *J* = 18.4, 3.1 Hz, 1H), 2.03 (s, 3H), 1.85 (s, 3H). ^{13}C NMR (CD_2Cl_2, 176 MHz) δ (ppm) 207.76, 171.25, 138.64, 138.60, 136.85, 129.43, 129.42, 128.97, 128.91, 128.55, 128.38, 127.37, 122.93, 120.66, 120.47, 111.70, 110.57, 81.32, 80.35, 77.88, 75.18, 64.04, 45.20, 39.55, 30.16, 21.28. HRMS (ESI$^+$) calc. for $[M+H]^+$ ($C_{31}H_{31}NO_5Na^+$), 520.2094, found:520.2097; $[\alpha]_D^{20}$ = +38.8 (*c* = 0.24, CH_2Cl_2).

1-((2*R*,3*S*,4*S*,5*R*)-4-(benzyloxy)-5-((benzyloxy)methyl)-2-(2-phenyl-1H-indol-3-yl)tetrahydrofuran-3-yl)propan-2-one (4q)

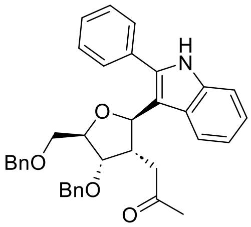

4q

The title product compound is prepared according to the general procedure B with 0.1 mmol scale in CH_2Cl_2 at room temperature for 1 h and isolated by flash column chromatography (3:1 Pentane: Ethyl Acetate) giving a colorless oil (49 mg, 90% yield, β/α ratio > 20:1). ^1H NMR (CD_2Cl_2, 700 MHz) δ (ppm) 8.38 (s, 1H), 7.87 (d, *J* = 8.0 Hz, 1H), 7.57-7.52 (m, 2H), 7.49-7.37 (m, 7H), 7.36-7.28 (m, 5H), 7.27-7.23 (m, 2H), 7.19-7.14 (m, 1H), 7.01-6.95 (m, 1H), 4.98 (d, *J* = 11.0 Hz, 1H), 4.66 (dd, *J* = 11.8 Hz, 2H), 4.55 (d, *J* = 11.5 Hz, 1H), 4.34 (d, *J* = 11.5 Hz, 1H), 4.29 (dd, *J* = 6.5, 2.2 Hz, 1H), 4.24-4.22 (m, 1H), 3.73 (qd, *J* = 9.8, 4.9 Hz, 2H), 3.23-3.14 (m, 1H), 2.72 (dd, *J* = 17.8, 10.9 Hz, 1H), 2.07 (dd, *J* = 17.8, 3.6 Hz, 1H), 1.91 (s, 3H). ^{13}C NMR (CD_2Cl_2, 176 MHz) δ (ppm) 207.71, 139.08, 139.00, 138.64, 136.80, 132.96, 129.50, 129.36, 128.91, 128.87, 128.79, 128.51, 128.25, 128.14, 128.08, 127.51, 122.82, 121.56, 120.37, 111.47, 109.98, 83.95, 81.92, 78.87, 74.02, 72.24, 72.16, 44.05, 39.54, 30.31. HRMS (ESI$^+$) calc. for $[M+H]^+$ ($C_{36}H_{35}NO_4Na^+$), 568.2458, found:568.2462; $[\alpha]_D^{20}$ = +38.8 (*c* = 0.24, CH_2Cl_2).

Ethyl 1-((2*S*,3*R*,4*R*,5*R*)-4-(benzyloxy)-5-((benzyloxy)methyl)-3-(2-oxopropyl)tetrahydrofuran-2-yl)pyrrolo[2,1-a]isoquinoline-2-carboxylate (4r)

ethyl 3-((2*S*,3*R*,4*R*,5*R*)-4-(benzyloxy)-5-((benzyloxy)methyl)-3-(2-oxopropyl)tetrahydrofuran-2-yl)pyrrolo[2,1-a]isoquinoline-2-carboxylate (4r')

General procedure : A mixture of glycosyl donor **1a** (1.0 eq. 0.1 mmol), **2s** (1.5 eq.) and cat.**F** (1 mol%) were dissolved in the 1 mL ethyl acetate, the resulting reaction mixture was stirred at room temperature for 1 h. Subsequently, the reaction mixture was filtered over a short silica plug and flushed with dichloromethane. The filtrate was removed under reduced pressure and the residue was analyzed by crude ^{1}H NMR and then subjected to flash column chromatography (dry loading) to give the desired product **4r** and **4r'** with 70% overall yield and 2.4:1 rr (**4r**:**4r'**), α/β ratio > 20:1 for both **4r** and **4r'**.

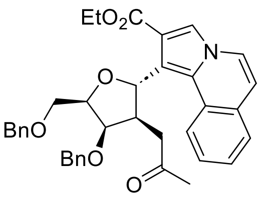

4r

^{1}H NMR (CD_2Cl_2, 500 MHz) δ (ppm) 8.41 (d, J = 8.2 Hz, 1H), 7.83 (s, 1H), 7.68 (d, *J* = 7.3 Hz, 1H), 7.60-7.51 (m, 2H), 7.45-7.40 (m, 1H), 7.38-7.31 (m, 9H), 7.28 (d, *J* = 6.3 Hz, 1H), 6.83 (d, *J* = 7.2 Hz, 1H), 6.08 (d, *J* = 11.7 Hz, 1H), 4.88-4.79 (m, 1H), 4.68 (d, *J* = 11.3 Hz, 1H), 4.57 (dd, *J* = 14.4, 11.2 Hz, 3H), 4.44 (d, *J* = 11.3 Hz, 1H), 4.36-4.27 (m, 2H), 3.89 (dd, *J* = 9.6, 6.6 Hz, 1H), 3.77 (dd, *J* = 9.6, 6.4 Hz, 1H), 3.18 (d, *J* = 10.7 Hz, 1H), 2.88 (dd, *J* = 18.5, 10.5 Hz, 1H), 2.16 (dd, *J* = 18.4, 3.0 Hz, 1H), 1.85 (s, 3H), 1.36 (t, *J* = 7.1 Hz, 3H). ^{13}C NMR (CD_2Cl_2, 126 MHz) δ (ppm) 208.07, 165.24, 139.11, 128.92, 128.84, 128.72, 128.49, 128.38, 128.19, 128.04, 127.85, 127.29, 126.87, 126.22, 125.00, 121.03, 118.48, 116.40, 114.16, 81.34, 75.87, 75.09, 73.98, 69.82, 60.72, 54.43, 54.22, 54.00, 53.78, 53.57, 44.65, 39.77, 30.24, 14.76. HRMS (ESI$^+$) calc. for [M+H]$^+$ ($C_{37}H_{37}NO_6Na^+$), 614.2513, found:614.2519; $[\alpha]_D^{20}$ = +7.5 (*c* = 0.2, CH_2Cl_2).

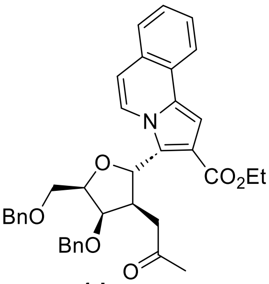

4r'

^{1}H NMR (CD_2Cl_2, 500 MHz) δ (ppm) 8.06-7.97 (m, 2H), 7.59 (d, *J* = 7.3 Hz, 1H), 7.52-7.46 (m, 1H), 7.43-7.40 (m, 1H), 7.38-7.30 (m, 11H), 6.84 (d, J = 7.6 Hz, 1H), 6.05 (d, J = 11.7 Hz, 1H), 4.70 – 4.65 (m, 2H), 4.62 – 4.53 (m, 2H), 4.52 (t, *J* = 4.0 Hz, 1H), 4.43 (d, *J* = 11.3 Hz, 1H), 4.39-4.27 (m, 2H), 3.86 (dd, *J* = 9.6, 6.4 Hz, 1H), 3.76 (dd, *J* = 9.6, 6 .3 Hz, 1H), 3.04-2.95 (m, 1H), 2.87 (dd, *J* = 18.8, 9.9 Hz, 1H), 2.31 (dd, *J* = 18.8, 3.4 Hz, 1H), 1.89 (s, 3H), 1.38 (t, *J* = 7.2 Hz, 3H). ^{13}C NMR (CD_2Cl_2, 126 MHz) δ (ppm) 207.65, 165.50, 138.90, 131.01, 128.97, 128.89, 128.52, 128.43, 128.37, 128.33, 128.17, 127.49, 127.16, 127.11, 126.82, 124.33, 122.70, 118.21, 113.16, 101.99, 81.83, 80.97, 75.89, 75.12, 74.11, 69.60, 60.82, 44.01, 39.41, 30.24, 14.77. HRMS (ESI$^+$) calc. for [M+H]$^+$ ($C_{37}H_{37}NO_6Na^+$), 614.2513, found:614.2519; $[\alpha]_D^{20}$ = +19.0 (*c* = 0.1, CH_2Cl_2).

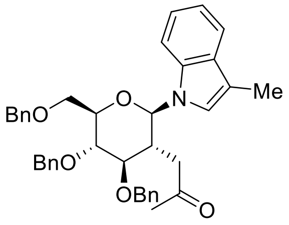

4s

The title product compound is prepared according to the general procedure A with 0.1 mmol scale in CH_2Cl_2 at room temperature for 6 h and isolated by flash column chromatography (3:1 Pentane: Ethyl Acetate) giving a colorless oil (31 mg, 51% yield, β/α = 10:1).^{1}H NMR (,CD_2Cl_2, 400 MHz) δ (ppm) 7.37 (s, 1H), 7.24 – 7.10 (m, 16H), 7.06 – 7.01 (m, 1H), 6.97 (d, J = 7.5 Hz, 1H), 6.86 (s, 1H), 5.52 (d, *J* = 10.2 Hz, 1H), 4.81 (d, *J* = 11.4 Hz, 1H), 4.70 (d, *J* = 10.9 Hz, 1H), 4.54 (d, J = 10.8 Hz, 1H), 4.48 – 4.39 (m, 2H), 4.33 (d, J = 12.0 Hz, 1H), 3.78 – 3.65 (m, 3H), 3.62 – 3.55 (m, 2H), 2.66 – 2.58 (m, 1H), 2.25 (d, J = 3.8 Hz, 1H), 2.15 (s, 3H), 1.94 (dd, *J* = 17.7, 5.7 Hz, 1H), 1.51 (s, 3H). ^{13}C NMR (CD_2Cl_2, 100 MHz) δ (ppm) 206.14, 138.31, 138.14, 138.11, 128.18, 128.15, 128.09, 127.64, 127.55, 127.49, 127.43, 127.36, 121.83, 119.47, 118.82, 82.40, 79.63, 77.43, 74.54, 74.47, 73.23, 68.79, 43.39, 39.89, 29.52, 29.51, 9.18. HRMS (ESI$^+$) calc. for $[M+H]^+$ ($C_{39}H_{41}NO_5Na^+$), 626.2877, found:626.2818.

4. Strain-release C-glycosylation substrate scope with XB catalysis

General procedure: A mixture of glycosyl donor **1** (1.0 eq. 0.1 mmol), trialkoxybenzene or indole **2** (1.5 eq.) and cat.**C** (1 mol%) were dissolved in the 1 mL corresponding solvent (CH_2Cl_2), the resulting reaction mixture was stirred at room temperature for 2 h to 12 h (depending on the substrate). Subsequently, the reaction mixture was filtered over a short silica plug and flushed with dichloromethane. The filtrate was removed under reduced pressure and the residue was analyzed by crude ^{1}H NMR using 1,1,2,2-tetrachloroethane as an internal standard.

Table S5. Substrate scope with XB catalysis

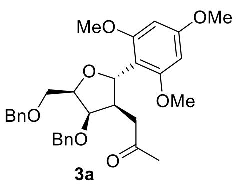

3a
DCM, rt, 2 h
52% yield
α:β = 3.6:1

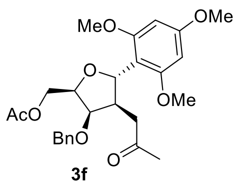

3f
DCM, rt,6 h
29% yield
α:β = 6.7:1

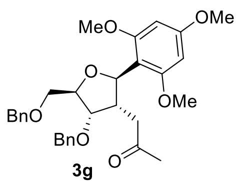

3g
DCM, rt, 6 h
22% yield
β:α = 1.7:1

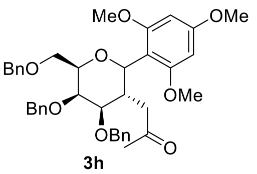

3h
DCM, rt, 6 h
trace

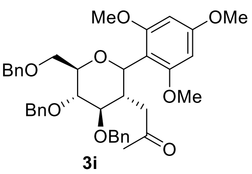

3i
DCM, rt, 6 h
no reaction

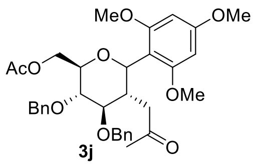

3j
DCM, rt, 6 h
no reaction

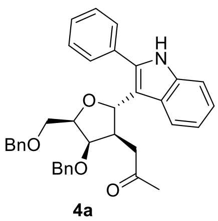

4a
DCM, rt, 12 h
62% yield
α:β = 5.6:1

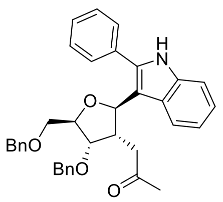

4q
DCM, rt, 3 h
67% yield
β:α = 11:1

General condition: 0.1 mmol **1a**, 1.5 eq **2**, 1 mol% cat.**C**; 1 mL DCM was used, yield and α:β ratio were determined by crude ^{1}H NMR spectra analysis using 1,1,2,2-tetrachloroethane as an internal standard.

5. Mechanistic study

5.1 *In situ* NMR monitoring at -40 °C for intermediate detection

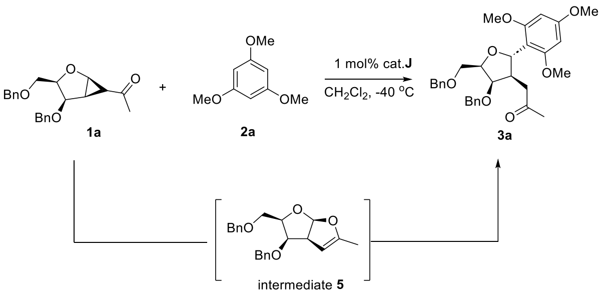

Procedure: A mixture of sugar **1a** (0.2 mmol, 1.0 eq.), **2a** (0.3 mmol) and cat. **J** (1 mol%) were dissolved in 2 mL CH_2Cl_2, the resulting reaction mixture was stirred at -40 °C. One drop of reaction solution at different reaction time periods was taken out and quenched with a drop of Et_3N, and the solvent was removed, to analyzed by ^1H NMR and the intermediate **5** was detected.

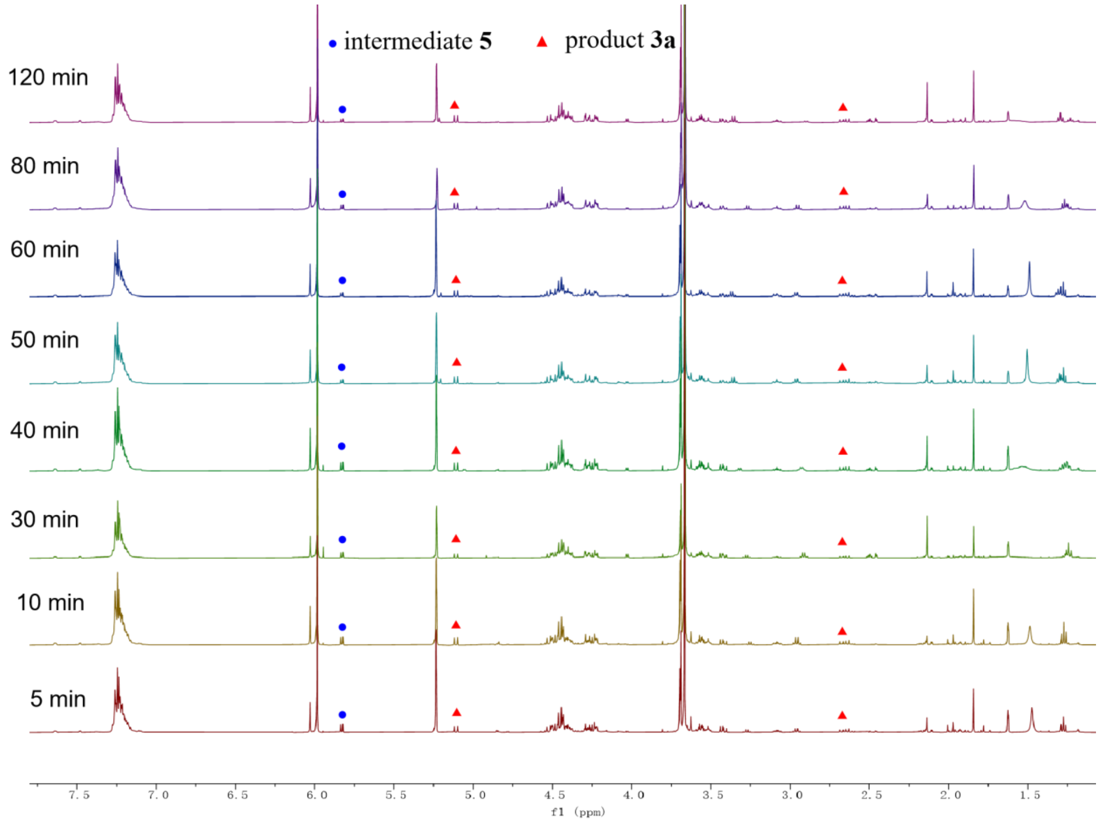

Figure S5. Reaction monitoring by ^1H NMR at -40 °C. Blue crosses denotes the signals of the intermediate **5** and red crosses denotes the signals of the product **3a**.

To further examine the variation trend of the concentration of the product **3a** relative to the intermediate **5**, relative integration of the product **3a** and intermediate **5** was analyzed by ^1H NMR, The results revealed that the concentration of the product relative to intermediates continuously increased over time (**Figure S6**).

t (min)	5	10	30	40	50	60	80	120
Intergration of **3a**/**5**	0.88	0.92	1.02	1.11	1.64	1.74	1.81	2.10

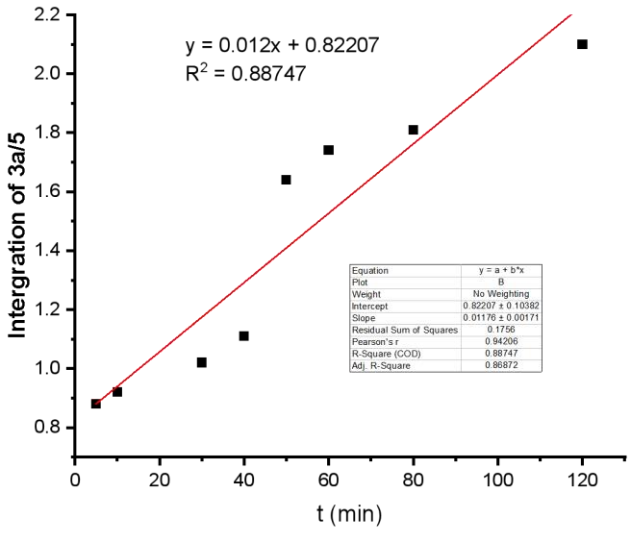

Figure S6. A plot of integration of product **3a** relative to intermediate **5**.

5.2 Synthesis of intermediate 5

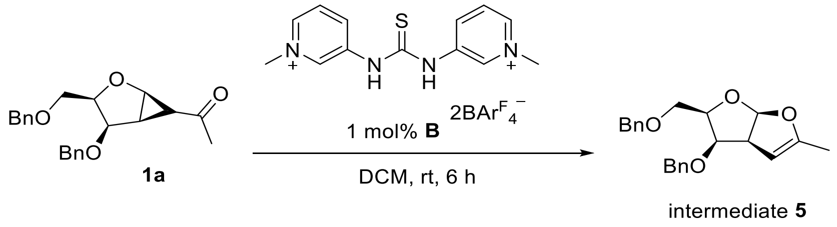

Intermediate 5 was synthesized according to reported literature.[5]

Procedure: A mixture of sugar **1a** (0.18 mmol, 1.0 eq.) and cat. **B** (1 mol%) were dissolved in 1 mL CH_2Cl_2, the resulting reaction mixture was stirred at room temperature for 6 h, and the solvent was removed under reduced pressure and the residue was subjected to flash column chromatography (dry loading) to give the intermediate **5** (29 mg, 46% yield)^{1}H NMR (CD_2Cl_2, 500 MHz) δ (ppm) 7.30-7.14 (m, 10H), 5.83 (d, *J* = 6.4 Hz, 1H), 4.52-4.40 (m, 5H), 4.30-4.21 (m, 2H), 3.56 (dd, *J* = 11.0, 3.1 Hz, 1H), 3.54-3.49 (m, 1H), 3.42 (dd, *J* = 10.9, 8.1 Hz, 1H), 1.63 (s,, 3H).The data is in accordance to literature.[5]

5.3 NMR monitoring for the downstream reaction of intermediate 5

5.3.1 Reaction of intermediate 5 and 2a in the presence of ChB catalyst J

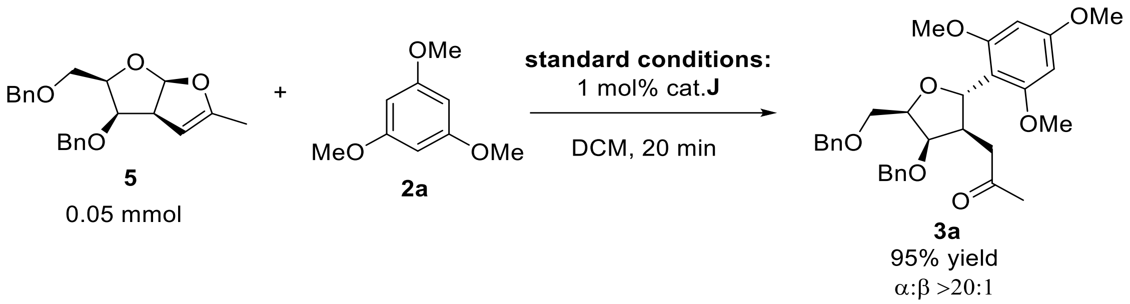

Procedure: A mixture of intermediate **5** (0.05 mmol, 1.0 eq.), 1,3,5-trimethoxybenzene **2a** (0.075 mmol, 1.5 eq.) and cat.**J** (1 mol%) were dissolved in 1 mL CH_2Cl_2, the resulting reaction mixture was stirred at room temperature for 20 min, the reaction mixture was filtered over a short silica plug and flushed with of dichloromethane. The filtrate was then evaporated and the reaction mixture was analyzed by crude ^{1}H NMR with 1,1,2,2-tetrachloroethane as the internal standard.

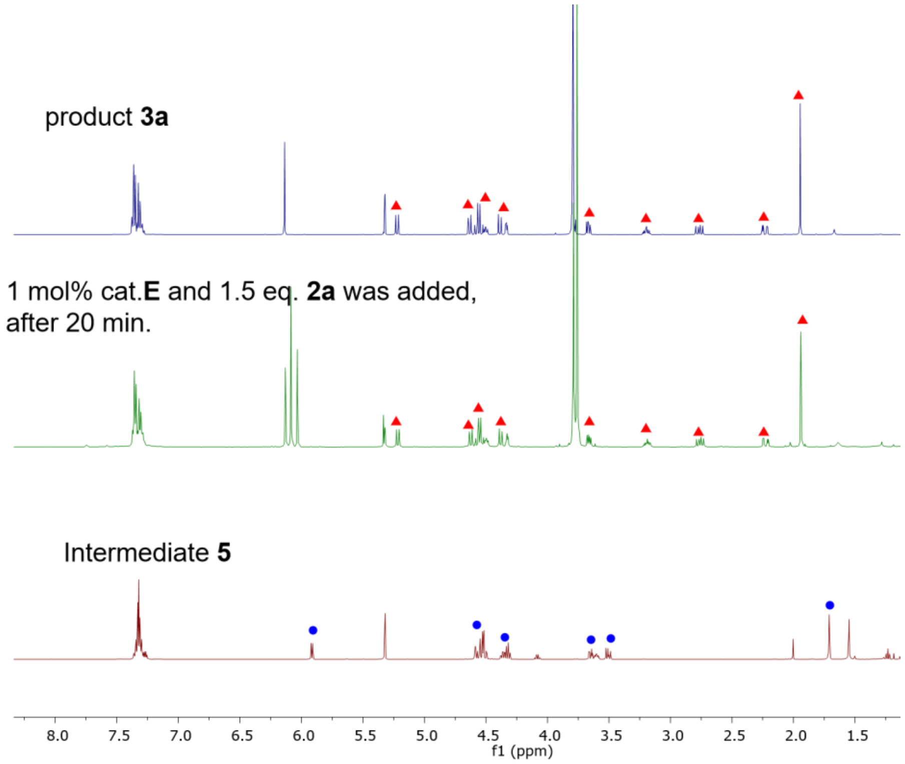

Figure S7. Downstream reaction with **2a** and ChB cat.**J**. Blue circles denotes the signals of the intermediate **5** and red triangles denotes the signals of the product **3a**.

5.3.2 Reaction of intermediate 5 and 2e in the presence of ChB catalyst H

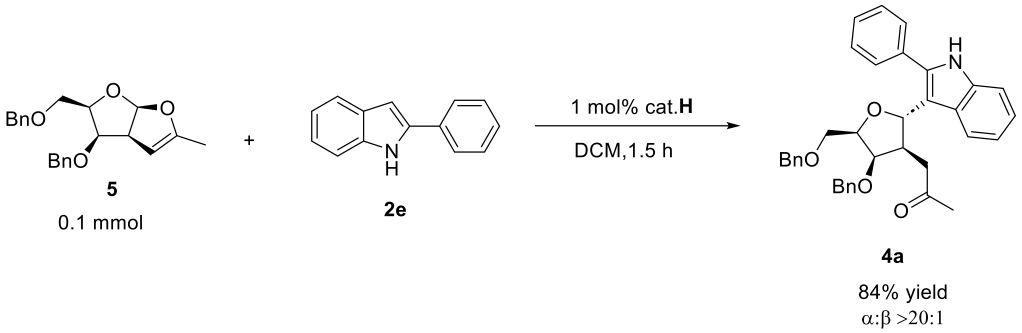

Procedure: A mixture of intermediate **5** (0.05 mmol, 1.0 eq.), indole **2e** (0.075 mmol, 1.5 eq.) and cat.**H** (1 mol%) were dissolved in 1 mL CH_2Cl_2, the resulting reaction mixture was stirred at room temperature for 1.5 h, the reaction mixture was filtered over a short silica plug and flushed with of dichloromethane. The filtrate was then evaporated and the reaction mixture was analyzed by crude ^{1}H NMR with 1,1,2,2-tetrachloroethane as the internal standard.

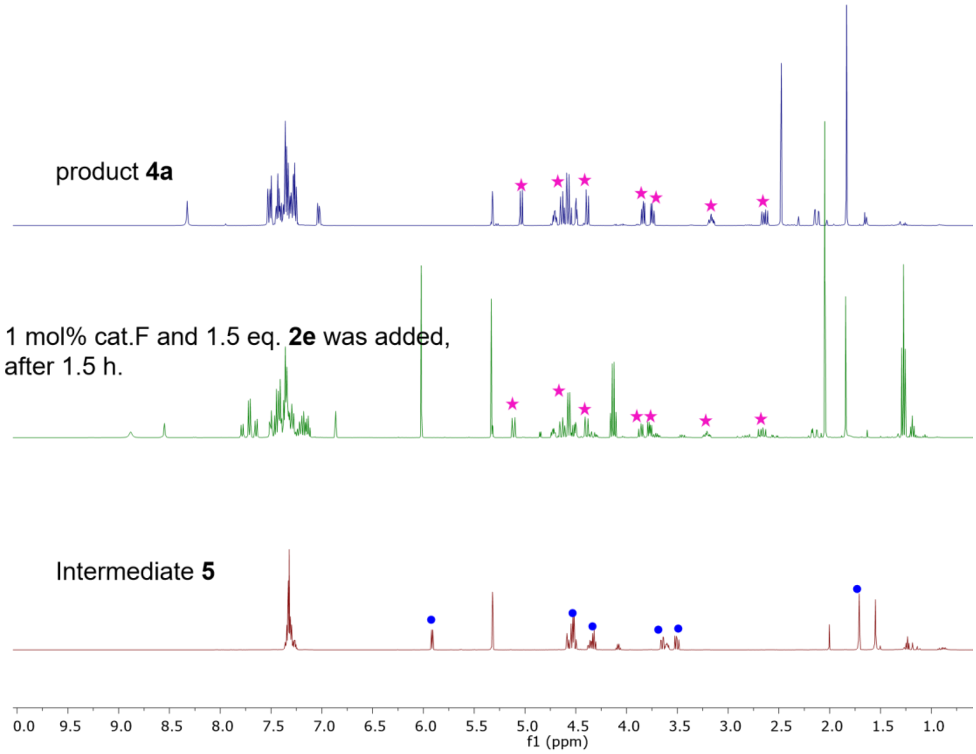

Figure S8. Downstream reaction with **2e** and cat. **H**. Blue circles denotes the signals of the intermediate **5** and pink stars denotes the signals of the product **4a**.

5.3.3 Reaction of intermediate 5 and 2a in the presence of XB catalyst C

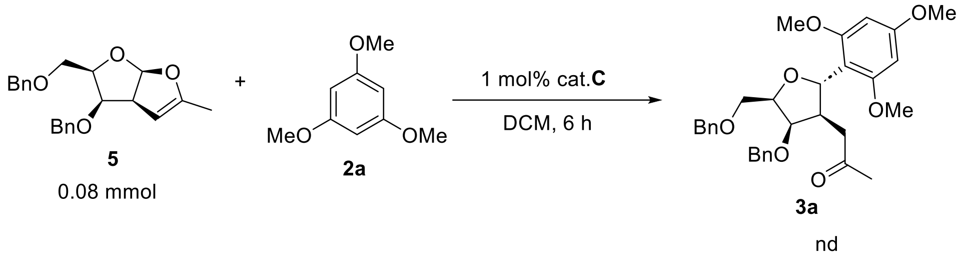

Procedure: A mixture of intermediate **5** (0.05 mmol, 1.0 eq.), 1,3,5-trimethoxybenzene **2a** (0.075 mmol, 1.5 eq.) and cat.**C** (1 mol%) were dissolved in 1 mL CH_2Cl_2, the resulting reaction mixture was stirred at room temperature for 6 h, the reaction mixture was filtered over a short silica plug and flushed with of dichloromethane. The filtrate was then evaporated and the reaction mixture was analyzed by crude ^1H NMR with 1,1,2,2-tetrachloroethane as the internal standard, no desired product **3a** was detected.

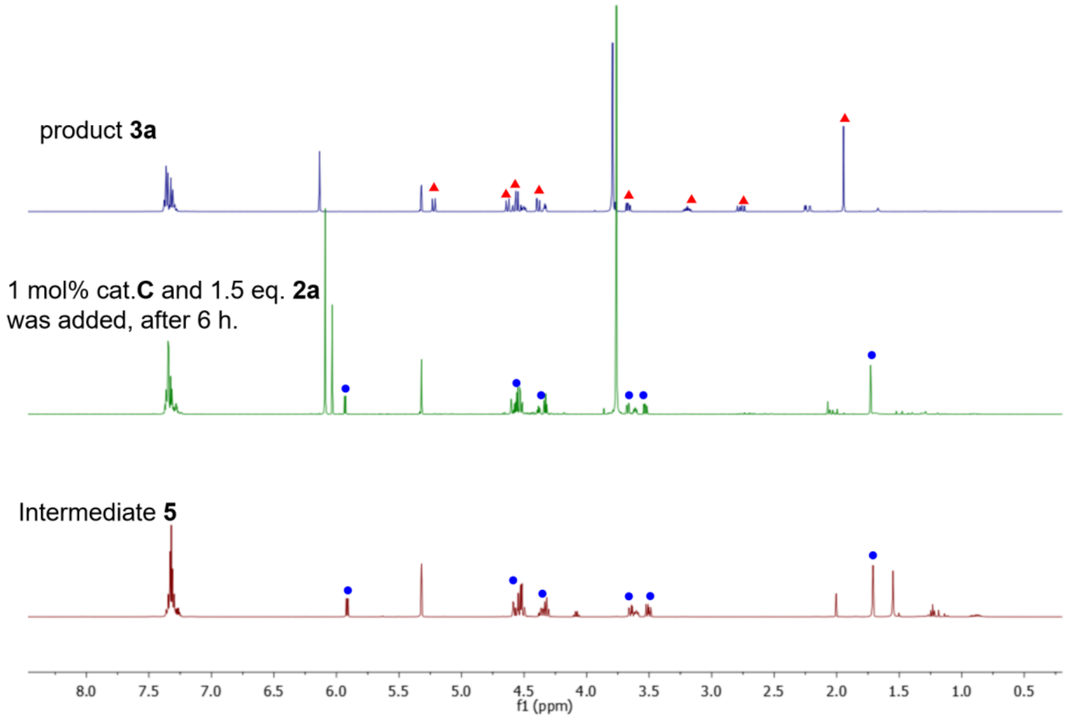

Figure S9. Downstream reaction with **2a** and XB cat.**C**. Blue circles denotes the signals of the intermediate **5** and red triangles denotes the signals of the product **3a.**

5.3.4 Reaction of intermediate 5 and 2e in the presence of XB catalyst C

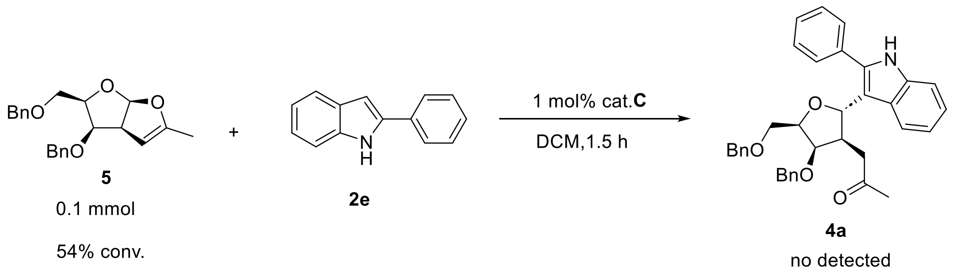

Procedure: A mixture of intermediate **5** (0.05 mmol, 1.0 eq.), indole **2e** (0.075 mmol, 1.5 eq.) and cat. **C** (1 mol%) were dissolved in 1 mL CH_2Cl_2, the resulting reaction mixture was stirred at room temperature for 1.5 h, the reaction mixture was filtered over a short silica plug and flushed with of dichloromethane. The filtrate was then evaporated and the reaction mixture was analyzed by crude ^{1}H NMR with 1,1,2,2-tetrachloroethane as the internal standard, conversion rate of 54%, and no desired product **4a** was detected.

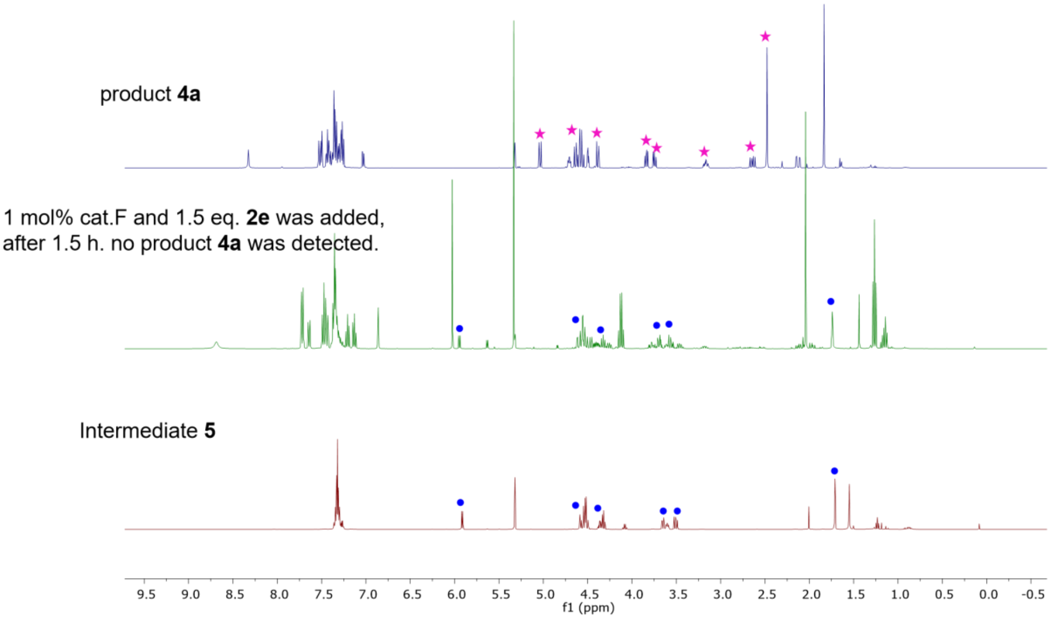

Figure S10. Downstream reaction with **2e** and cat.**C**. Blue circles denotes the signals of the intermediate **5** and pink stars denotes the signals of the product **4a**.

5.4 Poisoning experiments

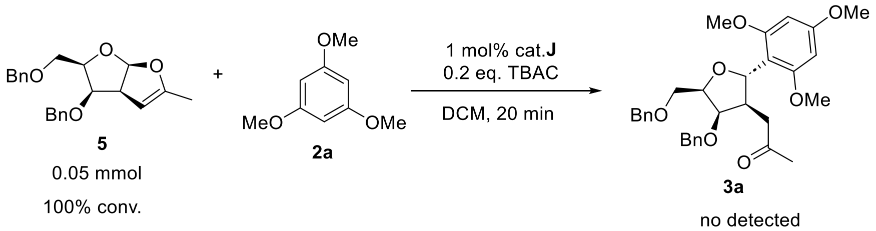

Procedure: A mixture of intermediate **5** (0.05 mmol, 1.0 eq.), 1,3,5-trimethoxybenzene **2a** (0.075 mmol, 1.5 eq.), TBAC (tetrabutylammonium chloride, 0.2 eq.) and cat.**J** (1 mol%) were dissolved in 0.2 mL CH_2Cl_2, the resulting reaction mixture was stirred at room temperature for 12 h, the reaction mixture was filtered over a short silica plug and flushed with of dichloromethane. The filtrate was then evaporated and the reaction mixture was analyzed by crude ^{1}H NMR with 1,1,2,2-tetrachloroethane as the internal standard, conversion rate of 100%, and no desired product **3a** was detected.

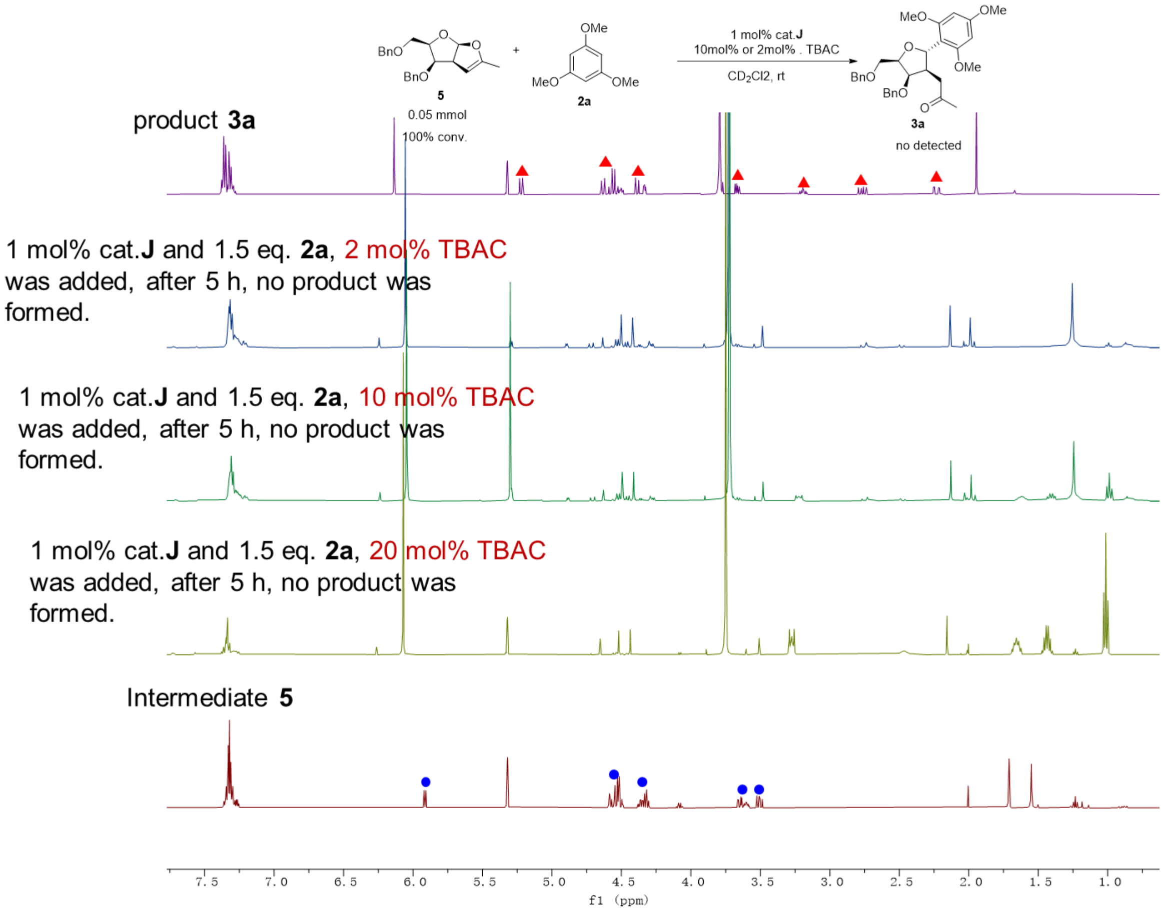

Figure S11. Poisoning experiment with **2a** and cat.**J**. Blue circles denotes the signals of the intermediate **5** and red triangles denotes the signals of the product **3a**.

5.5 NMR titration experiments

5.5.1 ^{77}Se NMR titration study of cyclopropyl ketone 6 and ChB catalyst J

^{77}Se NMR titration of Cat.**J** against cyclopropyl ketone **6** was studied in CD_2Cl_2. The solution of the Cat.**J** (10 mM) in CD_2Cl_2 was first prepared, then different equivalents of **6** was added into the NMR tube according to the ratio of Cat.**J** and **6**. The spectrum was recorded respectively. All ^{77}Se signals were referenced to the diphenyl diselenide (external standard) signal.

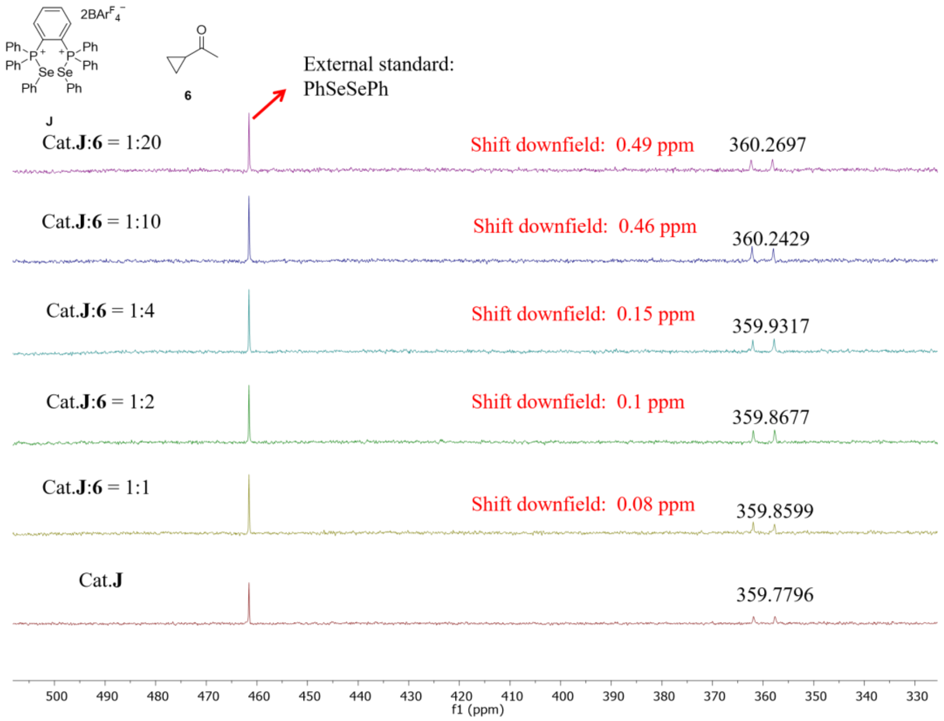

Figure S12. ^{77}Se NMR (298 K, CD_2Cl_2) of Cat.**J** (10 mM) upon addition of different amounts of **6**. Diphenyl diselenide as an external standard.

5.5.2 ^{1}H NMR and ^{13}C NMR titration study of cyclopropyl ketone 6 and ChB catalyst J

^{1}H NMR and ^{13}C NMR titration of Cat.**J** toward to D-glucal **6** was studied by ^{1}H NMR titrations in CD_2Cl_2 . The solutions of the Cat.**J** (10 mM) in CD_2Cl_2 was prepared, then different equivalents of **6** was added into the NMR tube according to the ratio of **J** and **6** (1:1, 1:2, 1:4, 1:10 and 1:20). The spectrum was recorded respectively.

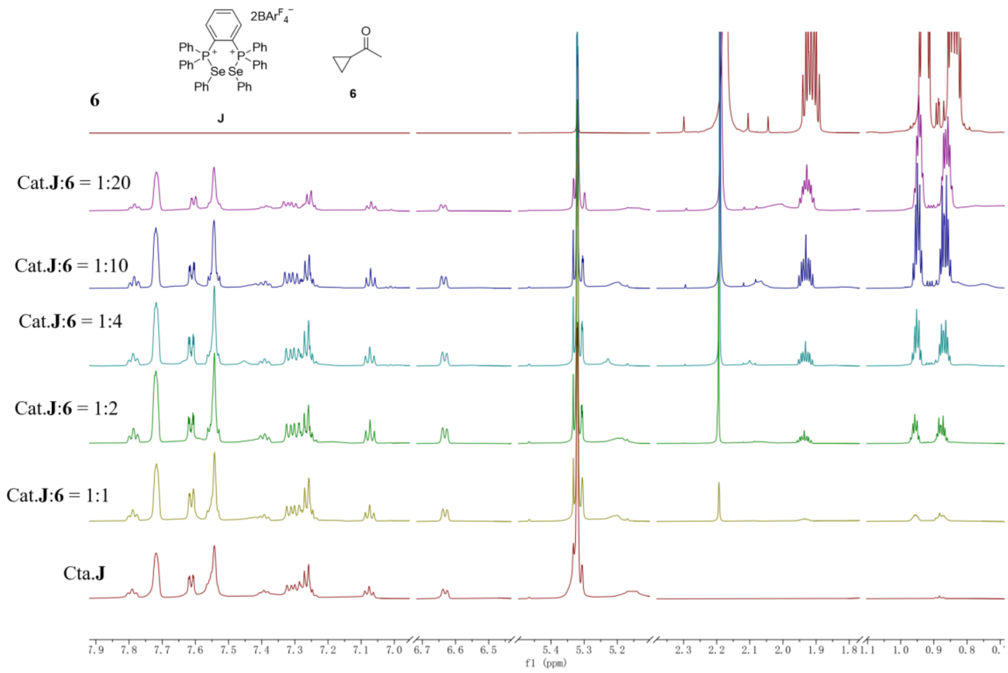

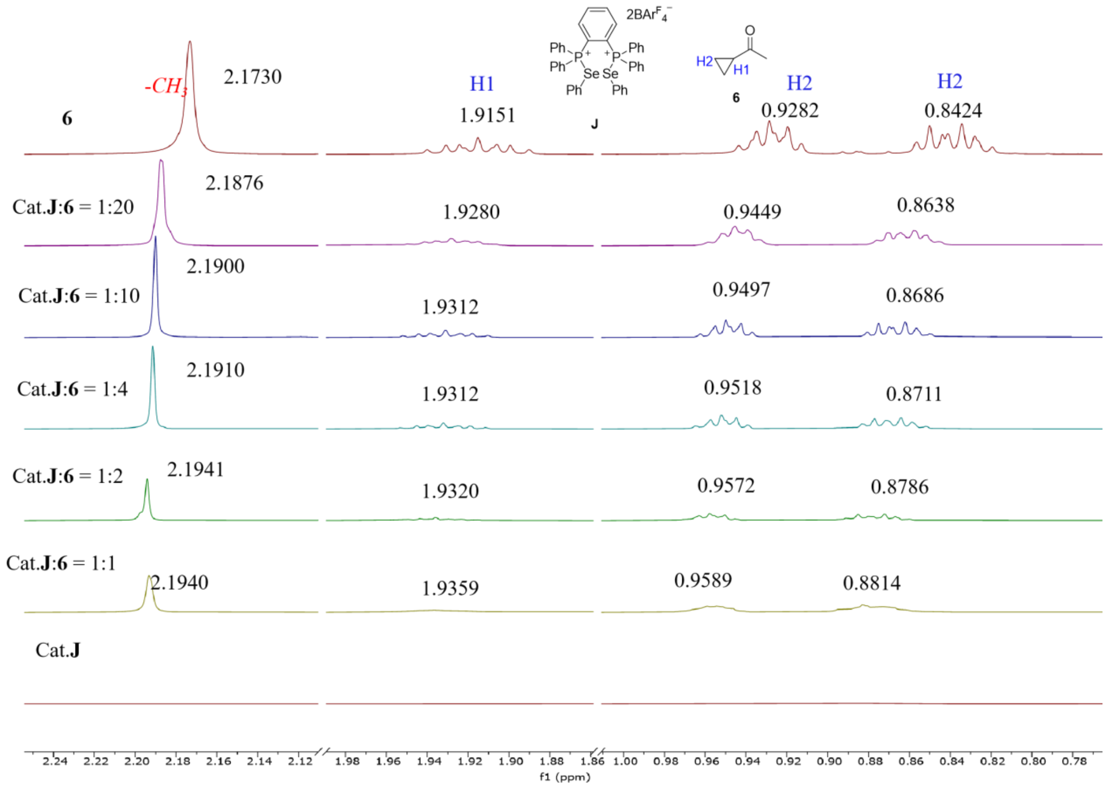

Figure S13. ^{1}H NMR (298 K, CD_2Cl_2) of Cat.**J** (10 mM) upon addition of different amounts of **6**.

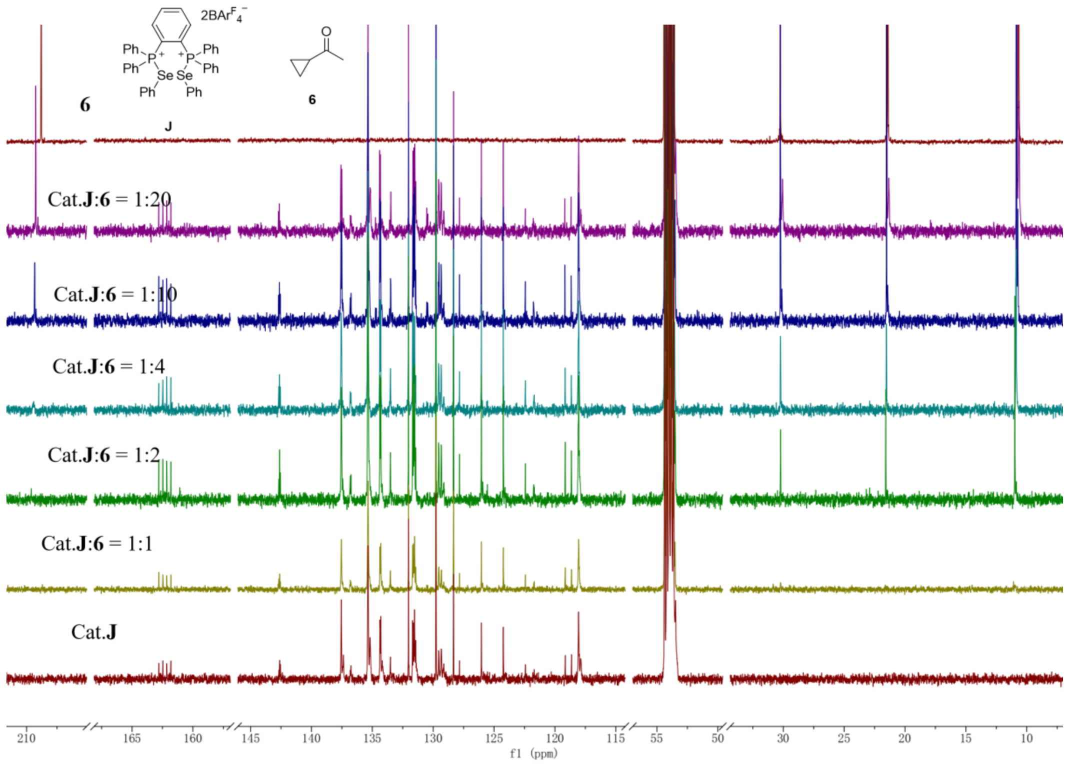

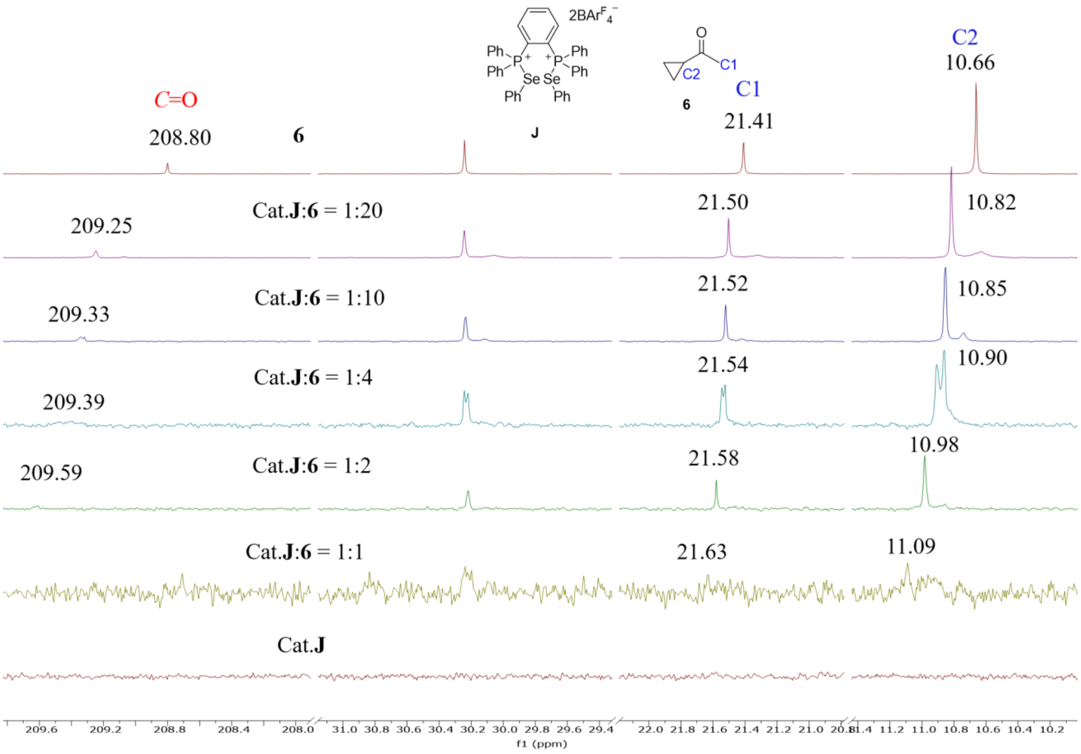

Figure S14. ^{13}C NMR (298 K, CD_2Cl_2) of Cat.**J** (10 mM) upon addition of different amounts of **6**.

5.5.3 ^{77}Se NMR titration study of **2a** and ChB catalyst J

^{77}Se NMR titration of Cat.**J** against **2a** was studied in CD_2Cl_2. The solution of the Cat.**J** (10 mM) in CD_2Cl_2 was first prepared, then different equivalents of **2a** was added into the NMR tube according to the ratio of Cat.**J** and **2a**. The spectrum was recorded respectively. All ^{77}Se signals were referenced to the diphenyl diselenide (external standard) signal.

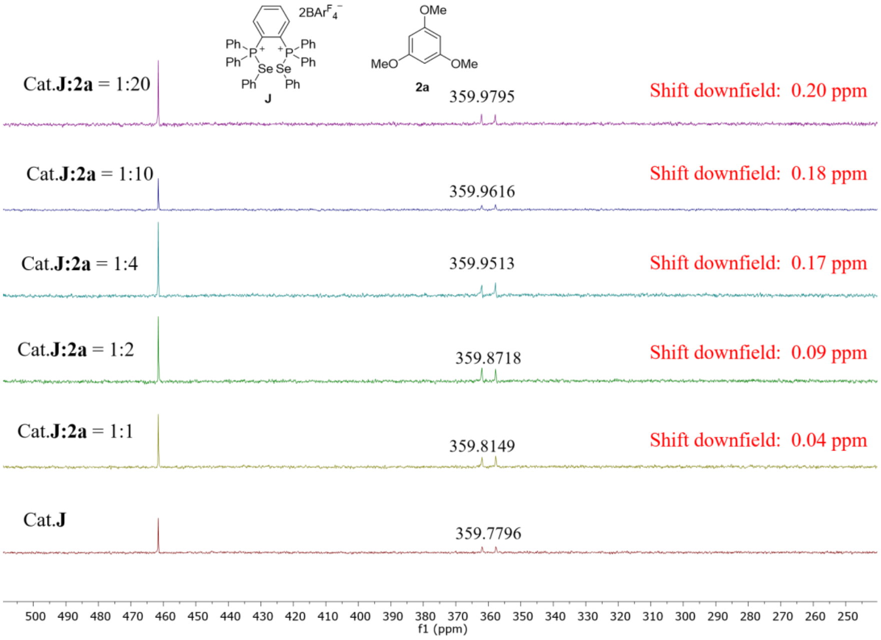

Figure S15. ^{77}Se NMR (298 K, CD_2Cl_2) of Cat.**J** (10 mM) upon addition of different amounts of **2a**. Diphenyl diselenide as an external standard.

5.5.4 ^{1}H NMR and ^{13}C NMR titration study of 2a and ChB catalyst J

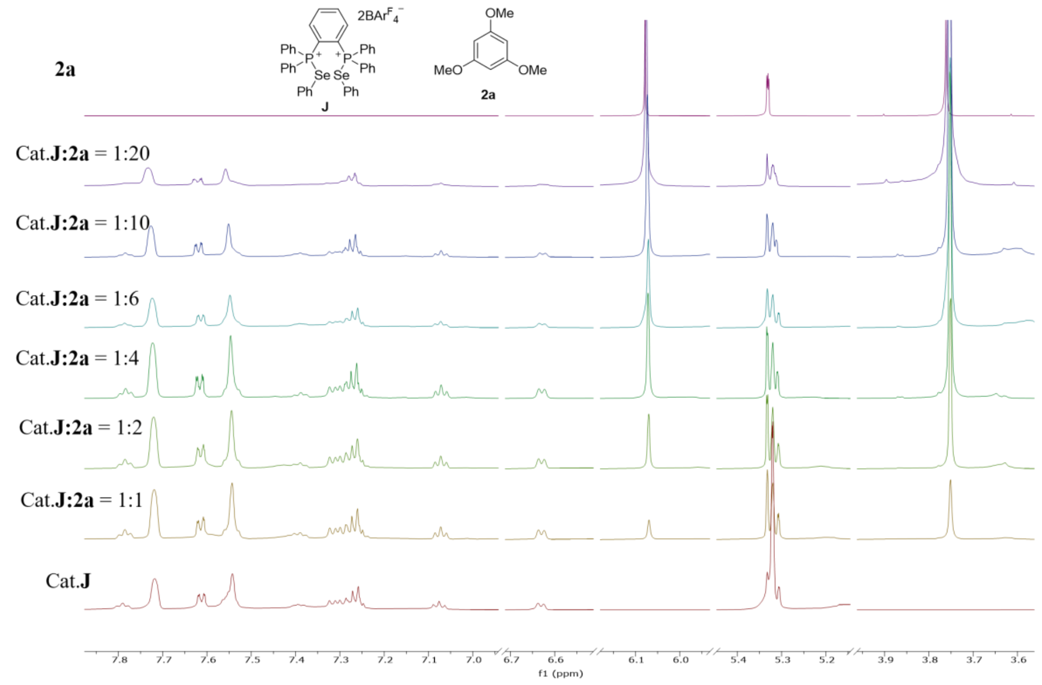

Figure S16. ^{1}H NMR (298 K, CD_2Cl_2) of Cat.**J** (10 mM) upon addition of different amounts of **2a**.

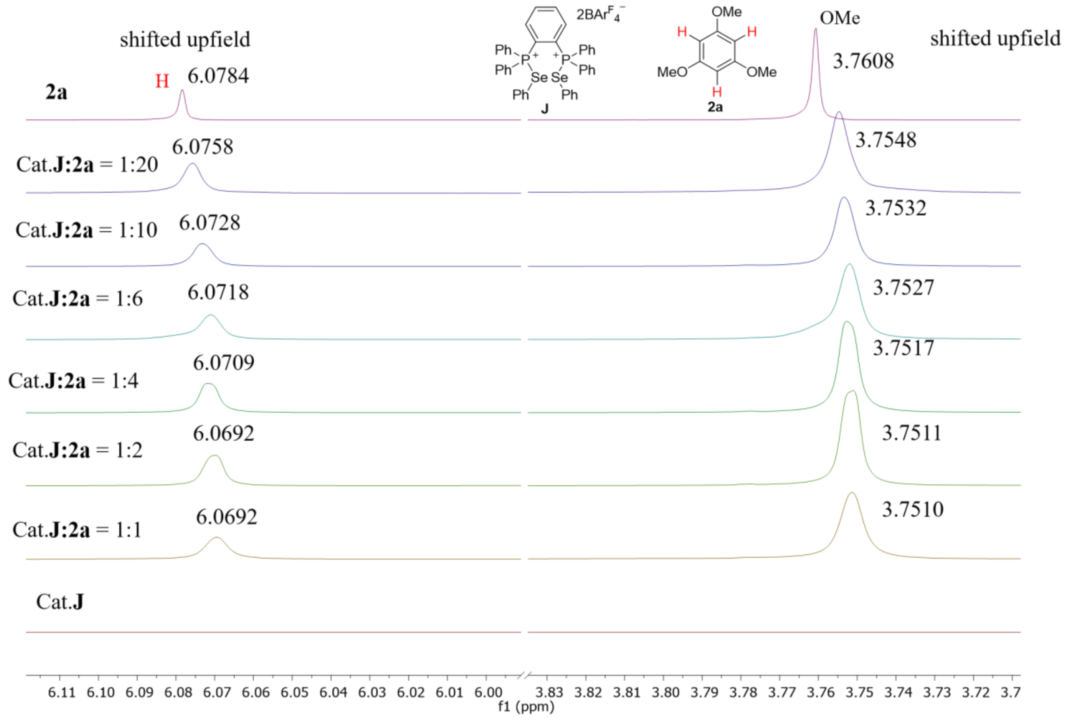

Figure S17. Zoom in region of **Figure S16**.

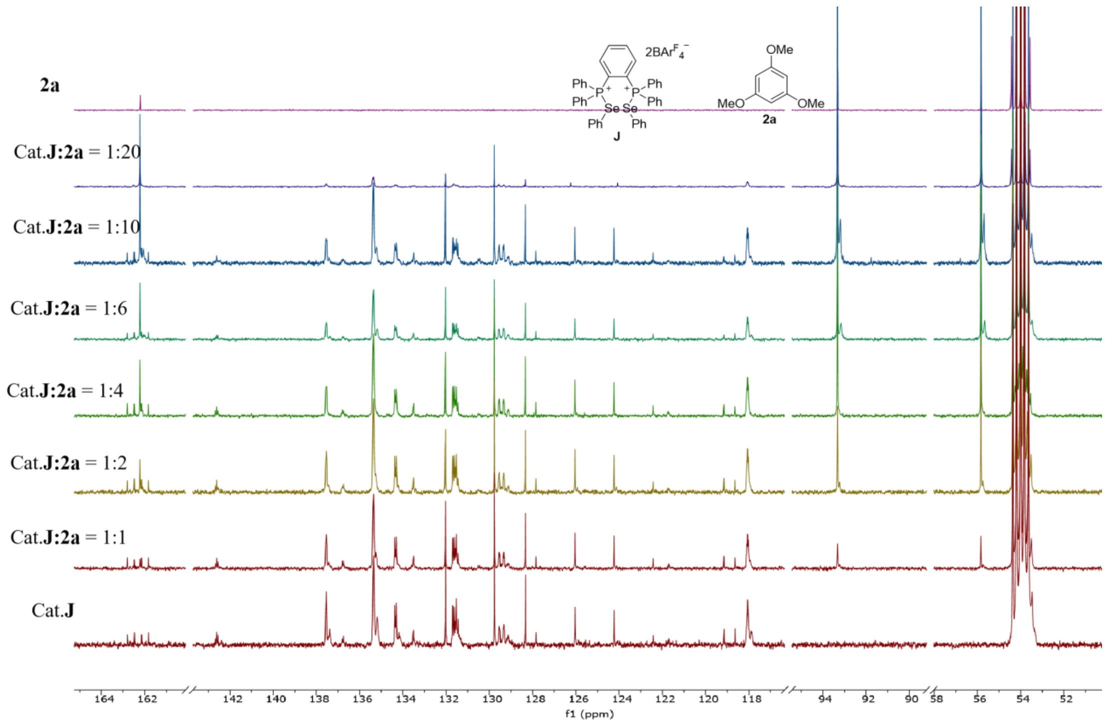

Figure S18. ^{13}C NMR (298 K, CD_2Cl_2) of Cat.**J** (10 mM) upon addition of different amounts of **2a**.

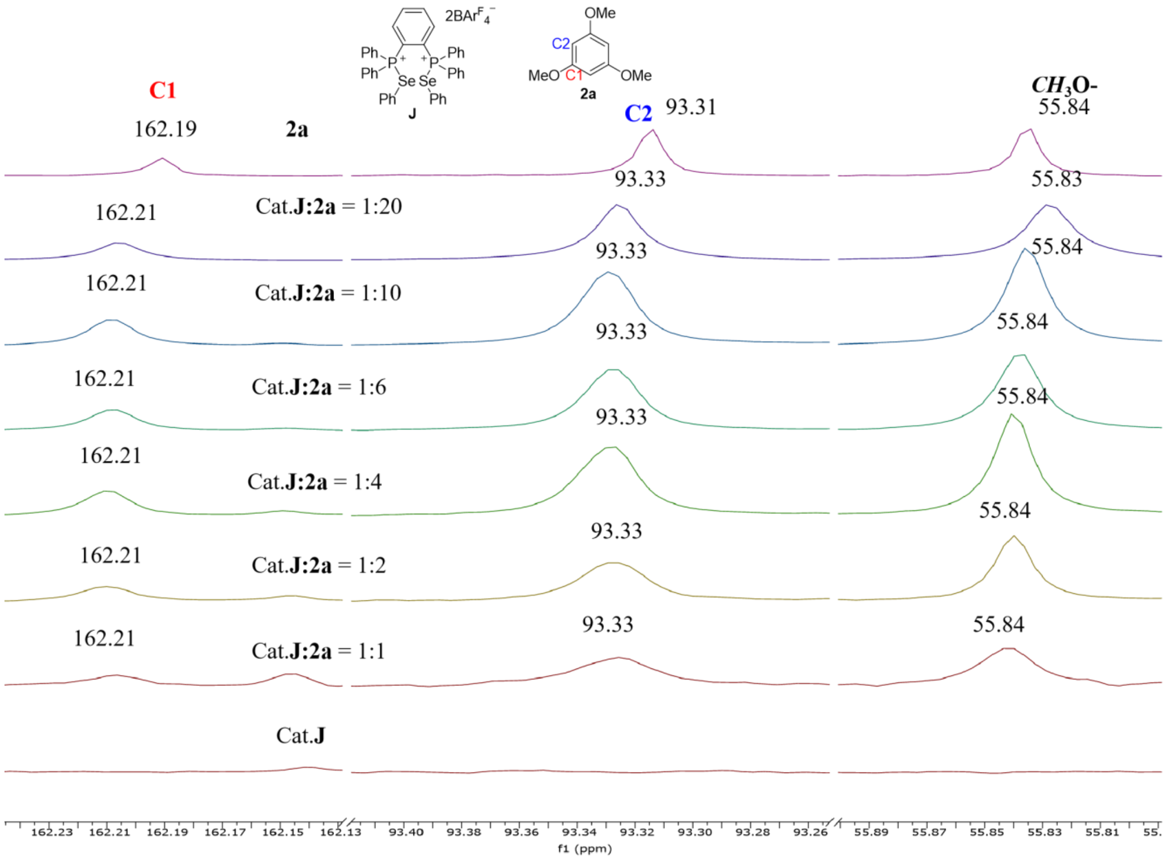

Figure S19. Zoom in region of **Figure S18**.

5.5.5 ^{1}H NMR and ^{13}C NMR titration study of cyclopropyl ketone 6 and XB catalyst C

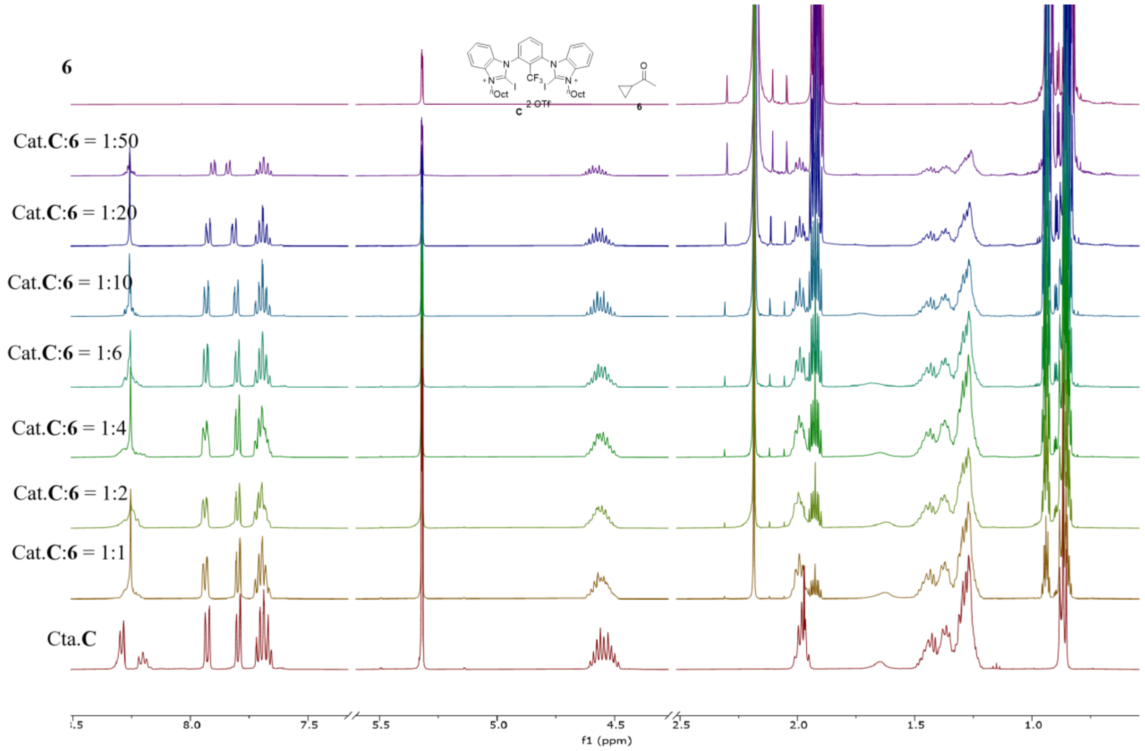

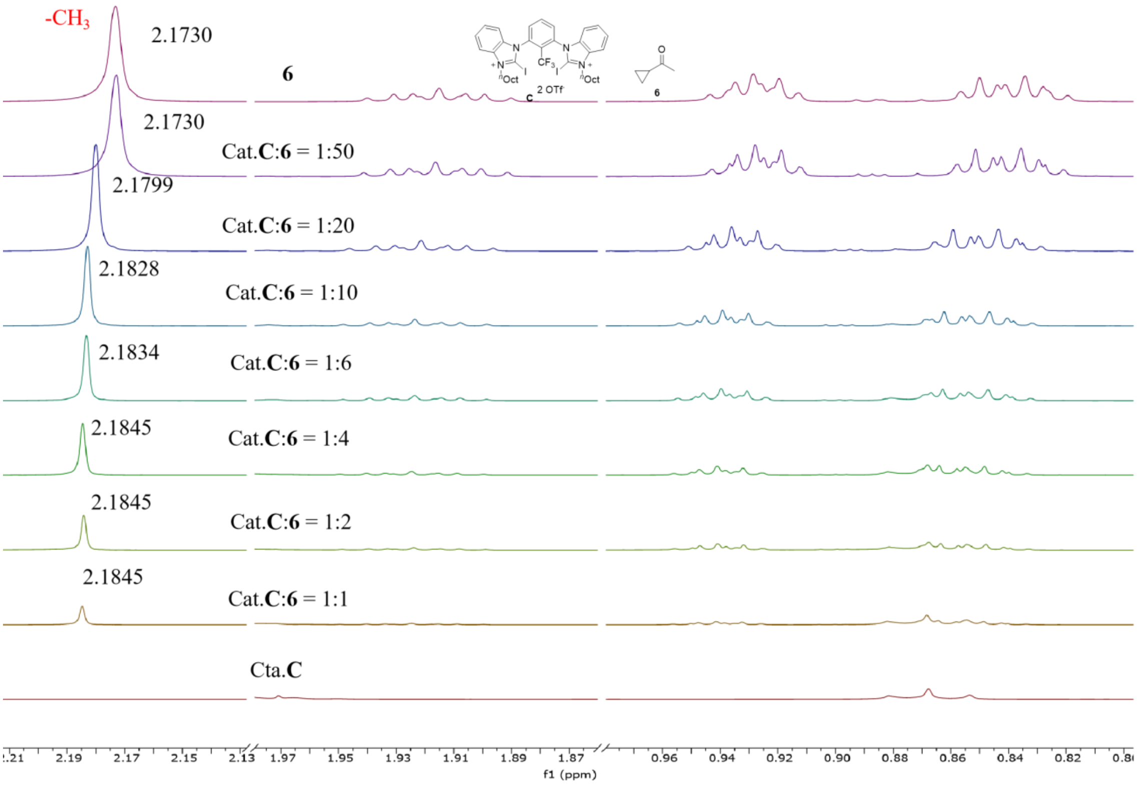

Figure S20. ^{1}H NMR (298 K, CD_2Cl_2) of Cat.**C** (10 mM) upon addition of different amounts of **6**.

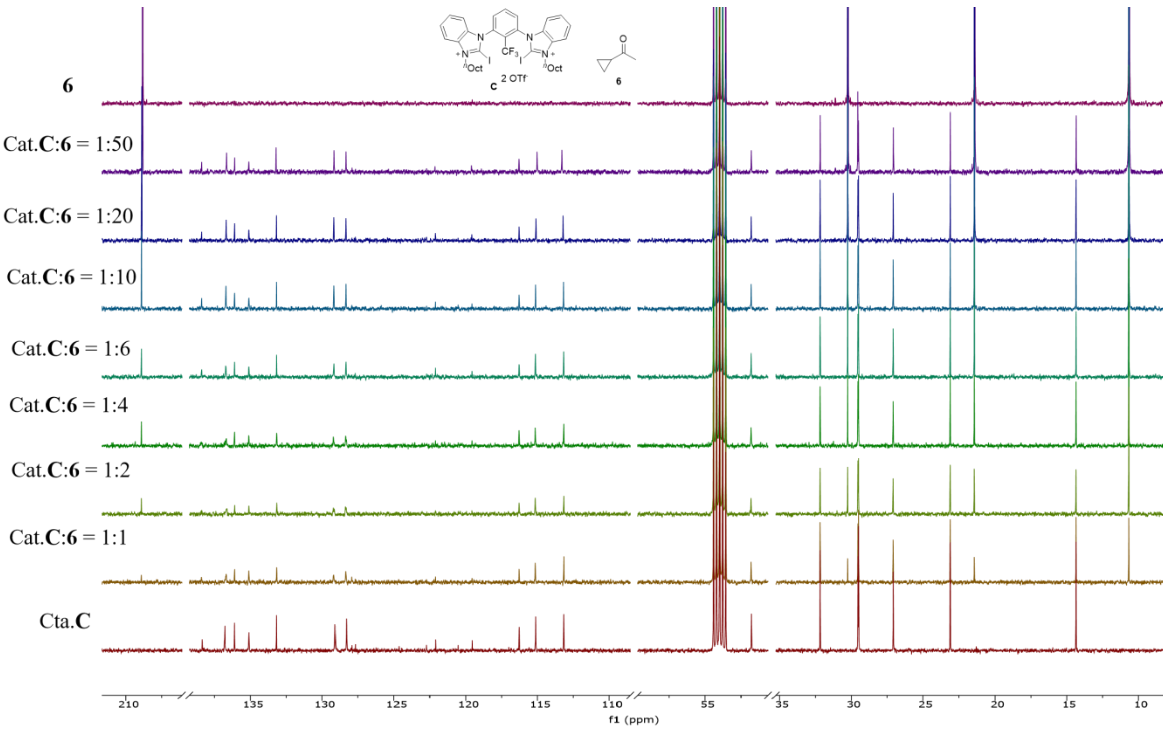

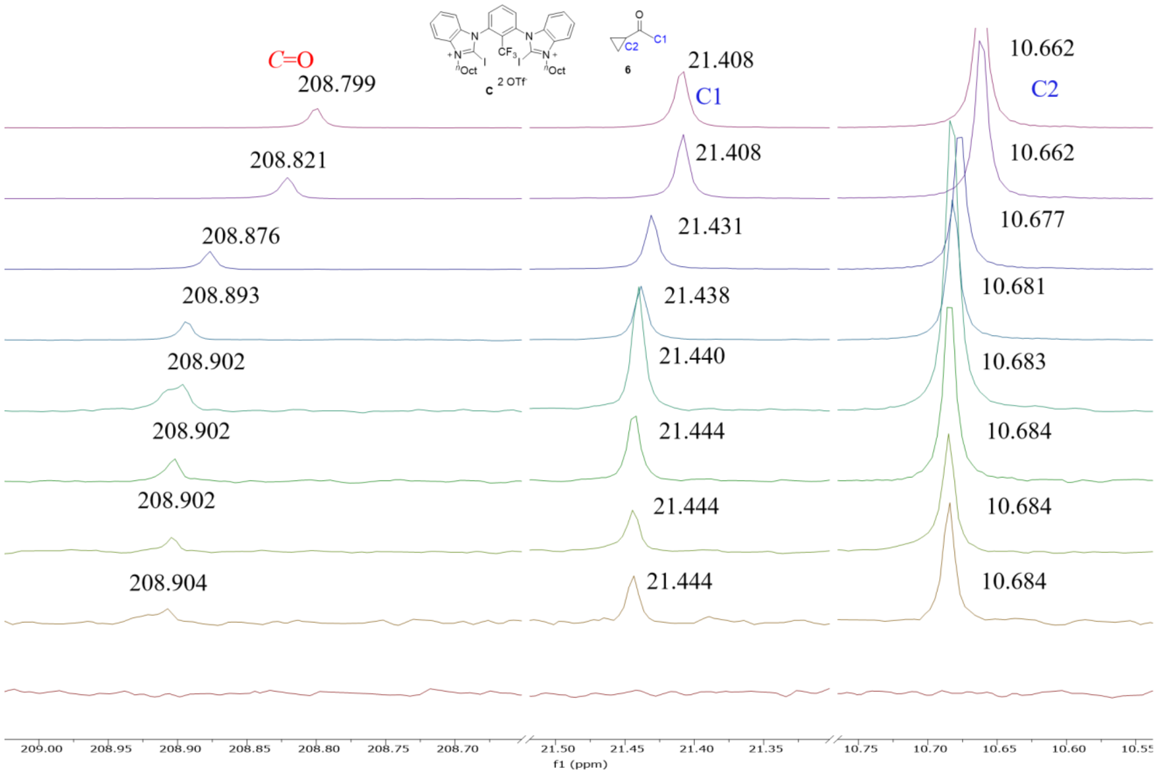

Figure S21. ^{13}C NMR (298 K, CD_2Cl_2) of Cat.**J** (10 mM) upon addition of different amounts of **6**.

5.5.6 ^{1}H NMR and ^{13}C NMR titration study of 2a and XB catalyst C

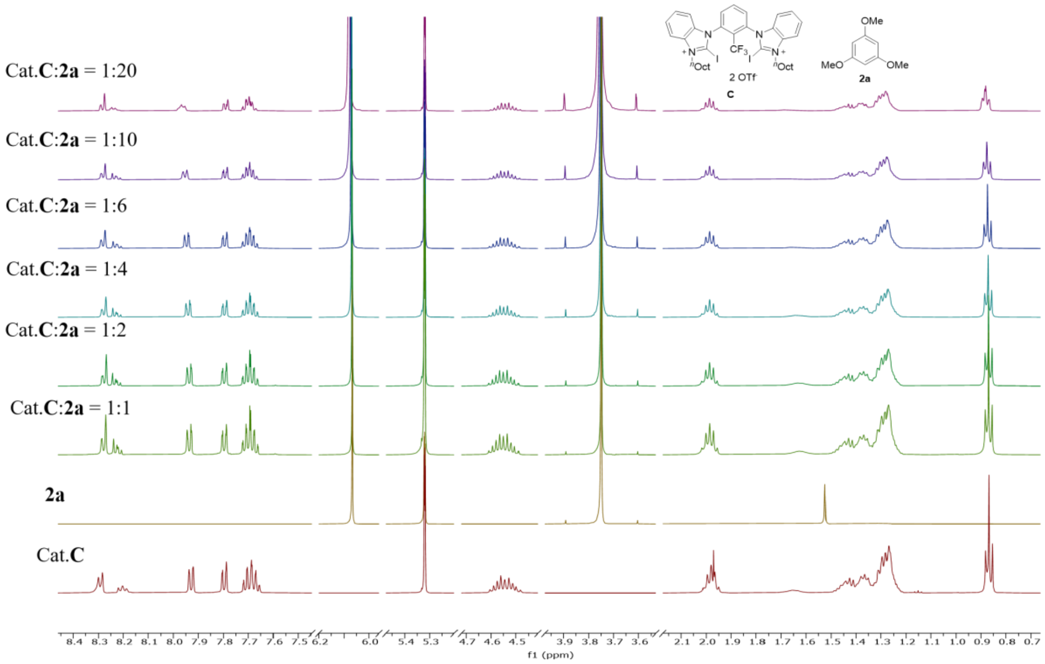

Figure S22. ^{1}H NMR (298 K, CD_2Cl_2) of Cat.**C** (10 mM) upon addition of different amounts of **2a**.

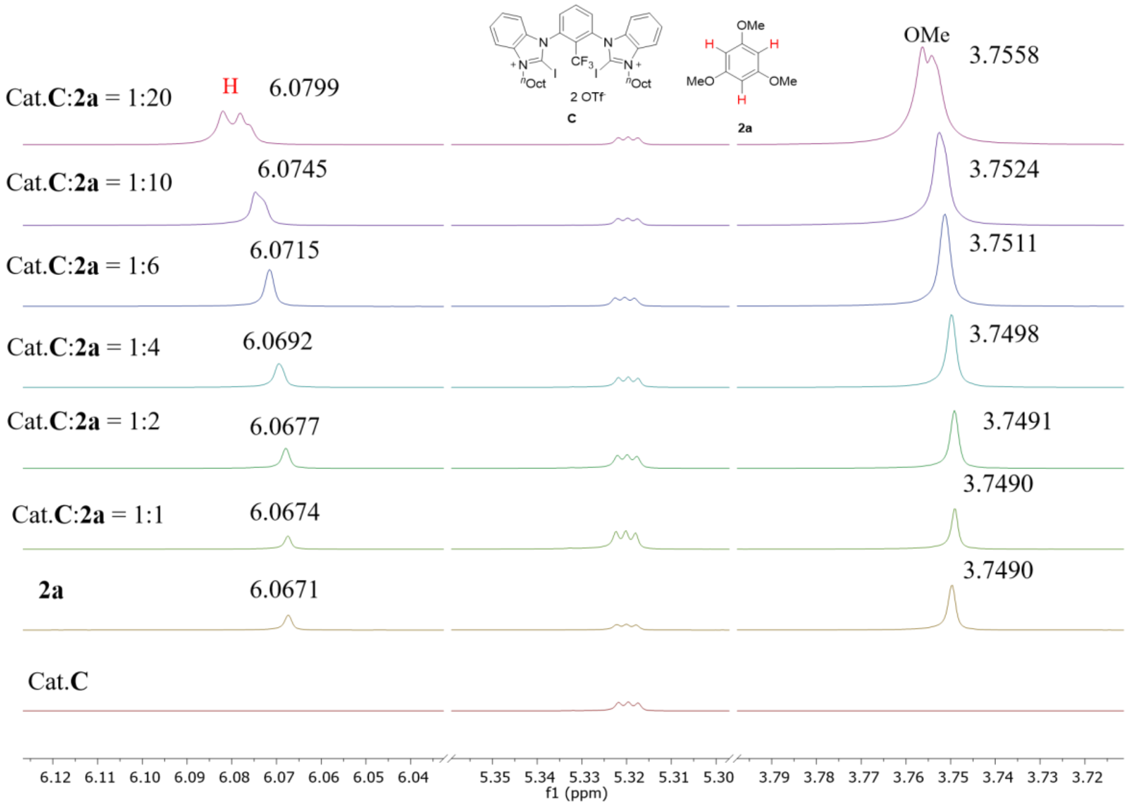

Figure S23. Zoom in region of **Figure S22**.

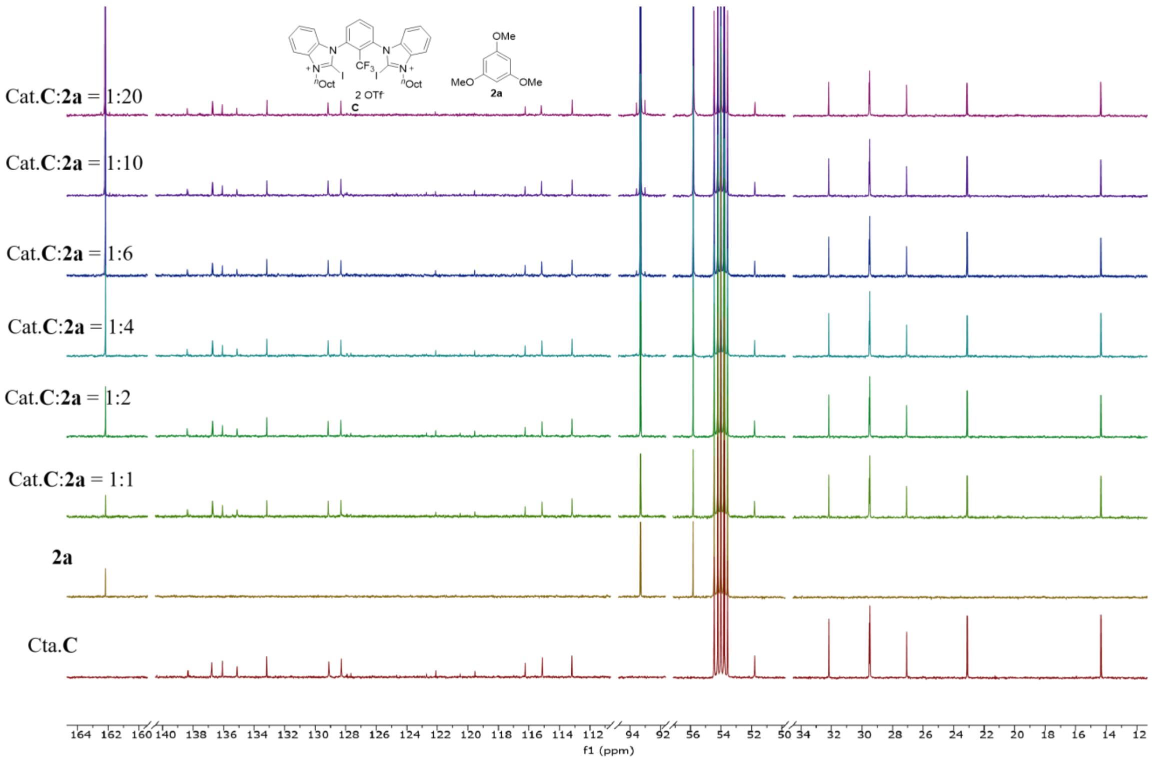

Figure S24. ^{13}C NMR (298 K, CD_2Cl_2) of Cat.**C** (10 mM) upon addition of different amounts of **2a**.

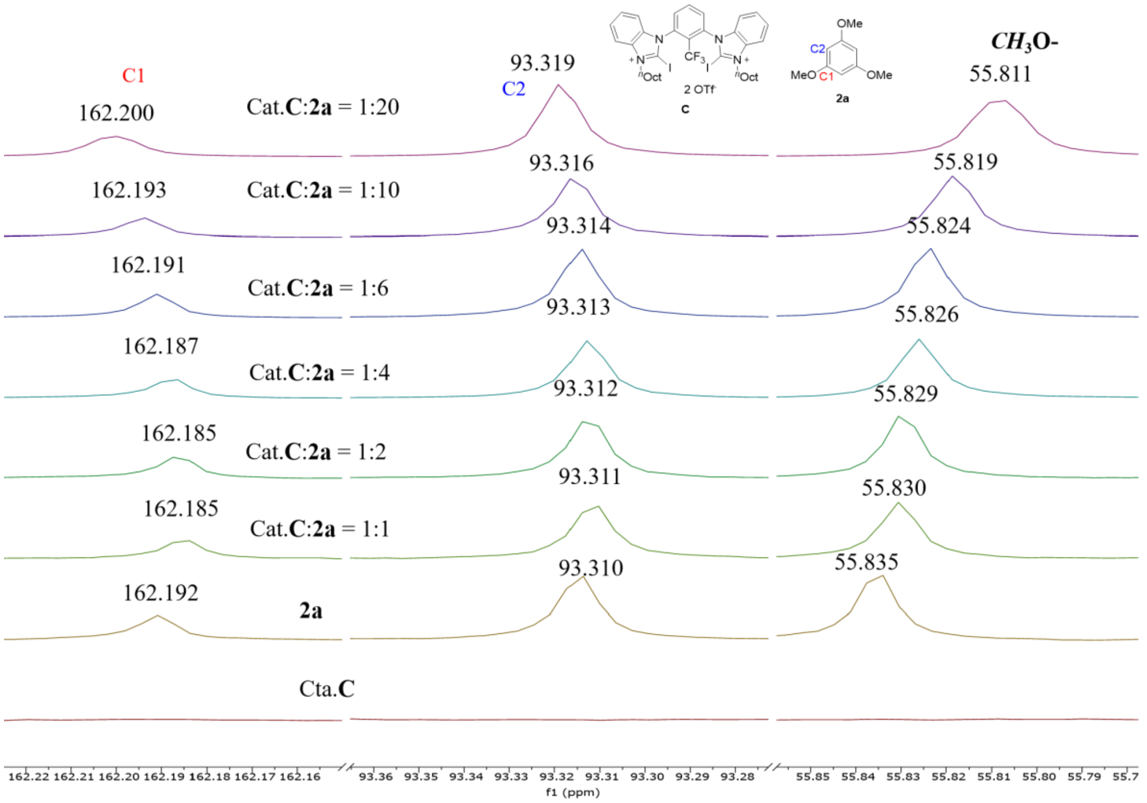

Figure S25. Zoom in region of **Figure S24**.

5.5.7 ^{1}H NMR titration study of intermediate 5 and ChB catalyst J

^{1}H NMR titration of Cat.**J** against **5** was studied in CD_2Cl_2. The solution of the Cat.**J** (10 mM) in CD_2Cl_2 was first prepared, then two equivalents of **5** was added into the NMR tube. The ^{1}H NMR spectrum was then recorded. The result indicated that intermediate **5** decomposed in the presence of cat.**J**.

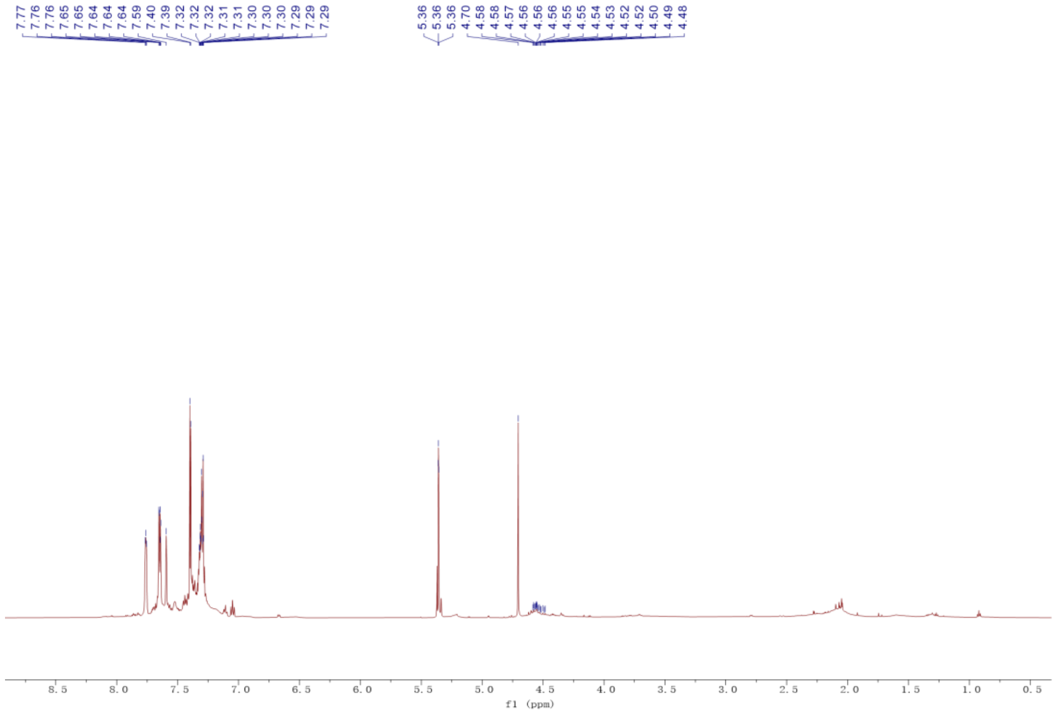

Figure S26. ^{1}H NMR (298 K, CD_2Cl_2) of mixture of Cat.**J** (10 mM) and intermediate **5**.

6. Hammett analysis

Hammett analysis was conducted according to an established procedure in the literature.[11]

General experimental procedure for ChB catalysis: A mixture of sugar **1a** (0.1 mmol, 1.0 eq.), indole **2e-2i** (0.075 mmol, 1.5 eq.) and cat.**H** (1 mol%) were dissolved in 1 mL CH_2Cl_2, the resulting reaction mixture was stirred at room temperature for 20 min, the reaction mixture was filtered over a short silica plug and flushed with of dichloromethane. The filtrate was then evaporated and the reaction mixture was analyzed by crude ^{1}H NMR with 1,1,2,2-tetrachloroethane as the internal standard.

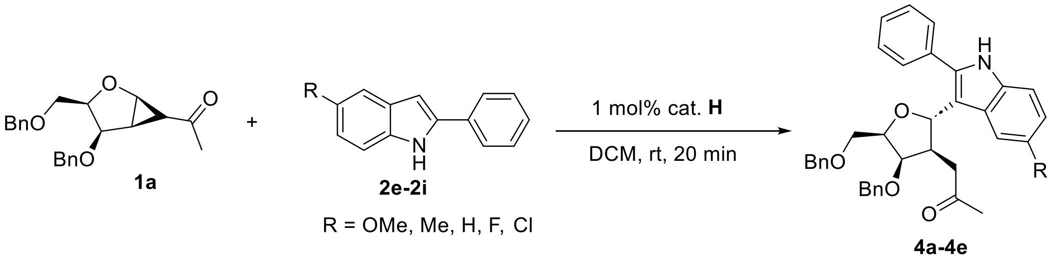

Entry	R	Product **4**	σ_p	k_X/k_H	log (k_X/k_H)
1	OMe	**4b**	-0.268	1.60	0.204119983
2	Me	**4c**	-0.170	1.35	0.130333768
3	H	**4a**	0	0	0

4	F	**4d**	+0.060	0.43	-0.366531544
5	Cl	**4e**	+0.227	0.30	-0.522878745

General experimental procedure for XB catalysis: A mixture of sugar **1a** (0.1 mmol, 1.0 eq.), indole **2e-2i** (0.075 mmol, 1.5 eq.) and cat.**C** (1 mol%) were dissolved in 1 mL CH_2Cl_2, the resulting reaction mixture was stirred at room temperature for 2 h, the reaction mixture was filtered over a short silica plug and flushed with of dichloromethane. The filtrate was then evaporated and the reaction mixture was analyzed by crude ^{1}H NMR with 1,1,2,2-tetrachloroethane as the internal standard.

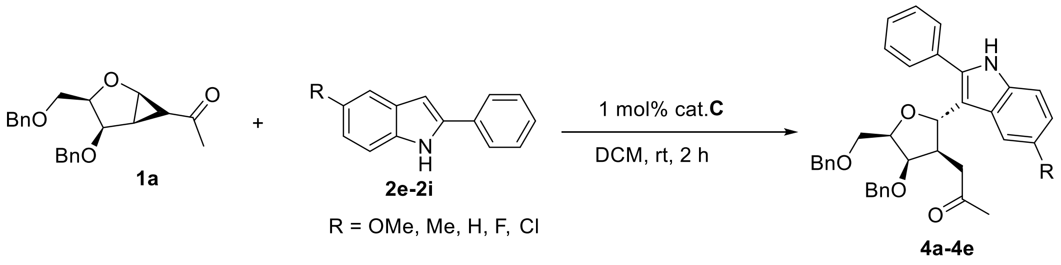

Entry	R	Product **4**	σ_p	k_X/k_H	log (k_X/k_H)
1	OMe	**4b**	-0.268	1.16	0.064458
2	Me	**4c**	-0.170	1.05	0.021189
3	H	**4a**	0	0	0
4	F	**4d**	+0.060	0.86	-0.065502
5	Cl	**4e**	+0.227	0.62	-0.207608

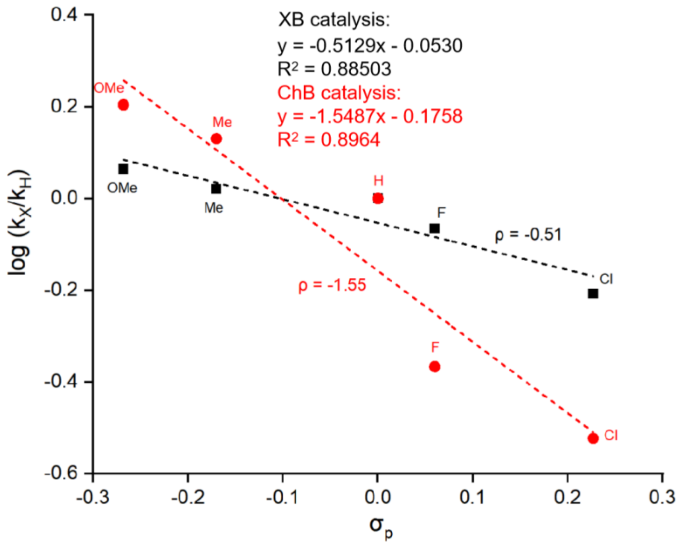

Figure S27. A plot of logarithms of the calculated rate constant vs the corresponding σ values of the para- substituents.

7. Anomerization stability of product 3h in the presence of catalyst J.

To study the anomerization stability of the β-*C*-glycoside **3h** in the presence of cat. **J**, product **3h** (0.05 mmol) and 1mol% catalyst **J** were dissolved in 0.5 mL CD_2Cl_2 in a dry NMR tube, then the solution was monitored by ^1H NMR at room temperature at the time points of 10 min, 4h, 15h, 24h. The results indicated that no anomerization of the β-anomer was observed in the presence of catalyst **J**, evidencing the stability of the β-*C*-glycosidic linkage.

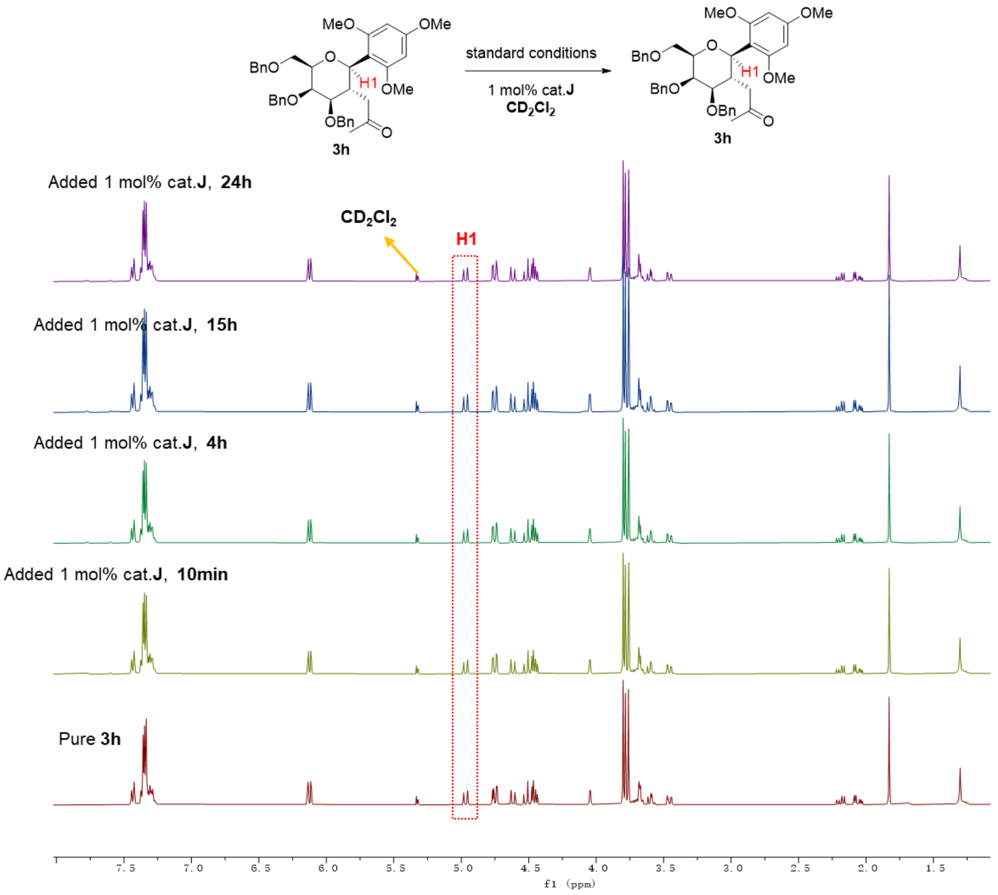

Figure S28. Evaluation of anomerization stability of product **3h** by ^1H NMR at room temperature in the presence of catalyst **J**.

8. Computational Details

All quantum chemical calculations were performed with the developmental version of the Orca 6.0.1 or Orca 6.1.0 program package,[12-13] and its interface to the open sourced xTB code.[14] All molecular geometries were preliminarily optimized using the Grimme's semi-empirical GFN2-xTB hamilitonian[14] with the analytical linearized Poisson-Boltzmann (ALPB) implicit solvation model for CH_2Cl_2.[15] The robust GOAT (Global Optimization Algorithm)[16] workflow developed by de Souza et al which was implemented in Orca was subsequently used at the same level of theory to search for the ensemble of energetically lowest lying conformers for the downstream ternary complexes **9** and **11** between the catalyst, intermediate and glycosyl acceptor. A similar conformational search protocol was also conducted for binary complexes **7** and **5-complex**.

After scrutinizing multiple plausible conformers, the energetically lowest conformer (global minima) from GOAT was assessed to be geometrically representative, which was then further optimized (utilizing TIGHTSCF settings) at a higher level using the meta-hybrid M06-2X functional[17] developed by Trular et al. with tight integration grids (DEFGRID3 setting in Orca), corrected using the D3(0) dispersion model (the zero-damping variant recovers the long-range contribution, which is deficient in M06-2X),[18-

[19] and using the def2-TZVP basis set.[20] The M06-2X functional had been demonstrated in benchmark studies[21-22] to be well suited in describing chalcogen bonding, halogen bonding and non-covalent interactions in general.[23]

The default Gaussian charge scheme with a scaled vdW-type cavity of the conductor-like polarizable continuum (CPCM) solvation model for CH_2Cl_2 as implemented in Orca was applied.[24-25] The resolution-of-identity approximation[26] for Coulomb integrals and COSX numerical integration for HF exchange (RIJCOSX) was applied along with the appropriate corresponding auxiliary basis sets.[27-28] Harmonic frequencies calculations at 298.15 K were computed at the same level of theory and used to verify the nature of the optimized stationary point as minima (no imaginary frequencies, threshold set at $i\omega < 10$ cm^{-1}) based on the modified ideal gas-rigid rotor-harmonic oscillator (RRHO) model.[29] Spectator counteranions were excluded in the computations. The Gibb's free energies of complexes **7** and **5-complex** were also obtained at the M06-2X-D3(0)/def2-TZVP/CPCM(CH_2Cl_2) level of theory.

The independent gradient model based on the Hirshfeld partition of molecular density (IGMH analysis)[30] by Lu et al. was performed to reveal the inter-fragment noncovalent isosurfaces, colored by the mapped function sign(λ_2)ρ, using algorithms implemented by Lu et al. in Multiwfn 3.8.[31]

IGMH isosurfaces were then rendered using VMD 1.9.4a51.

CYLView[32] rendering of ternary complexes 9 and 11

The cartesian coordinates (in Å) of the optimized stationary points (minima) at the M06-2X-D3(0)/def2-TZVP/CPCM(CH_2Cl_2) level of theory are provided. The geometries were rendered using CYLView20. (Red atoms = oxygen, grey atoms = carbon, yellow atoms = phosphorus, purple atoms = selenium, brown atoms = iodine, fluorine atoms = green, white atoms = hydrogen). The relevant NCIs are displayed using dotted lines, and the relevant distances (in in Å) are shown alongside.

Downstream encounter complex 9

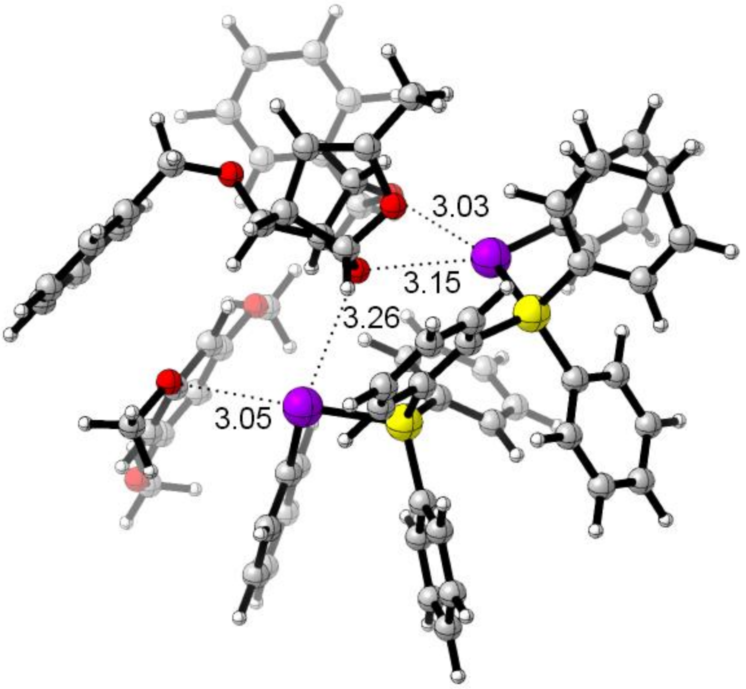

154

H	4.58079208083909	-4.12953318928367	3.66488282104434
H	0.38905613389817	-1.60899865646036	-2.89604063314567
H	5.95064289132838	-6.24077508544215	0.08913784342287
C	2.90558851167443	-2.21944202984990	0.57801056993582
C	2.46113601783534	-2.71210099980444	-0.64548970706011

C	2.90231242217891	-3.93561924985123	-1.12632154576869
C	3.77224059632058	-2.98270598996178	1.34092019758562
C	5.00871925091638	-3.16731976091685	3.37484267416638
C	4.22853381834556	-4.22717676250572	0.89667193753737
C	4.98603448573050	-6.71902796760897	-0.09301052875264
C	3.78106741114353	-4.68337086999101	-0.34016606382766
C	1.07629314484549	-2.37640692225970	-2.54978794008931
O	4.13995703105134	-5.88036844201306	-0.86168764479622
O	1.55041051153093	-1.93844796405593	-1.28675989979840
O	4.11926457844722	-2.44833792132067	2.53801896344344
H	2.57423977971466	-1.25041800091876	0.92639475363360
H	5.96937370547537	-3.32649177026753	2.88096618962152
H	0.55085819875676	-3.33049179579079	-2.45577838338184
H	5.13140879106770	-7.62058256886811	-0.68113831453455
H	2.56758413715019	-4.34945995467050	-2.06580252514579
H	4.89968662611011	-4.81744363256160	1.49839482757924
H	4.51807486907442	-6.97386033803844	0.86069474343318
H	1.89993675931045	-2.47732567473885	-3.25924143669617
H	5.15106995186303	-2.54889903609463	4.25799202330330
H	5.25131477492720	-0.96527129160124	-1.53891822255405
C	4.47323618103413	-0.25935747846964	-1.80257677098063
H	4.16782707139545	-1.08747912843663	-3.76110967076721
C	3.86259259929342	-0.32982493745155	-3.04994361623947
H	4.55967648859568	0.76924376861834	0.08087347348540
C	4.08423005411223	0.71260350006134	-0.89253260386201
H	-2.35990165310598	5.00053986032436	3.49447206129149
C	2.85759878918474	0.57005646858672	-3.37901404374268
C	3.07868101649668	1.62145445677333	-1.21600499201421
H	0.58469814983934	4.47835568271606	1.27392662075382
H	2.37393403847525	0.51479691453432	-4.34632380215069
H	1.86308840144460	3.29046553764385	-0.62771574090219
C	2.46494019452514	1.53920141399895	-2.46184579719625
C	-1.29242104632583	4.77318952486521	3.53053967315564
C	2.68593678944565	2.69053125320157	-0.22596117134728
H	-0.72397369972566	5.67738663179713	3.32629591534064
C	-0.07715064697672	3.67430351561730	1.55349195955872
H	-1.05964857944690	4.42499587651321	4.54084952592757
C	-0.95778501737981	3.71769246108476	2.54608663144675
O	2.33800015440621	2.16634658251267	1.04880970072559
H	1.67730141916389	2.24001813658892	-2.71581876415311
H	3.52963167917306	3.35533360185268	-0.03679830727862
C	-0.13701812938776	2.32695898161897	0.89117372293581
C	1.12790402546454	1.46002613807333	1.06392018726207
H	1.13200829659118	0.68074194740530	0.29022398312919
H	-0.44319638036779	2.37965316415102	-0.15367500084403
H	2.92585834169291	-0.54705271340831	5.63513225781631
O	-1.67743296000966	2.55055244100031	2.69475549696925
H	2.83789982747416	-0.74398170631507	3.87798988851239
C	2.87426120376771	-0.00766607592480	4.68813969024938
C	-1.17135408612623	1.59194482527745	1.76503832012948
O	1.66005955769507	0.73204594526392	4.75752416212165
C	0.90791427763280	0.77473789062405	2.41901100517635
O	-0.50672669089517	0.54505227050227	2.43128292745417
C	1.39692985300222	1.56009593156082	3.63555446162854
H	-2.02068000005282	1.16546082284729	1.24031361725503
C	4.09456795985637	0.86176655275174	4.51533263434602
H	0.62720208466526	2.26000748502721	3.95729206578102
H	1.37702117385349	-0.21018800052822	2.41932258792488
H	4.60456250722992	0.03610868622379	2.60770388237600
H	3.83097383709773	1.87842205251664	6.38644915099856

C	4.87482918009059	0.76062593973063	3.36873169755381
C	4.43838159126050	1.79458387679921	5.49215332399471
H	2.28860018522184	2.12878467343024	3.36662776174801
C	5.98656249863020	1.57878443922916	3.19572970257239
C	5.54471613607175	2.61449356367681	5.32192778531416
H	6.58523501616587	1.49331714866233	2.29747407053290
C	6.32159013745165	2.50742332836434	4.17173785434231
H	5.80568106005458	3.33612028432519	6.08572779902128
H	7.18470161847069	3.14744550848966	4.03964469799465
H	1.91599355863625	-2.65042803065607	5.24197983374113
C	0.86638501858718	-2.90541118913031	5.18601986352797
H	0.83104290905058	-3.88306761572638	7.09542854877396
H	0.61617602787382	-1.99908984142844	3.26820718198871
H	-4.25324953096558	-3.60080218769938	8.37625114125526
C	0.25662966733442	-3.60056784352909	6.22224106684412
C	0.13315097553052	-2.54239880771604	4.06920409540742
H	-0.78873254632798	-1.56407063476457	7.83663646712917
H	-0.70733370460358	-1.17001572044112	10.27614744319163
H	1.86230112923804	-6.49677262820384	1.88374029055215
C	-0.99361541578324	-0.57928968089678	8.23816764644277
C	-0.94978531731942	-0.35443065837264	9.60742784647345
H	-3.23947990907051	-1.51052076909989	7.57535576838679
H	1.28557768604456	-4.11766401013707	2.17338922357285
C	1.10296478723648	-6.04871242675110	1.25536840806921
C	0.77601318842950	-4.71076760547637	1.42593238660481
Se	-1.23488092380808	0.17891095494790	5.47423199070312
C	-4.47238161718604	-3.24731921780848	7.37742151383999
C	-1.21502376261925	0.91235153607318	10.11357064988796
C	-1.31006130659840	0.47073364221702	7.38153834811728
C	-3.89941244874927	-2.07023423009023	6.92624945693484
H	-1.18270832858567	1.08466986152961	11.18161251987435
C	0.47180020606213	-6.80174037545825	0.27199385844203
H	0.73522120658606	-7.84306231861292	0.13771034587641
C	-1.50834476907842	1.96156415117733	9.25118637176750
C	-1.54774823494998	1.74779406796945	7.87941793760365
C	-0.19521721440199	-4.13905326145518	0.61151332822545
C	-1.08616410475017	-3.94837861559788	6.13536327514604
Se	-0.64345782678756	-2.27848155415834	0.80102703172670
C	-1.21562374663969	-2.89137542977126	3.97661561094458
C	-0.49023694333925	-6.21988790657274	-0.54516379478518
C	-5.31927298756674	-3.97468007131770	6.54656143269008
H	-5.75462413494405	-4.90102082106993	6.89827128995166
H	-1.69779711697991	2.95250435041507	9.64275502133466
H	-1.75026649155626	2.56968049213560	7.20652075860183
C	-0.83249951840472	-4.88437637927313	-0.37507182677914
H	-1.56180562182293	-4.49991402695320	6.93588137254845
C	-1.82052226768009	-3.61247950266362	5.00928443017444
C	-4.17406119632824	-1.61916223918121	5.63467821669043
H	-4.97109221213542	-2.92234300096692	1.58045052175403
H	-0.97939618748160	-6.80439530489180	-1.31341813454274
H	-5.76331022450000	0.08301424277059	6.77779824715582
P	-2.13908327169367	-2.55180677554159	2.46820666201123
P	-3.41467746925533	-0.10274501991447	5.04078575257544
H	-1.58794961963794	-4.42678617031742	-1.00133339535881
C	-4.51230083868474	-3.88547500715795	1.75383402600226
C	-5.38748554462180	1.08099444192261	6.59492423021942
H	-6.28938428113603	-4.94172610184678	1.21046107700516
C	-5.26458203483008	-5.03219434245697	1.54495274635361
H	-6.87257451085327	2.01380373665138	7.82277188314226
C	-6.01551399488867	2.17295618059182	7.18220507642192

C	-5.61772994463973	-3.50951571334605	5.27303348690208
C	-4.27794888161451	1.28941083882927	5.77954117241621
C	-3.19348748545138	-3.99148605620694	2.19806019085025
C	-5.05423375570349	-2.32552935580169	4.81512787578904
H	-2.85350523455713	-3.92859354736994	4.93669415801743
C	-5.54663750509102	3.45701064716025	6.94631230239057
C	-3.80217468893389	2.58263434388196	5.54285356815699
C	-4.70794731732749	-6.28420081121345	1.76896176642989
C	-2.63612177422111	-5.25510021499679	2.42623073283698
C	-4.44405366171282	3.66298737457406	6.12092474208274
H	-6.03986159767849	4.30372770190328	7.40597467871608
C	-3.64086048450148	-0.08827928912291	3.22808743571068
C	-3.18841321217782	-1.05201201219893	2.29337155922092
H	-6.28934716782788	-4.06363505233570	4.63028834560712
H	-2.93830002773237	2.73566185217684	4.90476559467576
H	-5.29892780399619	-7.17581616187498	1.60428286606302
C	-3.39445283268758	-6.39378966134814	2.20931027423808
H	-1.61713660282353	-5.35655783769145	2.77753401173655
H	-5.31110879554049	-1.96234184031278	3.82916862920398
H	-4.08024849016412	4.66508529143539	5.93523315232910
H	-2.95618529667899	-7.36684556881046	2.38737018093226
C	-4.45731425159530	0.94678995282035	2.77266835533010
C	-3.60617423131971	-0.92746453027690	0.96509283534003
H	-4.82194535424853	1.69076313606517	3.46325847898315
H	-3.28839792201616	-1.65305049364330	0.22895224618611
C	-4.84699386270993	1.05509973053263	1.44424939635801
C	-4.43084175632497	0.10467204138162	0.53771261124633
H	-5.48094215864958	1.87711794458255	1.14074140540452
H	-4.72934805630340	0.15168376120639	-0.50079175210869

Ternary encounter complex 11 for XB catalyzed *C*-glycosylation pathway

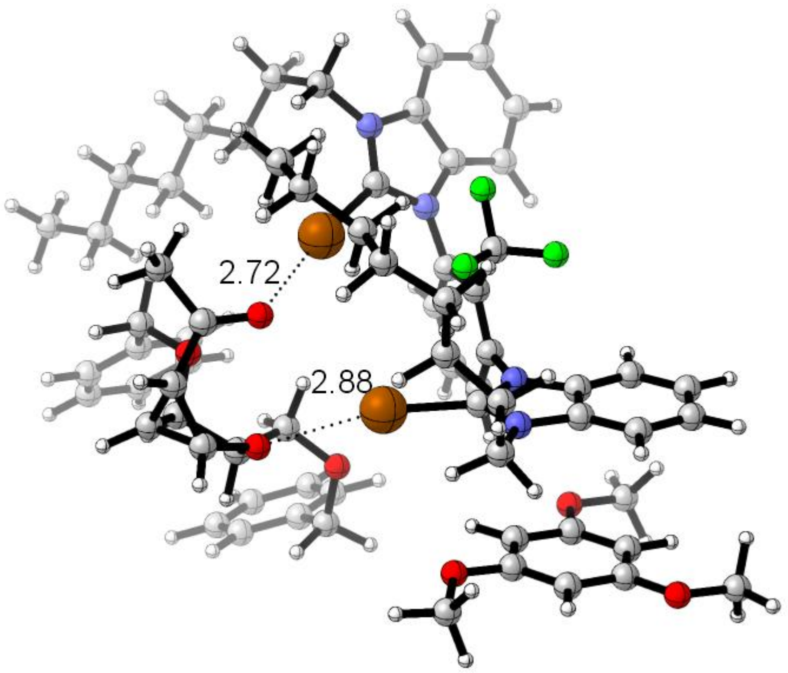

165

H	3.03363077416677	3.40182026457303	5.29985323740980
H	5.06866797563222	4.10925898386720	6.51701273315764
C	3.99697217443996	2.98608950261929	5.03111337123790
C	5.14266686547484	3.38641934175248	5.71411351267150

H	5.51231604633481	-2.46006367660438	-0.73825664157785
C	4.09090107259197	2.05994674291078	4.00514445696160
H	6.11131289821942	-1.33236487563629	0.48027826279678
H	3.20169380456053	1.74829354915272	3.46645705530573
C	5.21163018231487	-1.79011884917494	0.07322745887452
C	6.37570978983572	2.85591790703611	5.36654170487177
H	4.54312560837589	-1.03261298462441	-0.33316586839444
H	6.42893047520420	0.15765658492110	2.39953514566195
H	7.26897093048831	3.16280762497280	5.89576384784059
C	5.32673782948415	1.51397357572132	3.65454506585804
C	6.46504417025859	1.91731721121279	4.34121133638120
C	5.39392574223328	0.47753490274089	2.56182311436991
O	4.57002288655974	-0.64536316638627	2.85630073239507
H	7.42694125221706	1.49250287158867	4.07665522201259
C	4.50885019317511	-2.62157828798904	1.10485983242642
H	5.00661110481546	0.87923260294023	1.62296133095681
O	3.30442479878066	-2.79052377252666	1.08458289292205
C	5.08499618011408	-1.47875815500894	3.87787203477500
C	5.39297959569745	-3.32843722483339	2.07973389777547
H	5.68135222203507	-0.88878455674528	4.57776187945767
C	5.88901397283270	-2.66382727933065	3.34566824226610
H	6.16039971704159	-3.90885010900422	1.57707929650713
H	6.93507933751951	-2.75344043276098	3.59544426807576
O	1.71367891110967	-1.88972869766455	5.60649680868603
C	4.95537763736500	-3.83865944753675	3.40954729337473
C	3.94256267279122	-2.17263969309736	4.61925736302811
H	1.18408875205479	-2.27897676755324	7.49656043083176
O	3.71331369177042	-3.39536552821593	3.89819521974200
H	4.48624849148500	-0.91587583157508	7.76474253325874
C	2.67894177504246	-1.34886146046922	4.72690745089753
C	2.06367463019080	-1.88423839676756	6.98722875912010
C	3.70182165992878	-0.17208425298878	7.85566996788191
H	2.20134942907912	-1.28353075088239	3.74767531225324
C	2.39677521020982	-0.50917967731511	7.50890687991735
C	4.00374447541450	1.10313805592064	8.32088816651718
H	5.02288860365854	1.35425707491663	8.58649989595150
H	4.31760059684917	-2.43774825878012	5.61294833821565
H	5.25995722375892	-4.81775543054680	3.75290459079956
C	1.39547054406564	0.45273948301470	7.62693025178011
C	2.99917211483096	2.05452518522256	8.43653449748408
C	1.69270170983231	1.72804692025456	8.08732117736557
H	0.37596665583249	0.19251690898503	7.36507688390498
H	3.23223824169947	3.04897758496008	8.79576717256294
H	2.89659663365778	-2.56654863720068	7.17861681601842
H	2.96067422829849	-0.33455394811819	5.03088576944049
H	0.90632092582854	2.46632664068469	8.18154111162235
H	-4.74087464773471	-3.63196714042829	7.85432670925005
H	0.58014114109611	-8.42220977293499	6.94513158193501
H	-5.63602016842199	-7.54701179072086	7.65178786487343
C	-1.41768948964599	-5.13269480704048	6.26696602966967
C	-1.01515002078781	-6.45919502381655	6.12463500570886
C	-1.93497572747460	-7.49682543367940	6.19044227894324
C	-2.75984630672387	-4.85592946551281	6.48537065115298
C	-4.42325448668414	-3.19727554883232	6.90464872530798
C	-3.71184234885520	-5.87509118949559	6.55923122661477
C	-5.49450908390876	-8.01005133089582	6.67306934055685
C	-3.28011226775129	-7.18717332585581	6.40070095494951
C	0.81208663704415	-7.97561968833715	5.97633453196286
O	-4.11873091946384	-8.24894139625954	6.42183879702016
O	0.30946609806880	-6.64964898362441	5.90403501285754

O	-3.07684362559250	-3.54394220463898	6.61816157774152
H	-0.68316709442162	-4.33750268436913	6.23876536030954
H	-4.44548296343760	-2.11304260722831	6.97170054947052
H	1.88861003601897	-7.89577714442335	5.85367424648419
H	-5.97688869934201	-8.98307685528563	6.65469162465600
H	-1.65199630567384	-8.53361732637279	6.08402741214793
H	-4.75265238580255	-5.64913945217298	6.72037425732809
H	-5.92637705756030	-7.37078717848551	5.89996854443697
H	0.40167908088608	-8.60175388635442	5.18159230028097
H	-5.09079546403928	-3.52953434915394	6.10709914622777
H	0.29543402169838	-7.05724984684266	3.24537287414756
H	-0.05723946523306	-5.77481522676714	0.57783230975645
H	2.32097970744175	-7.06917441583137	-1.37024864878388
H	3.46840948001199	-5.08083725389356	-2.42099454874975
I	1.06460439122209	-4.18213139595952	3.08824023644248
C	-0.71124284809501	-7.20172080038998	2.85419838909104
C	0.23488936667721	-6.82688261195990	0.52441839627263
C	1.37246048847812	-6.67184796761829	-1.74733727959374
C	2.51262427783464	-4.60555767417220	-2.66317438067956
H	-1.22437139394295	-7.91986657007442	3.49292813574607
H	1.25498929885248	-6.88627818251160	0.92092708687472
H	1.28765030991639	-7.00696748489519	-2.78532463401357
H	2.27284839787267	-4.90113493530207	-3.68862942284049
H	-1.67461781194612	-2.34878737239080	5.28028860412987
H	3.40702379023594	-2.72217691793995	-3.28872676846747
H	-1.25667517537704	0.10396873742632	5.37421009427882
C	-0.92457167677822	-4.70777003953214	3.04422432651592
C	0.22675951016739	-7.27050711630581	-0.93469524399876
C	-0.68473046524691	-7.66434069081201	1.40283851726925
N	-1.43247487714910	-5.93406599844771	3.00982339666476
C	1.43855123949781	-5.14746299369012	-1.72559578172460
C	2.66310822821268	-3.09218328863711	-2.58155741559352
C	-1.60861342654568	-1.75963349748401	4.37569707291684
C	-1.37515534521883	-0.39328403548871	4.42192861015620
H	6.80152785978199	6.57051787386723	3.86522430360967
H	-0.73157647790682	-6.99579713773230	-1.38703951691054
H	-1.70340469118323	-7.66360697543733	1.00368846171119
H	1.64217937705230	-4.79398291818227	-0.70737883673494
H	2.97509510221354	-2.79442655682187	-1.57813546613737
I	1.47092851168403	-0.88584578296299	0.42910164709595
N	-1.92917771529297	-3.81018070673379	3.13592335397536
C	-1.75882373666451	-2.38757603562110	3.15336940098818
H	4.17969699793817	6.26387945957839	1.14300595274996
C	-2.81763138381901	-5.84909816467309	3.11924171312674
C	-1.25219221172500	0.32168736723931	3.24544007290867
C	6.37199651277541	6.54872393125439	2.86246784184179
H	-0.34607490706160	-8.70295915582301	1.40391990573897
H	0.29149074267761	-8.36165007473498	-0.97889020321581
H	0.46404867035575	-4.73140257560853	-2.00776279953544
H	1.71473934122867	-2.59295055149253	-2.79568955570367
H	-3.55303558549500	-7.88527166384135	3.06126775891707
H	-1.02067888935741	1.37920693816413	3.25260813149690
H	7.19227978879248	6.45655013303741	2.14746724611896
H	4.59110476442664	5.48809084618770	3.45209310426190
C	-3.79809138972321	-6.83453101600287	3.13902950017017
C	-3.13661692061022	-4.49915705050041	3.21279488817689
H	2.46602251929403	4.97401246098043	-0.28447379782459
C	4.75325624186663	5.34679209134868	1.31742309271422
C	5.38379191087079	5.40055318299353	2.70341311990426
C	-1.69999560744831	-1.67842653831012	1.94936533324503

C	-1.40450153237238	-0.31874800961531	2.02328676587218
F	-1.08574017998627	-3.38484968388564	0.42765641109738
H	5.88360931754408	7.50946618047925	2.68545670682744
H	3.10521084179387	4.11173582475654	1.93843873706156
H	0.81409087425226	3.70045136114667	-1.68327930590932
C	3.13233979156512	4.10828999743793	-0.21031457896243
C	3.84805236333944	4.13593988725065	1.13313094649594
C	-0.09163692296549	0.41819996438936	0.07442067703655
C	-5.10223498737641	-6.39813105969437	3.27268376022567
C	-4.44442803869249	-4.05416419711137	3.34841470574003
H	5.54243266208790	5.32659744292218	0.55774429769881
H	1.66095605331591	2.68708592473771	0.44044679296131
C	-1.98441795389281	-2.41941867783376	0.65512990398809
N	-1.18775628759420	0.46962613727766	0.85198282640570
C	1.51506073966584	2.86168976099016	-1.70637020332684
C	2.32730596231774	2.83096283385058	-0.41829559292252
C	-5.41960828973032	-5.03357995915559	3.38104255780883
H	5.88316485601323	4.44763430774101	2.90875137508357
H	-4.68026374658017	-3.00045649911327	3.42064317311824
H	-5.90184176928220	-7.12645946822289	3.29472054708182
H	3.86340483243371	4.20794565378214	-1.01925331327854
H	1.43276311967045	0.71948974194607	-2.04373486844594
F	-3.18255415935812	-3.00714397687782	0.71327832446517
N	-0.22219119935290	1.27746853280080	-0.93024538908487
C	0.75675459125979	1.57102057867819	-1.98363160926137
F	-1.99257090695538	-1.63578324240259	-0.42056688790359
H	4.44548436898300	3.22420169878292	1.24706711797744
H	2.17678967923406	3.02694607185283	-2.55997290061606
C	-2.06553083177562	1.41338555120860	0.33006965099724
H	-6.45603816769418	-4.74183180531693	3.48706986216929
H	3.00796303078654	1.97229645497847	-0.43422498841676
C	-1.44594792003308	1.92967868085363	-0.80396736005987
H	-3.80011945392538	1.41427706663574	1.62168839897330
C	-3.32446186989943	1.82897539297620	0.74315921783189
H	0.19838636222278	1.62828559768885	-2.91734360770370
C	-2.05506546002557	2.90339770487585	-1.58779885132018
C	-3.92973355393154	2.79722107212067	-0.03537848069308
H	-1.57809701222358	3.30798187367794	-2.47002904774364
C	-3.30631152428564	3.32447474547794	-1.17850584599234
H	-4.91111952376241	3.15819748500031	0.24102704761689
H	-3.82168333709126	4.08045851664460	-1.75528941039168

CYLView[32] rendering of binary complexes 5-complex and 7

Binary 5-complex (PCH catalyst J + intermediate 5 without nucleophile)

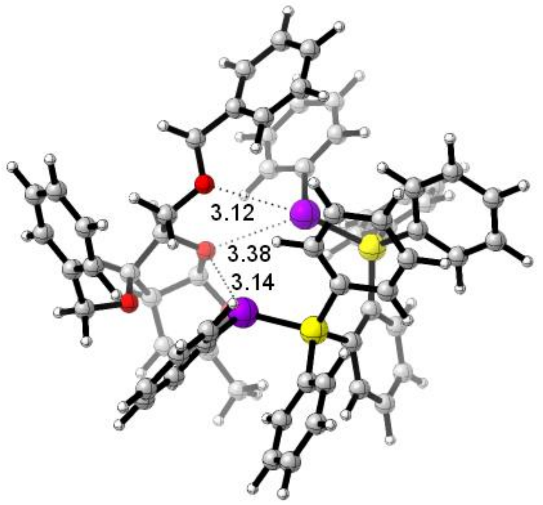

130

H	3.09859513300262	4.18188340157903	4.71111352521963
C	3.27134756028089	4.02437025585477	3.65370752011593
H	5.39236121525437	3.75865003828915	3.87803755000310
C	4.56103644857791	3.78804012089978	3.18522292959245
H	1.19879997825047	4.23615197095780	3.12315651228483
C	2.20837695301423	4.06017583700534	2.76365571324919
H	-3.85570545497860	0.62991081133085	0.73143877380097
C	4.78120166781457	3.60206714380033	1.82660070389135
C	2.41814500623668	3.85475631575518	1.40111653252136
H	-1.47735300433778	2.45396251317393	-1.39176577996148
H	5.78425466909442	3.42847013799940	1.45807885512009
H	1.53635327674668	3.93882729244704	-0.56066653984611
C	3.71126670707360	3.63711551599509	0.93783146374054
C	-3.63861880155554	0.75720425615492	-0.33292873574237
C	1.23212440716021	3.78214669167915	0.47815993249120
H	-4.19076950859938	-0.00955107103484	-0.87963396567039
C	-1.27482186635108	1.44931392449087	-1.05729244939211
H	-3.97815316370129	1.74030042763715	-0.65216072867092
C	-2.18249508242765	0.61382574912659	-0.56625797414126
O	0.55107756491303	2.53611509440361	0.59869745311967
H	3.88207286948558	3.48810279301521	-0.12268740738396
H	0.49840468328995	4.54305551175513	0.74476108228622
C	0.07779999639031	0.79568596974496	-1.04178447698982
C	1.13088750119053	1.45788405580875	-0.10833552415496
H	2.00052261245149	1.80184842031289	-0.66974627655590
H	0.49482966445779	0.65242910693774	-2.03825210871596
H	3.24255446107550	-1.70794775341872	2.02569642025708
O	-1.66357138780149	-0.61791205921718	-0.21367914411703
H	4.09082155752681	-0.52238599198497	3.03227097092536
C	3.21358923692868	-1.18174146695768	2.98694698240213
C	-0.26100942696969	-0.54180222985471	-0.34807497376976
O	2.03973839220835	-0.40429377389478	3.10197973325465
C	1.50669065628633	0.29364866808120	0.82373597321755
O	0.30900058994657	-0.46755830918410	0.93532437492100
C	1.96674291771957	0.69128863574672	2.21333352094358
H	0.08223364486448	-1.44191820195282	-0.86359456716088
C	3.25660839697370	-2.18488502419976	4.10947747058468
H	1.25285421790672	1.39041306967215	2.64419148214834
H	2.27226203866877	-0.31614148421311	0.32415442044620
H	2.08913287855561	-1.00241129530686	5.46472186936517
H	4.41693675725428	-3.59638021671022	2.98691839125516

C	2.62712281658133	-1.93189121893719	5.32569483153510
C	3.93919519809563	-3.38538005987140	3.93735755956301
H	2.93697709759105	1.19704800014469	2.14262266854083
C	2.66925222071066	-2.87263069131677	6.34797920299432
C	3.99483123725661	-4.31952960026603	4.96251820748684
H	2.16775124912244	-2.66828466836324	7.28663376572594
C	3.35419710911297	-4.06882552579982	6.17017874453028
H	4.52365971721181	-5.25248499845585	4.81215431290423
H	3.38704006676941	-4.80184815828629	6.96628377341967
H	-5.16962770096364	-4.51755505420209	7.92491690229715
C	-4.16832768528504	-4.31196994977582	7.57037706230885
H	-3.22463490822711	-4.88338637642594	9.41086010520249
H	-4.83740586306865	-3.69878775029112	5.63198241352585
H	0.50775832339061	-0.27899016489448	7.76441388966805
C	-3.07337023942794	-4.51679291452114	8.40373811068234
C	-3.98092986546753	-3.84786706396803	6.27838455878370
H	-0.87544795003738	2.73604960482401	5.40714888046369
H	-0.76478255856945	5.16176163302041	5.80396499513182
H	2.20595059221541	-4.39448605507778	0.18285038623154
C	-1.09530011979070	3.42333021287826	4.59982838379163
C	-1.03107346614943	4.79277796781560	4.82199100444242
H	-0.33218901802551	0.38961108060594	5.54619002008839
H	0.86169416242349	-2.56616779010799	1.18839774046845
C	1.79102240646826	-4.51345207423035	1.17568122361020
C	1.03715943508948	-3.48597039557577	1.72956562927486
Se	-1.55510077479928	1.07184272402442	2.94023113073361
C	-0.54548984914634	-0.38004651811008	7.53807868536365
C	-1.29445046100895	5.68205095693919	3.78615982033231
C	-1.45609224481126	2.95568357071594	3.34130726136542
C	-1.01771520688031	-0.00407887876297	6.28774844031349
H	-1.23539566278777	6.74829735481547	3.96167754936096
C	1.99865250540315	-5.68933687333610	1.88518145592633
H	2.57549838280706	-6.49283593172826	1.44555635565010
C	-1.62928042263965	5.20308222771505	2.52550423570102
C	-1.72271537538635	3.83560586045308	2.29920328988345
C	0.51376603132135	-3.64568522558481	3.00632671253416
C	-1.78991891354909	-4.24380981767759	7.95208738375097
Se	-0.45511539465882	-2.15645395066530	3.76235761288919
C	-2.68693817935738	-3.58065288132667	5.82463536737764
C	1.47375133044723	-5.83251366287162	3.16411182225706
C	-1.42449418183286	-0.87383511490525	8.49247474307825
H	-1.05318570961992	-1.15833301400414	9.46884020148929
H	-1.82940676437437	5.89275467767928	1.71549348978093
H	-1.99630658827627	3.45531095450320	1.32223437548667
C	0.74110582095657	-4.80347933889683	3.73894234212800
H	-0.93866081381730	-4.38858666280232	8.60446145048265
C	-1.59128275439324	-3.76862762171484	6.66263768254711
C	-2.37270916103799	-0.14722122410729	5.99563308382523
H	-2.69526927357810	-5.95907689983960	4.84160489591443
H	1.64547366090292	-6.74160183176216	3.72621396793729
H	-3.77357781310158	1.79581750239061	7.01394885371947
P	-2.44033626657983	-3.14402406679660	4.09707119520409
P	-3.08738519106119	0.48502580105175	4.47760481926042
H	0.35322895038392	-4.91425026632609	4.74336051999060
C	-2.53966605336489	-5.89650184762384	3.77255212301496
C	-4.16197395376140	2.38850515958482	6.19794598856784
H	-2.53915621777564	-8.02152544965156	3.50921993890884
C	-2.44742246848936	-7.06121073448013	3.01972623506705
H	-4.90397169005104	3.90891744556849	7.50672116917869
C	-4.79598785820909	3.59251961803590	6.47793787338672

C	-2.77800519340960	-1.01571959027029	8.20162149510202
C	-4.01957581231544	1.97671036047446	4.87444355701730
C	-2.42038671210140	-4.66366225533017	3.13845474402307
C	-3.25510488502207	-0.66569885922021	6.95130364137899
H	-0.58936167210921	-3.53062517396201	6.32548885781880
C	-5.27425486316711	4.38635361454254	5.44651632146185
C	-4.49166105205975	2.78467067624593	3.83410968915216
C	-2.23269647461205	-6.98996778830021	1.65148749174835
C	-2.20670109082822	-4.58786027917945	1.75984791598133
C	-5.12221267225627	3.98104257117588	4.12351133740478
H	-5.76013471865621	5.32746125083803	5.66926603188649
C	-4.18341455493106	-0.78800511205737	3.75359820747789
C	-3.88930773342126	-2.16063238845001	3.54113848067347
H	-3.45846098623448	-1.40959032281834	8.94465518845979
H	-4.35449475188847	2.48665088026704	2.80077122932273
H	-2.15520219723807	-7.89932319430892	1.06961082061252
C	-2.11519504627443	-5.75335064144540	1.02060615309654
H	-2.10475944367103	-3.62464662876046	1.27174121299864
H	-4.30957877317215	-0.78035123299849	6.72324882719779
H	-5.48499422511051	4.60319796445308	3.31613608526991
H	-1.94625215891794	-5.69971496416090	-0.04674778814716
C	-5.42696590660101	-0.30406799981442	3.35137049259295
C	-4.85824955365979	-2.95319607663294	2.92349015957903
H	-5.68987378109911	0.72688717315500	3.52669200379188
H	-4.67434571368517	-4.00493661417588	2.76214106492592
C	-6.37231195521680	-1.11402100573735	2.73526299927998
C	-6.08440297375363	-2.44237947947413	2.51521236121954
H	-7.32403988104300	-0.69226065091337	2.44164884689376
H	-6.80128487632213	-3.09890311429204	2.04092662181462

Binary complex 7 (PCH catalyst J + cyclopropanated donor 1a)

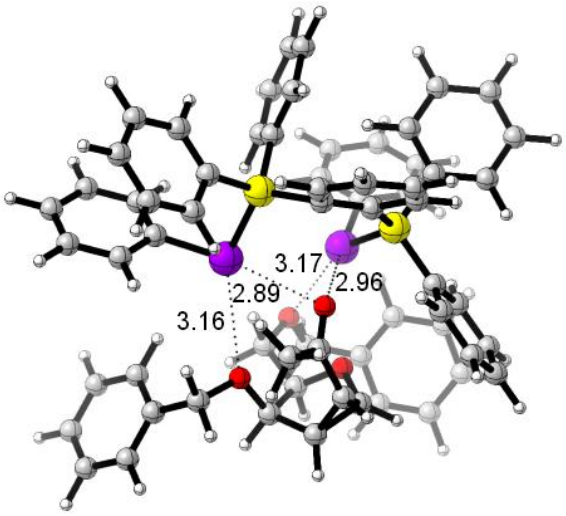

130

C	-3.36276914225788	2.98168085524602	1.44108732961166
C	-2.76553369239233	4.14617178984745	-0.42590803478436
C	-2.32531373666419	2.73149718109162	-0.67949688941299
C	-2.43454270197760	2.03067596627327	0.67428054188795
O	-1.18693796836985	1.92836631252070	1.33762981258370
H	-2.88660930969342	1.04105870218044	0.57199595154235

C	-3.20442727775319	2.90983965370414	2.94535556736973
C	-1.34745476434614	3.88585561785197	-0.79467976959564
H	-2.59240350651430	2.19990285672310	-1.57989701386082
C	-0.16594897070772	4.05308544817676	0.10516279459574
H	-1.08156919259993	4.10026775964166	-1.82590452535077
C	1.08202754074790	3.34659743184054	-0.33857074092893
H	0.85749693455434	2.40863416753779	-0.84332939109213
H	1.74233391898582	3.18585903191155	0.51176354048807
H	1.58603264261903	4.00306347410889	-1.05551631617754
O	-0.15114984352123	4.80040554441125	1.06303352401936
C	-0.47803852708813	0.73155658434343	1.06210863550811
O	-3.95600330918798	3.85313165760416	3.68055806744895
H	-2.16451785456915	3.08713804183673	3.21198202972002
H	-3.46787033859818	1.88891611372840	3.25255264625535
O	-3.05554718737576	4.28084490460824	0.93741787664338
H	-4.39144003081414	2.73261928542000	1.15359642034075
H	-3.41568245043296	4.69312727870502	-1.09674431645311
C	-5.36467161235298	3.81860257577103	3.48389470217274
C	-5.81231585188173	4.87562770793282	2.50421151902280
H	-5.81067867646851	4.01139449958647	4.46180981025816
H	-5.68338615897673	2.82441522319753	3.15657639054318
C	-6.45901647845316	4.55482348693628	1.31737545853978
C	-6.81718036843566	5.55281865601784	0.41581848557924
C	-6.53015916605084	6.88153395586878	0.69901967456671
C	-5.89565273802053	7.21202991782563	1.89393260219674
C	-5.54724208654708	6.21368834515048	2.79141185527138
H	-5.05930713331157	6.46798892344637	3.72589779965757
H	-6.80683312014294	7.65849291109451	-0.00265131642718
H	-5.68116397955966	8.24847696047513	2.12786035356692
H	-6.67741120675757	3.51718568216936	1.08928487638786
H	-7.31847069106525	5.29069553351062	-0.50740308278896
C	-1.01027866445437	-0.43486731633978	1.85706713535889
H	0.55861210728421	0.93555018518357	1.34466317878679
H	-0.49869910623631	0.50870497753385	-0.01023183681276
C	-1.25866186455787	-0.28215925686926	3.22063967716774
C	-1.23511227451619	-1.66992345585380	1.26087337545747
C	-1.69405913691119	-2.74438462934295	2.01645488864781
C	-1.93647818153557	-2.58625753421692	3.37399582032406
C	-1.71951309855272	-1.35025409826142	3.97612971349884
H	-1.05259297962620	-1.79217871049855	0.19917446799121
H	-1.86795278970400	-3.70170960619401	1.54180390533399
H	-2.29673198964681	-3.42045031296251	3.96231159252072
H	-1.90829335338117	-1.22152243169579	5.03454486711830
H	-1.09470040137432	0.68328904304188	3.68786333964688
H	-1.56285798029380	12.25905402394981	2.12419713887407
C	-1.15165561149298	11.41642280910304	2.66396756188517
H	-0.76034994033376	12.54277053486000	4.44516729653363
C	-0.69825181824573	11.57409385030031	3.96639829328160
H	-1.43146038269405	10.06008025819228	1.02627644185875
H	-0.15901562583060	6.67324691614071	7.70491690840660
H	-3.20133861101221	6.93172024146520	7.87696739189973
H	0.55529020192198	3.09627495929085	6.45532630689684
C	-1.07899282669317	10.17515036671158	2.04333553071616
H	1.53801392560315	1.48131056251726	8.03088148032267
C	1.20481690103792	2.31442058236902	6.08700637440444
C	1.75409882543219	1.39742307397546	6.97385639850782
C	-3.19055248342276	7.34659310951771	6.87732455836607
H	0.12668998162598	5.14263200650687	5.78289895774605
H	-2.01528620916773	5.73883914406490	6.08182208685310
Se	0.67224076025450	3.38717182810118	3.44008119620395

C	-2.51069893492864	6.67614031776024	5.86596981018416
C	0.64890568572573	6.83437628165666	7.00362852064803
C	1.49106967696761	2.20754875730392	4.72979750762876
C	2.56918130610547	0.37442194815690	6.50471310064459
C	-3.84477952941352	8.53886851659342	6.60537607912699
H	-4.36598095597642	9.06449583827382	7.39483392451842
C	0.81117833419223	5.96772477489317	5.92908916876042
H	2.99409374004028	-0.33873718421549	7.19918935450583
C	-0.16296709440024	10.49195335377159	4.65879050643826
C	2.29211903180351	1.17439209000365	4.25182001392001
C	2.83131952381461	0.25826065086928	5.14490369639986
C	-2.49571232448764	7.20006443031946	4.57784064360180
C	-0.55202811889741	9.09292006358850	2.74200309162761
C	-3.84602410862972	9.04854997773549	5.31064186066363
Se	-1.73147638570684	6.06807033702959	3.21553253076113
C	1.51707549792735	7.90393813237938	7.17106274135073
H	1.38587381284158	8.57862706516012	8.00716127566066
H	0.19112366795725	10.61155414953745	5.67470535546035
C	-3.18929592397165	8.37459209480802	4.29292221706807
H	2.49125384324682	1.08682348461384	3.19122351316737
H	3.45503193473628	-0.54556672123535	4.77576232991408
C	-0.09277981727694	9.25071544928220	4.05118264710523
C	1.84627628913337	6.17865585320131	5.02798067914009
H	-4.37444791817642	9.96608558431539	5.08570399910055
H	-3.14517287425319	7.89057575482260	1.19230507914001
H	3.90164924839691	4.89200490172548	5.94966856659874
P	2.13777104958359	5.08328520557664	3.62828012780396
P	-0.44082358521458	7.45054066801201	2.01090487575130
C	4.30380495745531	4.27194652130703	5.15946747819234
C	-2.56508570668833	7.72029163293875	0.29300967259652
H	-3.23306765686567	8.76658066308069	3.28629209472177
H	5.89825061898374	3.52896375928880	6.37923045005199
H	-4.27013091805509	7.94668134292009	-0.97421646566598
C	5.43252019021477	3.49743946155400	5.40324733465600
C	3.69946014114946	4.22795386428976	3.90620778024712
C	2.55292669094778	8.11626356668966	6.26558409832882
C	-3.20821585542546	7.74187762936346	-0.93438814383764
C	-1.19503959394467	7.46296301023453	0.36138702409115
C	2.72091733790654	7.25896038662658	5.19111414629348
H	0.29947625277544	8.40353214240704	4.60317924571377
C	5.95565886568568	2.69171596522366	4.40314417760913
C	4.21055473658565	3.39385686407924	2.90737482962548
C	-2.49432889679929	7.48375517653563	-2.09804646756045
C	-0.48357357517964	7.19226022852037	-0.80474628853987
C	5.34517549871209	2.64219325561668	3.15317159196963
C	2.30566383294685	6.23379577820058	2.20722783670105
C	1.37257217326585	7.20917110536707	1.78031896130481
H	3.22741077834643	8.95240049438454	6.39393599967033
H	6.83636536912326	2.09326622501928	4.59695320143856
H	3.72040968705502	3.32666712799526	1.94265297803551
C	-1.13660206672073	7.20229772841575	-2.02954041873155
H	-2.99847084685808	7.49160994489334	-3.05585186471285
H	0.56970689629531	6.94935292686925	-0.77290906499593
H	3.52665747648975	7.43080134479410	4.48633734587185
H	5.74497417885841	2.00573526503757	2.37496085898447
H	-0.57848872581087	6.98265277901823	-2.93026438652076
C	3.61117249452633	6.31963140107042	1.72151241275141
C	1.82415044826313	8.24326768417824	0.96017843731821
H	4.35959018796213	5.61551846012510	2.04508668410562
H	1.14021306551932	9.02381588138265	0.64920076882008

C	4.02011132665546	7.32574400971323	0.85411079549708
C	3.12954953489526	8.31029533006341	0.49121582332953
H	5.04323518785227	7.34000296369392	0.50365091283332
H	3.42596150524500	9.12907514427346	-0.15021397358840

IGMH analysis[30] of ternary complexes 9 and 11

Green isosurfaces denotes intermolecular weak van der Waals interactions computed from IGMH analysis (Red atoms = oxygen, cyan atoms = carbon, yellow atoms = phosphorus, purple atoms = selenium, white atoms = hydrogen)

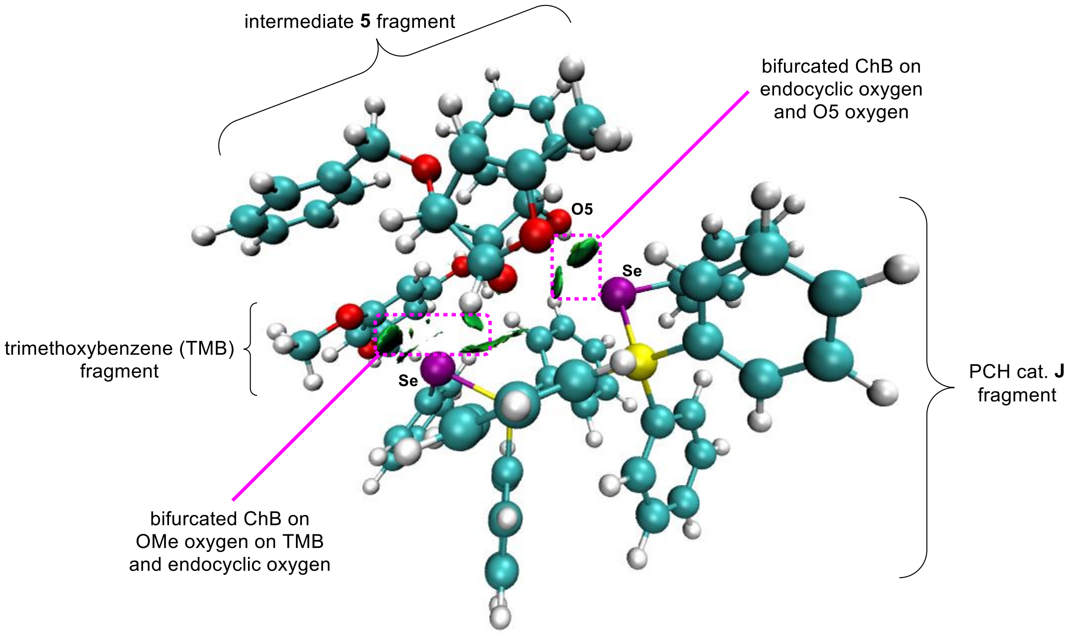

Figure S29: IGMH analysis of modelled PCH catalyzed downstream complex **9**

The DFT optimized downstream complex **9** constitutes a compact ternary geometry where intermediate **5**, TMB and catalyst **J** are held in a favorable multidentate conformation, which is stabilized by four bifurcated ChB s to facilitate the exclusive β-nucleophilic attack.

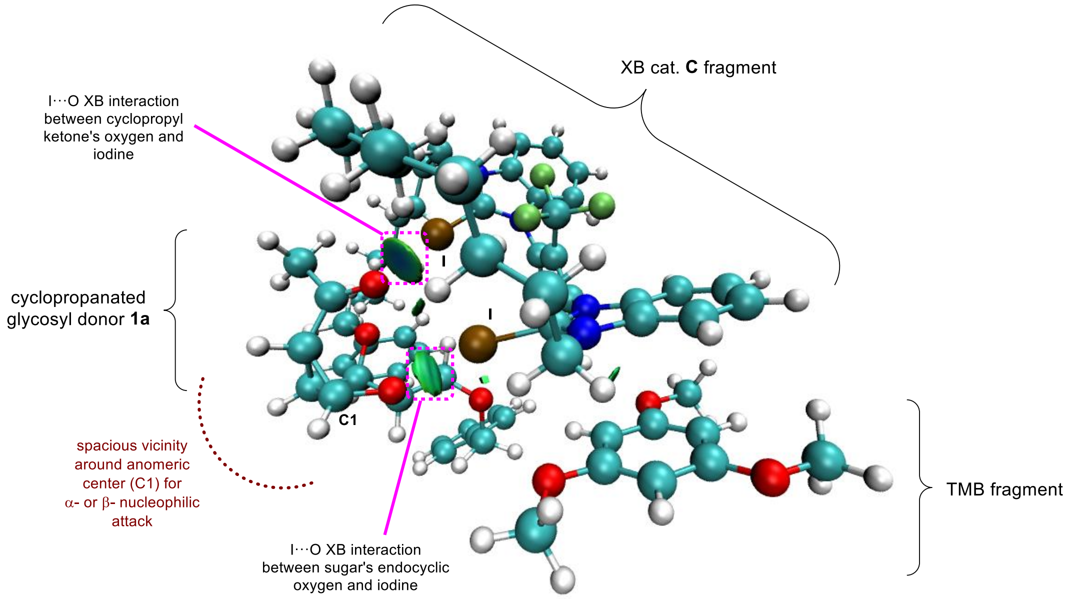

Figure S30: IGMH analysis of modelled XB catalyzed ternary complex **11**

The DFT optimized downstream complex **11** constitutes a sparse geometry where the vicinity of the anomeric carbon (C1) is relatively unhindered for nucleophilic attack on both faces. Further, due to the lower quantity of I···O interactions compared to Se···O in **9** revealed by IGMH analysis, this looser geometry is likely responsible for the lower anomeric selectivity observed in the proposed asynchronous S_N2 pathway by XB catalysis.

IGMH analysis[30] of binary complexes 5-complex and 7

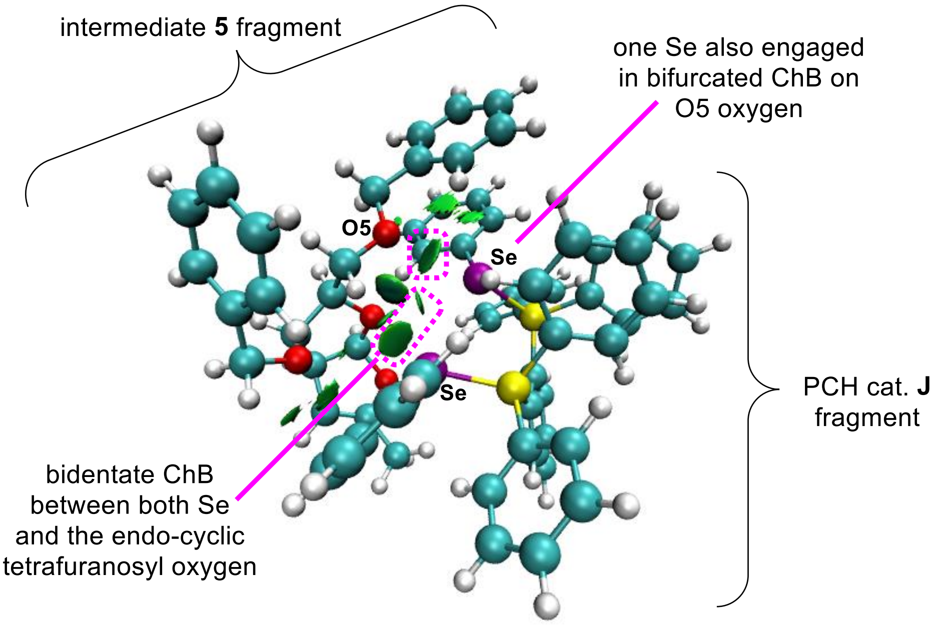

Figure S31: IGMH analysis of modelled PCH catalyzed binary **5-complex**

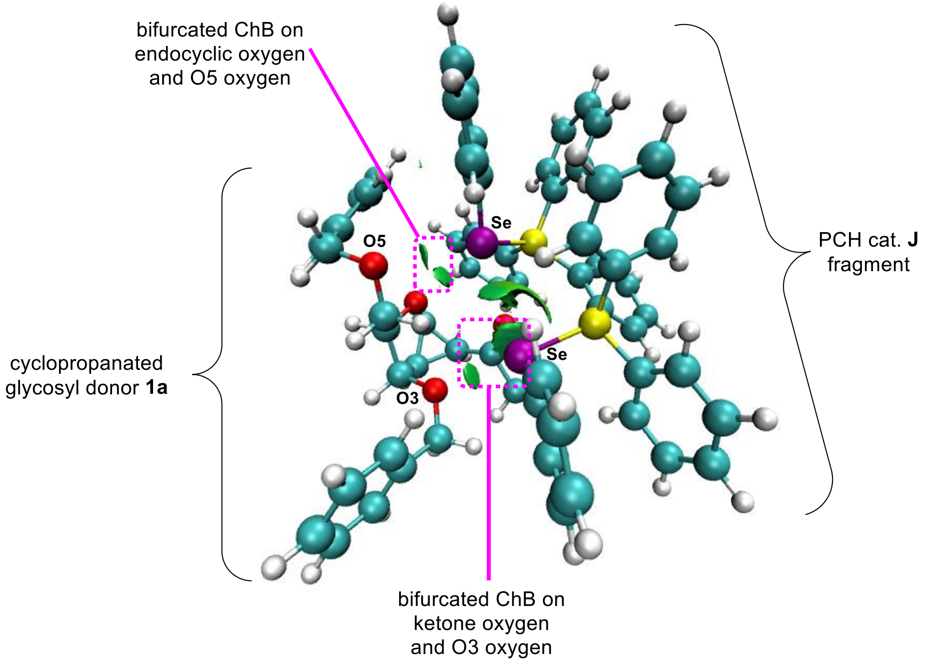

Figure S32: IGMH analysis of modelled PCH catalyzed binary **7**

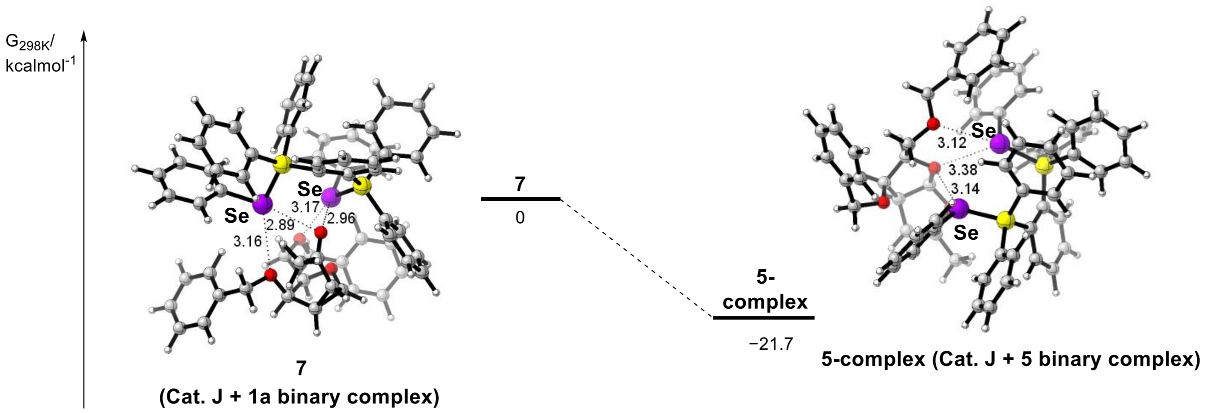

Figure S33: Gibb's free energy diagram for the exergonic conversion of binary complex **7** to **5-complex**.

By modelling binary complexes **7** (catalyst **J** engagement with the cyclopropanated substrate **1a**) and binary **5-complex** (the precursor to ternary complex **9** that involves catalyst **J** engagements with the intermediate **5** and the TMB nucleophile), we derive computational support that **5-complex** is −21.7 kcalmol^{-1} more stable than **7**.

9. References

1. Guo, H.; Kirchhoff, J.-L.; Strohmann, C.; Grabe, B.; Loh, C. C. J. Exploiting π and Chalcogen Interactions for the β-Selective Glycosylation of Indoles through Glycal Conformational Distortion. *Angew. Chem. Int. Ed*. **2024**, *63*, e202316667.

2. Ma, W.; Kirchhoff, J.-L.; Strohmann, C.; Grabe, B.; Loh, C.C.J. Cooperative Bifurcated Chalcogen Bonding and Hydrogen Bonding as Stereocontrolling Elements for Selective Strain-Release Septanosylation. *J. Am. Chem. Soc.* **2023**, *145*, 26611-26622.

3. Wang, C.; Krupp, A.; Strohmann, C.; Grabe, B.; Loh, C. C. J. Harnessing Multistep Chalcogen Bonding Activation in the α-Stereoselective Synthesis of Iminoglycosides. *J. Am. Chem. Soc.* **2024**, *146,* 10608-10620.

4. Xu, C.; Rao, V. U. B.; Weigen, J.; Loh, C. C. J. A robust and tunable halogen bond organocatalyzed 2-deoxyglycosylation involving quantum tunneling. *Nat. Commun*. **2020**, *11*, 4911.

5. Xu, C.; Loh, C. C. J. An ultra-low thiourea catalyzed strain-release glycosylation and a multicatalytic diversification strategy. *Nat. Commun*. **2018**, *9*, 4057.

6. Xu, C.; Loh, C. C. J. A Multistage Halogen Bond Catalyzed Strain-Release Glycosylation Unravels New Hedgehog Signaling Inhibitors. *J. Am. Chem. Soc.* **2019**, *141*, 5381-5391.

7. Bazin, M.-A.; Bodero, L.; Tomasoni, C.; Rousseau, B.; Roussakis, C.; Marchand, P. Synthesis and antiproliferative activity of benzofuran-based analogs of cercosporamide against non-small cell lung cancer cell lines. Eur.J.Med.Chem. **2013**, *89*, 823-832.

8. Li, H.; Homan, E. A.; Lampkins, A. J.; Ghiviriga, I.; Castellano, R. K. Synthesis and Self-Assembly of Functionalized Donor−σ−Acceptor Molecules. *Org. Lett*. **2005**, *7*, 443-446.

9. Khanam, S.; Rai, S. K.; Verma, D.; Khanna, R. S.; Tewari, A. K. An efficient and controlled synthesis of persulfonylated G1 dendrimers via click reaction. *RSC Adv*. **2016**, *6*, 56952-56962.

10. Zhu, Z.; Wu, Q.; Song, X.; Ni, Q. Thermodynamic Controlled Regioselective C1-Functionalization of Indolizines with 3-Hydroxyisoindolinones via Brønsted Acid Catalyzed aza-Friedel-Crafts Reaction. *J. Org. Chem*. **2024**, *89*, 2794-2799.

11. Tanaka, H.; Ukegawa, N.; Uyanik, M.; Ishihara, K. Hypoiodite-Catalyzed Oxidative Umpolung of Indoles for Enantioselective Dearomatization. *J. Am. Chem. Soc.* **2022***, 144,* 5756-5761.

12. Neese, F. The ORCA program system. *Wiley Interdiscip. Rev. Comput. Mol. Sci.* **2012**, *2*, 73-78.

13. Nesse, F. Software update: The ORCA program system—Version 5.0. *WIREs Comput. Mol. Sci.* **2022**, 5, e1606.

14. Bannwarth, C.; Ehlert, S.; Grimme, S. GFN2-xTB — An Accurate and Broadly Parametrized Self-Consistent Tight-Binding Quantum Chemical Method with Multipole Electrostatics and Density-Dependent Dispersion Contributions. *J. Chem. Theory Comput.* **2019**, *15*, 1652-1671.

15. Ehlert, S.; Stahn, M.; Spicher, S.; Grimme, S. Robust and Efficient Implicit Solvation Model for Fast Semiempirical Methods. *J. Chem. Theory Comput.* **2021**, *17*, 4250-4261.

16. de Souza B., GOAT: A Global Optimization Algorithm for Molecules and Atomic Clusters *Angew. Chem. Int. Ed.* **2025**, *64*, e202500393.

17. Zhao, Y.; Truhlar, D. G., The M06 suite of density functionals for main group thermochemistry, thermochemical kinetics, noncovalent interactions, excited states, and transition elements: two new functionals and systematic testing of four M06-class functionals and 12 other functionals. *Theor. Chem. Acc.* **2008**, *120*, 215-241.

18. Grimme, S.; Antony, J.; Ehrlich, S.; Krieg, H. A consistent and accurate ab initio parametrization of density functional dispersion correction (DFT-D) for the 94 elements H-Pu. *J. Chem. Phys*. **2010**, *132*, 154104.

19. Grimme, S.; Hansen, A.; Brandenburg, J. G.; Bannwarth, C., Dispersion-Corrected Mean-Field Electronic Structure Methods. *Chem. Rev.* **2016**, *116*, 5105-5154.

20. Weigend, F.; Ahlrichs, R., Balanced basis sets of split valence, triple zeta valence and quadruple zeta valence quality for H to Rn: Design and assessment of accuracy. *Phys. Chem. Chem. Phys.* **2005,** *7*, 3297-3305.

21. de Azevedo Santos, L.; Ramalho, T. C.; Hamlin, T. A.; Bickelhaupt, F. M., Chalcogen bonds: Hierarchical ab initio benchmark and density functional theory performance study. *J. Comput. Chem.* **2021,** *42*, 688-698.

22. Bauzá, A.; Alkorta, I.; Frontera, A.; Elguero, J., On the Reliability of Pure and Hybrid DFT Methods for the Evaluation of Halogen, Chalcogen, and Pnicogen Bonds Involving Anionic and Neutral Electron Donors. *J. Chem. Theory Comput.* **2013,** *9*, 5201-5210.

23. a) Zhao, Z.; Liu, Y.; Wang, Y. Weak Interaction Activates Esters: Reconciling Catalytic Activity and Turnover Contradiction by Tailored Chalcogen Bonding. *J. Am. Chem. Soc.* **2024**, *146*, 13296-13305. b) Dreger, A.; Wonner, P.; Engelage, E.; Walter, S. M.; Stoll, R.; Huber, S. M. A halogen-bonding-catalysed Nazarov cyclisation reaction. *Chem. Commun.* **2019**, *55*, 8262-8265.

24. Garcia-Ratés, M.; Nesse, F. Effect of the Solute Cavity on the Solvation Energy and its Derivatives within the Framework of the Gaussian Charge Scheme. *J. Comput. Chem.* **2020**, *41*, 922-939.

25. Barone, V.; Cossi, M. Potential energy surfaces for the low-lying ^2A‘ and ^2A’ States of HO_2: Use of the diatomics in molecules model to fit *ab initio* data**.** *J. Phys. Chem. A* **1998**, *102*, 1995.

26. F. Neese, F. Wennmohs, A. Hansen, U. Becker. Efficient, approximate and parallel Hartree–Fock and hybrid DFT calculations. A ‘chain-of-spheres’ algorithm for the Hartree–Fock exchange. *Chem. Phys.* **2009**, *356*, 98-109.

27. K. Eichkorn, O. Treutler, H. Oehm, M. Häser, R. Ahlrichs. Auxiliary basis sets to approximate Coulomb potentials *Chem. Phys.* **1995**, *242*, 652-660.

28. K. Eichkorn, F. Weigend, O. Treutler, R. Ahlrichs. Auxiliary basis sets for main row atoms and transition metals and their use to approximate Coulomb potentials. *Theor. Chem. Acc.* **1997**, *97*, 119-124.

29. Grimme, S. Supramolecular Binding Thermodynamics by Dispersion-Corrected Density Functional Theory. *Chem. Eur. J.* **2012**, *18*, 9955-9964.

30. Lu, T.; Chen, Q., Independent gradient model based on Hirshfeld partition: A new method for visual study of interactions in chemical systems. *J. Comput. Chem.* **2022**, *43*, 539-555.

31. T. Lu, F. Chen, Multiwfn: A Multifunctional Wavefunction Analyzer, *J. Comput. Chem.* **2012**, *33*, 580-592.

32. CYLview20; Legault, C. Y., Université de Sherbrooke, **2020** (http://www.cylview.org).

10. NMR spectra

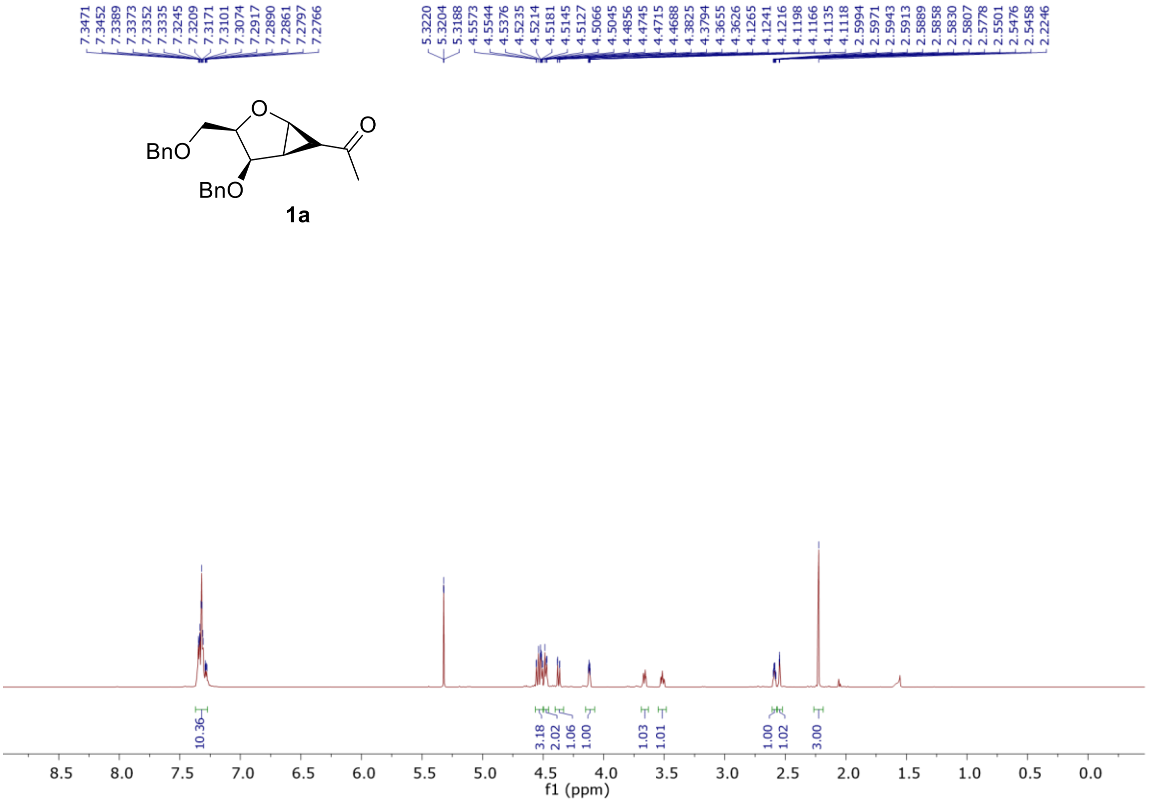

Supplementary Figure S34: ^{1}H NMR spectra for 1a

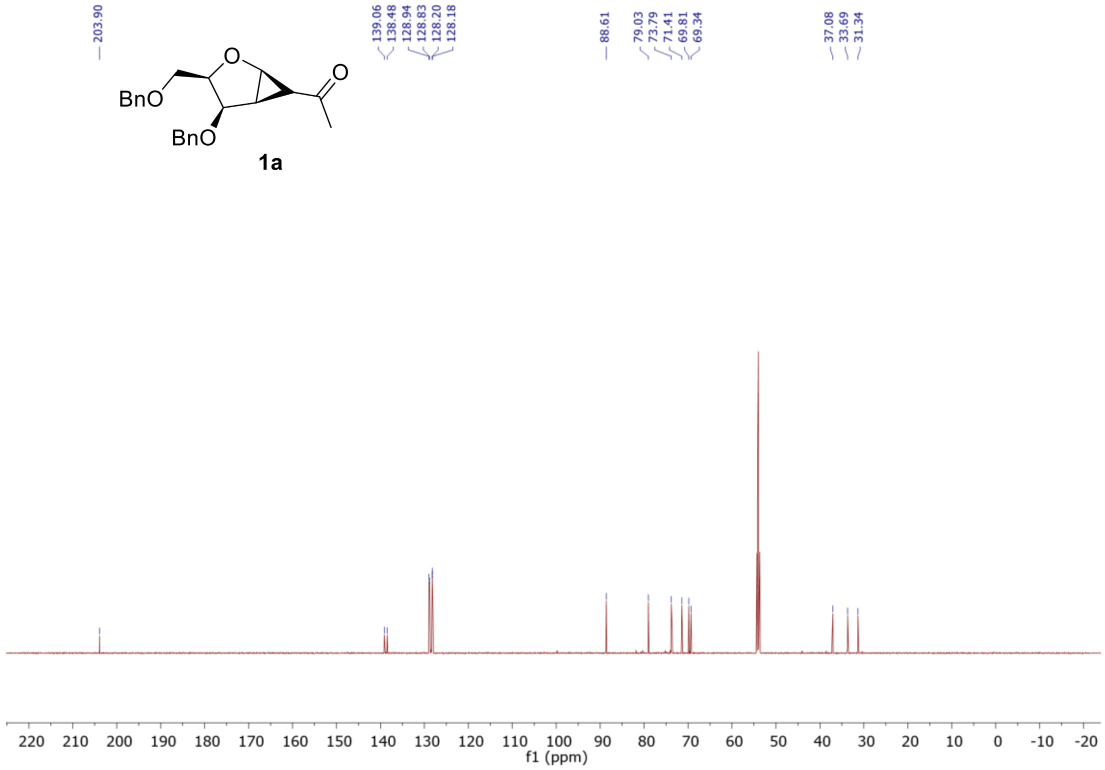

Supplementary Figure S35: ^{13}C NMR spectra for 1a

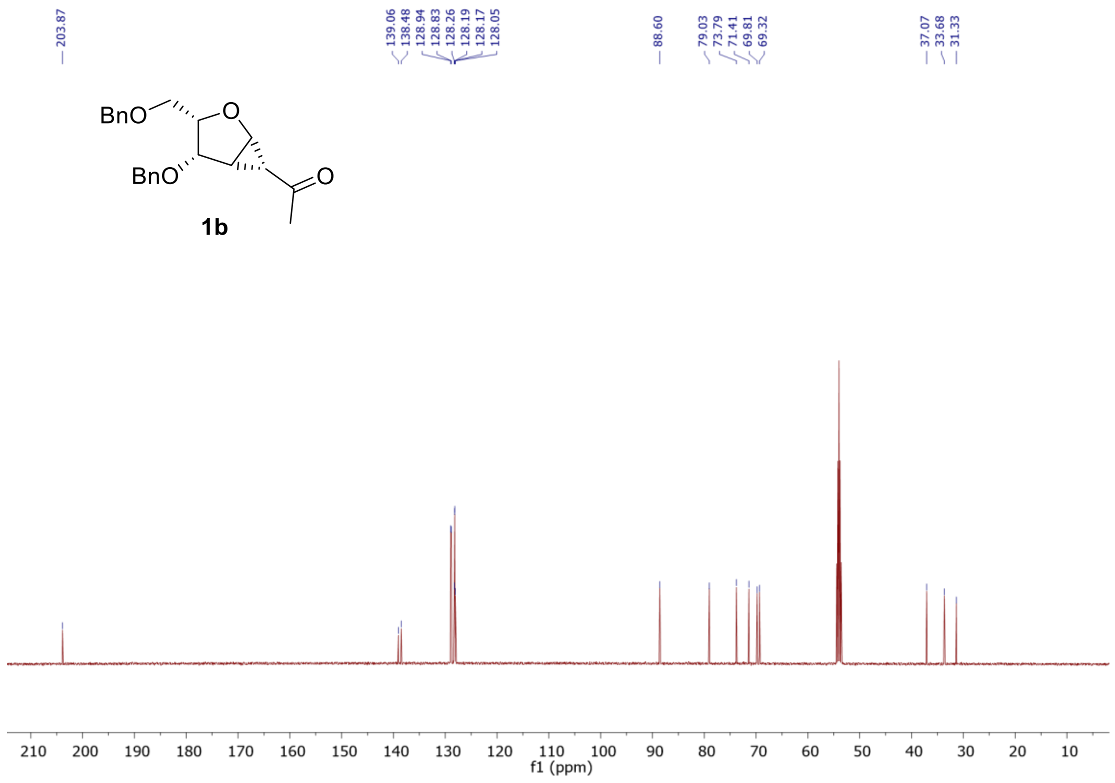

Supplementary Figure S36: ^{1}H NMR spectra for 1b

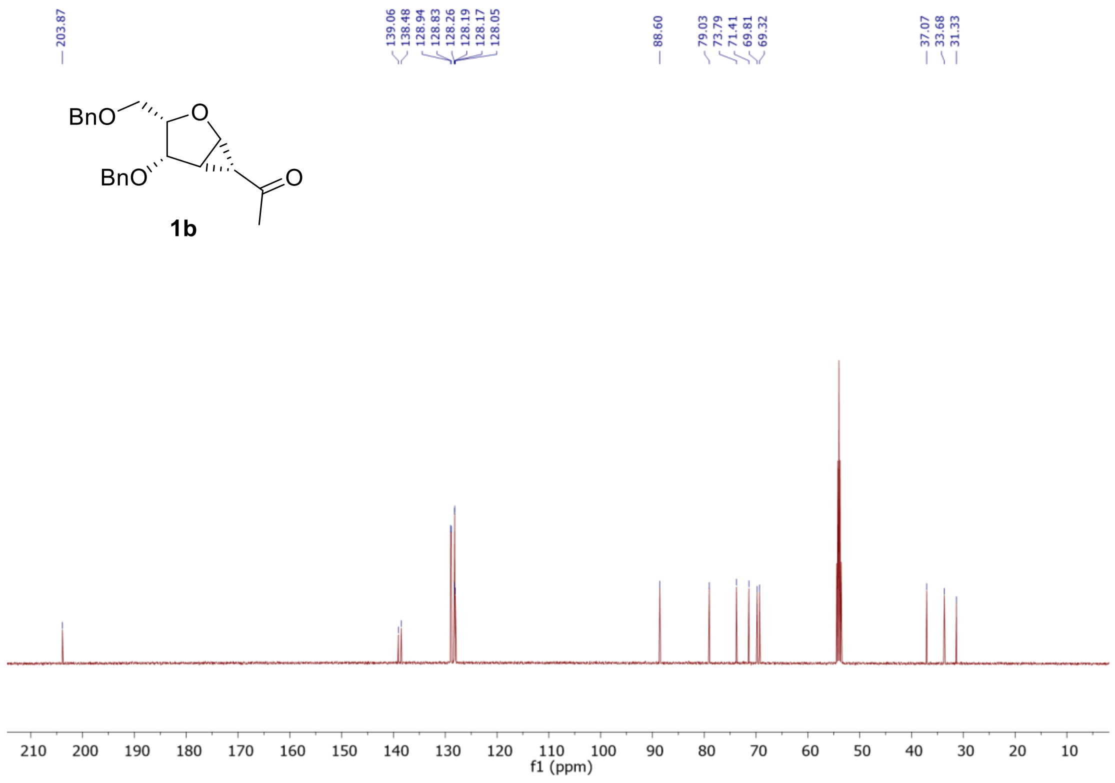

Supplementary Figure S37: ^{13}C NMR spectra for 1b

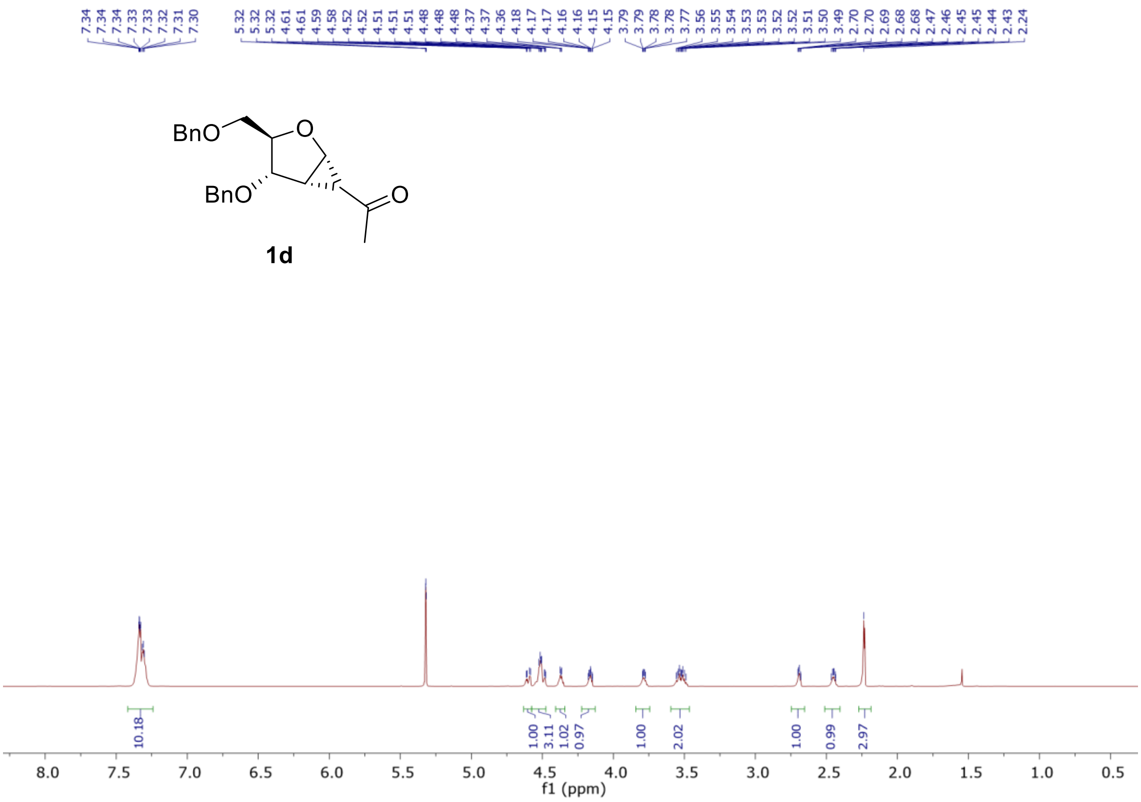

Supplementary Figure S38: ^{1}H NMR spectra for 1d

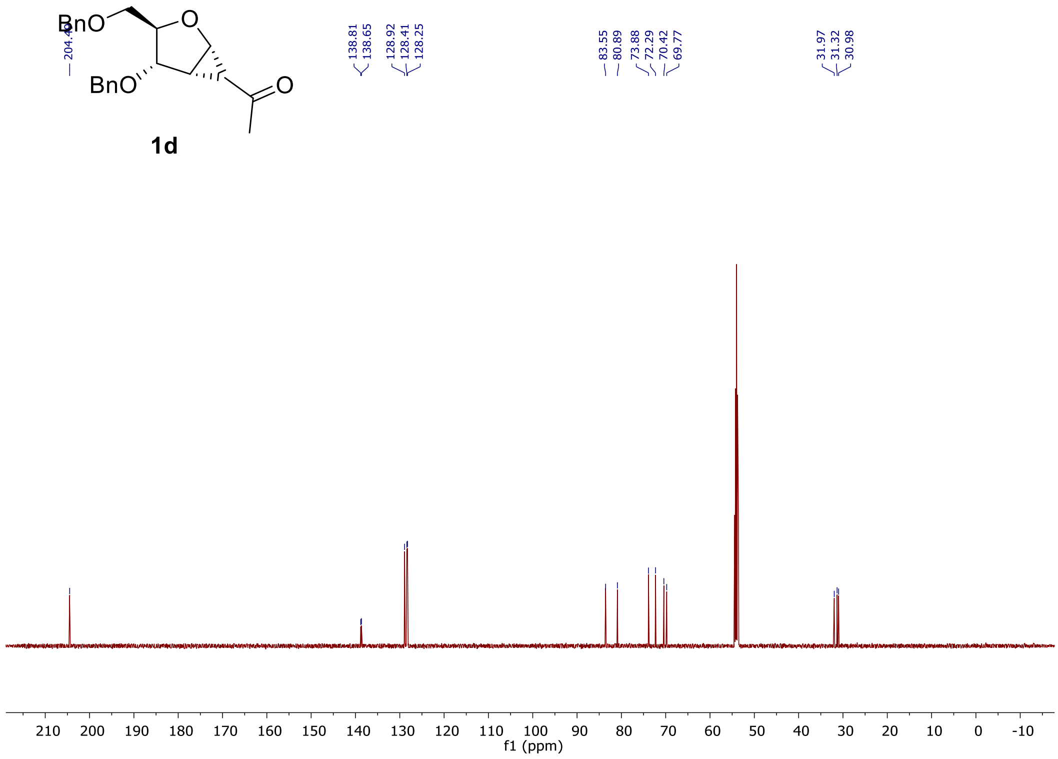

Supplementary Figure S39: ^{13}C NMR spectra for 1d

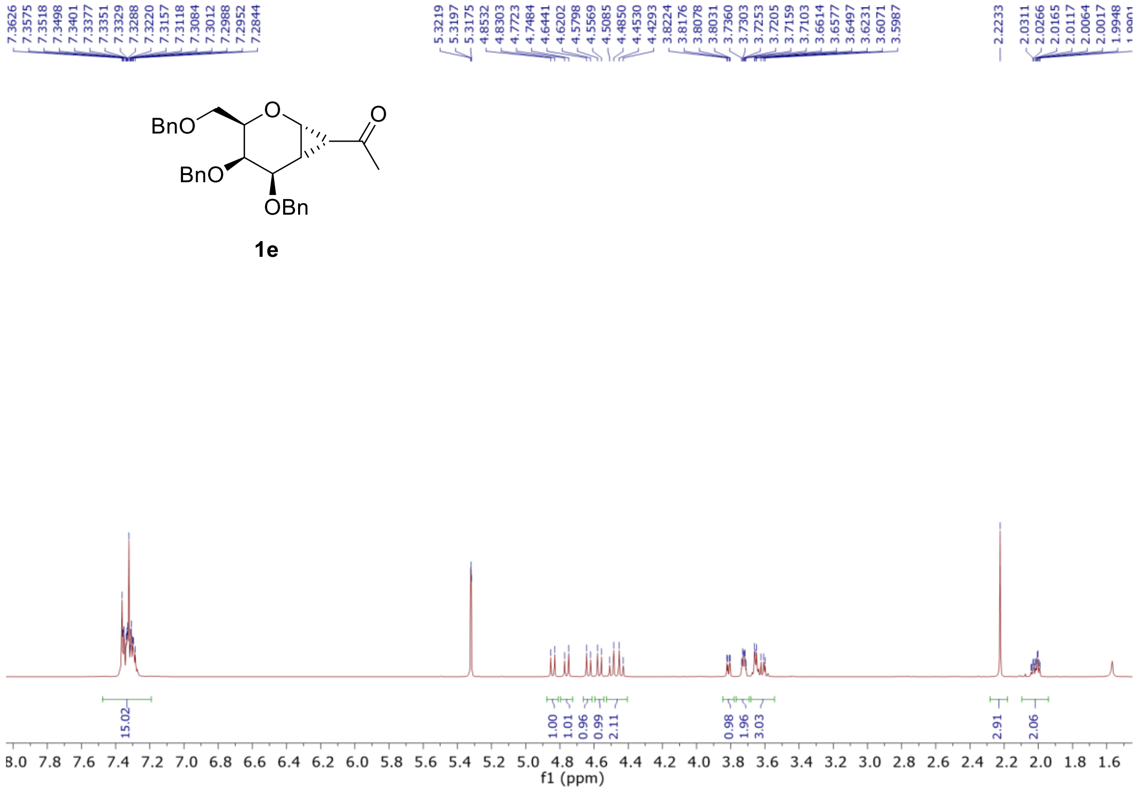

Supplementary Figure S40: ^{1}H NMR spectra for 1e

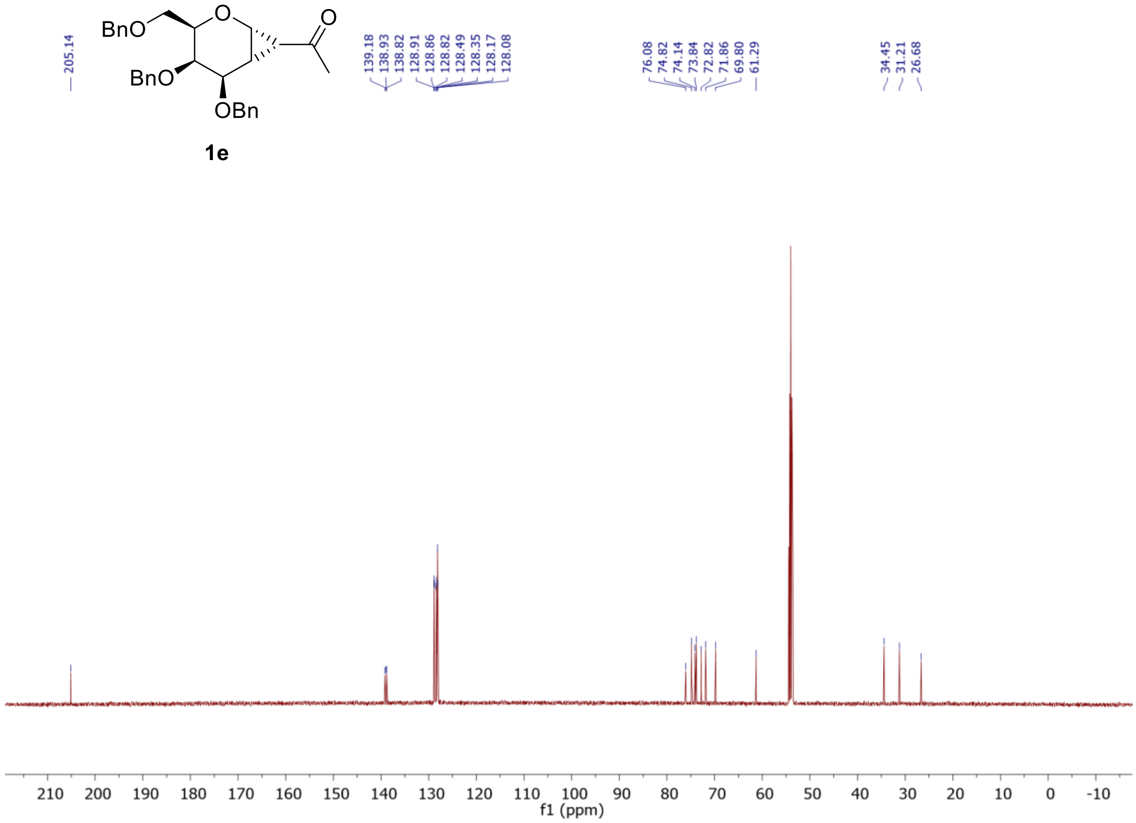

Supplementary Figure S41: ^{13}C NMR spectra for 1e

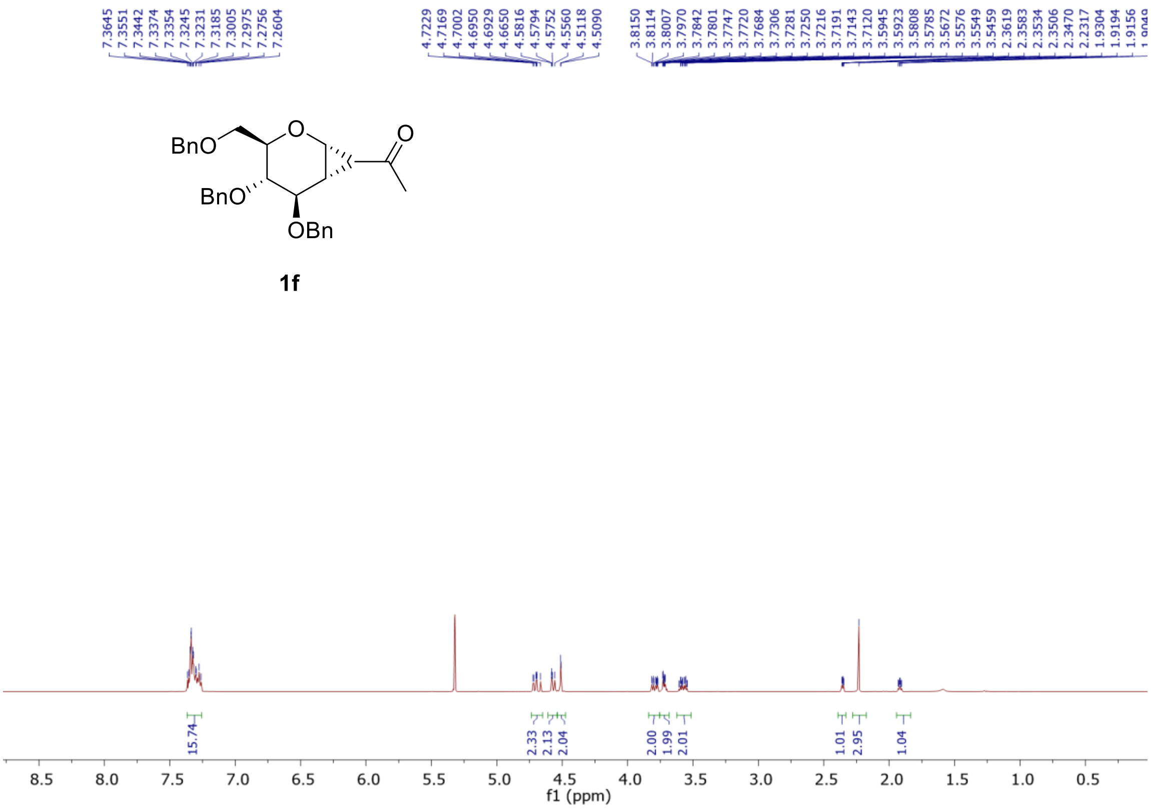

Supplementary Figure S42: ^{1}H NMR spectra for 1f

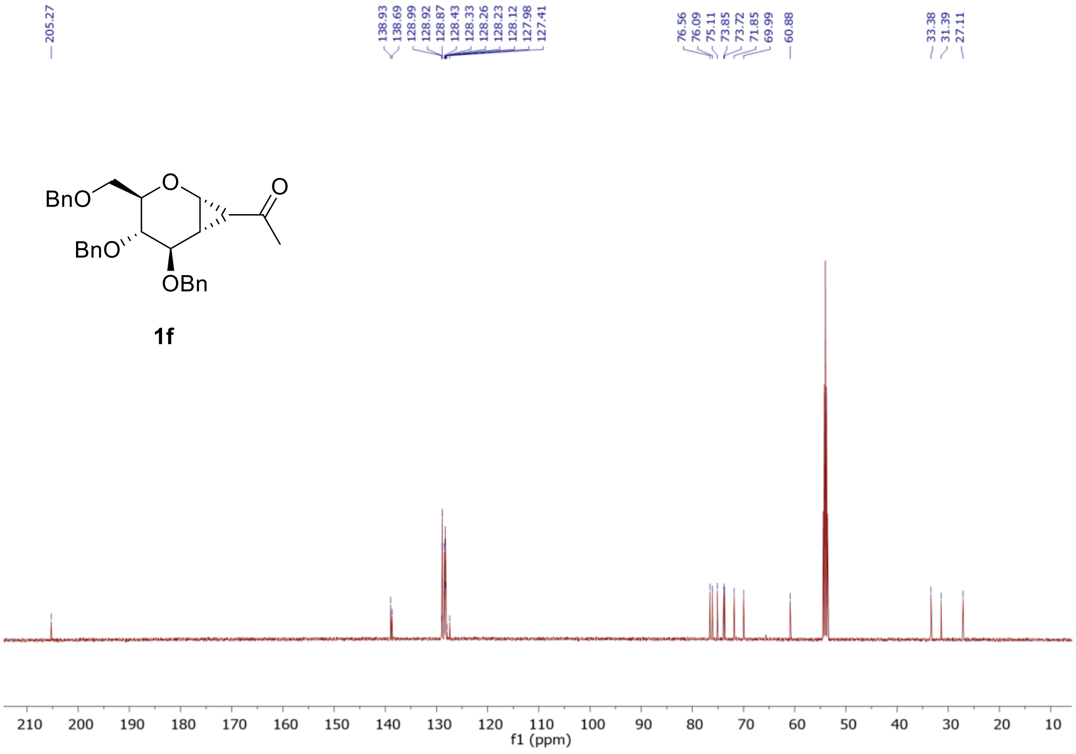

Supplementary Figure S43: ^{13}C NMR spectra for 1f

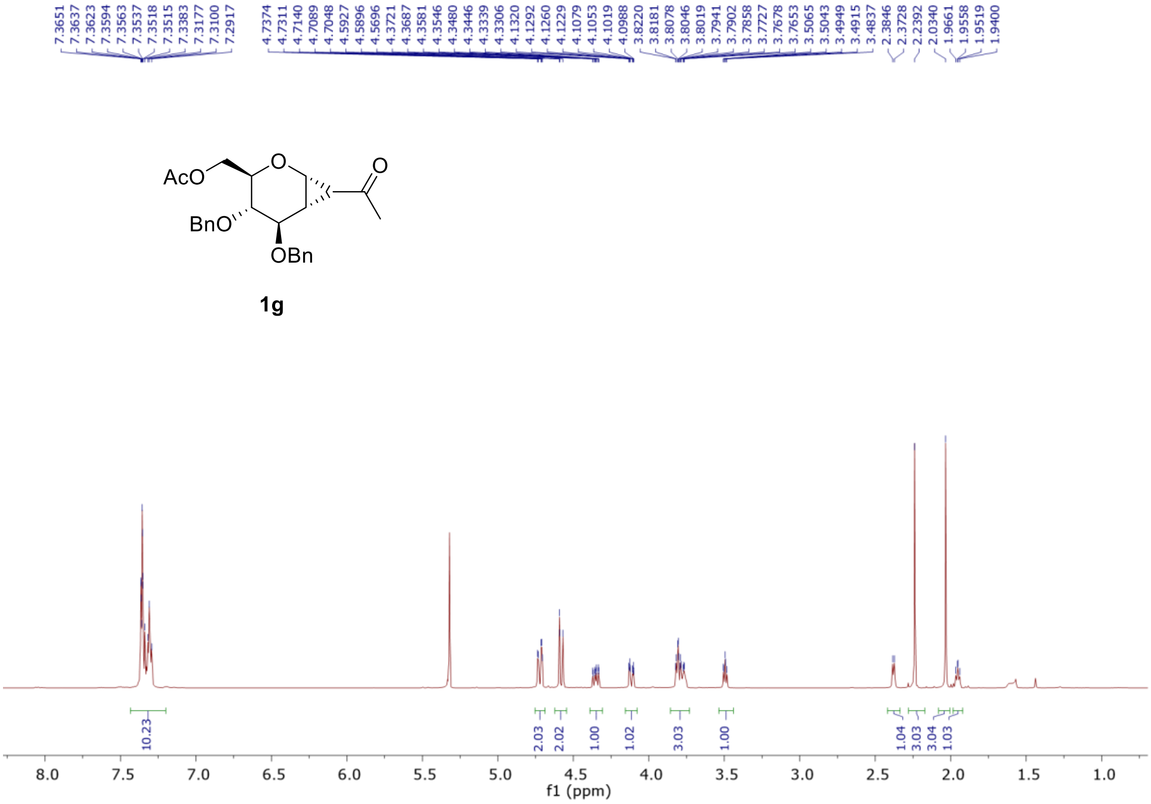

Supplementary Figure S44: ^{1}H NMR spectra for 1g

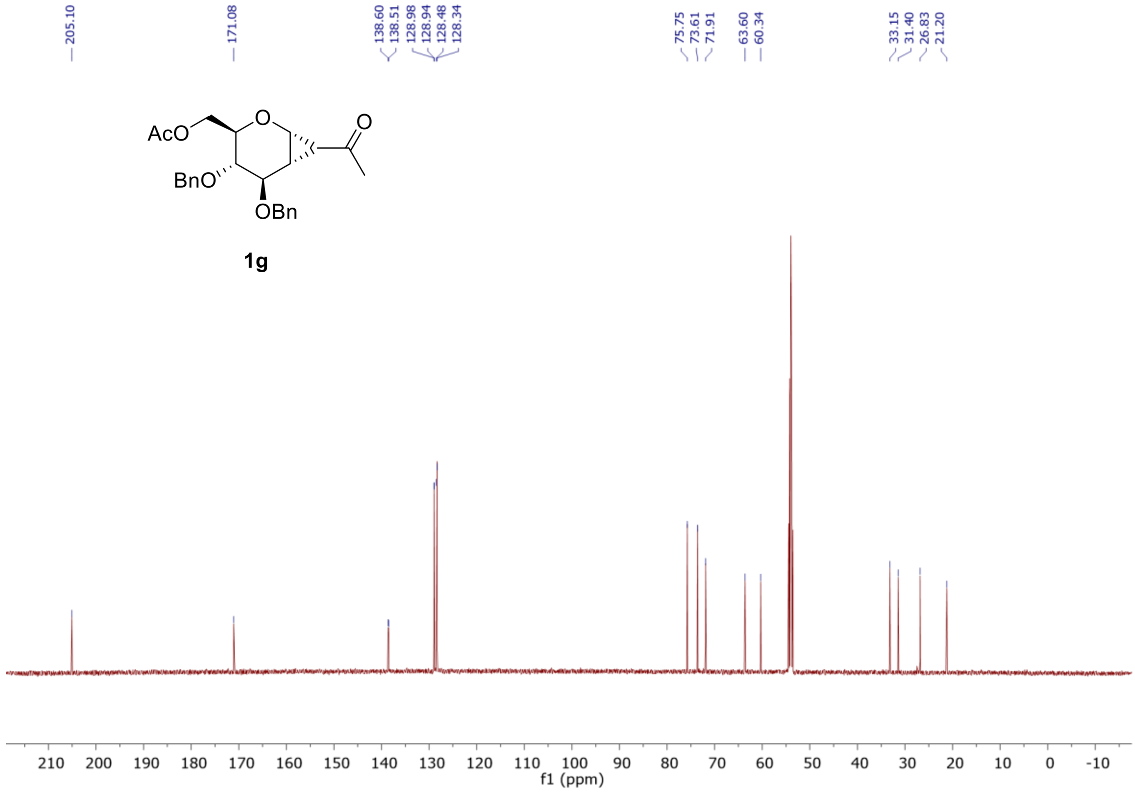

Supplementary Figure S45: ^{13}C NMR spectra for 1g

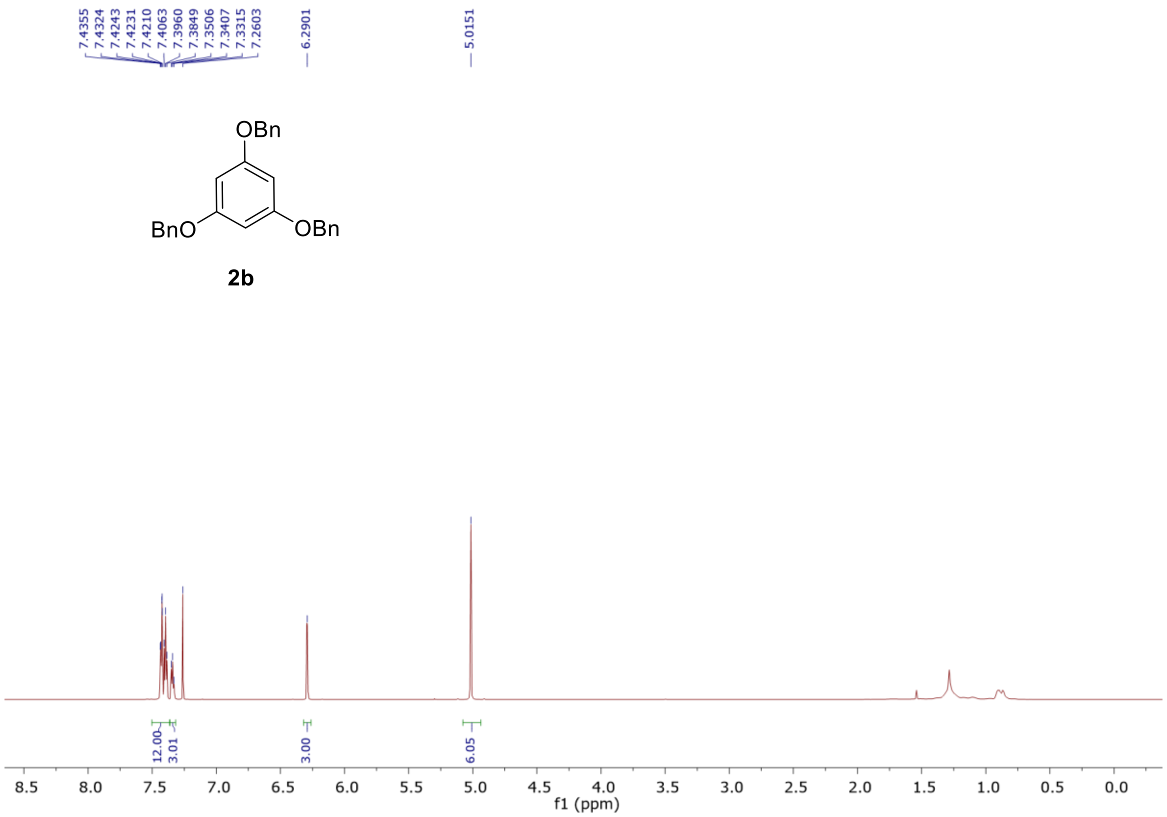

Supplementary Figure S46: ^{1}H NMR spectra for 2b

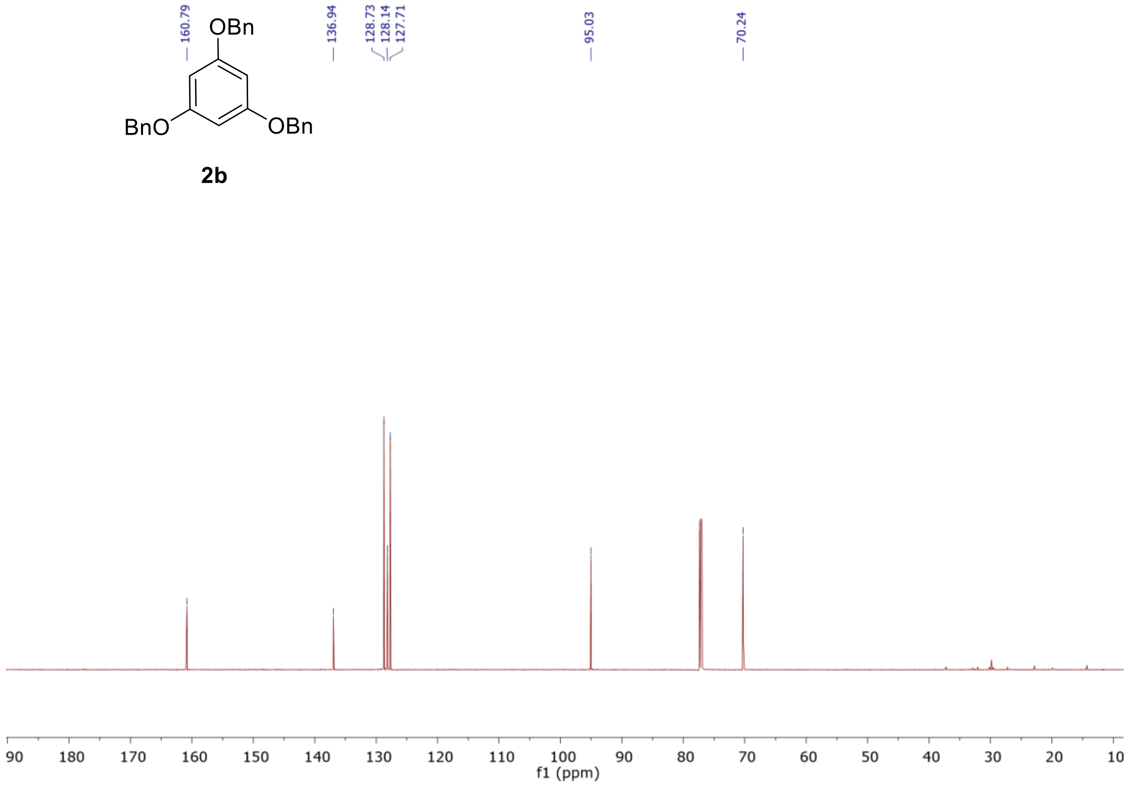

Supplementary Figure S47: ^{13}C NMR spectra for 2b

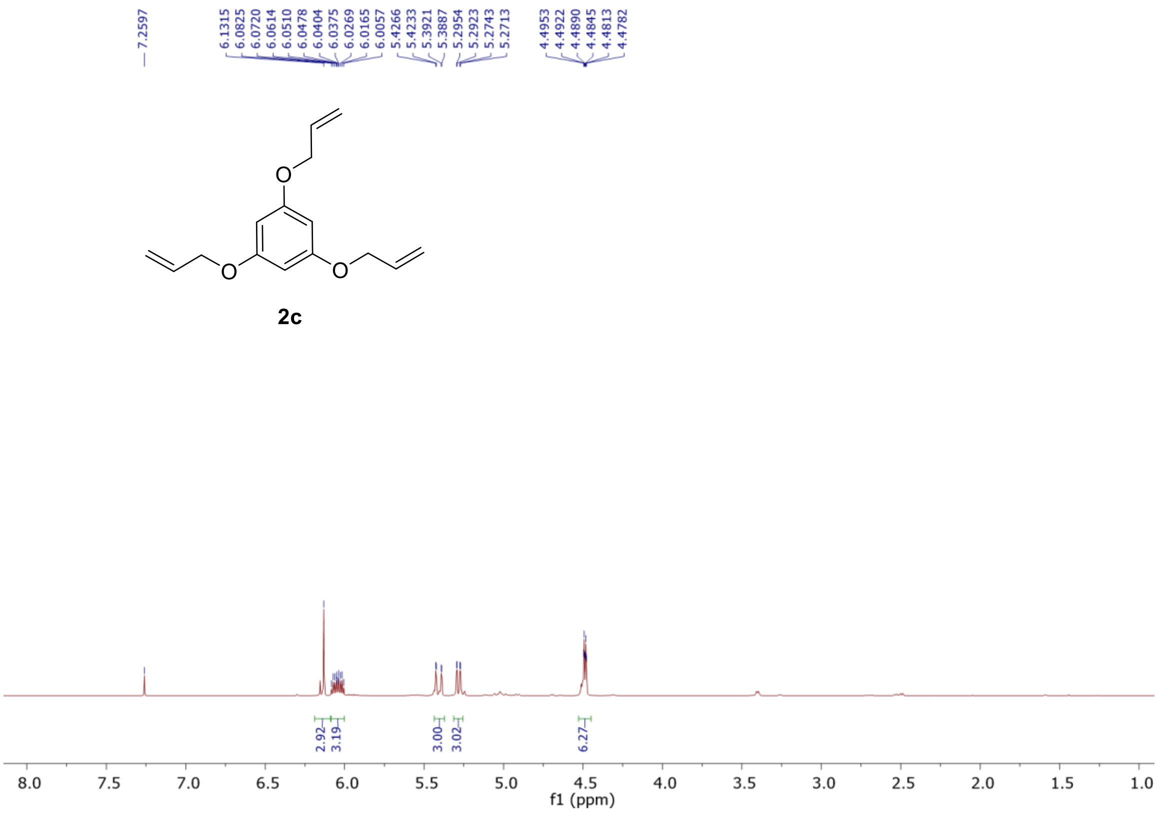

Supplementary Figure S48: ^{1}H NMR spectra for 2c

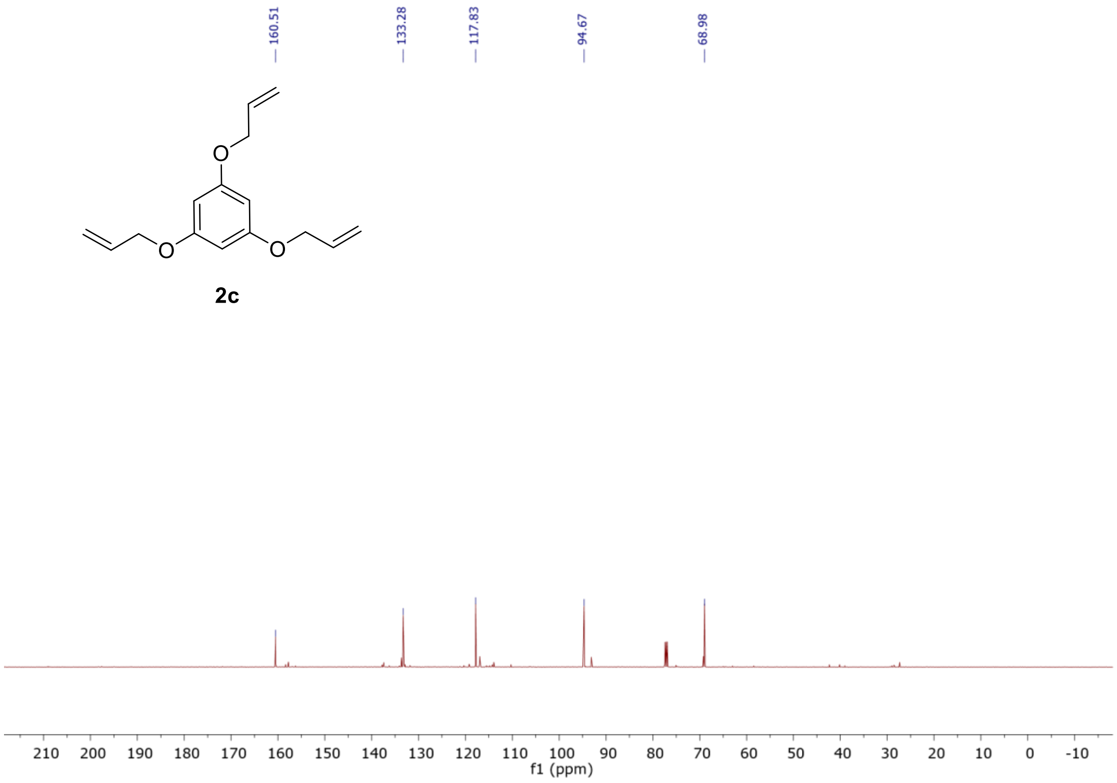

Supplementary Figure S49: ^{13}C NMR spectra for 2c

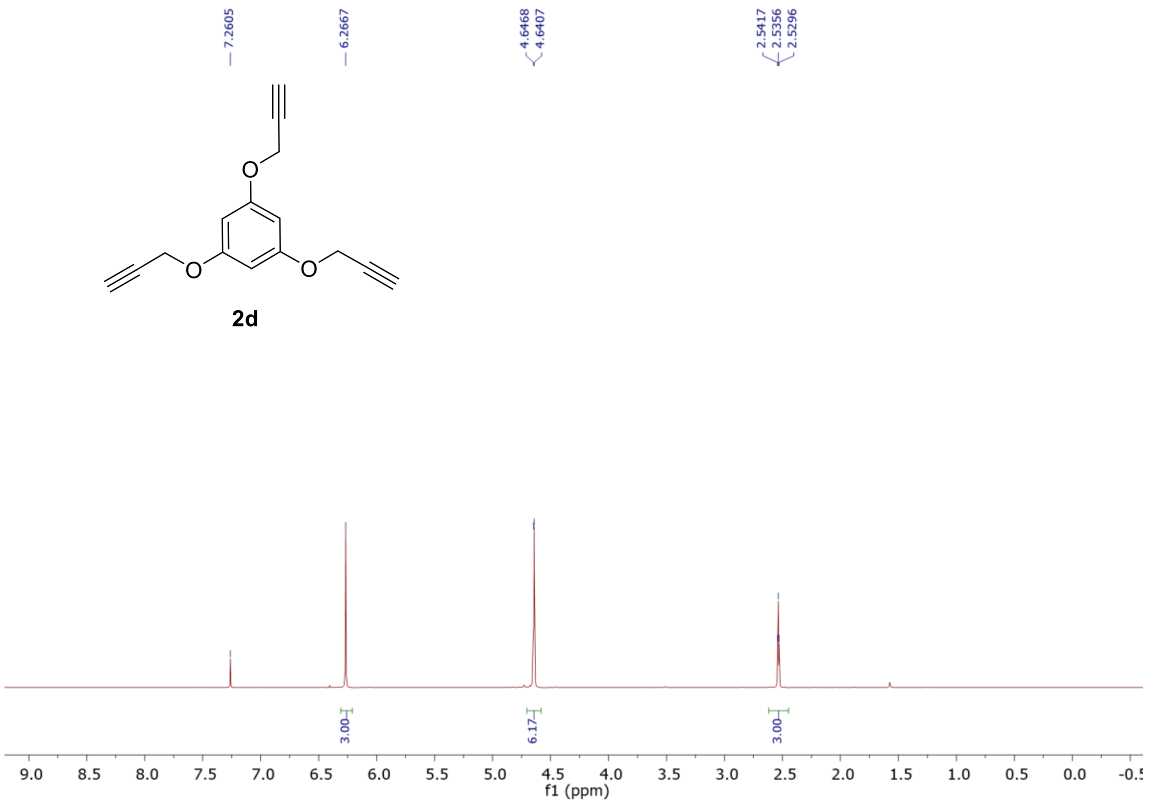

Supplementary Figure S50: ^{1}H NMR spectra for 2d

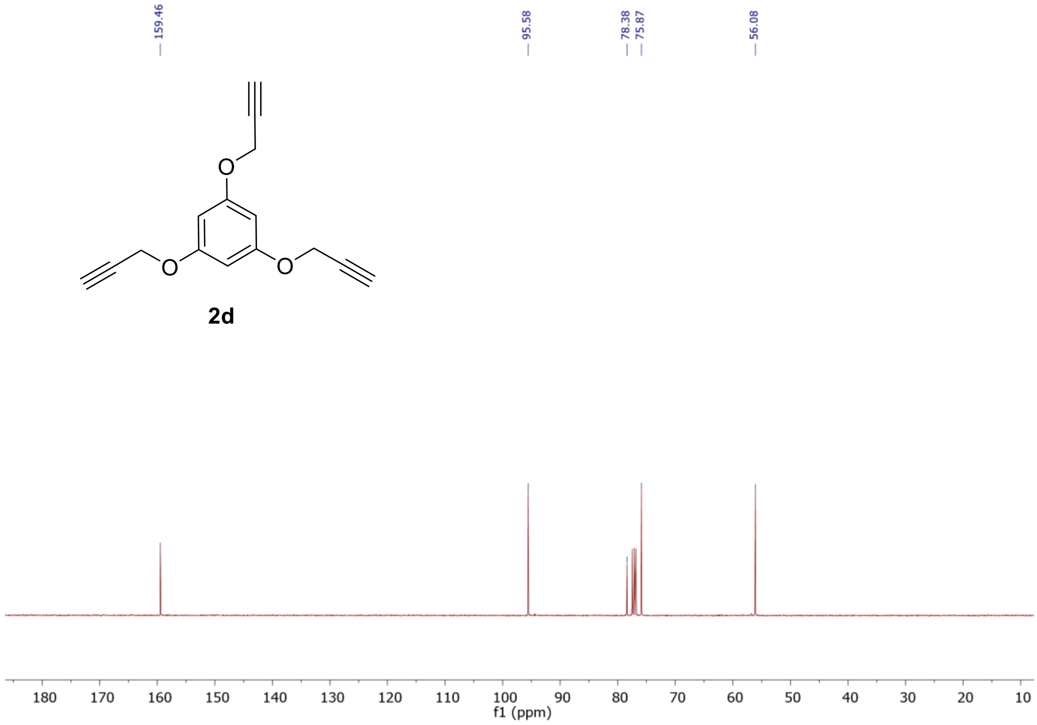

Supplementary Figure S51: ^{13}C NMR spectra for 2d

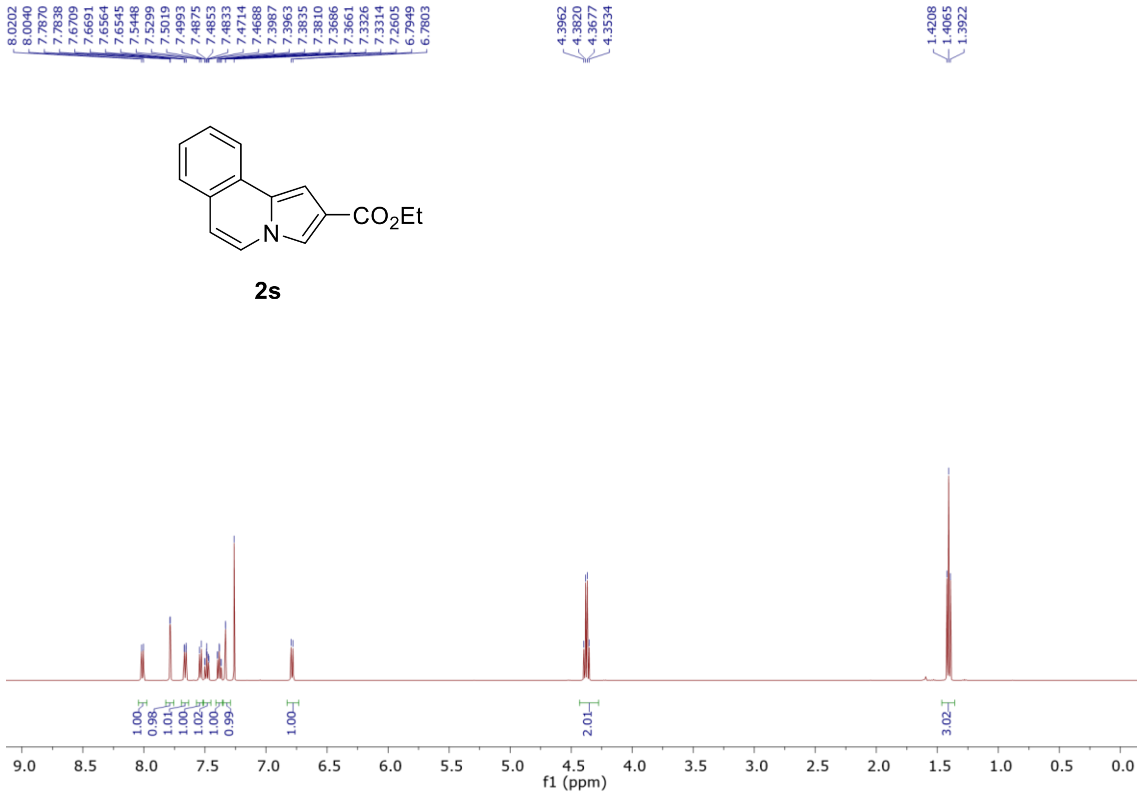

Supplementary Figure S52: ^{1}H NMR spectra for 2s

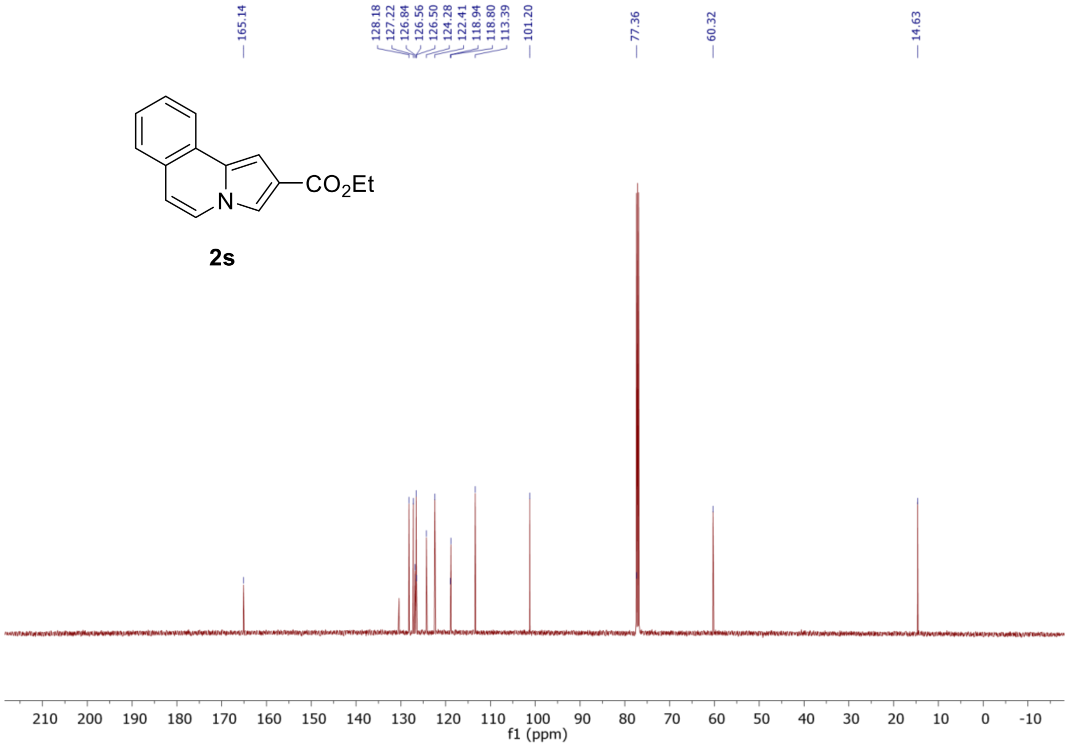

Supplementary Figure S53: ^{13}C NMR spectra for 2s

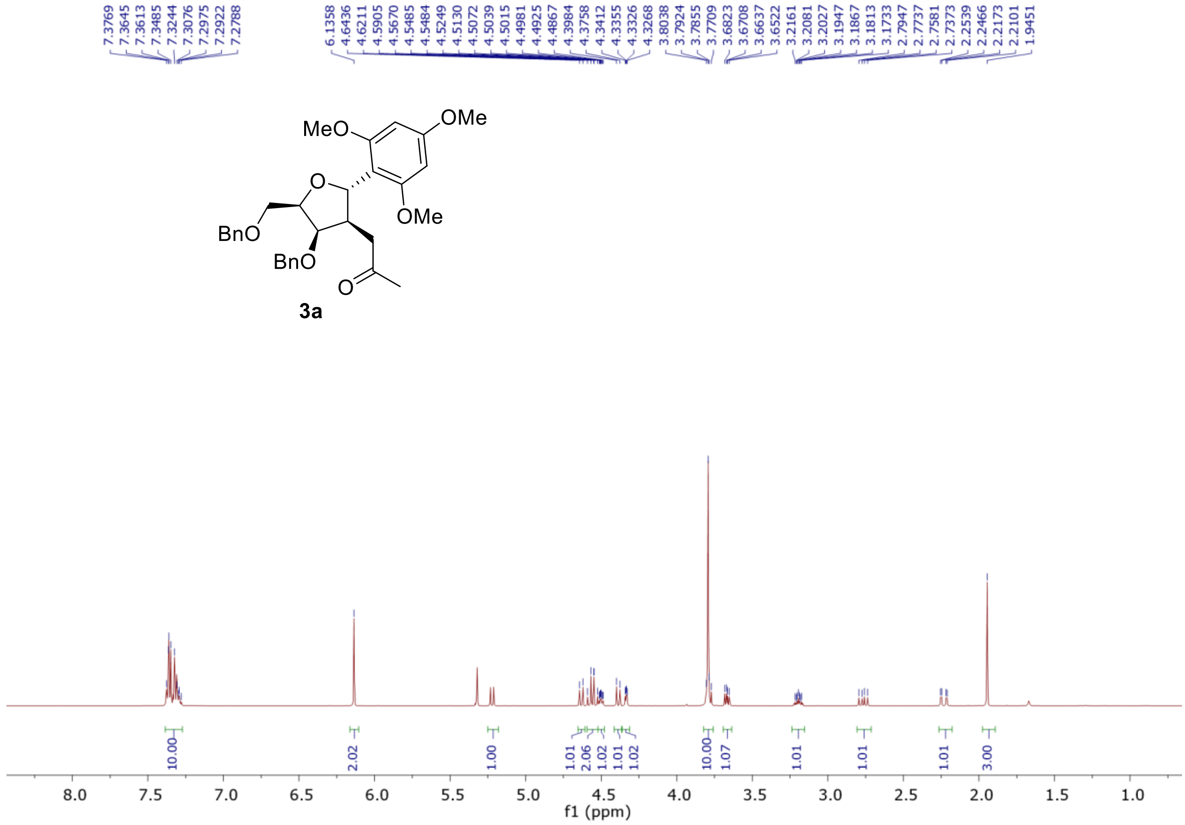

Supplementary Figure S54: ^{1}H NMR spectra for 3a

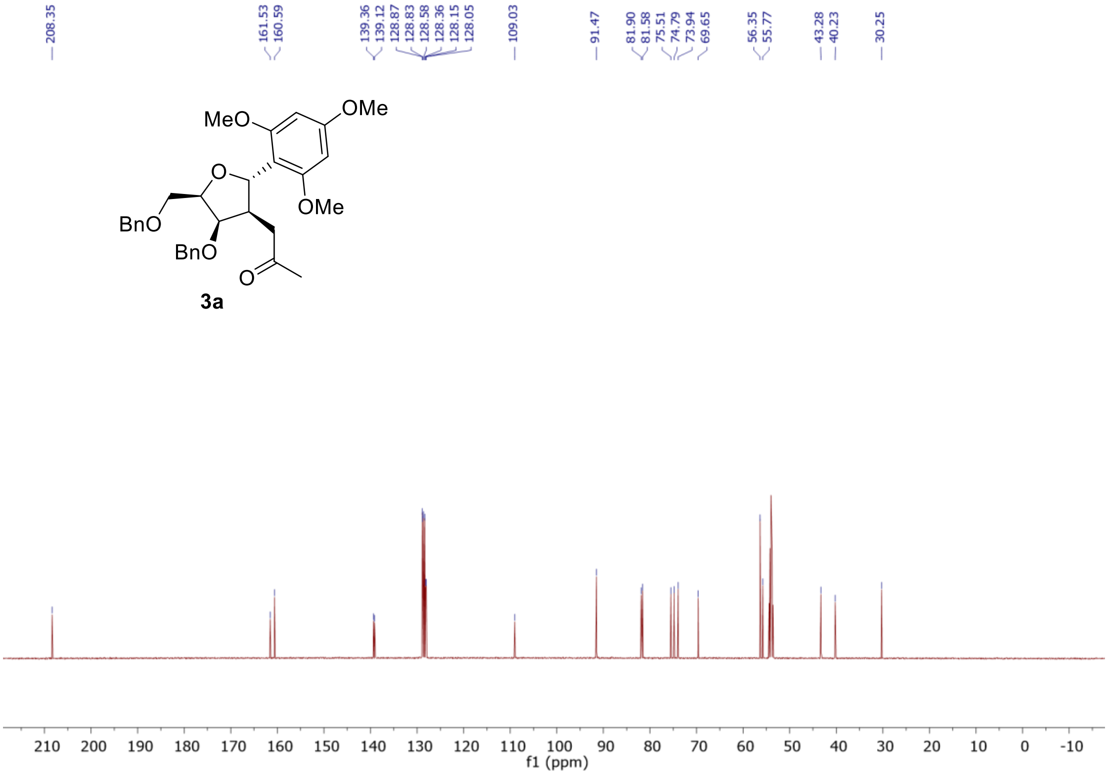

Supplementary Figure S55: ^{13}C NMR spectra for 3a

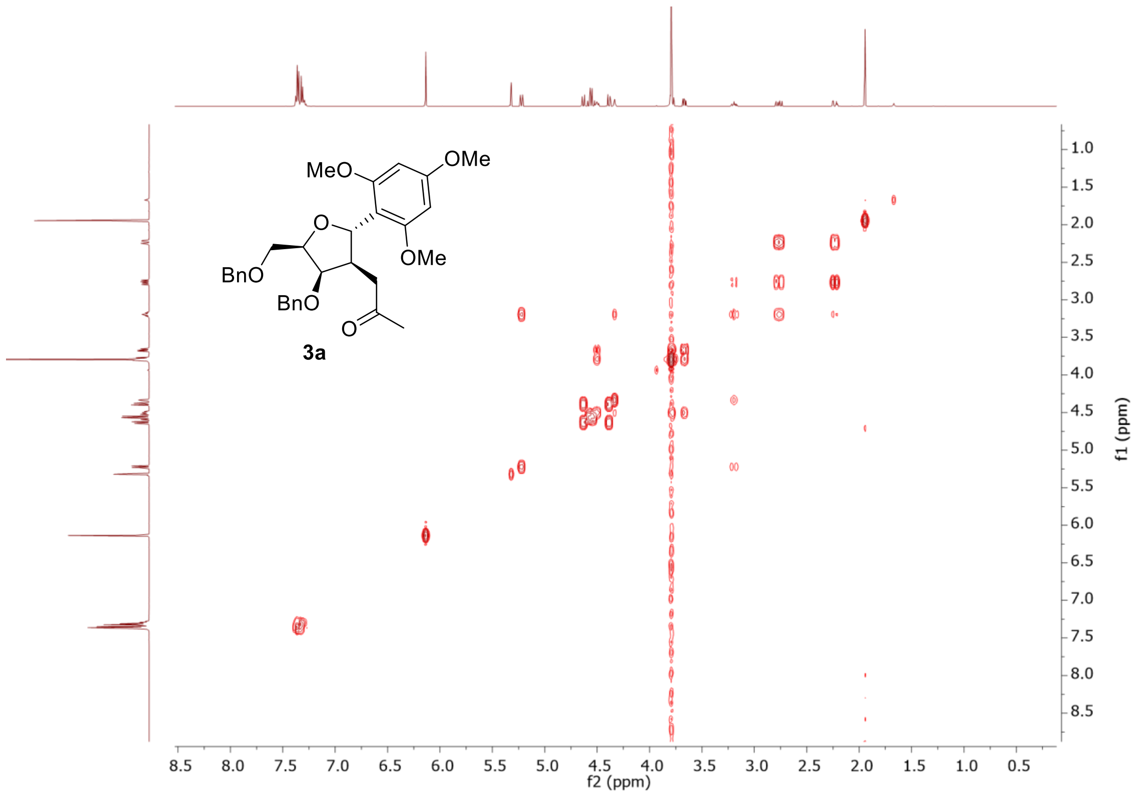

Supplementary Figure S56: COSY spectra for 3a

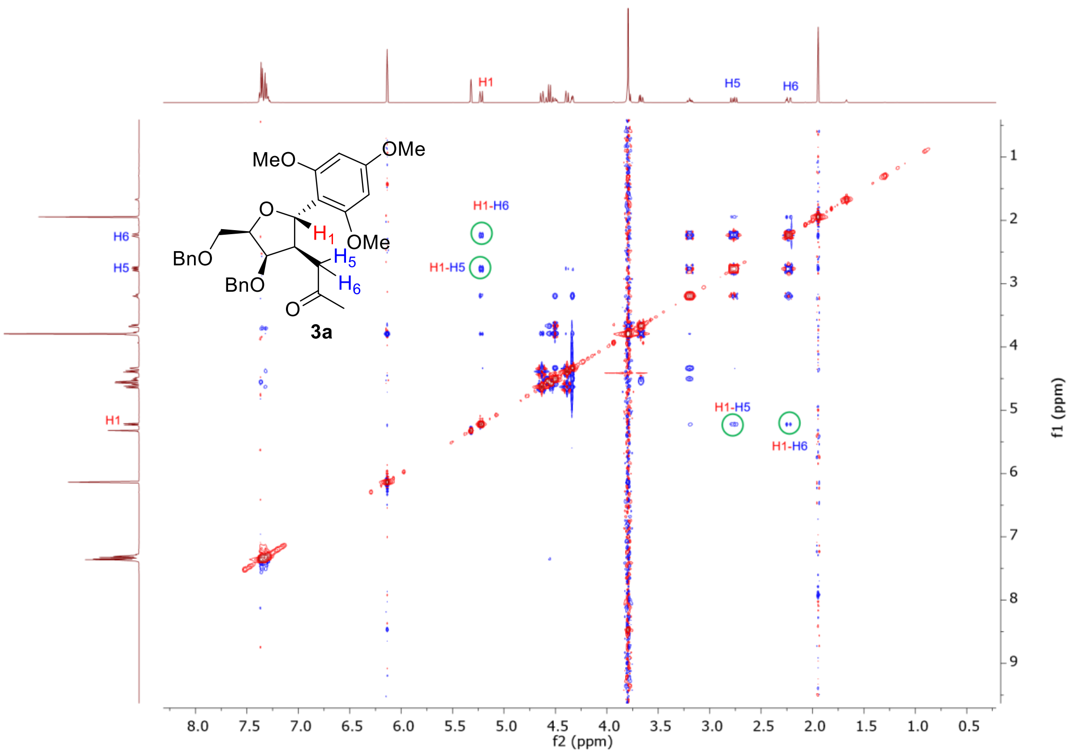

Supplementary Figure S57: NOESY spectra for 3a

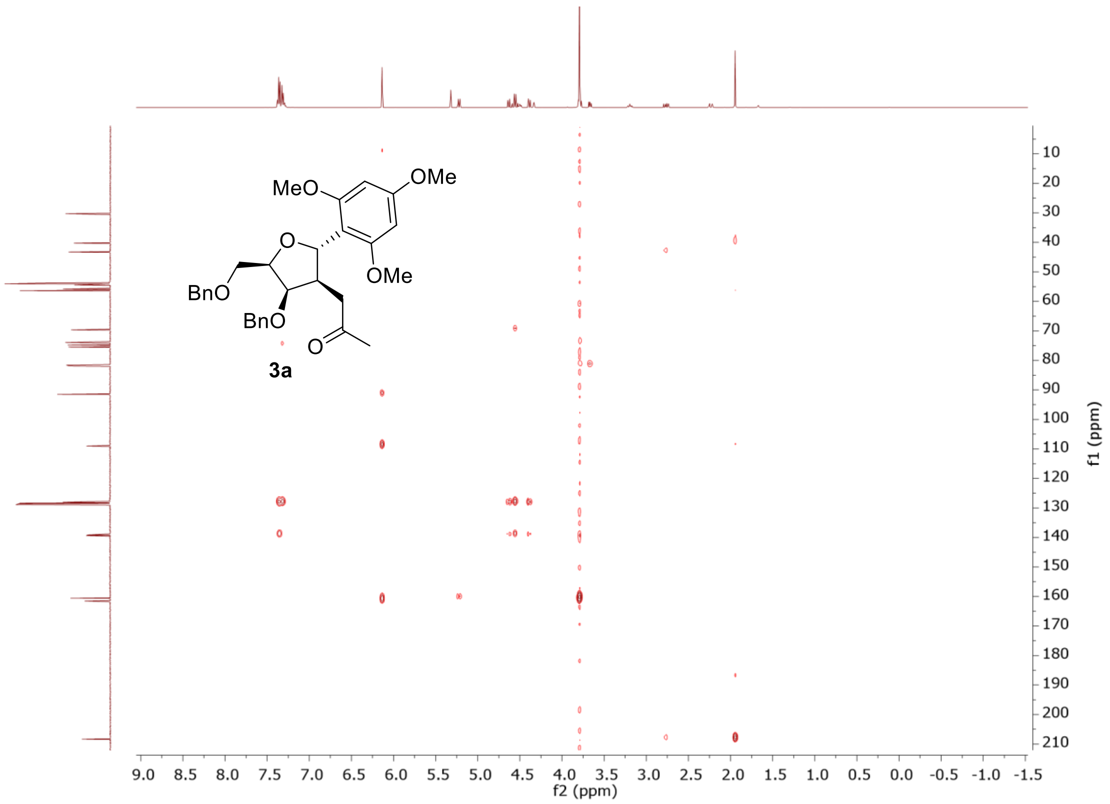

Supplementary Figure S58: HMBC spectra for 3a

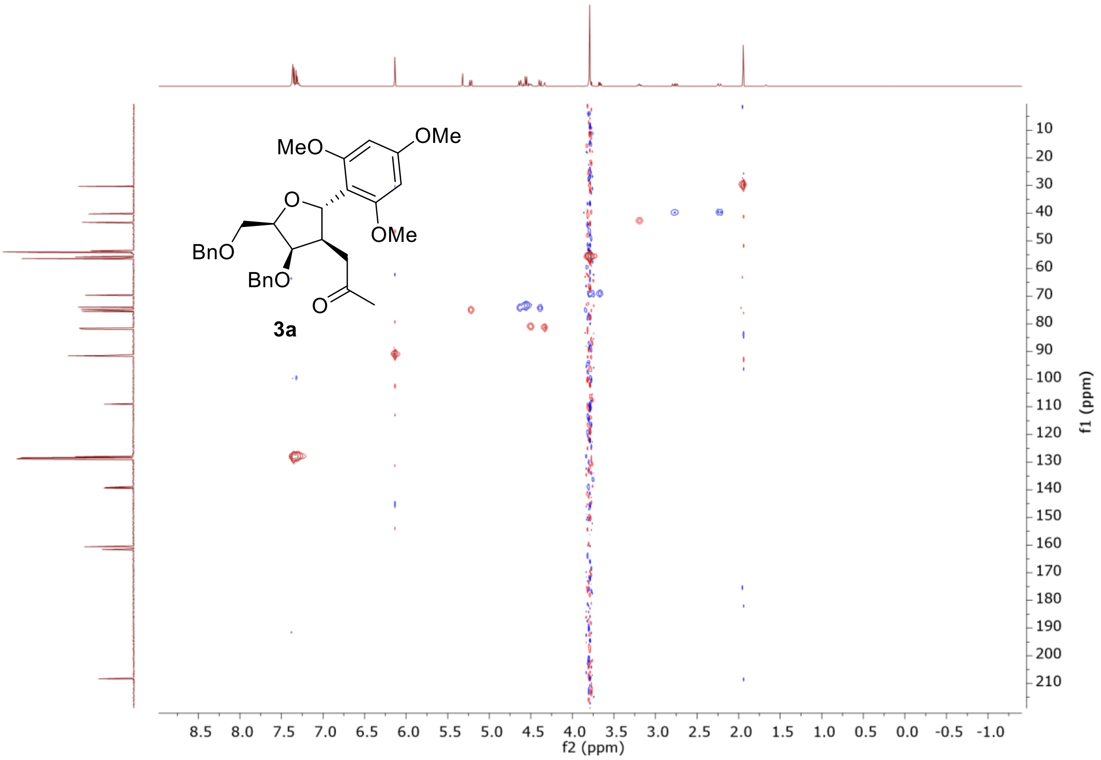

Supplementary Figure S59: HSQC spectra for 3a

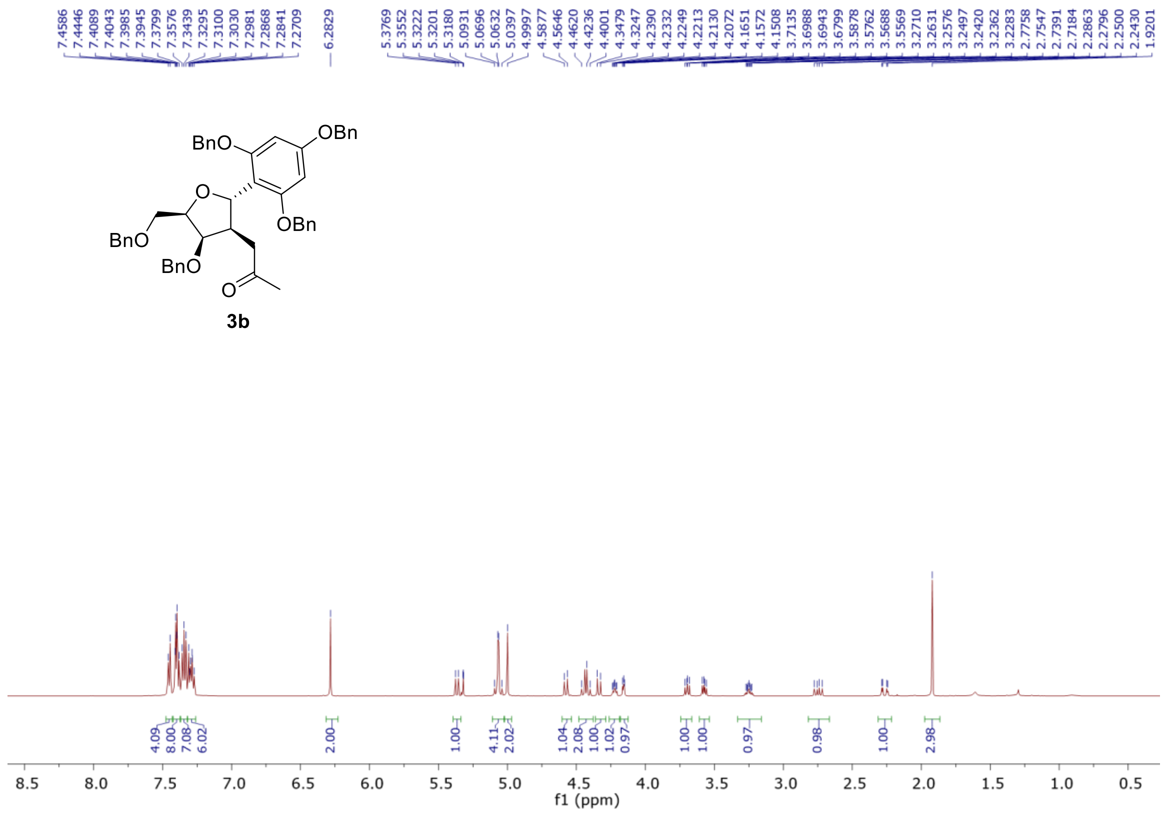

Supplementary Figure S60: ^{1}H NMR spectra for 3b

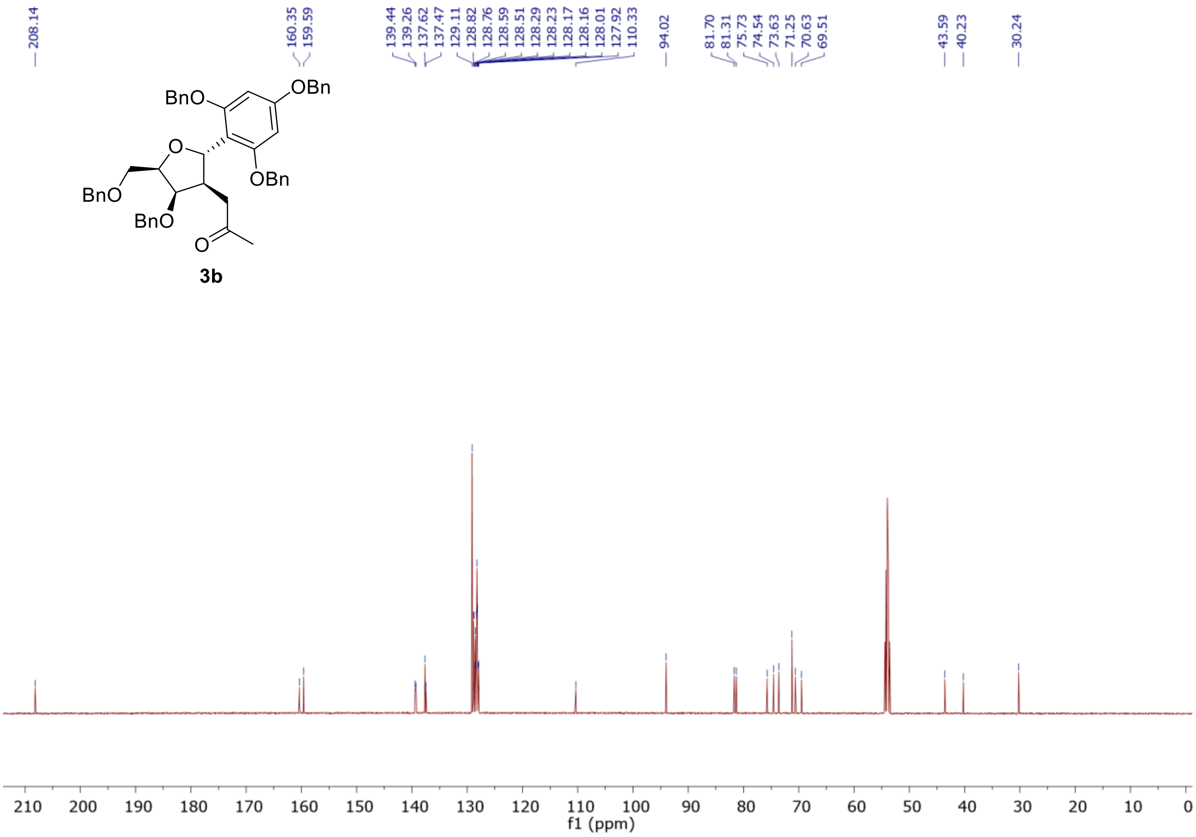

Supplementary Figure S61: ^{13}C NMR spectra for 3b

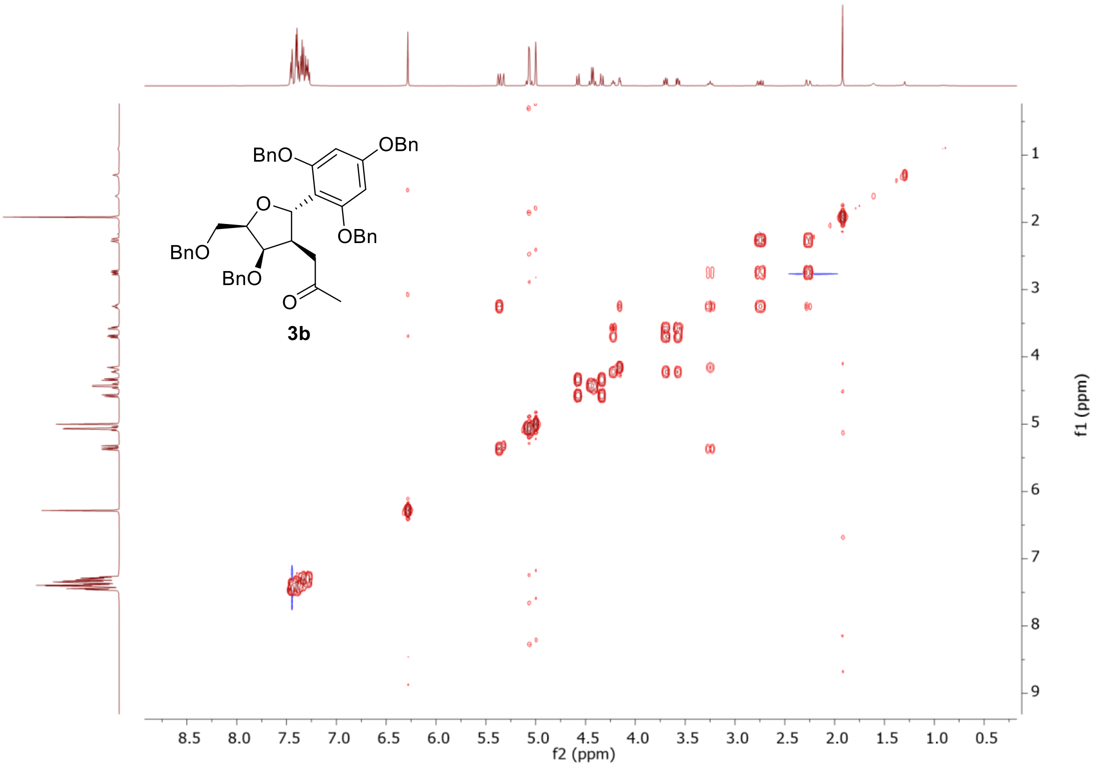

Supplementary Figure S62: COSY spectra for 3b

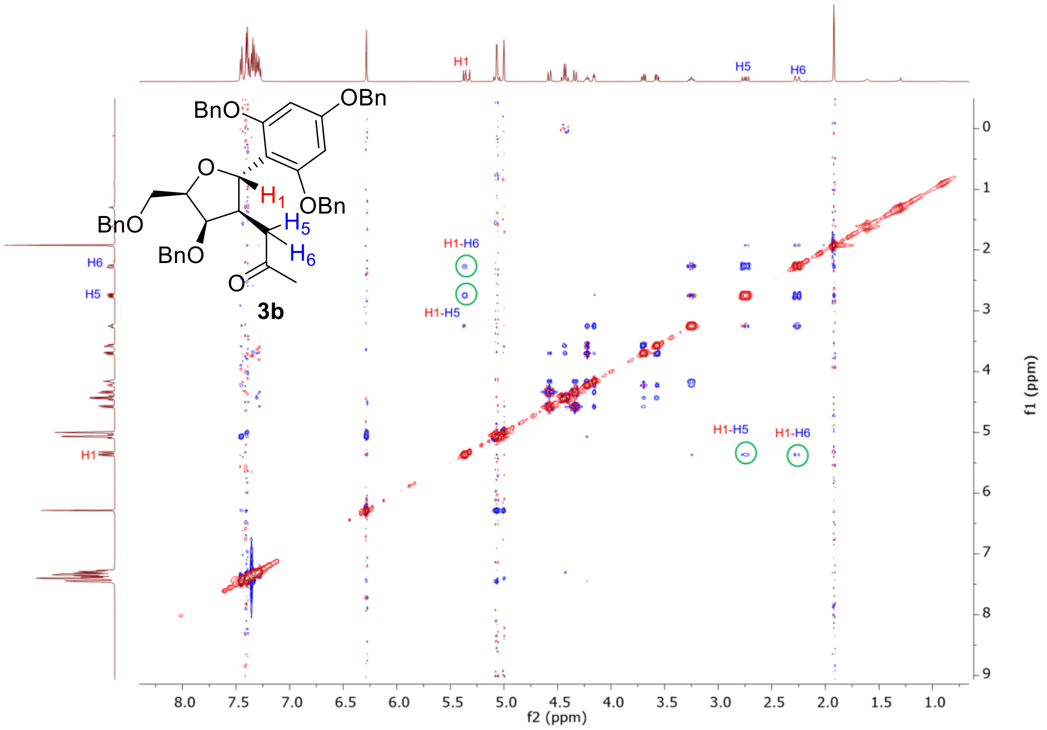

Supplementary Figure S63: NOESY spectra for 3b

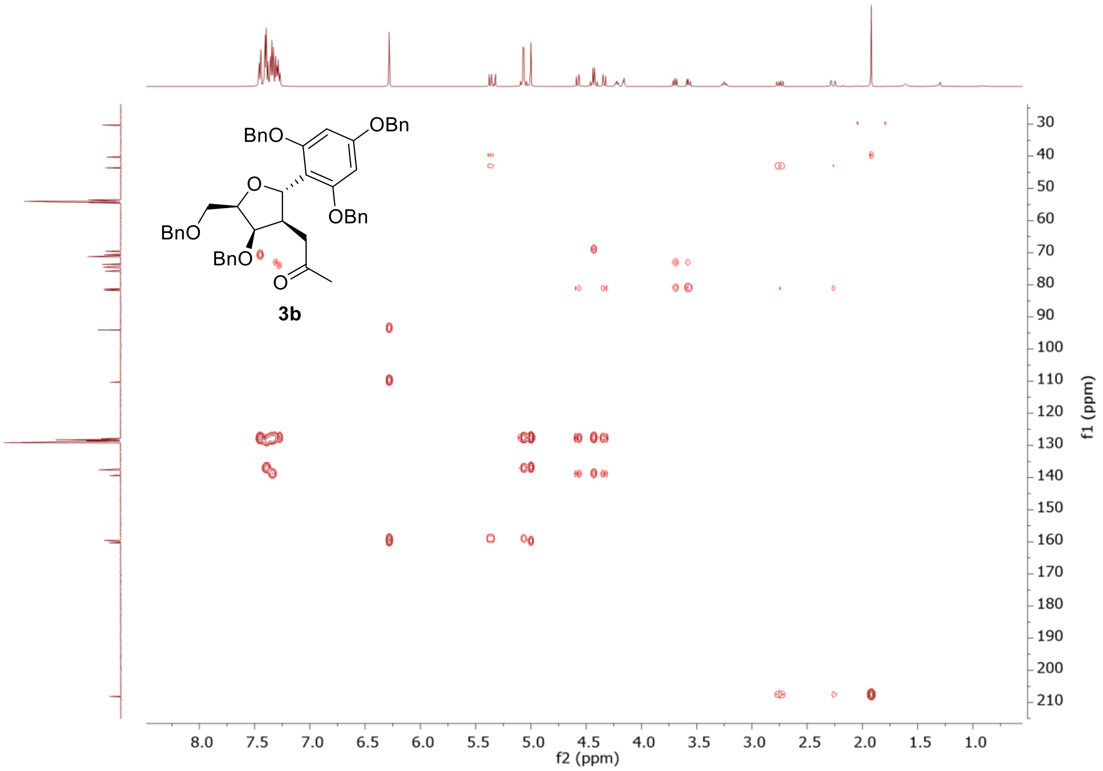

Supplementary Figure S64: HMBC spectra for 3b

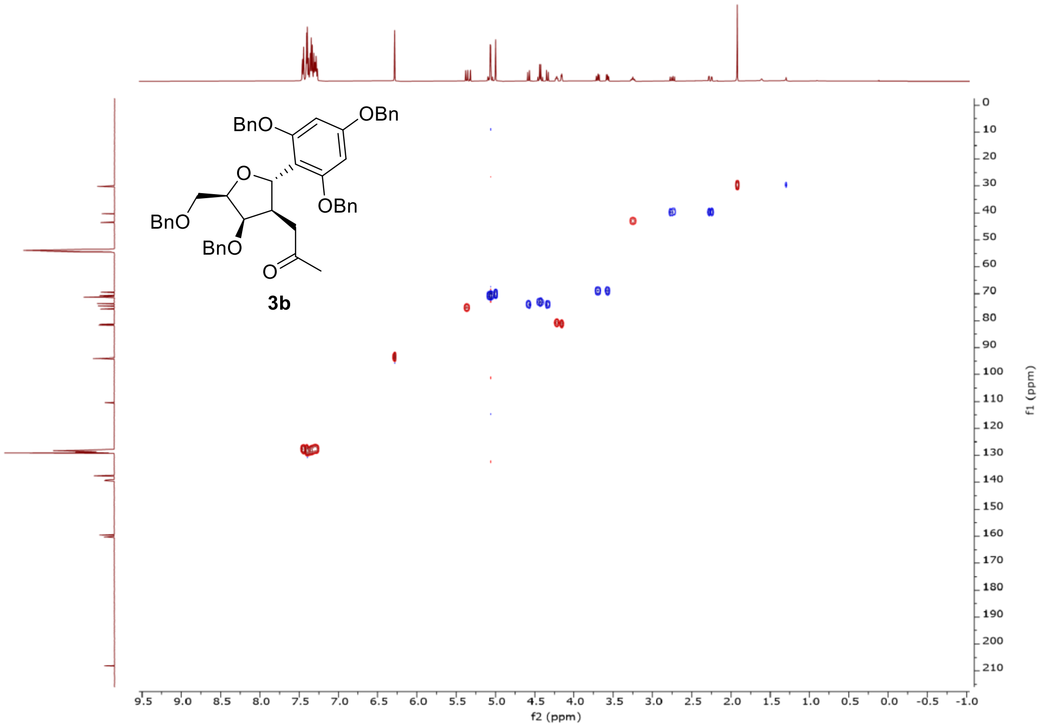

Supplementary Figure S65: HSQC spectra for 3b

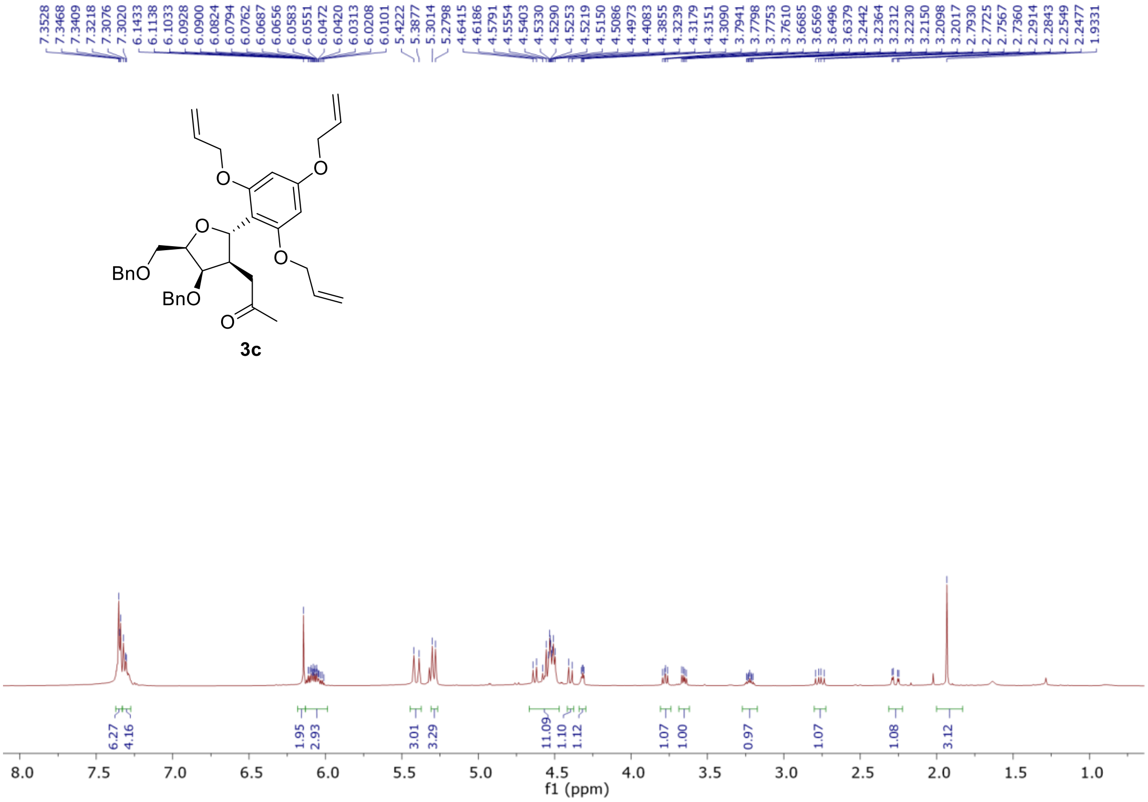

Supplementary Figure S66: ^{1}H NMR spectra for 3c

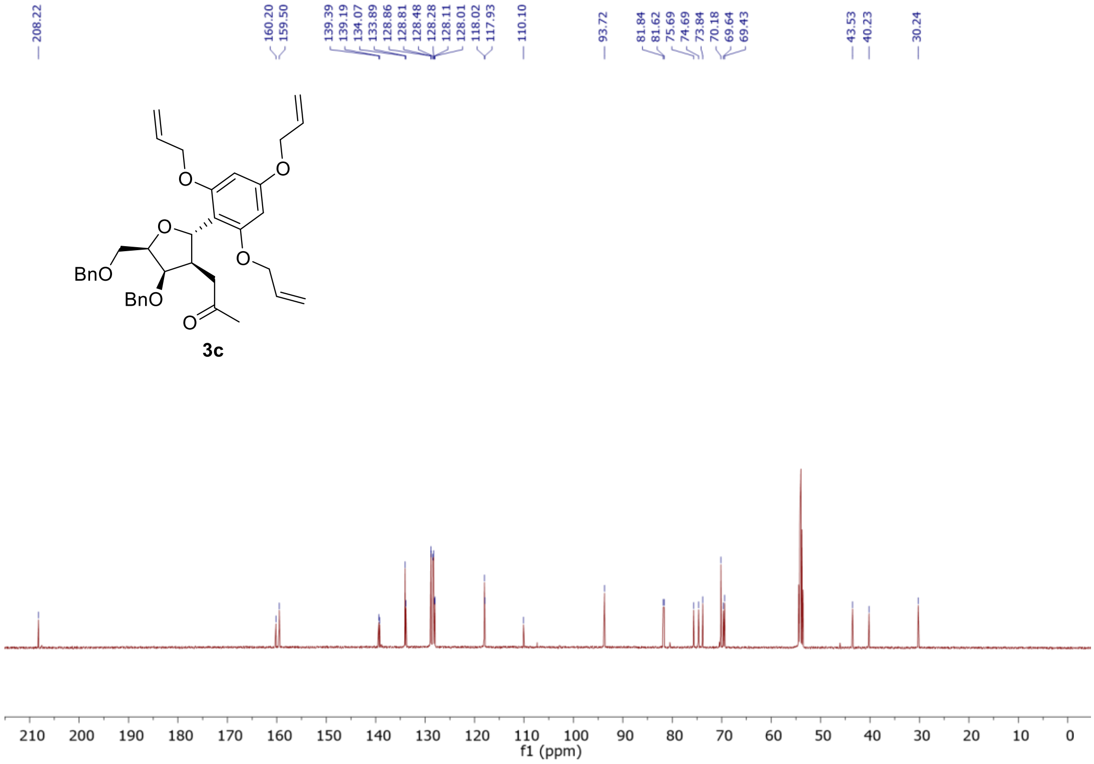

Supplementary Figure S67: ^{13}C NMR spectra for 3c

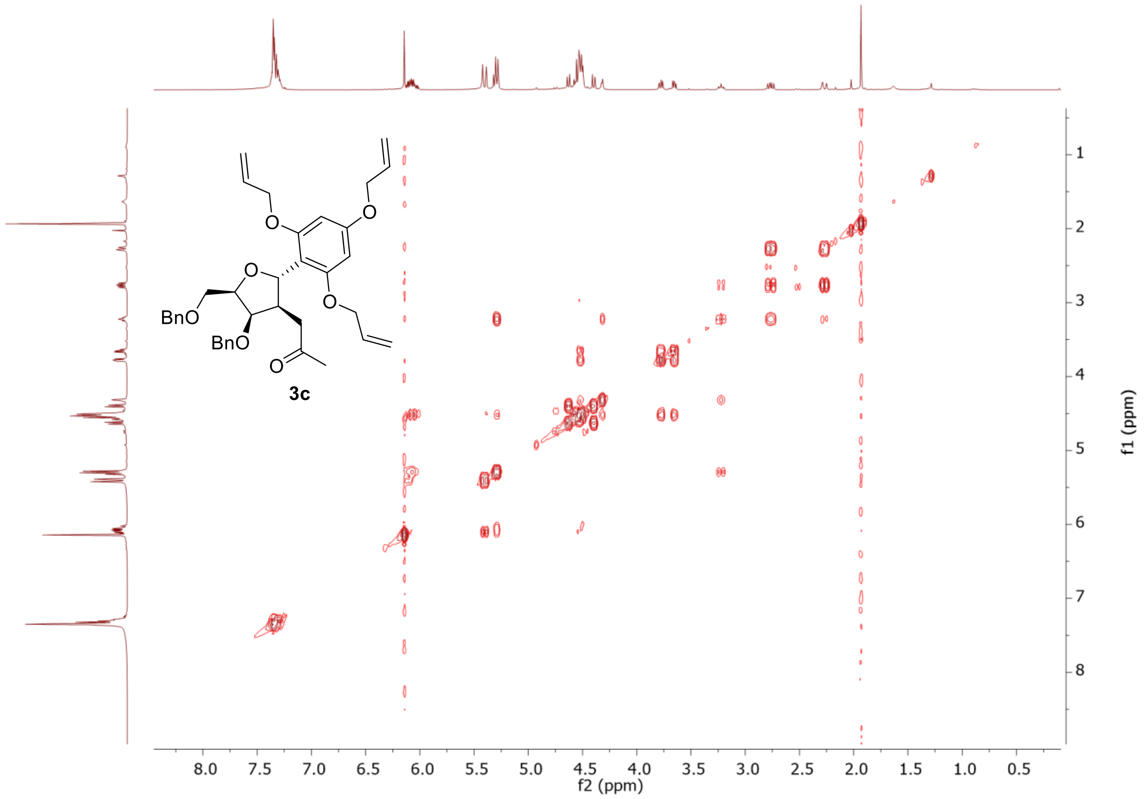

Supplementary Figure S68: COSY spectra for 3c

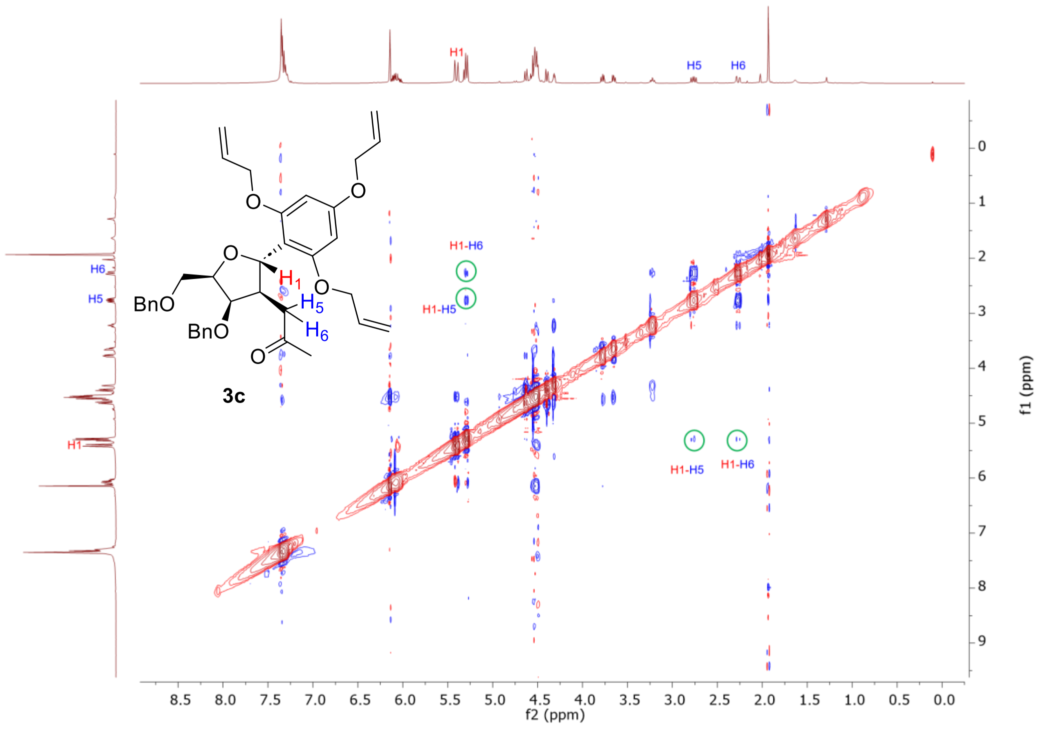

Supplementary Figure S69: NOESY spectra for 3c

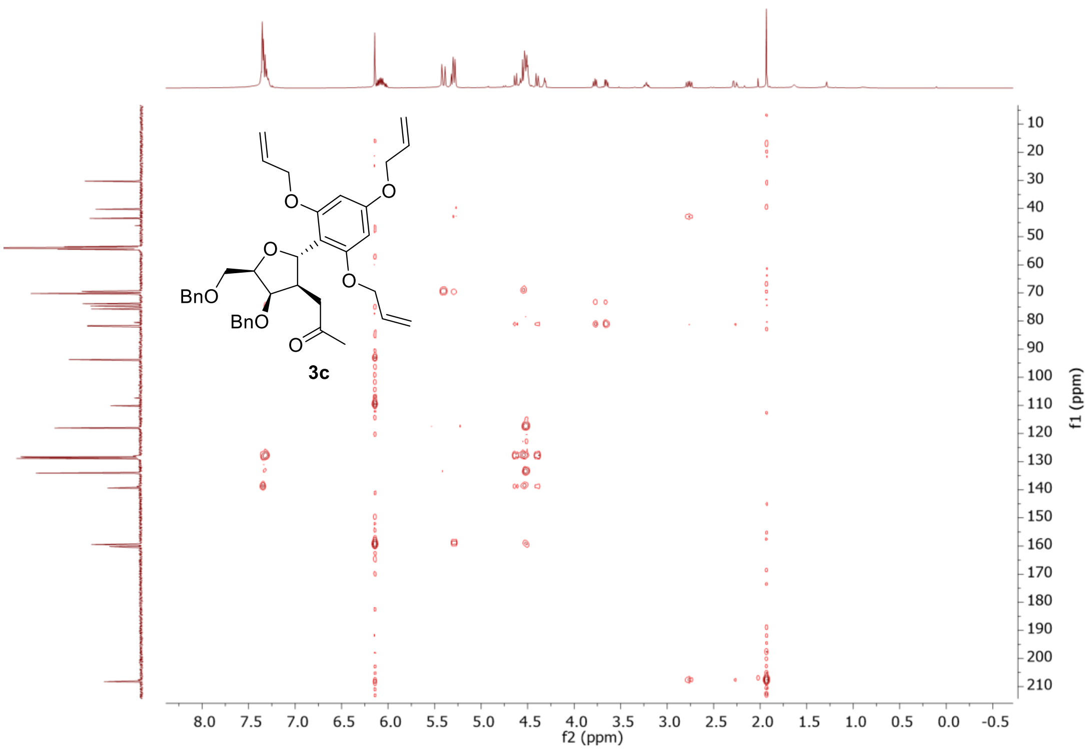

Supplementary Figure S70: HMBC spectra for 3c

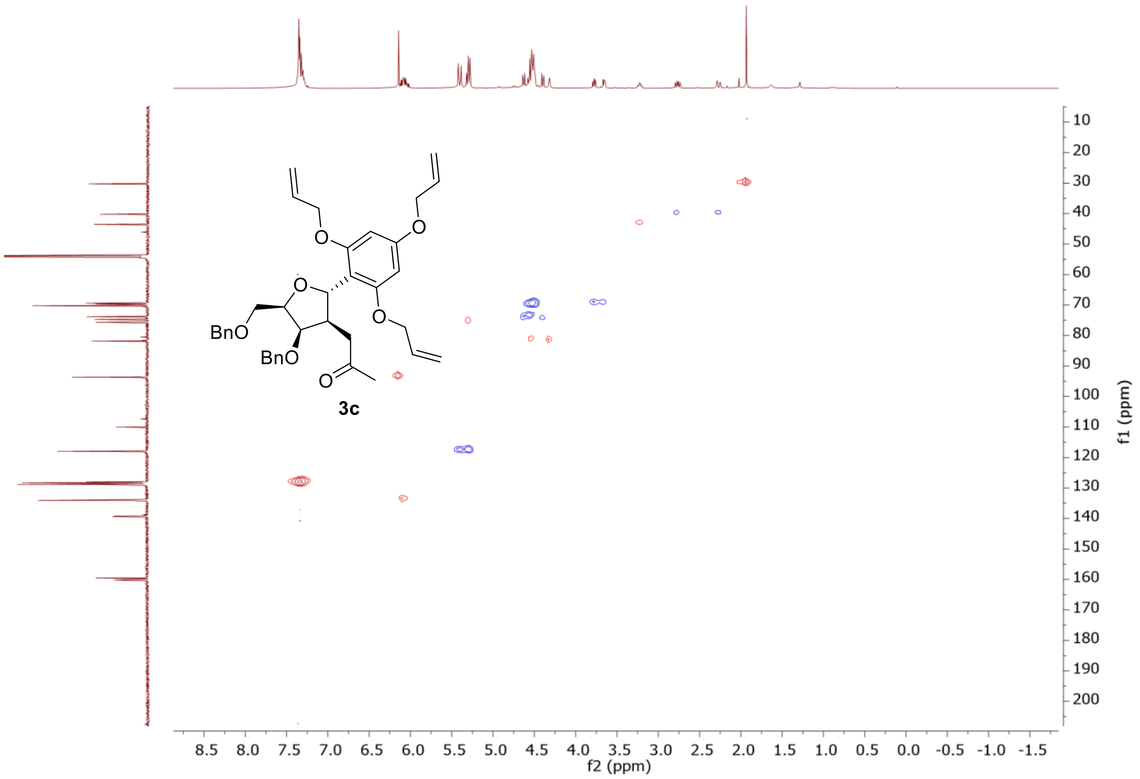

Supplementary Figure S71: HSQC spectra for 3c

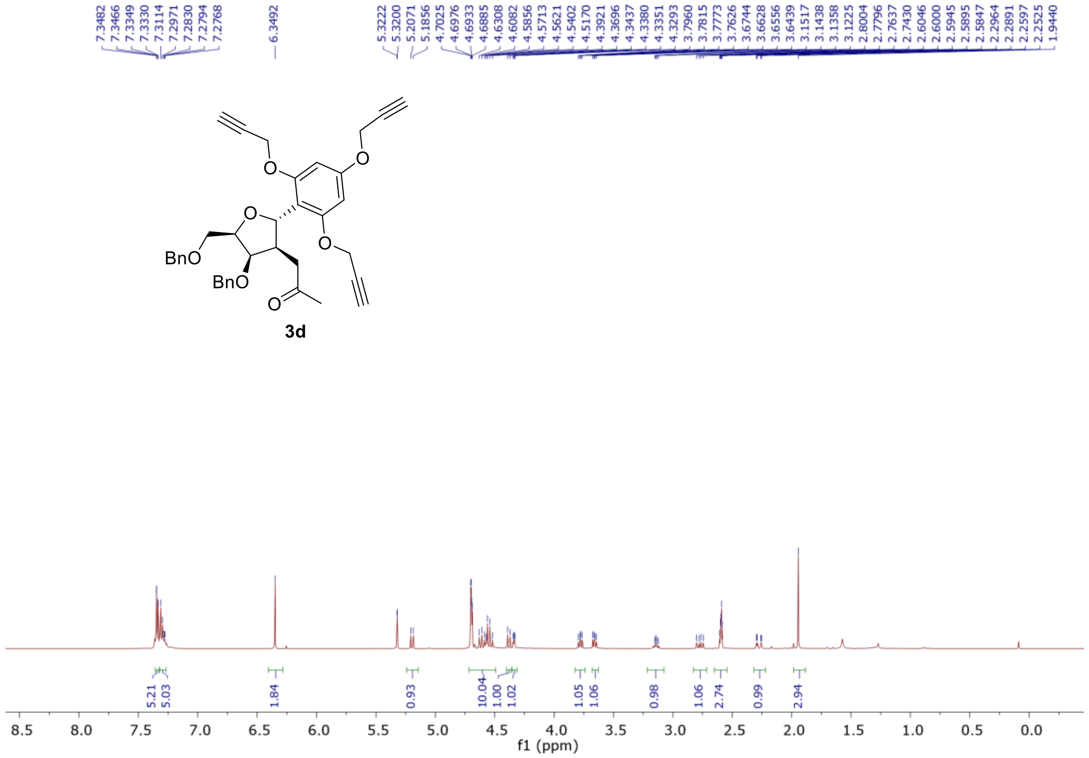

Supplementary Figure S72: ^{1}H NMR spectra for 3d

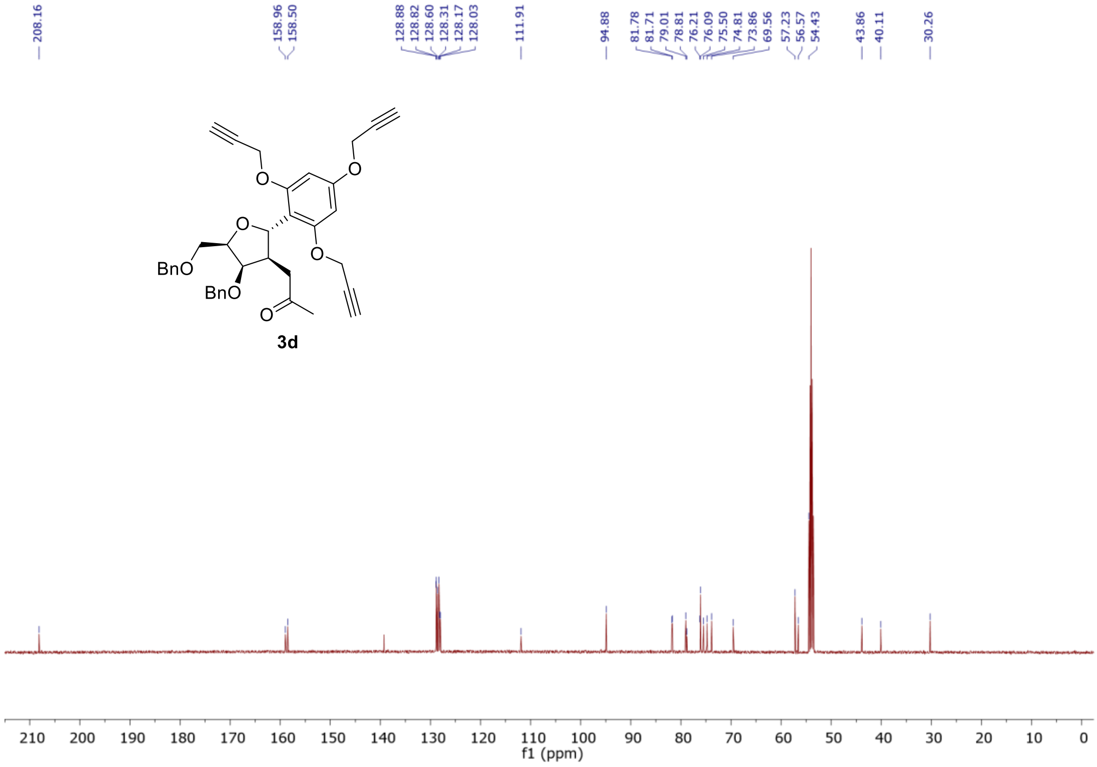

Supplementary Figure S73: ^{13}C NMR spectra for 3d

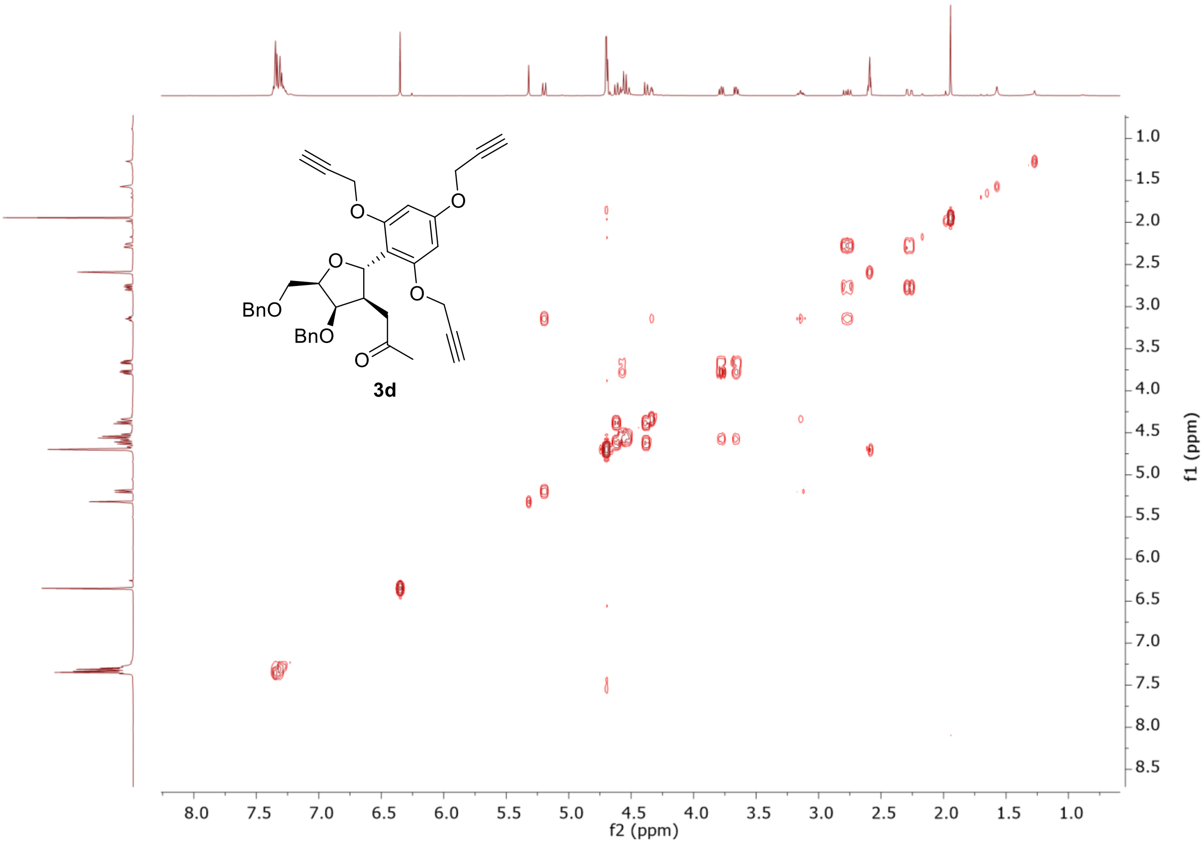

Supplementary Figure S74: COSY spectra for 3d

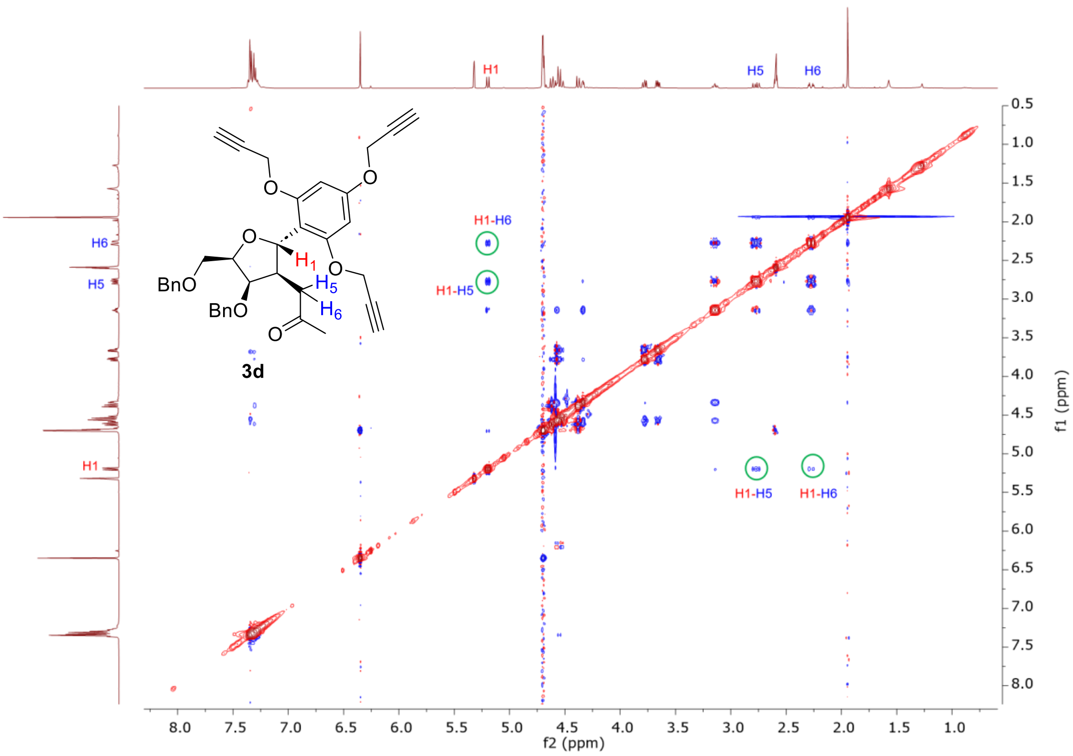

Supplementary Figure S75: NOESY spectra for 3d

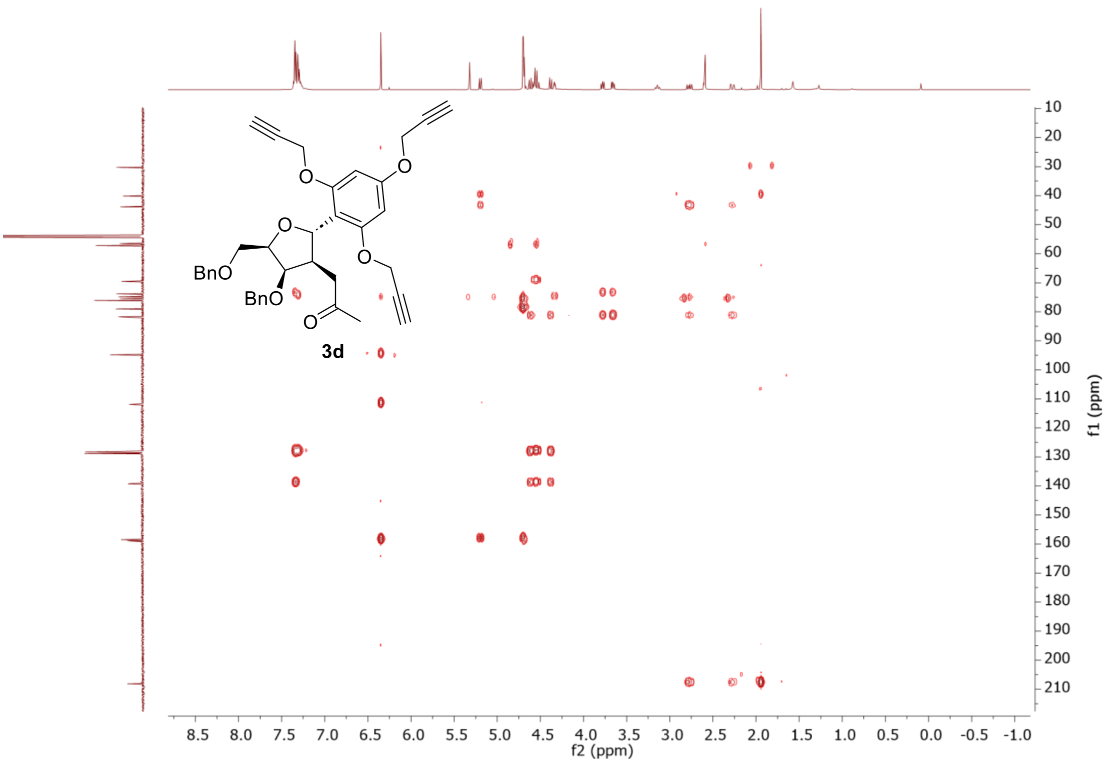

Supplementary Figure S76: HMBC spectra for 3d

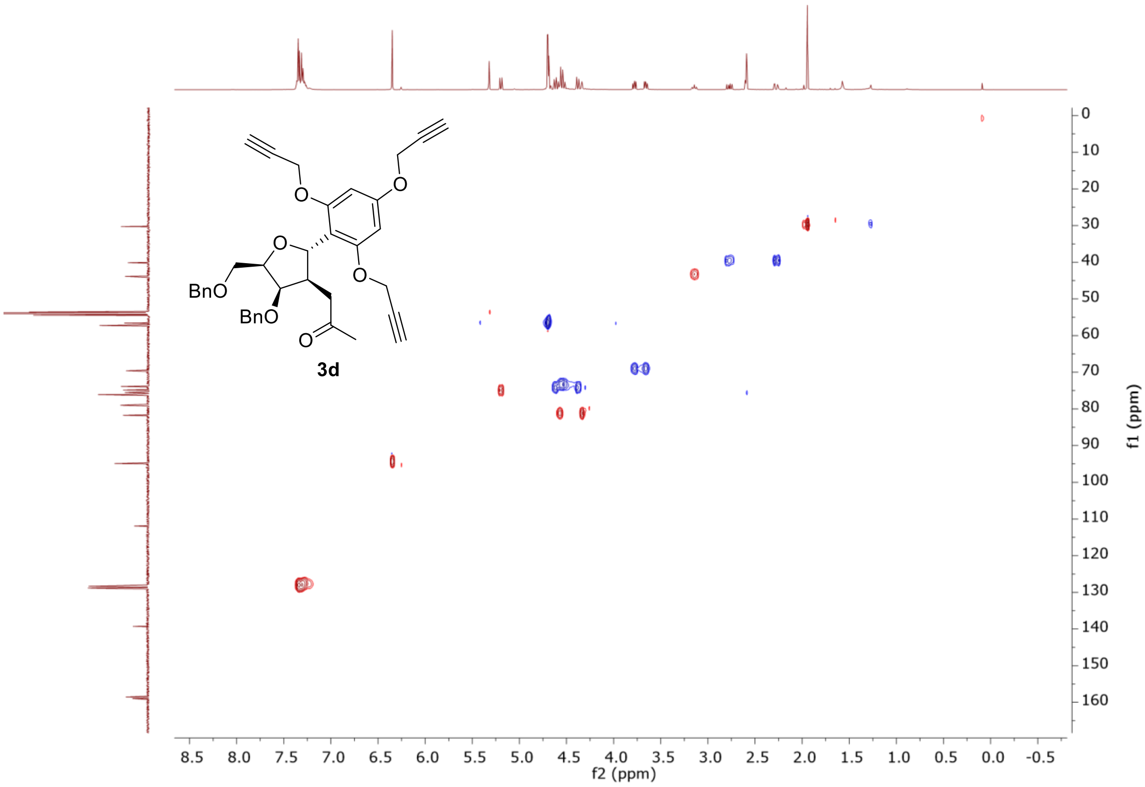

Supplementary Figure S77: HSQC spectra for 3d

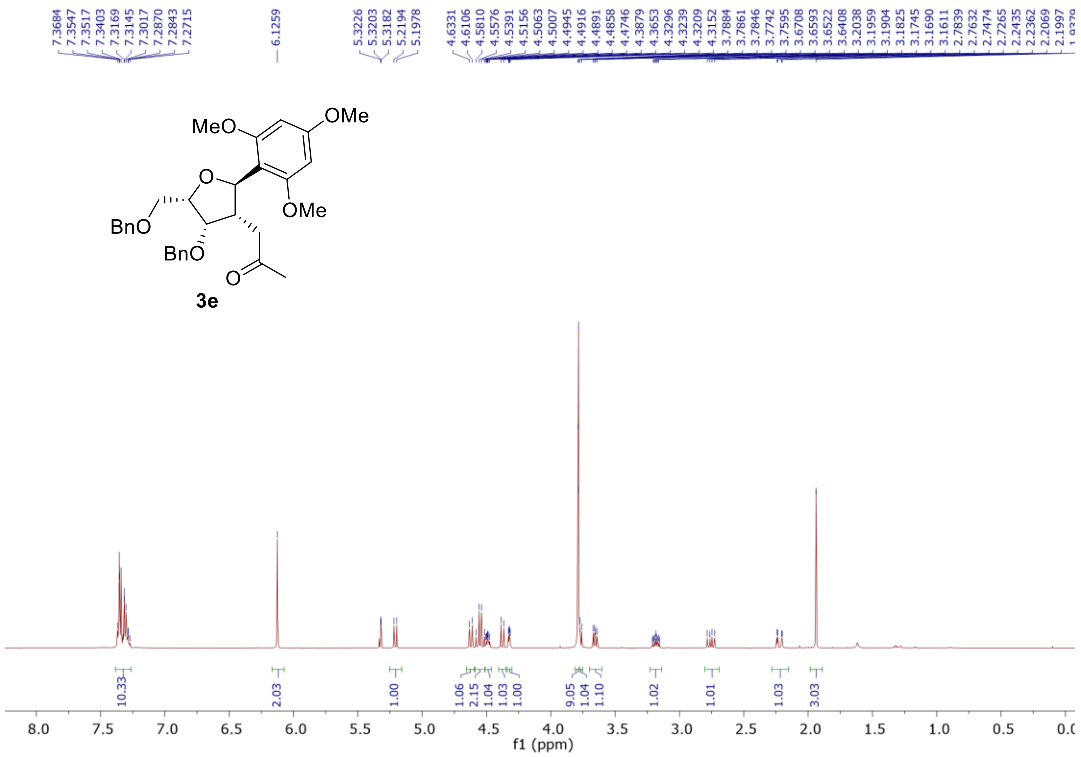

Supplementary Figure S78: ^{1}H NMR spectra for 3e

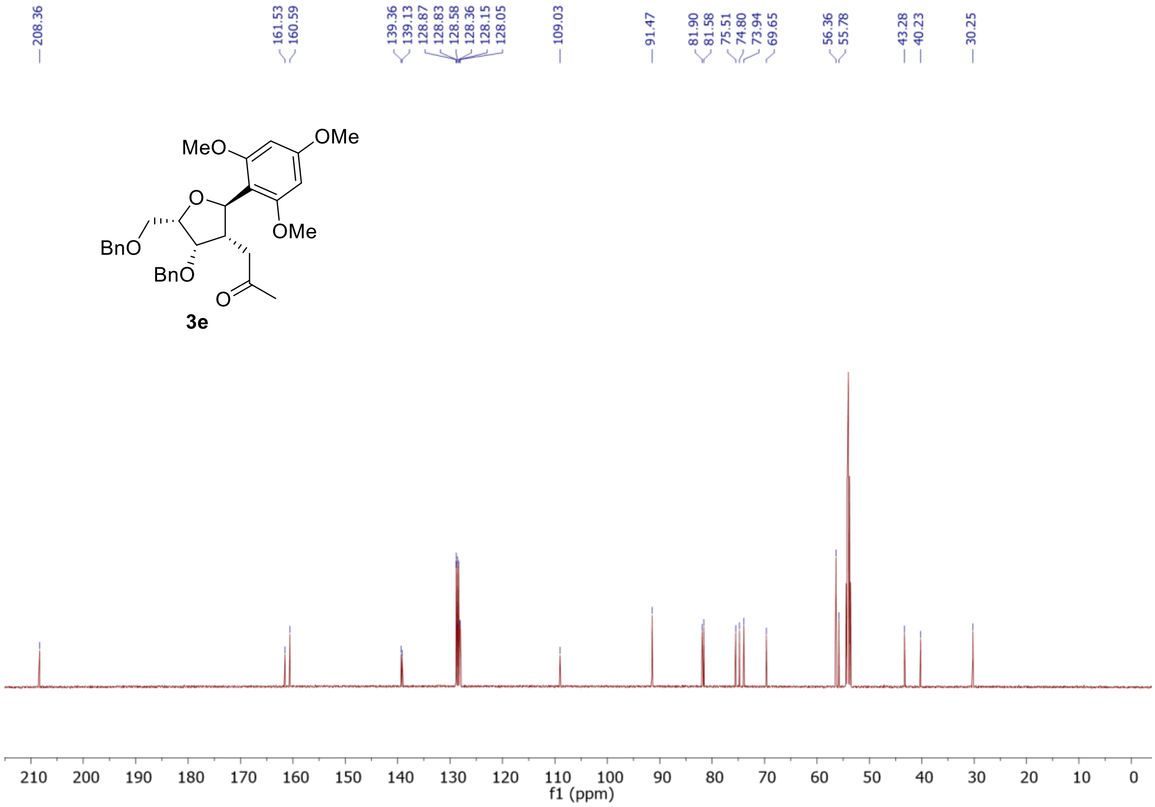

Supplementary Figure S79: ^{13}C NMR spectra for 3e

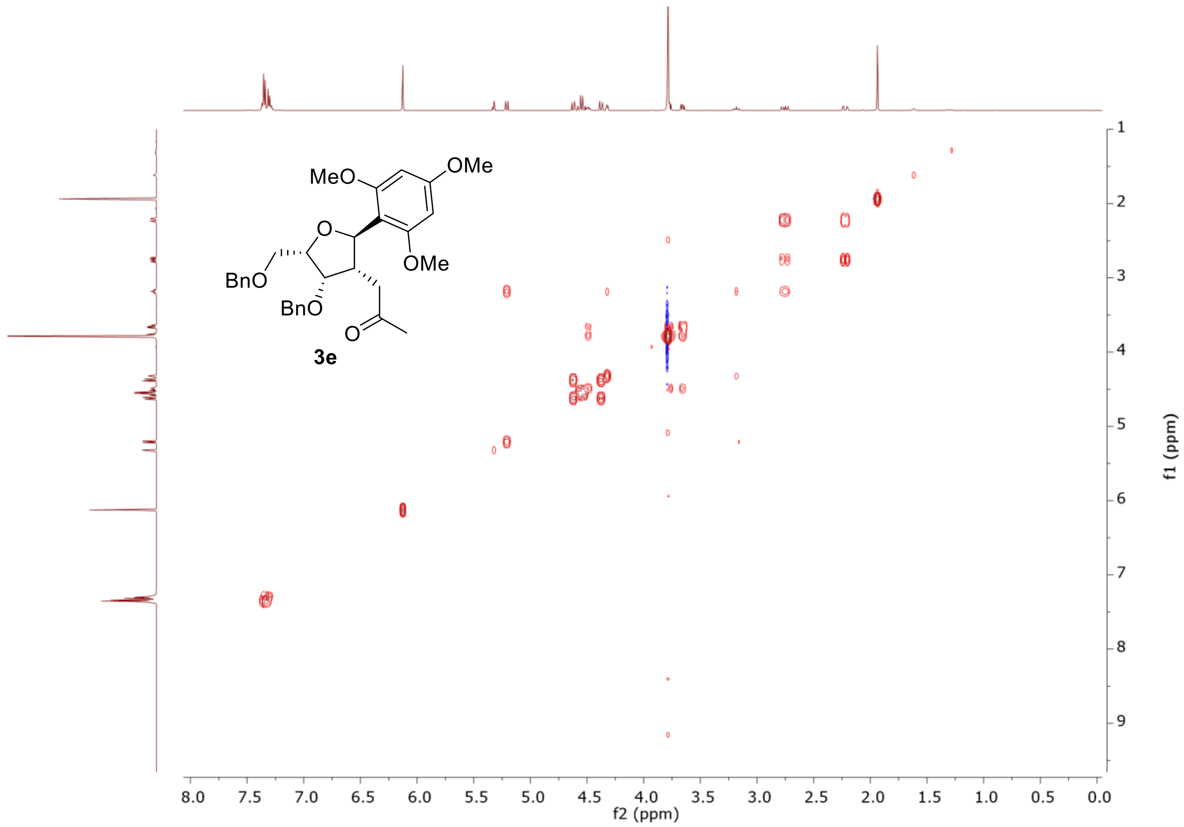

Supplementary Figure S80: COSY spectra for 3e

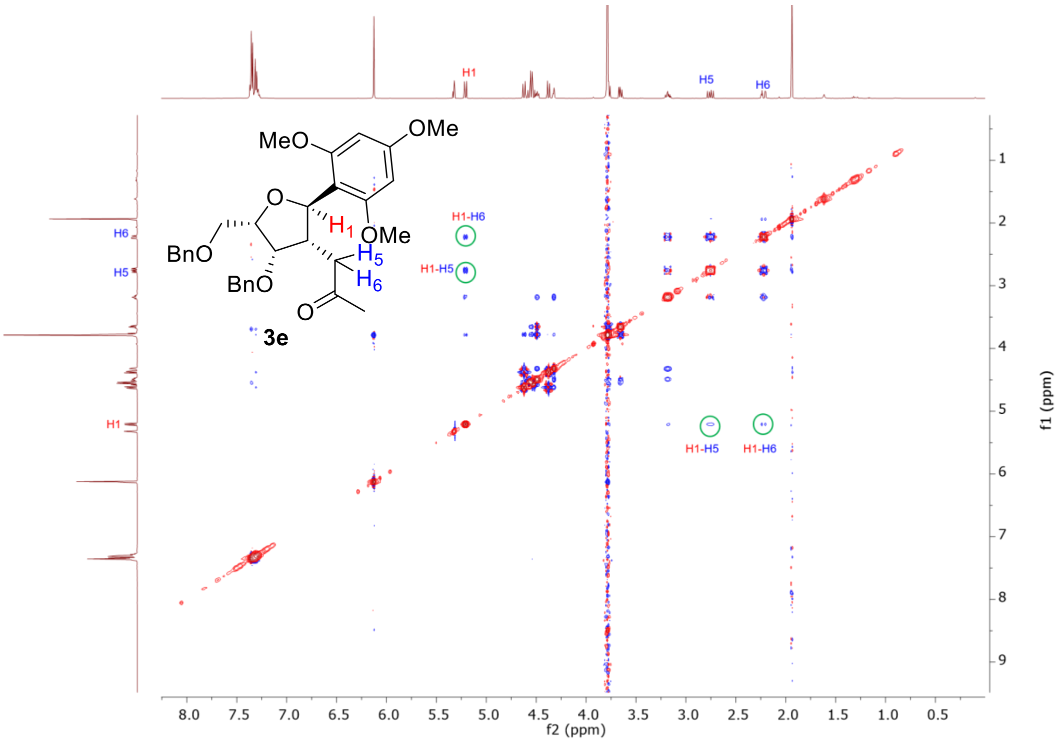

Supplementary Figure S81: NOESY spectra for 3e

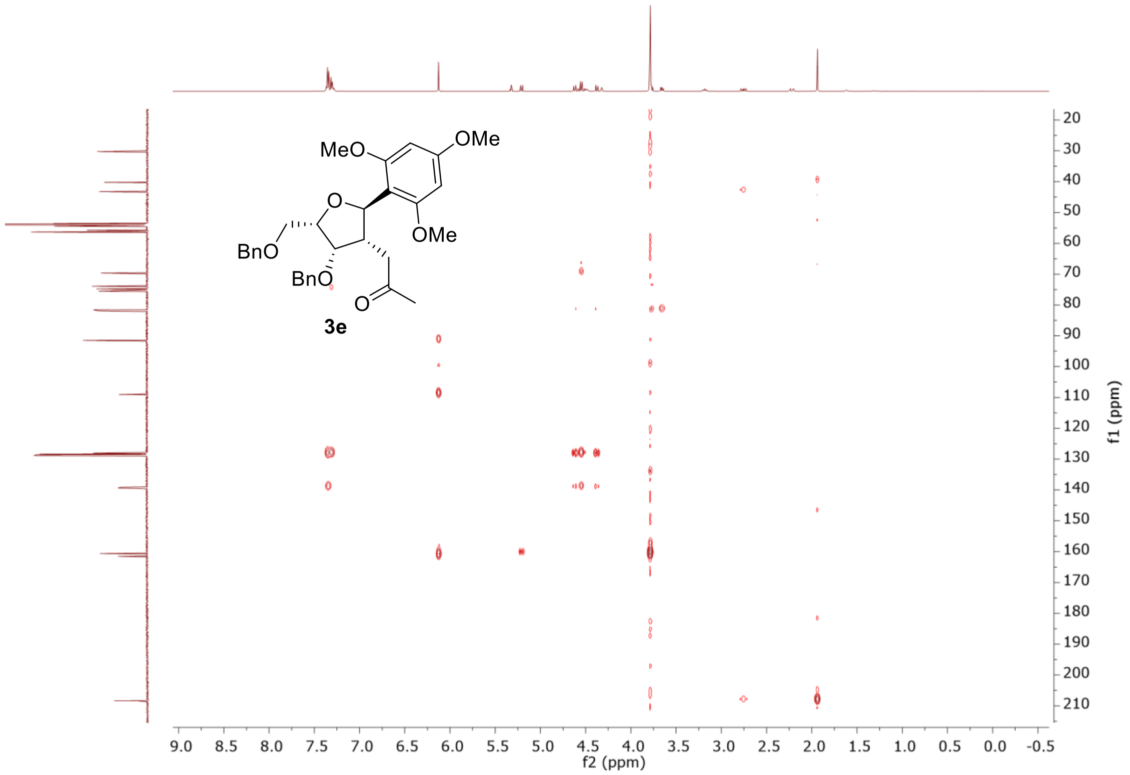

Supplementary Figure S82: HMBC spectra for 3e

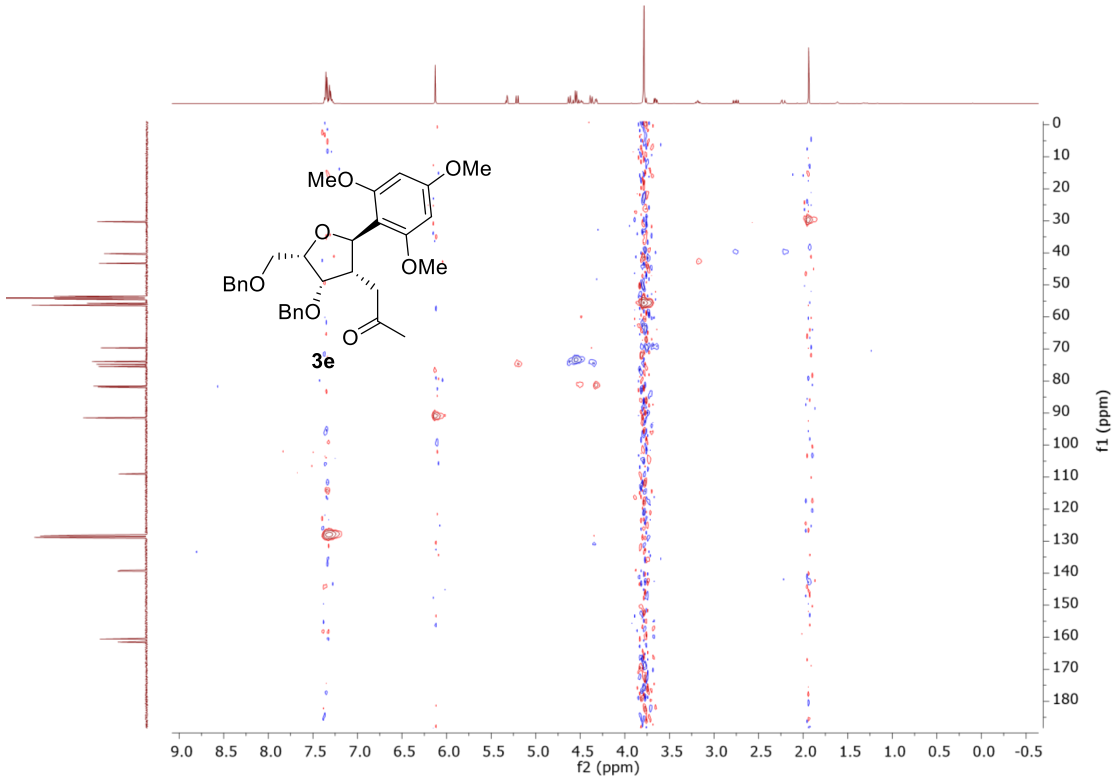

Supplementary Figure S83: HSQC spectra for 3e

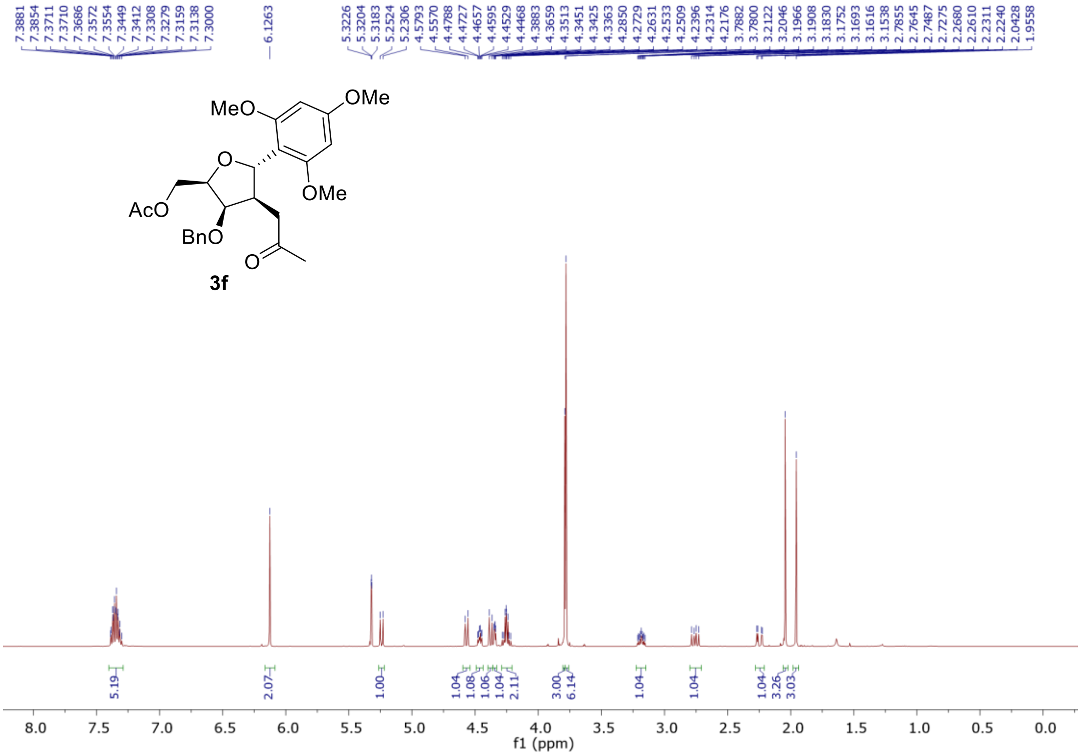

Supplementary Figure S84: ^{1}H NMR spectra for 3f

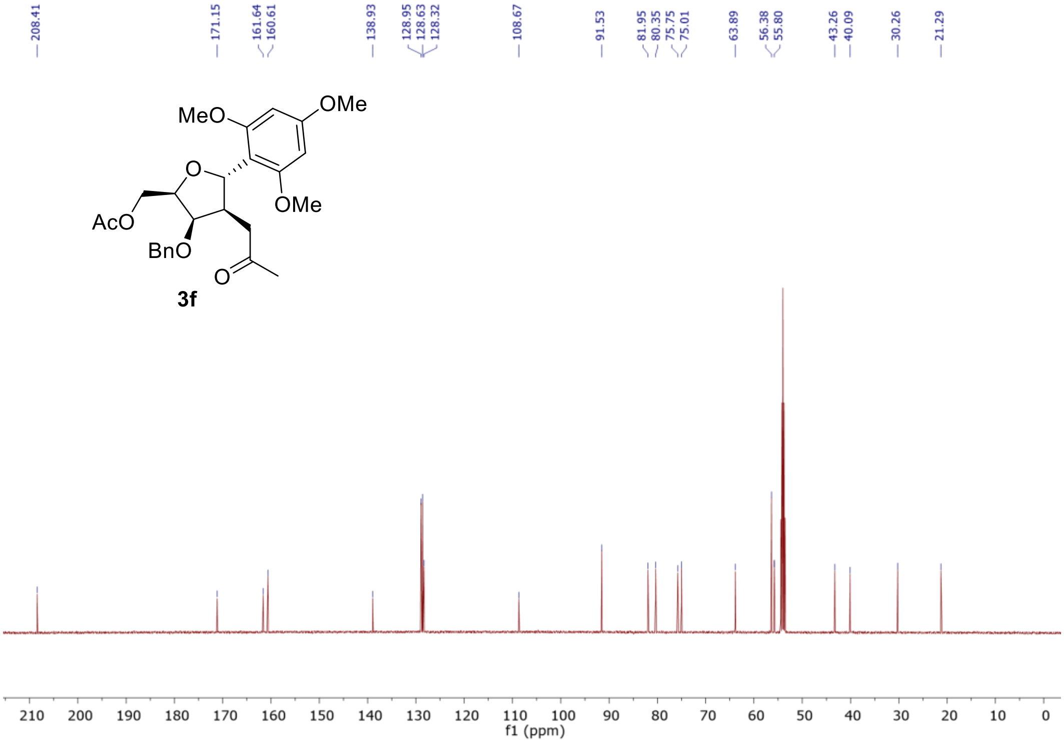

Supplementary Figure S85: ^{13}C NMR spectra for 3f

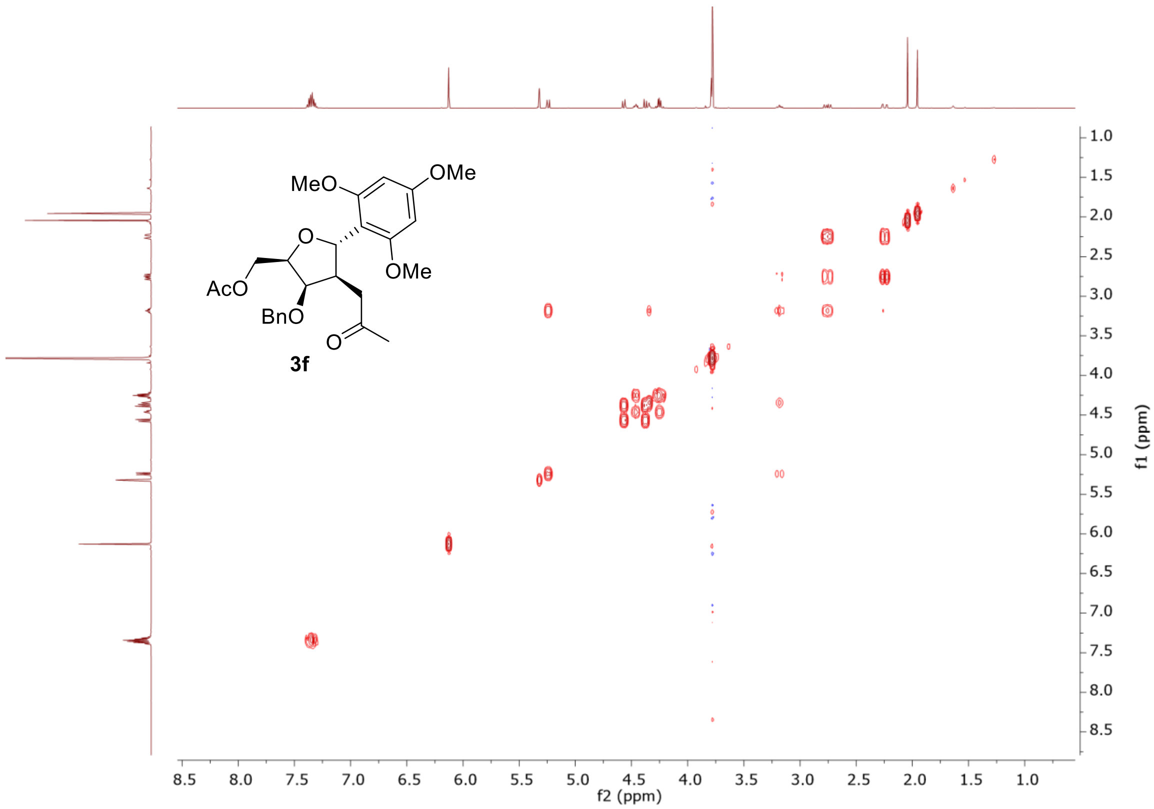

Supplementary Figure S86: COSY spectra for 3f

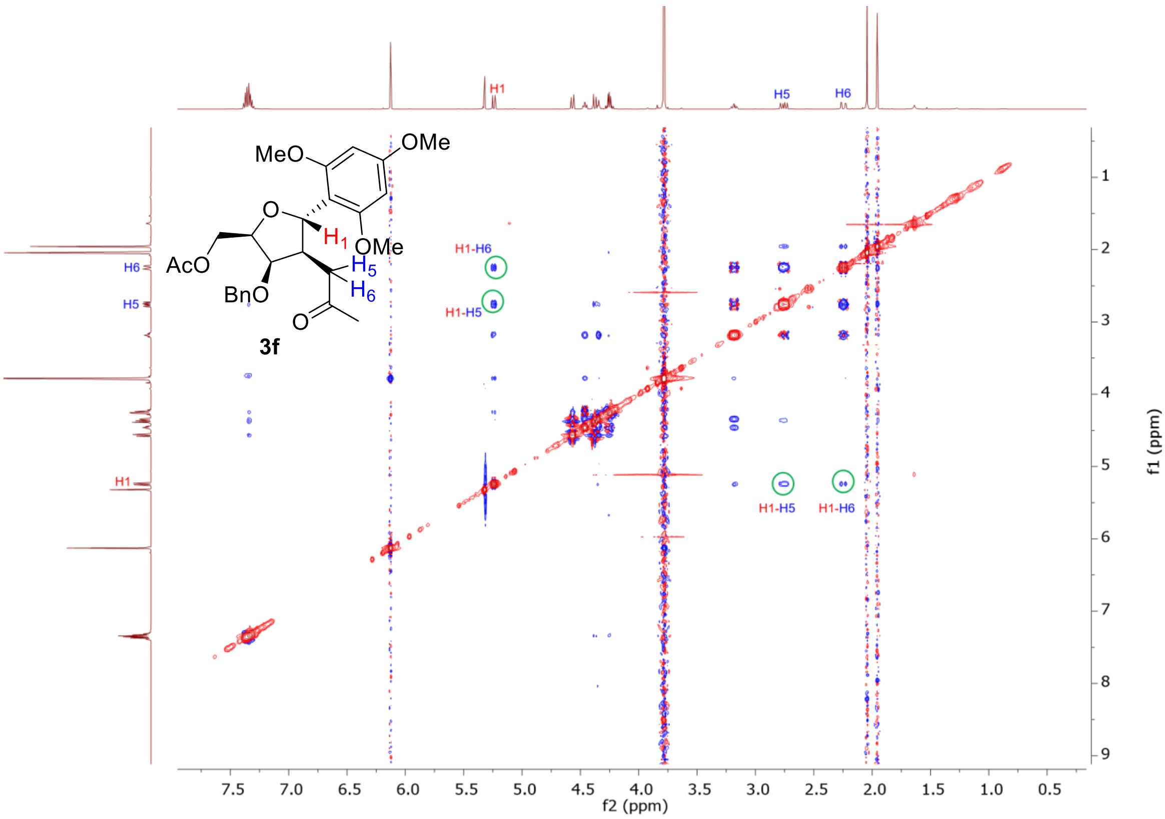

Supplementary Figure S87: NOESY spectra for 3f

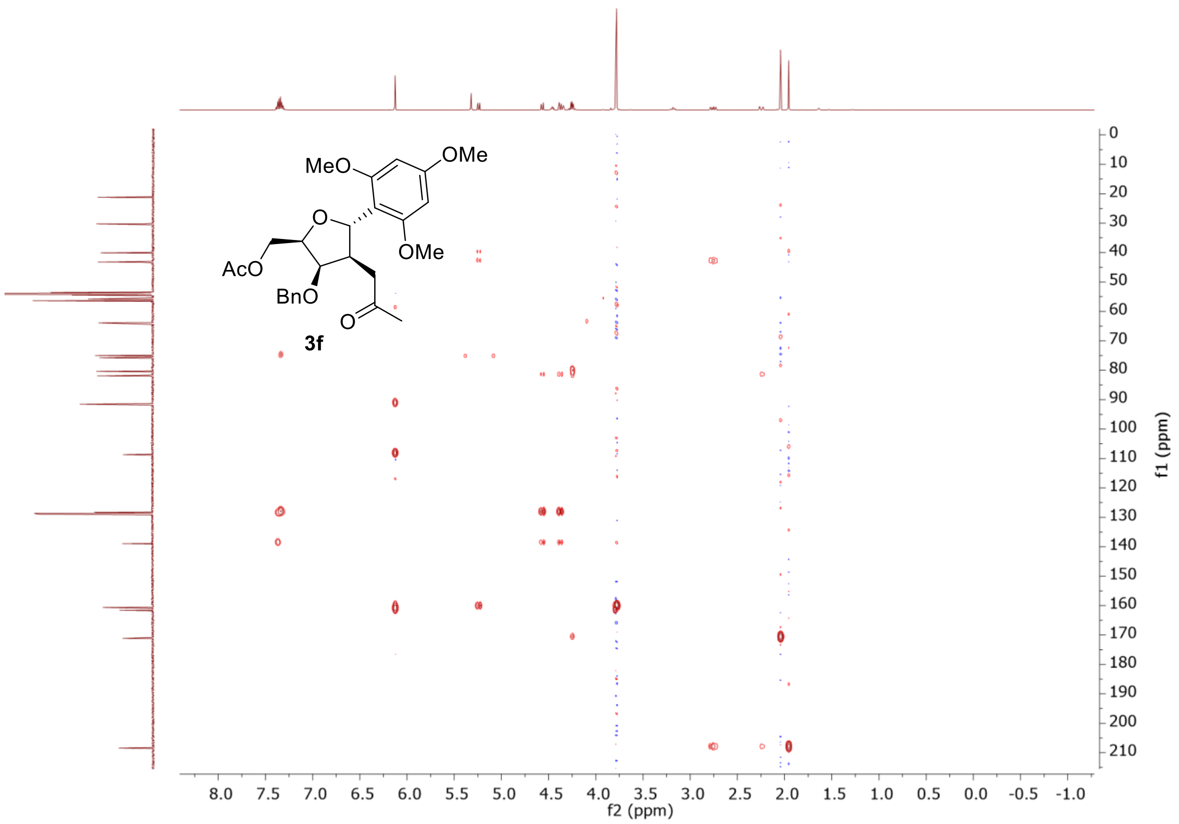

Supplementary Figure S88: HMBC spectra for 3f

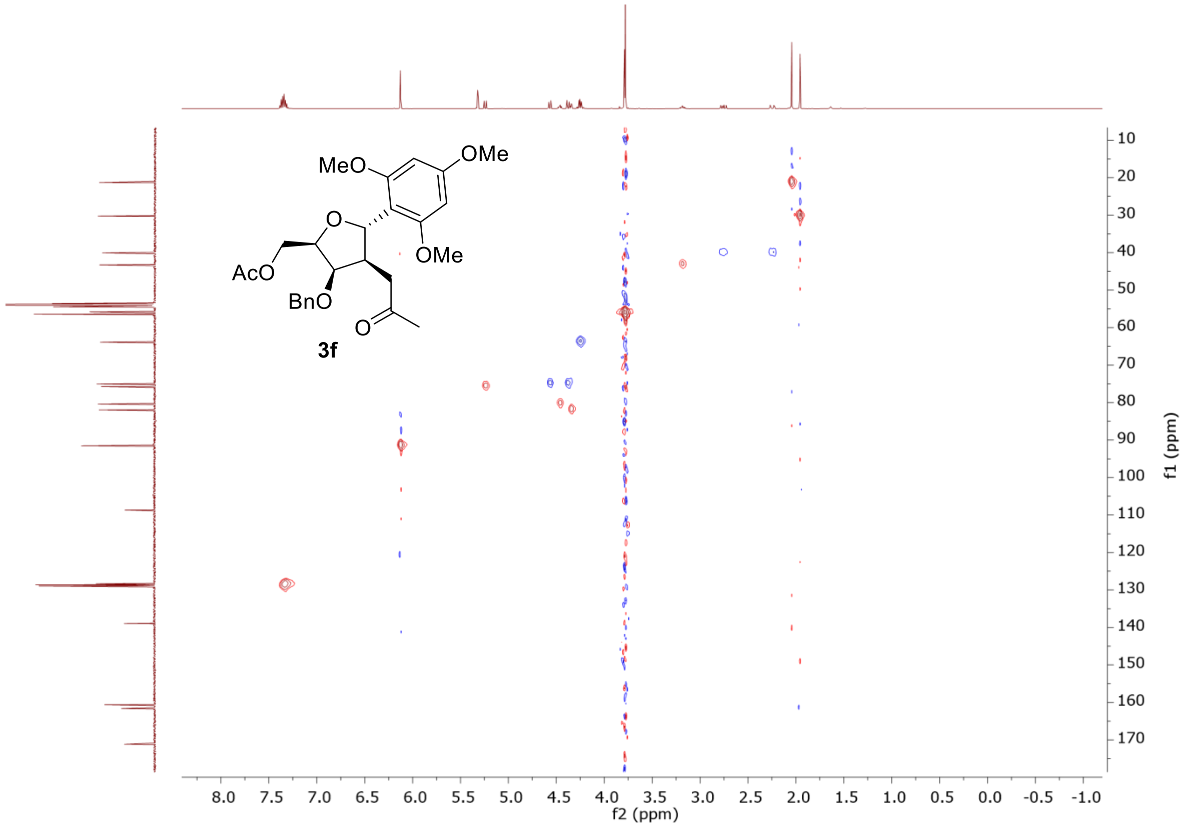

Supplementary Figure S89: HSQC spectra for 3f

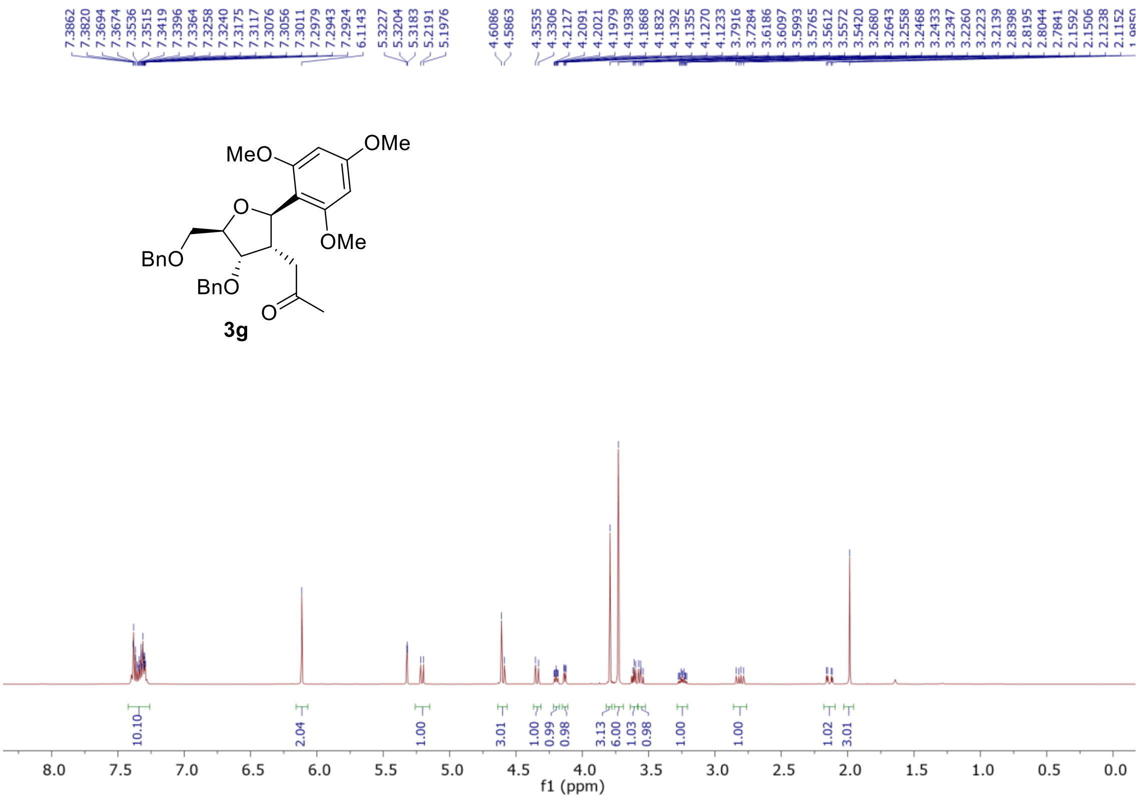

Supplementary Figure S90: ^{1}H NMR spectra for 3g

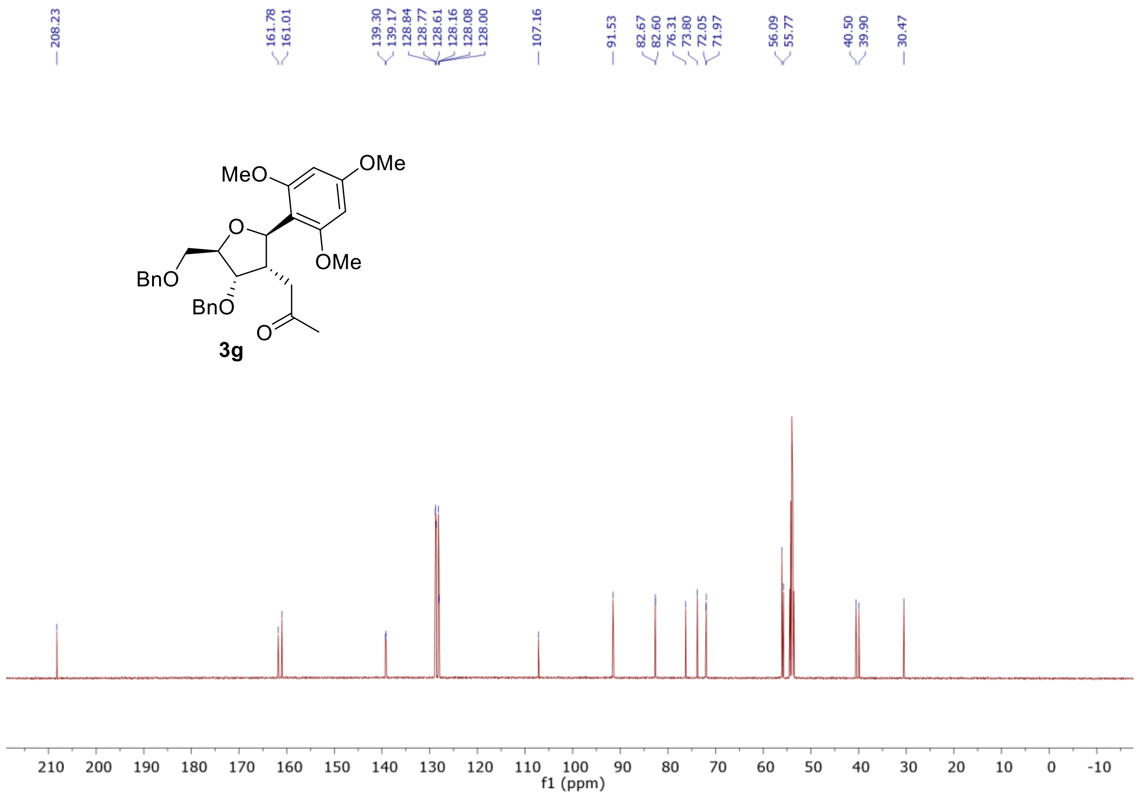

Supplementary Figure S91: ^{13}C NMR spectra for 3g

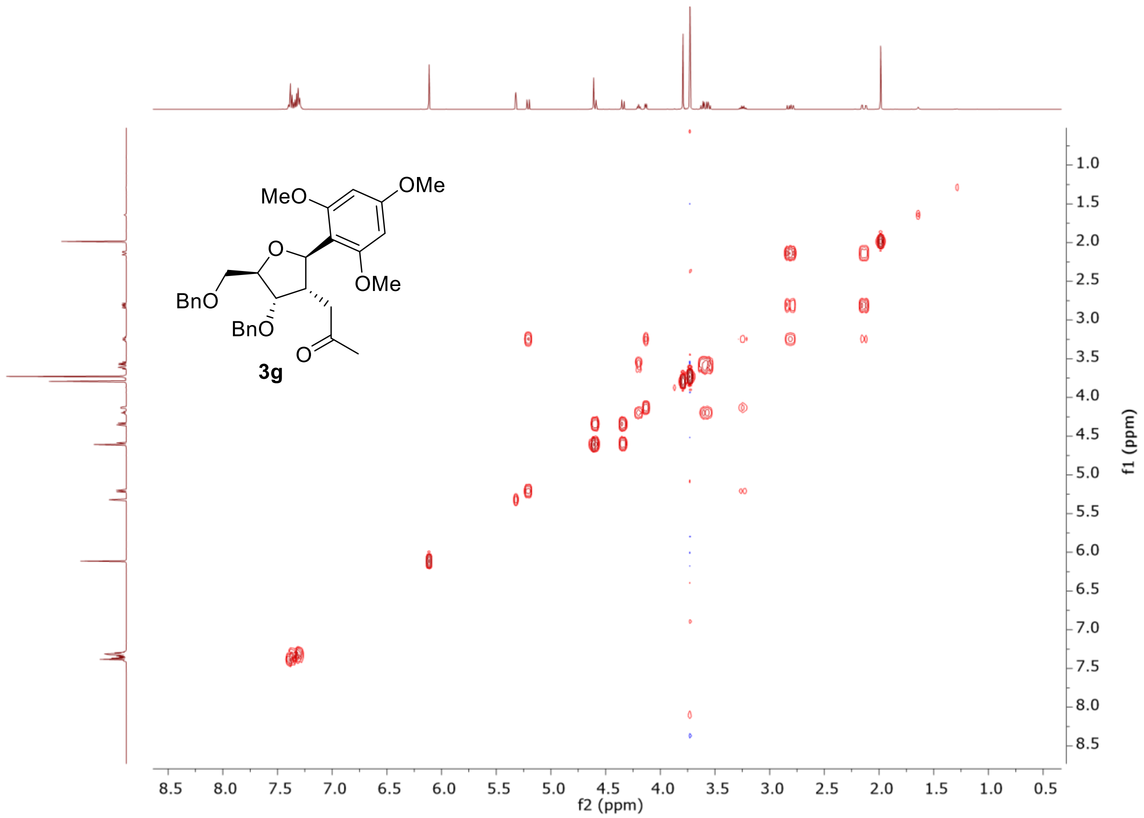

Supplementary Figure S92: COSY spectra for 3g

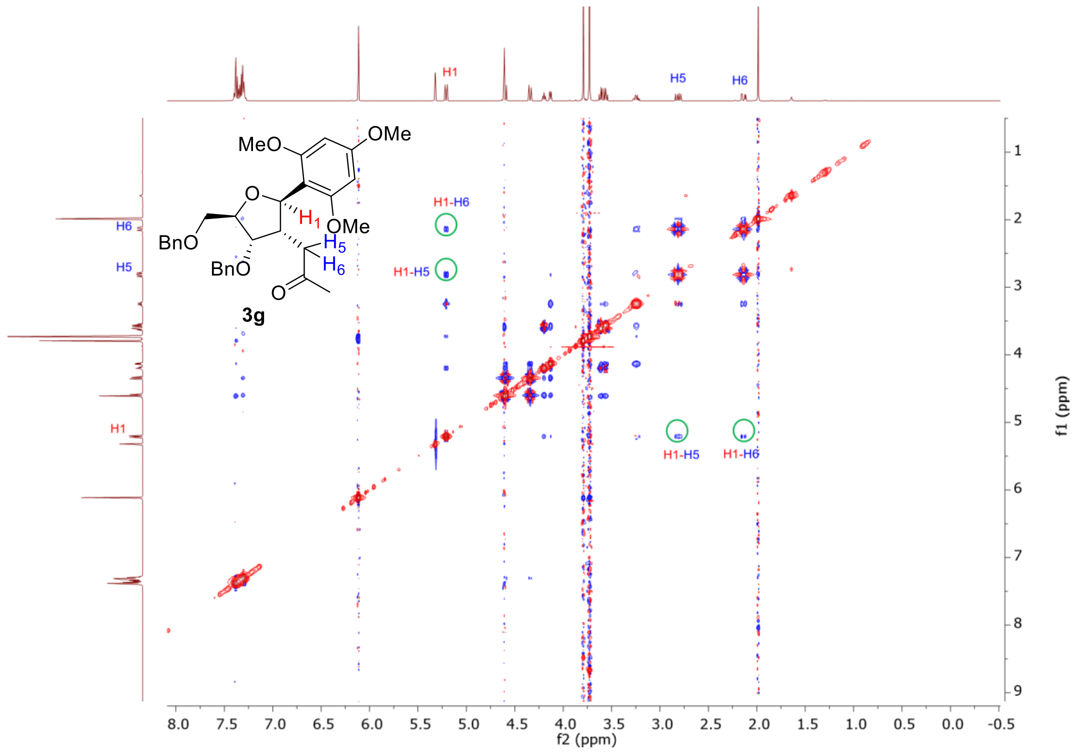

Supplementary Figure S93: NOESY spectra for 3g

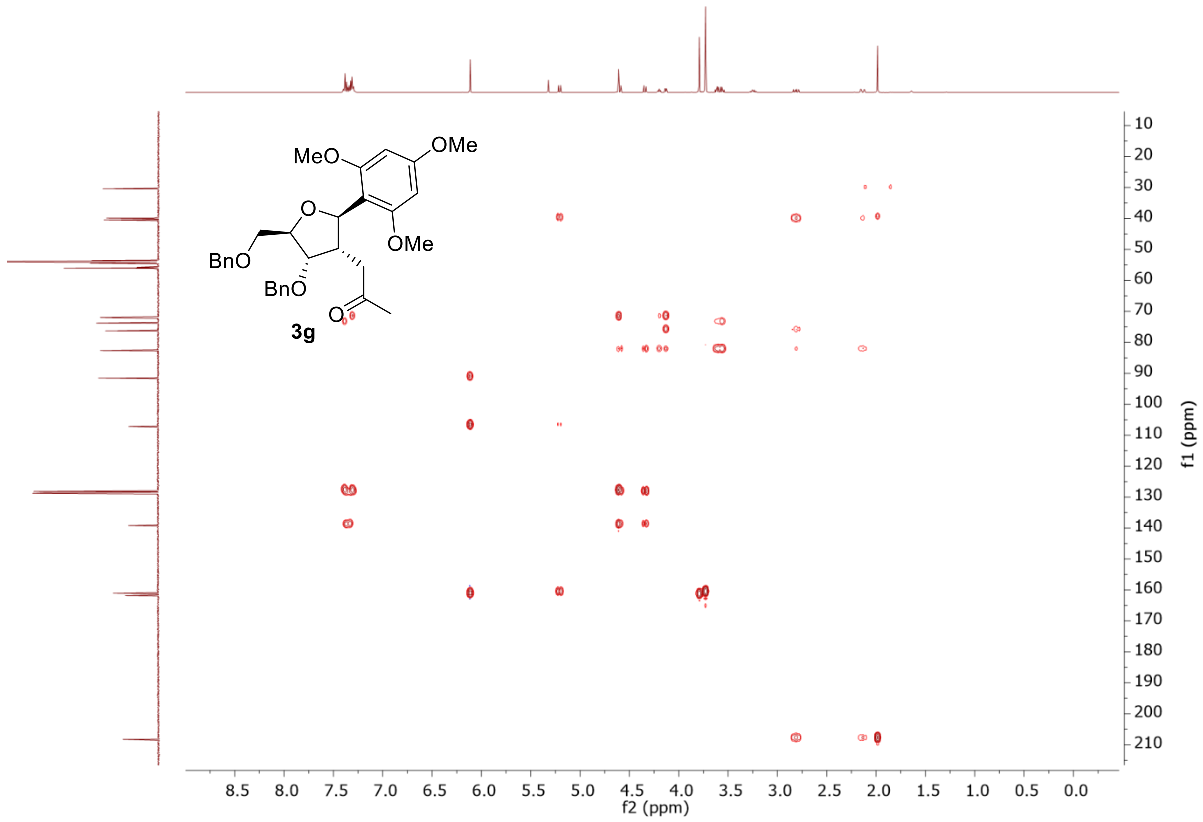

Supplementary Figure S94: HMBC spectra for 3g

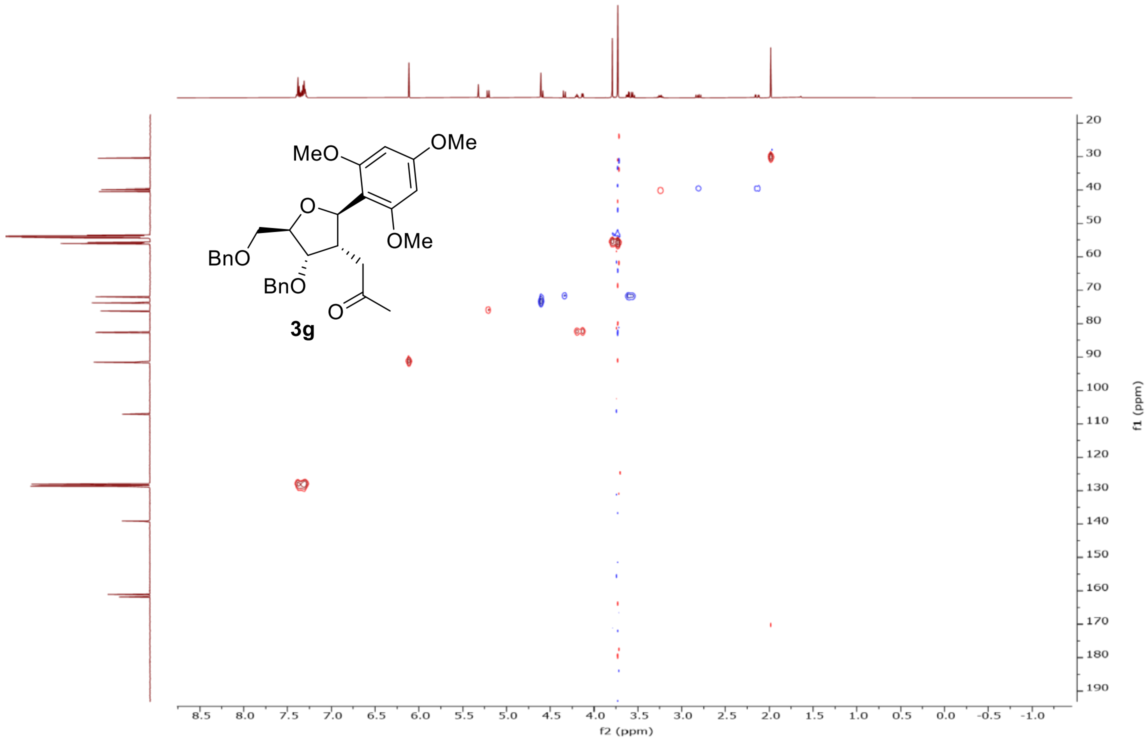

Supplementary Figure S95: HSQC spectra for 3g

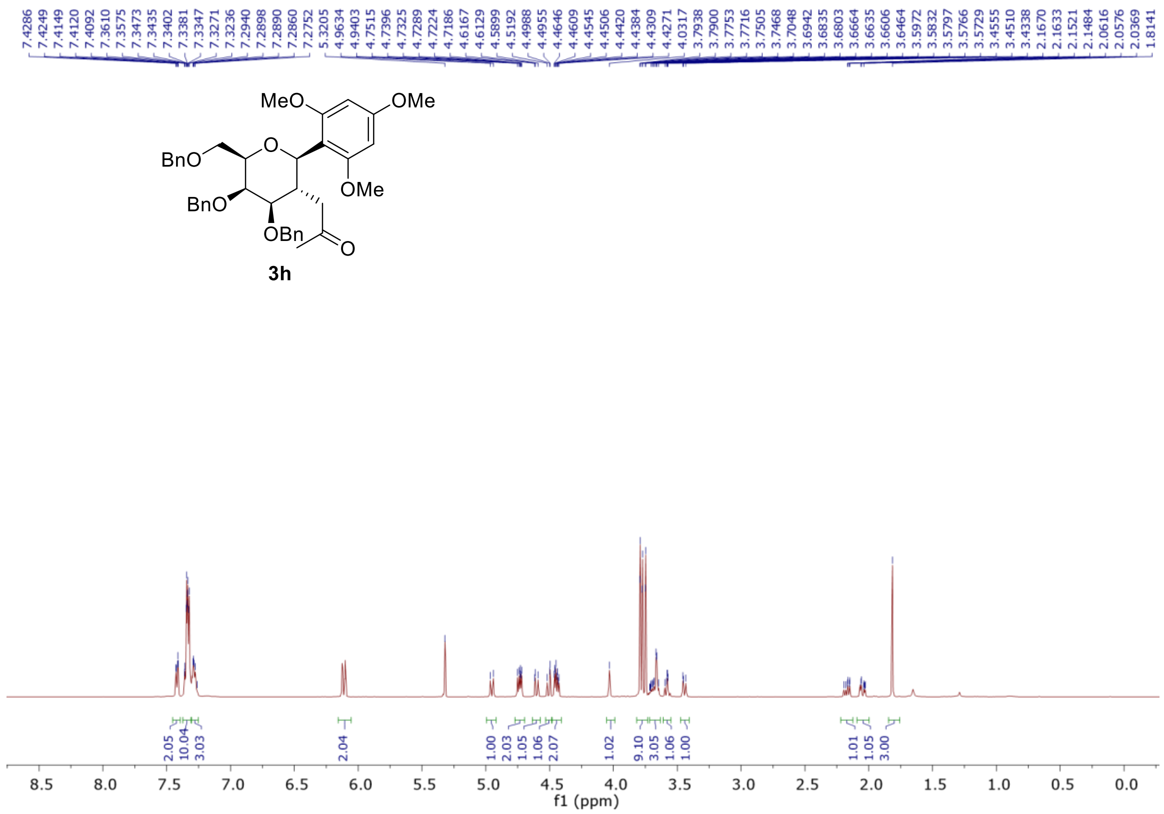

Supplementary Figure S96: ^{1}H NMR spectra for 3h

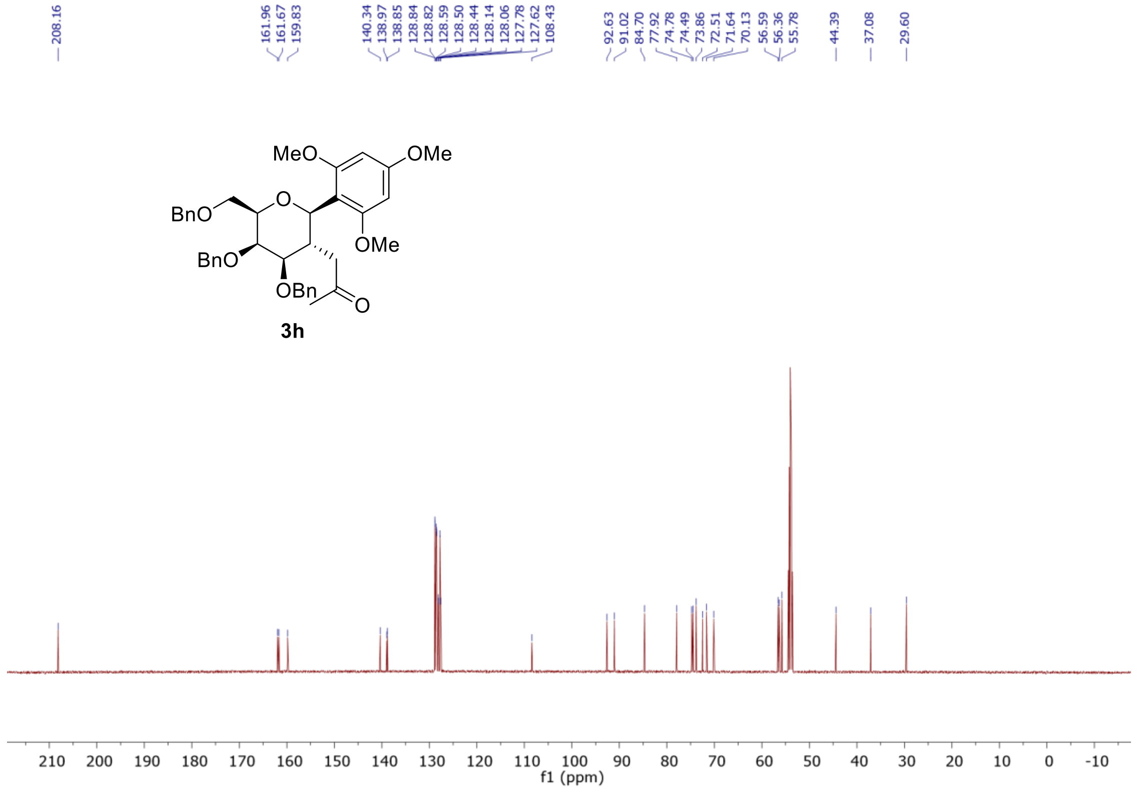

Supplementary Figure S97: ^{13}C NMR spectra for 3h

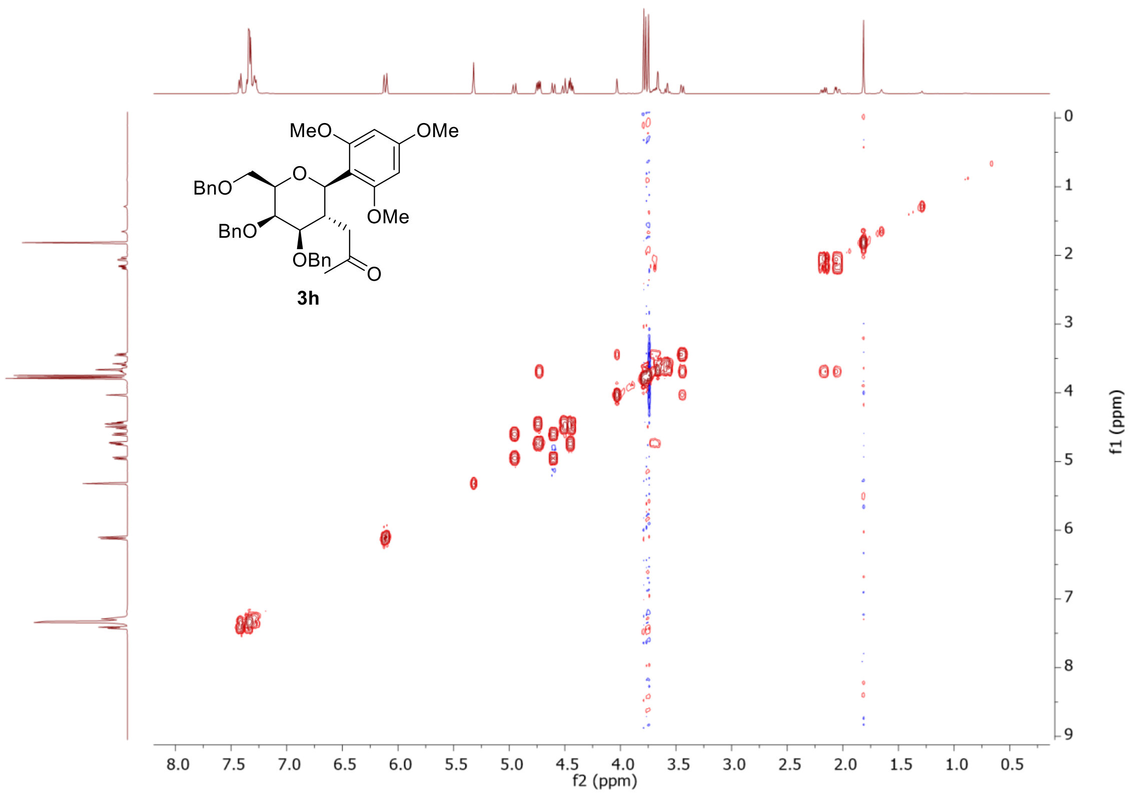

Supplementary Figure S98: COSY spectra for 3h

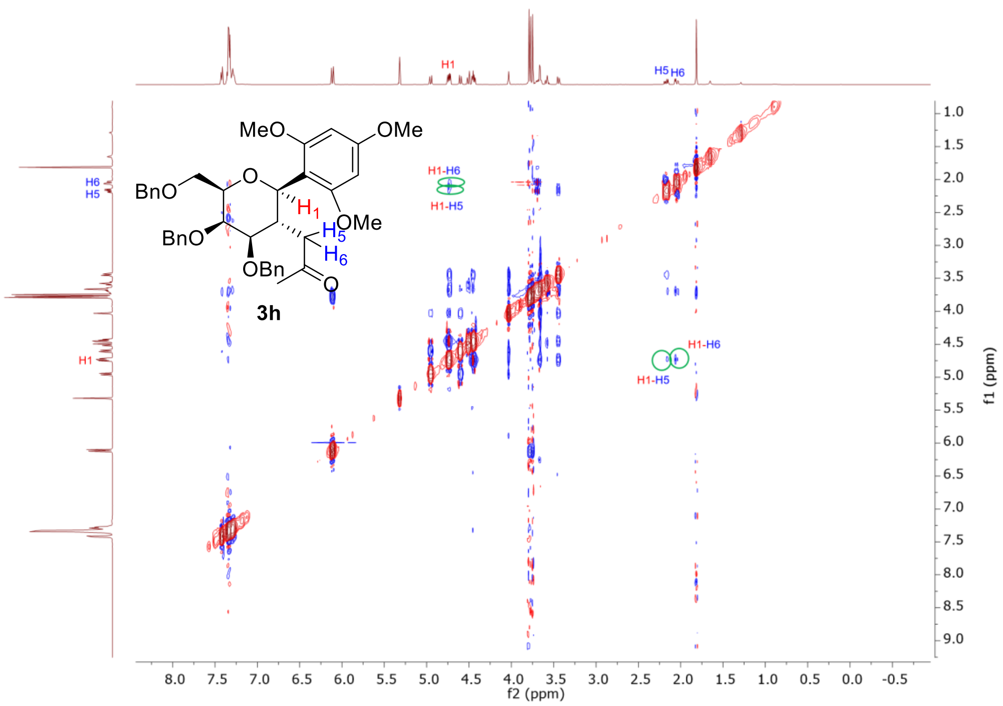

Supplementary Figure S99: NOESY spectra for 3h

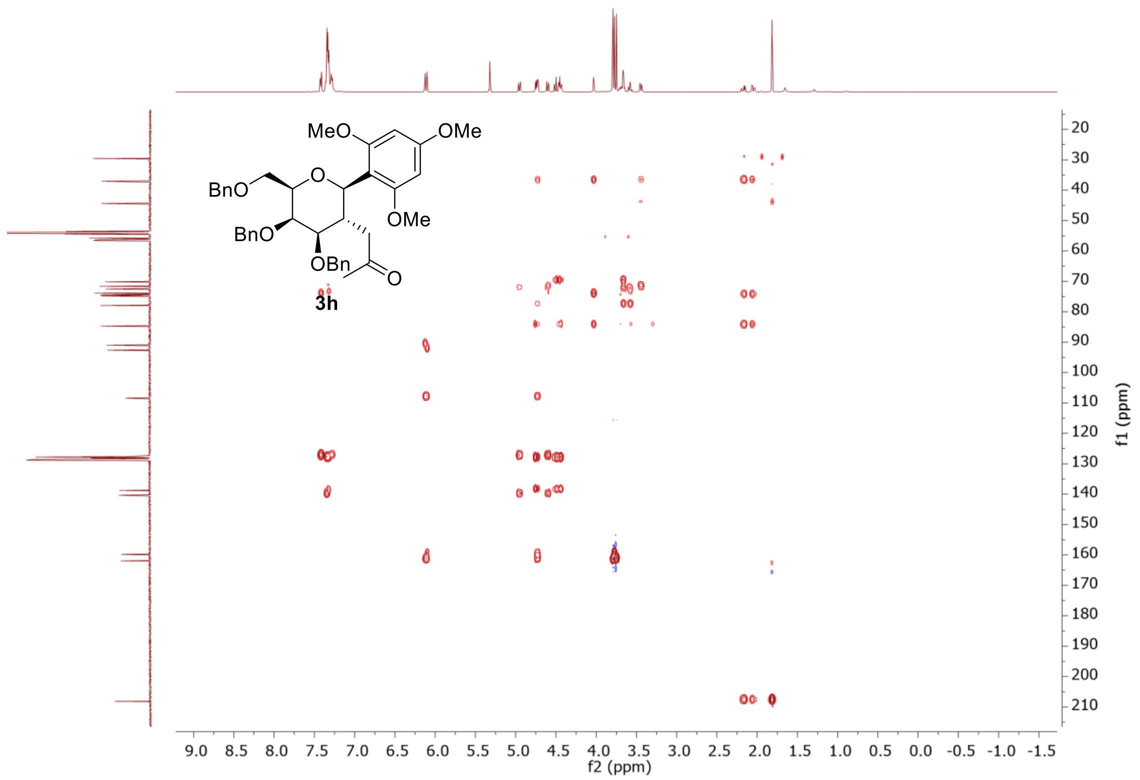

Supplementary Figure S100: HMBC spectra for 3h

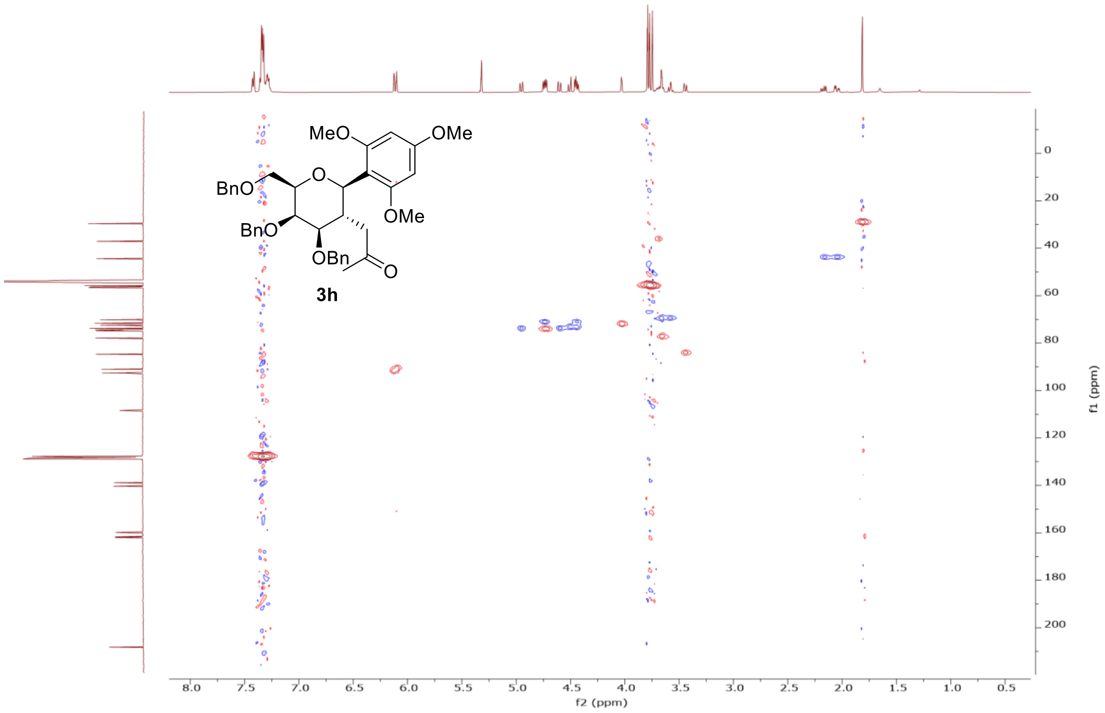

Supplementary Figure S101: HSQC spectra for 3h

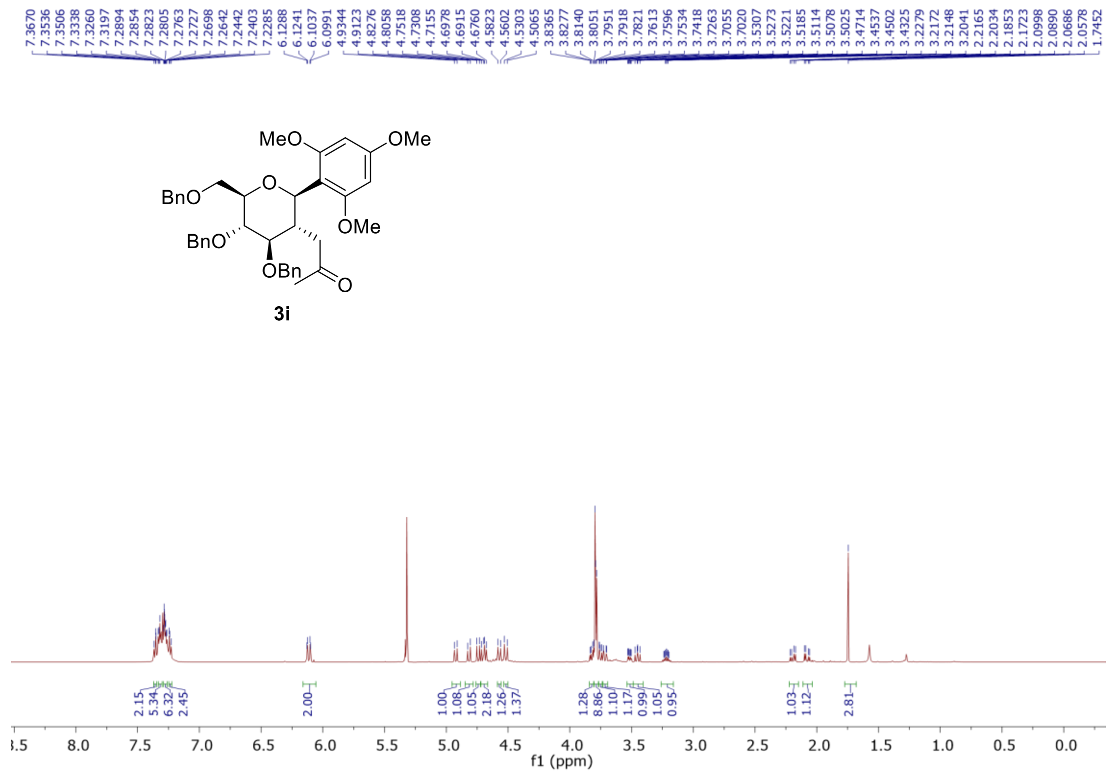

Supplementary Figure S102: ^{1}H NMR spectra for 3i

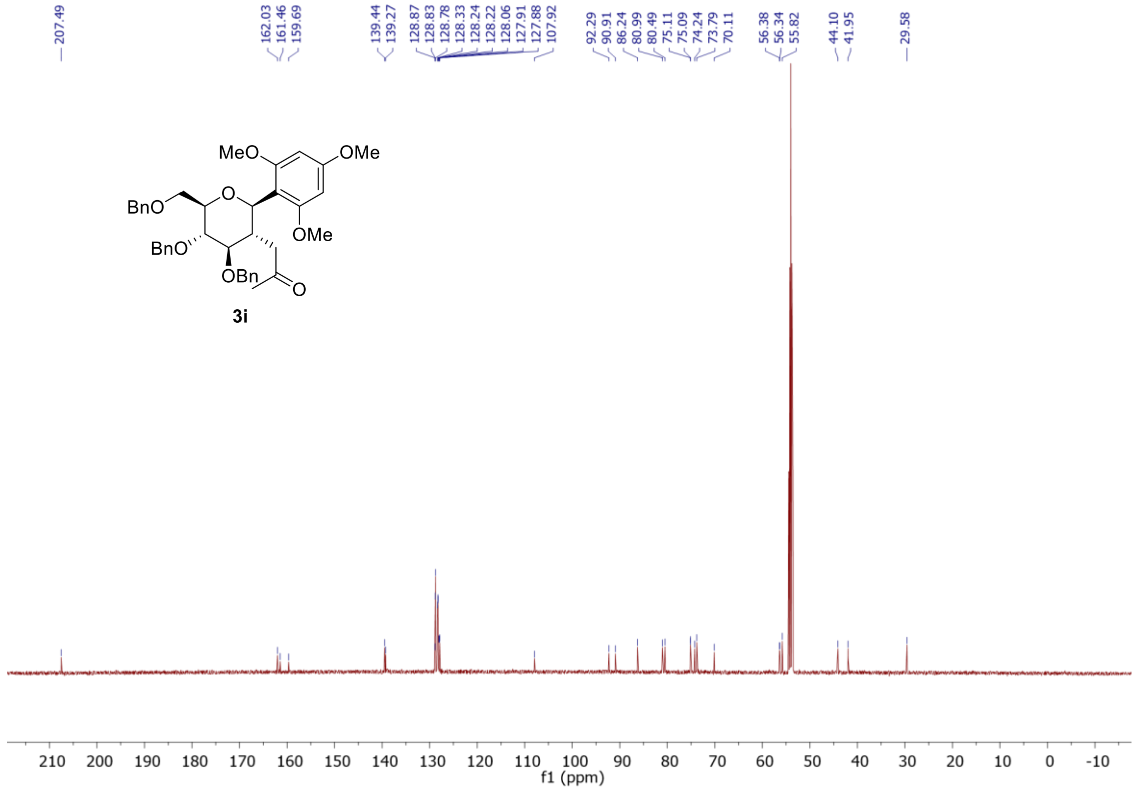

Supplementary Figure S103: ^{13}C NMR spectra for 3i

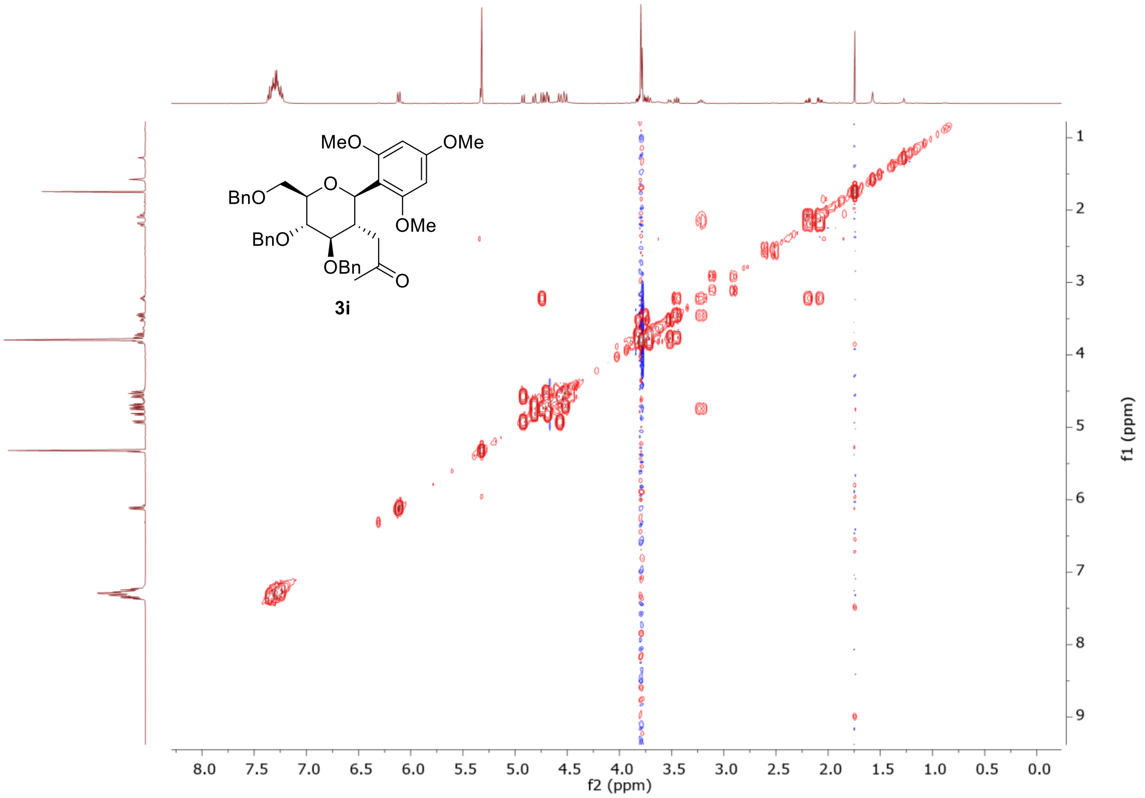

Supplementary Figure S104: COSY spectra for 3i

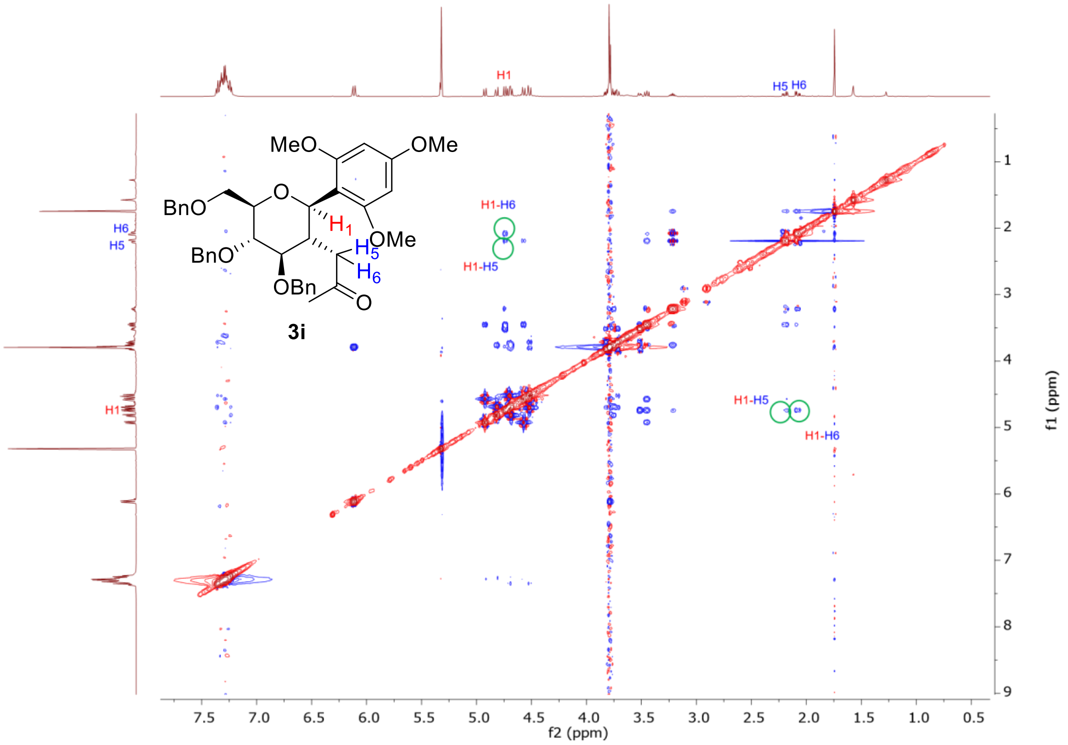

Supplementary Figure S105: NOESY spectra for 3i

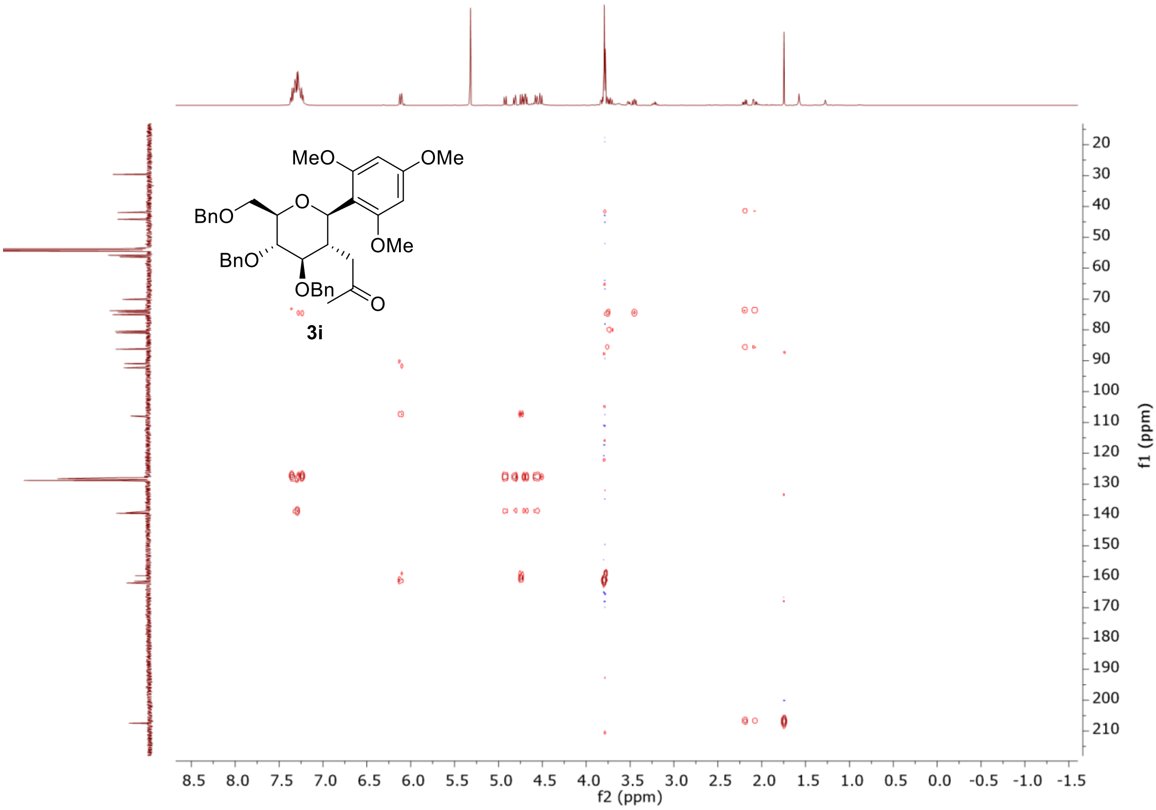

Supplementary Figure S106: HMBC spectra for 3i

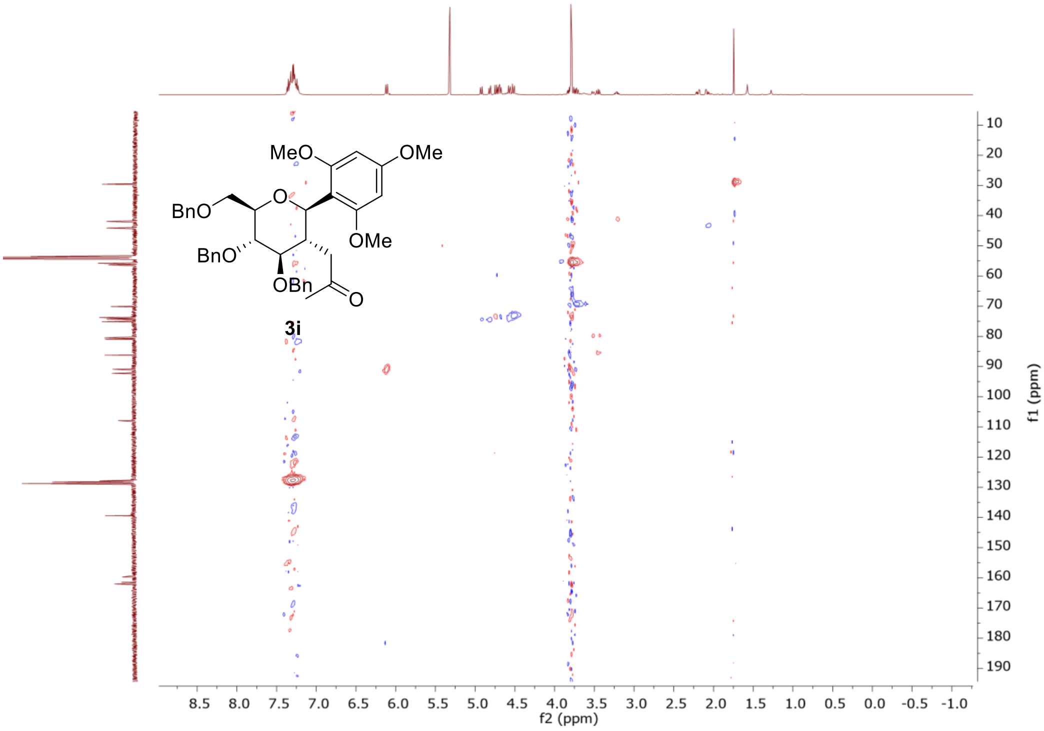

Supplementary Figure S107: HSQC spectra for 3i

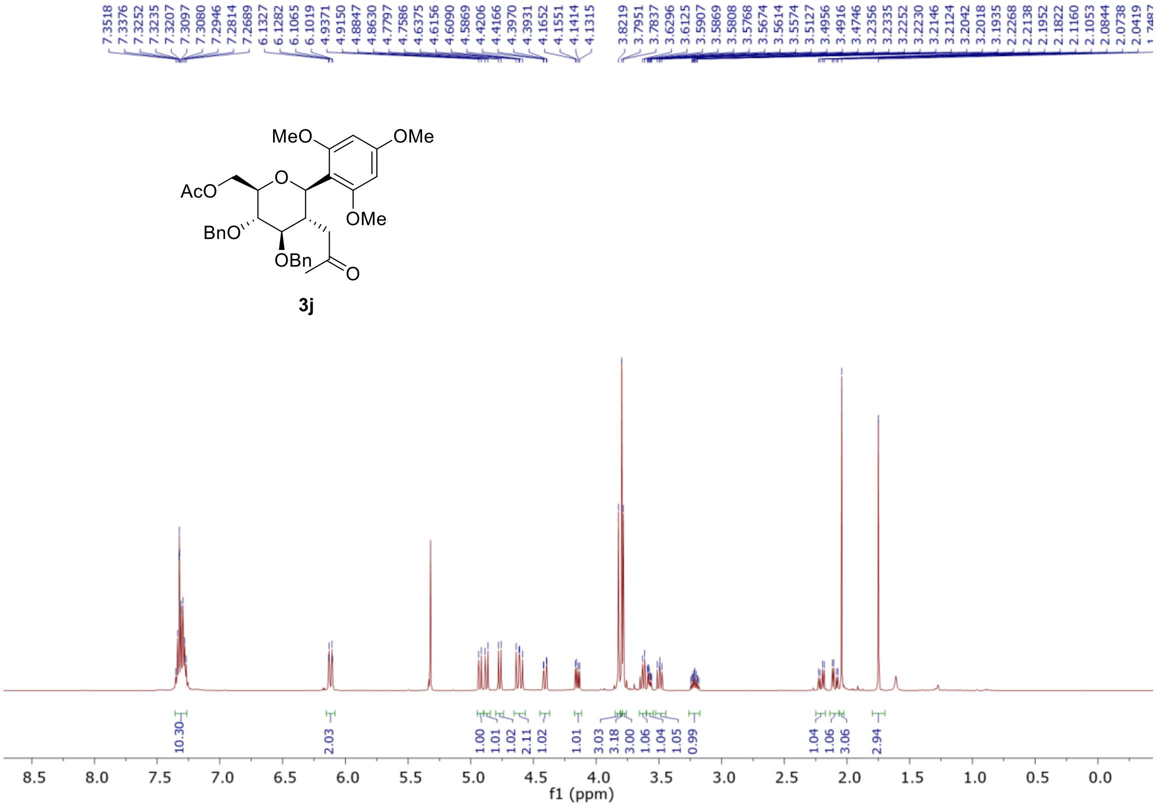

Supplementary Figure S108: ^{1}H NMR spectra for 3j

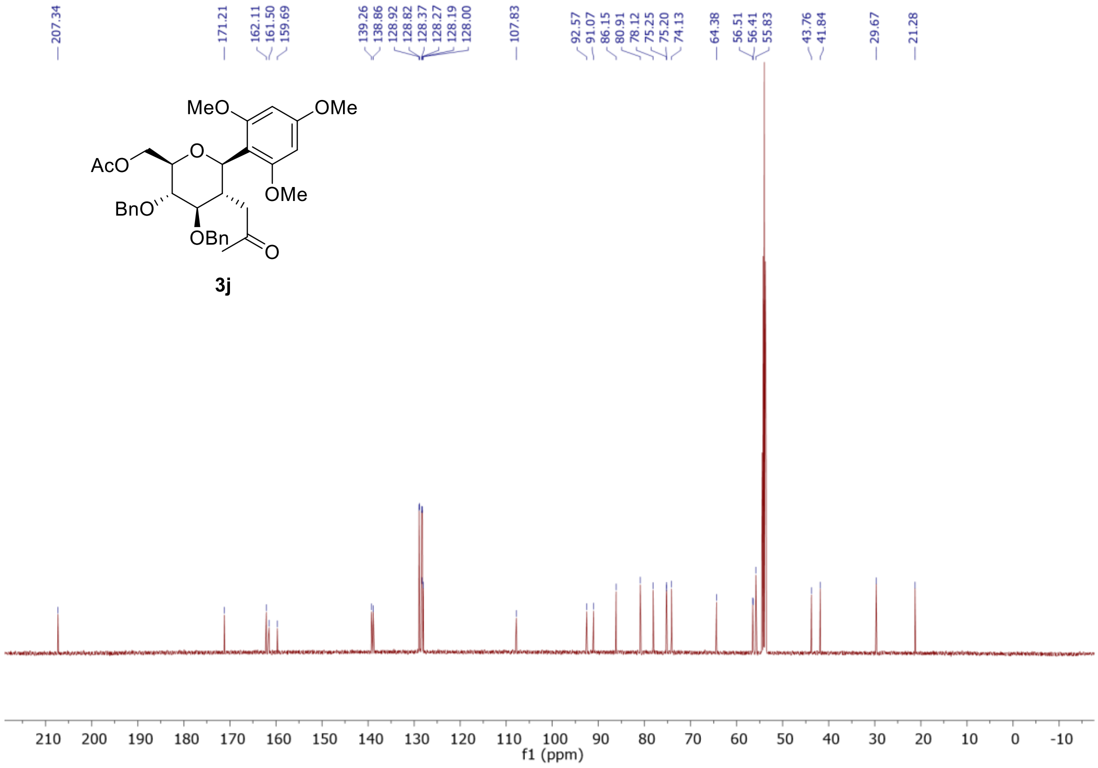

Supplementary Figure S109: ^{13}C NMR spectra for 3j

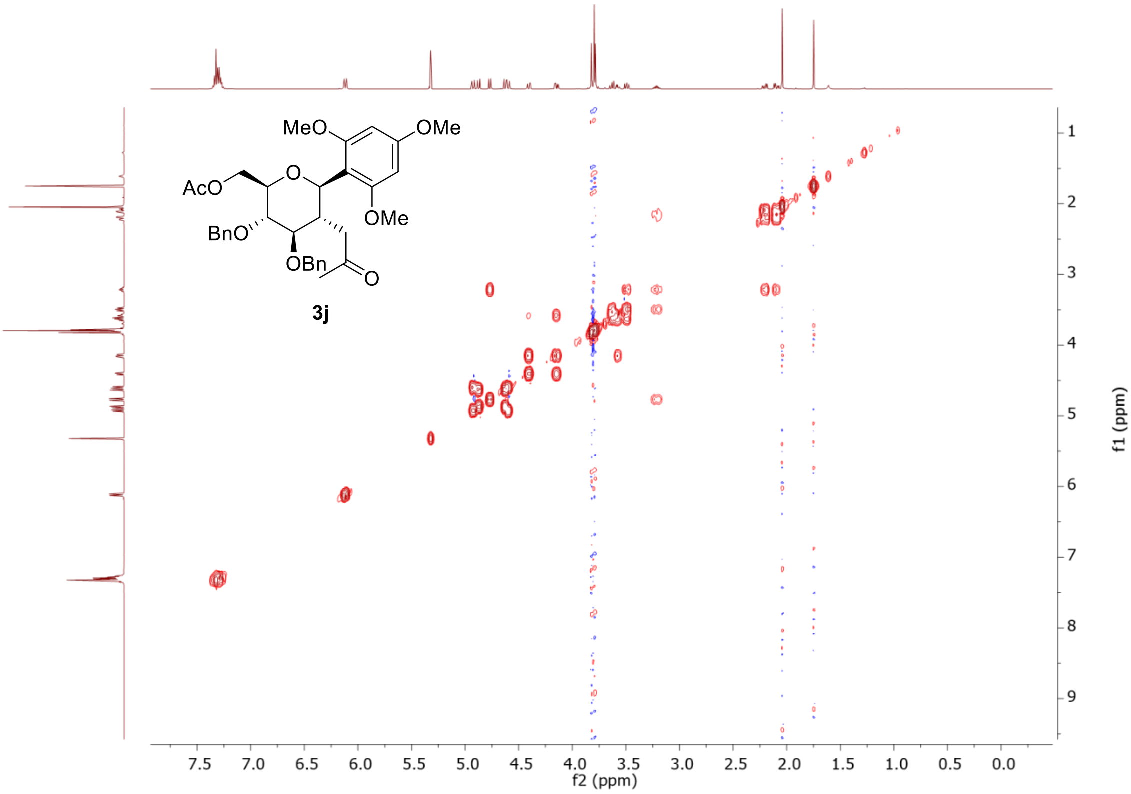

Supplementary Figure S110: COSY spectra for 3j

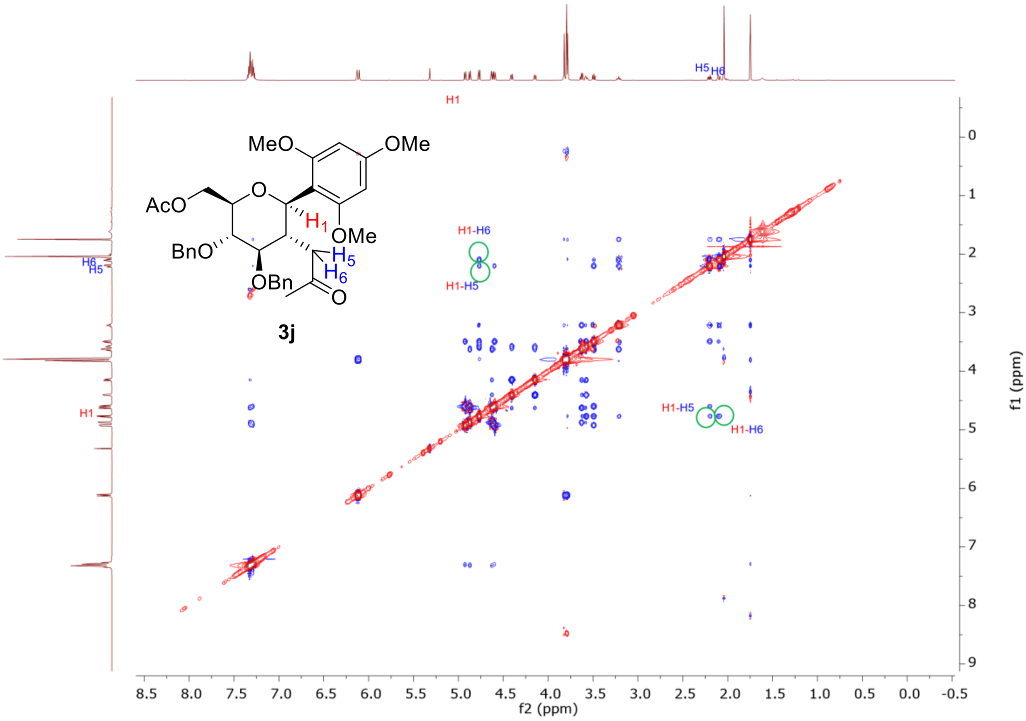

Supplementary Figure S111: NOESY spectra for 3j

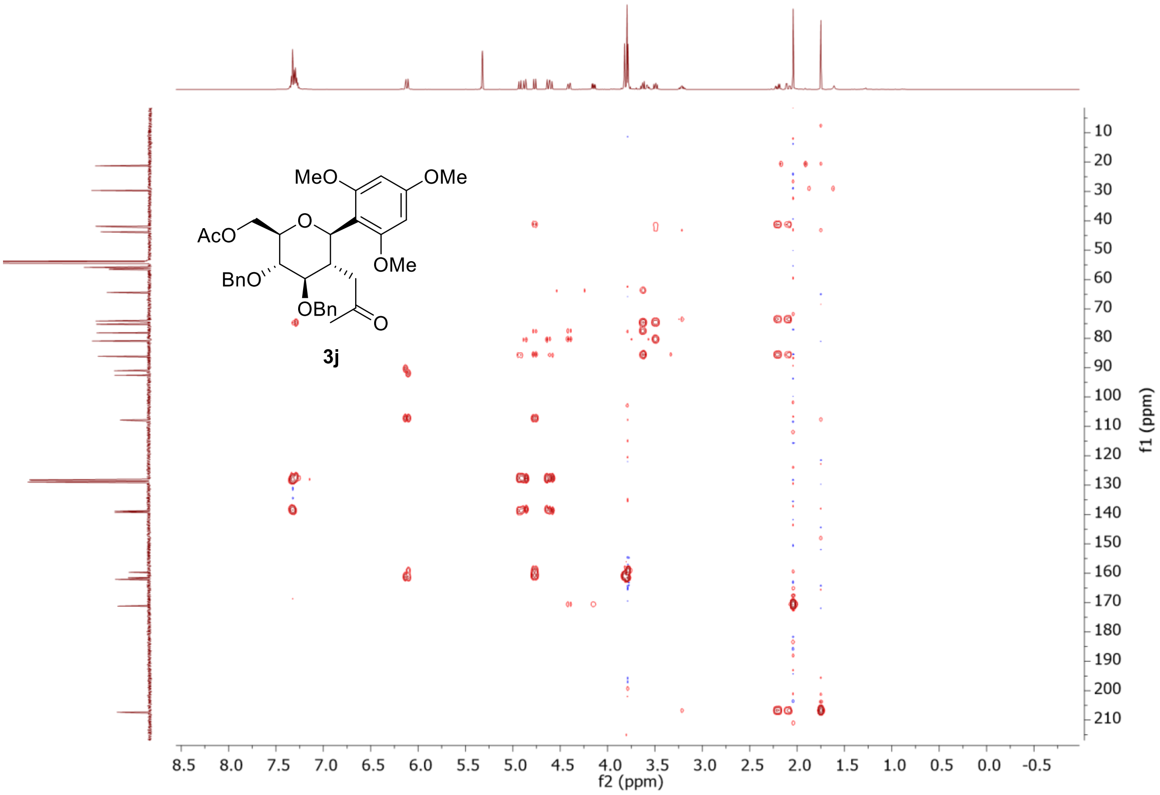

Supplementary Figure S112: HMBC spectra for 3j

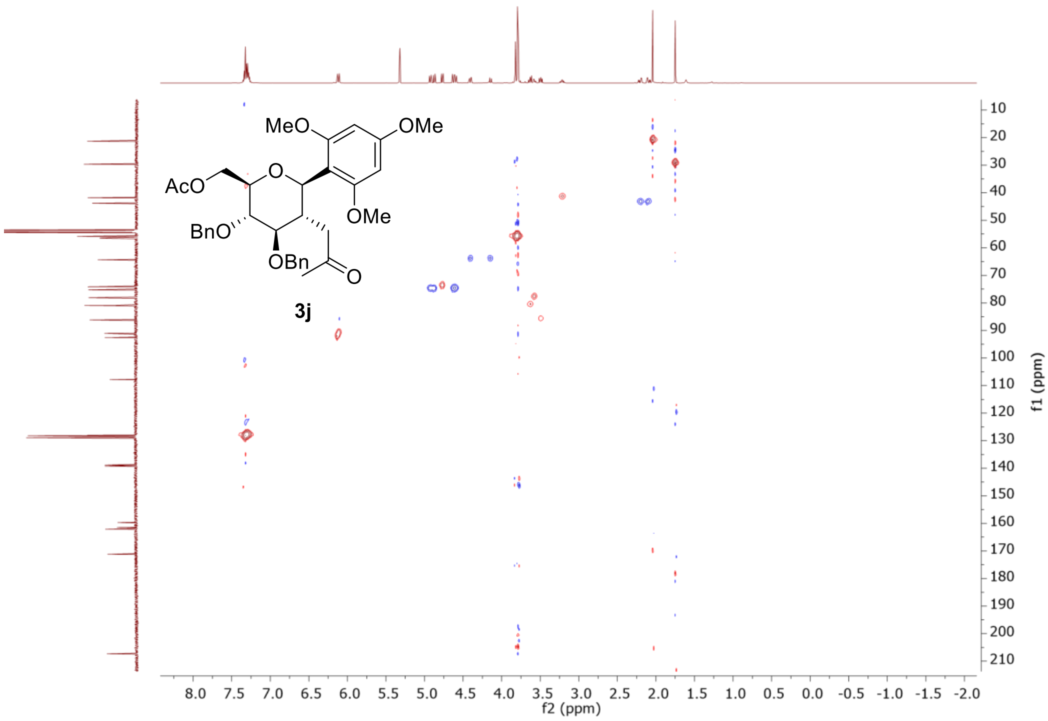

Supplementary Figure S113: HSQC spectra for 3j

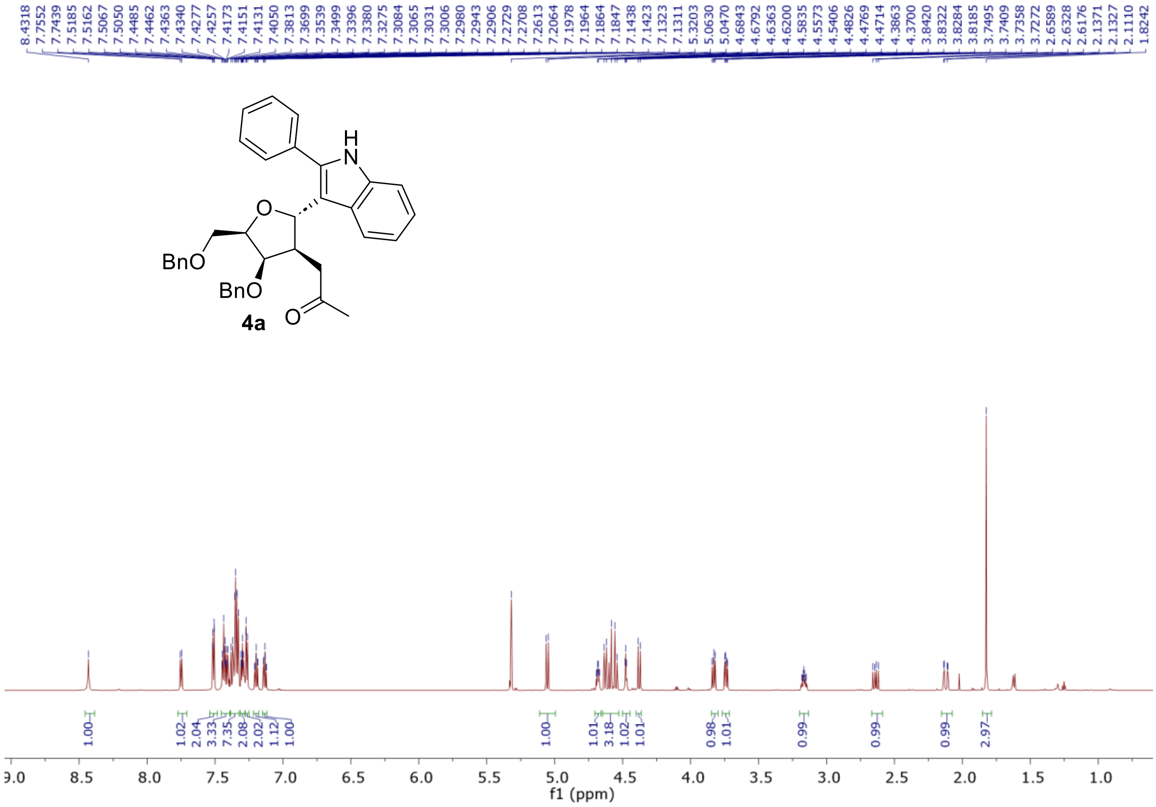

Supplementary Figure S114: ^{1}H NMR spectra for 4a

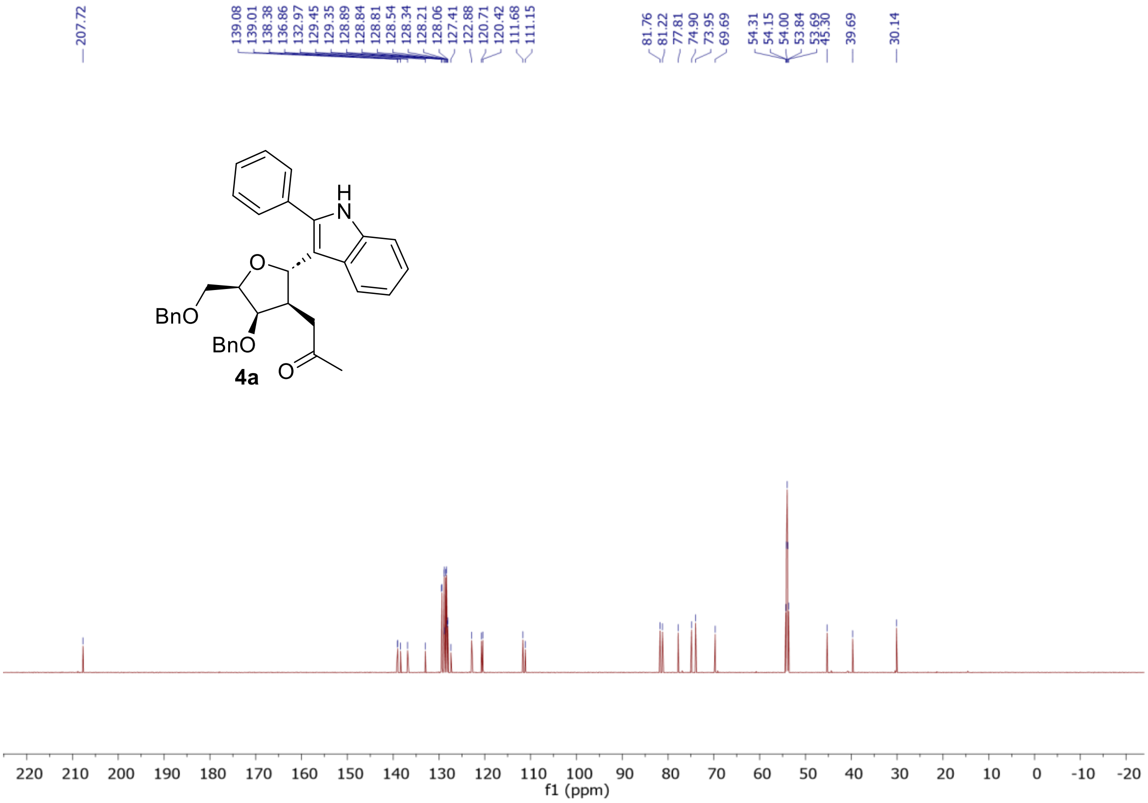

Supplementary Figure S115: ^{13}C NMR spectra for 4a

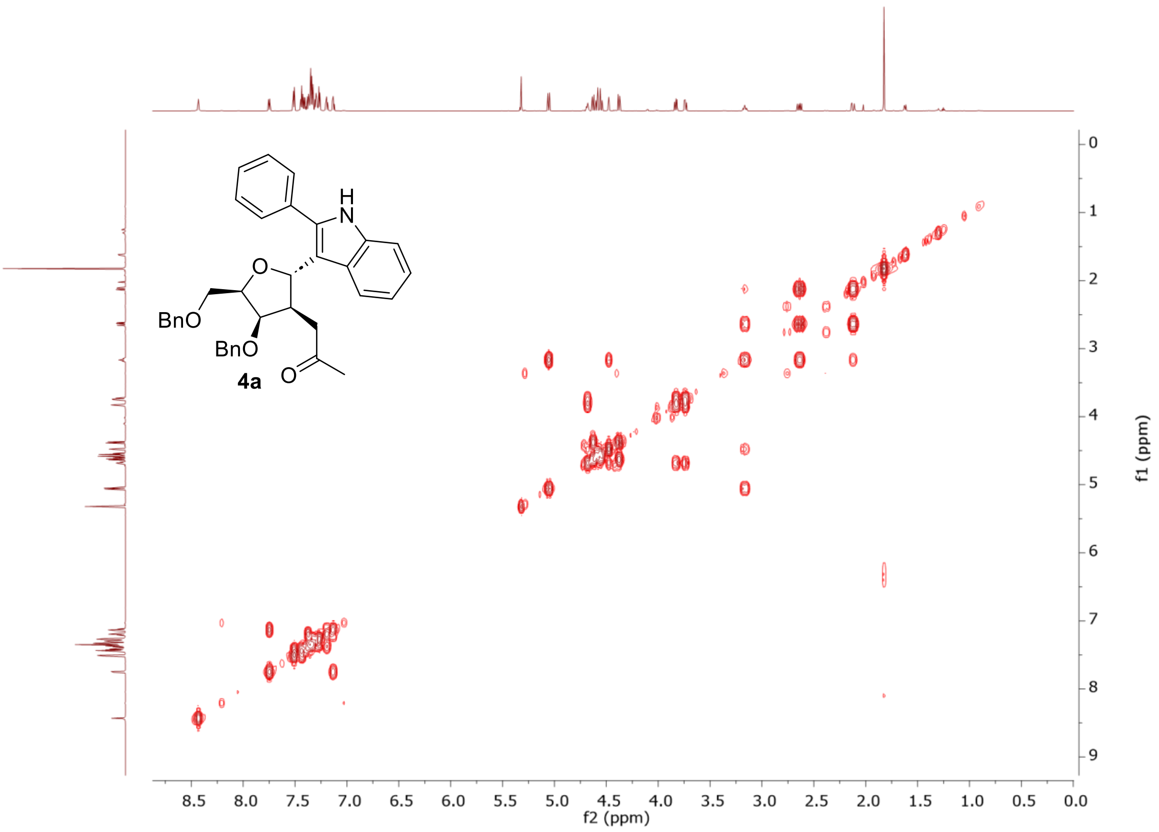

Supplementary Figure S116: COSY spectra for 4a

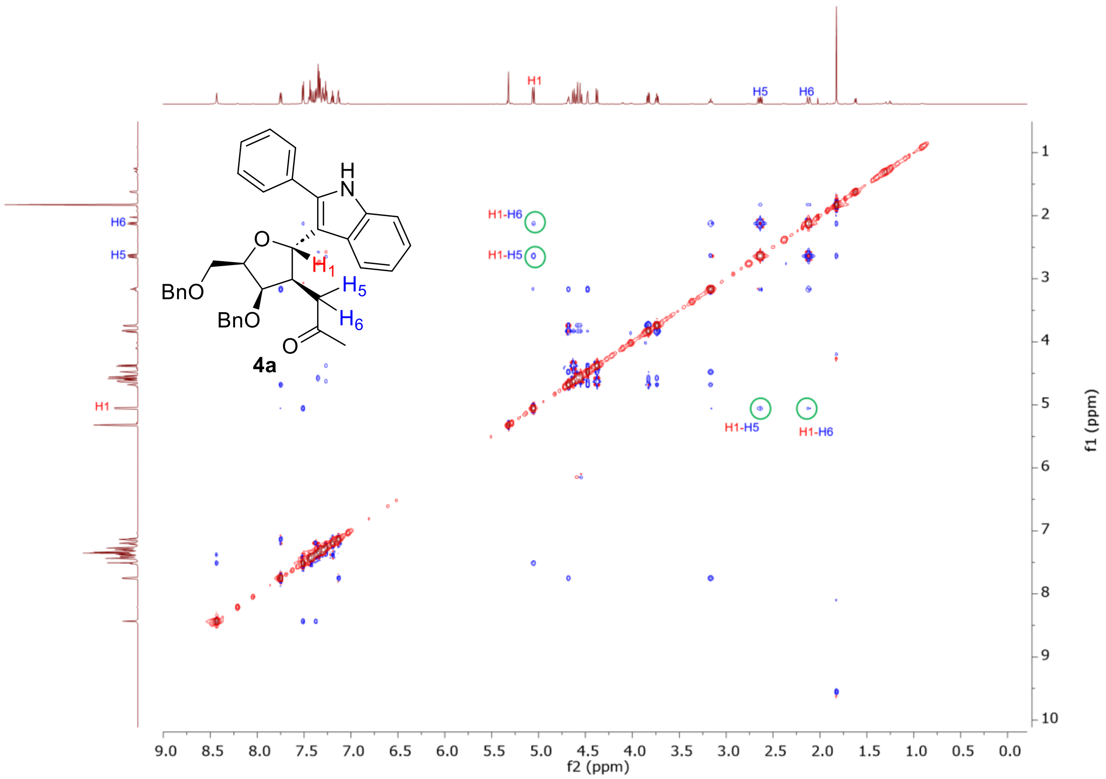

Supplementary Figure S117: NOESY spectra for 4a

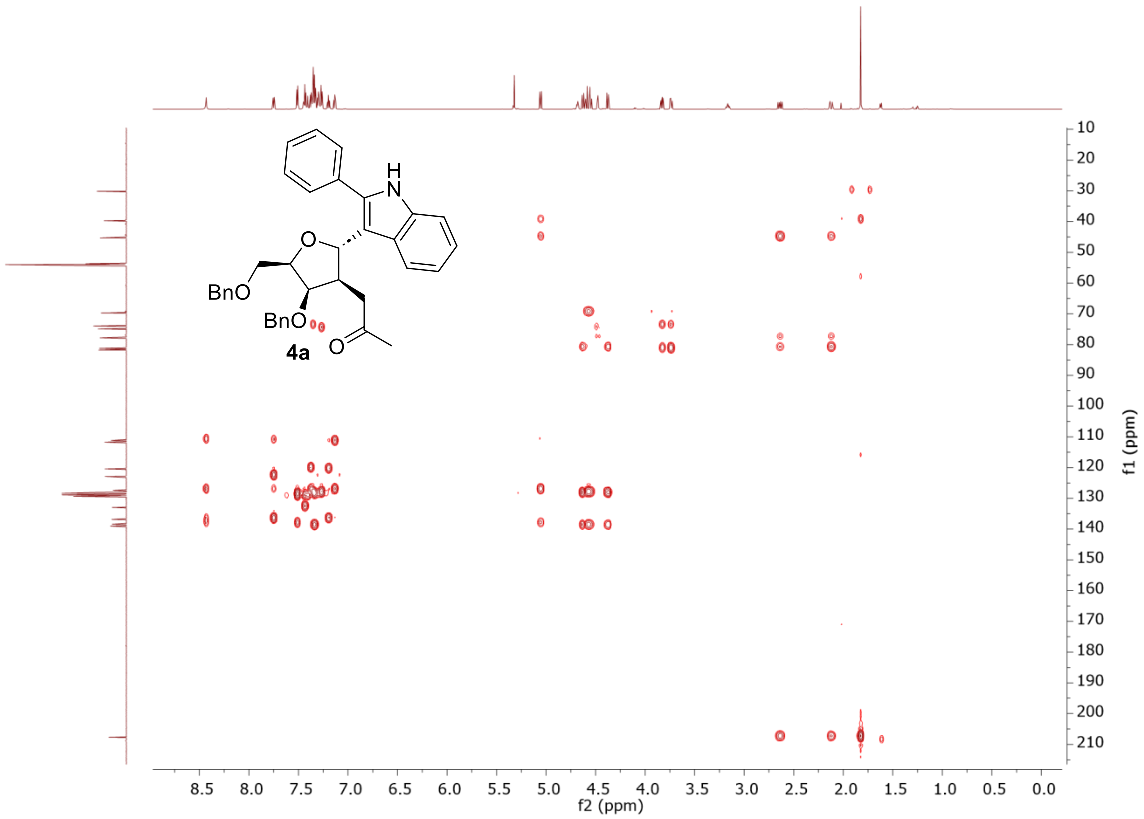

Supplementary Figure S118: HMBC spectra for 4a

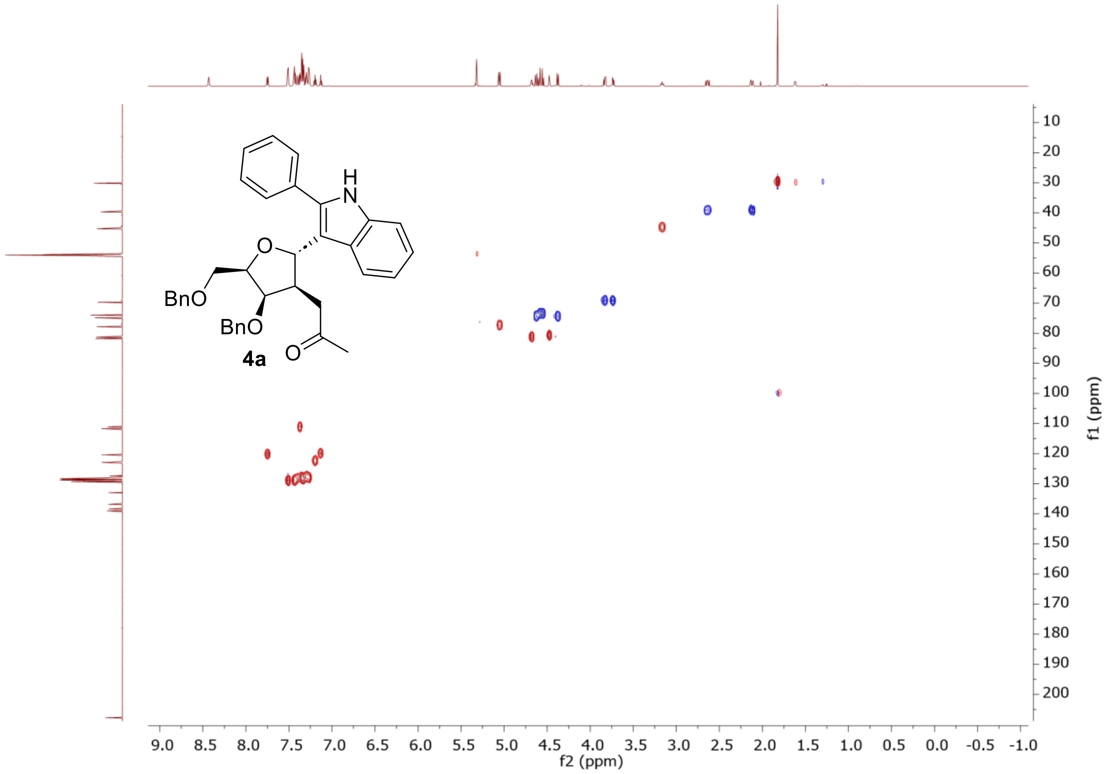

Supplementary Figure S119: HSQC spectra for 4a

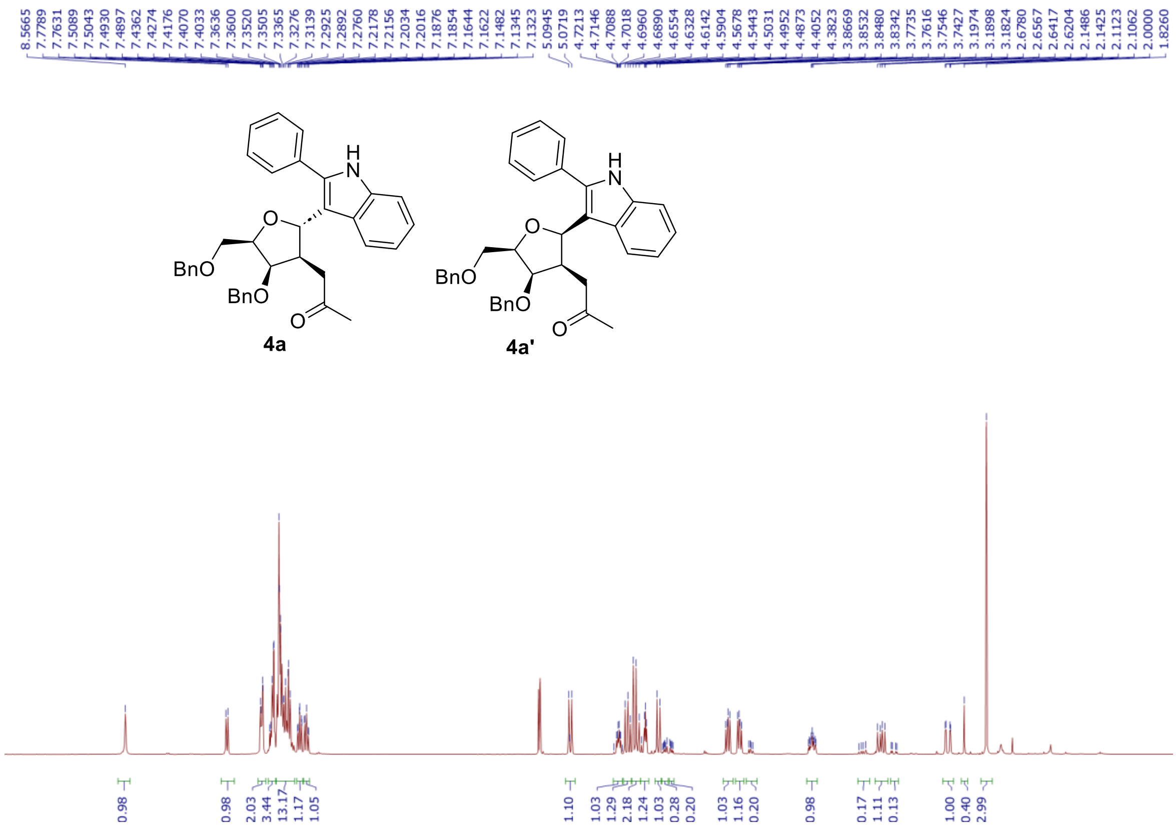

Supplementary Figure S120: ^{1}H NMR spectra for 4a and 4a'

Note: the mixture (4a and 4a') was isolated under XB catalysis (see Table S2, Entry 3)

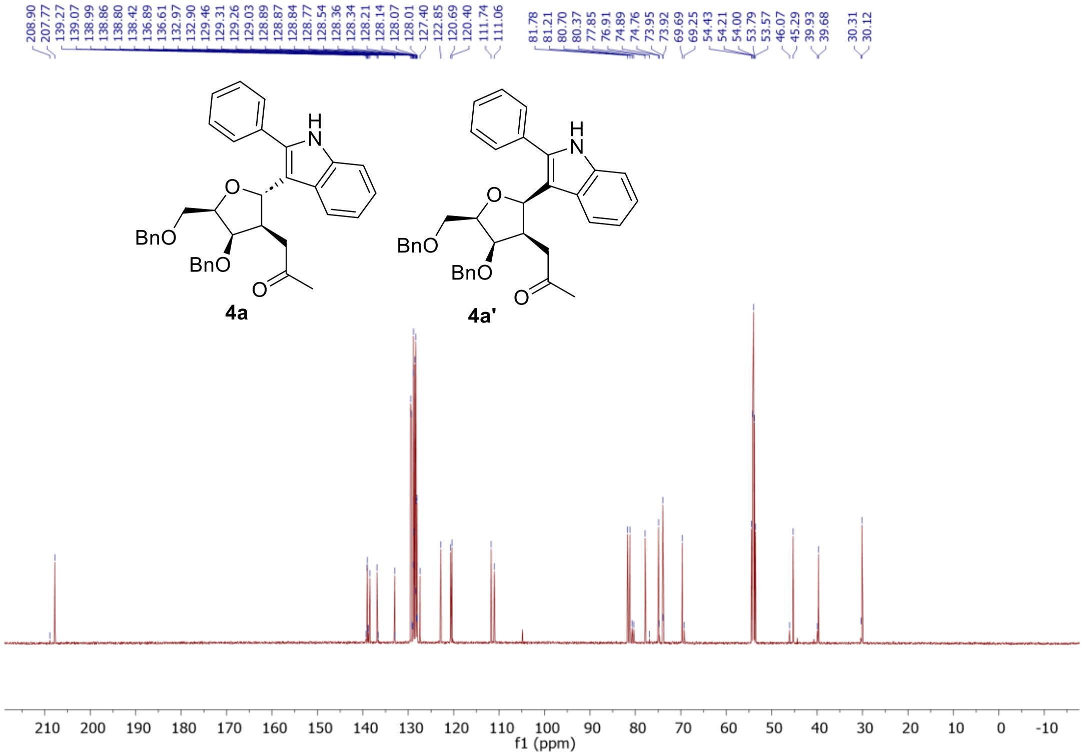

Supplementary Figure S121: ^{13}C NMR spectra for 4a and 4a'

Note: the mixture (4a and 4a') was isolated under XB catalysis (see Table S2, Entry 3)

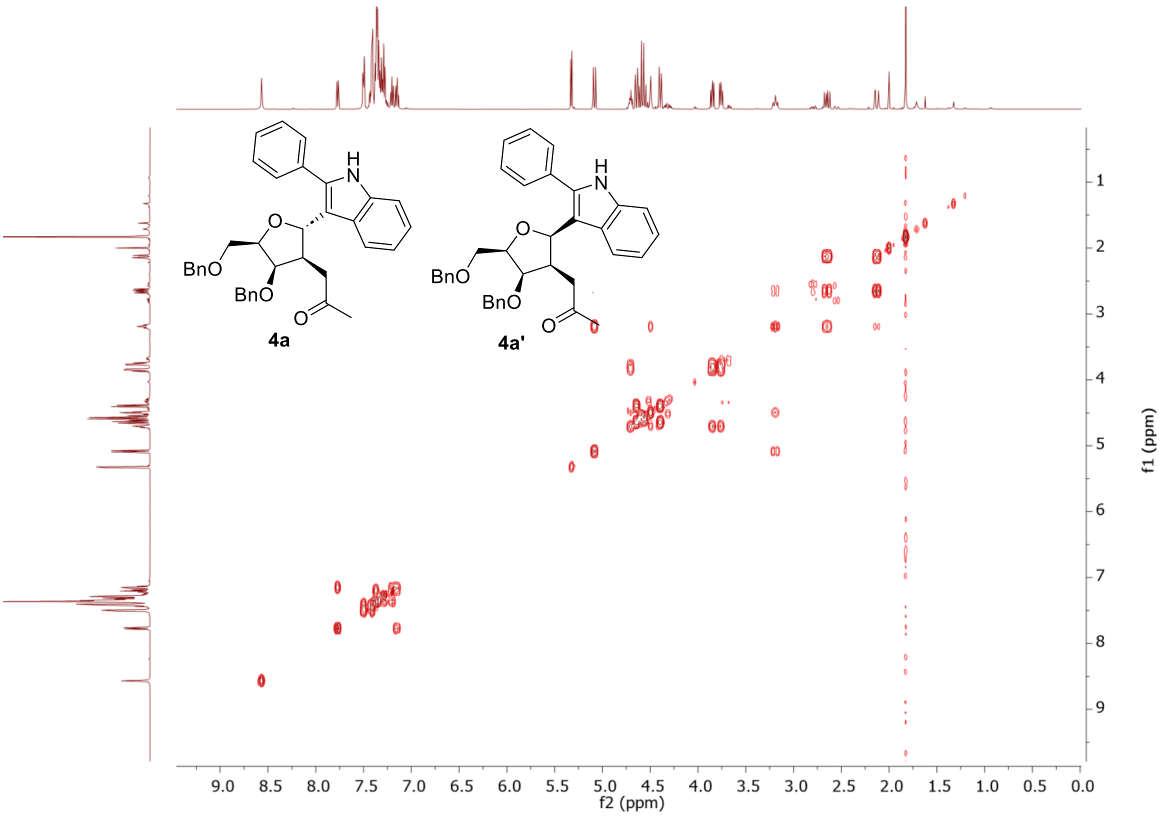

Supplementary Figure S122: COSY spectra for 4a and 4a’

Note: the mixture (4a and 4a’) was isolated under XB catalysis (see Table S2, Entry 3)

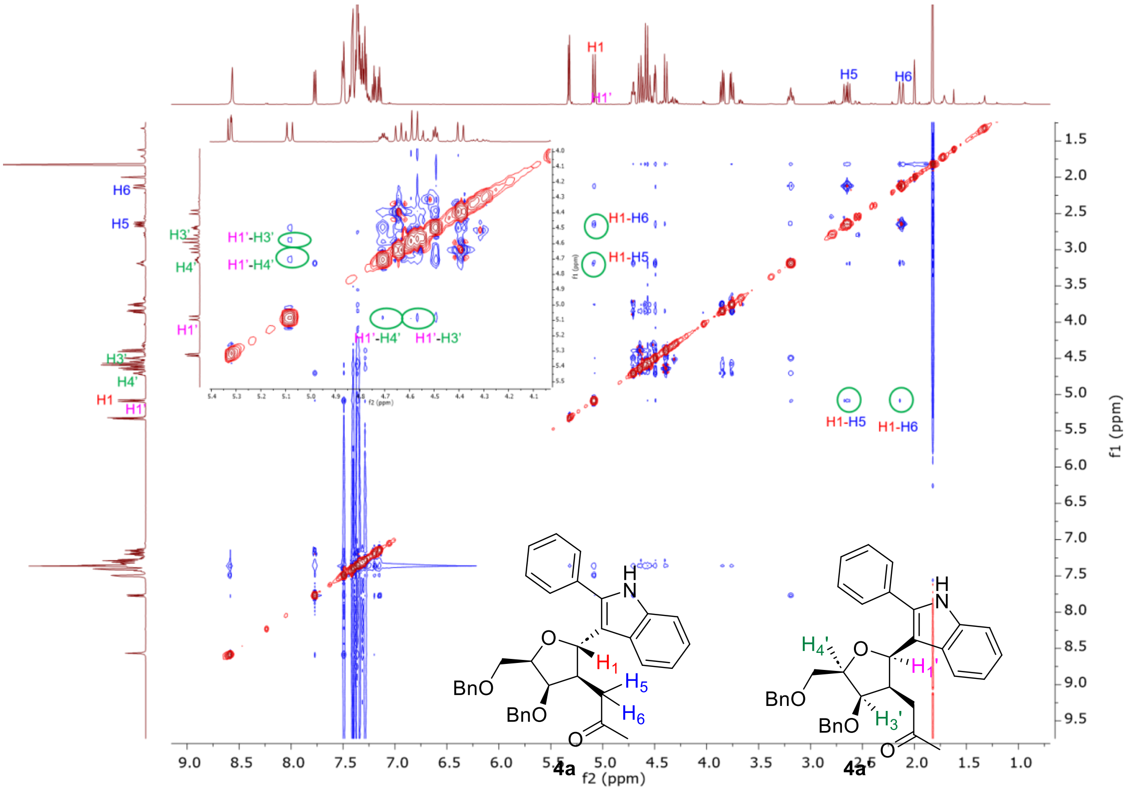

Supplementary Figure S123: NOESY spectra for 4a and 4a’

Note: the mixture (4a and 4a’) was isolated under XB catalysis (see Table S2, Entry 3)

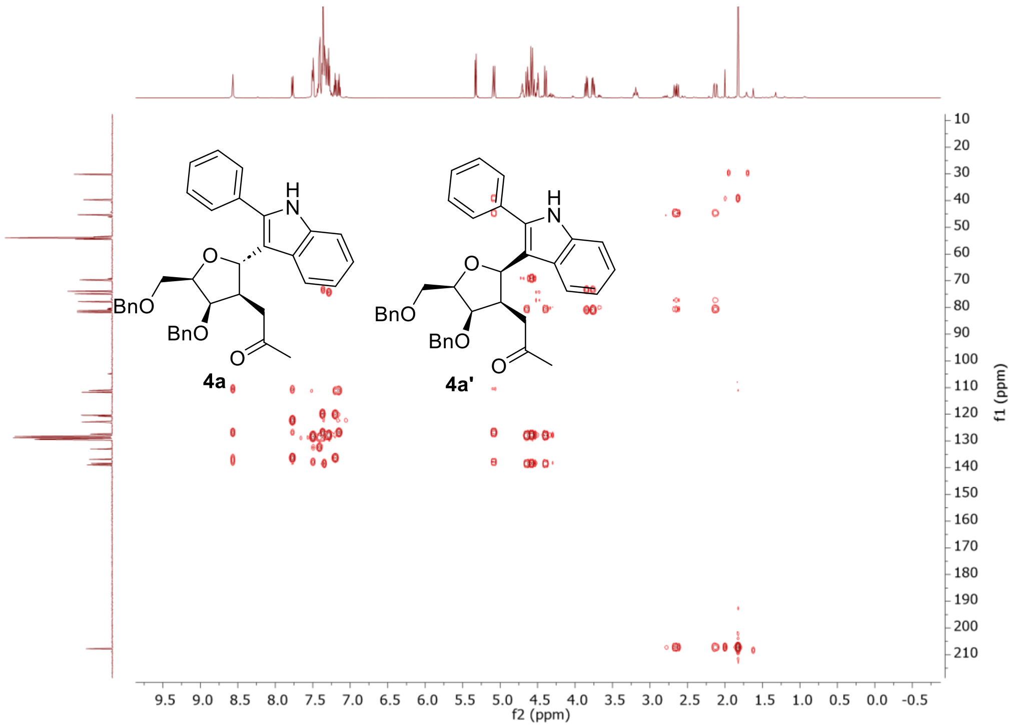

Supplementary Figure S124: HMBC spectra for 4a and 4a'

Note: the mixture (4a and 4a') was isolated under XB catalysis (see Table S2, Entry 3)

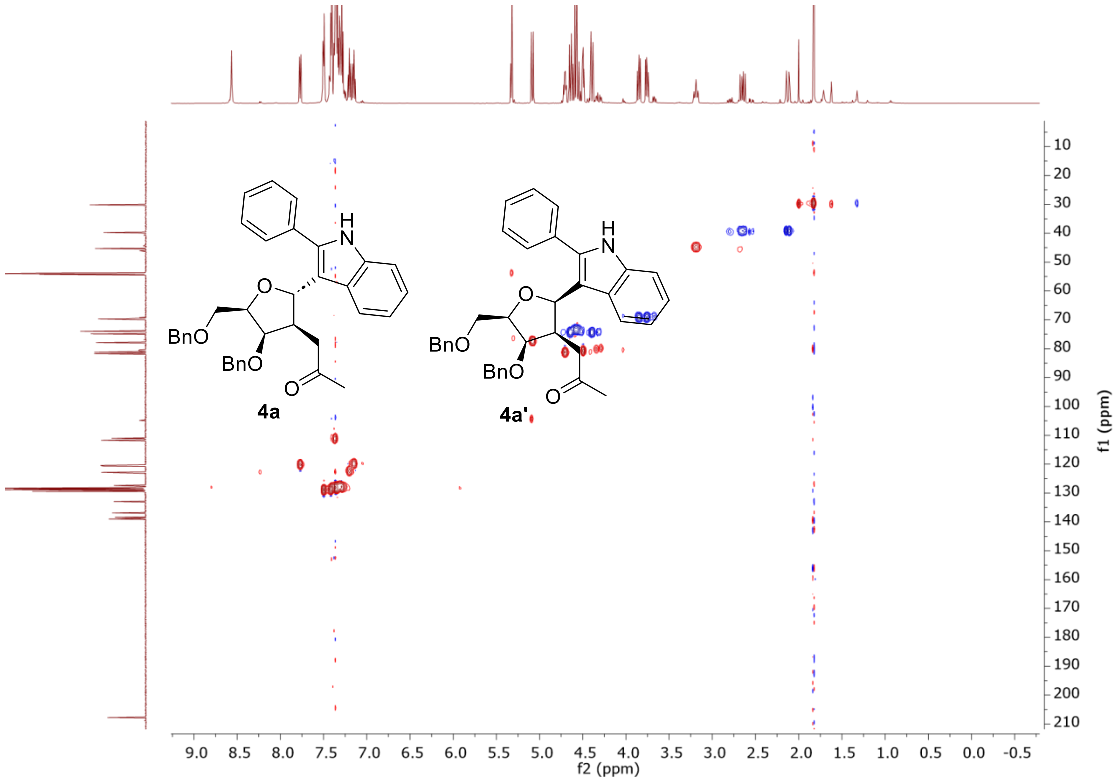

Supplementary Figure S125: HSQC spectra for 4a and 4a'

Note: the mixture (4a and 4a') was isolated under XB catalysis (see Tables S2, Entry 3)

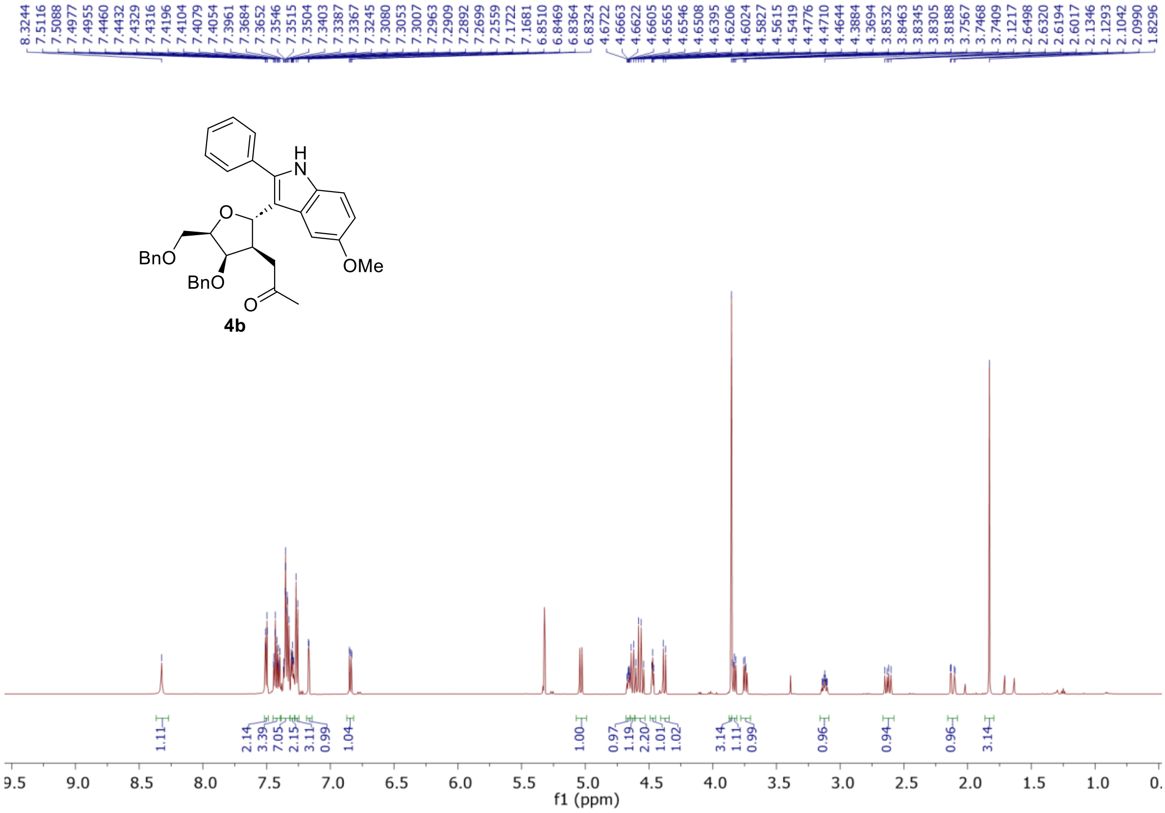

Supplementary Figure S126: ^{1}H NMR spectra for 4b

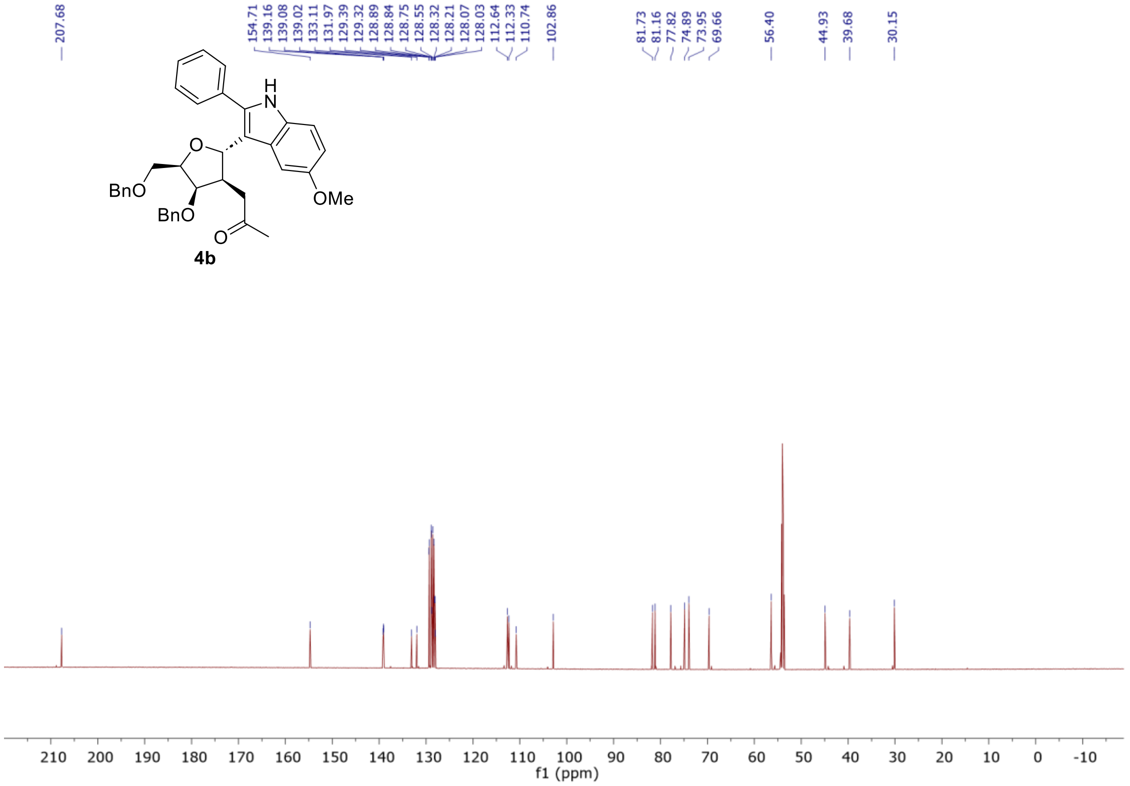

Supplementary Figure S127: ^{13}C NMR spectra for 4b

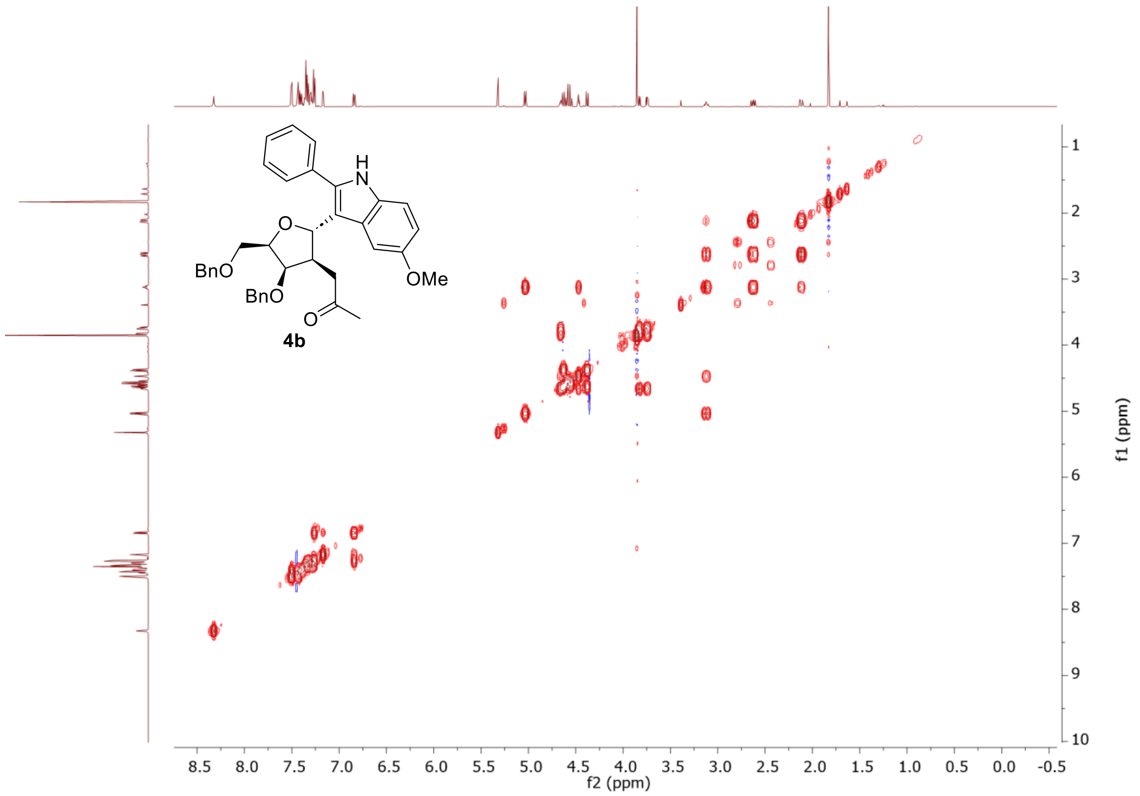

Supplementary Figure S128: COSY spectra for 4b

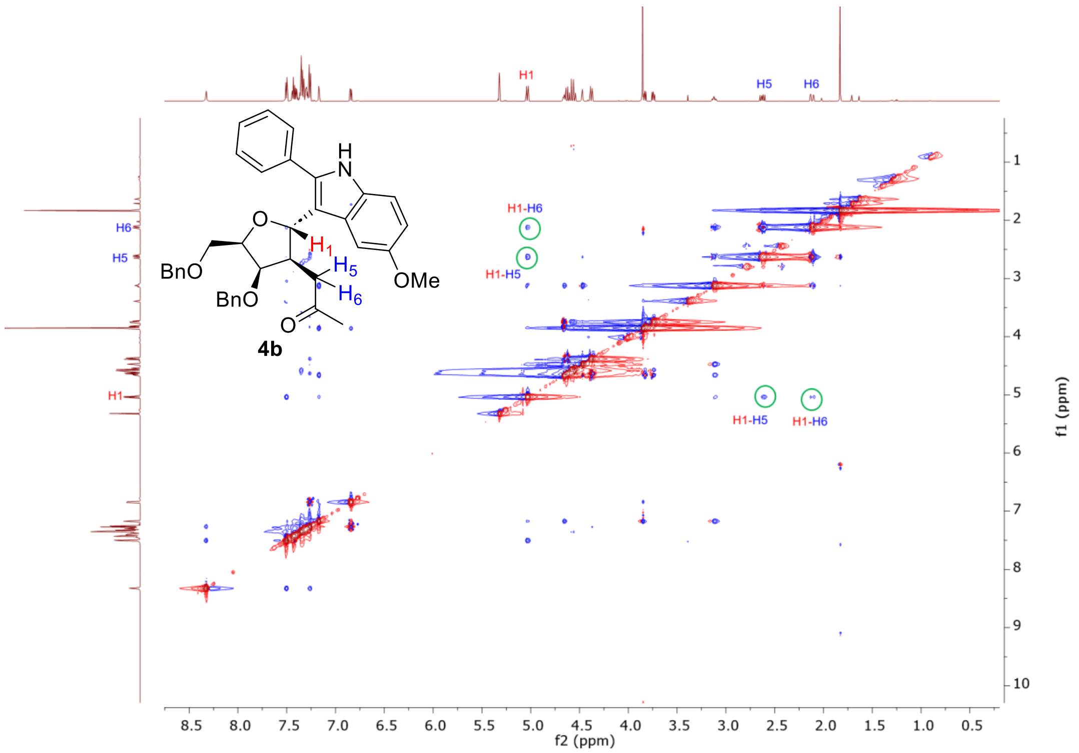

Supplementary Figure S129: NOESY spectra for 4b

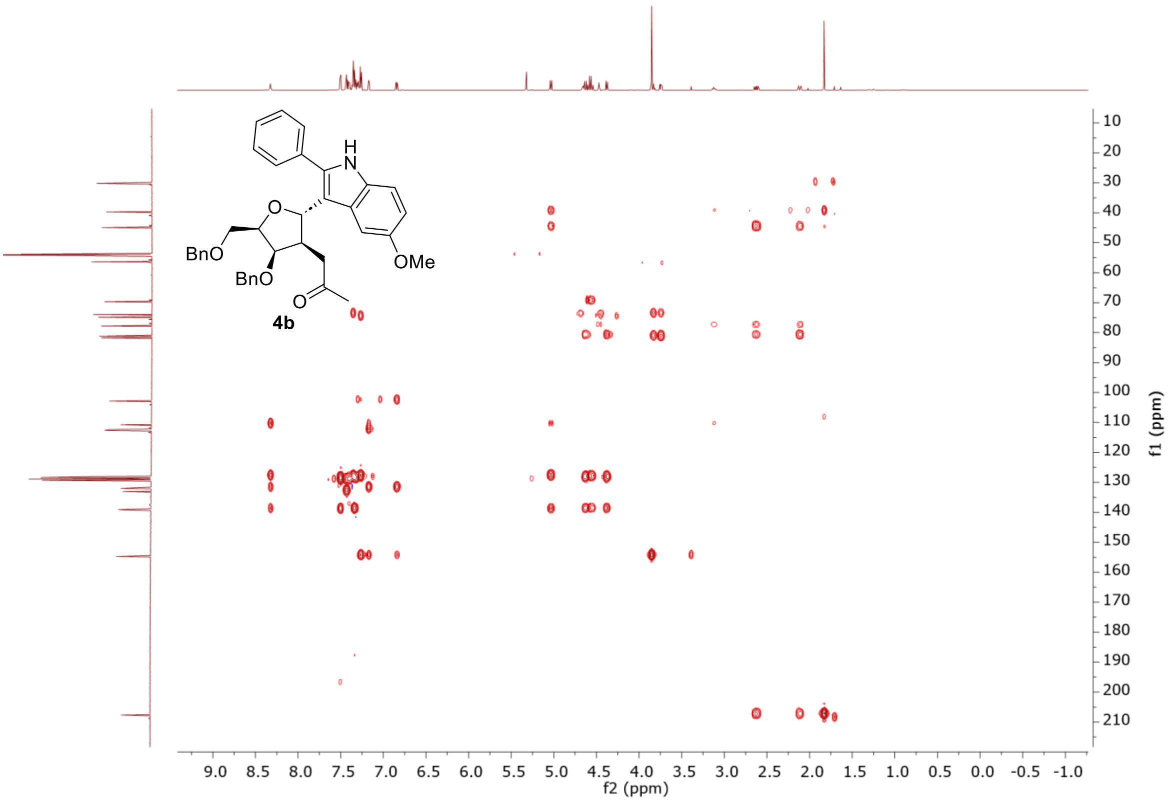

Supplementary Figure S130: HMBC spectra for 4b

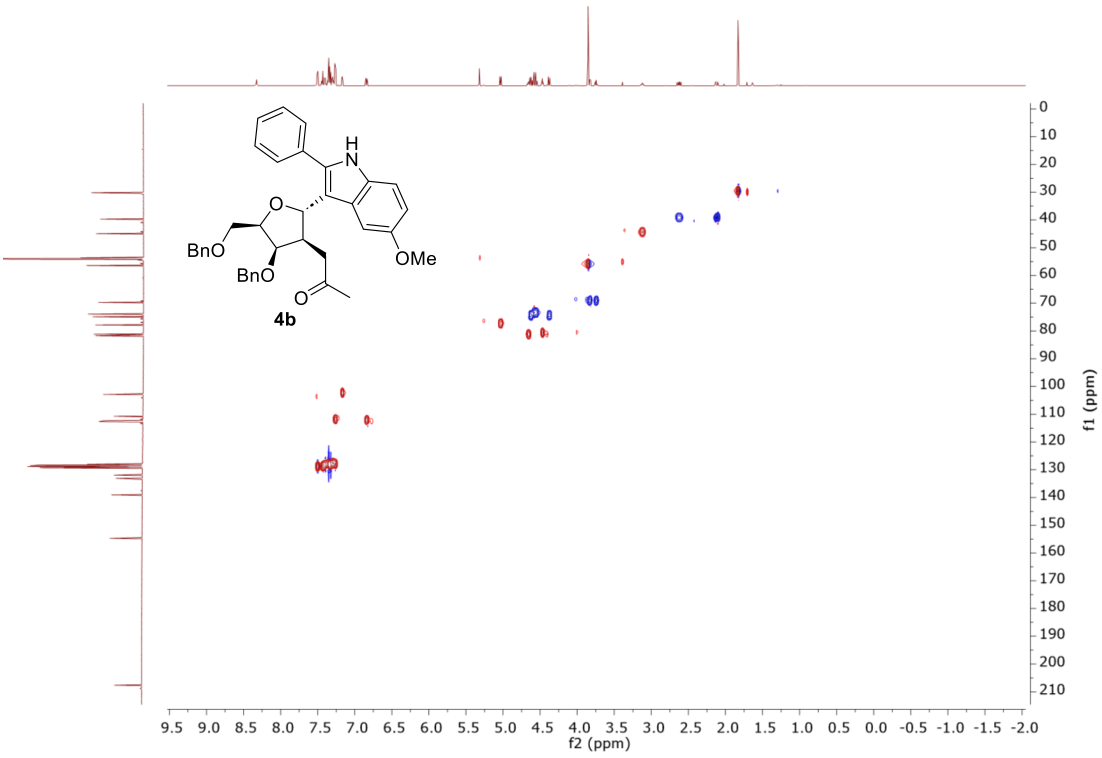

Supplementary Figure S131: HSQC spectra for 4b

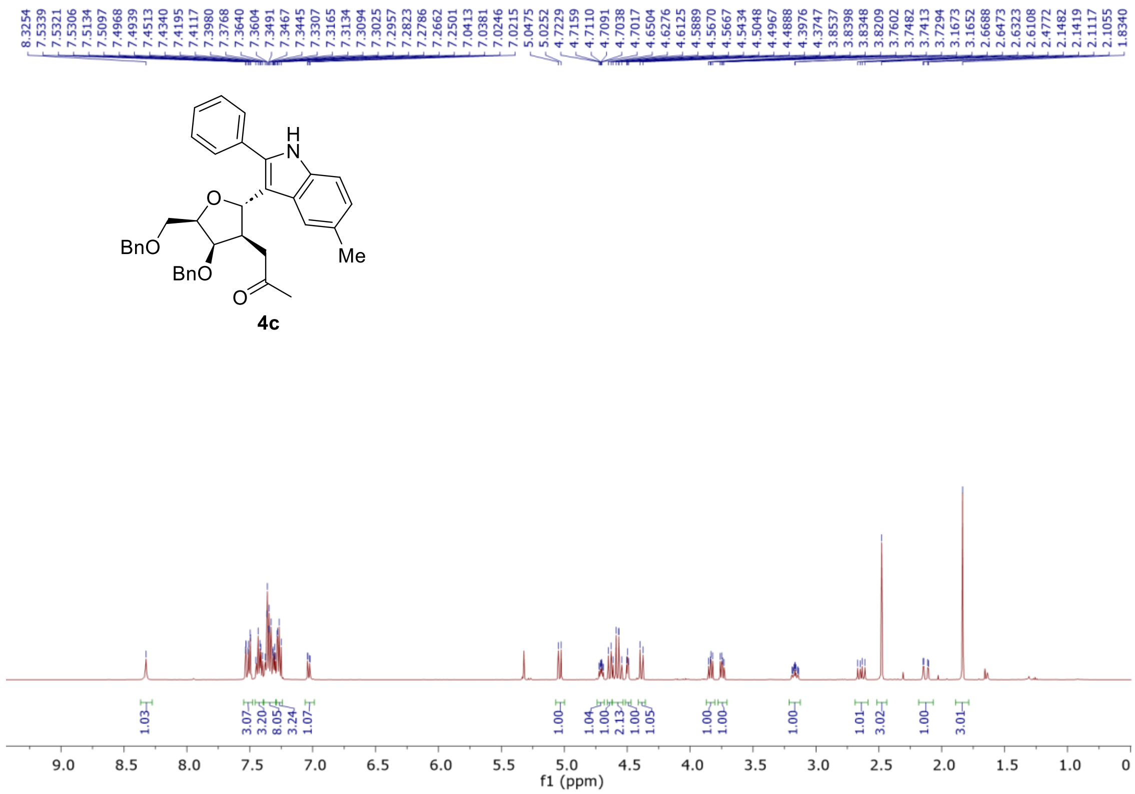

Supplementary Figure S132: ^{1}H NMR spectra for 4c

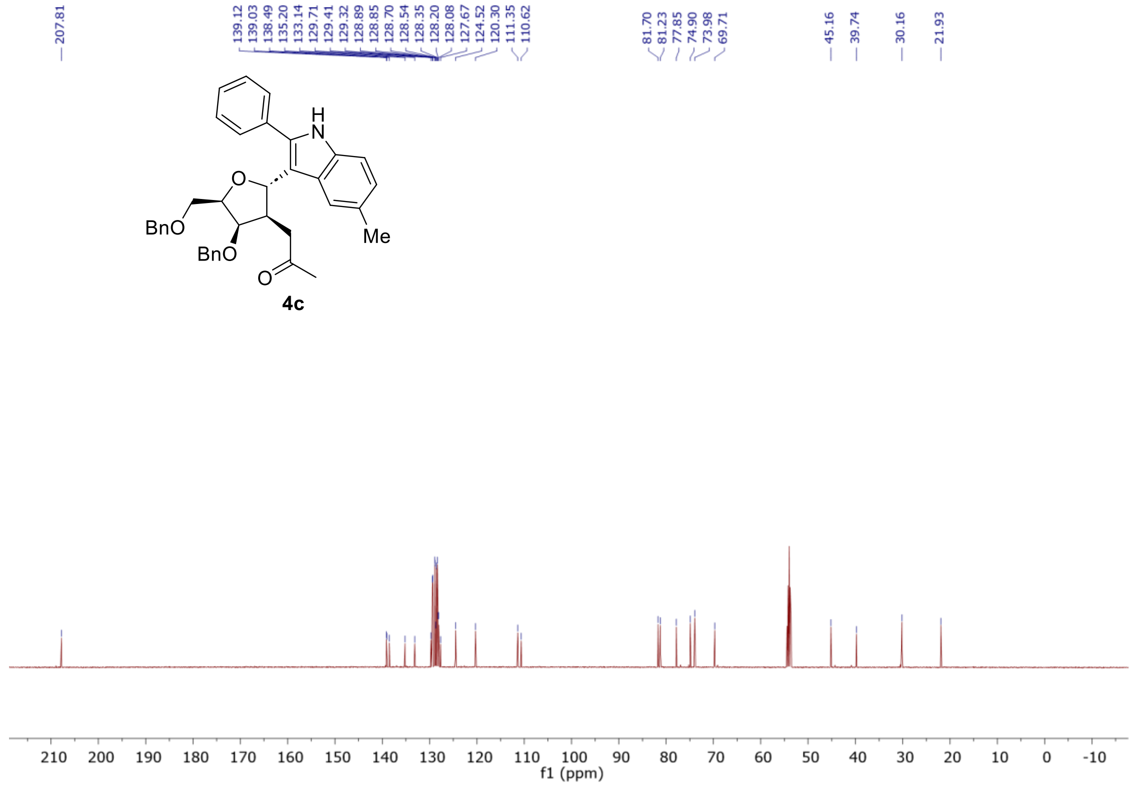

Supplementary Figure S133: ^{13}C NMR spectra for 4c

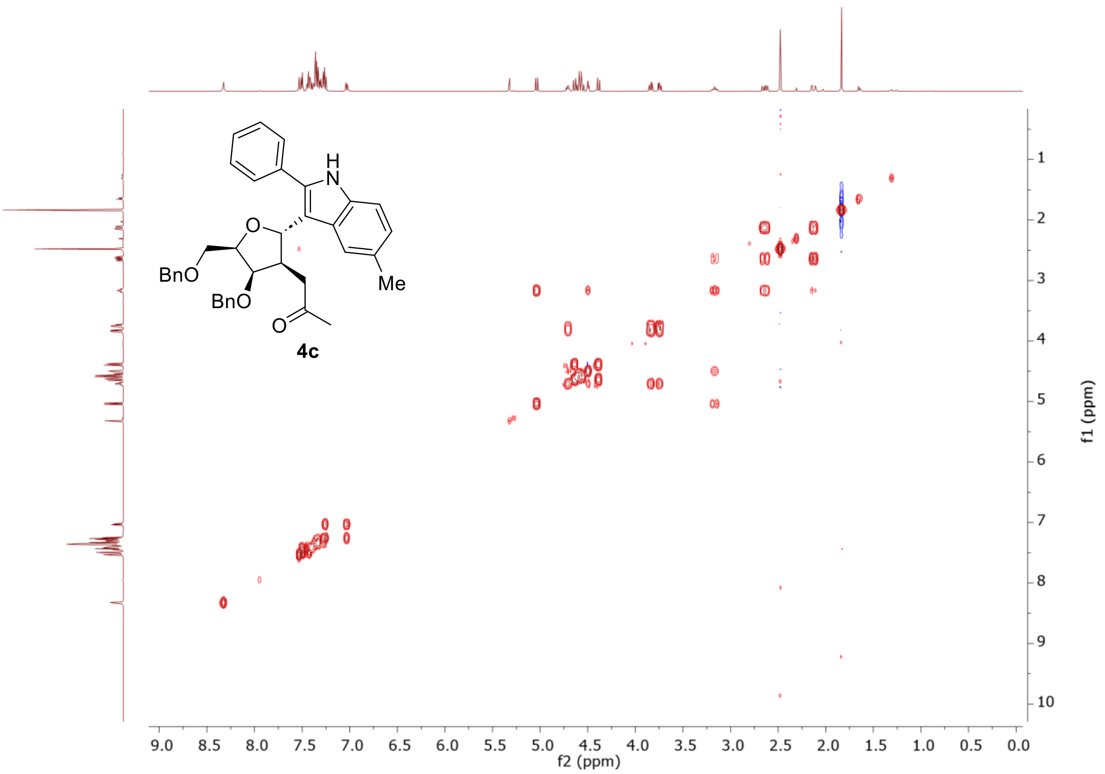

Supplementary Figure S134: COSY NMR spectra for 4c

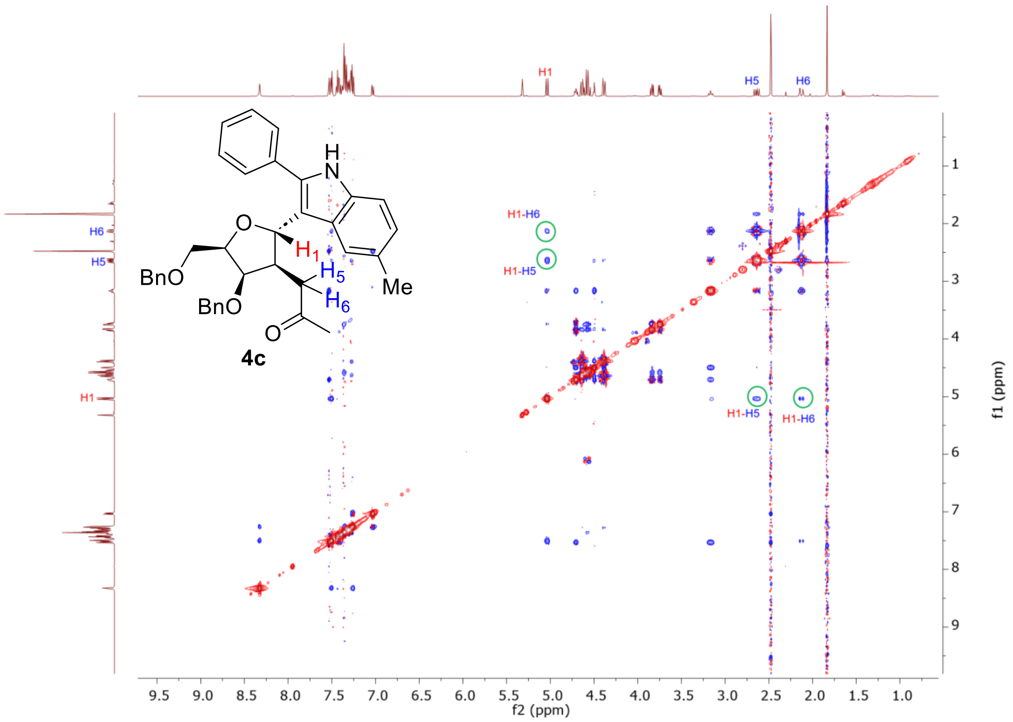

Supplementary Figure S135: NOESY NMR spectra for 4c

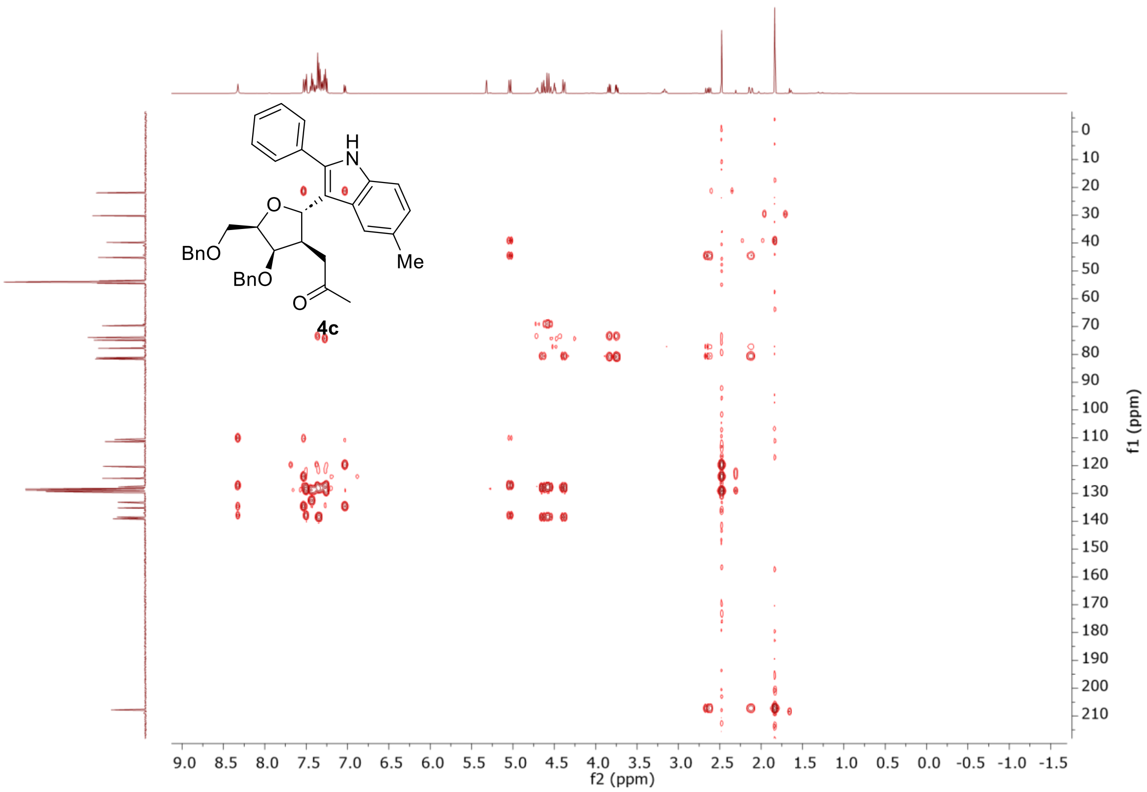

Supplementary Figure S136: HMBC NMR spectra for 4c

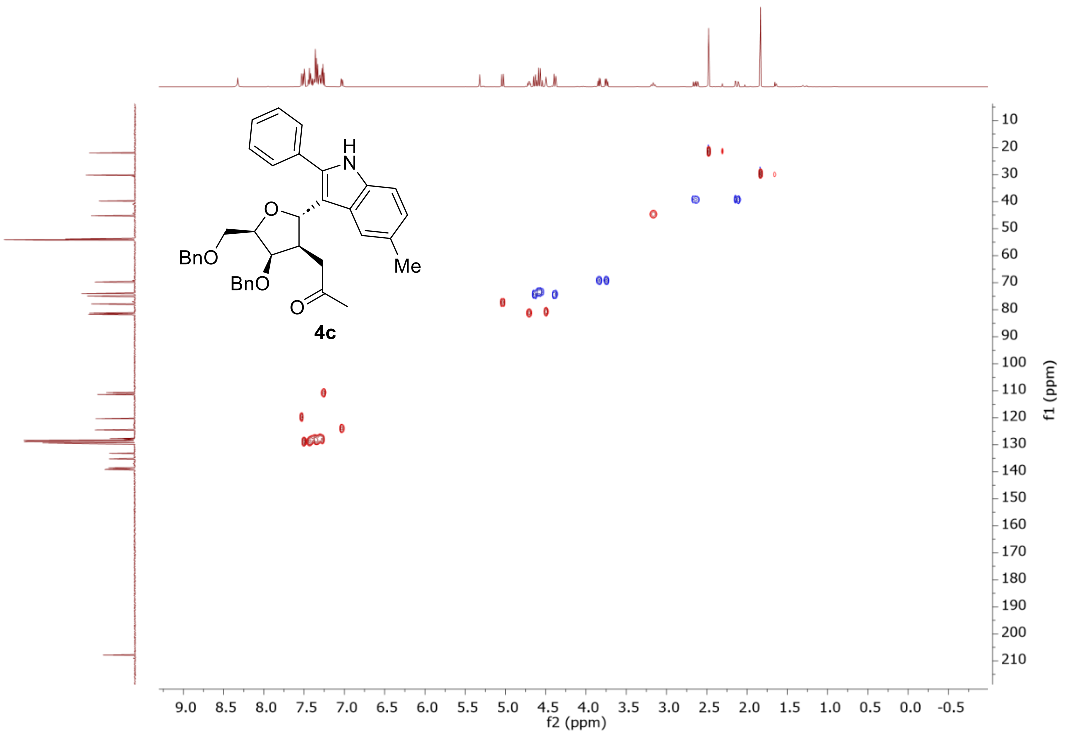

Supplementary Figure S137: HSQC NMR spectra for 4c

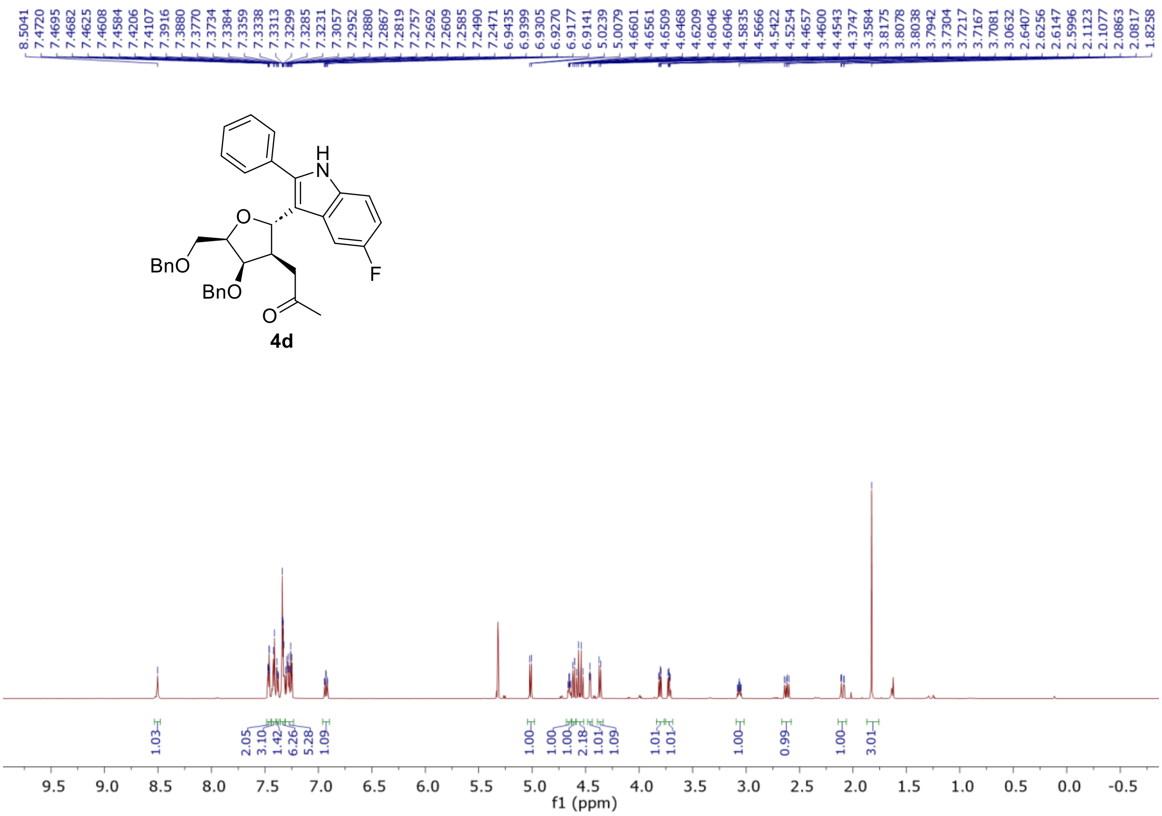

Supplementary Figure S138: ^{1}H NMR spectra for 4d

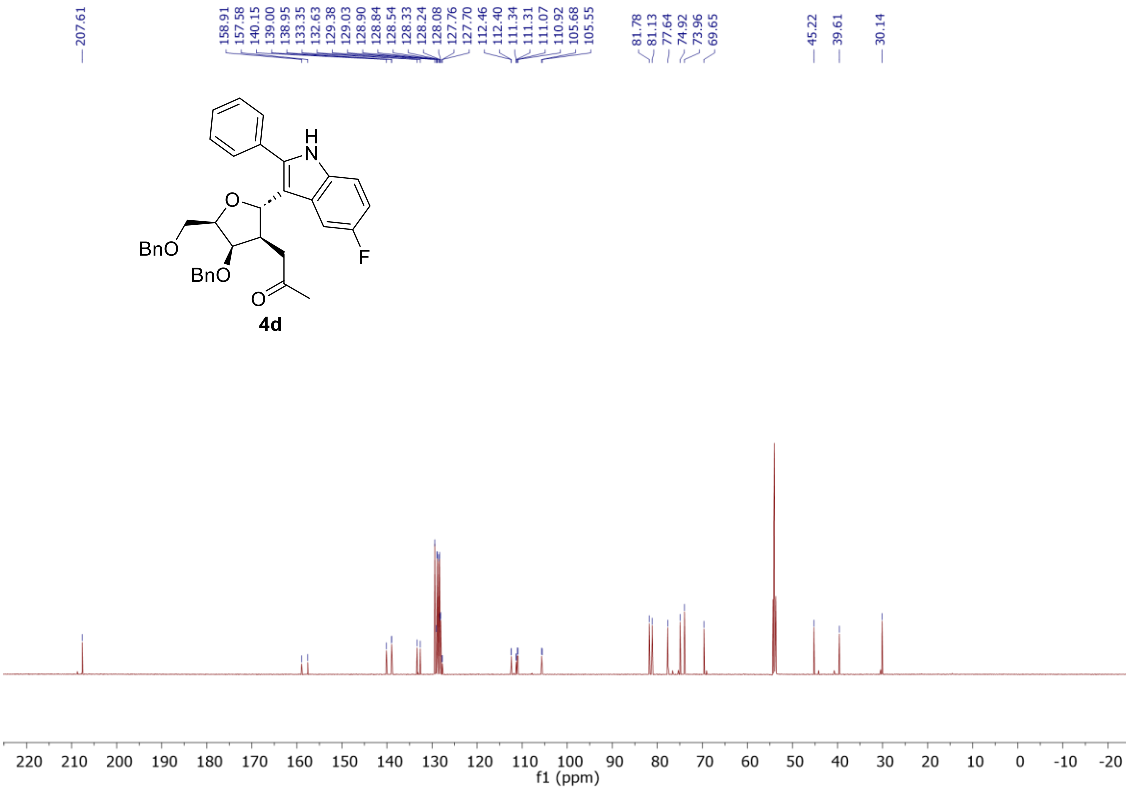

Supplementary Figure S139: ^{13}C NMR spectra for 4d

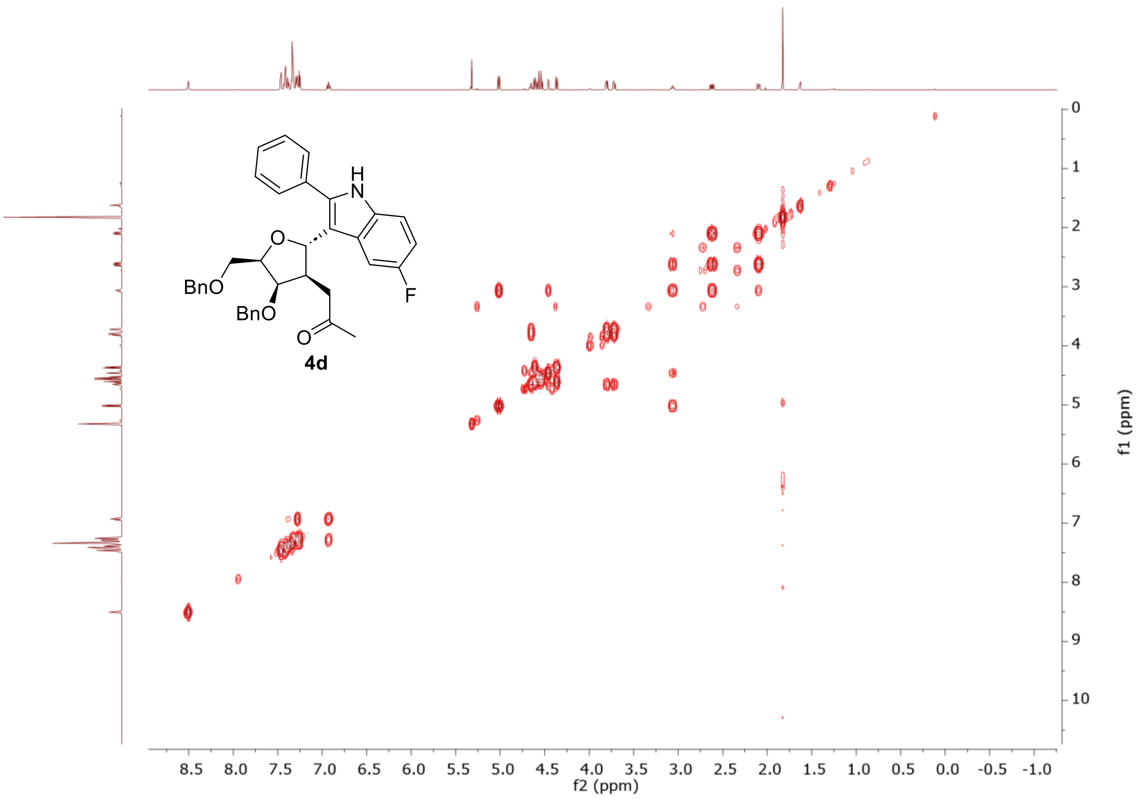

Supplementary Figure S140: COSY spectra for 4d

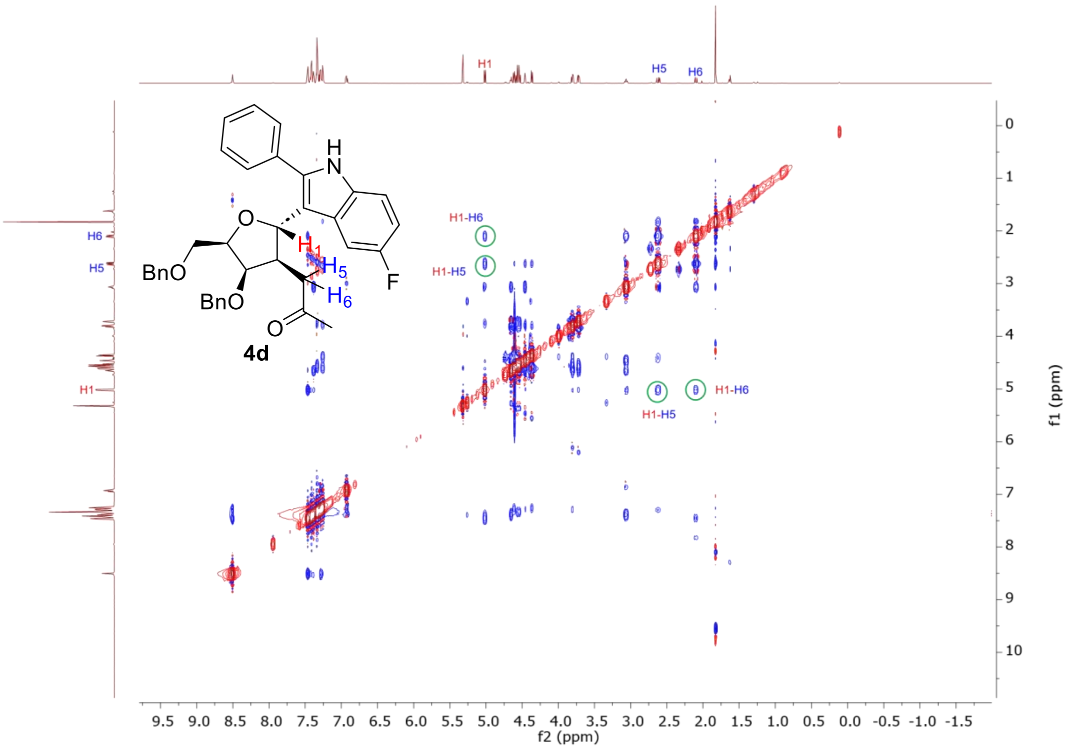

Supplementary Figure S141: NOESY spectra for 4d

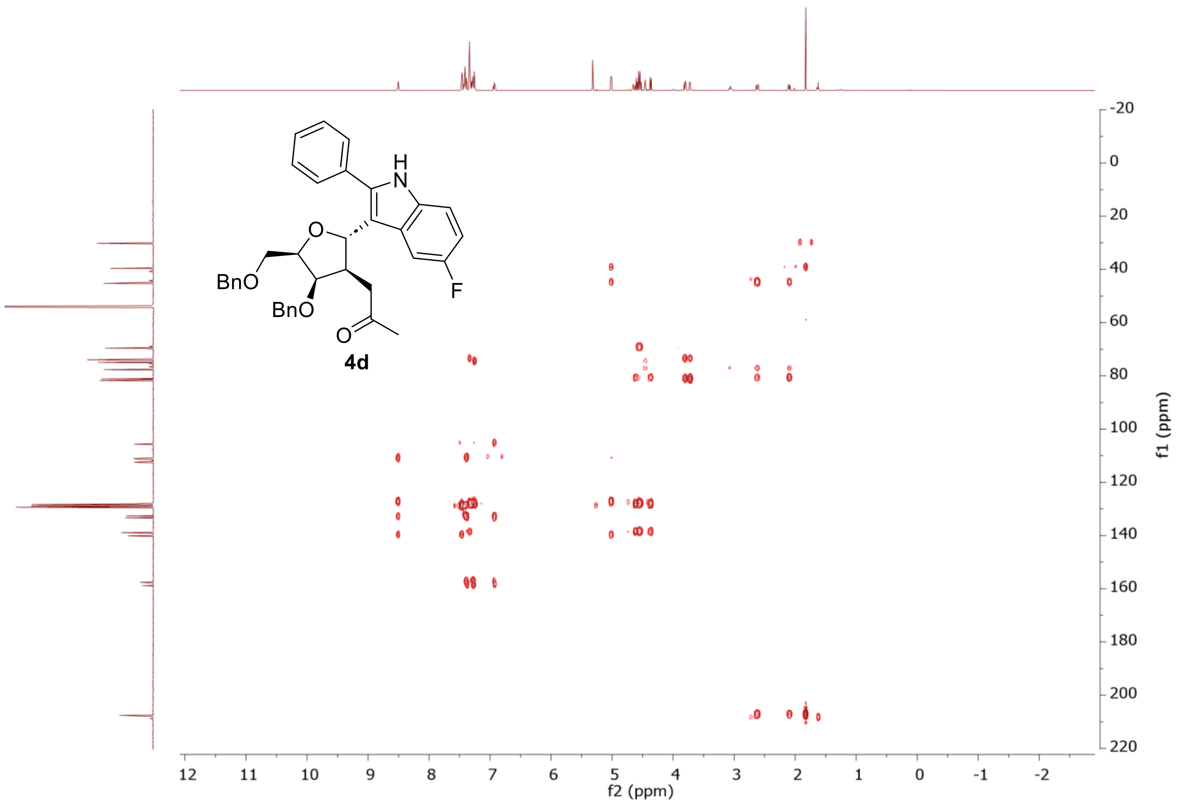

Supplementary Figure S142: HMBC spectra for 4d

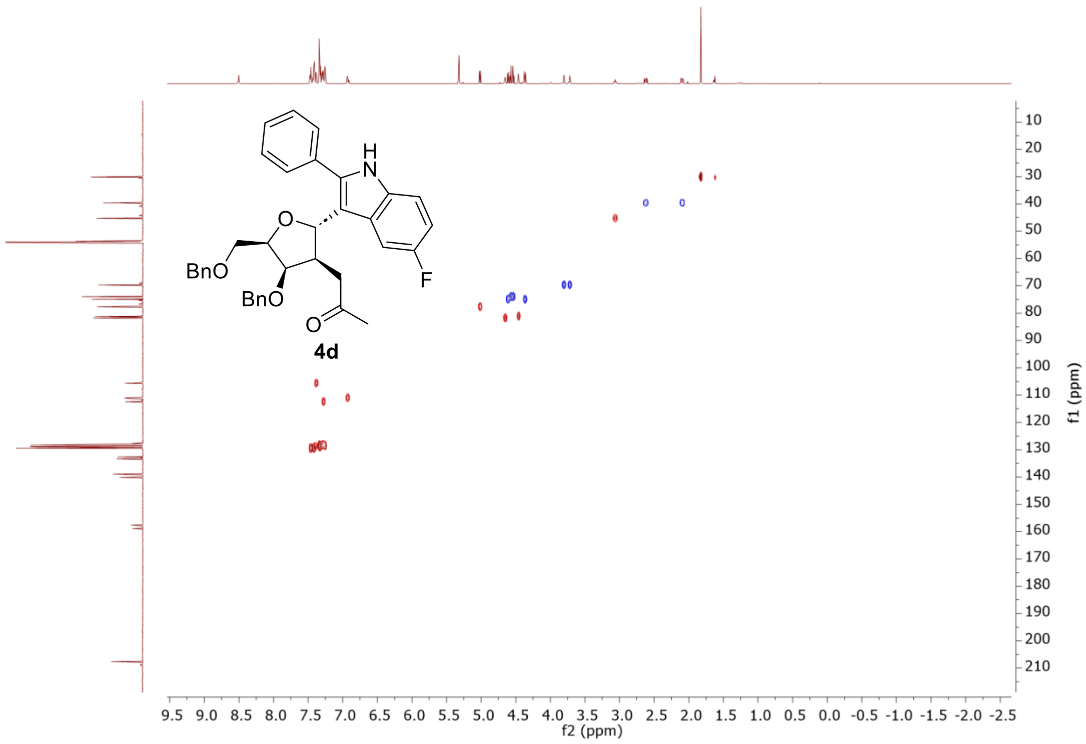

Supplementary Figure S143: HSQC spectra for 4d

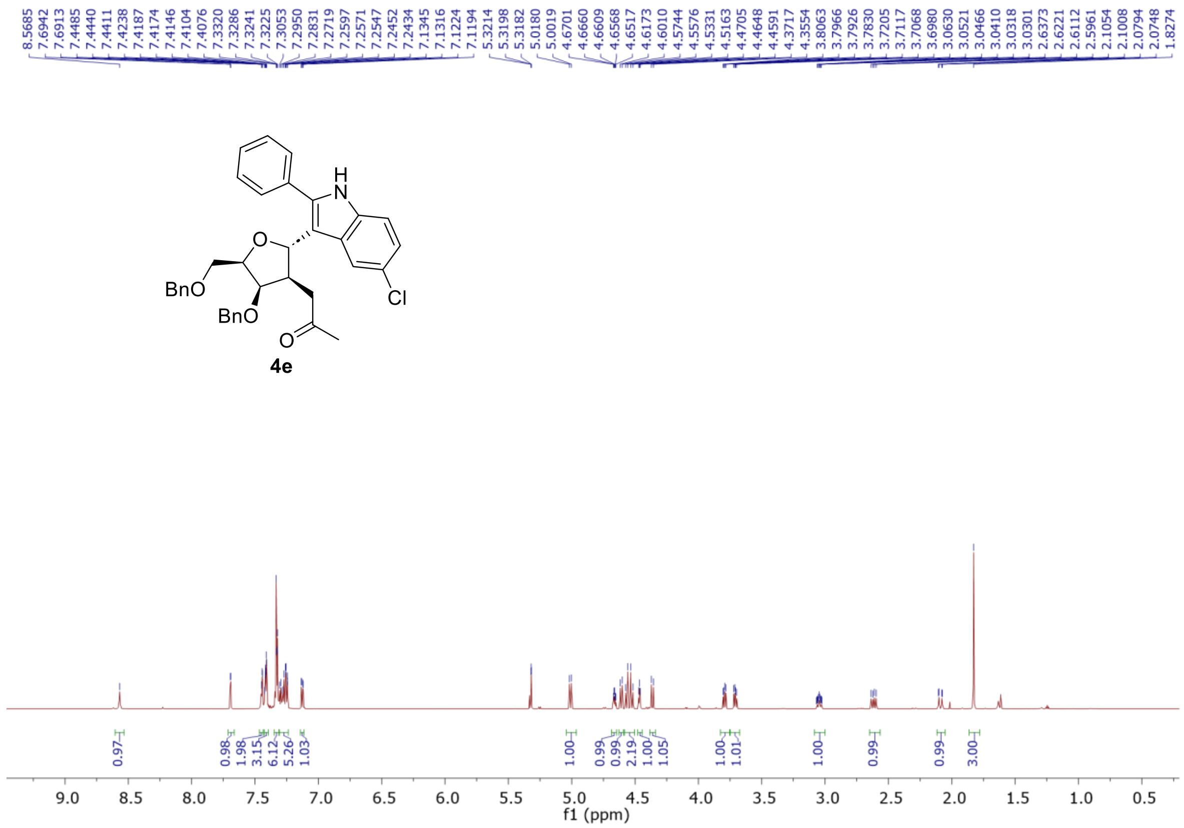

Supplementary Figure S144: ^{1}H NMR spectra for 4e

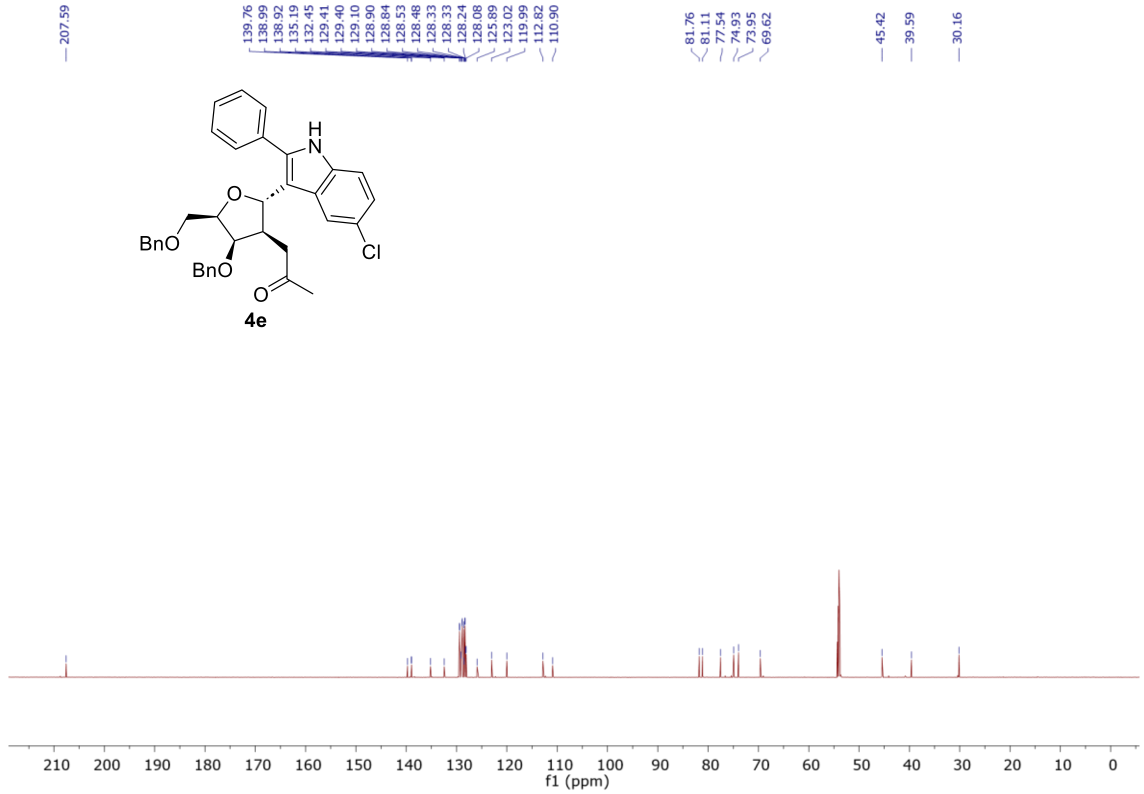

Supplementary Figure S145: ^{13}C NMR spectra for 4e

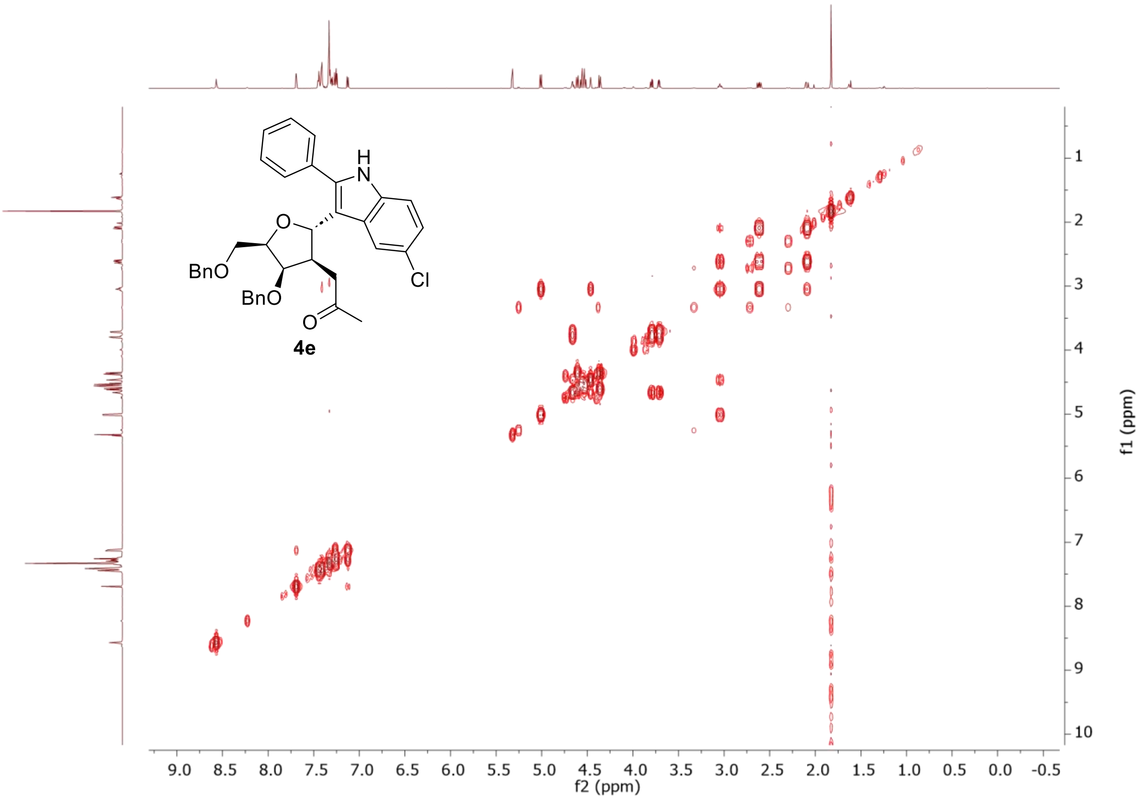

Supplementary Figure S146: COSY spectra for 4e

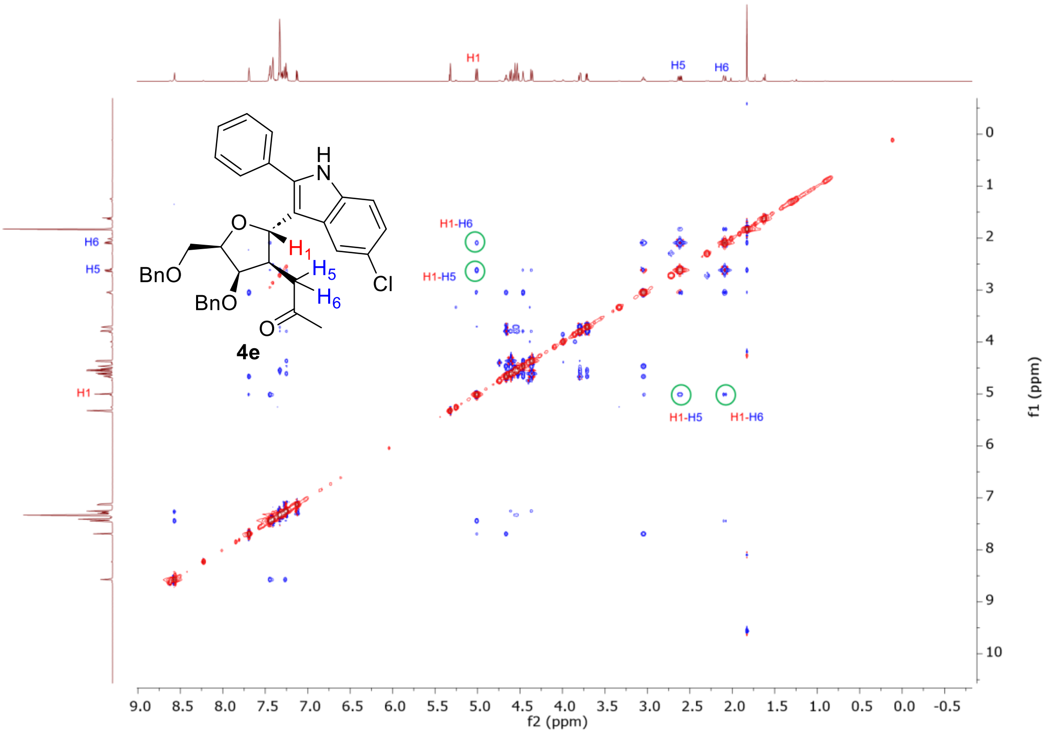

Supplementary Figure S147: NOESY spectra for 4e

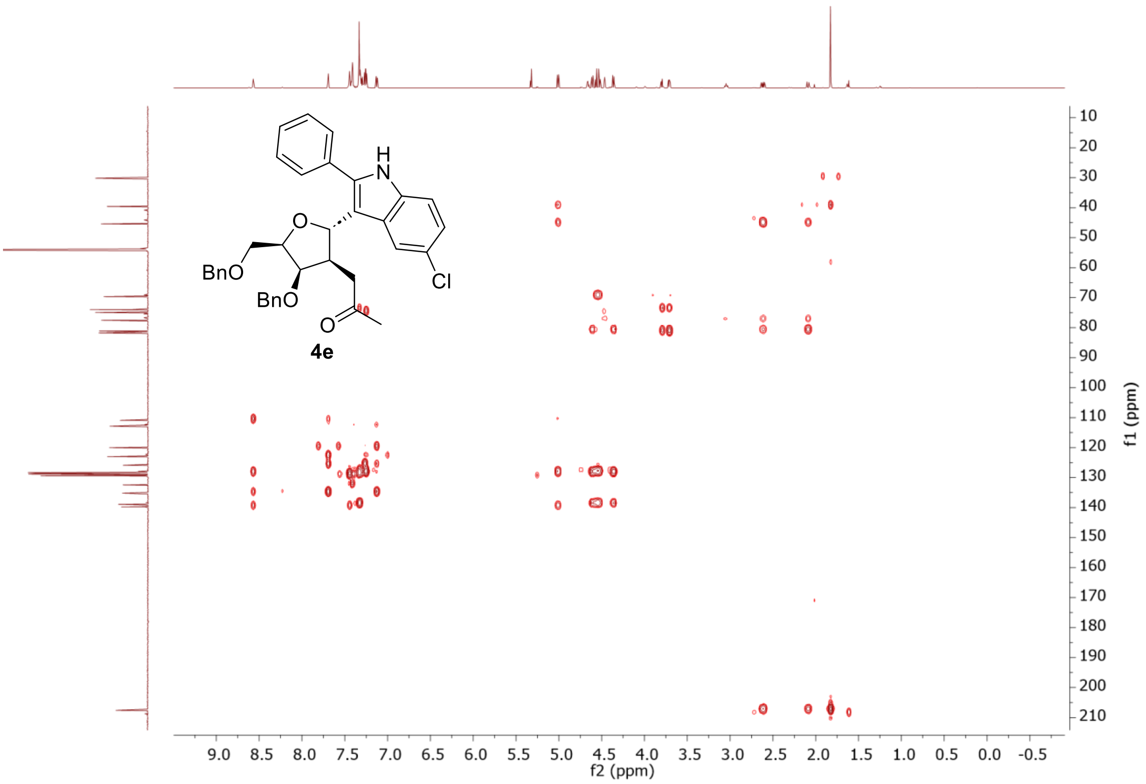

Supplementary Figure S148: HMBC spectra for 4e

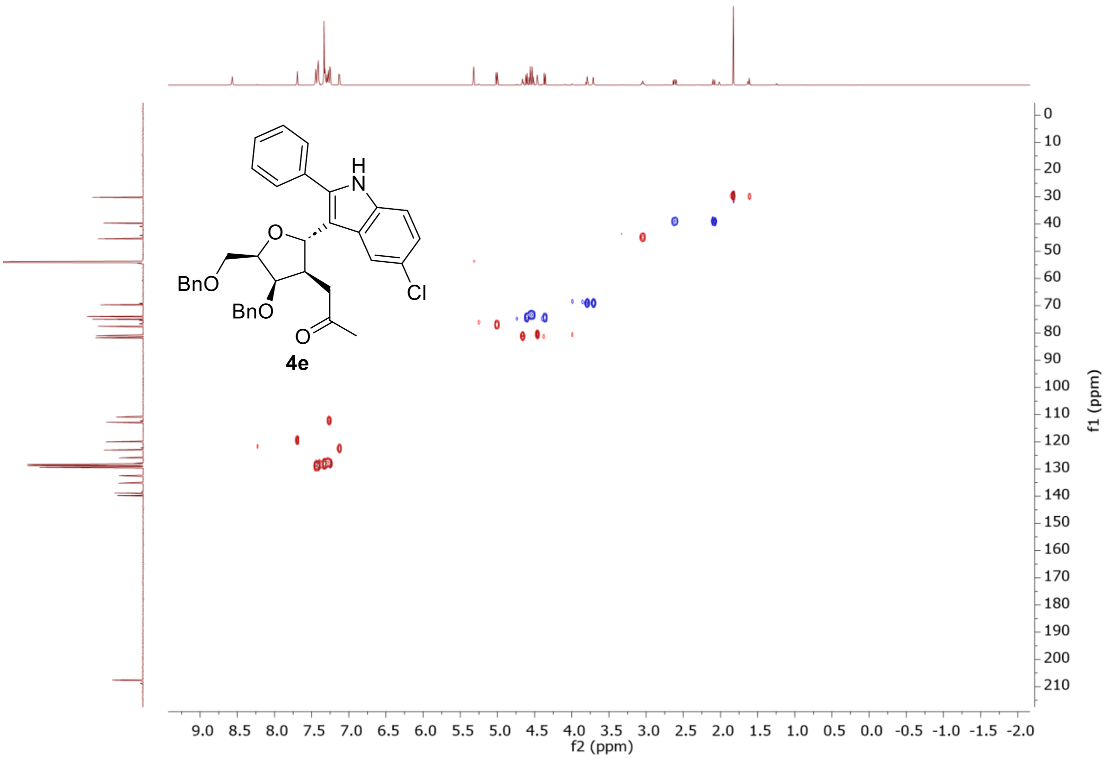

Supplementary Figure S149: HSQC spectra for 4e

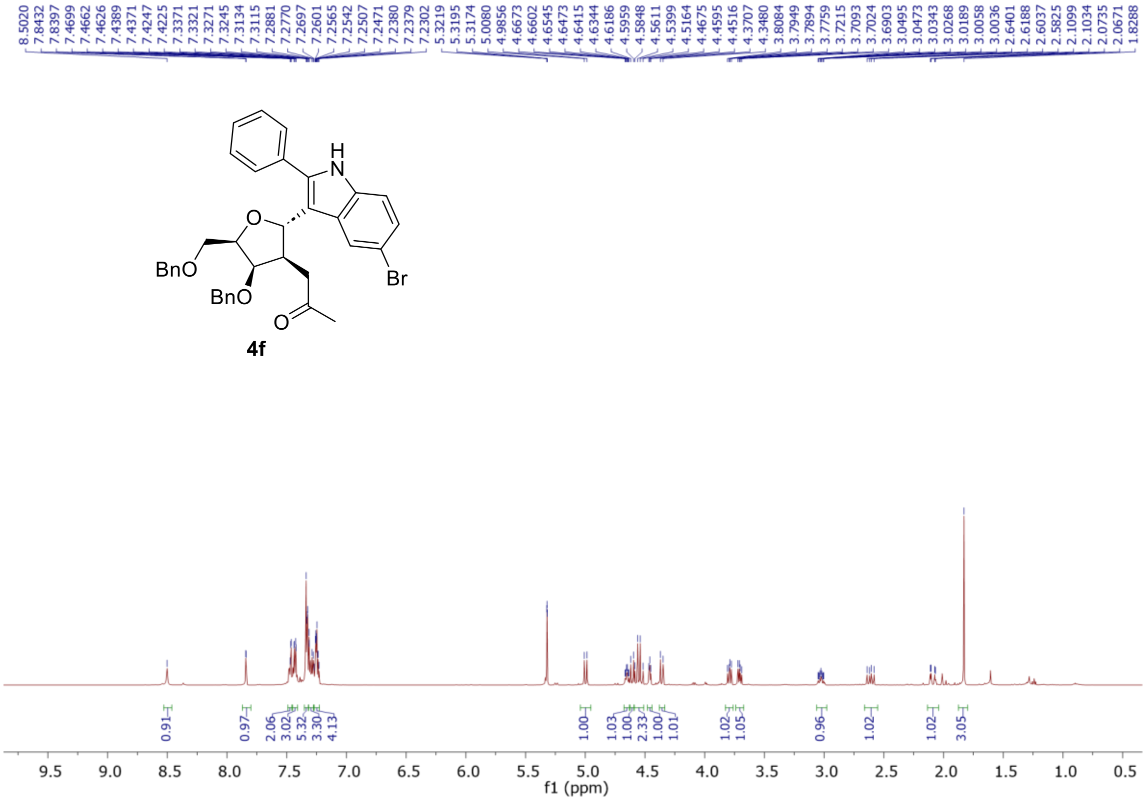

Supplementary Figure S150: ^{1}H NMR spectra for 4f

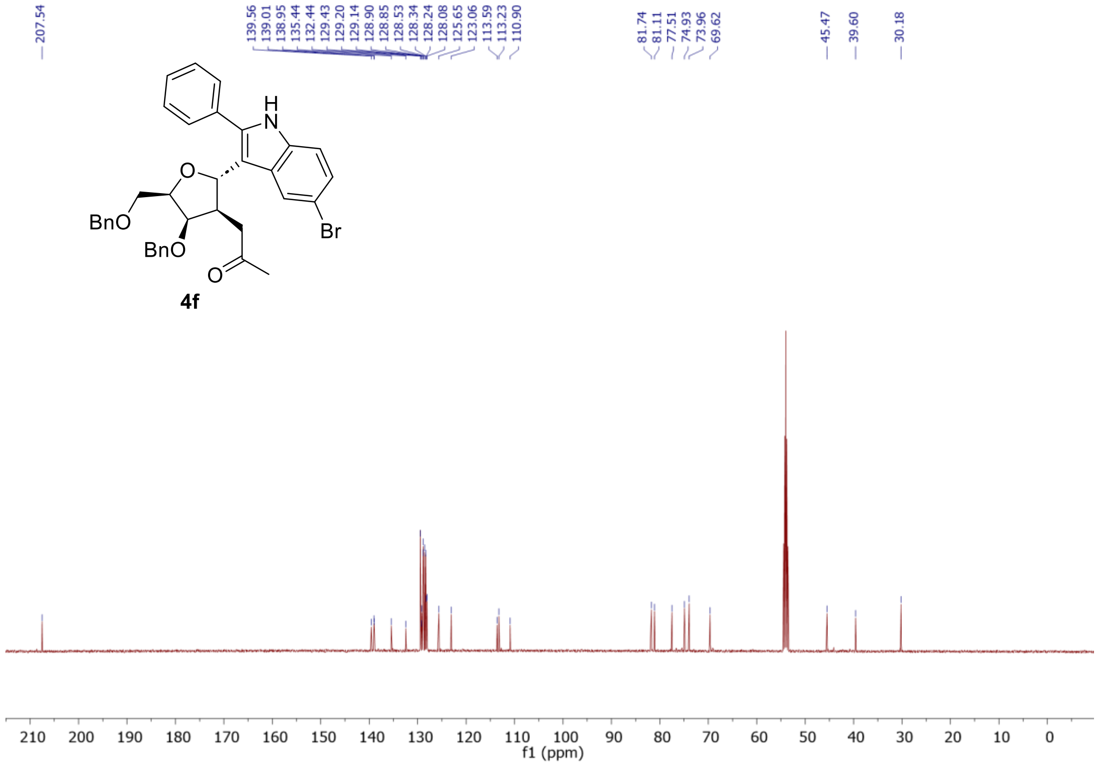

Supplementary Figure S151: ^{13}C NMR spectra for 4f

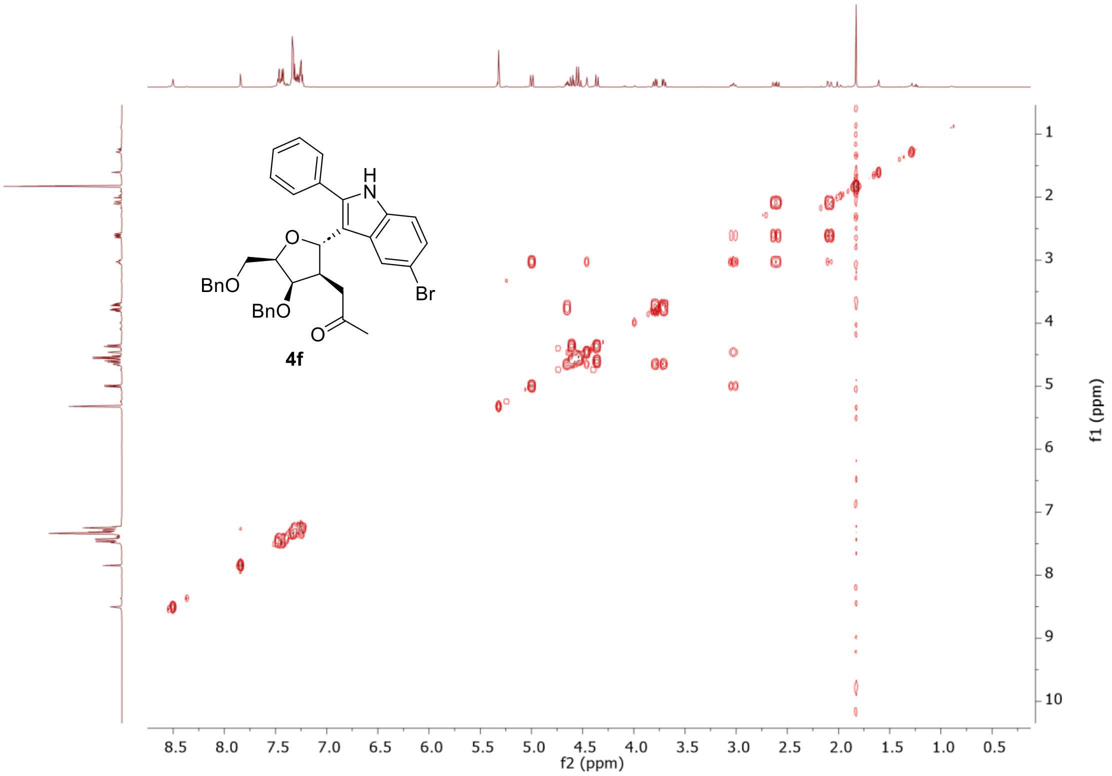

Supplementary Figure S152: COSY spectra for 4f

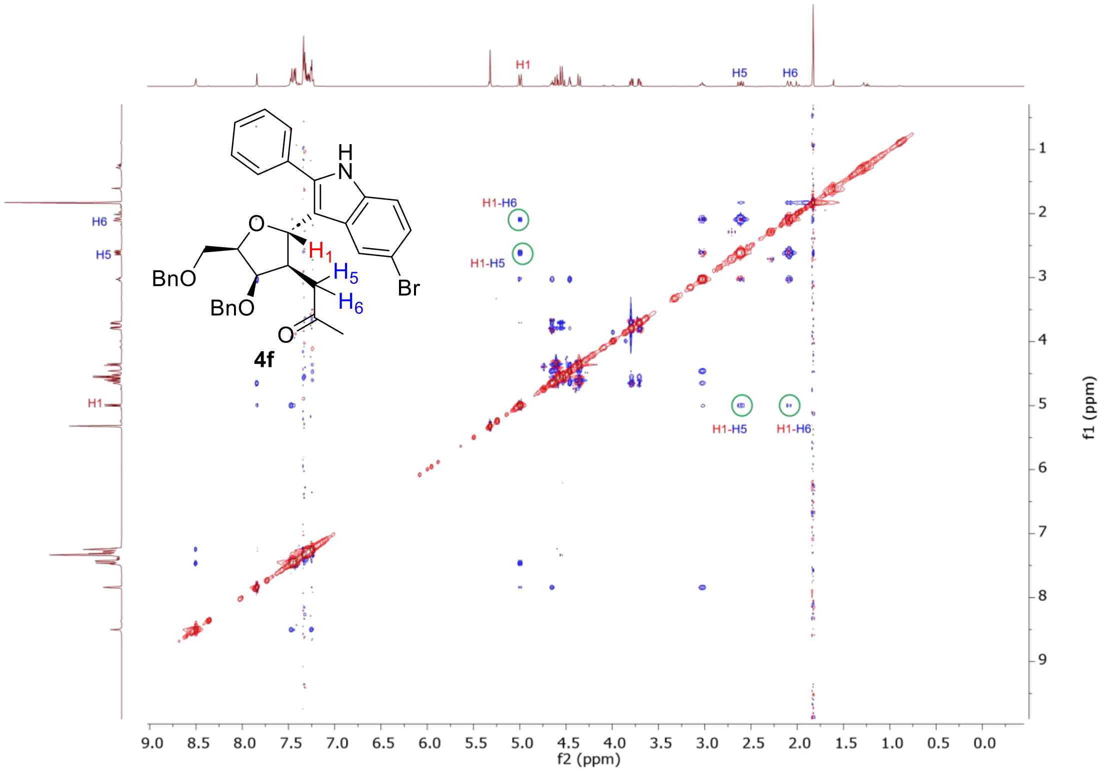

Supplementary Figure S153: NOESY spectra for 4f

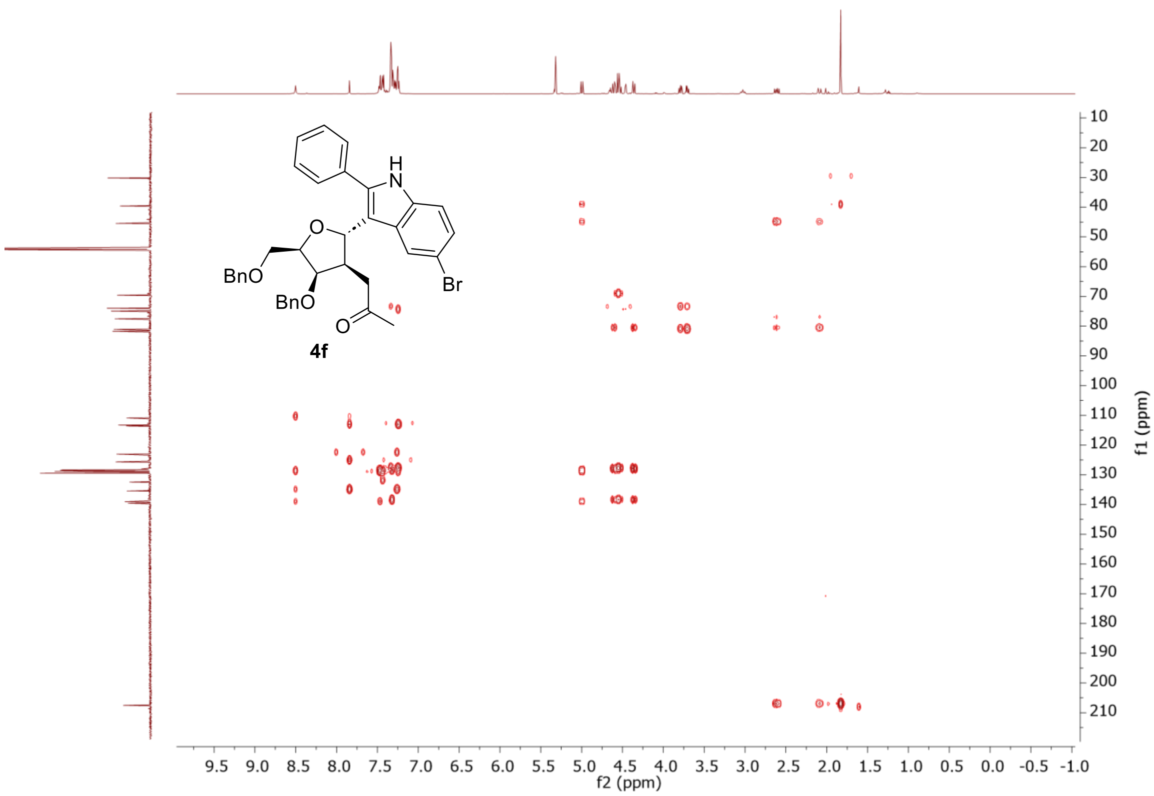

Supplementary Figure S154: HMBC spectra for 4f

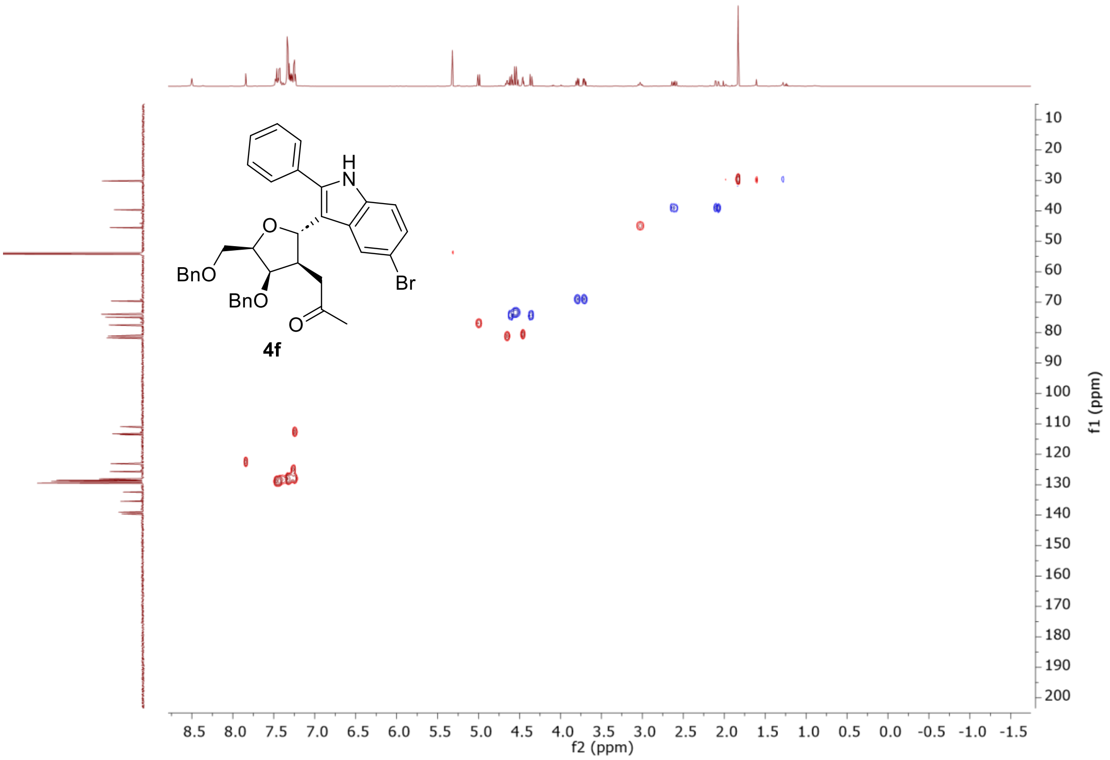

Supplementary Figure S155: HSQC spectra for 4f

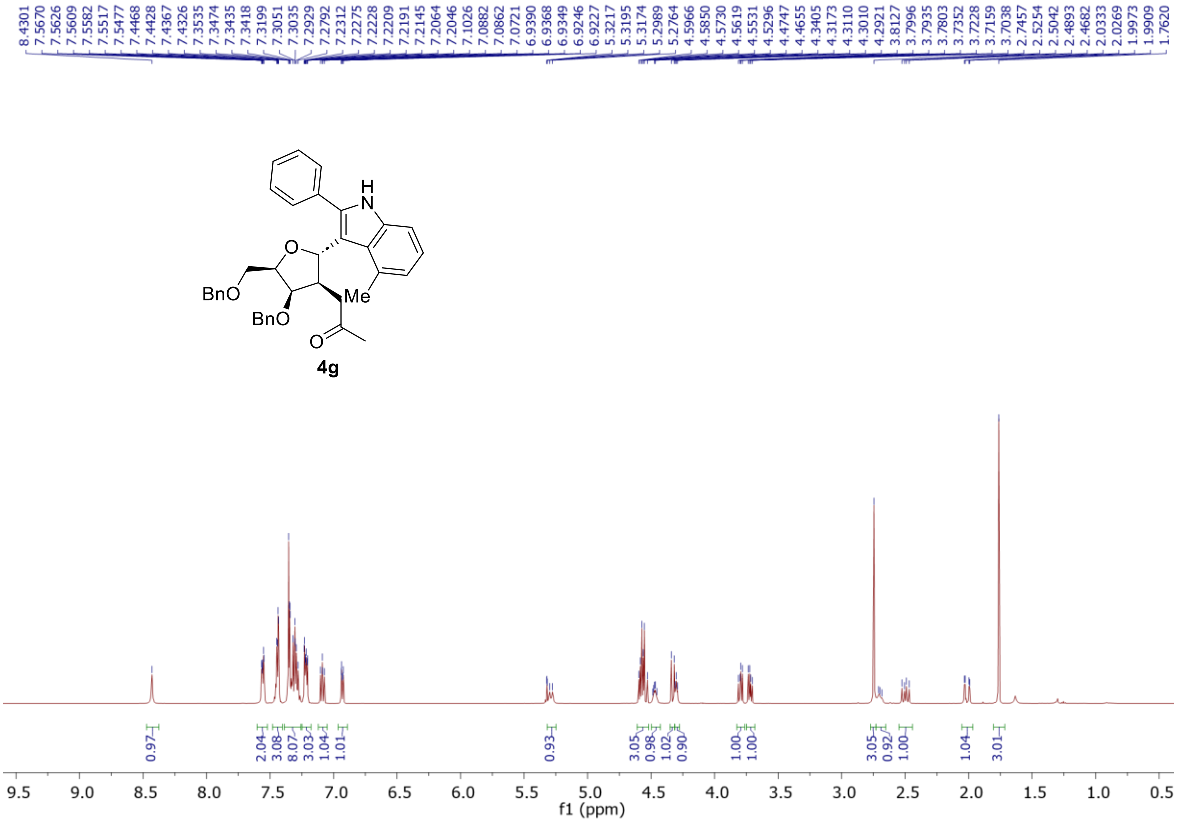

Supplementary Figure S156: ^{1}H NMR spectra for 4g

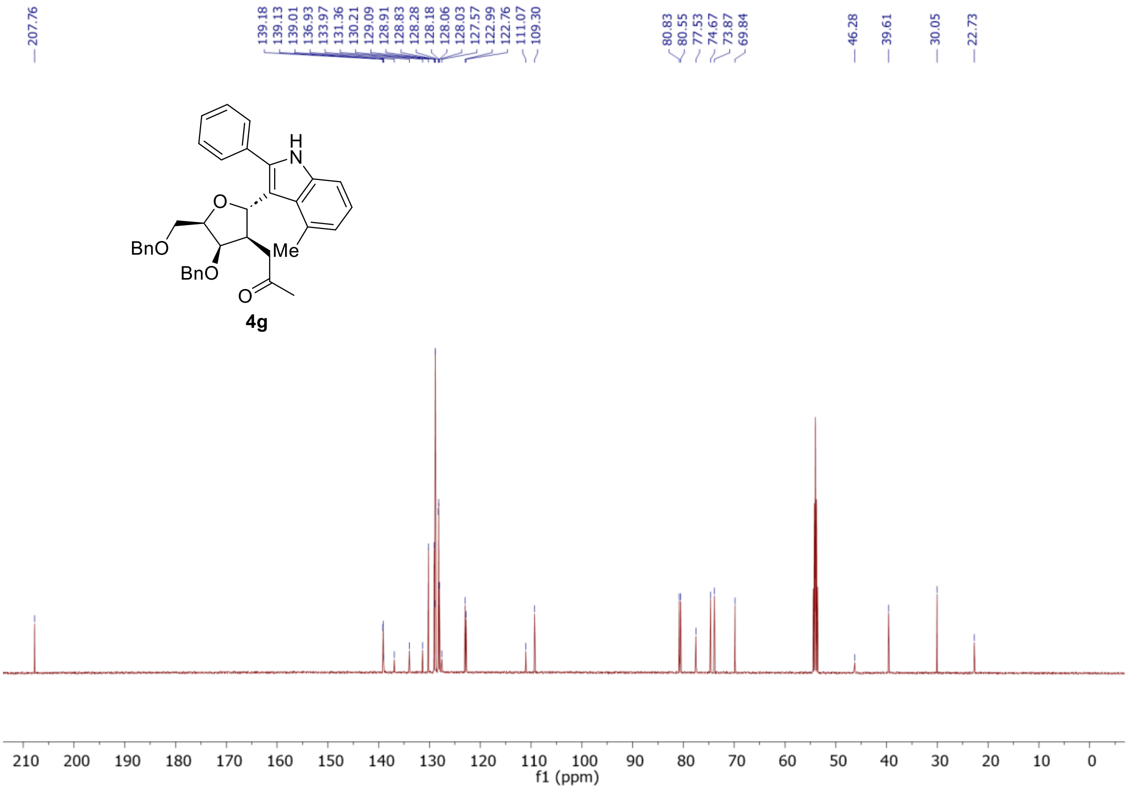

Supplementary Figure S157: ^{13}C NMR spectra for 4g

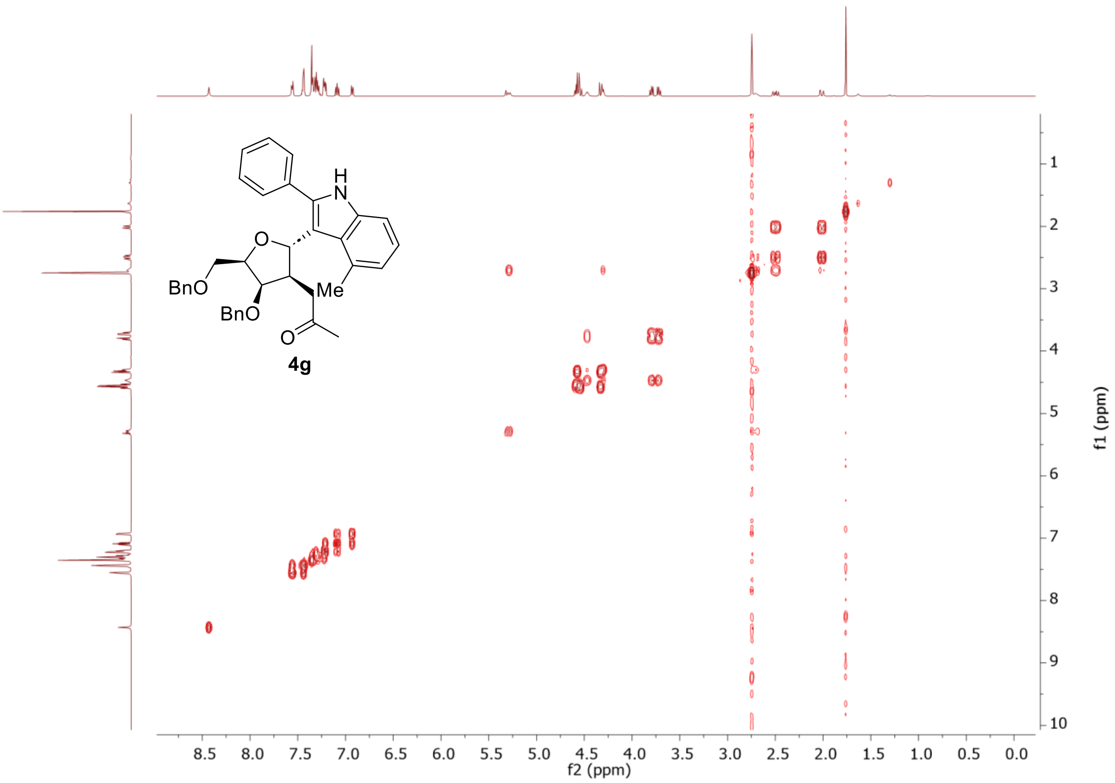

Supplementary Figure S158: CSOY spectra for 4g

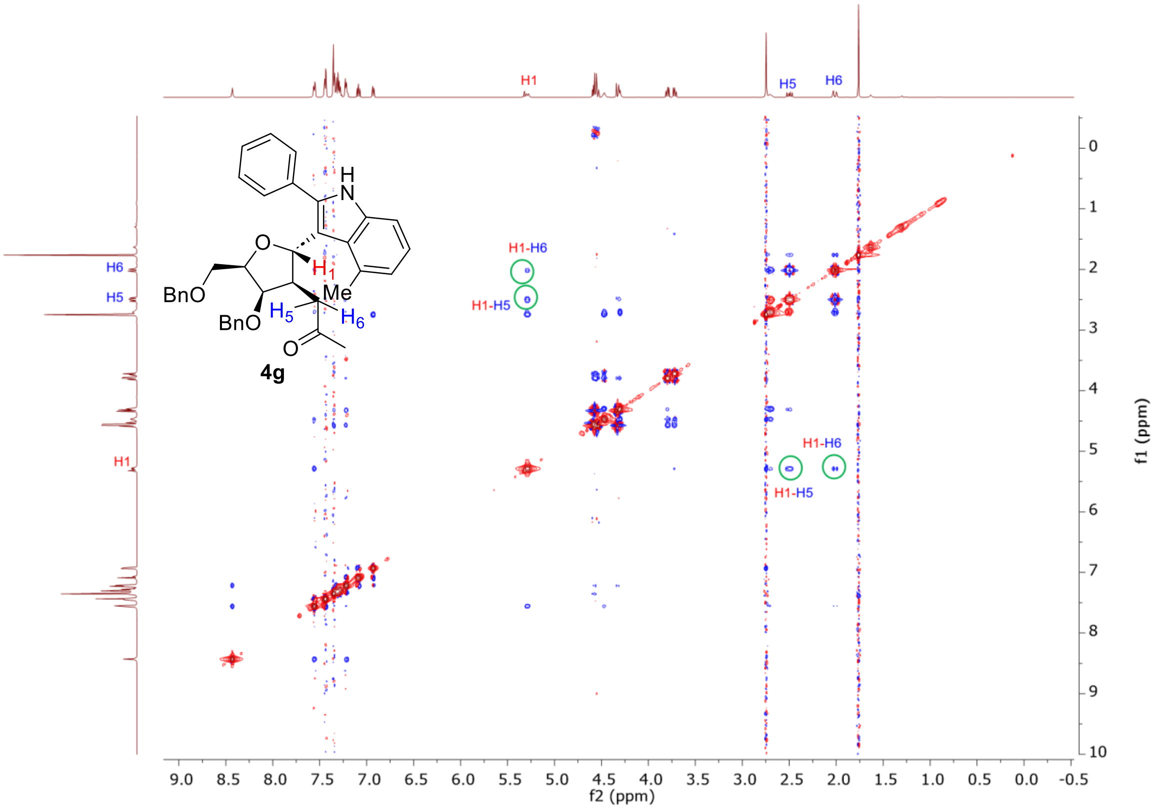

Supplementary Figure S159: NOESY spectra for 4g

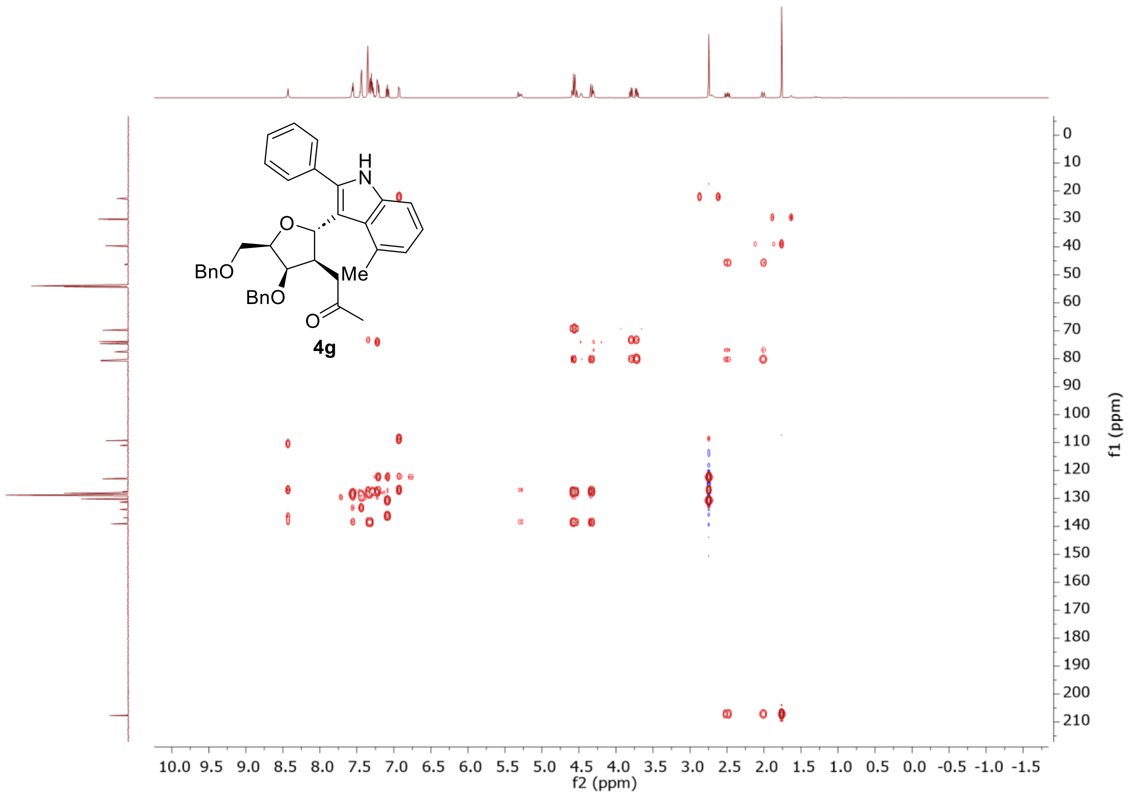

Supplementary Figure S160: HMBC spectra for 4g

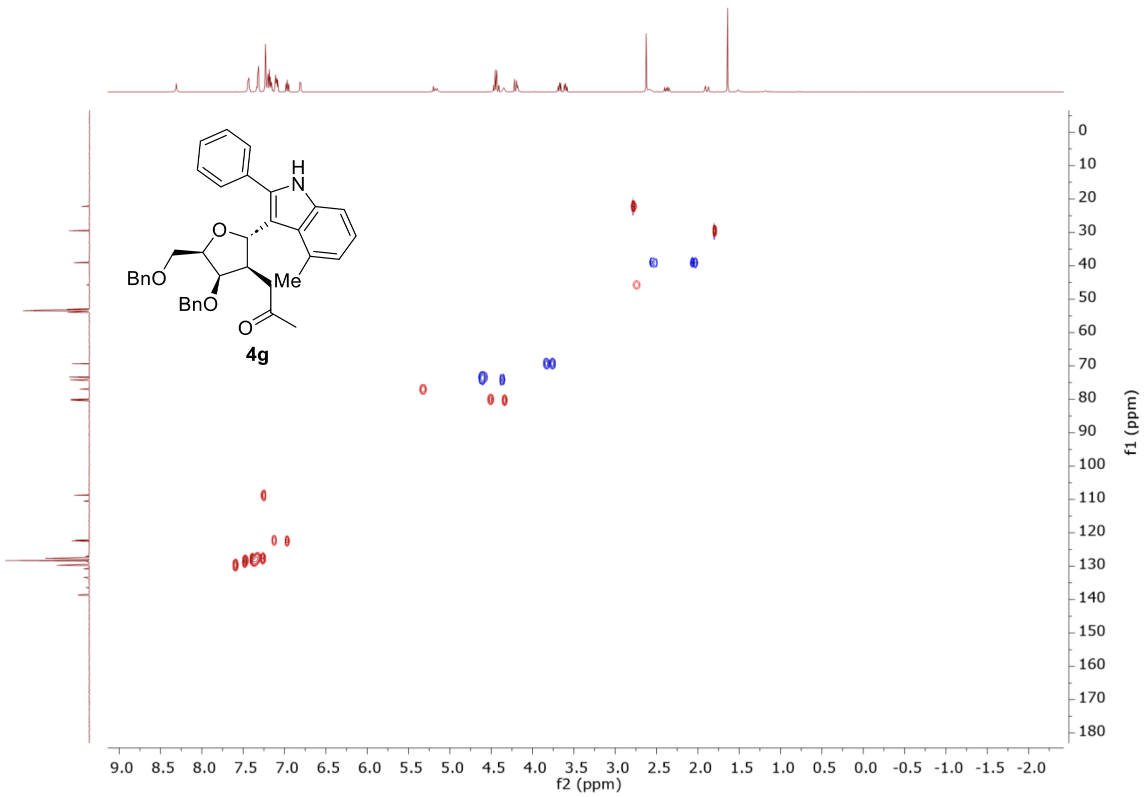

Supplementary Figure S161: HSQC spectra for 4g

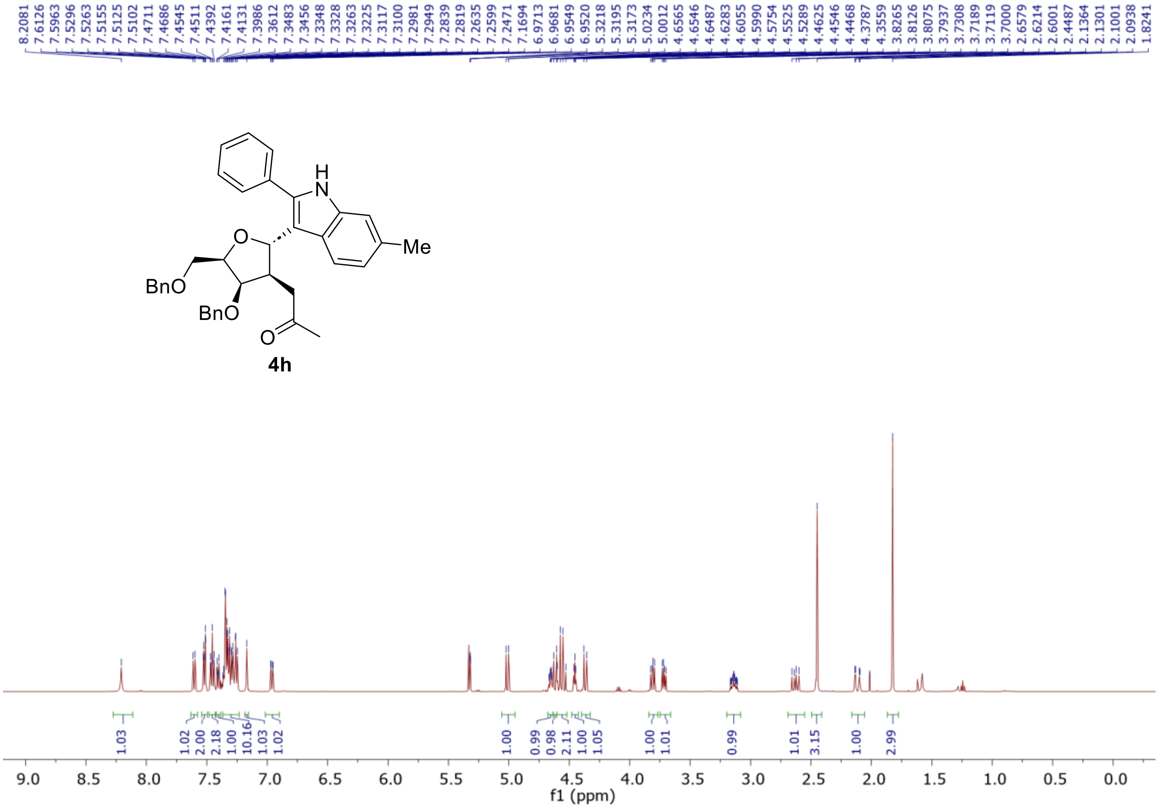

Supplementary Figure S162: ^{1}H NMR spectra for 4h

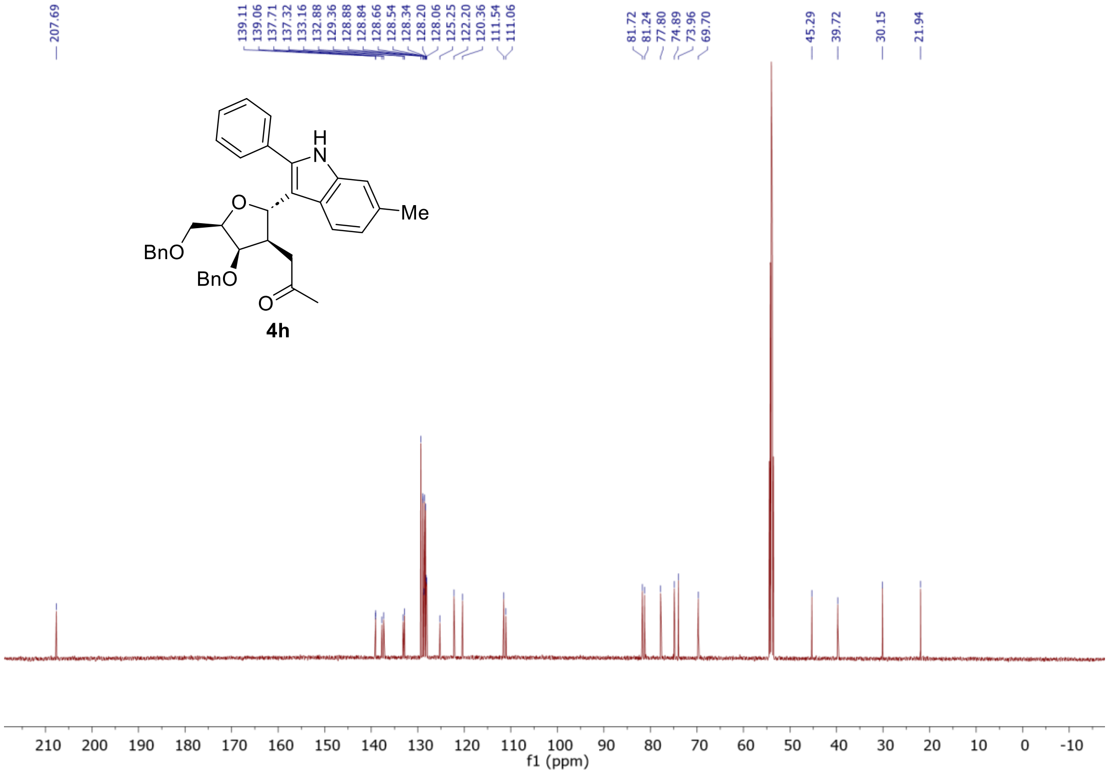

Supplementary Figure S163: ^{13}C NMR spectra for 4h

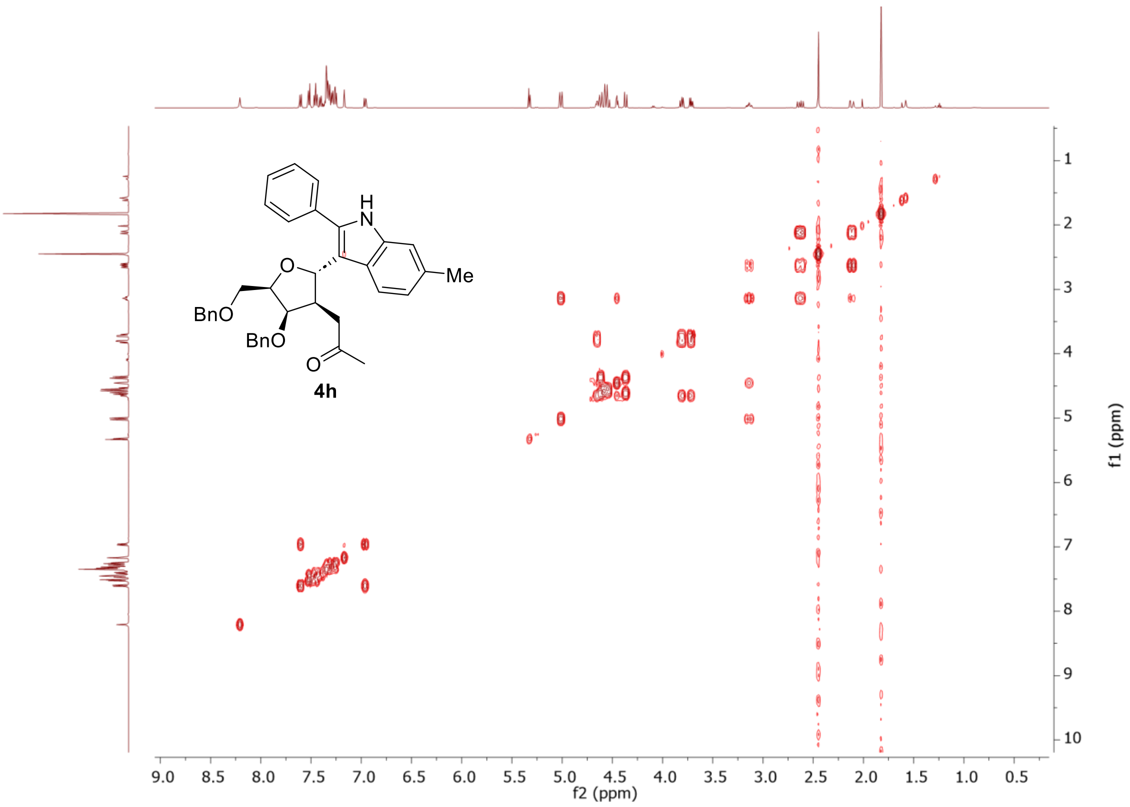

Supplementary Figure S164: COSY NMR spectra for 4h

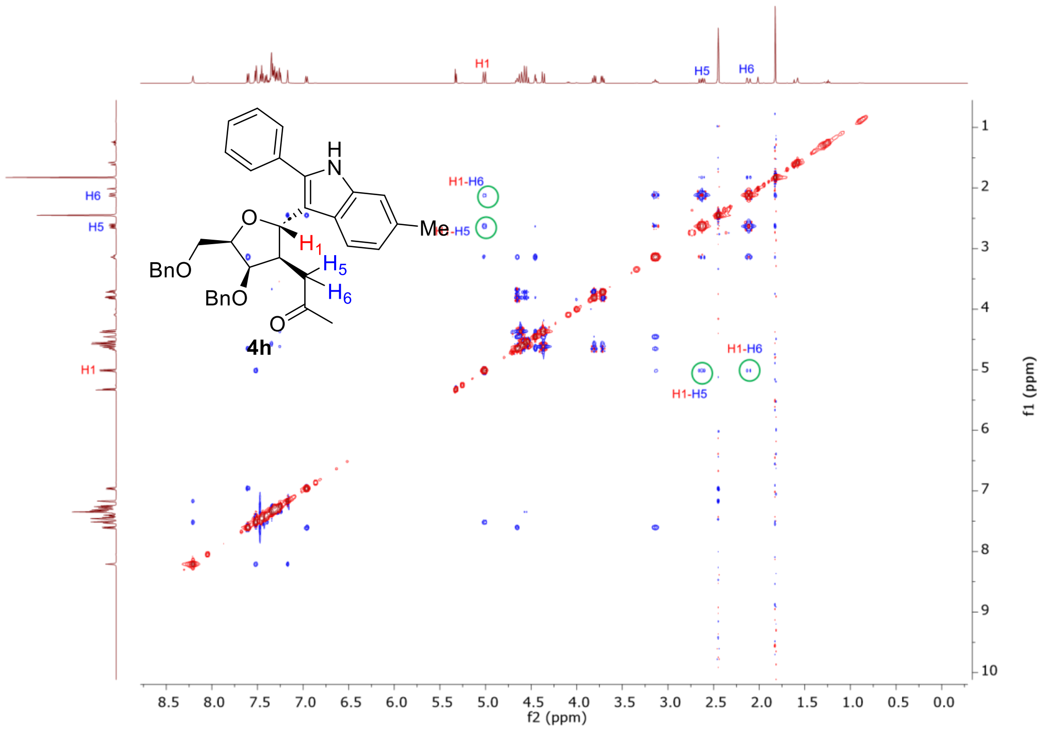

Supplementary Figure S165: NOESY NMR spectra for 4h

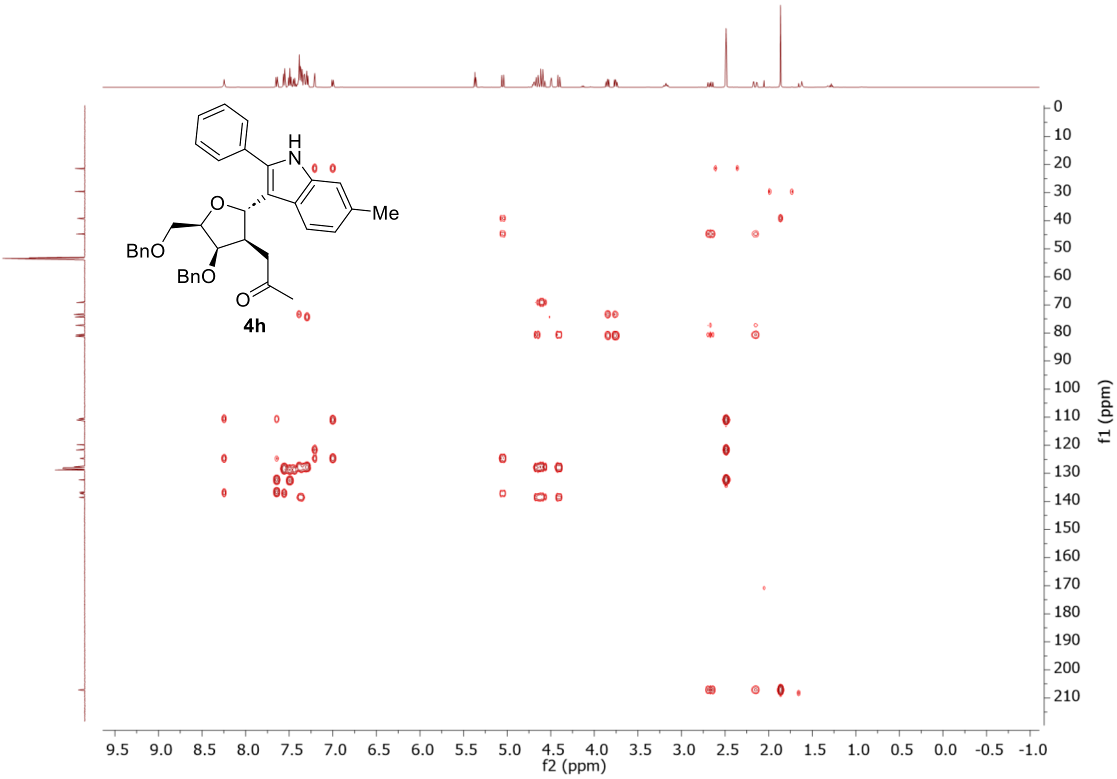

Supplementary Figure S166: HMBC spectra for 4h

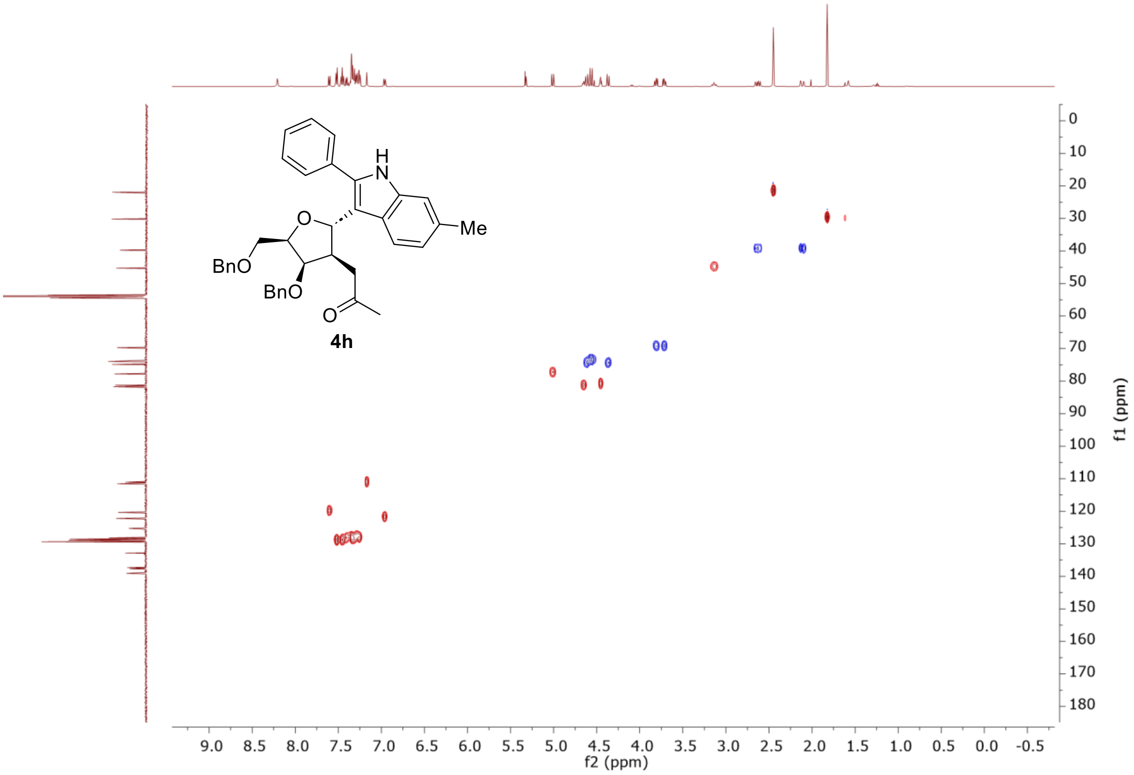

Supplementary Figure S167: HSQC spectra for 4h

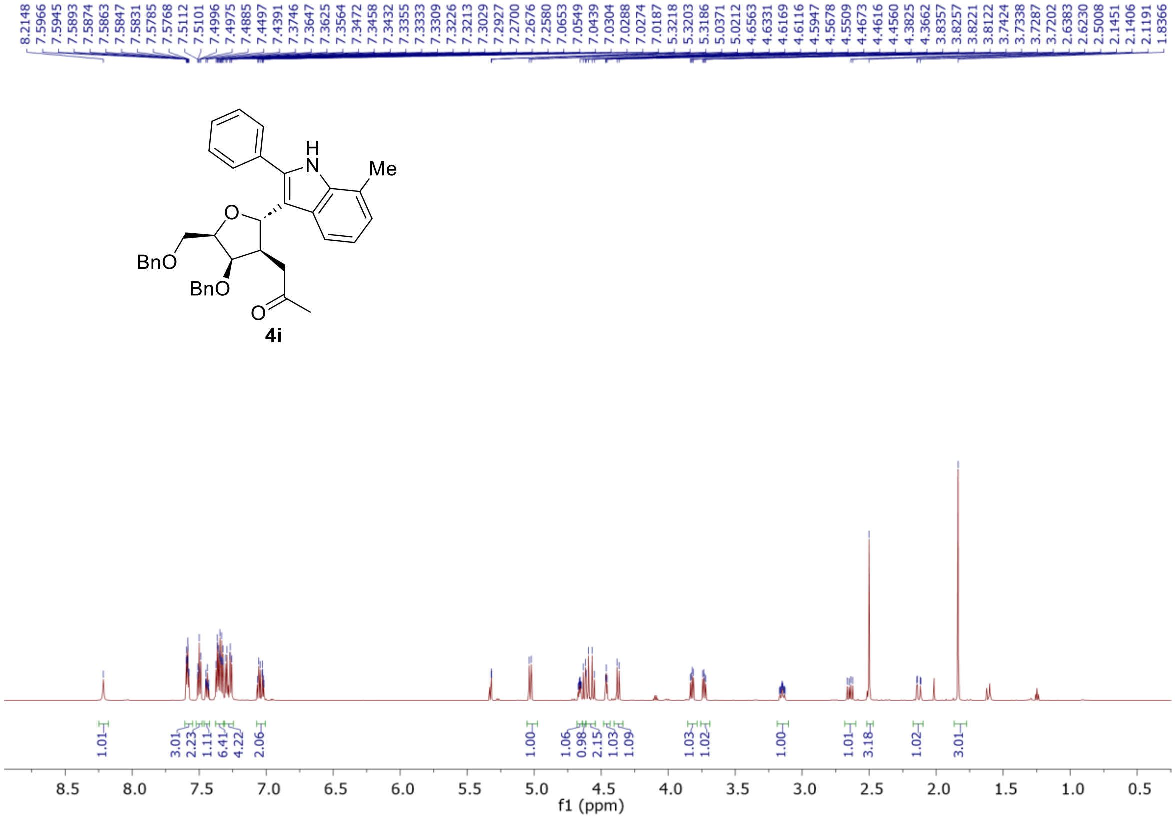

Supplementary Figure S168: ^{1}H NMR spectra for 4i

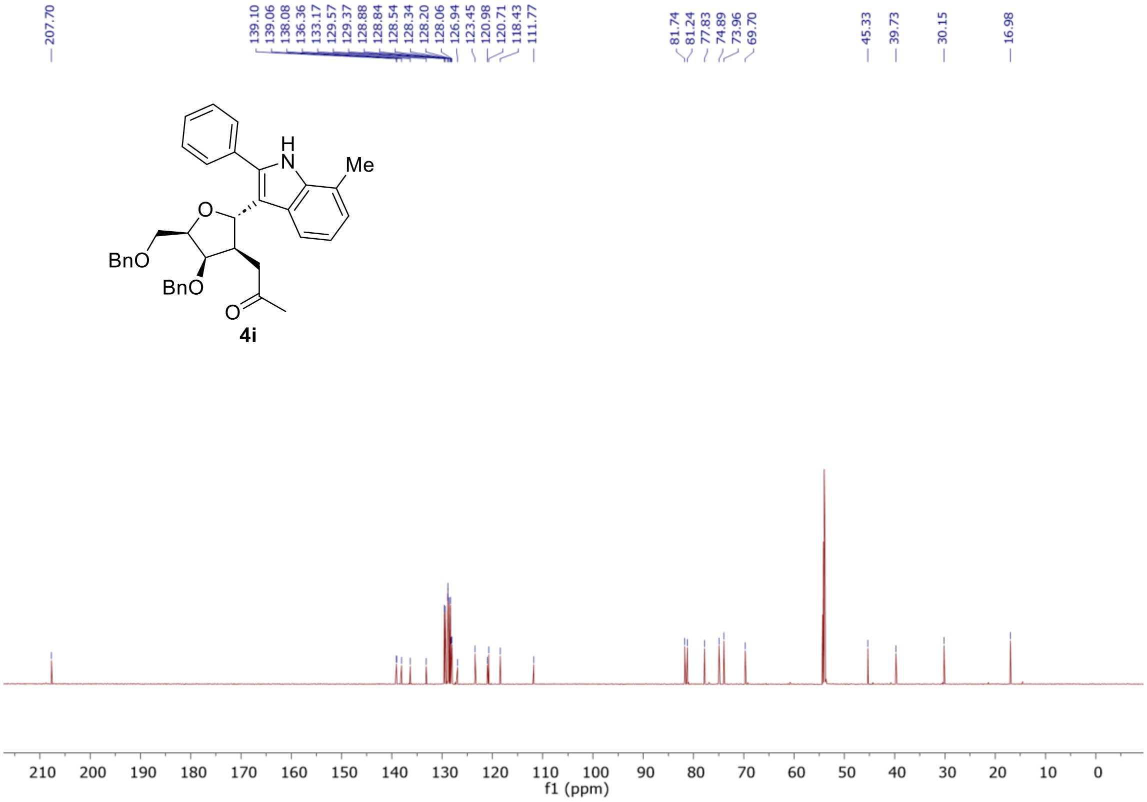

Supplementary Figure S169: ^{13}C NMR spectra for 4i

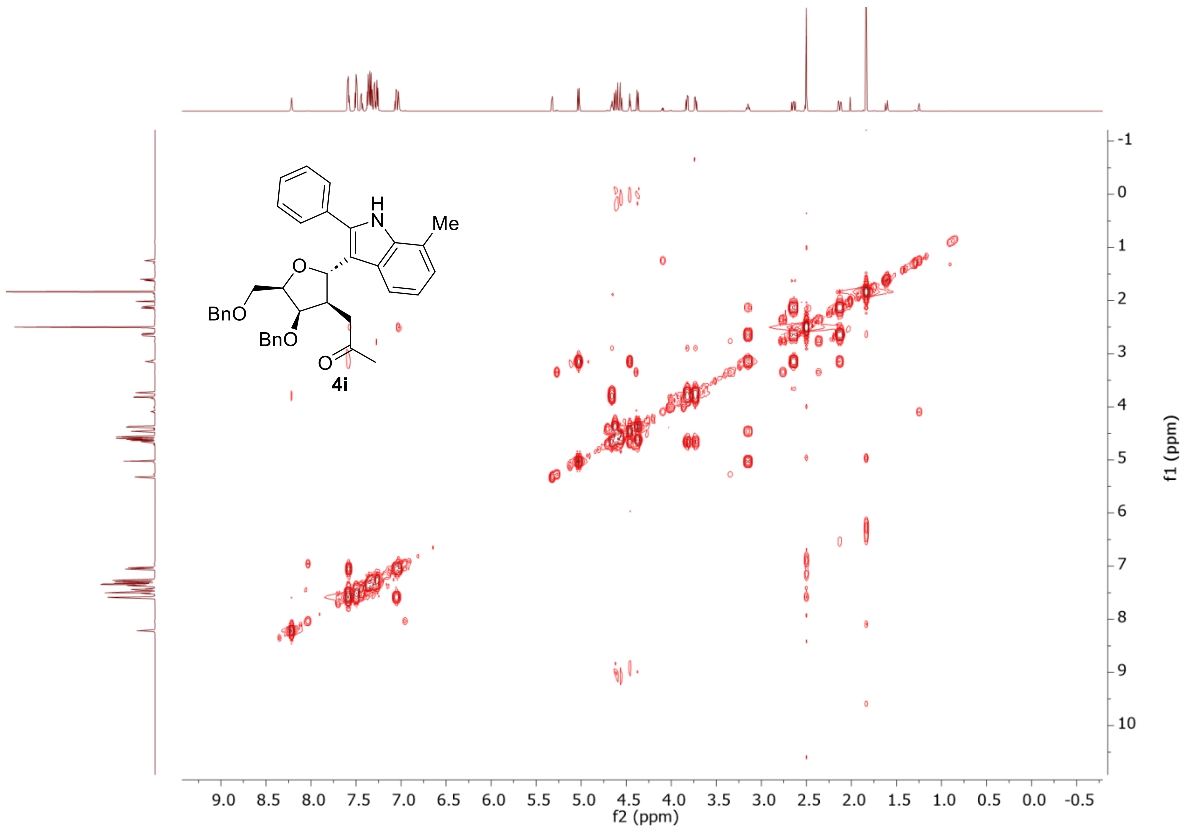

Supplementary Figure S170: COSY spectra for 4i

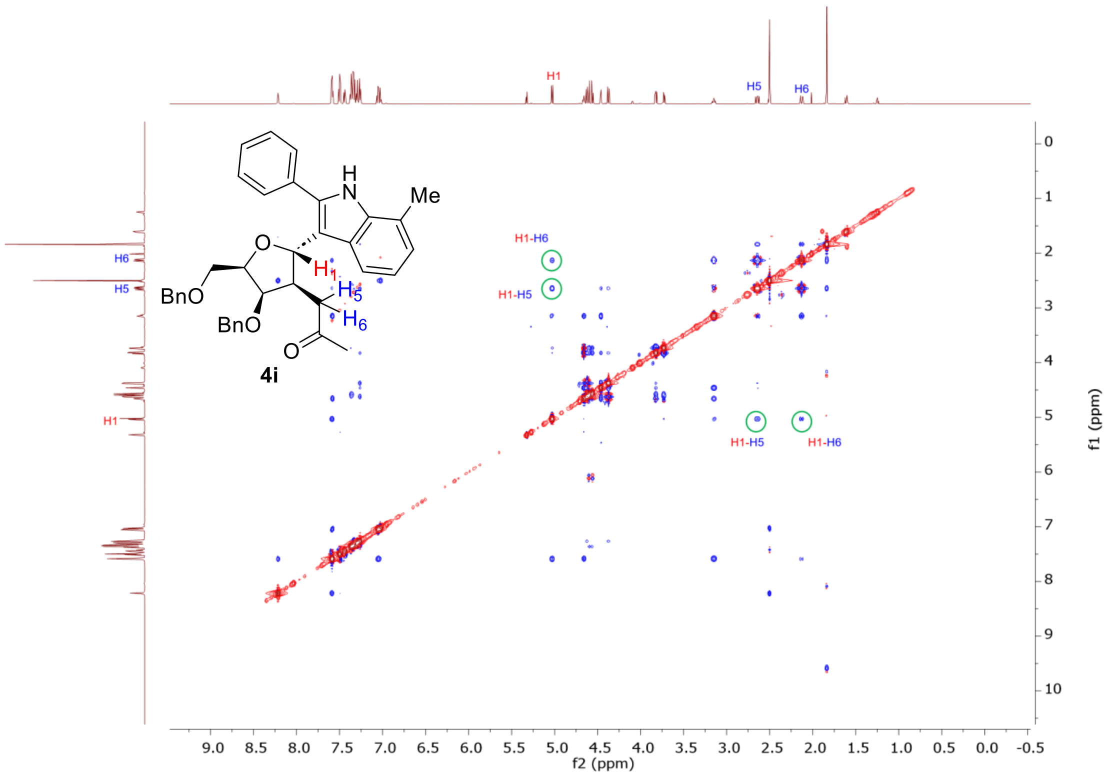

Supplementary Figure S171: NOESY spectra for 4i

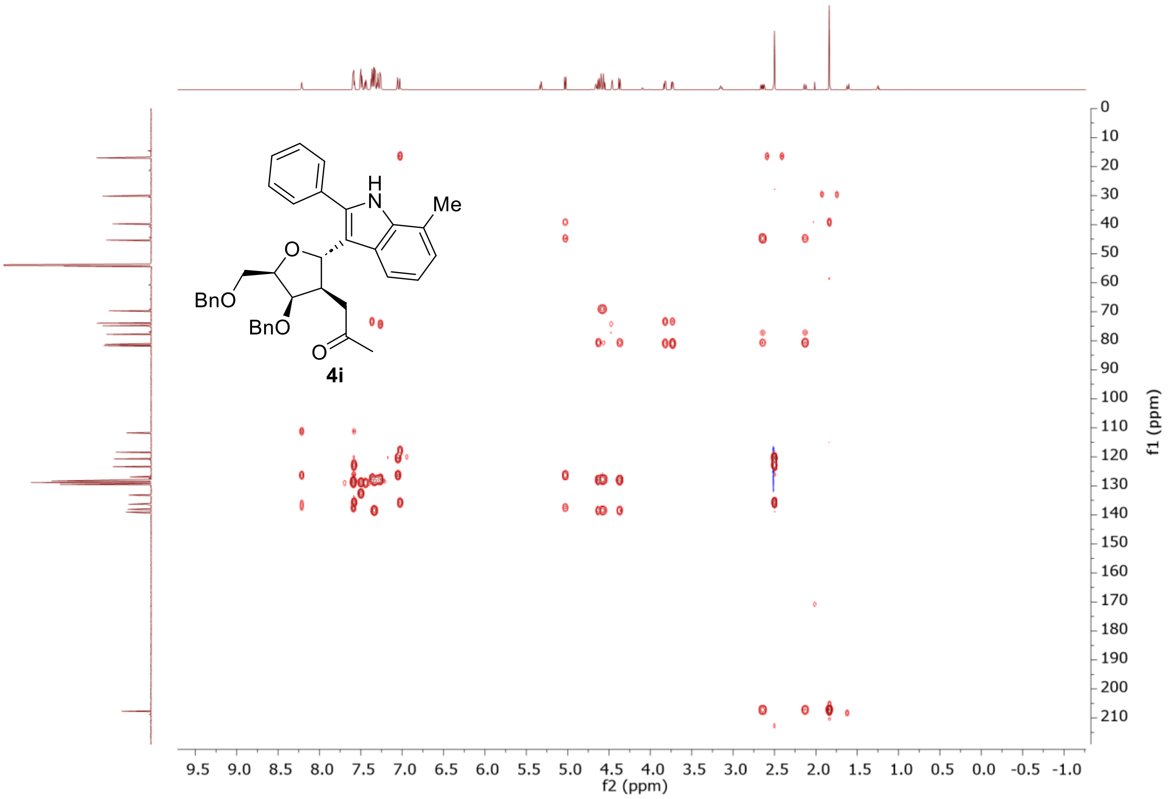

Supplementary Figure S172: HMBC spectra for 4i

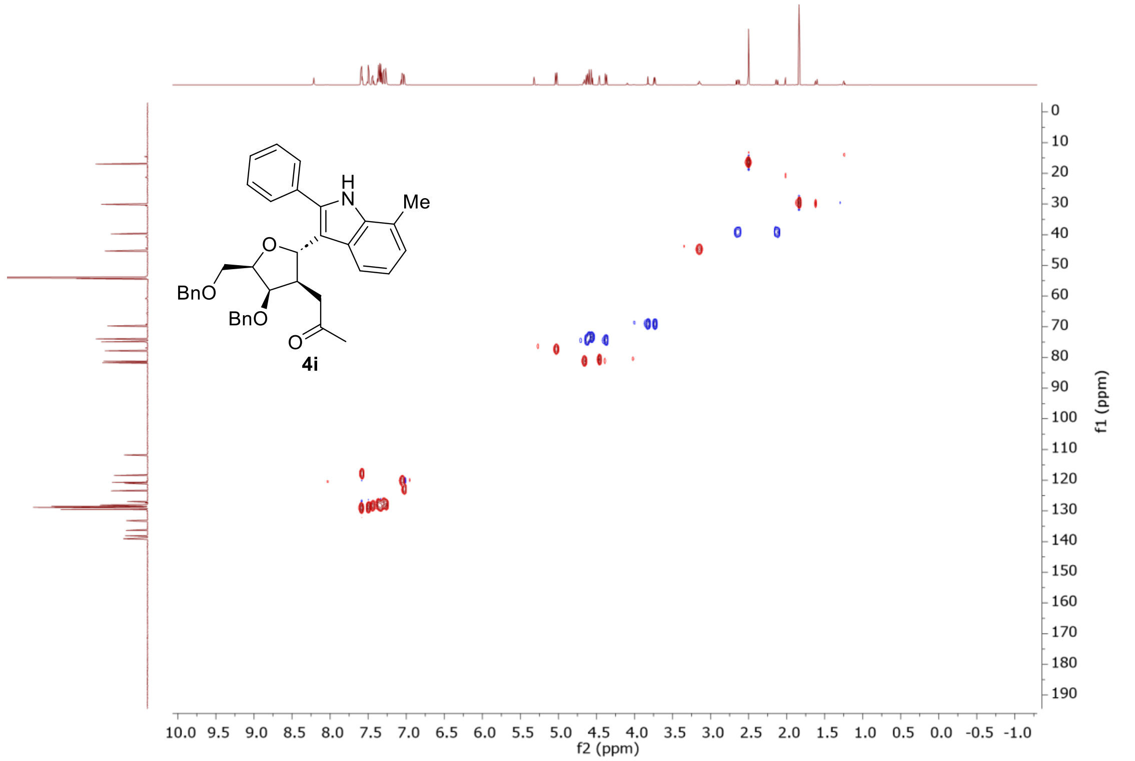

Supplementary Figure S173: HSQC spectra for 4i

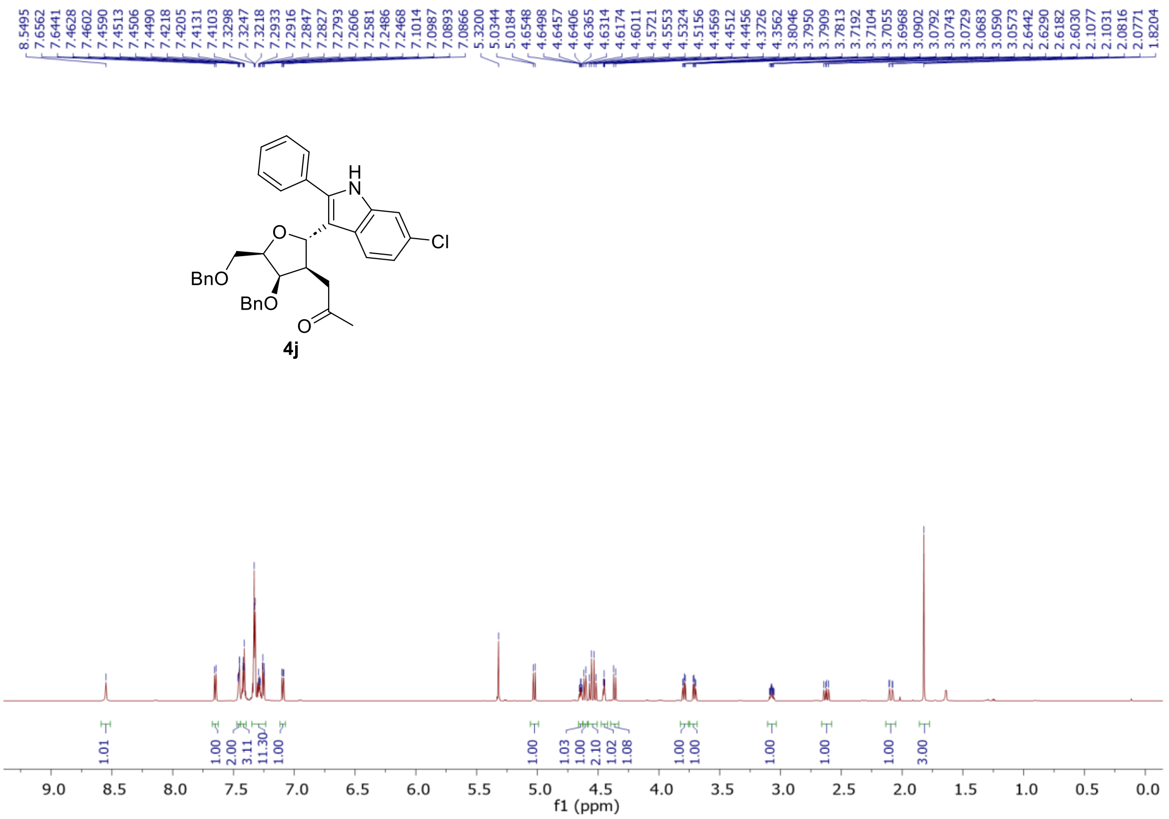

Supplementary Figure S174: ^{1}H NMR spectra for 4j

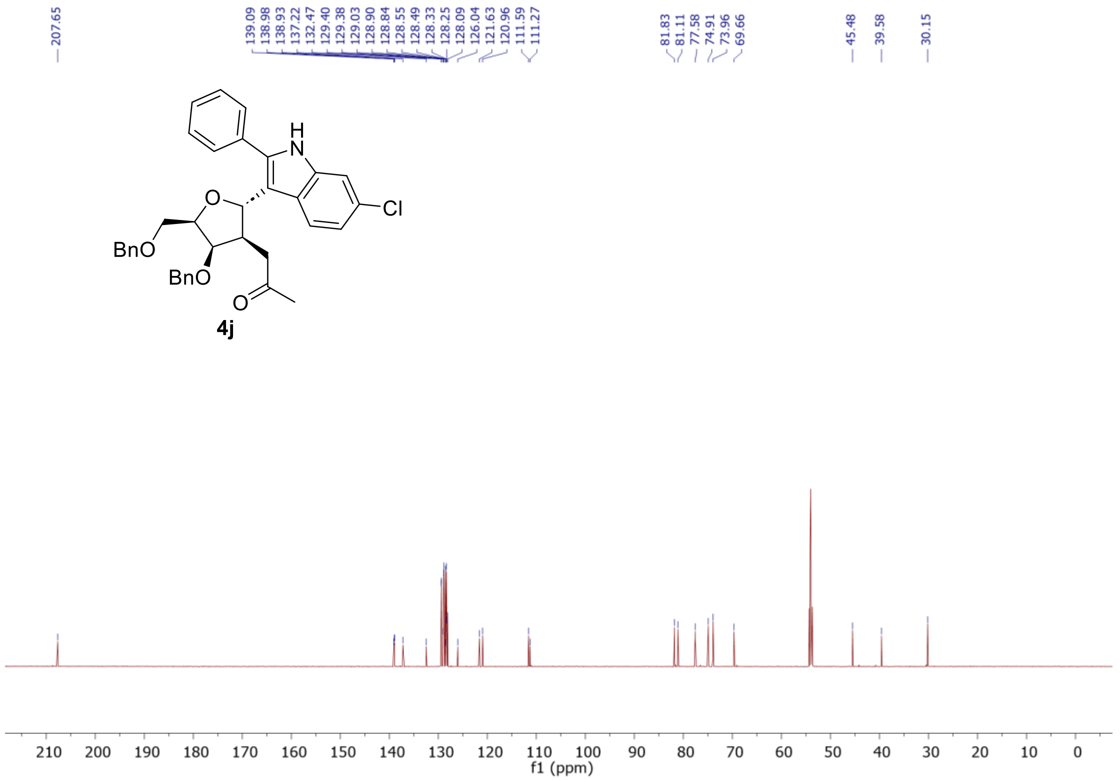

Supplementary Figure S175: ^{13}C NMR spectra for 4j

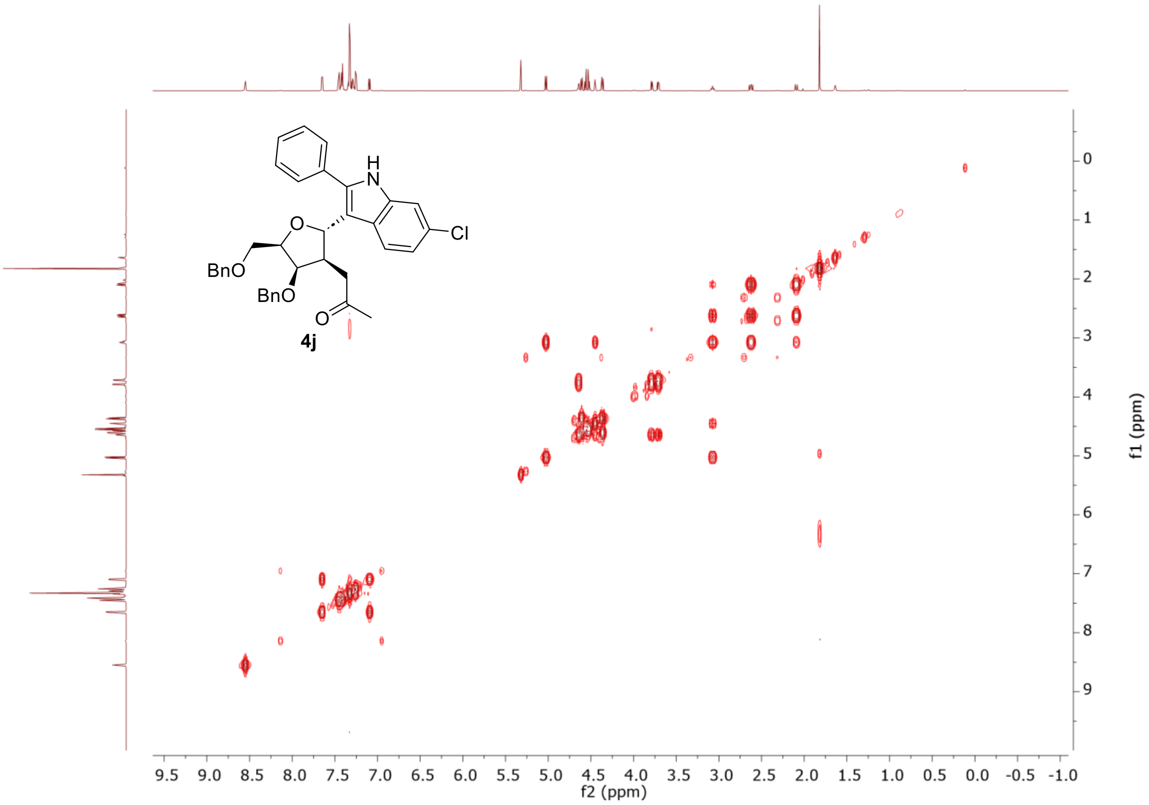

Supplementary Figure S176: COSY spectra for 4j

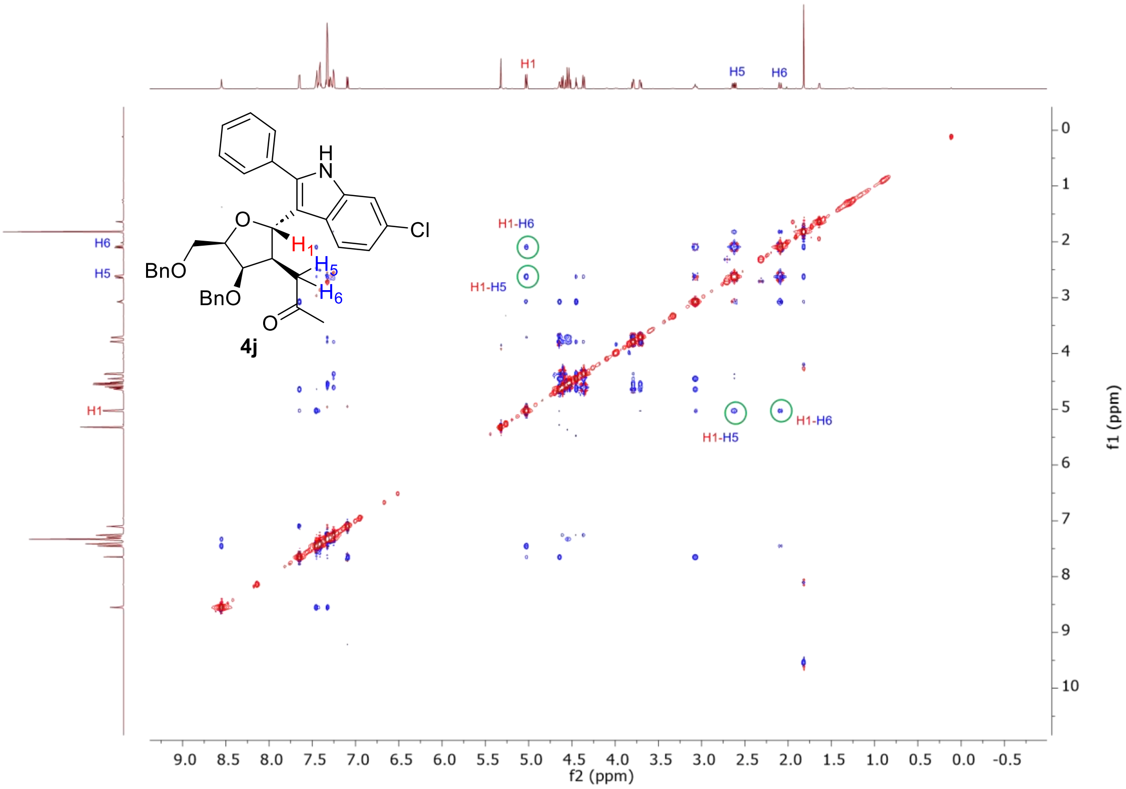

Supplementary Figure S177: NOESY spectra for 4j

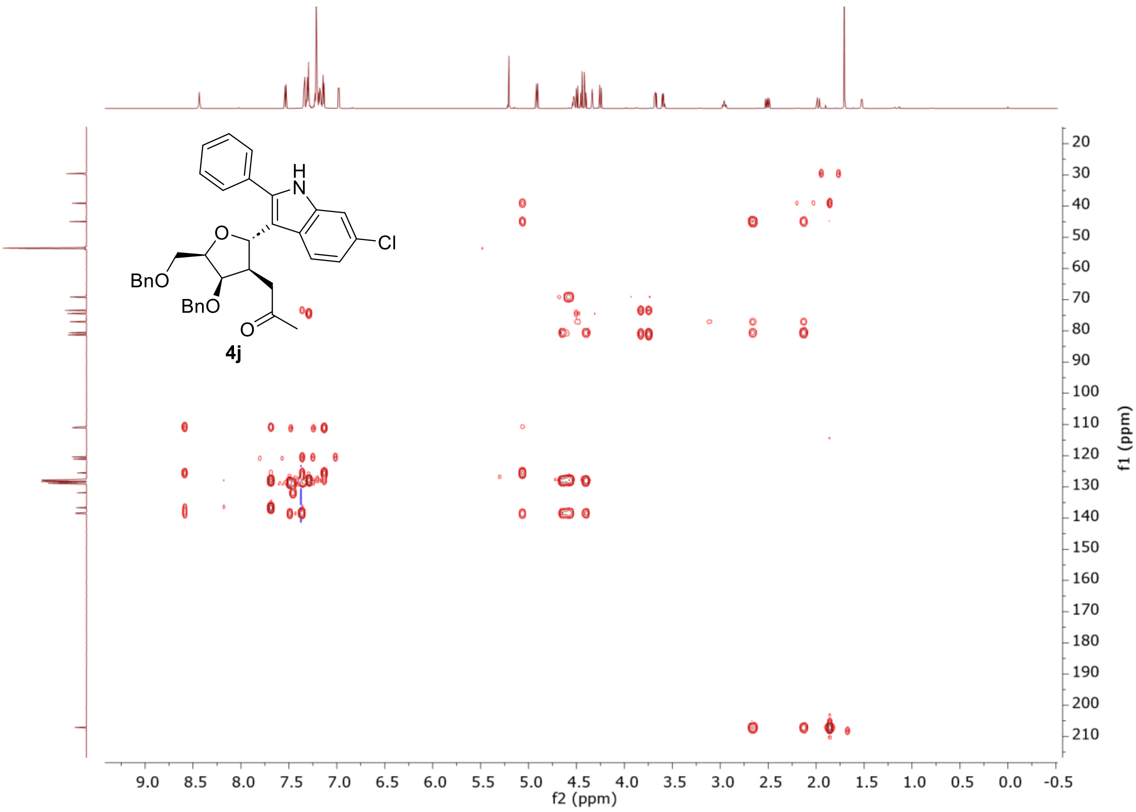

Supplementary Figure S178: HMBC spectra for 4j

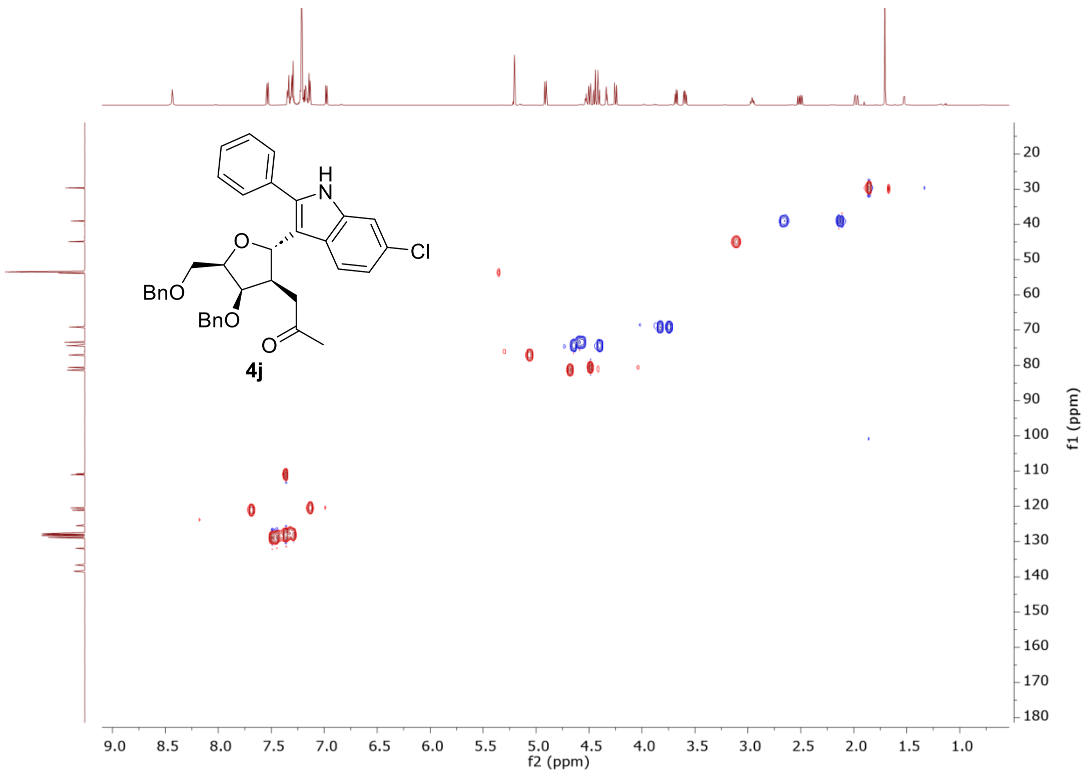

Supplementary Figure S179: HSQC spectra for 4j

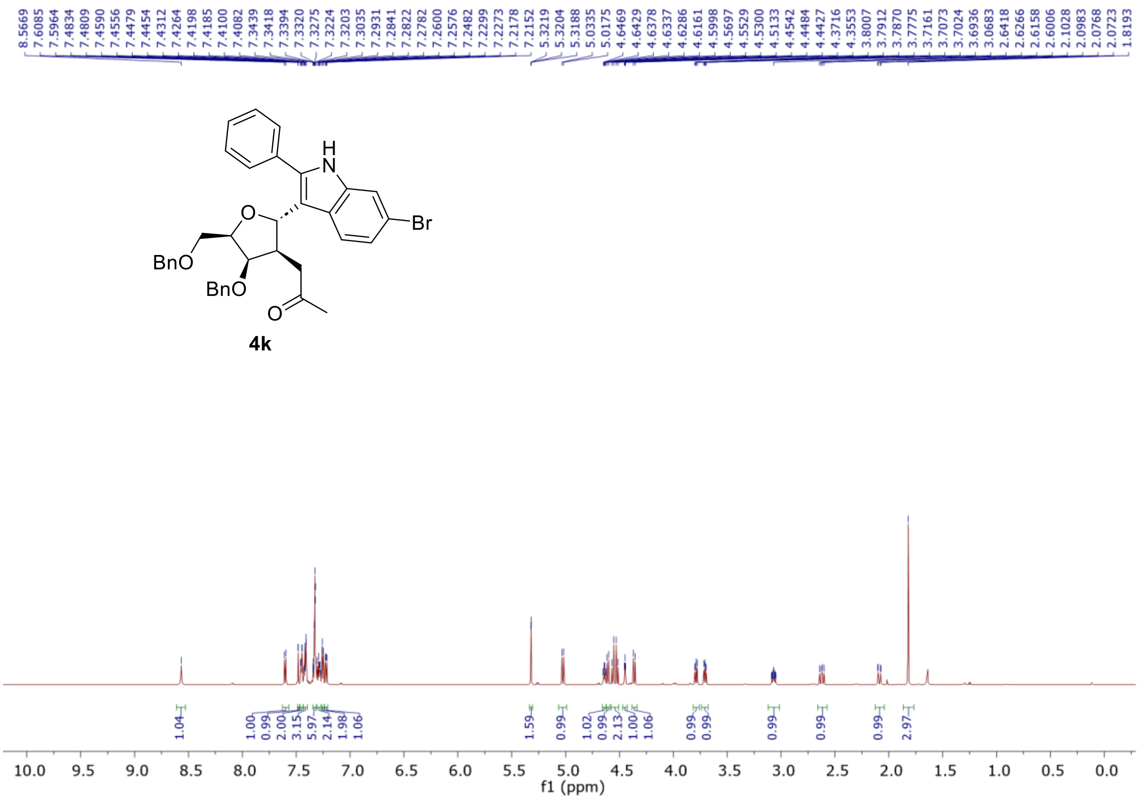

Supplementary Figure S180: ¹H NMR spectra for 4k

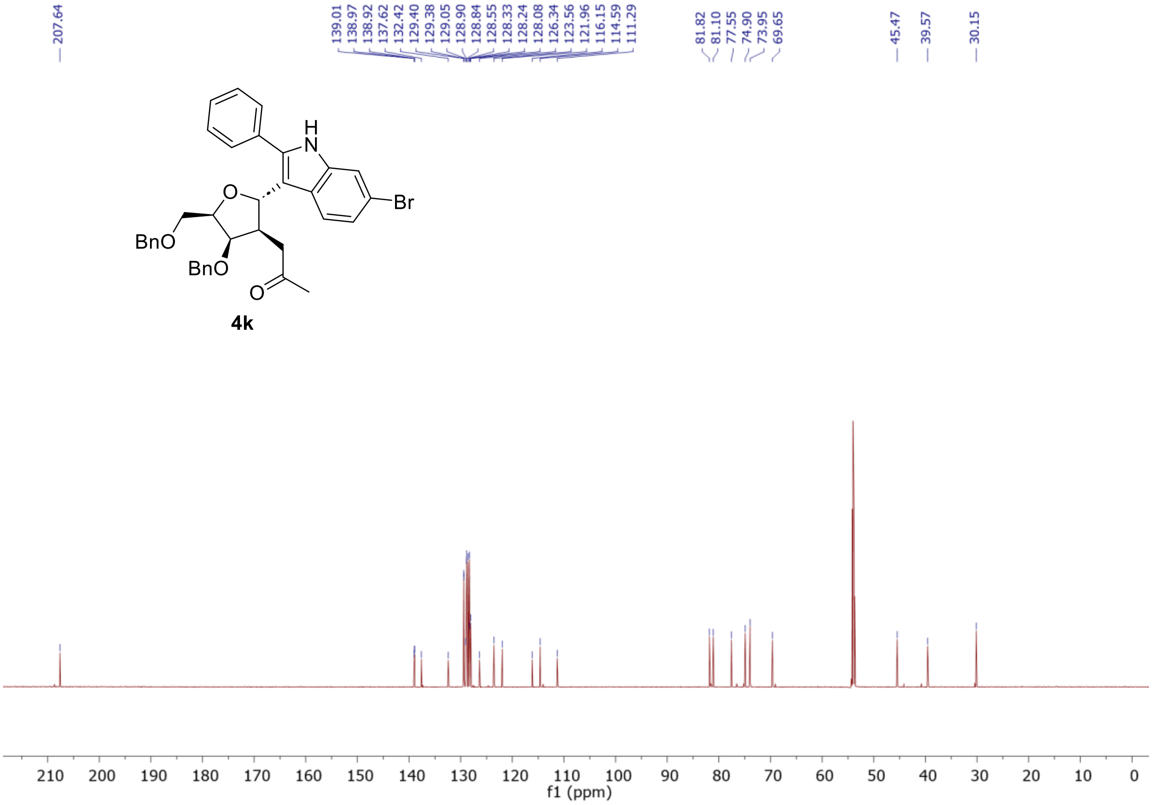

Supplementary Figure S181: ¹³C NMR spectra for 4k

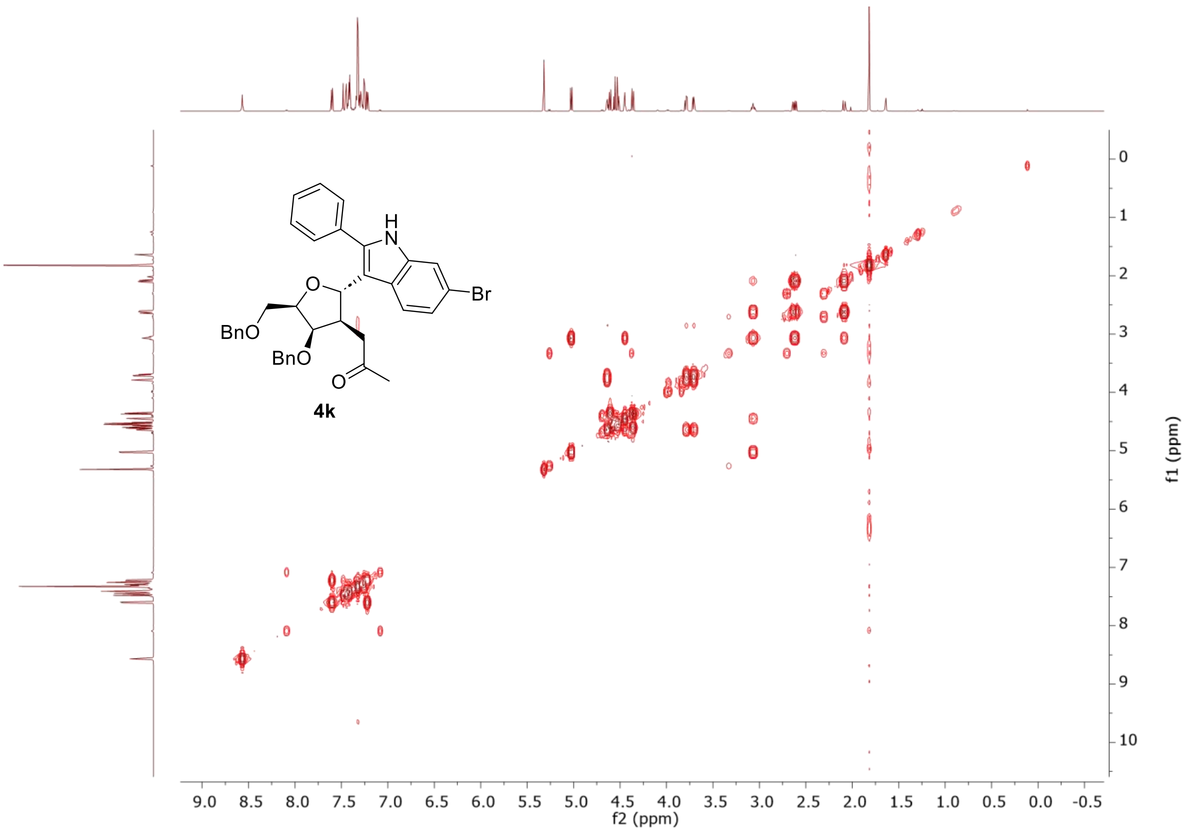

Supplementary Figure S182: COSY spectra for 4k

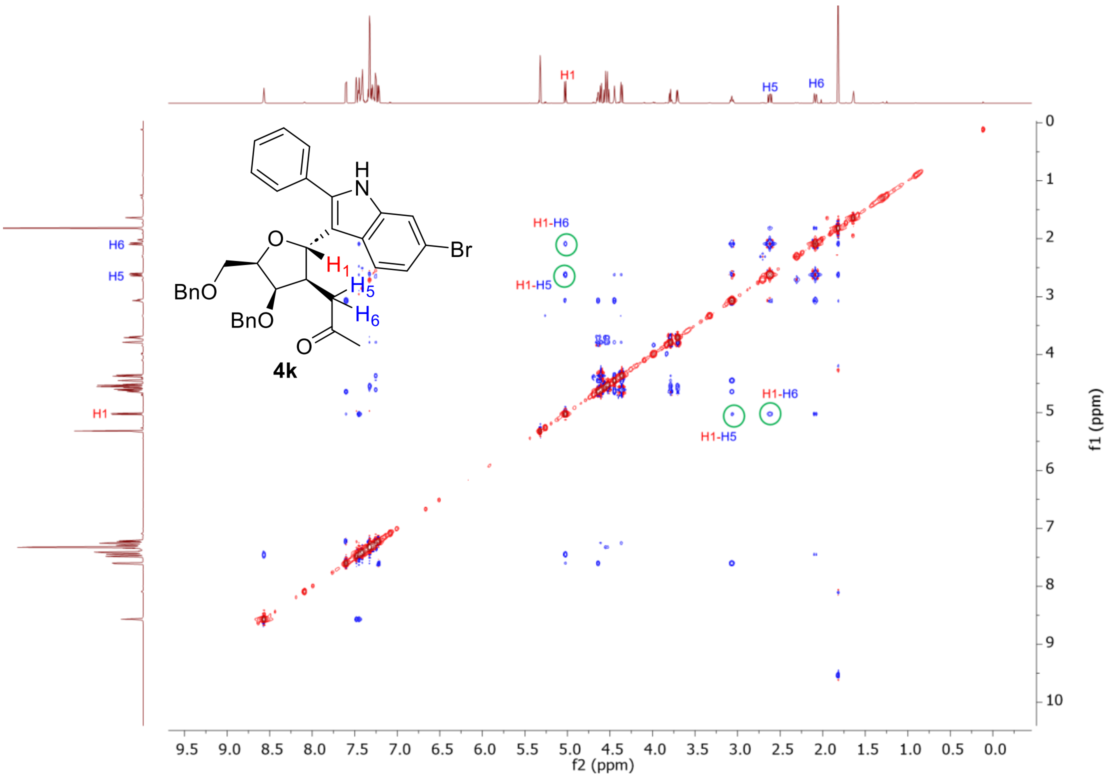

Supplementary Figure S183: NOESY spectra for 4k

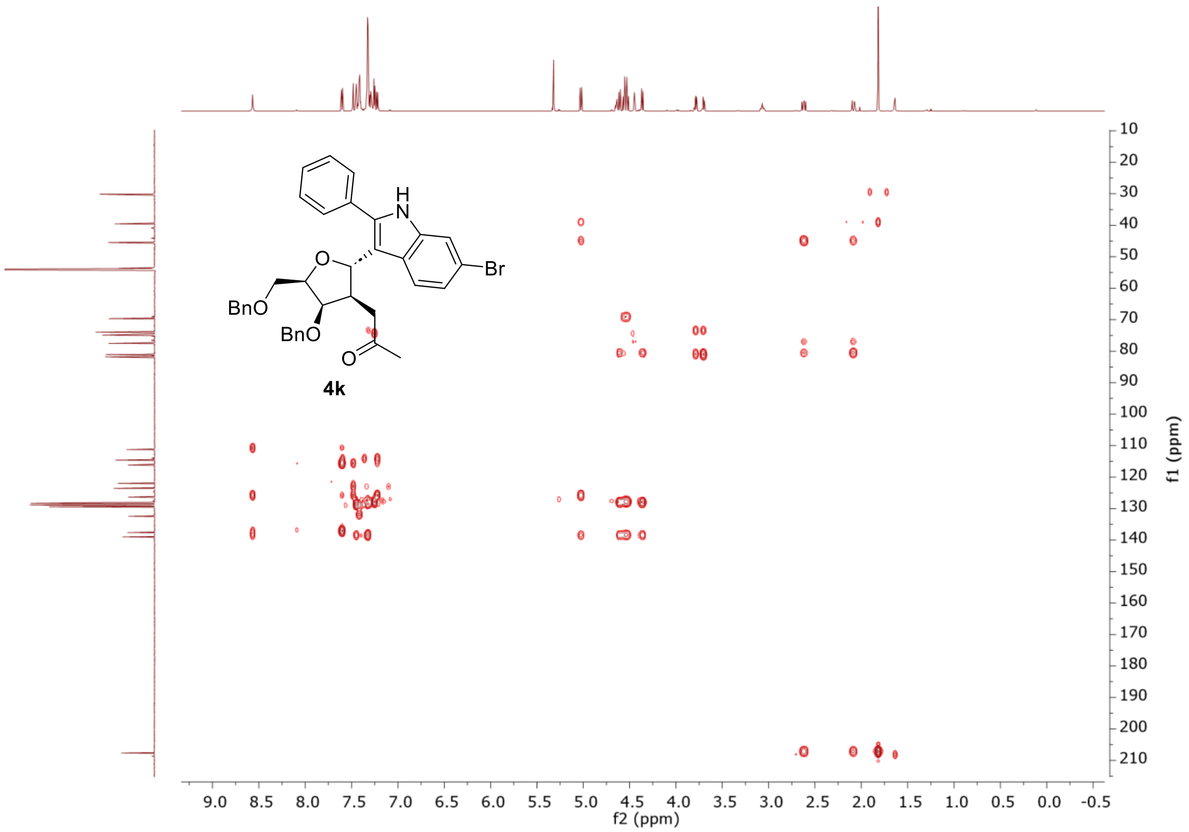

Supplementary Figure S184: HMBC spectra for 4k

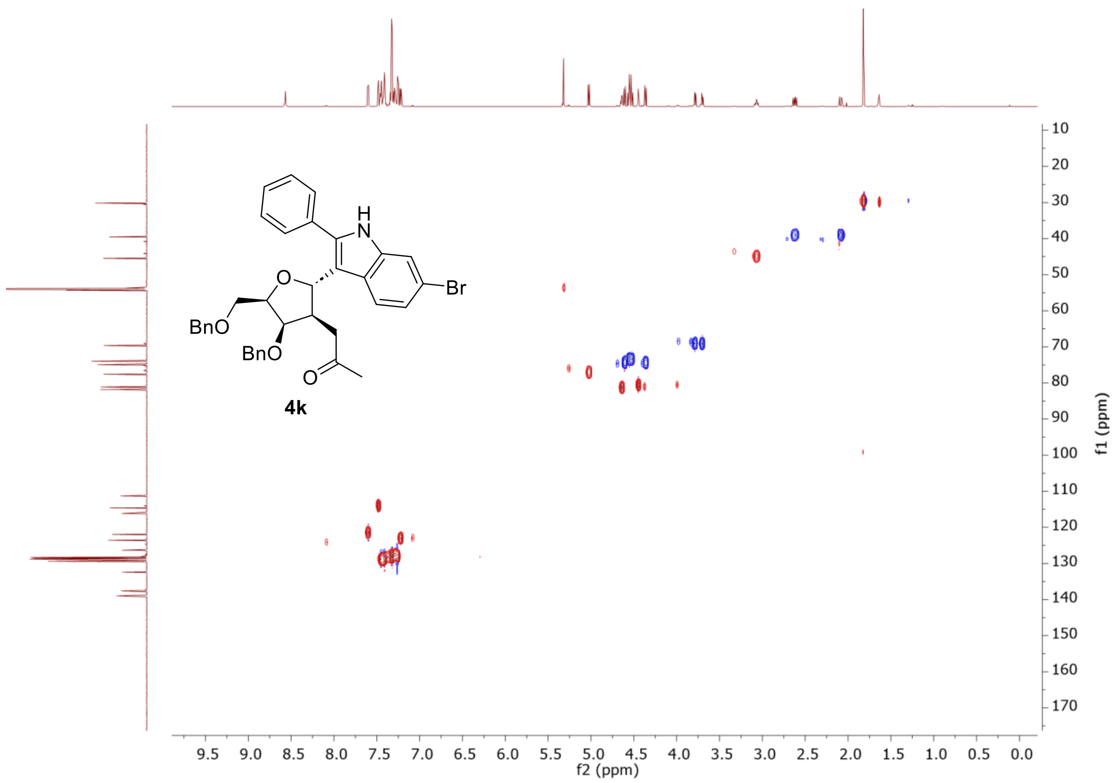

Supplementary Figure S185: HSQC spectra for 4k

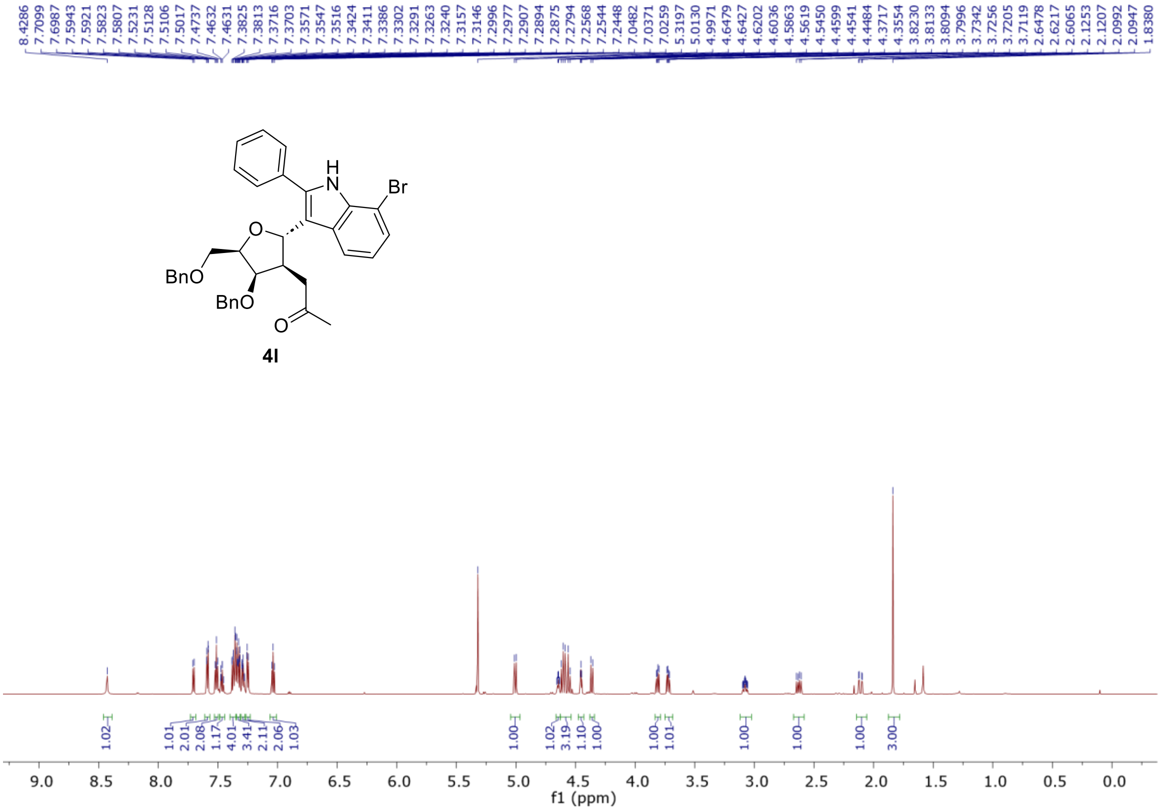

Supplementary Figure S186: ^{1}H NMR spectra for 4l

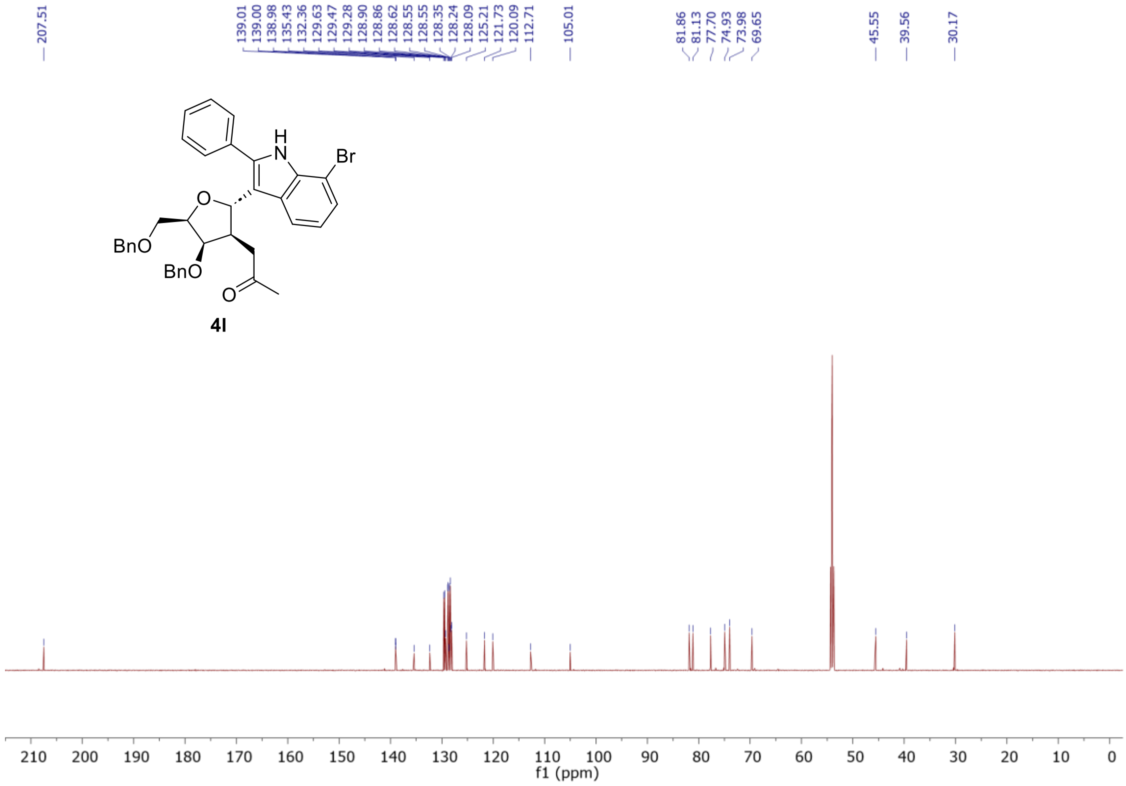

Supplementary Figure S187: ^{13}C NMR spectra for 4l

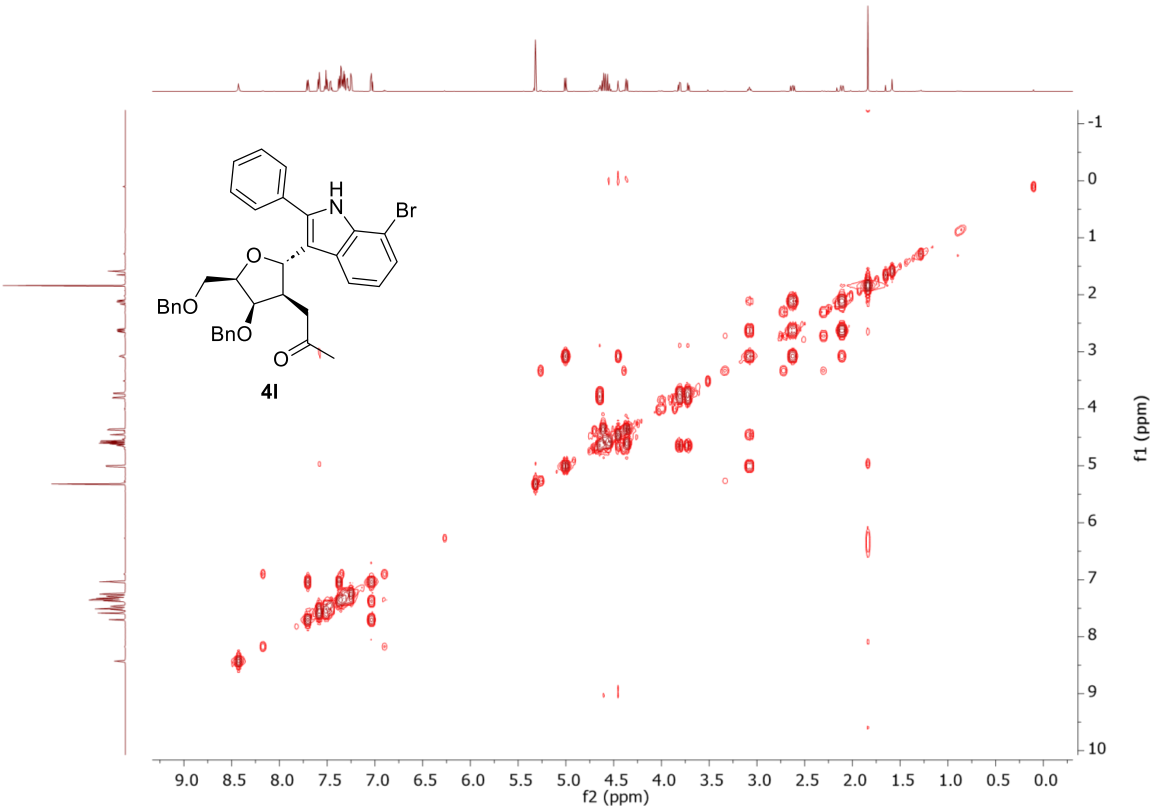

Supplementary Figure S188: COSY spectra for 4l

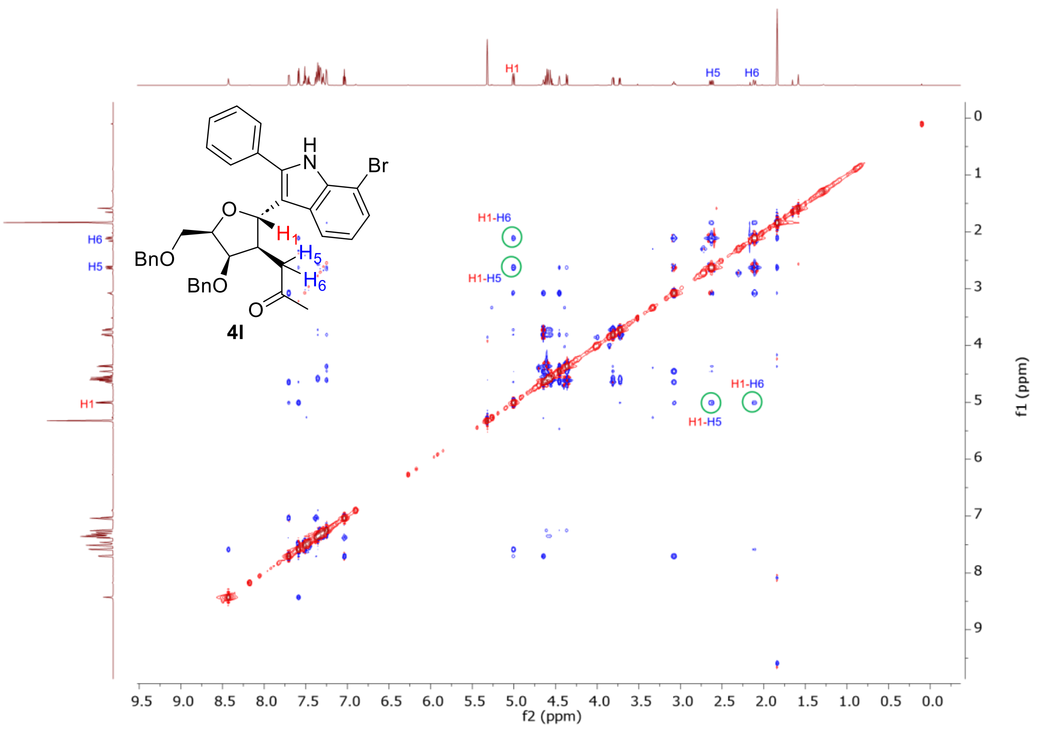

Supplementary Figure S189: NOESY spectra for 4l

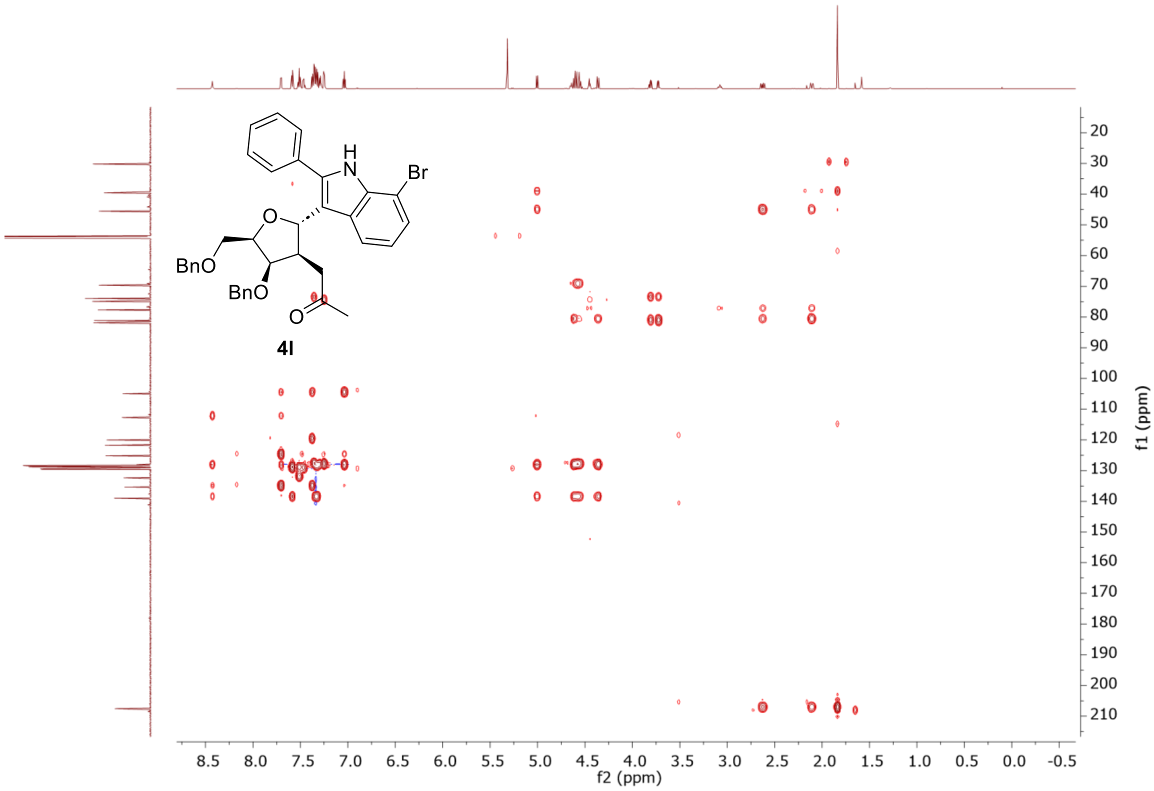

Supplementary Figure S190: HMBC spectra for 4l

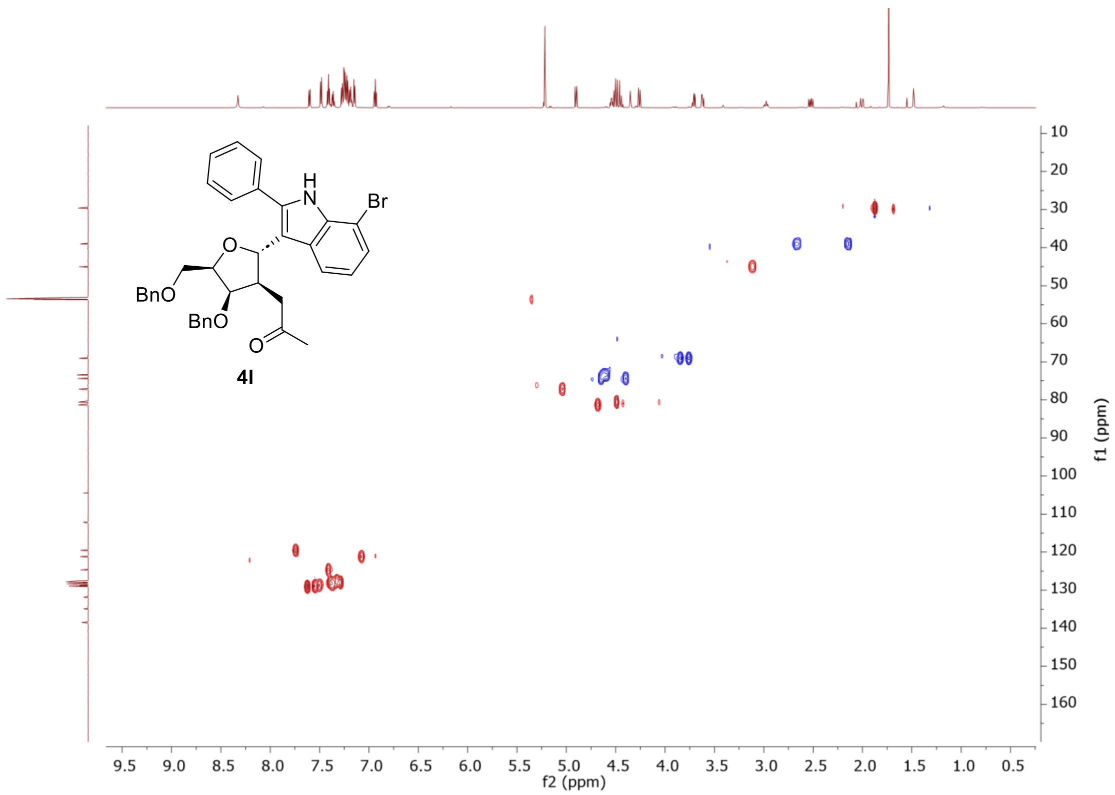

Supplementary Figure S191: HSQC spectra for 4l

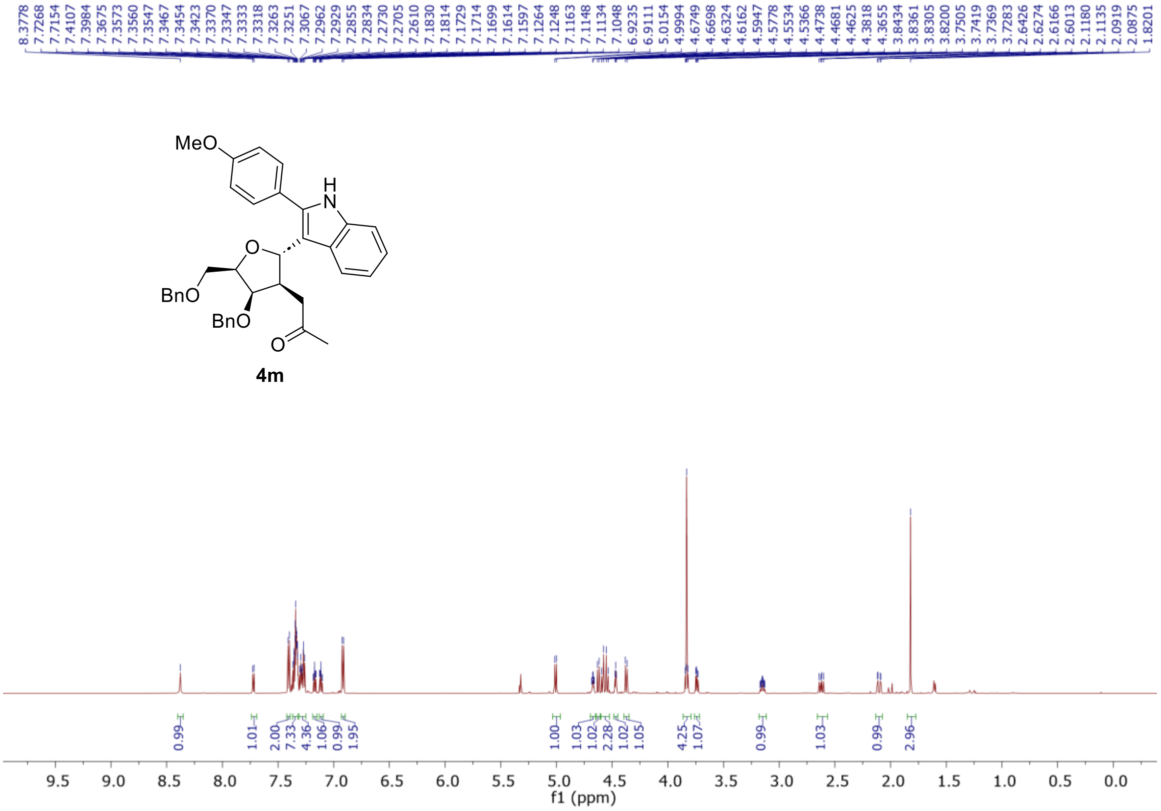

Supplementary Figure S192: ^{1}H NMR spectra for 4m

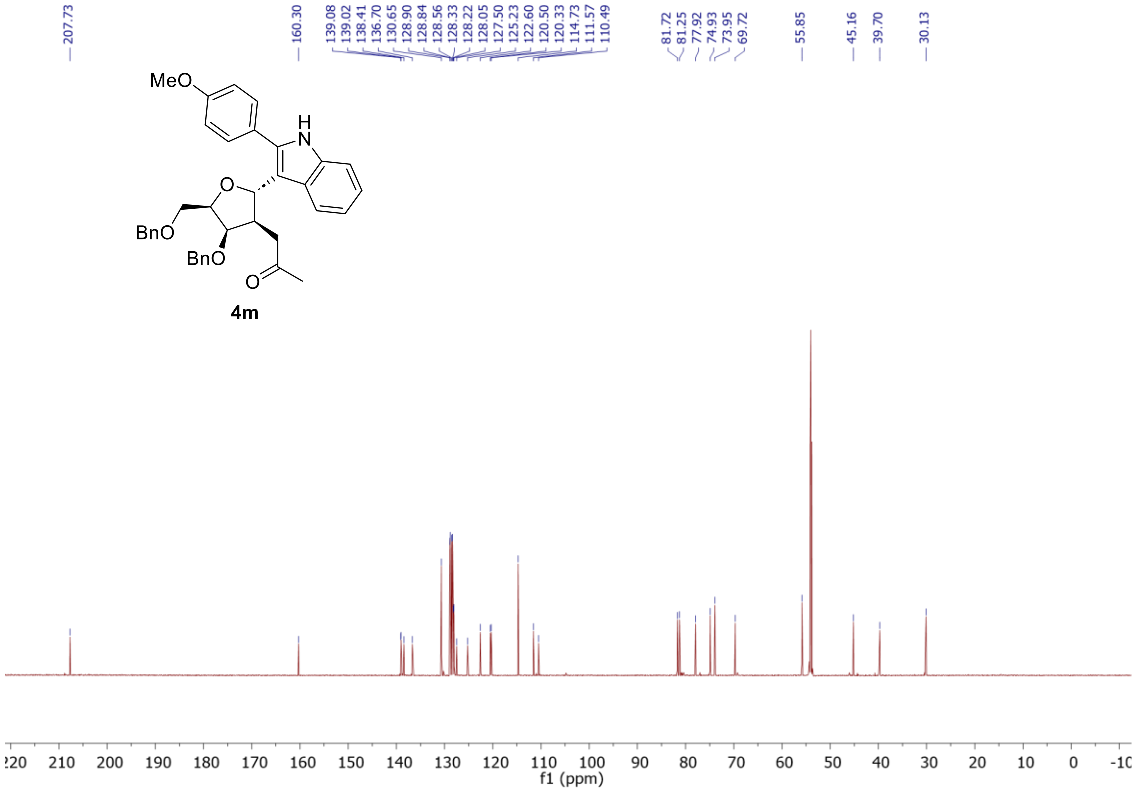

Supplementary Figure S193: ^{13}C NMR spectra for 4m

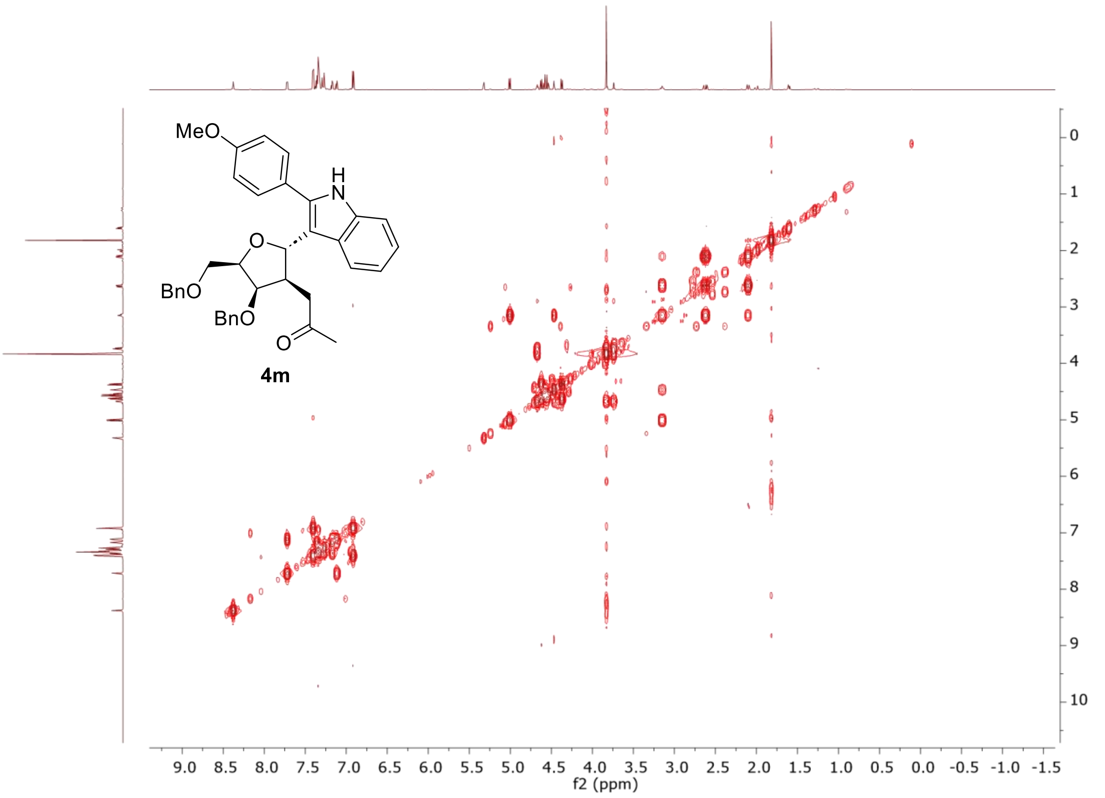

Supplementary Figure S194: COSY spectra for 4m

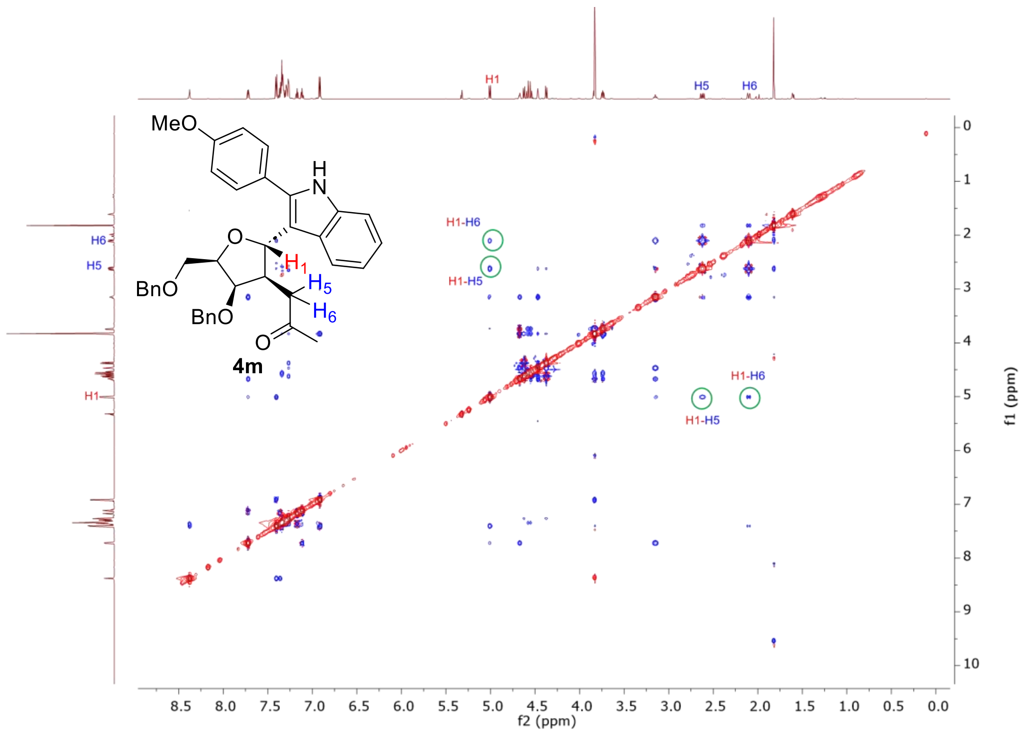

Supplementary Figure S195: NOESY spectra for 4m

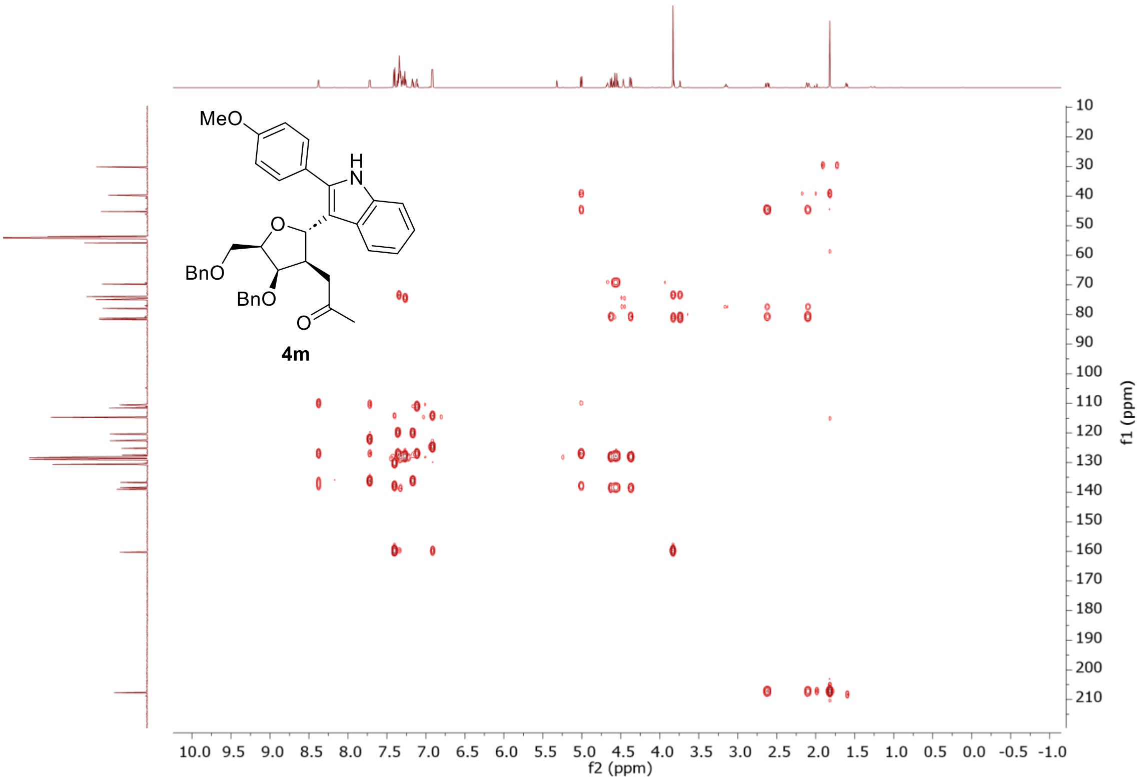

Supplementary Figure S196: HMBC spectra for 4m

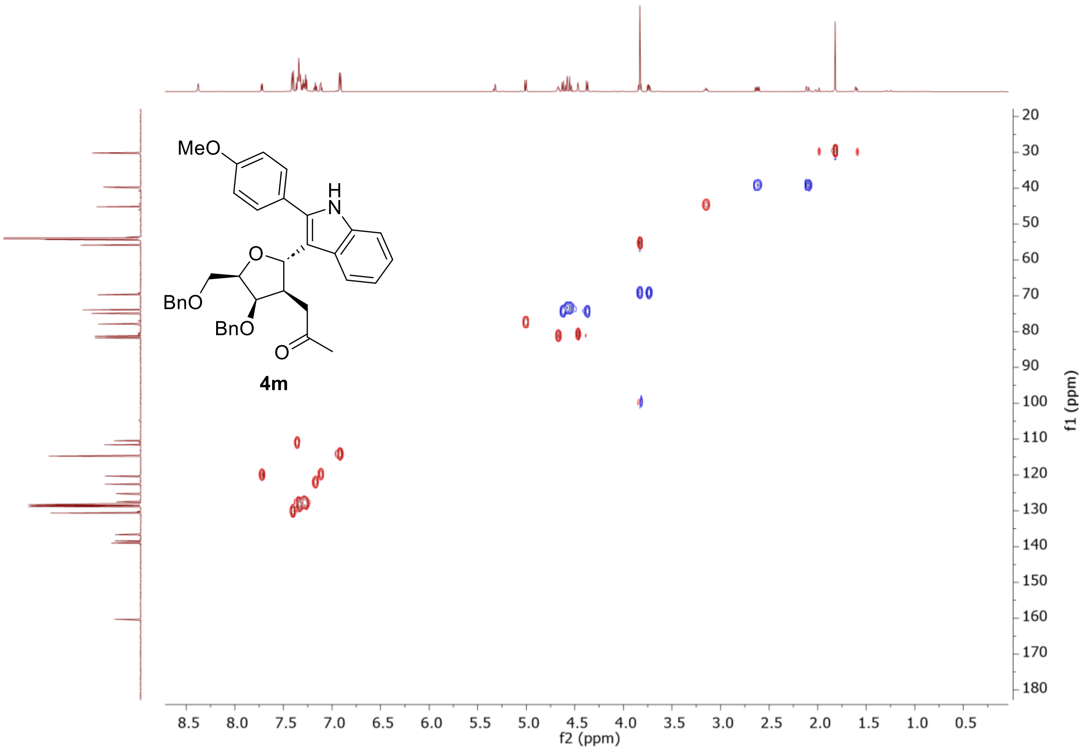

Supplementary Figure S197: HSQC spectra for 4m

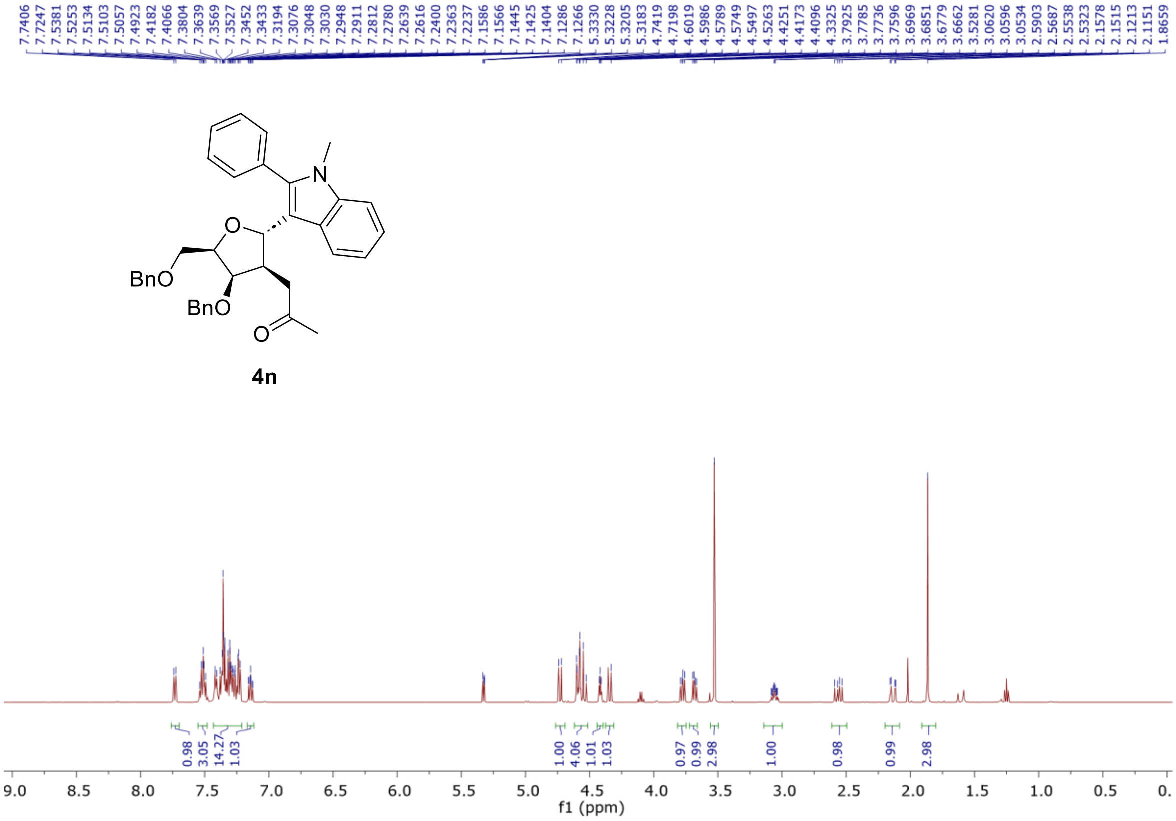

Supplementary Figure S198: ^{1}H NMR spectra for 4n

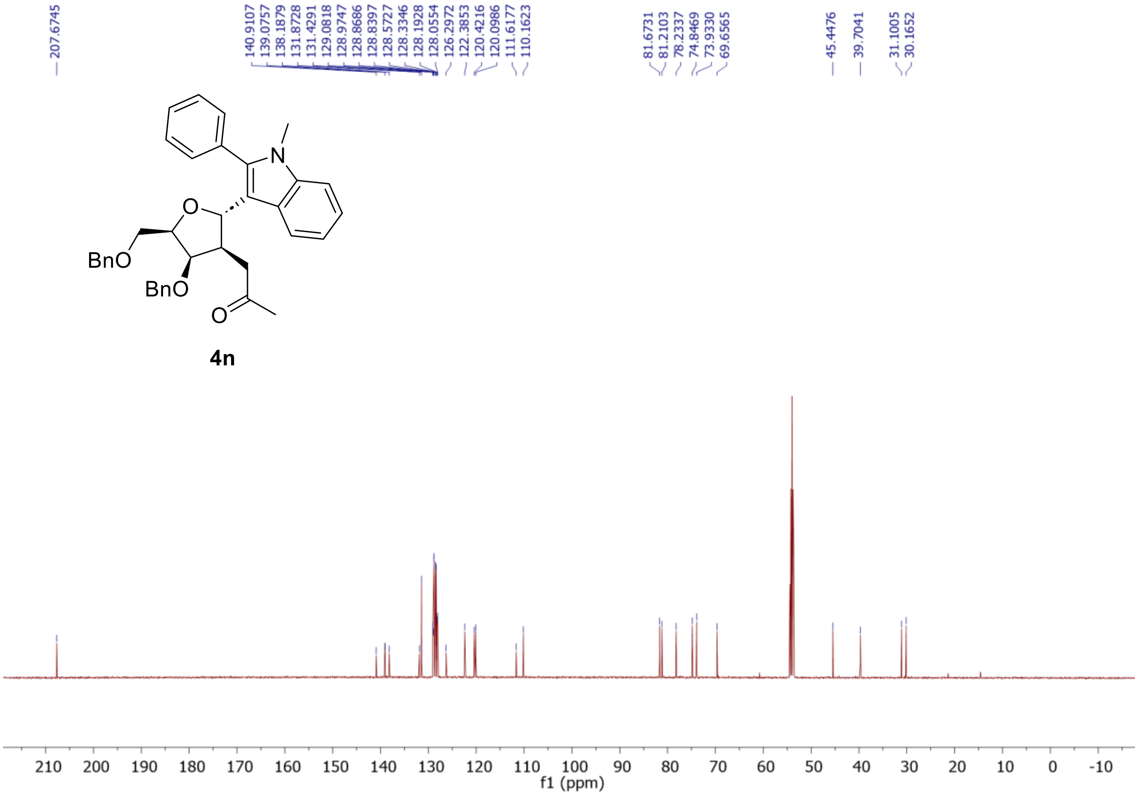

Supplementary Figure S199: ^{13}C NMR spectra for 4n

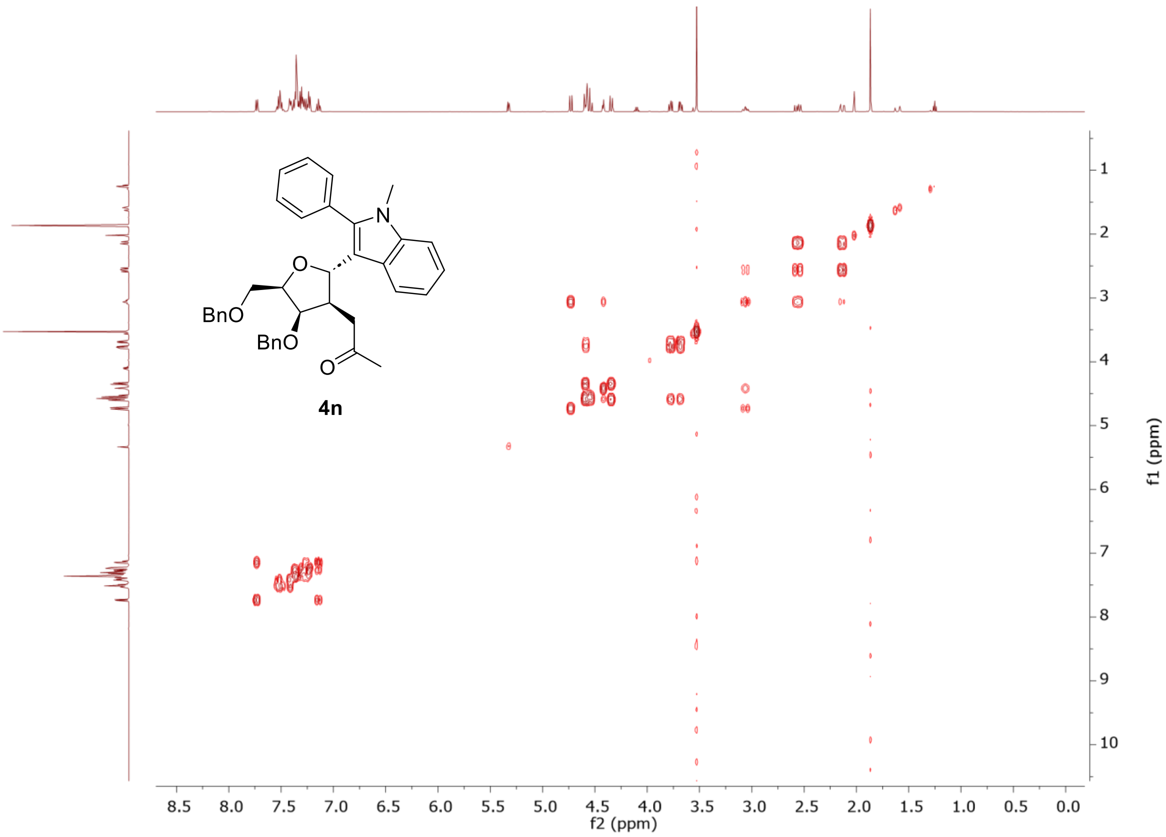

Supplementary Figure S200: COSY spectra for 4n

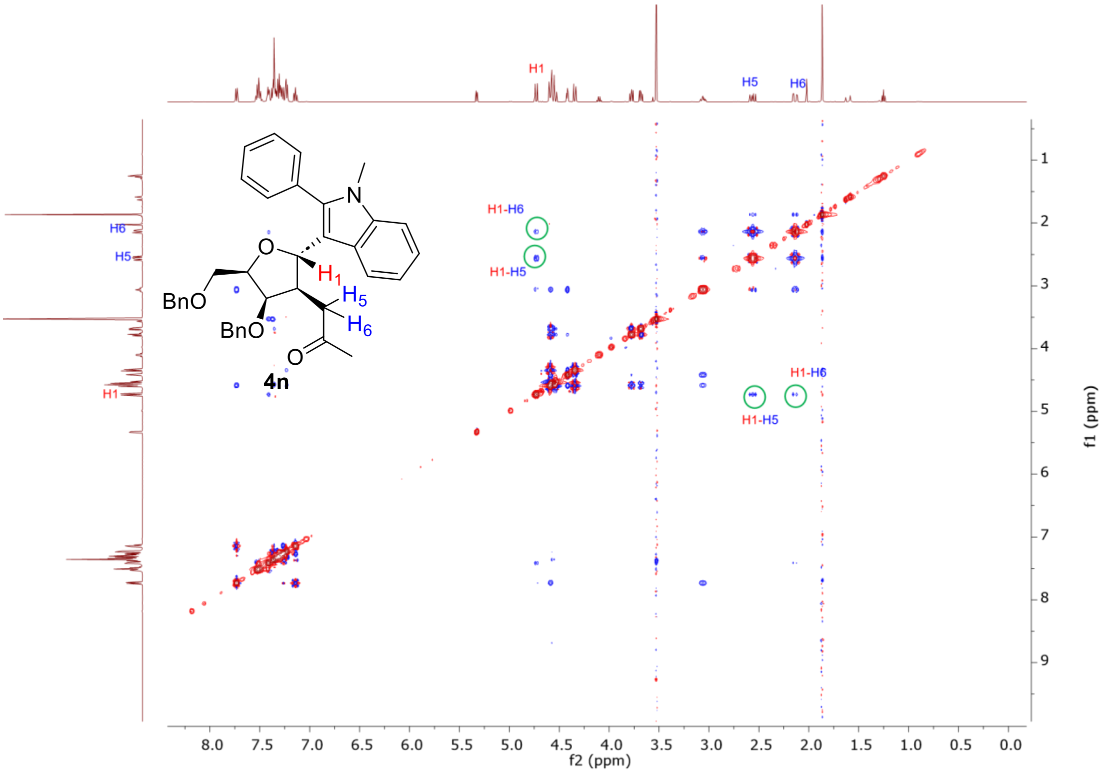

Supplementary Figure S201: NOESY spectra for 4n

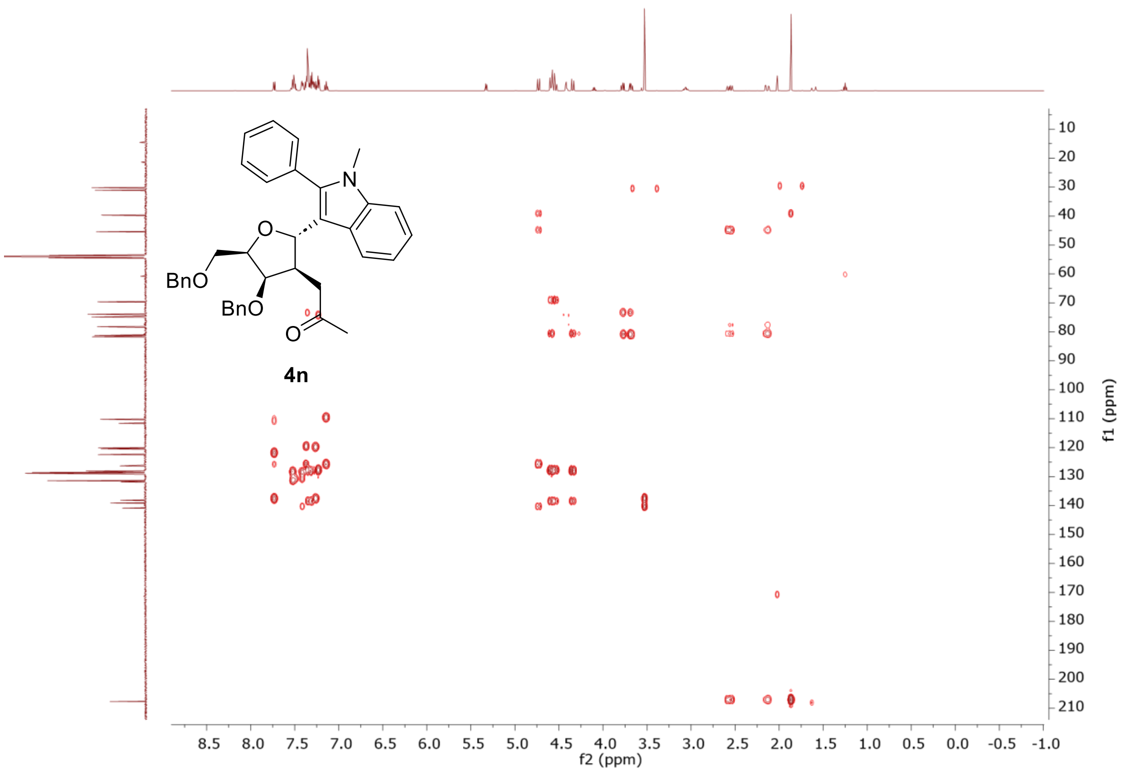

Supplementary Figure S202: HMBC spectra for 4n

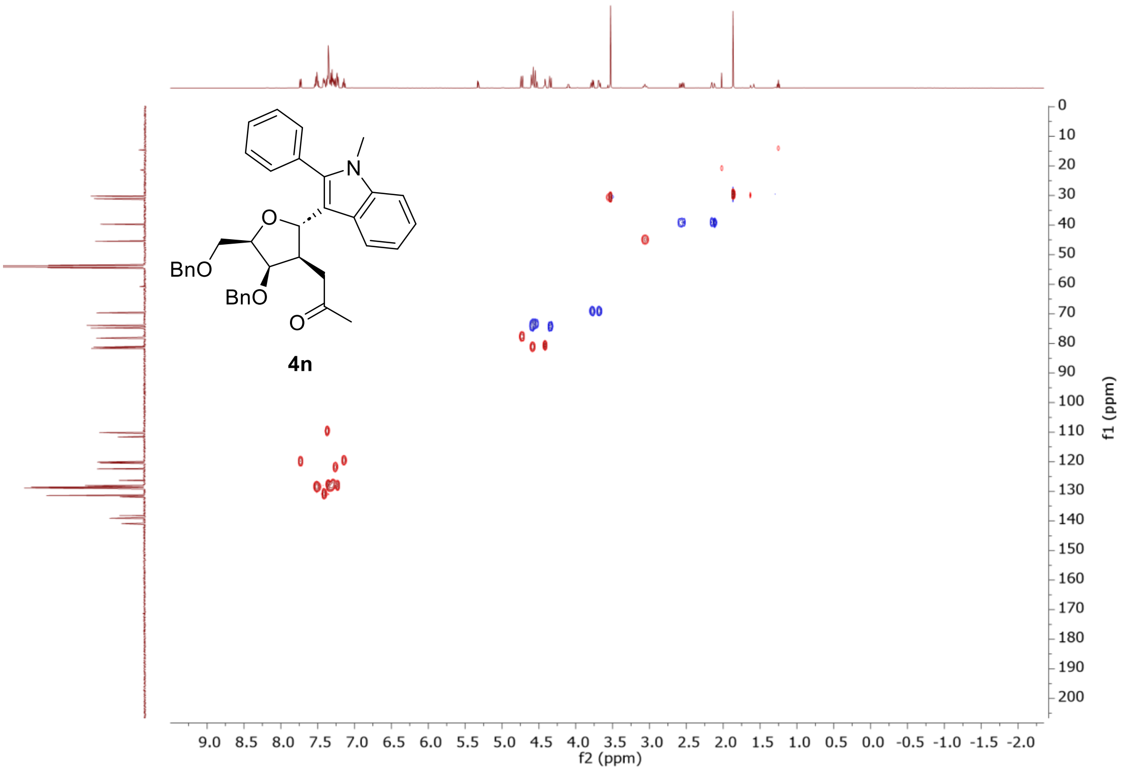

Supplementary Figure S203: HSQC spectra for 4n

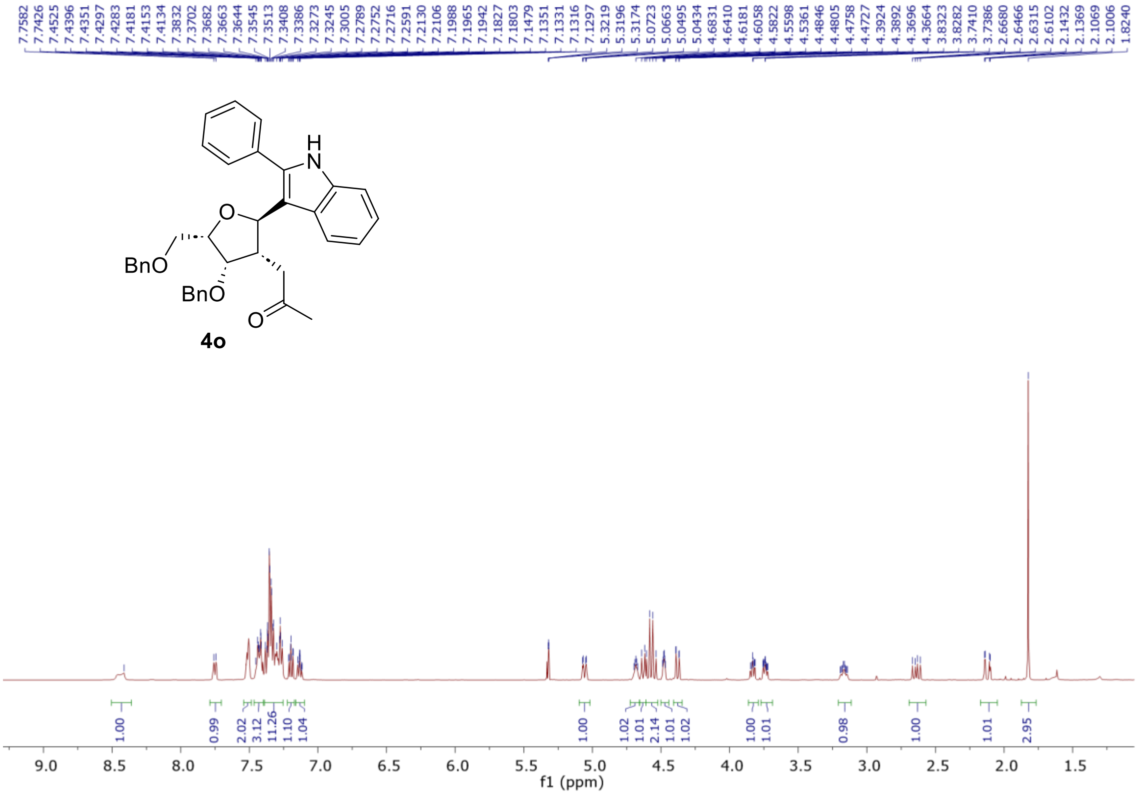

Supplementary Figure S204: ^{1}H NMR spectra for 4o

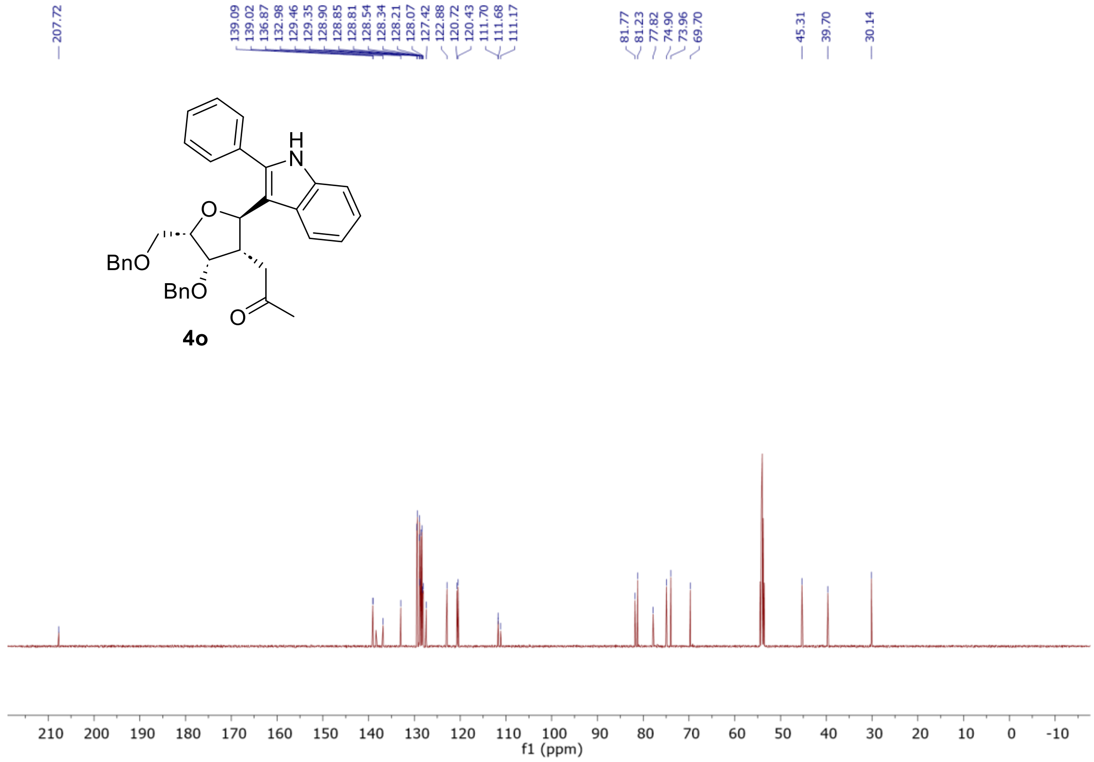

Supplementary Figure S205: ^{13}C NMR spectra for 4o

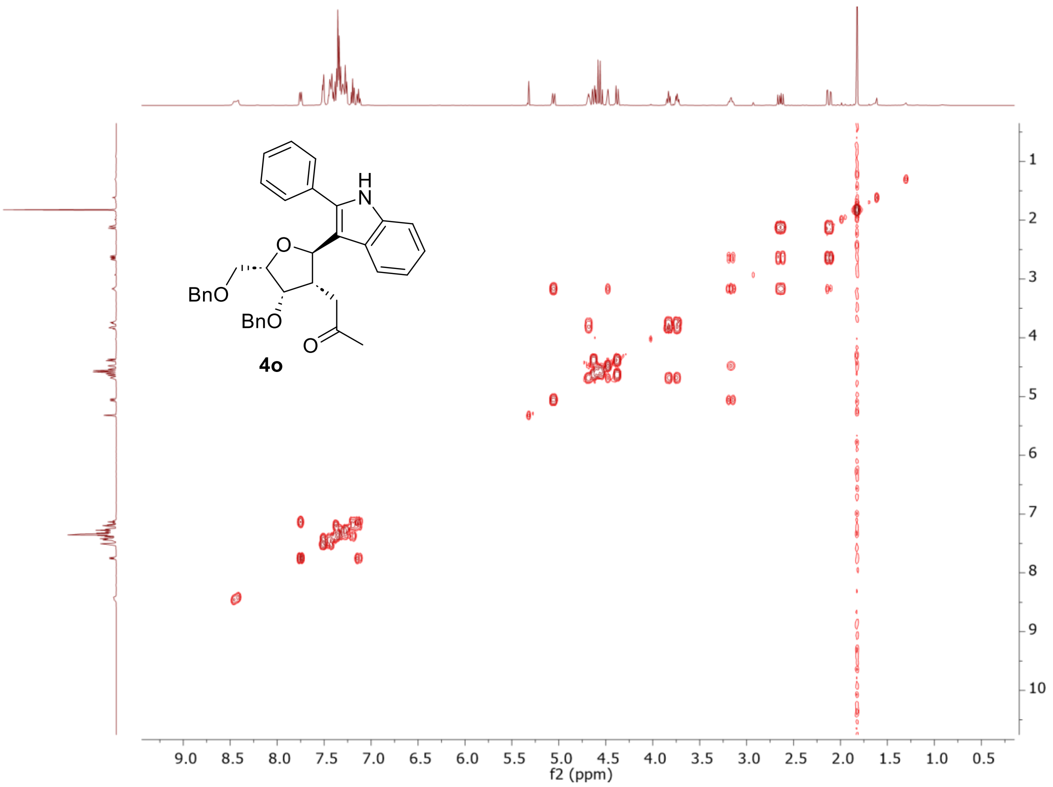

Supplementary Figure S206: COSY spectra for 4o

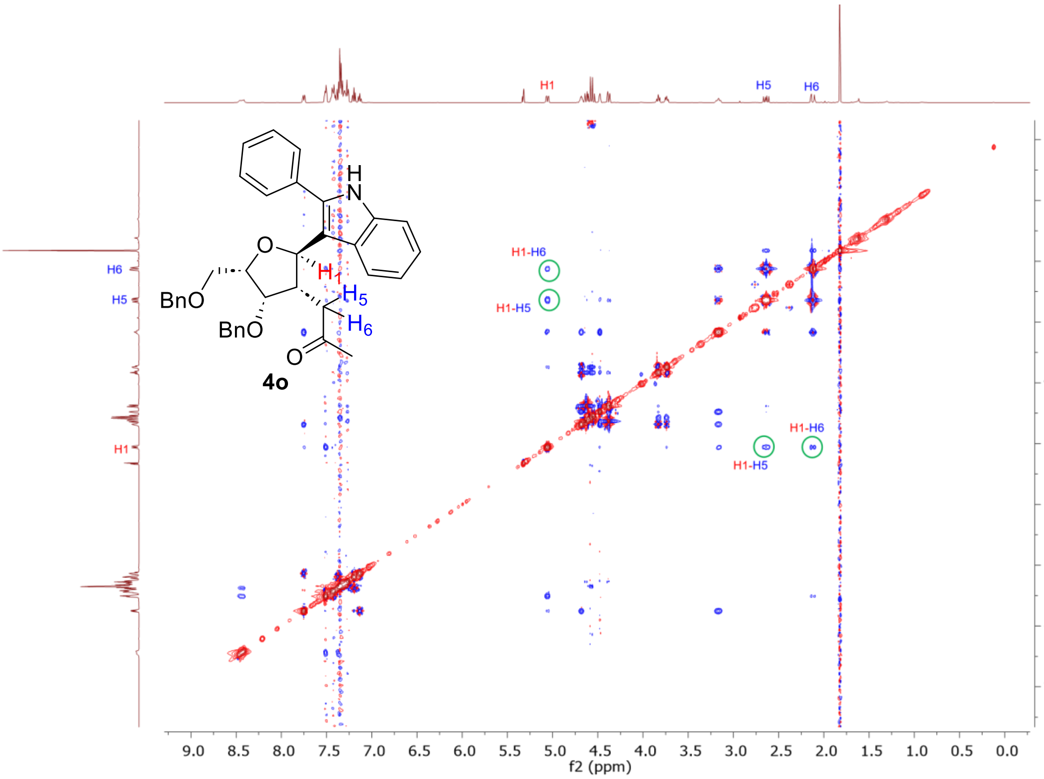

Supplementary Figure S207: NOESY spectra for 4o

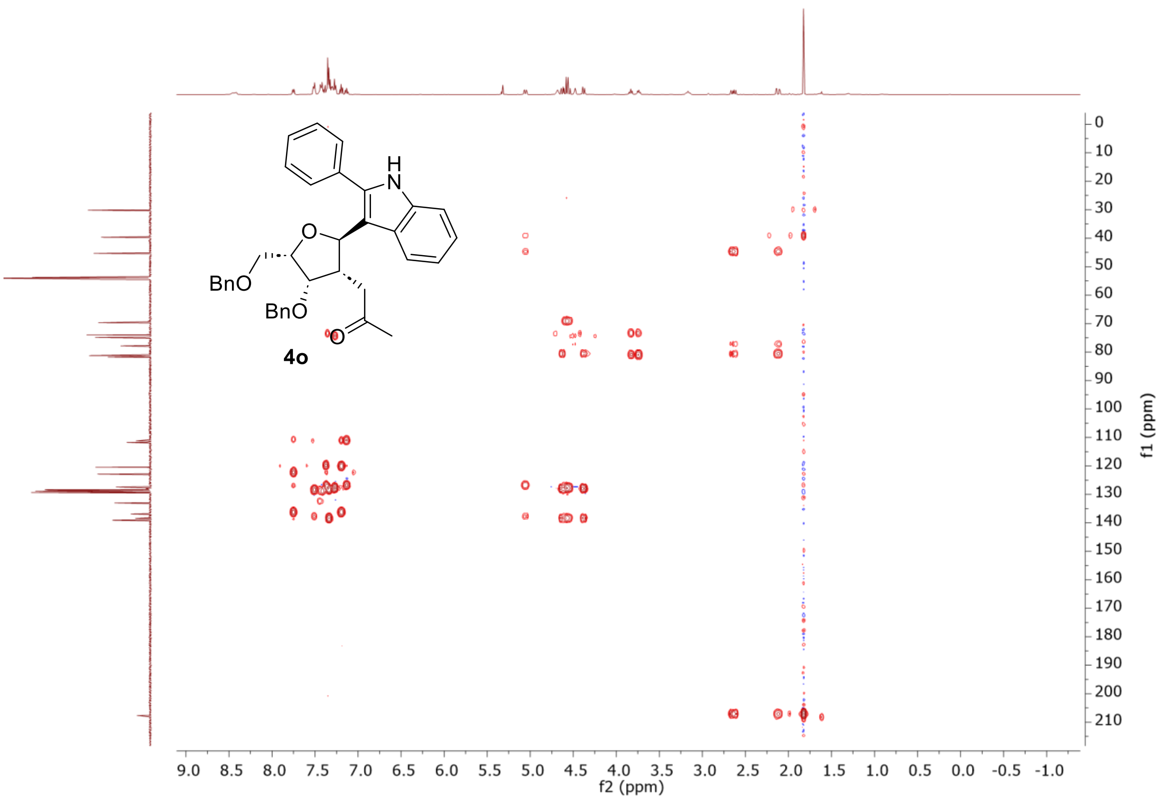

Supplementary Figure S208: HMBC spectra for 4o

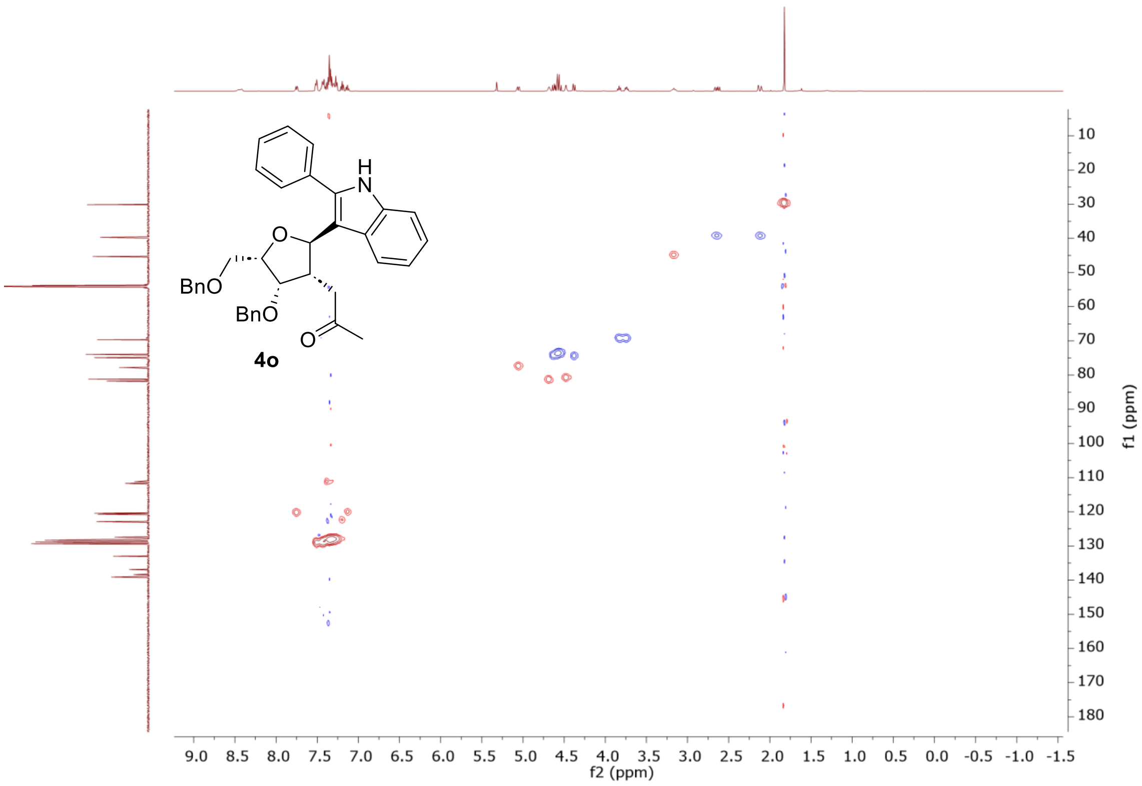

Supplementary Figure S209: HSQC spectra for 4o

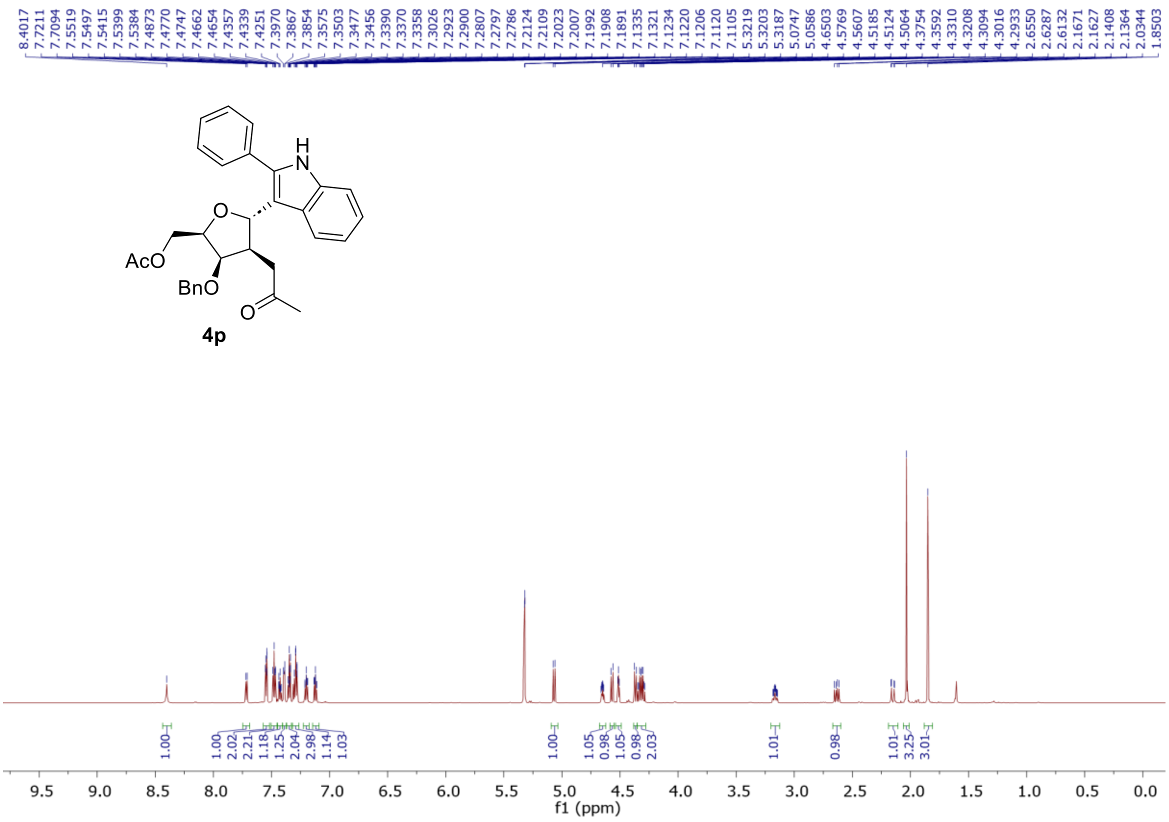

Supplementary Figure S210: ^{1}H NMR spectra for 4p

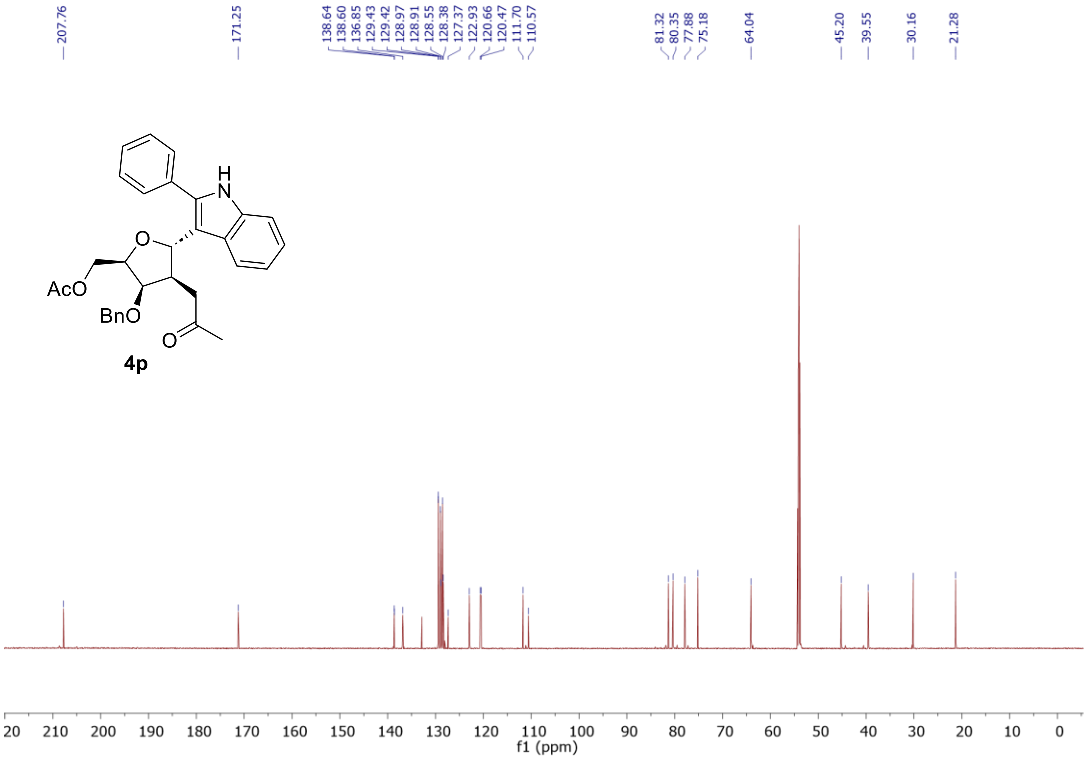

Supplementary Figure S211: ^{13}C NMR spectra for 4p

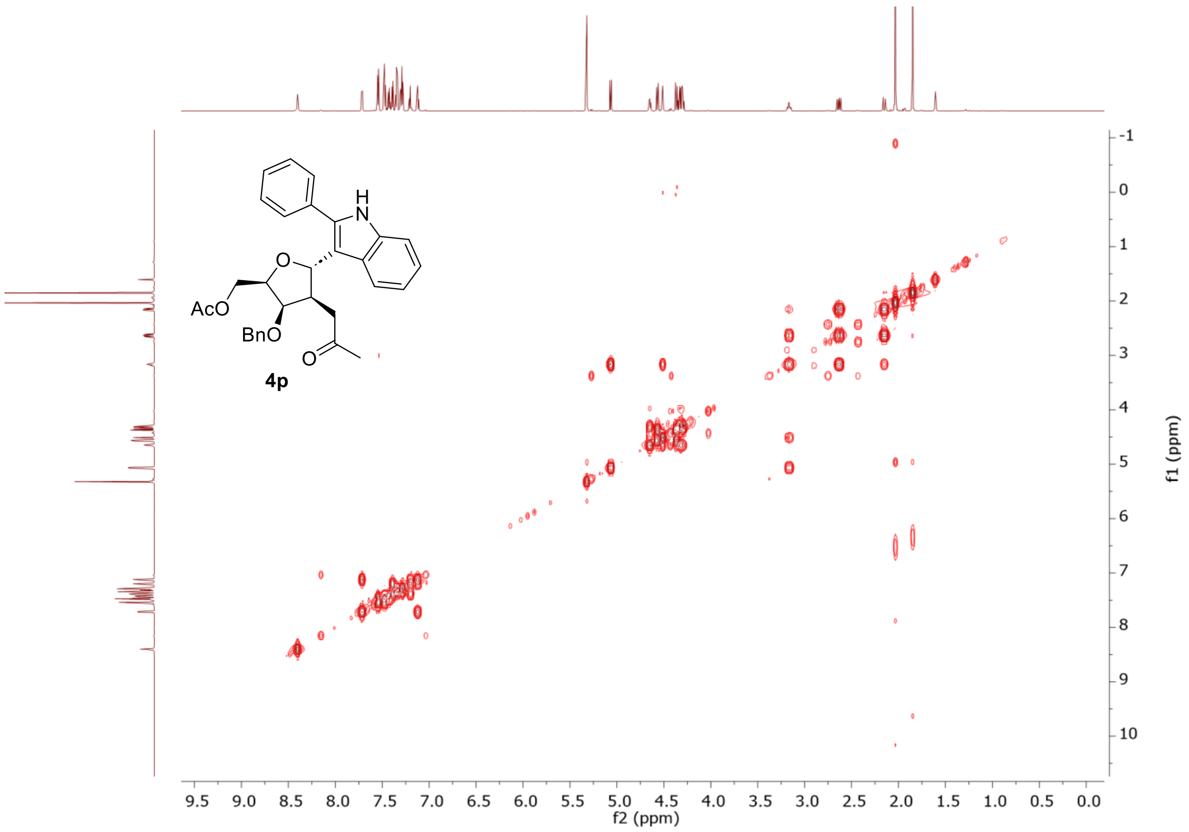

Supplementary Figure S212: COSY spectra for 4p

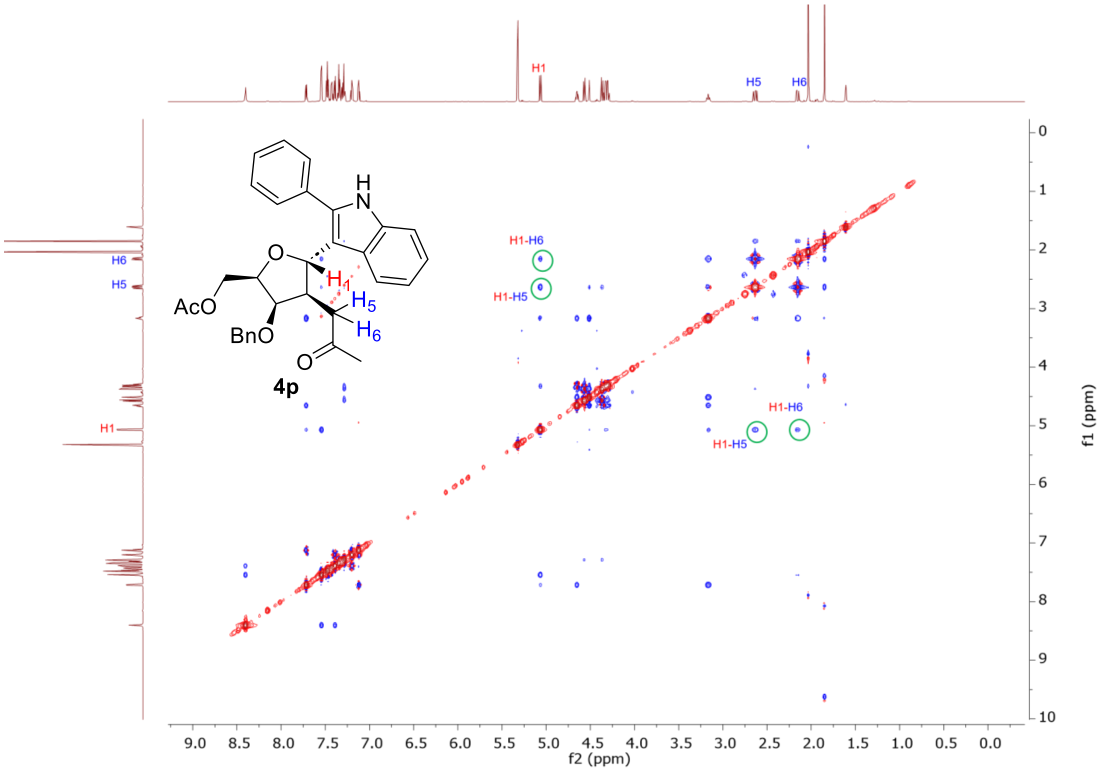

Supplementary Figure S213: NOESY spectra for 4p

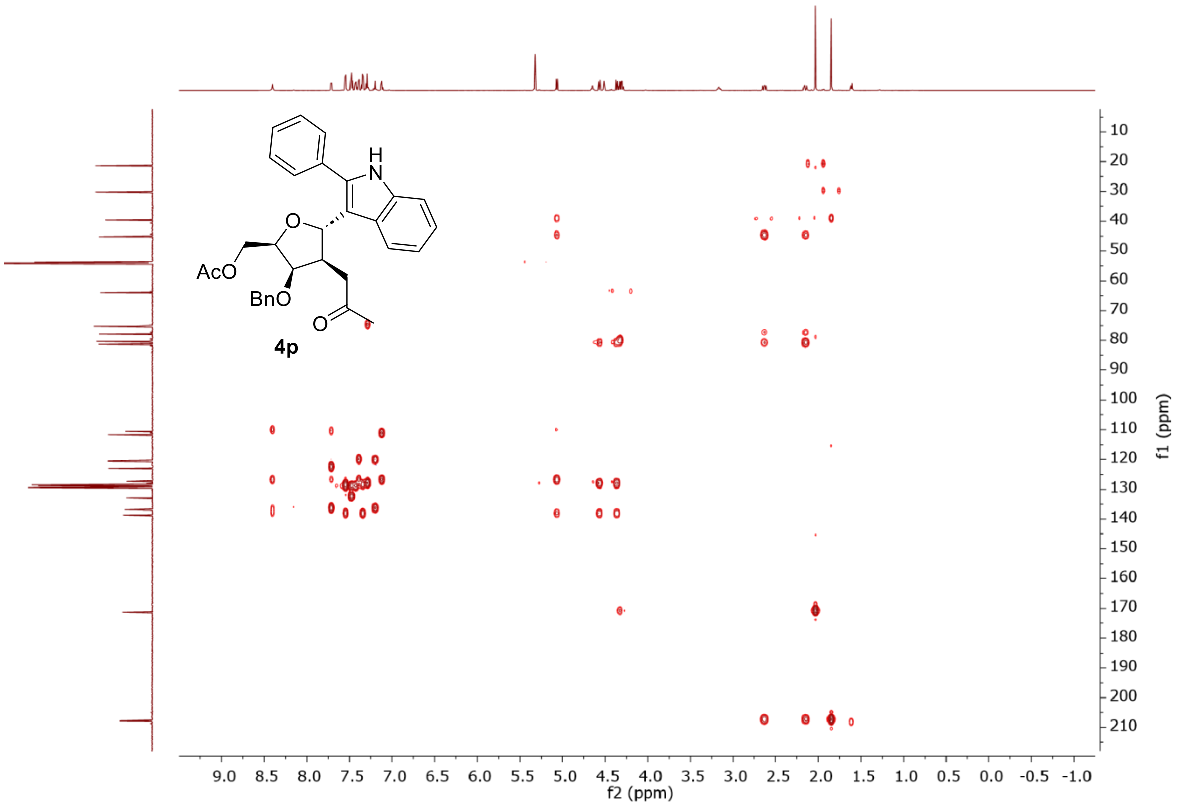

Supplementary Figure S214: HMBC spectra for 4p

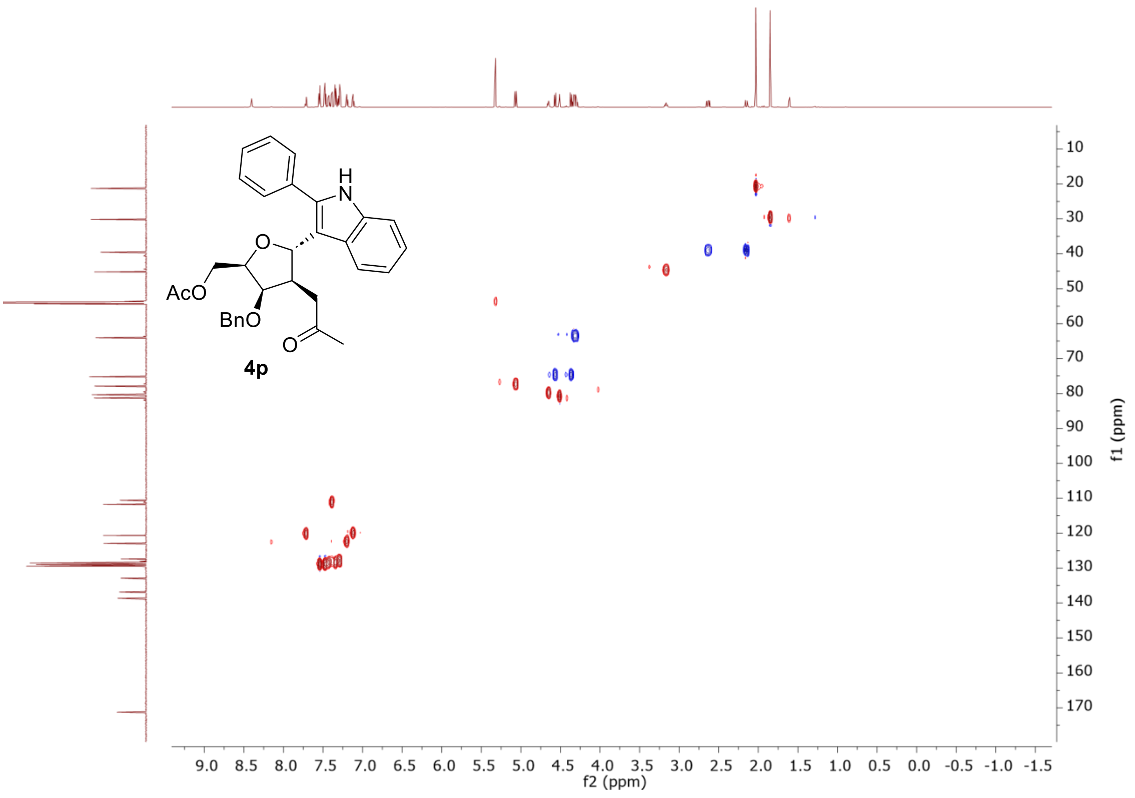

Supplementary Figure S215: HSQC spectra for 4p

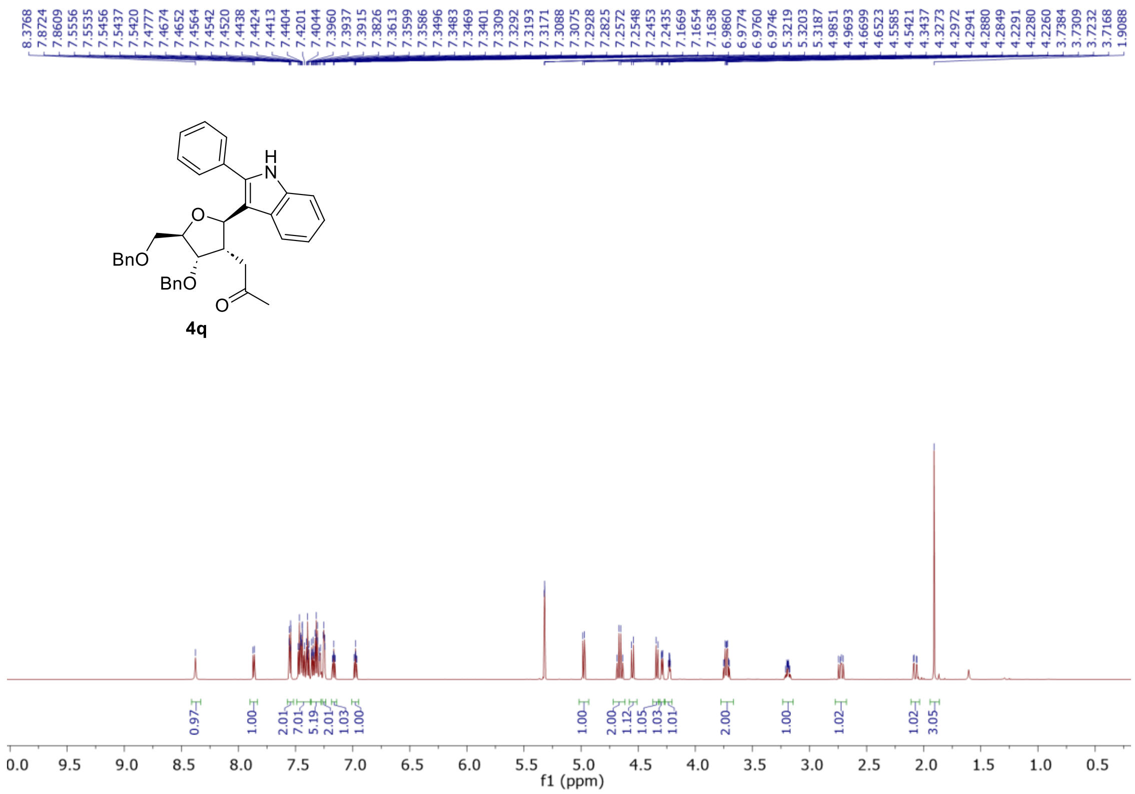

Supplementary Figure S216: ^{1}H NMR spectra for 4q

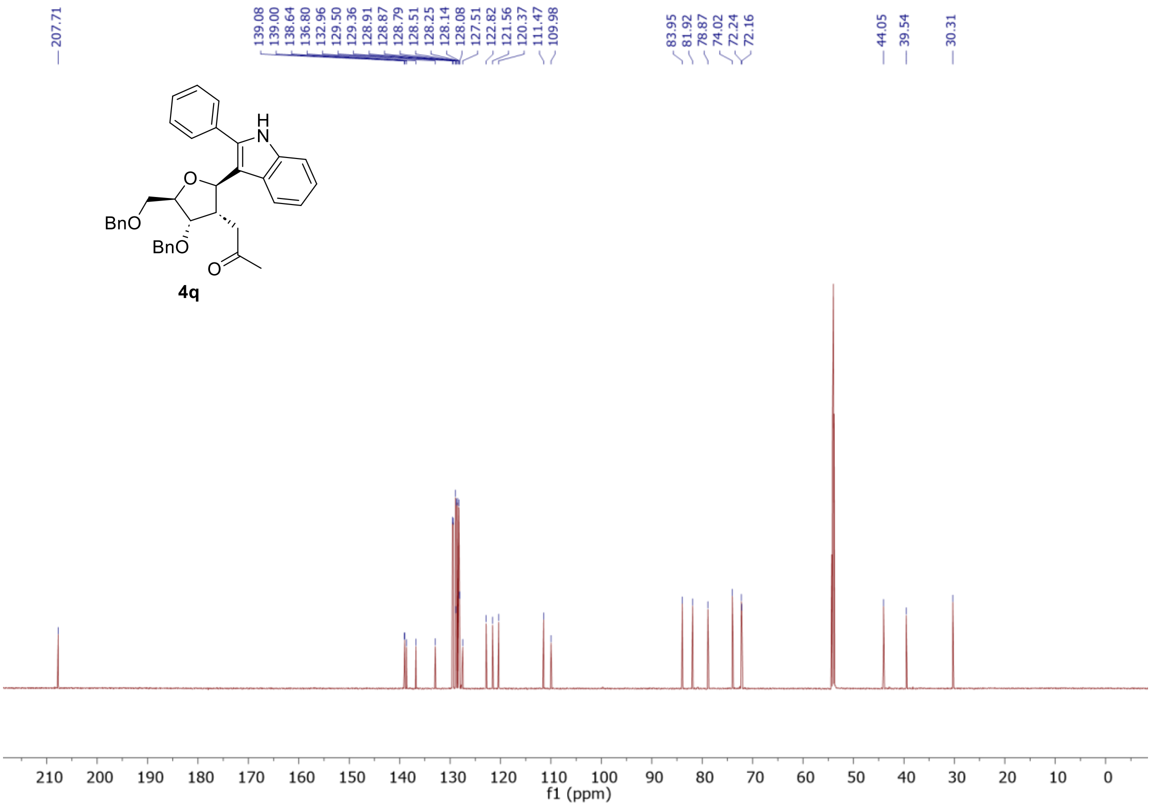

Supplementary Figure S217: ^{13}C NMR spectra for 4q

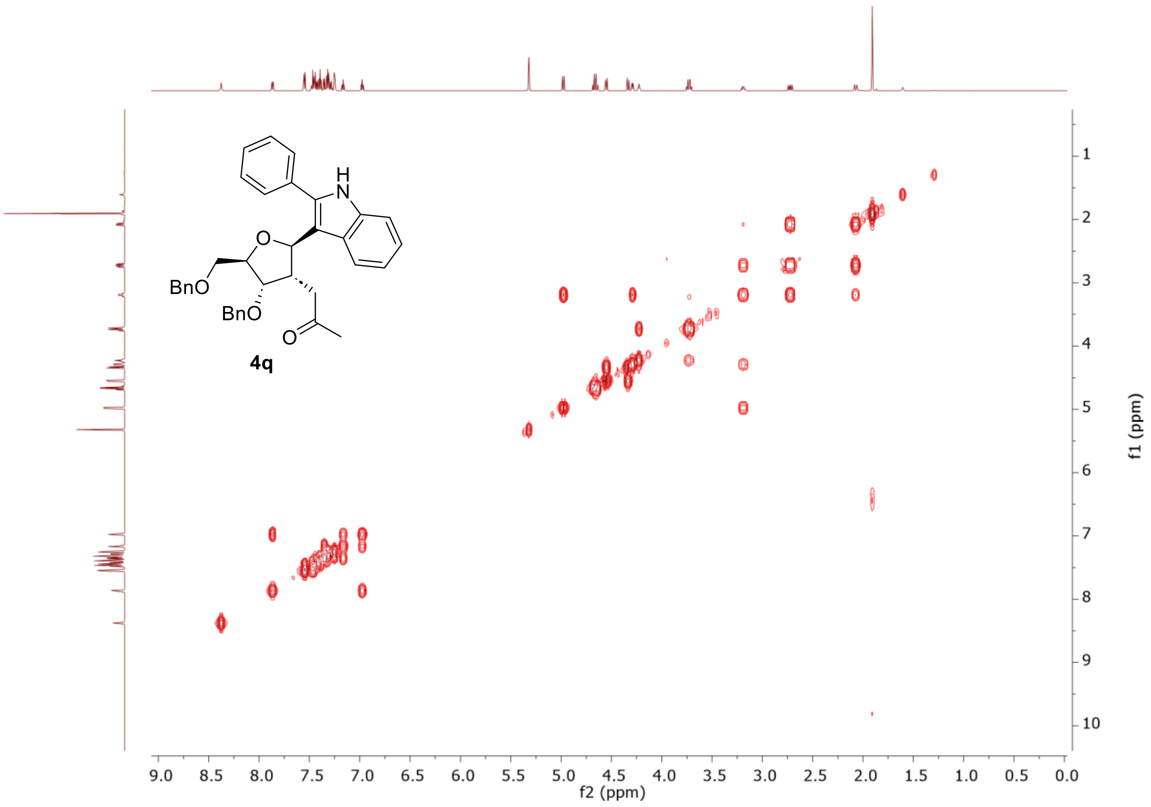

Supplementary Figure S218: COSY spectra for 4q

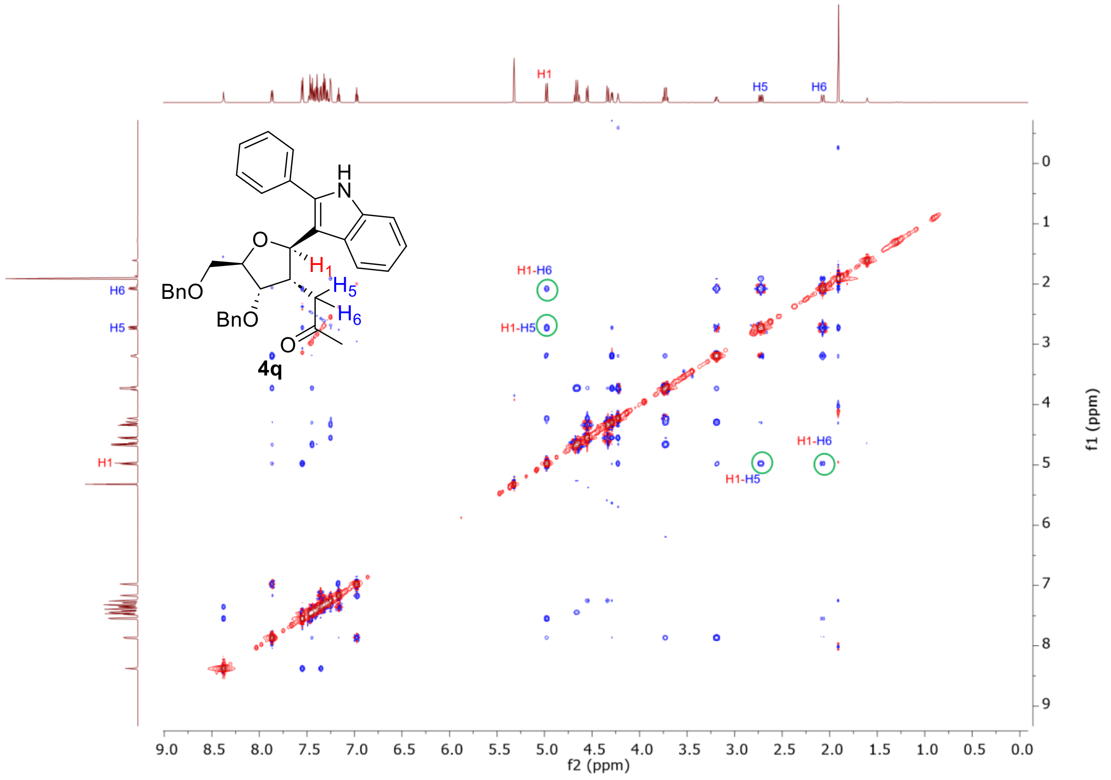

Supplementary Figure S219: NOESY spectra for 4q

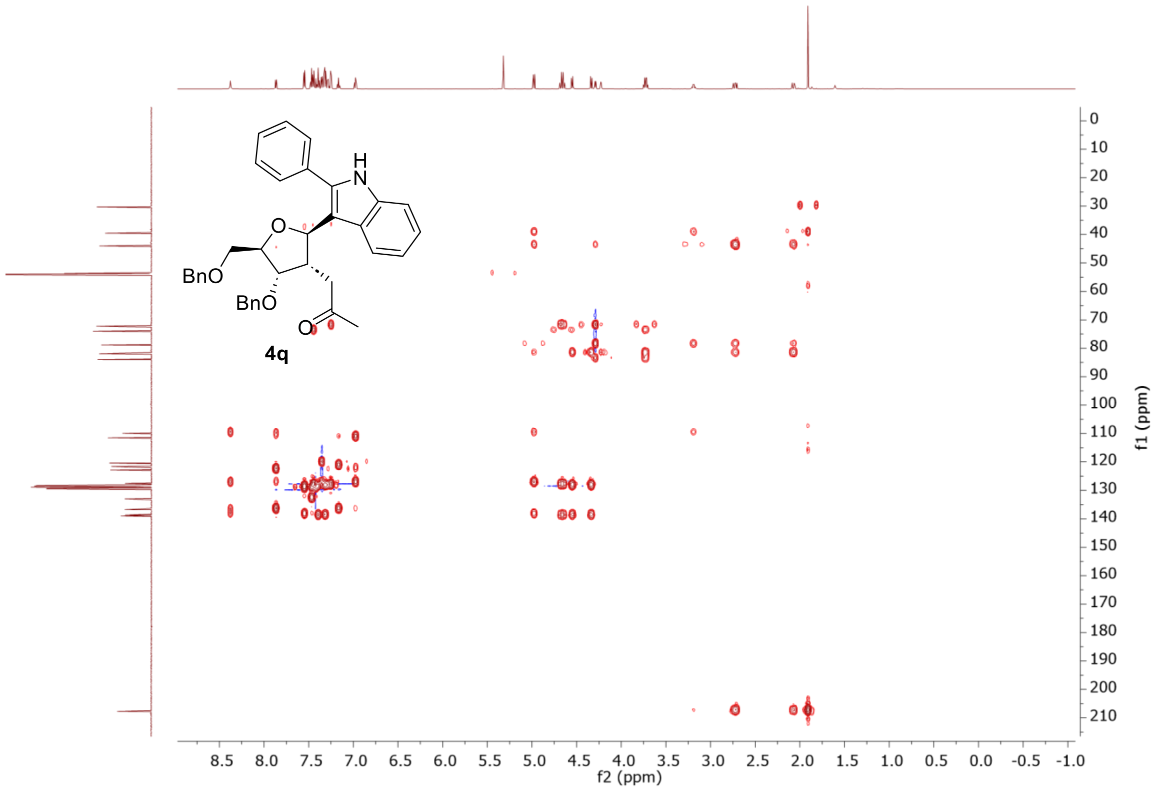

Supplementary Figure S220: HMBC spectra for 4q

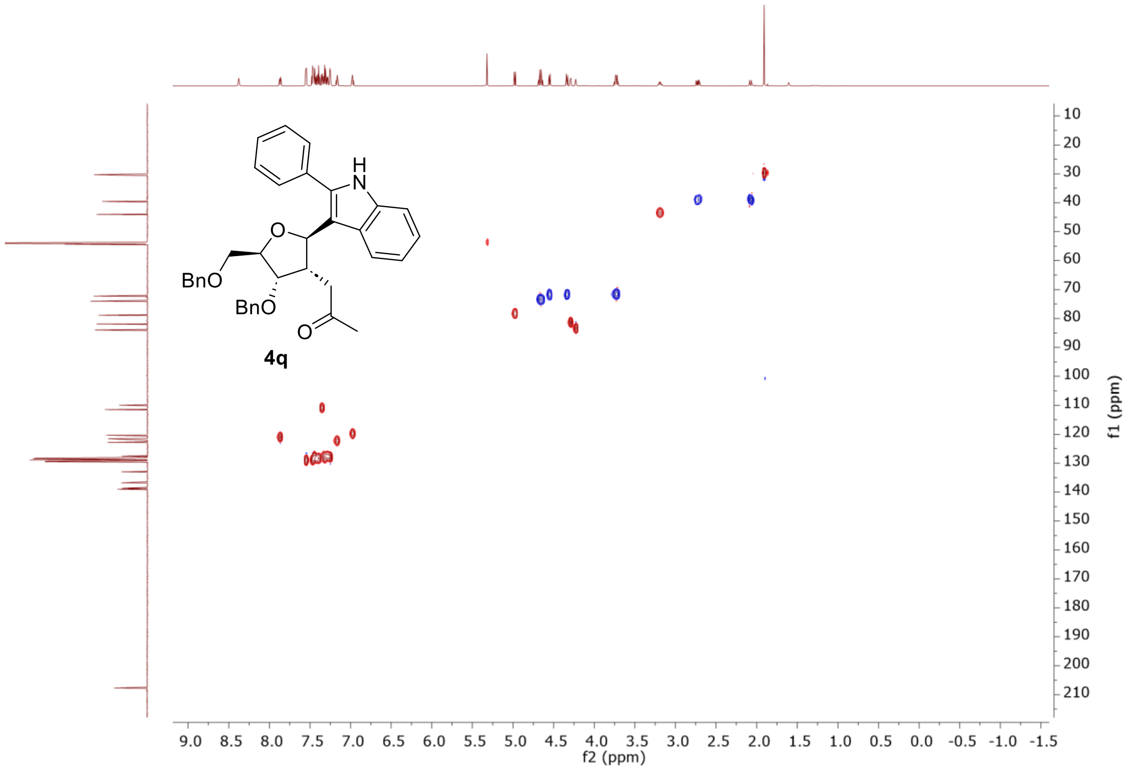

Supplementary Figure S221: HSQC spectra for 4q

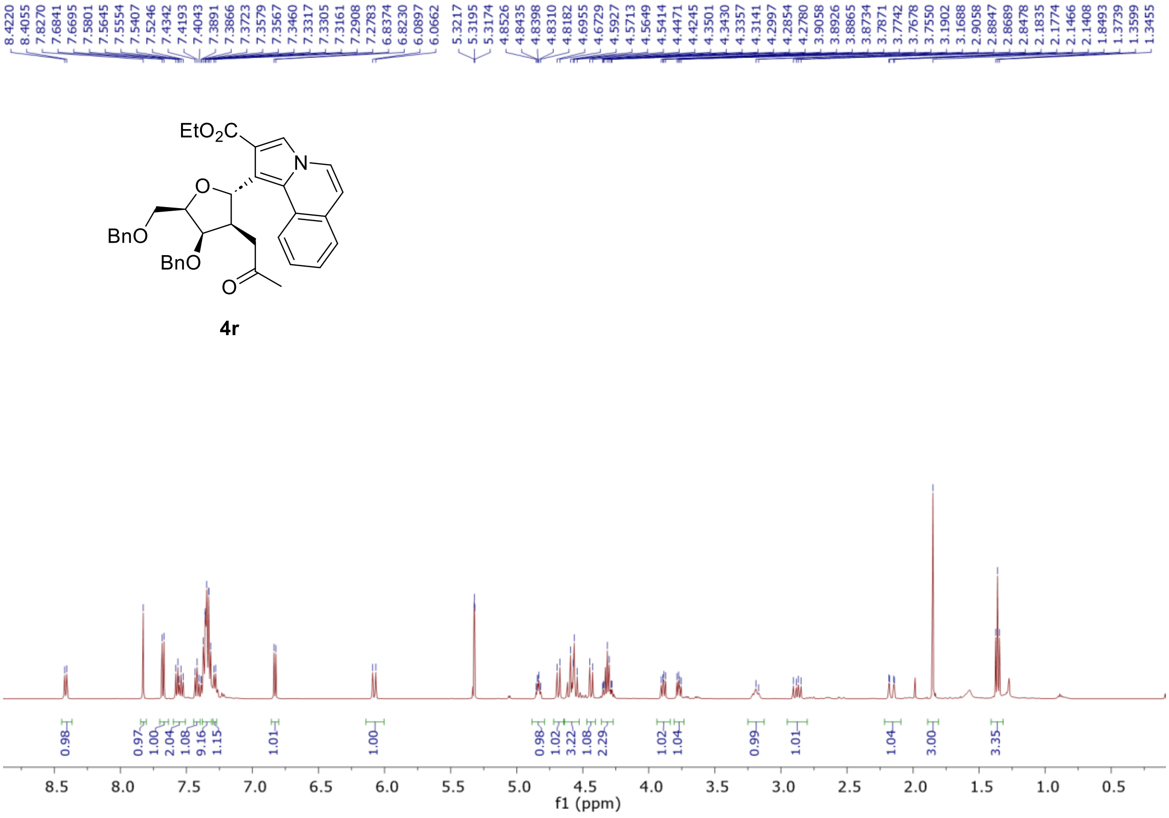

Supplementary Figure S222: ^{1}H NMR spectra for 4r

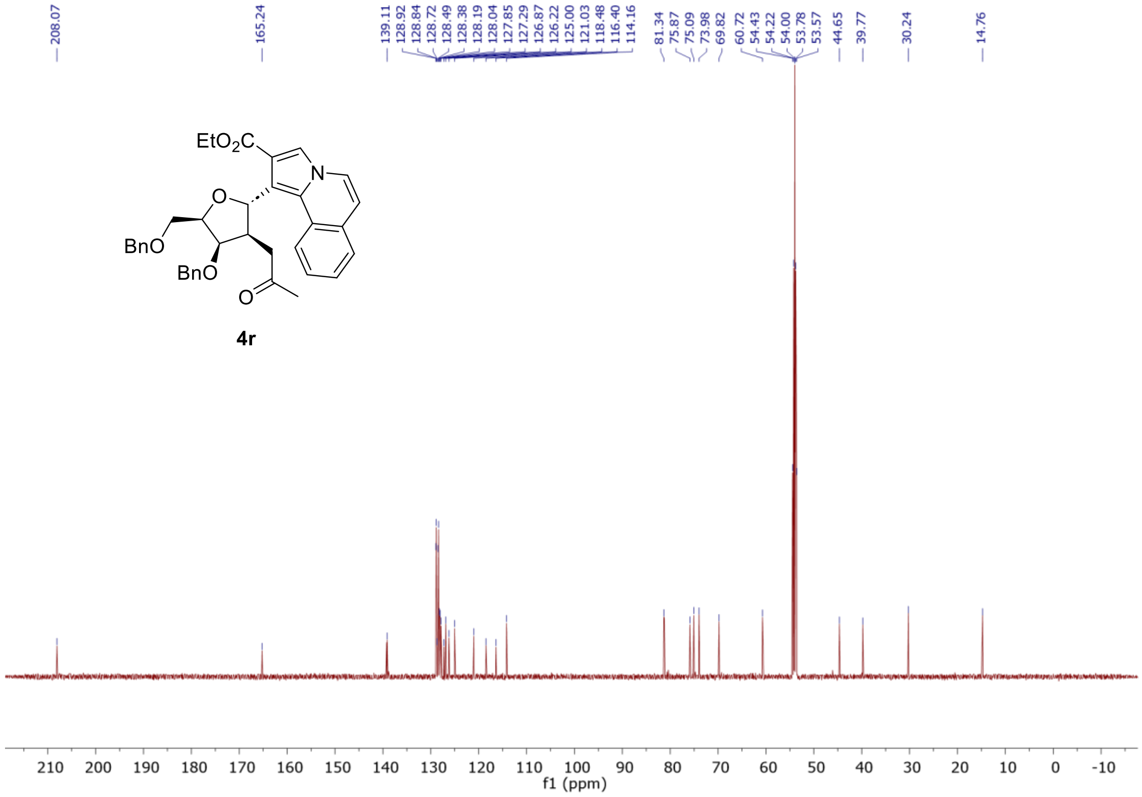

Supplementary Figure S223: ^{13}C NMR spectra for 4r

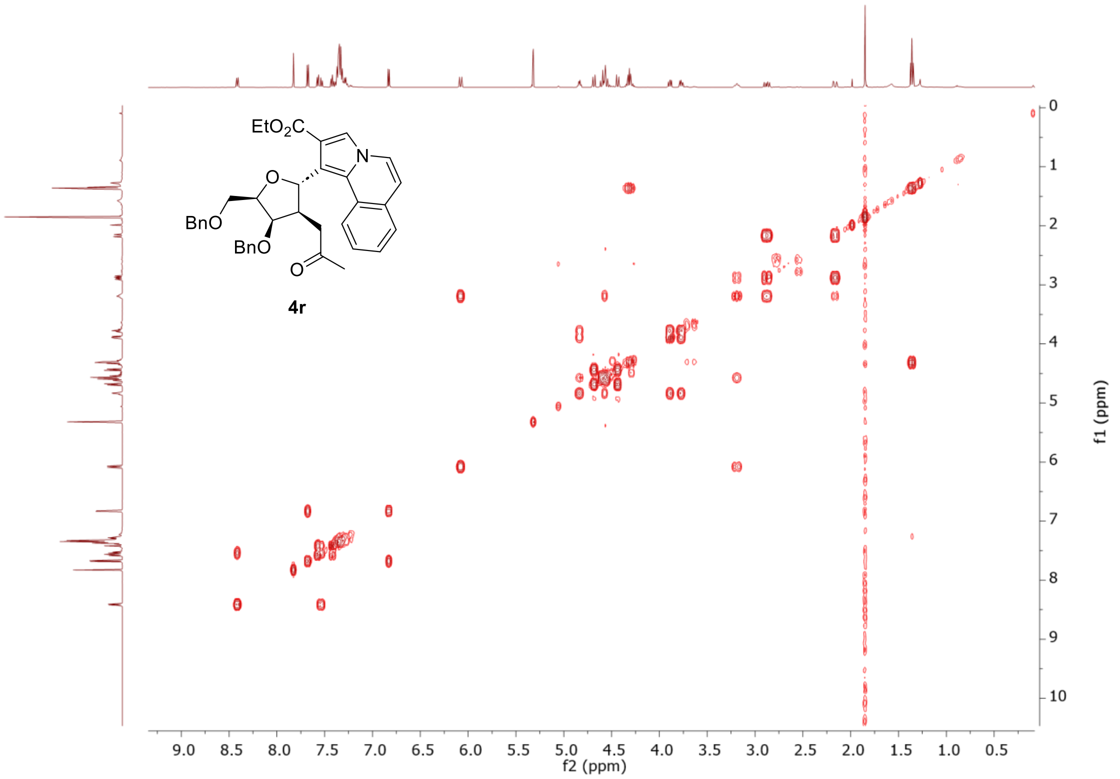

Supplementary Figure S224: COSY spectra for 4r

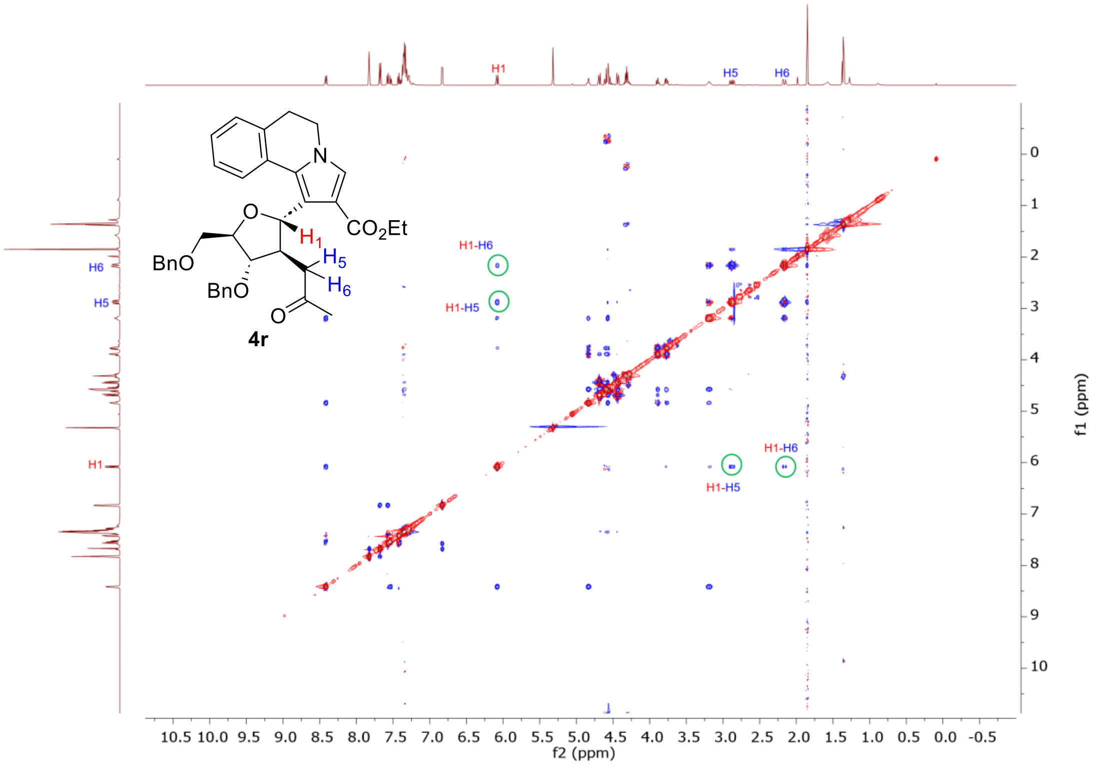

Supplementary Figure S225: NOESY spectra for 4r

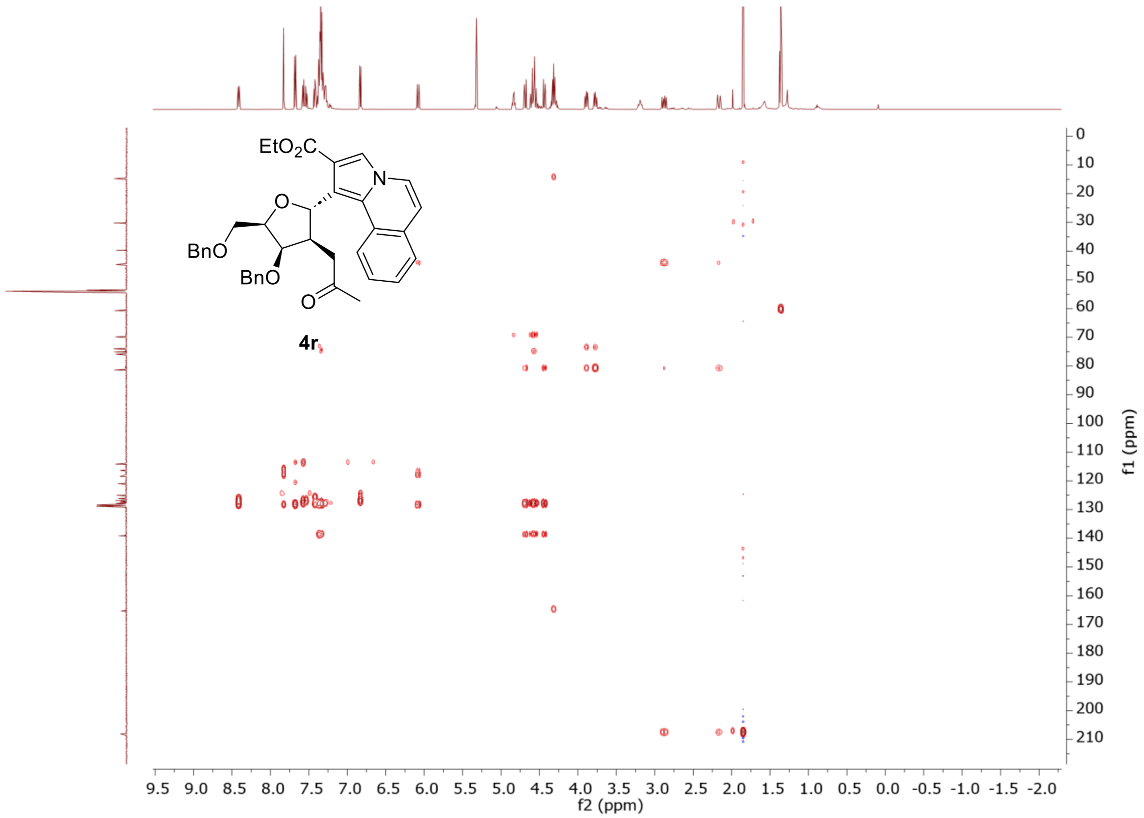

Supplementary Figure S226: HMBC spectra for 4r

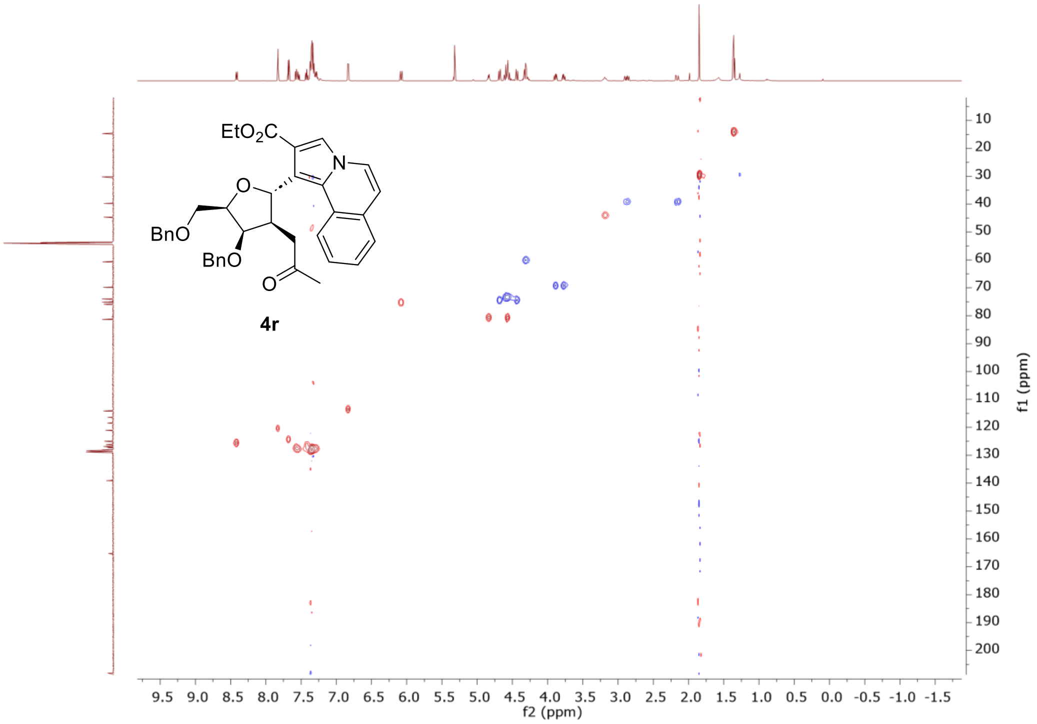

Supplementary Figure S227: HSQC spectra for 4r

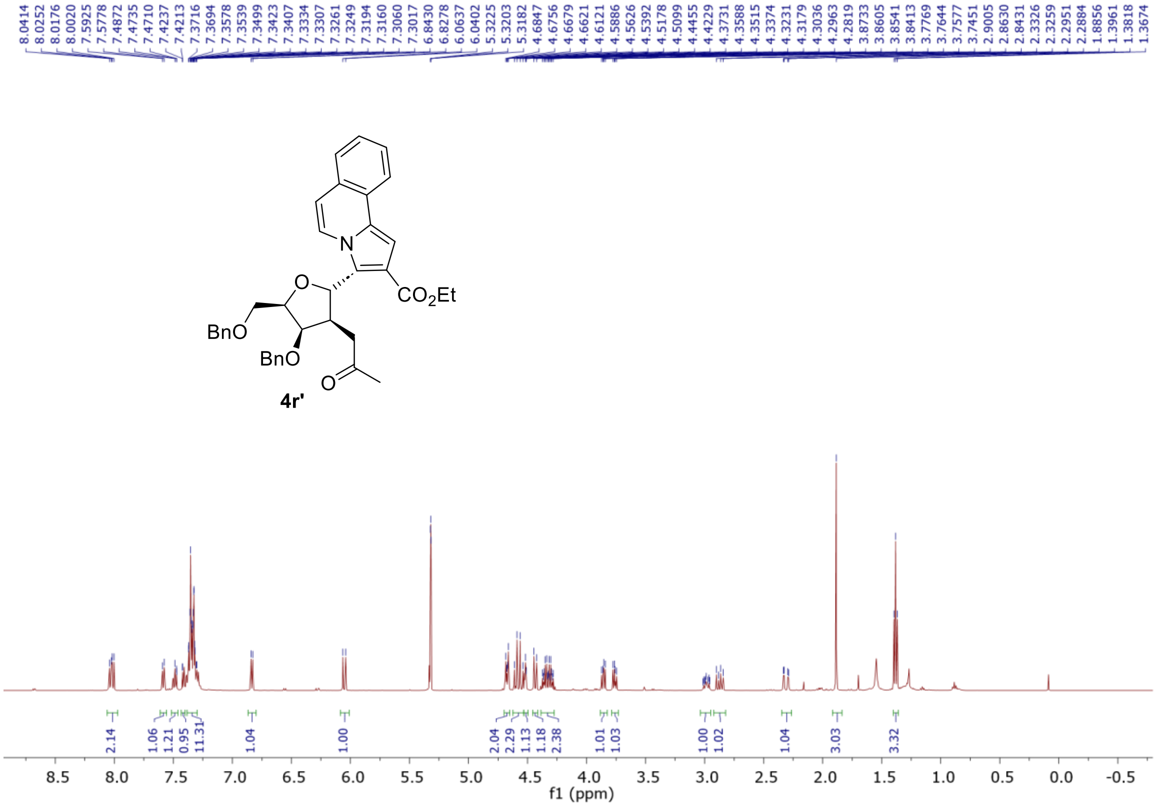

Supplementary Figure S228: ^{1}H NMR spectra for 4r'

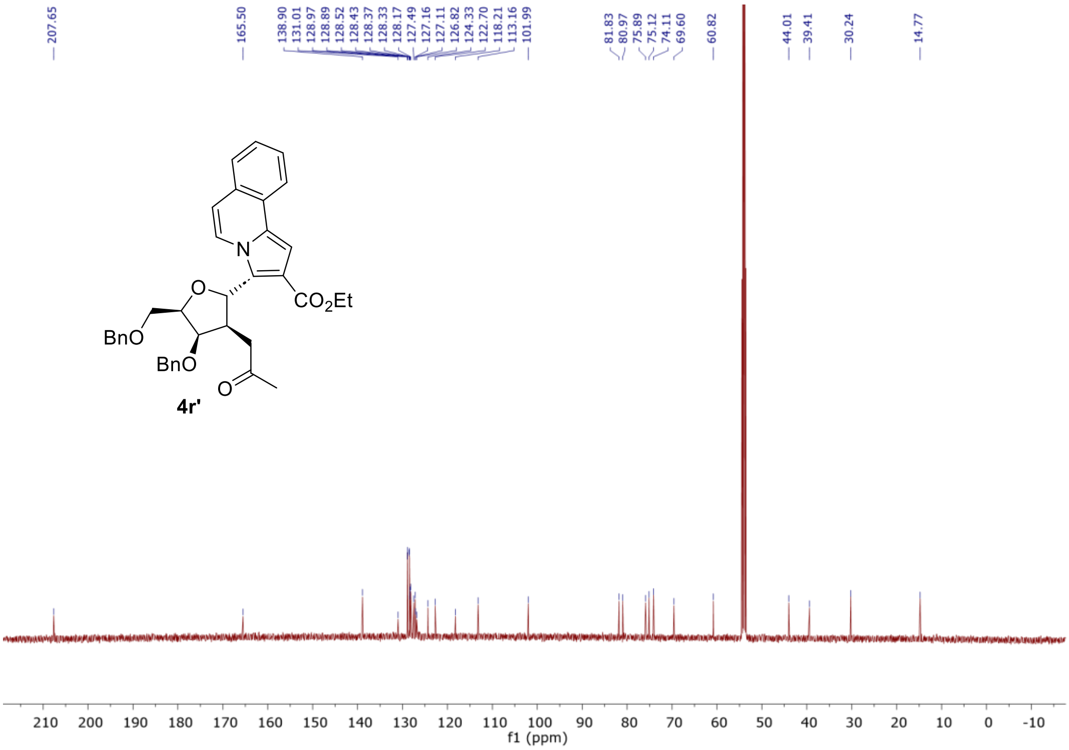

Supplementary Figure S229: ^{13}C NMR spectra for 4r'

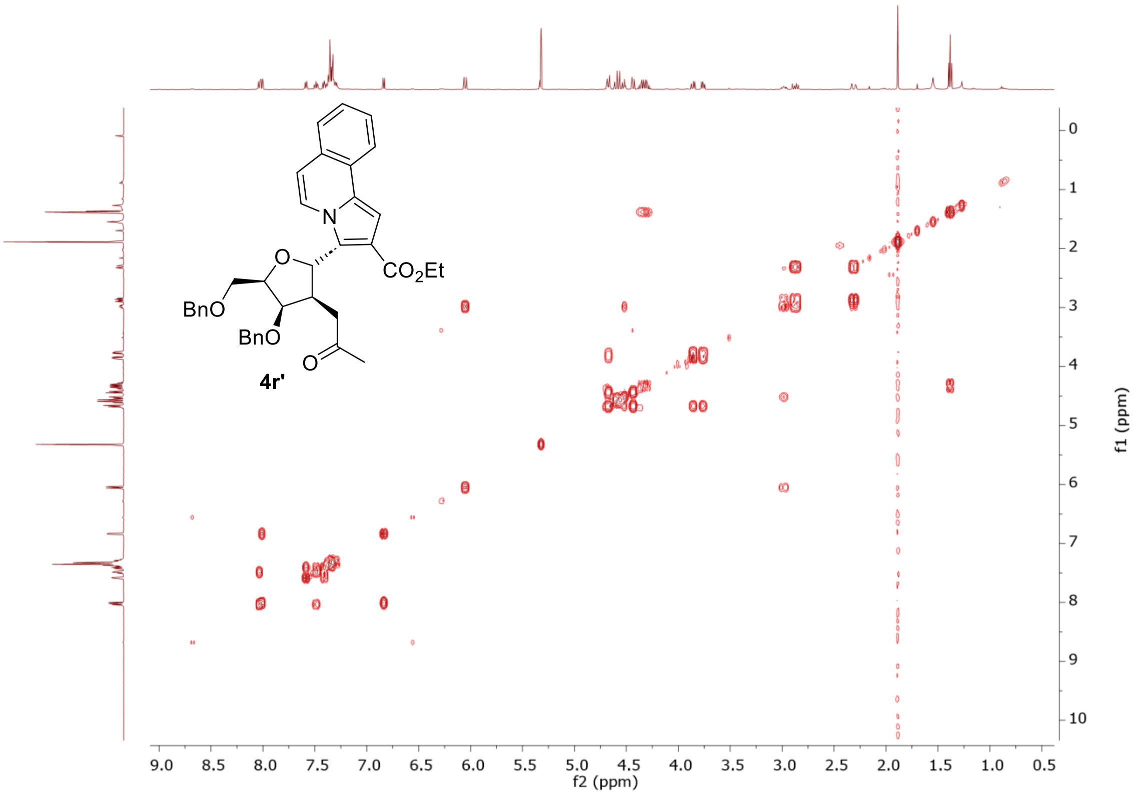

Supplementary Figure S230: COSY spectra for 4r’

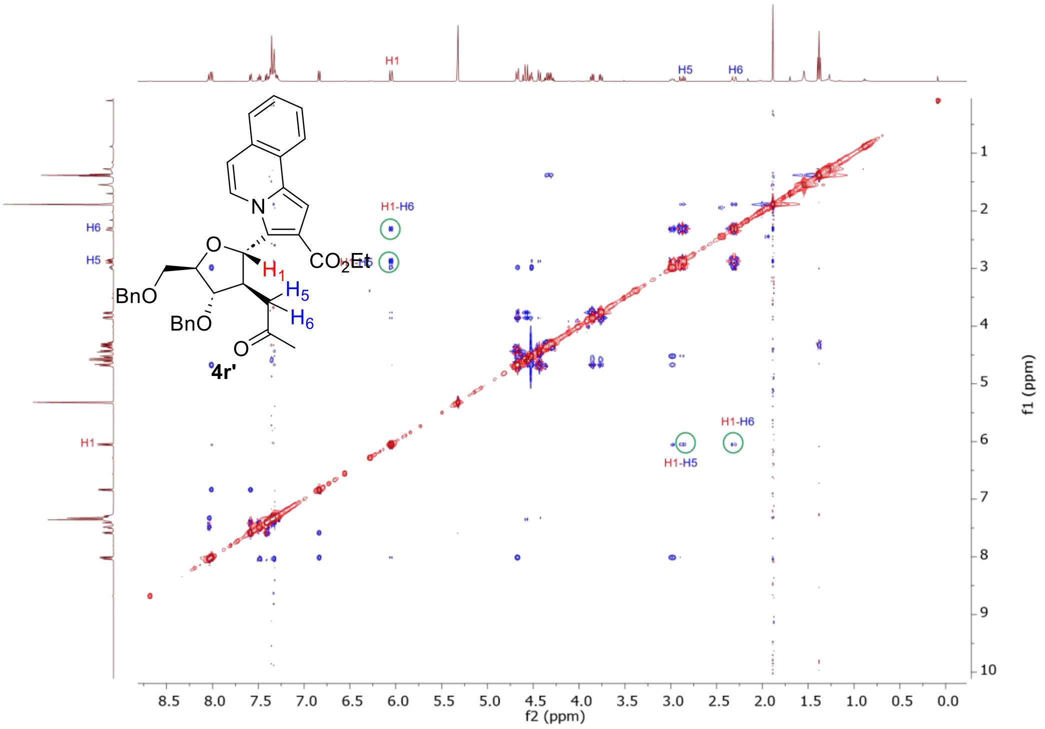

Supplementary Figure S231: NOESY spectra for 4r’

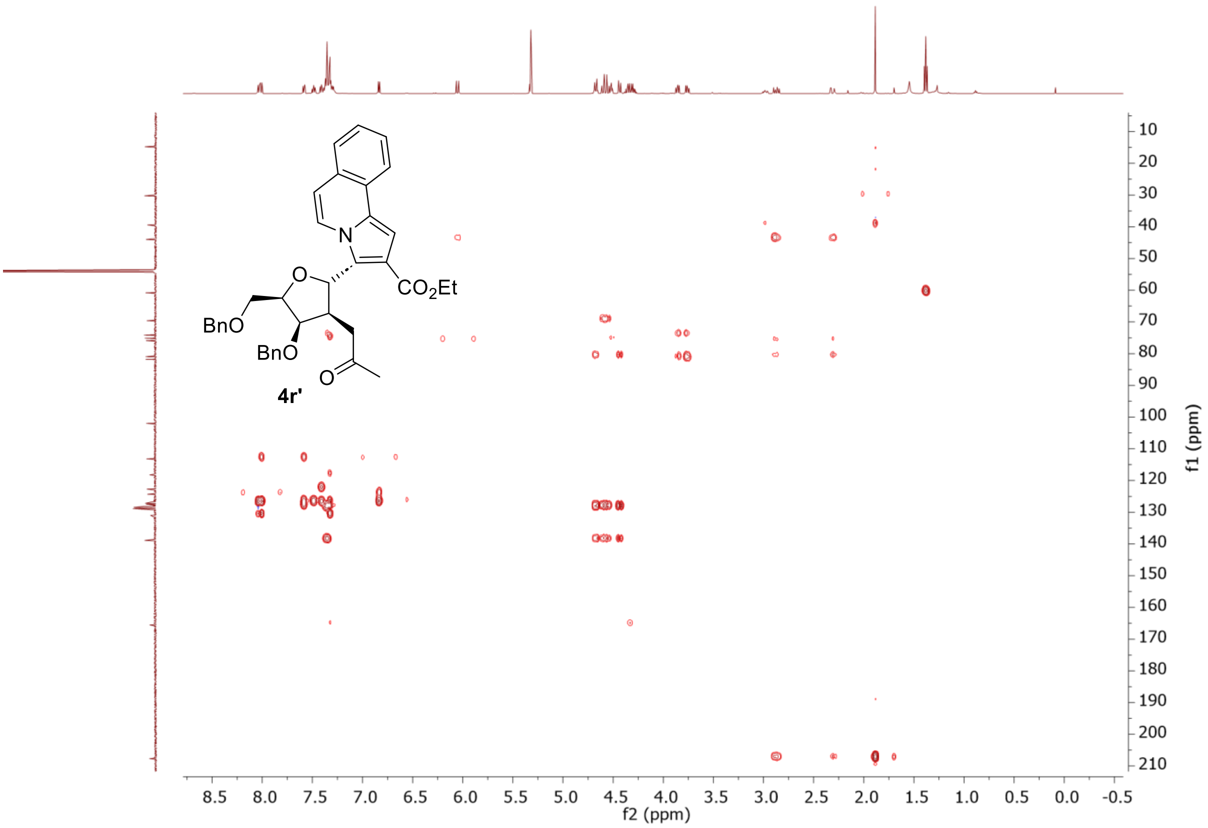

Supplementary Figure S232: HMBC spectra for 4r’

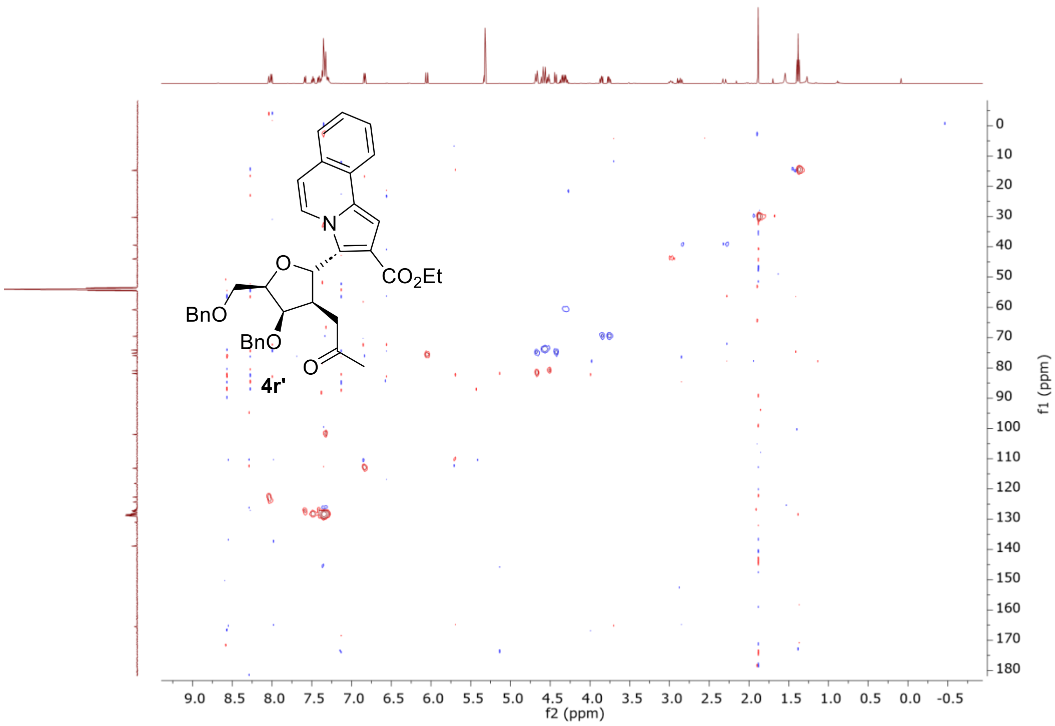

Supplementary Figure S233: HSQC spectra for 4r’

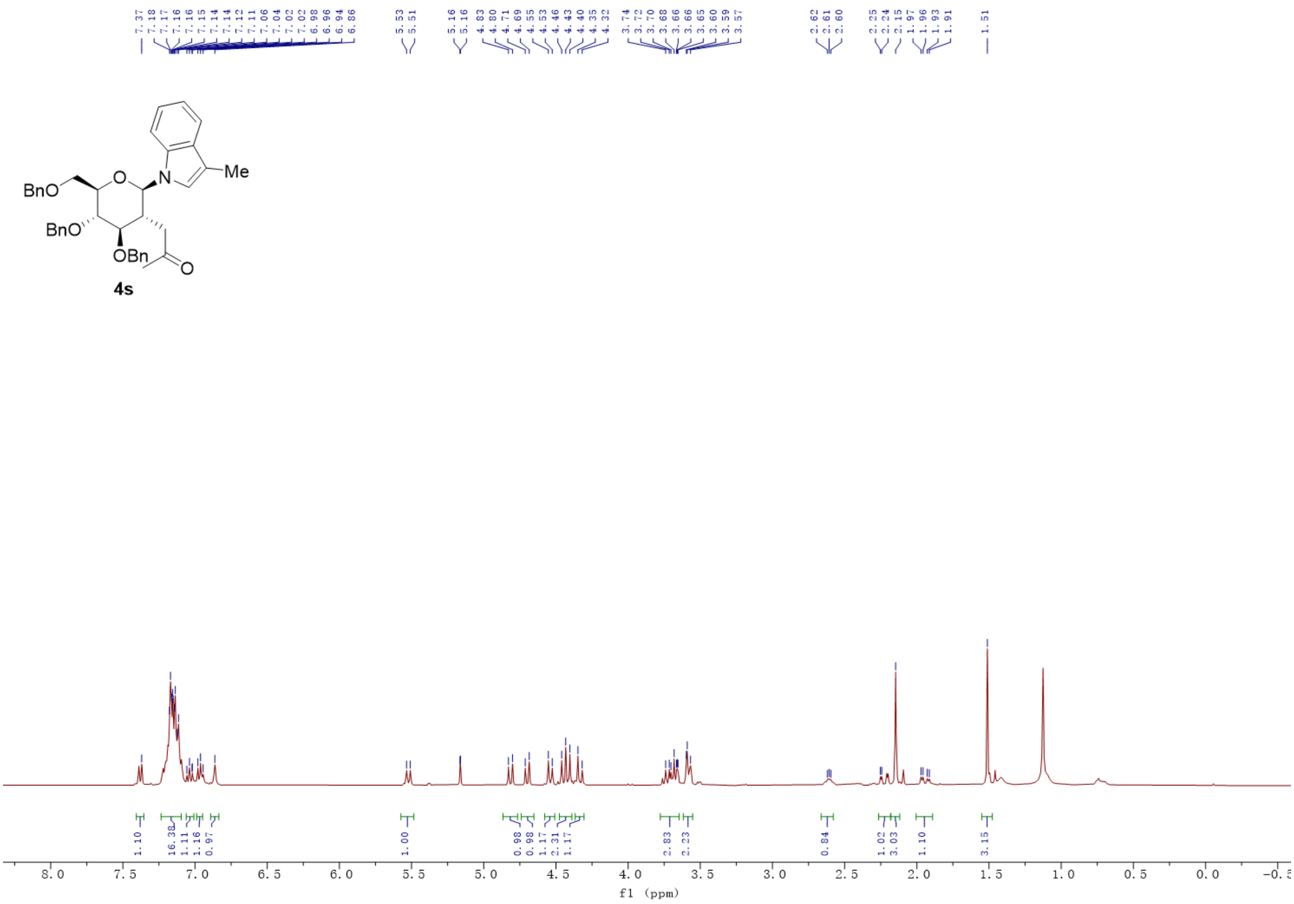

Supplementary Figure S234: ^{1}H NMR spectra for 4s

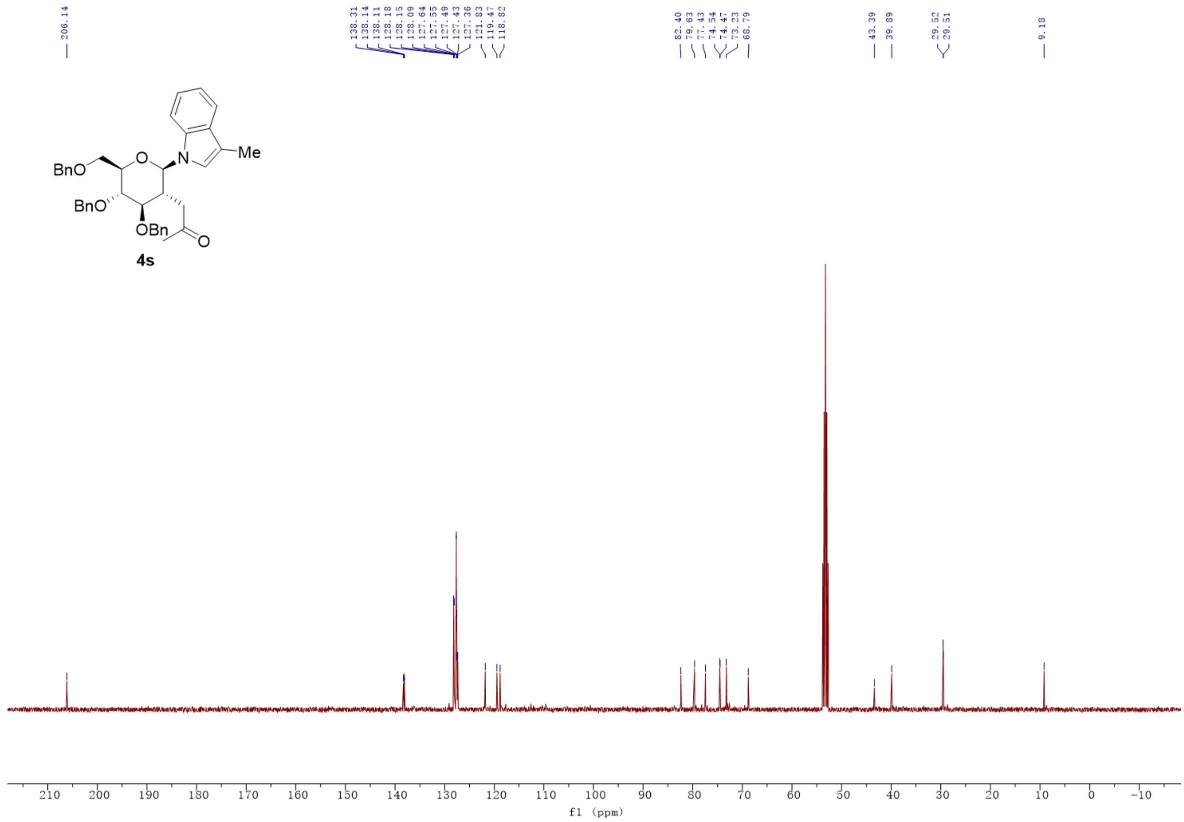

Supplementary Figure S235: ^{13}C NMR spectra for 4s

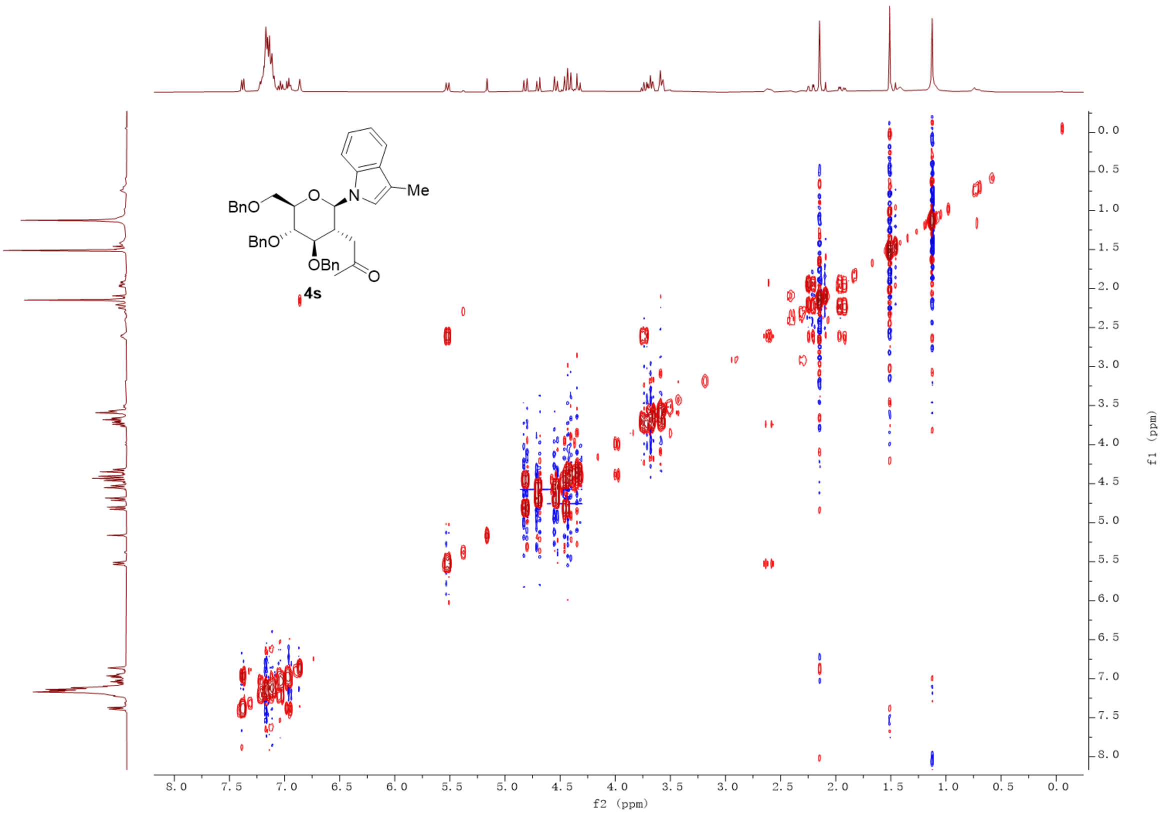

Supplementary Figure S236: COSY NMR spectra for 4s

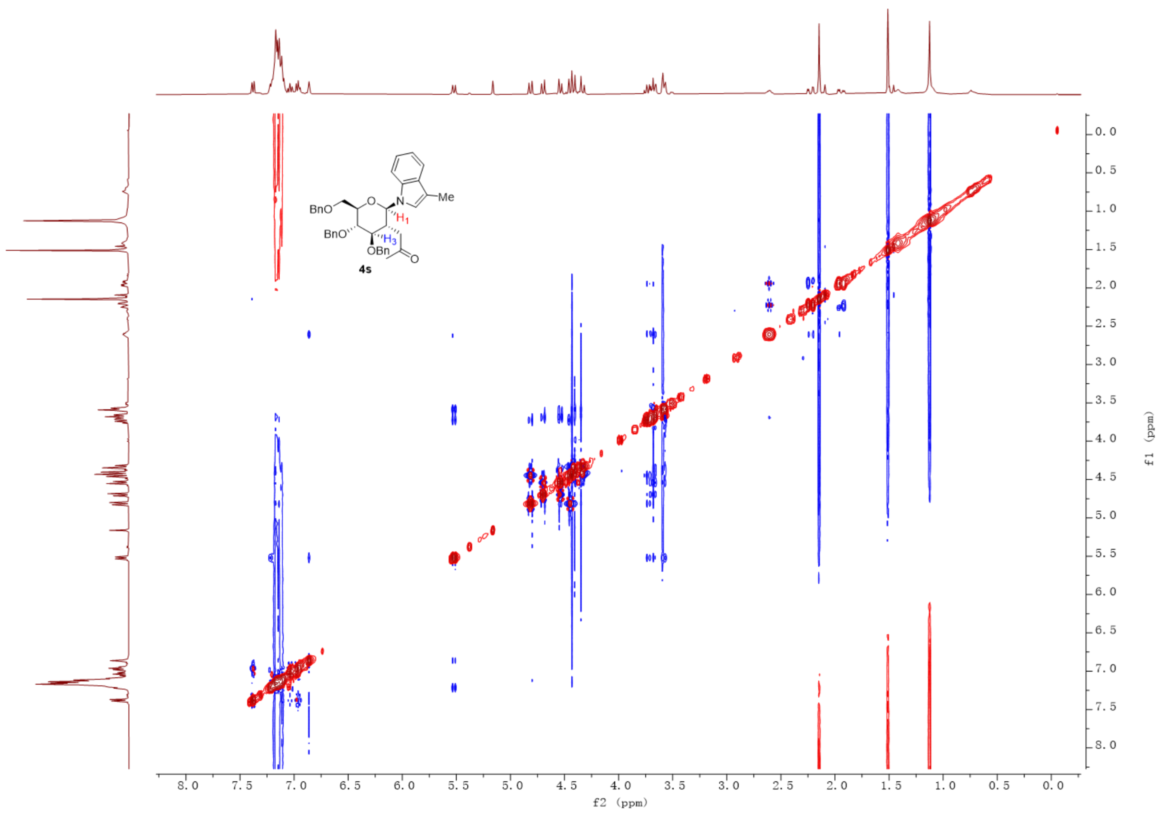

Supplementary Figure S237: NOESY NMR spectra for 4s

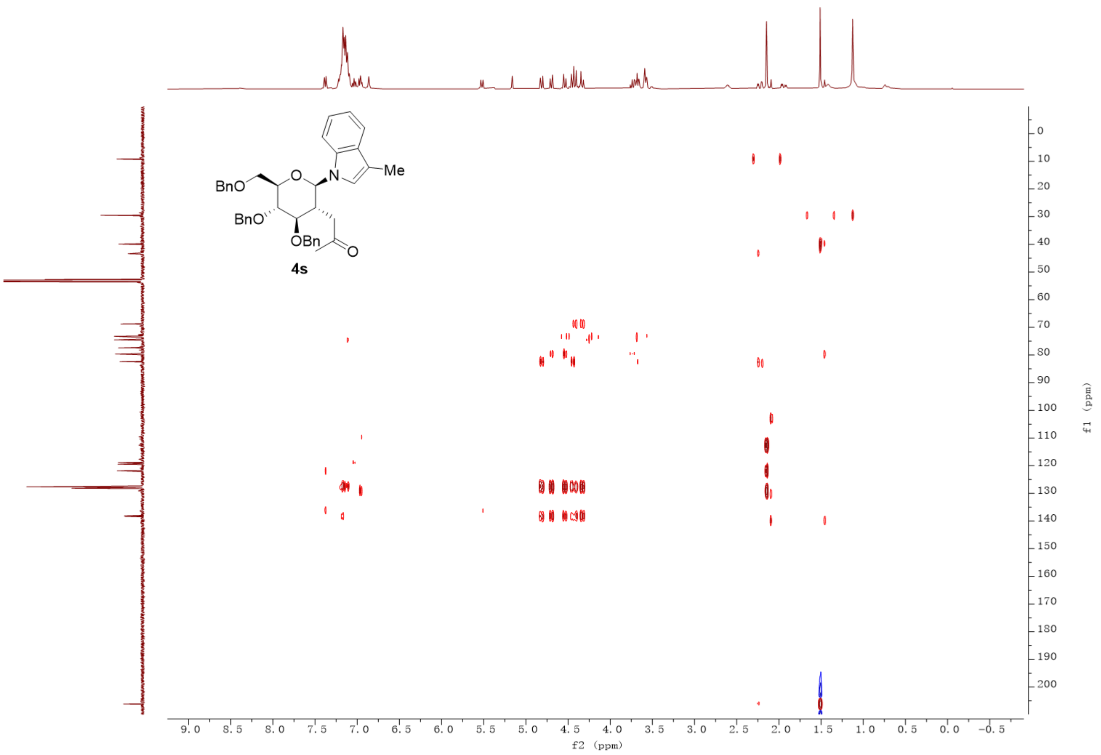

Supplementary Figure S238: HMBC NMR spectra for 4s

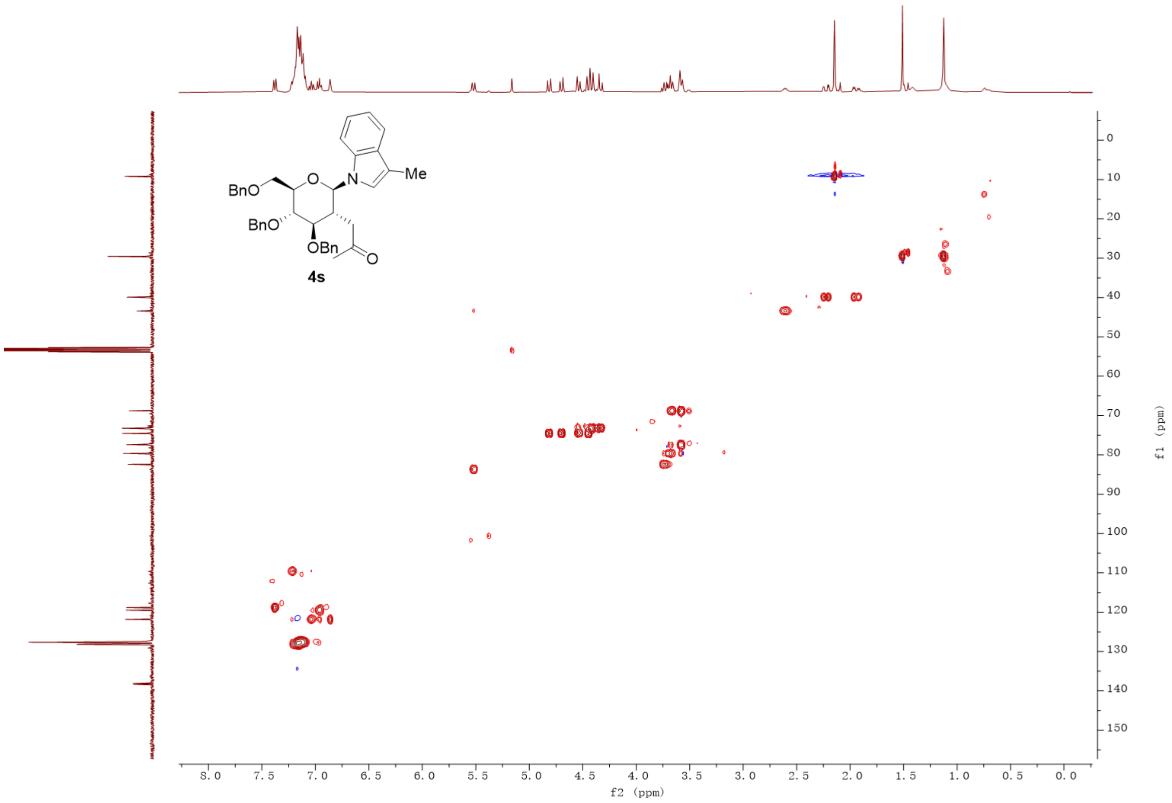

Supplementary Figure S239: HSQC NMR spectra for 4s

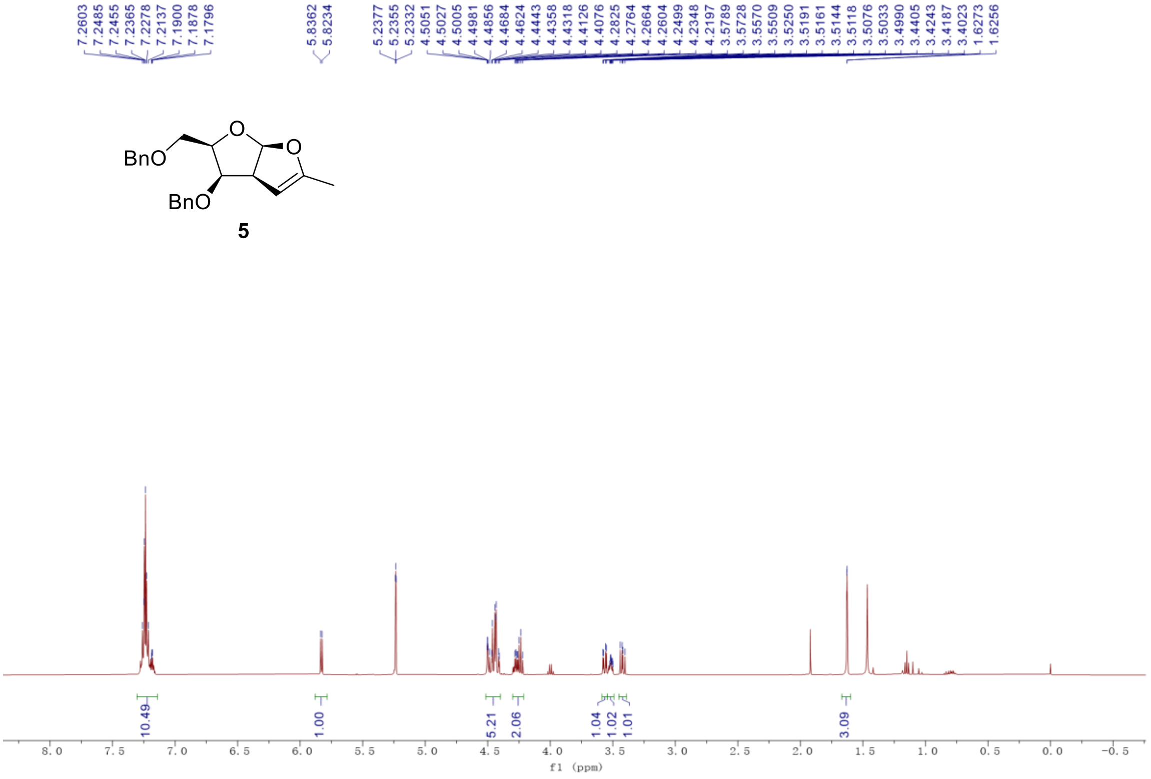

Supplementary Figure S240: ^{1}H NMR spectra for 5
